# Supplementary material for: A systematic comprehensive longitudinal evaluation of dietary factors associated with acute myocardial infarction and fatal coronary heart disease
Source: Nat Commun. 2020 Nov 27;11:6074. doi: 10.1038/s41467-020-19888-2 (PMC7699643; doi:10.1038/s41467-020-19888-2)
Supplement: Supplementary file 1 — Supplementary Information [file 41467_2020_19888_MOESM1_ESM.pdf]

A Systematic Comprehensive Longitudinal Evaluation of Dietary Factors Associated with Acute Myocardial Infarction and Fatal Coronary Heart Disease

Soodabeh Milanlouei[1†], Giulia Menichetti[1†], Yanping Li[2], Joseph Loscalzo[3], Walter C Willett[2,3], Albert-László Barabási[1,3,4*]

[1]Center for Complex Network Research, Northeastern University, Boston, USA
[2]Department of Nutrition, Harvard T.H. Chan School of Public Health, Boston, USA
[3]Channing Division of Network Medicine, Department of Medicine, Brigham and Women's Hospital, Boston, USA
[4]Center for Network Science, Central European University, Budapest, Hungary

[†]These authors contributed equally.
[*]Corresponding author. e-mail: alb@neu.edu

SUPPLEMENTARY INFORMATION

Contents

1 Supplementary Tables **3**

2 Supplementary Figures **27**

3 Supplementary Methods **33**

3.1 Participant Selection 33

3.2 Cumulative Average of Dietary Exposures 33

3.3 Multiple Testing 34

Supplementary References **37**

1 Supplementary Tables

Supplementary Table 1: **Population Characteristics.** Characteristics of the Study Participants in NHS I at Baseline.

	Control	Case
Population	60,037 (96%)	2,774 (4%)
Age (year)	52.5* (7.1✧)	56.2 (6.5)
BMI (kg/m^2)	25.1 (4.6)	26.1 (5.1)
Physical activity (MET/week)	14.2 (20.3)	13.4 (22.7)
Calorie intake (kcal/day)	1,768.5 (524.9)	1,772.8 (551.7)
Aspirin use (%)	68%	68%
Multi-vitamin use (%)	42%	40%
Vitamin E use (%)	16%	14%
Ever use of hormone therapy (%)	67%	58%
Ever smoked (%)	55%	64%
Family history of MI (%)	24%	35%
Family history of HBP (%)	49%	53%
Elevated cholesterol level (%)	6%	11%
HBP (%)	14%	26%

BMI, Body Mass Index; MI, Myocardial Infarction; HBP, High Blood Pressure
* Mean
✧ Standard deviation

Supplementary Table 2: **Descriptive Characteristics of Exposures.** s/d: serving per day.

Type	Unit	Exposure	Mean (SD)	Median (25th and 75th percentiles)
Nutrient	mcg	Alpha carotene	781.66 (654.63)	610 (402.00, 943.00)
Nutrient	mcg	Acrylamide	18.24 (9.48)	16.92 (12.01, 22.57)
Nutrient	gm	Discretionary liquid fat	9.30 (6.25)	7.97 (4.99, 11.98)
Nutrient	gm	Discretionary solid fat	38.81 (16.21)	36.65 (27.22, 48.03)
Nutrient	gm	Added sugar	41.66 (26.78)	36.04 (23.25, 53.29)
Nutrient	gm	Animal fat	36.06 (14.60)	34.1 (25.70, 44.20)
Nutrient	gm	Alanine	3.93 (1.29)	3.79 (3.02, 4.67)
Nutrient	gm	Alcohol	6.31 (10.76)	1.8 (0.00, 7.80)
Nutrient	gm	Alpha linolenic	0.91 (0.42)	0.83 (0.61, 1.13)
Nutrient	gm	Total fiber	19.06 (7.59)	18 (13.80, 23.10)
Nutrient	gm	Animal protein	59.16 (21.33)	57 (44.20, 71.50)
Nutrient	gm	Arginine	4.57 (1.50)	4.42 (3.53, 5.43)
Nutrient	gm	Aspartic acid	7.16 (2.27)	6.93 (5.56, 8.50)
Nutrient	mg	Dietary thiamine	1.33 (0.47)	1.28 (1.00, 1.61)
Nutrient	mcg	Dietary vitamin B12	7.12 (4.27)	6.3 (4.60, 8.40)
Nutrient	mcg	Total vitamin B12	10.90 (16.91)	8 (6.00, 12.00)
Nutrient	mg	Thiamine	4.22 (7.90)	1.75 (1.20, 3.19)
Nutrient	mg	Dietary riboflavin	1.77 (0.66)	1.68 (1.29, 2.16)
Nutrient	mg	Total riboflavin	4.60 (7.66)	2.33 (1.55, 3.94)
Nutrient	mg	Dietary pyridoxine	1.94 (0.68)	1.86 (1.47, 2.32)
Nutrient	mg	Total pyridoxine	8.91 (25.27)	2.5 (1.80, 4.50)
Nutrient	mg	Natural vitamin B6	1.86 (0.63)	1.78 (1.42, 2.20)
Nutrient	mg	Synthetic vitamin B6	0.14 (0.26)	0.03 (0.00, 0.18)
Nutrient	mcg	Dietary beta carotene	4,285.78 (2,645.66)	3713 (2,554.00, 5,365.00)
Nutrient	mcg	Total beta carotene	4,367.91 (2,762.11)	3737 (2,564.00, 5,441.00)
Nutrient	mcg	Beta cryptoxanthin	197.81 (129.21)	180.6 (101.80, 259.90)
Nutrient	ug	Bergamottin	216.57 (353.68)	81 (0.40, 251.70)
Nutrient	ug	Bergapten	0.98 (1.63)	0.44 (0.22, 1.02)
Nutrient	ug	Bergaptol	32.74 (94.67)	1.4 (0.00, 29.50)
Nutrient	mg	Dietary betaine	107.13 (50.83)	97.5 (72.70, 131.40)
Nutrient	mg	Choline derivative betaine	107.17 (50.84)	97.5 (72.70, 131.50)
Nutrient	mg	Sum of betaine and choline	465.81 (146.56)	450.2 (362.70, 551.40)
Nutrient	mg	Biochanin A	0.07 (0.05)	0.05 (0.02, 0.10)
Nutrient	gm	Added bran from wheat, rice, oat, corn	1.71 (5.05)	0 (0.00, 0.80)
Nutrient	gm	Natural bran	2.01 (2.27)	1.3 (0.50, 2.70)
Nutrient	mg	Caffeine	294.65 (228.43)	253 (108.00, 409.00)
Nutrient	mg	Dietary calcium	791.73 (360.88)	716 (531.00, 992.00)
Nutrient	mg	Total calcium	1,141.69 (547.34)	1068 (713.00, 1,484.00)
Nutrient	gm	Carbohydrates	214.15 (74.54)	206 (161.00, 258.00)
Nutrient	gm	Cereal fiber	4.90 (3.54)	4.1 (2.70, 6.10)
Nutrient	mg	Cholesterol	262.57 (102.49)	252 (191.00, 319.00)
Nutrient	mg	Dietary choline	349.05 (106.78)	339 (273.80, 412.90)
Nutrient	mg	Total choline (no betaine)	358.64 (115.67)	345.5 (277.50, 424.40)
Nutrient	gm	Fiber from cruciferous veg	1.12 (0.88)	0.9 (0.60, 1.50)
Nutrient	gm	Crude fiber	5.56 (2.31)	5.2 (4.00, 6.80)
Nutrient	mg	Dietary copper	1.48 (0.50)	1.4 (1.10, 1.70)
Nutrient	mg	Total copper	1.72 (0.90)	1.5 (1.20, 1.90)
Nutrient	mg	Sulfur from cysteine	315.79 (112.30)	300.4 (234.50, 382.00)
Nutrient	gm	Cystine	1.00 (0.31)	0.97 (0.78, 1.18)
Nutrient	mg	Daidzein	0.33 (0.68)	0.22 (0.11, 0.33)
Nutrient	mg	Dairy calcium	490.98 (316.05)	417.4 (261.70, 629.30)
Nutrient	gm	Dairy fat	15.08 (8.59)	13.47 (9.25, 18.87)
Nutrient	gm	Dairy protein	16.76 (9.96)	14.52 (9.60, 22.42)
Nutrient	IU	Dairy vitamin D	108.93 (97.03)	90.3 (32.70, 126.70)
Nutrient	mcg	Dihydrovitamin K	18.64 (10.88)	16.42 (10.94, 24.01)
Nutrient	mg	Dietary alpha tocopherol	9.11 (3.56)	8.57 (6.71, 10.83)
Nutrient	mg	Total vitamin E	41.63 (77.90)	11.4 (7.90, 19.70)
Nutrient	mg	Epiprogoitrin	0.02 (0.01)	0.01 (0.01, 0.03)
Nutrient	mg	Food supplemented alpha tocopherol	0.23 (1.48)	0 (0.00, 0.00)
Nutrient	gm	Asparagine	2.91 (0.92)	2.82 (2.26, 3.45)

Supplementary Table 2. cont.

Type	Unit	Exposure	Mean (SD)	Median (25th and 75th percentiles)
Nutrient	gm	Cysteine	1.39 (0.43)	1.35 (1.09, 1.65)
Nutrient	gm	Glutamine	7.27 (2.27)	7.04 (5.65, 8.66)
Nutrient	gm	Capric acid	0.34 (0.19)	0.31 (0.21, 0.43)
Nutrient	gm	Lauric acid	0.46 (0.31)	0.37 (0.26, 0.55)
Nutrient	gm	Myristic acid	2.02 (0.96)	1.86 (1.37, 2.49)
Nutrient	gm	Palmitic acid	12.15 (4.58)	11.55 (8.89, 14.77)
Nutrient	gm	Palmitoleic acid	1.31 (0.52)	1.25 (0.94, 1.61)
Nutrient	gm	Stearic acid	5.49 (2.23)	5.18 (3.91, 6.73)
Nutrient	gm	Oleic acid	21.70 (8.32)	20.6 (15.80, 26.40)
Nutrient	gm	Linoleic acid	10.43 (4.47)	9.8 (7.30, 12.80)
Nutrient	gm	Linolenic acid	1.11 (0.43)	1.05 (0.80, 1.35)
Nutrient	gm	Eicosenoic acid	0.16 (0.11)	0.14 (0.08, 0.21)
Nutrient	gm	Arachadonic acid	0.15 (0.06)	0.14 (0.11, 0.18)
Nutrient	gm	EPA fatty acid	0.08 (0.08)	0.05 (0.03, 0.11)
Nutrient	gm	22:5 fatty acid	0.02 (0.02)	0.02 (0.01, 0.03)
Nutrient	gm	DHA fatty acid	0.16 (0.12)	0.12 (0.07, 0.20)
Nutrient	gm	Butyric acid	0.42 (0.26)	0.37 (0.25, 0.53)
Nutrient	gm	Caproic acid	0.24 (0.16)	0.2 (0.13, 0.30)
Nutrient	gm	Caprylic acid	0.13 (0.08)	0.12 (0.08, 0.17)
Nutrient	mcg	Dietary folate	308.33 (121.22)	292 (224.00, 372.00)
Nutrient	mcg	Total folate	424.15 (233.34)	356 (254.00, 562.00)
Nutrient	mcg	Supplemental or fortified folic acid	155.10 (204.49)	57.3 (8.00, 247.60)
Nutrient	mg	Formononetin	0.05 (0.04)	0.04 (0.02, 0.07)
Nutrient	mg	Dietary free choline	79.38 (23.98)	77 (62.60, 93.40)
Nutrient	mg	Total free choline	81.43 (29.03)	77.5 (62.90, 94.40)
Nutrient	gm	Fruit fiber	4.52 (3.16)	3.9 (2.30, 6.00)
Nutrient	gm	Fruit carbohydrate minus juice sugar	33.09 (20.87)	29.34 (18.32, 43.30)
Nutrient	gm	Fructose	24.14 (13.03)	21.72 (15.31, 29.91)
Nutrient	ug	6'7'-Dihydroxybergamottin	269.67 (424.31)	118.3 (0.60, 263.20)
Nutrient	ug	Total furocoumarins	519.93 (838.84)	201 (2.00, 596.00)
Nutrient	mg	Genistein	0.30 (0.86)	0.15 (0.10, 0.21)
Nutrient	gm	Added germ from wheat	0.26 (1.20)	0 (0.00, 0.20)
Nutrient	gm	Natural germ	0.53 (0.61)	0.4 (0.20, 0.70)
Nutrient	mg	Glucoalyssin	0.10 (0.10)	0.06 (0.05, 0.19)
Nutrient	mg	Glucobarbarin	0.03 (0.03)	0.02 (0.01, 0.07)
Nutrient	mg	Glucoiberin	0.74 (0.88)	0.55 (0.22, 0.98)
Nutrient	mg	Glucobrassicanapin	0.11 (0.11)	0.08 (0.04, 0.23)
Nutrient	mg	Glucobrassicin	3.96 (3.50)	3.03 (1.79, 5.22)
Nutrient	mg	Glucocheirolin	0.05 (0.10)	0 (0.00, 0.11)
Nutrient	mg	Glucoerucin	0.02 (0.02)	0.01 (0.01, 0.03)
Nutrient	mg	Glucoerysolin	0.02 (0.03)	0 (0.00, 0.04)
Nutrient	mg	Glucoibervirin	0.10 (0.10)	0.08 (0.05, 0.11)
Nutrient	mg	Gluconapin	0.11 (0.15)	0.05 (0.02, 0.17)
Nutrient	mg	Gluconasturtiin	0.06 (0.05)	0.04 (0.03, 0.08)
Nutrient	mg	Glucoraphanin	2.40 (2.22)	1.59 (0.97, 4.77)
Nutrient	mg	Glucoraphenin	0.02 (0.02)	0.01 (0.01, 0.02)
Nutrient	mg	Total glucosinolates	11.83 (10.27)	9.14 (5.18, 15.66)
Nutrient	gm	Glucose	21.93 (11.71)	19.67 (14.09, 26.90)
Nutrient	gm	Glutamic acid	15.40 (4.96)	14.87 (11.88, 18.33)
Nutrient	gm	Gluten	5.43 (2.57)	5.09 (3.59, 6.87)
Nutrient	mg	Glycosolated B6	0.27 (0.11)	0.3 (0.20, 0.30)
Nutrient	mg	Glycitein	0.03 (0.13)	0.01 (0.00, 0.01)
Nutrient	gm	Glycine	3.44 (1.14)	3.31 (2.64, 4.09)
Nutrient	mg	Choline from glycerophosphocholine	55.11 (26.35)	49.42 (36.00, 67.88)
Nutrient	mg	Heme iron	1.23 (0.59)	1.1 (0.80, 1.60)
Nutrient	gm	Histidine	2.29 (0.74)	2.22 (1.77, 2.73)
Nutrient	gm	Hydroxyproline	0.37 (0.16)	0.35 (0.26, 0.46)
Nutrient	mg	4-Hydroxyglucobrassicin	0.32 (0.29)	0.21 (0.14, 0.45)
Nutrient	mcg	Supplemental iodine	17.96 (50.54)	0 (0.00, 0.00)
Nutrient	gm	Carbohydrate from intact wholegrain	3.78 (5.74)	1.97 (0.40, 5.25)

Supplementary Table 2. cont.

Type	Unit	Exposure	Mean (SD)	Median (25th and 75th percentiles)
Nutrient	mg	Dietary iron	13.47 (6.00)	12.3 (9.80, 15.70)
Nutrient	mg	Total iron	19.40 (15.90)	14.1 (10.50, 21.90)
Nutrient	gm	Isoleucine	3.79 (1.23)	3.66 (2.92, 4.52)
Nutrient	gm	Sugar from fruit juice	10.62 (10.52)	8.2 (2.47, 16.19)
Nutrient	mg	Dietary potassium	3,227.13 (998.80)	3126 (2,517.00, 3,829.00)
Nutrient	mg	Total potassium	3,277.43 (1,022.49)	3173 (2,550.00, 3,893.00)
Nutrient	gm	Lactose	14.69 (12.28)	12.5 (5.66, 17.91)
Nutrient	gm	Dietary long chain fatty acid	0.26 (0.21)	0.2 (0.12, 0.34)
Nutrient	gm	Total long chain fatty acid	0.26 (0.21)	0.2 (0.12, 0.34)
Nutrient	gm	Carbohdyrate from legumes	5.16 (4.09)	4.45 (2.54, 6.53)
Nutrient	gm	Fiber from legumes	0.81 (0.89)	0.7 (0.30, 1.00)
Nutrient	gm	Leucine	6.26 (2.02)	6.05 (4.82, 7.46)
Nutrient	mcg	Lutein and zeaxanthin	3,122.07 (2,312.92)	2675 (1,776.00, 3,809.00)
Nutrient	mcg	Lycopene	7,258.96 (4,904.80)	6100 (4,264.00, 8,848.00)
Nutrient	gm	Lysine	5.77 (1.92)	5.57 (4.41, 6.89)
Nutrient	mg	Dietary magnesium	310.25 (100.08)	299 (239.00, 368.00)
Nutrient	mg	Total magnesium	321.47 (105.69)	309 (246.00, 383.00)
Nutrient	gm	Maltose	1.82 (1.00)	1.63 (1.13, 2.29)
Nutrient	mg	Sulfur from methionine	418.82 (139.86)	404.5 (321.30, 498.50)
Nutrient	gm	Methionine	1.92 (0.64)	1.86 (1.47, 2.30)
Nutrient	mg	4-Methoxyglucobrassicin	0.44 (0.42)	0.35 (0.19, 0.60)
Nutrient	gm	Carbohydrate from milled wholegrain	6.78 (8.95)	3.44 (1.59, 8.34)
Nutrient	mg	Dietary manganese	2.79 (1.14)	2.6 (2.00, 3.40)
Nutrient	mg	Total manganese	4.11 (8.50)	2.8 (2.00, 3.80)
Nutrient	gm	Total MUFA	23.82 (8.98)	22.6 (17.40, 29.00)
Nutrient	gm	Animal MUFA	12.84 (5.28)	12.14 (9.05, 15.84)
Nutrient	gm	Plant MUFA	10.61 (5.42)	9.65 (6.82, 13.27)
Nutrient	mg	Sodium	3,125.43 (1,438.23)	2857 (2,134.00, 3,793.00)
Nutrient	mg	Napoleiferin	0.08 (0.08)	0.04 (0.04, 0.13)
Nutrient	gm	Natural sugar	59.99 (28.23)	56 (39.68, 76.07)
Nutrient	mg	Neoglucobrassicin	0.98 (0.87)	0.66 (0.42, 1.76)
Nutrient	mg	Total niacin	38.86 (33.01)	26.7 (19.30, 41.90)
Nutrient	mg	Dietary niacin	21.34 (6.85)	20.5 (16.60, 25.20)
Nutrient	mg	Nitrate	156.16 (95.85)	140 (95.00, 191.00)
Nutrient	gm	Total dietary omega 3 (no alpha 18:3)	0.23 (0.20)	0.18 (0.10, 0.31)
Nutrient	gm	Total dietary omega 3	1.31 (0.53)	1.23 (0.93, 1.59)
Nutrient	gm	Dietary omega 6 (no gamma 18:3)	10.58 (4.54)	9.89 (7.34, 13.00)
Nutrient	gm	Total omega 3	1.31 (0.53)	1.23 (0.93, 1.59)
Nutrient	gm	Omega 3 (20:5+22:6, no alpha 18:3)	0.23 (0.20)	0.18 (0.10, 0.31)
Nutrient	mg	Oxalate	179.78 (121.06)	159.2 (106.60, 218.40)
Nutrient	mg	Pantothenic acid	11.49 (13.22)	5.9 (4.10, 14.20)
Nutrient	mg	Choline from phosphocholine	15.91 (6.35)	14.93 (11.29, 19.58)
Nutrient	mg	Dietary phosphorous	1,226.03 (409.37)	1177 (927.00, 1,477.00)
Nutrient	mg	Total phospherous	1,228.40 (409.80)	1180 (929.00, 1,480.00)
Nutrient	gm	Phenylalanine	3.44 (1.09)	3.33 (2.66, 4.09)
Nutrient	mg	Phytate	768.46 (357.62)	704 (526.00, 934.00)
Nutrient	gm	Total polyunsaturated fat	12.10 (4.94)	11.4 (8.60, 14.80)
Nutrient	gm	Carbohydrate from potato	15.62 (11.21)	15.17 (6.87, 20.10)
Nutrient	mg	Proanthocyanidin, 4-6mers	23.75 (16.73)	20.11 (12.28, 31.10)
Nutrient	mg	Proanthocyanidin, 7-10mers	17.63 (13.36)	14.43 (8.52, 23.15)
Nutrient	gm	Proline	4.85 (1.58)	4.68 (3.71, 5.82)
Nutrient	mg	Proanthocyanidin, dimers	20.09 (14.05)	16.76 (10.29, 26.33)
Nutrient	mg	Progoitrin	0.40 (0.58)	0.17 (0.08, 0.66)
Nutrient	mg	Proanthocyanidin, monomers	22.25 (23.54)	13.57 (7.56, 27.36)
Nutrient	mg	Proanthocyanidin, polymers	37.31 (29.32)	31.31 (18.47, 48.69)
Nutrient	mg	Sum of sulfur from methionine and cysteine	734.61 (245.28)	709 (561.00, 879.00)
Nutrient	gm	Protein	22.16 (7.87)	21.2 (16.60, 26.60)
Nutrient	mg	Proanthocyanidin, trimers	8.99 (5.45)	7.94 (5.17, 11.61)
Nutrient	mg	Dietary choline from phosphatidylcholine	177.91 (63.77)	171.3 (134.00, 213.00)
Nutrient	mg	Choline from phosphatidylcholine	185.46 (73.53)	174.5 (135.70, 220.10)

Supplementary Table 2. cont.

Type	Unit	Exposure	Mean (SD)	Median (25th and 75th percentiles)
Nutrient	gm	Carbohydrate from refined grain	47.70 (22.43)	44.29 (31.77, 60.08)
Nutrient	mcg	Dietary retinol	2,091.16 (1,519.12)	1749 (1,134.00, 2,536.00)
Nutrient	mcg	Total retinol	4,424.88 (4,750.47)	2668 (1,494.00, 6,283.00)
Nutrient	gm	Total saturated fat	23.06 (8.88)	21.9 (16.80, 28.10)
Nutrient	mcg	Supplemental selenium	3.77 (18.18)	0 (0.00, 0.00)
Nutrient	gm	Serine	3.49 (1.11)	3.38 (2.69, 4.16)
Nutrient	mg	Sinigrin	1.63 (2.09)	0.96 (0.30, 2.46)
Nutrient	mg	Choline from sphingomyelin	20.74 (7.89)	19.85 (15.40, 24.97)
Nutrient	gm	Starch	66.66 (26.51)	63.8 (48.10, 82.00)
Nutrient	gm	Sucrose	37.82 (18.91)	34.79 (24.55, 47.58)
Nutrient	gm	Total sugar	100.39 (44.55)	94.2 (68.90, 124.70)
Nutrient	mg	Dietary sulfur	1,088.93 (313.87)	1063 (868.00, 1,280.00)
Nutrient	gm	Carbohydrate from wholegrain	10.56 (11.43)	6.87 (3.08, 14.37)
Nutrient	gm	Trans 16:1	0.17 (0.07)	0.16 (0.12, 0.21)
Nutrient	gm	Trans 18:1	2.75 (1.37)	2.51 (1.77, 3.50)
Nutrient	gm	Trans 18:2	0.40 (0.21)	0.37 (0.26, 0.51)
Nutrient	gm	Taurine	0.15 (0.07)	0.14 (0.10, 0.19)
Nutrient	gm	Total fat	64.54 (23.03)	61.6 (48.10, 77.90)
Nutrient	gm	Threonine	3.20 (1.04)	3.1 (2.47, 3.82)
Nutrient	gm	Isoflavones (no biochanin A or formononetin)	0.65 (1.66)	0.37 (0.23, 0.55)
Nutrient	gm	Total isoflavones	0.77 (1.67)	0.49 (0.33, 0.69)
Nutrient	mg	Total anthocyanidins	14.74 (17.21)	10.77 (5.09, 19.35)
Nutrient	mg	Total flavan-3-ols	53.09 (68.33)	24 (13.00, 70.70)
Nutrient	mg	Total flavanones	46.75 (40.17)	40.41 (15.28, 64.47)
Nutrient	mg	Total flavonoids	363.81 (320.38)	260.9 (158.00, 441.70)
Nutrient	mg	Total flavones	2.40 (1.55)	2.2 (1.25, 3.15)
Nutrient	mg	Total flavonols	14.97 (10.25)	12.15 (8.10, 18.12)
Nutrient	mg	Total theaflavins and polymers proanthocyanidis	229.28 (234.03)	149 (80.10, 280.10)
Nutrient	gm	Total trans fat	2.77 (1.39)	2.5 (1.78, 3.48)
Nutrient	gm	Tryptophan	0.93 (0.30)	0.9 (0.72, 1.11)
Nutrient	gm	Tyrosine	2.84 (0.92)	2.75 (2.18, 3.39)
Nutrient	mg	Apigenin	0.37 (0.44)	0.26 (0.14, 0.43)
Nutrient	mg	Alpha tocotrienol	0.30 (0.18)	0.26 (0.18, 0.38)
Nutrient	mg	Beta tocotrienol	0.48 (0.48)	0.31 (0.20, 0.57)
Nutrient	mg	Beta tocopherol	0.23 (0.15)	0.2 (0.15, 0.28)
Nutrient	mg	Catechin	8.84 (6.61)	7.38 (4.45, 11.45)
Nutrient	mg	Cyanidin	3.89 (3.62)	3.12 (1.54, 5.06)
Nutrient	mg	Delphinidin	2.33 (3.95)	0.76 (0.39, 3.36)
Nutrient	mg	Delta tocotrienol	0.01 (0.02)	0 (0.00, 0.01)
Nutrient	mg	Delta tocopherol	2.04 (1.03)	1.87 (1.31, 2.57)
Nutrient	mg	Epicatechin	9.44 (6.96)	7.69 (4.53, 12.42)
Nutrient	mg	Epicatechin 3-gallate	8.18 (14.27)	1.14 (0.02, 11.20)
Nutrient	mg	Epigallocatechin	11.78 (19.26)	2.58 (0.79, 16.82)
Nutrient	mg	Eriodictyol	0.01 (0.02)	0 (0.00, 0.01)
Nutrient	mg	Gallocatechin	1.71 (2.92)	0.29 (0.02, 2.52)
Nutrient	mg	Gamma tocotrienol	0.14 (0.11)	0.11 (0.07, 0.18)
Nutrient	mg	Gamma tocopherol	7.57 (3.38)	7.06 (5.17, 9.39)
Nutrient	mg	Hesperetin	30.27 (28.98)	23.93 (7.01, 49.22)
Nutrient	mg	Isorhamnetin	0.10 (0.13)	0.07 (0.02, 0.12)
Nutrient	mg	Kaempferol	3.13 (3.44)	1.81 (0.94, 3.88)
Nutrient	mg	Luteolin	2.03 (1.41)	1.84 (0.96, 2.72)
Nutrient	mg	Malvidin	3.54 (5.45)	1.56 (0.50, 4.80)
Nutrient	mg	Myricetin	1.36 (1.16)	0.99 (0.63, 1.64)
Nutrient	mg	Naringenin	16.48 (18.83)	10.91 (5.47, 21.30)
Nutrient	mg	Pelargonidin	2.95 (4.17)	2.03 (1.88, 3.30)
Nutrient	mg	Peonidin	0.63 (0.97)	0.26 (0.09, 0.88)
Nutrient	mg	Petunidin	1.31 (2.20)	0.43 (0.21, 1.89)
Nutrient	mg	Quercetin	10.38 (6.22)	8.92 (6.02, 13.07)
Nutrient	mg	Flavonoids (no proanthocyanidins)	252.37 (293.85)	137.3 (76.30, 320.55)
Nutrient	mg	Theaflavin	2.20 (3.84)	0.3 (0.00, 3.00)

Supplementary Table 2. cont.

Type	Unit	Exposure	Mean (SD)	Median (25th and 75th percentiles)
Nutrient	mg	Thearubigins	113.06 (197.75)	15 (0.00, 154.00)
Nutrient	mg	Dietary tocopherols	16.50 (6.50)	15.57 (11.89, 20.01)
Nutrient	gm	Valine	4.15 (1.34)	4.02 (3.20, 4.95)
Nutrient	gm	Vegetable fiber	6.68 (3.23)	6.1 (4.50, 8.20)
Nutrient	gm	Vegetable fat	28.48 (13.25)	26.3 (19.10, 35.30)
Nutrient	IU	Dietary vitamin A	11,572.67 (6,703.76)	10088 (7,140.00, 14,414.00)
Nutrient	IU	Total vitamin A	14,043.27 (8,545.85)	12158 (8,326.50, 17,599.50)
Nutrient	mg	Dietary vitamin C	161.71 (85.17)	148 (104.00, 201.00)
Nutrient	mg	Total vitamin C	354.56 (366.57)	213 (134.00, 398.00)
Nutrient	IU	Dietary vitamin D	217.77 (133.83)	188.7 (124.00, 288.50)
Nutrient	IU	Total vitamin D	358.70 (258.26)	291.3 (159.60, 518.30)
Nutrient	mcg	Dietary phylloquinone vitamin K1	188.13 (114.23)	166.9 (117.60, 230.55)
Nutrient	mcg	Menaquinone vitamin K2 (4 isoprenoid residues)	21.89 (10.62)	20.48 (14.64, 26.68)
Nutrient	mcg	Phylloquinone vitamin K1	189.19 (114.41)	167.9 (118.40, 232.00)
Nutrient	gm	Vegetable protein	22.16 (7.87)	21.2 (16.60, 26.60)
Nutrient	mg	Dietary zinc	12.34 (4.35)	11.8 (9.30, 14.80)
Nutrient	mg	Total zinc	16.58 (13.64)	12.9 (9.90, 17.40)
Food	s/d	Apple juice or cider	0.07 (0.18)	0 (0.00, 0.07)
Food	s/d	Apple and pear	0.36 (0.39)	0.21 (0.07, 0.50)
Food	s/d	Avocado	0.02 (0.06)	0 (0.00, 0.00)
Food	s/d	Bacon	0.09 (0.15)	0.07 (0.00, 0.14)
Food	s/d	banana	0.26 (0.30)	0.14 (0.07, 0.43)
Food	s/d	Beans or lentils	0.07 (0.10)	0.07 (0.00, 0.07)
Food	s/d	Beer	0.08 (0.35)	0 (0.00, 0.00)
Food	s/d	Blueberries	0.05 (0.11)	0 (0.00, 0.07)
Food	s/d	Beef or lamb as a main dish	0.26 (0.23)	0.14 (0.14, 0.43)
Food	s/d	Beef/pork/lamb as a sandwich	0.16 (0.16)	0.14 (0.07, 0.14)
Food	s/d	Brown rice	0.04 (0.09)	0 (0.00, 0.07)
Food	s/d	Broccoli	0.21 (0.20)	0.14 (0.07, 0.43)
Food	s/d	Brussels sprouts	0.03 (0.07)	0 (0.00, 0.07)
Food	s/d	Butter	0.34 (0.71)	0 (0.00, 0.43)
Food	s/d	Cabbage	0.07 (0.11)	0.07 (0.00, 0.07)
Food	s/d	Cake	0.08 (0.12)	0.07 (0.00, 0.14)
Food	s/d	Cantaloupe	0.12 (0.17)	0.07 (0.00, 0.14)
Food	s/d	Cauliflower	0.11 (0.14)	0.07 (0.07, 0.14)
Food	s/d	Cooked carrot	0.11 (0.15)	0.07 (0.07, 0.14)
Food	s/d	Candy bar	0.05 (0.13)	0 (0.00, 0.07)
Food	s/d	Candy without chocolate	0.08 (0.25)	0 (0.00, 0.07)
Food	s/d	Celery	0.21 (0.32)	0.07 (0.07, 0.43)
Food	s/d	Cold breakfast cereal	0.32 (0.38)	0.14 (0.07, 0.43)
Food	s/d	Chocolate bars	0.10 (0.23)	0.07 (0.00, 0.07)
Food	s/d	Chowder/cream soup	0.05 (0.08)	0 (0.00, 0.07)
Food	s/d	Chicken or turkey, with skin	0.11 (0.16)	0.07 (0.00, 0.14)
Food	s/d	Chicken/turkey, without skin	0.25 (0.18)	0.22 (0.12, 0.34)
Food	s/d	Other cooked cereal	0.03 (0.11)	0 (0.00, 0.00)
Food	s/d	Coffee	1.28 (1.24)	1 (0.07, 2.16)
Food	s/d	Non-dairy coffee whitener	0.27 (0.87)	0 (0.00, 0.00)
Food	s/d	Cookies	0.41 (0.70)	0.14 (0.07, 0.43)
Food	s/d	Corn	0.12 (0.13)	0.07 (0.07, 0.14)
Food	s/d	Cottage ricotta cheese	0.15 (0.24)	0.07 (0.00, 0.14)
Food	s/d	Crackers	0.31 (0.60)	0.14 (0.07, 0.43)
Food	s/d	Cream	0.26 (0.69)	0.07 (0.00, 0.14)
Food	s/d	Cream cheese	0.06 (0.12)	0 (0.00, 0.07)
Food	s/d	Cooked spinach	0.06 (0.10)	0.07 (0.00, 0.07)
Food	s/d	Canned tuna	0.15 (0.17)	0.14 (0.07, 0.14)
Food	s/d	Decaffeinated coffee	0.42 (0.79)	0 (0.00, 0.50)
Food	s/d	Wholegrain bread	0.65 (0.80)	0.43 (0.07, 0.79)
Food	s/d	Dark m,eat fish	0.04 (0.08)	0 (0.00, 0.07)
Food	s/d	Doughnuts	0.06 (0.11)	0.07 (0.00, 0.07)
Food	s/d	Eggplnt, zucchini, summer squash	0.09 (0.13)	0.07 (0.00, 0.14)

Supplementary Table 2. cont.

Type	Unit	Exposure	Mean (SD)	Median (25th and 75th percentiles)
Food	s/d	Egg	0.29 (0.28)	0.14 (0.07, 0.43)
Food	s/d	English muffins/bagels/rolls	0.18 (0.25)	0.07 (0.07, 0.14)
Food	s/d	French fried potatoes	0.06 (0.08)	0.07 (0.00, 0.07)
Food	s/d	Grapefruit and juice	0.23 (0.39)	0.07 (0.00, 0.28)
Food	s/d	Green/red peppers	0.18 (0.26)	0.07 (0.00, 0.14)
Food	s/d	Plain water	2.68 (1.85)	2.5 (1.00, 4.50)
Food	s/d	Hamburger	0.15 (0.14)	0.14 (0.07, 0.14)
Food	s/d	Hotdog	0.07 (0.10)	0.07 (0.00, 0.07)
Food	s/d	Ice cream	0.19 (0.28)	0.07 (0.07, 0.14)
Food	s/d	Iceberg or head lettuce	0.55 (0.46)	0.43 (0.14, 0.79)
Food	s/d	Jams, jellies	0.21 (0.33)	0.07 (0.00, 0.14)
Food	s/d	Kale	0.01 (0.06)	0 (0.00, 0.00)
Food	s/d	Ketchup or red chili sauce	0.02 (0.09)	0 (0.00, 0.00)
Food	s/d	Liquor	0.19 (0.52)	0 (0.00, 0.07)
Food	s/d	Beef liver	0.02 (0.02)	0.02 (0.00, 0.02)
Food	s/d	Chicken liver	0.01 (0.02)	0 (0.00, 0.02)
Food	s/d	Low caff beverages	0.51 (0.85)	0.14 (0.00, 0.79)
Food	s/d	1% -2% milk	0.39 (0.69)	0.07 (0.00, 0.43)
Food	s/d	Margarine	0.88 (0.99)	0.43 (0.07, 1.00)
Food	s/d	Mayo	0.28 (0.32)	0.14 (0.07, 0.43)
Food	s/d	Mixed vegetables	0.08 (0.13)	0.07 (0.00, 0.14)
Food	s/d	Muffins or biscuits	0.09 (0.16)	0.07 (0.00, 0.07)
Food	s/d	Cooked oatmeal/oatbran	0.06 (0.15)	0 (0.00, 0.07)
Food	s/d	Other fish	0.11 (0.13)	0.07 (0.07, 0.14)
Food	s/d	Onions as a garnish	0.31 (0.39)	0.14 (0.07, 0.43)
Food	s/d	Cooked onions	0.12 (0.24)	0.07 (0.00, 0.14)
Food	s/d	Other nuts	0.06 (0.15)	0 (0.00, 0.07)
Food	s/d	Olive oil	0.16 (0.33)	0 (0.00, 0.14)
Food	s/d	Oranges	0.20 (0.29)	0.07 (0.07, 0.14)
Food	s/d	Orange juice	0.46 (0.55)	0.43 (0.07, 1.00)
Food	s/d	Other cheese	0.34 (0.30)	0.29 (0.14, 0.43)
Food	s/d	Other fruit juices	0.14 (0.32)	0 (0.00, 0.14)
Food	s/d	Pasta	0.16 (0.14)	0.14 (0.07, 0.14)
Food	s/d	Peanut butter	0.20 (0.34)	0.07 (0.00, 0.14)
Food	s/d	Pancakes or waffles	0.04 (0.07)	0 (0.00, 0.07)
Food	s/d	Potato chips or corn chips	0.12 (0.21)	0.07 (0.00, 0.14)
Food	s/d	Popcorn	0.12 (0.26)	0.07 (0.00, 0.14)
Food	s/d	Peach/apricot	0.18 (0.26)	0.14 (0.07, 0.21)
Food	s/d	Peas, or lima beans	0.13 (0.14)	0.07 (0.07, 0.14)
Food	s/d	Pie	0.06 (0.08)	0.07 (0.00, 0.07)
Food	s/d	Pizza	0.07 (0.08)	0.07 (0.00, 0.07)
Food	s/d	Pork as a main dish	0.08 (0.08)	0.07 (0.00, 0.14)
Food	s/d	Peanuts	0.09 (0.22)	0.07 (0.00, 0.07)
Food	s/d	Potatoes, baked/boiled/mashed	0.33 (0.28)	0.43 (0.14, 0.43)
Food	s/d	All processed meats	0.13 (0.21)	0.07 (0.00, 0.14)
Food	s/d	Prune	0.04 (0.20)	0 (0.00, 0.00)
Food	s/d	Punch	0.09 (0.28)	0 (0.00, 0.07)
Food	s/d	Raisins or grapes	0.11 (0.23)	0.07 (0.00, 0.07)
Food	s/d	Raw carrots	0.22 (0.34)	0.14 (0.07, 0.43)
Food	s/d	Romaine or leaf lettuce	0.17 (0.32)	0.07 (0.00, 0.14)
Food	s/d	Raw spinach	0.05 (0.10)	0 (0.00, 0.07)
Food	s/d	Red wine	0.06 (0.23)	0 (0.00, 0.07)
Food	s/d	Salad/oil and vinegar dressing	0.27 (0.35)	0.14 (0.07, 0.43)
Food	s/d	Salsa	0.06 (0.13)	0 (0.00, 0.07)
Food	s/d	String beans	0.19 (0.18)	0.14 (0.07, 0.14)
Food	s/d	Sherbet or frozen yogurt	0.05 (0.15)	0 (0.00, 0.07)
Food	s/d	Shrimp, lobster, scallops	0.04 (0.06)	0 (0.00, 0.07)
Food	s/d	Skim milk	0.74 (0.90)	0.43 (0.00, 1.00)
Food	s/d	Strawberries	0.11 (0.18)	0.07 (0.07, 0.14)
Food	s/d	Beverages with sugar	0.23 (0.52)	0.07 (0.00, 0.14)

Supplementary Table 2. cont.

Type	Unit	Exposure	Mean (SD)	Median (25th and 75th percentiles)
Food	s/d	Sweet rolls	0.08 (0.13)	0.07 (0.00, 0.07)
Food	s/d	Tea	0.58 (1.03)	0.07 (0.00, 0.79)
Food	s/d	Tofu or soybeans	0.01 (0.07)	0 (0.00, 0.00)
Food	s/d	Tomato juice or V-8	0.06 (0.17)	0 (0.00, 0.07)
Food	s/d	Tomatoes	0.40 (0.37)	0.43 (0.14, 0.43)
Food	s/d	Tomato sauce	0.15 (0.15)	0.14 (0.07, 0.14)
Food	s/d	Whole grains	0.11 (0.36)	0 (0.00, 0.07)
Food	s/d	White bread	0.56 (0.81)	0.14 (0.07, 0.79)
Food	s/d	Whole milk	0.15 (0.43)	0 (0.00, 0.07)
Food	s/d	White rice	0.11 (0.15)	0.07 (0.07, 0.14)
Food	s/d	White wine	0.18 (0.44)	0 (0.00, 0.14)
Food	s/d	Yams or sweet potatoes	0.04 (0.06)	0 (0.00, 0.07)
Food	s/d	Yogurt	0.12 (0.24)	0 (0.00, 0.14)
Food	s/d	Yellow squash	0.06 (0.10)	0.07 (0.00, 0.07)

Supplementary Table 3: **EWAS Results.** All the exposures analyzed in our study, statistically significant or non-significant, are listed here. P-values are associated with two-sided Wald tests.

Type	Exposure	Effect size	SE(effect size)	Hazard ratio	95% CI	P-value of PH	VIF	P-value	FDR
Nutrient	Alcohol	-0.13	0.02	0.88	(0.84, 0.91)	0.19	1.12	4.98E-11	0.000
Nutrient	Added bran from wheat, rice, oat, corn	-0.13	0.02	0.87	(0.84, 0.91)	0.1	1.22	5.56E-10	0.000
Nutrient	Trans 16:1	0.17	0.03	1.19	(1.12, 1.25)	0.41	2.15	9.59E-10	0.000
Nutrient	Discretionary liquid fat	-0.15	0.02	0.86	(0.82, 0.91)	0.84	1.48	1.33E-09	0.000
Nutrient	Animal MUFA	0.16	0.03	1.17	(1.11, 1.24)	0.78	2.09	1.42E-08	0.001
Nutrient	Discretionary solid fat	0.17	0.03	1.18	(1.11, 1.25)	0.71	2.51	3.82E-08	0.001
Food	White wine	-0.12	0.02	0.89	(0.85, 0.92)	0.66	1.07	4.14E-08	0.001
Nutrient	Palmitoleic acid	0.16	0.03	1.17	(1.11, 1.24)	0.64	2.34	4.42E-08	0.001
Nutrient	Animal fat	0.16	0.03	1.17	(1.10, 1.24)	0.9	2.26	6.73E-08	0.001
Food	Salad/oil and vinegar dressing	-0.1	0.02	0.9	(0.87, 0.94)	0.24	1.09	3.41E-07	0.001
Food	Yogurt	-0.11	0.02	0.9	(0.86, 0.94)	0.09	1.07	1.37E-06	0.003
Nutrient	Phytate	-0.13	0.03	0.88	(0.84, 0.93)	0.02	1.96	4.01E-06	0.005
Nutrient	Stearic acid	0.15	0.03	1.16	(1.09, 1.23)	0.82	2.76	4.25E-06	0.005
Nutrient	Carbohydrate from milled wholegrain	-0.1	0.02	0.91	(0.87, 0.95)	0.29	1.29	5.03E-06	0.005
Nutrient	Sodium	0.13	0.03	1.14	(1.08, 1.21)	0.13	2.28	5.11E-06	0.005
Food	Raw carrots	-0.09	0.02	0.91	(0.87, 0.95)	0.06	1.1	6.33E-06	0.005
Nutrient	Total saturated fat	0.15	0.03	1.16	(1.08, 1.24)	0.78	3.14	1.20E-05	0.007
Nutrient	Hydroxyproline	0.11	0.03	1.12	(1.06, 1.17)	0.25	1.64	1.26E-05	0.007
Nutrient	Isorhamnetin	-0.09	0.02	0.91	(0.87, 0.95)	0.32	1.27	1.59E-05	0.007
Food	Liquor	-0.08	0.02	0.92	(0.89, 0.96)	0.12	1.06	2.06E-05	0.009
Nutrient	Carbohydrate from wholegrain	-0.09	0.02	0.91	(0.87, 0.95)	0.22	1.28	2.46E-05	0.010
Nutrient	Cereal fiber	-0.1	0.02	0.91	(0.87, 0.95)	0.2	1.49	4.04E-05	0.012
Food	Red wine	-0.09	0.02	0.91	(0.87, 0.95)	0.63	1.04	4.28E-05	0.012
Nutrient	Trans 18:2	0.11	0.03	1.12	(1.06, 1.18)	0.19	1.83	4.48E-05	0.012
Nutrient	Dietary tocopherols	-0.13	0.03	0.88	(0.83, 0.94)	0.75	2.78	5.71E-05	0.013
Nutrient	Palmitic acid	0.14	0.03	1.15	(1.07, 1.23)	0.93	3.37	7.01E-05	0.015
Nutrient	Dietary folate	-0.11	0.03	0.9	(0.85, 0.95)	0.49	1.91	8.33E-05	0.016
Food	Doughnuts	0.07	0.02	1.08	(1.04, 1.12)	0.1	1.1	9.84E-05	0.017
Nutrient	Beta tocotrienol	-0.09	0.02	0.92	(0.88, 0.96)	0.43	1.31	1.07E-04	0.018
Nutrient	Plant MUFA	-0.11	0.03	0.9	(0.85, 0.95)	0.8	2.05	1.22E-04	0.019
Food	Hotdog	0.07	0.02	1.07	(1.04, 1.11)	0.05	1.09	1.26E-04	0.019
Food	White bread	0.07	0.02	1.08	(1.04, 1.12)	0.08	1.12	1.61E-04	0.022
Nutrient	Natural germ	-0.08	0.02	0.92	(0.88, 0.96)	0.39	1.24	1.78E-04	0.022
Nutrient	Apigenin	-0.08	0.02	0.92	(0.89, 0.96)	0.81	1.15	1.79E-04	0.022
Nutrient	Beta tocopherol	-0.1	0.03	0.91	(0.86, 0.96)	0.55	1.87	2.73E-04	0.028
Nutrient	Natural bran	-0.08	0.02	0.92	(0.89, 0.96)	0.45	1.26	2.90E-04	0.028
Nutrient	Supplemental selenium	-0.08	0.02	0.92	(0.88, 0.96)	0.89	1.38	4.01E-04	0.034
Food	Apple juice or cider	0.07	0.02	1.07	(1.03, 1.11)	0.95	1.07	4.49E-04	0.036
Nutrient	Dietary manganese	-0.09	0.03	0.91	(0.86, 0.96)	0.08	2	4.82E-04	0.037
Food	Peanuts	-0.07	0.02	0.93	(0.89, 0.97)	0.09	1.08	4.92E-04	0.037
Nutrient	Alpha tocotrienol	-0.08	0.02	0.92	(0.88, 0.96)	0.43	1.51	5.37E-04	0.037
Nutrient	Myristic acid	0.09	0.03	1.1	(1.04, 1.15)	0.59	1.99	5.44E-04	0.037
Nutrient	Cholesterol	0.09	0.03	1.1	(1.04, 1.16)	0.43	2	6.09E-04	0.039
Nutrient	Supplemental or fortified folic acid	-0.08	0.02	0.92	(0.88, 0.97)	0.53	1.55	6.63E-04	0.040
Food	All processed meats	0.07	0.02	1.07	(1.03, 1.11)	0.23	1.14	6.73E-04	0.040
Nutrient	Trans 18:1	0.09	0.03	1.09	(1.04, 1.15)	0.62	1.93	6.94E-04	0.040
Nutrient	Total manganese	-0.08	0.02	0.92	(0.88, 0.97)	0.99	1.59	8.72E-04	0.046
Food	Hamburger	0.07	0.02	1.07	(1.03, 1.11)	0.09	1.18	9.53E-04	0.047
Food	Beverages with sugar	0.07	0.02	1.07	(1.03, 1.11)	0.86	1.13	9.70E-04	0.047
Nutrient	Synthetic vitamin B6	-0.07	0.02	0.94	(0.90, 0.97)	0.66	1.07	1.02E-03	0.047
Food	Cold breakfast cereal	-0.07	0.02	0.94	(0.90, 0.97)	0.13	1.1	1.02E-03	0.047
Food	Raisins or grapes	-0.07	0.02	0.93	(0.90, 0.97)	0.91	1.11	1.09E-03	0.048
Nutrient	Heme iron	0.08	0.02	1.08	(1.03, 1.14)	0.51	1.55	1.17E-03	0.050
Nutrient	Food supplemented alpha tocopherol	-0.06	0.02	0.94	(0.90, 0.98)	0.43	1.09	1.54E-03	0.058
Food	Cookies	-0.07	0.02	0.94	(0.90, 0.98)	0.93	1.21	1.55E-03	0.058
Nutrient	Total trans fat	0.08	0.03	1.09	(1.03, 1.15)	0.54	1.99	2.05E-03	0.069
Food	Beef or lamb as a main dish	0.06	0.02	1.07	(1.02, 1.11)	0.49	1.21	2.10E-03	0.069
Nutrient	Total magnesium	-0.09	0.03	0.91	(0.86, 0.97)	1	2.43	3.24E-03	0.090
Food	Dark m,eat fish	-0.06	0.02	0.94	(0.90, 0.98)	0.14	1.05	3.32E-03	0.090
Nutrient	Dietary choline from phosphatidylcholine	0.08	0.03	1.09	(1.03, 1.15)	0.74	2.23	3.40E-03	0.090

Supplementary Table 3. cont.

Type	Exposure	Effect size	SE(effect size)	Hazard ratio	95% CI	P-value of PH	VIF	P-value	FDR
Food	Broccoli	-0.06	0.02	0.94	(0.91, 0.98)	0.08	1.07	3.69E-03	0.093
Nutrient	Vegetable protein	-0.1	0.03	0.91	(0.85, 0.97)	0.07	3.14	3.72E-03	0.093
Nutrient	Total flavones	-0.06	0.02	0.94	(0.90, 0.98)	0.63	1.23	3.81E-03	0.093
Nutrient	Choline from sphingomyelin	0.08	0.03	1.08	(1.02, 1.14)	0.5	1.92	4.14E-03	0.095
Food	Romaine or leaf lettuce	-0.06	0.02	0.94	(0.91, 0.98)	0.28	1.08	4.18E-03	0.095
Nutrient	Glucoraphanin	-0.06	0.02	0.95	(0.91, 0.98)	0.07	1.07	4.27E-03	0.095
Nutrient	Total vitamin E	-0.07	0.02	0.93	(0.89, 0.98)	0.54	1.68	4.36E-03	0.095
Food	Other nuts	-0.06	0.02	0.94	(0.90, 0.98)	0.31	1.1	4.48E-03	0.095
Food	Olive oil	-0.06	0.02	0.94	(0.91, 0.98)	0.57	1.05	4.55E-03	0.095
Nutrient	Eicosenoic acid	-0.06	0.02	0.94	(0.90, 0.98)	0.4	1.35	4.56E-03	0.095
Nutrient	Dietary magnesium	-0.09	0.03	0.91	(0.86, 0.97)	0.58	2.81	4.75E-03	0.096
Nutrient	Fiber from legumes	-0.06	0.02	0.94	(0.90, 0.98)	0.15	1.26	4.98E-03	0.098
Nutrient	Vegetable fat	-0.08	0.03	0.92	(0.87, 0.98)	0.54	2.34	5.18E-03	0.099
Nutrient	Total fiber	-0.08	0.03	0.93	(0.88, 0.98)	0.03	2.07	5.55E-03	0.103
Food	Pie	0.05	0.02	1.06	(1.01, 1.10)	0.39	1.17	6.94E-03	0.119
Food	Plain water	0.05	0.02	1.05	(1.01, 1.10)	0.75	1.06	7.93E-03	0.129
Nutrient	Neoglucobrassicin	-0.05	0.02	0.95	(0.91, 0.99)	0.06	1.07	8.27E-03	0.131
Nutrient	Total isoflavones	-0.06	0.02	0.95	(0.91, 0.99)	0.58	1.14	8.57E-03	0.133
Food	String beans	0.05	0.02	1.05	(1.01, 1.09)	0.11	1.08	8.83E-03	0.134
Nutrient	Dietary alpha tocopherol	-0.08	0.03	0.93	(0.87, 0.98)	0.7	2.39	9.83E-03	0.143
Food	Whole grains	-0.05	0.02	0.95	(0.91, 0.99)	0.27	1.08	1.03E-02	0.146
Nutrient	Supplemental iodine	-0.06	0.02	0.95	(0.90, 0.99)	0.42	1.39	1.16E-02	0.157
Nutrient	Quercetin	-0.05	0.02	0.95	(0.91, 0.99)	0.68	1.27	1.22E-02	0.161
Nutrient	Hesperetin	-0.05	0.02	0.95	(0.92, 0.99)	0.62	1.13	1.31E-02	0.167
Nutrient	Glucobarbarin	-0.05	0.02	0.95	(0.91, 0.99)	0.11	1.06	1.43E-02	0.177
Food	Wholegrain bread	-0.05	0.02	0.95	(0.92, 0.99)	0.73	1.11	1.70E-02	0.198
Nutrient	Glucobrassicanapin	-0.05	0.02	0.95	(0.92, 0.99)	0.03	1.07	1.72E-02	0.198
Nutrient	Total calcium	-0.05	0.02	0.95	(0.91, 0.99)	0.14	1.39	1.77E-02	0.200
Nutrient	Carbohydrate from intact wholegrain	-0.05	0.02	0.95	(0.91, 0.99)	0.2	1.13	1.84E-02	0.204
Nutrient	Total beta carotene	-0.05	0.02	0.95	(0.91, 0.99)	0.01	1.29	1.89E-02	0.205
Food	Ketchup or red chili sauce	-0.05	0.02	0.95	(0.91, 0.99)	0.7	1.11	1.92E-02	0.205
Nutrient	Proanthocyanidin, 7-10mers	-0.05	0.02	0.95	(0.91, 0.99)	0.97	1.28	2.00E-02	0.207
Nutrient	Total furocoumarins	-0.05	0.02	0.96	(0.92, 0.99)	0.25	1.06	2.03E-02	0.207
Food	Cream cheese	0.04	0.02	1.04	(1.01, 1.08)	0.99	1.05	2.04E-02	0.207
Nutrient	Dietary long chain fatty acid	-0.05	0.02	0.95	(0.92, 0.99)	0.31	1.11	2.12E-02	0.210
Nutrient	Total flavanones	-0.05	0.02	0.95	(0.92, 0.99)	0.97	1.14	2.15E-02	0.210
Nutrient	Total dietary omega 3 (no alpha 18:3)	-0.05	0.02	0.95	(0.92, 0.99)	0.27	1.11	2.16E-02	0.210
Nutrient	Crude fiber	-0.06	0.03	0.94	(0.89, 0.99)	0.05	1.9	2.23E-02	0.211
Nutrient	Dairy fat	0.05	0.02	1.06	(1.01, 1.11)	0.55	1.62	2.24E-02	0.211
Nutrient	Total fat	0.09	0.04	1.09	(1.01, 1.18)	0.95	4.21	2.26E-02	0.211
Nutrient	Dihydrovitamin K	0.06	0.02	1.06	(1.01, 1.11)	0.77	1.65	2.32E-02	0.211
Nutrient	6'7'-Dihydroxybergamottin	-0.04	0.02	0.96	(0.92, 0.99)	0.24	1.06	2.33E-02	0.211
Nutrient	Total vitamin A	-0.05	0.02	0.95	(0.91, 0.99)	0.08	1.36	2.37E-02	0.212
Nutrient	Maltose	0.06	0.03	1.06	(1.01, 1.11)	0.89	1.82	2.45E-02	0.212
Food	Beef/pork/lamb as a sandwich	0.05	0.02	1.05	(1.01, 1.09)	0.82	1.22	2.46E-02	0.212
Food	Cooked oatmeal/oatbran	-0.05	0.02	0.96	(0.92, 0.99)	0.65	1.1	2.47E-02	0.212
Nutrient	Total flavonols	-0.05	0.02	0.95	(0.91, 0.99)	0.68	1.22	2.51E-02	0.213
Nutrient	Genistein	-0.05	0.02	0.95	(0.91, 0.99)	0.4	1.1	2.59E-02	0.216
Food	Avocado	-0.05	0.02	0.95	(0.92, 0.99)	0.64	1.03	2.74E-02	0.223
Food	Chicken or turkey, with skin	0.04	0.02	1.05	(1.00, 1.09)	0.34	1.08	2.82E-02	0.225
Nutrient	Proanthocyanidin, 4-6mers	-0.05	0.02	0.95	(0.91, 0.99)	0.84	1.34	2.84E-02	0.225
Nutrient	Oxalate	-0.05	0.02	0.95	(0.91, 1.00)	0.22	1.39	3.07E-02	0.236
Food	Muffins or biscuits	0.04	0.02	1.04	(1.00, 1.08)	0.69	1.09	3.16E-02	0.239
Nutrient	Bergamottin	-0.04	0.02	0.96	(0.92, 1.00)	0.23	1.06	3.23E-02	0.241
Nutrient	Naringenin	-0.04	0.02	0.96	(0.92, 1.00)	0.5	1.12	3.32E-02	0.244
Nutrient	Proanthocyanidin, polymers	-0.05	0.02	0.96	(0.92, 1.00)	0.62	1.27	3.48E-02	0.249
Nutrient	Malvidin	-0.04	0.02	0.96	(0.92, 1.00)	0.18	1.12	3.48E-02	0.249
Nutrient	Total vitamin B12	-0.05	0.02	0.95	(0.91, 1.00)	0.04	1.37	3.62E-02	0.252
Nutrient	Capric acid	0.05	0.02	1.05	(1.00, 1.10)	0.27	1.64	3.63E-02	0.252
Nutrient	Dietary vitamin D	-0.05	0.02	0.95	(0.91, 1.00)	0.03	1.35	3.73E-02	0.254

Supplementary Table 3. cont.

Type	Exposure	Effect size	SE(effect size)	Hazard ratio	95% CI	P-value of PH	VIF	P-value	FDR
Nutrient	Dietary iron	-0.06	0.03	0.94	(0.89, 1.00)	0.08	2.14	3.75E-02	0.254
Food	Tofu or soybeans	-0.05	0.02	0.95	(0.91, 1.00)	0.09	1.04	4.11E-02	0.268
Nutrient	Gamma tocopherol	-0.06	0.03	0.94	(0.89, 1.00)	0.77	2.25	4.12E-02	0.268
Nutrient	Glycitein	-0.05	0.02	0.96	(0.91, 1.00)	0.08	1.05	4.19E-02	0.269
Food	Prune	-0.04	0.02	0.96	(0.93, 1.00)	0.41	1.05	4.21E-02	0.269
Nutrient	Animal protein	0.06	0.03	1.06	(1.00, 1.12)	0.77	2.15	4.40E-02	0.275
Food	Punch	0.04	0.02	1.04	(1.00, 1.08)	0.47	1.08	4.48E-02	0.275
Nutrient	Glucocheirolin	0.04	0.02	1.04	(1.00, 1.08)	0.04	1.02	4.51E-02	0.275
Nutrient	Epiprogoitrin	-0.04	0.02	0.96	(0.92, 1.00)	0.21	1.05	4.59E-02	0.275
Food	Pork as a main dish	0.04	0.02	1.04	(1.00, 1.08)	0.02	1.1	4.60E-02	0.275
Nutrient	Isoflavones (no biochanin A or formononetin)	-0.04	0.02	0.96	(0.92, 1.00)	0.54	1.11	4.65E-02	0.275
Nutrient	Luteolin	-0.04	0.02	0.96	(0.92, 1.00)	0.83	1.2	4.85E-02	0.277
Food	Brussels sprouts	0.04	0.02	1.04	(1.00, 1.07)	0.04	1.02	4.86E-02	0.277
Nutrient	Dietary thiamine	-0.07	0.03	0.93	(0.87, 1.00)	0.25	3.17	4.87E-02	0.277
Nutrient	Caproic acid	0.04	0.02	1.05	(1.00, 1.09)	0.33	1.45	4.89E-02	0.277
Food	Candy bar	0.04	0.02	1.04	(1.00, 1.08)	0.68	1.08	5.16E-02	0.285
Nutrient	Total free choline	-0.06	0.03	0.94	(0.89, 1.00)	0.89	2.25	5.20E-02	0.285
Food	Apple and pear	-0.04	0.02	0.96	(0.92, 1.00)	0.6	1.1	5.24E-02	0.285
Nutrient	Sinigrin	0.04	0.02	1.04	(1.00, 1.08)	0.02	1.07	5.33E-02	0.285
Food	Tomato juice or V-8	0.03	0.02	1.04	(1.00, 1.07)	0.43	1.04	5.34E-02	0.285
Nutrient	Total phospherous	-0.07	0.03	0.94	(0.87, 1.00)	0.67	3.35	5.39E-02	0.285
Food	Pasta	-0.04	0.02	0.96	(0.92, 1.00)	0.59	1.16	5.85E-02	0.299
Food	Canned tuna	-0.04	0.02	0.96	(0.92, 1.00)	0.1	1.07	5.91E-02	0.299
Nutrient	Daidzein	-0.04	0.02	0.96	(0.92, 1.00)	0.41	1.07	5.91E-02	0.299
Nutrient	Menaquinone vitamin K2 (4 isoprenoid residues)	0.04	0.02	1.04	(1.00, 1.09)	0.1	1.45	6.02E-02	0.301
Food	Corn	0.04	0.02	1.04	(1.00, 1.08)	0.88	1.11	6.30E-02	0.309
Nutrient	Glucoalyssin	-0.04	0.02	0.96	(0.93, 1.00)	0.04	1.07	6.47E-02	0.313
Nutrient	Choline derivative betaine	-0.05	0.03	0.95	(0.91, 1.00)	0.08	1.76	6.61E-02	0.316
Nutrient	Total long chain fatty acid	-0.04	0.02	0.96	(0.93, 1.00)	0.74	1.11	6.66E-02	0.316
Nutrient	Linoleic acid	-0.06	0.03	0.95	(0.89, 1.00)	0.93	2.55	6.72E-02	0.316
Nutrient	Total polyunsaturated fat	-0.06	0.03	0.94	(0.89, 1.00)	0.92	2.72	6.89E-02	0.316
Nutrient	Protein	-0.05	0.03	0.95	(0.91, 1.00)	0.01	1.99	6.90E-02	0.316
Nutrient	Omega 3 (20:5+22:6, no alpha 18:3)	-0.04	0.02	0.96	(0.93, 1.00)	0.7	1.11	7.01E-02	0.316
Nutrient	Dietary choline	0.06	0.03	1.06	(0.99, 1.14)	0.65	3.2	7.02E-02	0.316
Nutrient	Total vitamin C	-0.04	0.02	0.96	(0.92, 1.00)	0.83	1.29	7.05E-02	0.320
Nutrient	Gluconapin	0.03	0.02	1.03	(1.00, 1.07)	0.02	1.04	7.21E-02	0.325
Nutrient	Proanthocyanidin, dimers	-0.04	0.02	0.96	(0.92, 1.00)	0.89	1.24	7.43E-02	0.325
Food	Coffee	-0.03	0.02	0.97	(0.93, 1.00)	0.56	1.04	7.53E-02	0.325
Nutrient	Dietary betaine	-0.05	0.03	0.96	(0.91, 1.00)	0.08	1.77	7.61E-02	0.325
Nutrient	Total iron	-0.04	0.02	0.96	(0.91, 1.00)	0.6	1.44	7.64E-02	0.330
Nutrient	Added germ from wheat	-0.04	0.02	0.96	(0.93, 1.00)	0.9	1.06	7.86E-02	0.336
Nutrient	Total pyridoxine	-0.04	0.02	0.96	(0.92, 1.00)	0.22	1.37	8.11E-02	0.339
Food	Low caff beverages	-0.04	0.02	0.96	(0.93, 1.00)	0	1.1	8.29E-02	0.340
Nutrient	Choline from phosphatidylcholine	0.05	0.03	1.05	(0.99, 1.10)	0.95	1.96	8.39E-02	0.342
Nutrient	Total zinc	-0.04	0.02	0.96	(0.92, 1.01)	0.37	1.5	8.50E-02	0.345
Nutrient	Dietary niacin	-0.05	0.03	0.95	(0.89, 1.01)	0.34	2.72	8.68E-02	0.357
Nutrient	Nitrate	-0.04	0.02	0.96	(0.93, 1.01)	0.03	1.22	9.20E-02	0.357
Nutrient	Acrylamide	-0.04	0.02	0.96	(0.92, 1.01)	0.76	1.41	9.21E-02	0.364
Nutrient	Dietary riboflavin	-0.05	0.03	0.95	(0.90, 1.01)	0.21	2.41	9.57E-02	0.364
Nutrient	Dietary phosphorous	-0.06	0.03	0.94	(0.88, 1.01)	0.67	3.39	9.63E-02	0.370
Nutrient	Bergaptol	-0.03	0.02	0.97	(0.93, 1.01)	0.22	1.05	9.93E-02	0.372
Nutrient	Butyric acid	0.04	0.02	1.04	(0.99, 1.09)	0.49	1.53	1.01E-01	0.372
Nutrient	Dietary pyridoxine	-0.05	0.03	0.95	(0.90, 1.01)	0.49	2.32	1.02E-01	0.372
Nutrient	Glycine	0.05	0.03	1.06	(0.99, 1.13)	0.46	2.93	1.02E-01	0.379
Nutrient	Dietary vitamin A	-0.04	0.02	0.96	(0.92, 1.01)	0.06	1.36	1.06E-01	0.381
Food	Orange juice	-0.03	0.02	0.97	(0.93, 1.01)	0.71	1.08	1.07E-01	0.381
Nutrient	Added sugar	0.04	0.03	1.04	(0.99, 1.09)	1	1.74	1.08E-01	0.381
Food	Peas, or lima beans	0.03	0.02	1.03	(0.99, 1.07)	0.48	1.11	1.09E-01	0.398
Food	Bacon	0.03	0.02	1.03	(0.99, 1.07)	0.21	1.09	1.16E-01	0.398
Nutrient	Proanthocyanidin, trimers	-0.04	0.02	0.96	(0.92, 1.01)	0.72	1.44	1.17E-01	0.400

Supplementary Table 3. cont.

Type	Exposure	Effect size	SE(effect size)	Hazard ratio	95% CI	P-value of PH	VIF	P-value	FDR
Nutrient	DHA fatty acid	-0.03	0.02	0.97	(0.93, 1.01)	0.47	1.11	1.19E-01	0.400
Food	Jams, jellies	-0.03	0.02	0.97	(0.93, 1.01)	0.89	1.15	1.20E-01	0.400
Food	Mayo	-0.03	0.02	0.97	(0.93, 1.01)	0.7	1.15	1.21E-01	0.400
Nutrient	Total vitamin D	-0.04	0.02	0.96	(0.92, 1.01)	0.12	1.49	1.21E-01	0.403
Nutrient	EPA fatty acid	-0.03	0.02	0.97	(0.93, 1.01)	0.86	1.08	1.23E-01	0.405
Nutrient	Alpha carotene	-0.03	0.02	0.97	(0.93, 1.01)	0.08	1.2	1.26E-01	0.405
Nutrient	Histidine	0.05	0.03	1.05	(0.99, 1.13)	0.55	3.28	1.26E-01	0.405
Food	Other cheese	-0.03	0.02	0.97	(0.93, 1.01)	0.69	1.13	1.27E-01	0.409
Nutrient	Arachadonic acid	0.04	0.02	1.04	(0.99, 1.09)	0.44	1.51	1.30E-01	0.409
Food	Tomatoes	-0.03	0.02	0.97	(0.93, 1.01)	0.12	1.08	1.30E-01	0.411
Nutrient	Sulfur from methionine	0.05	0.03	1.05	(0.99, 1.11)	0.84	2.66	1.32E-01	0.411
Food	Sherbet or frozen yogurt	-0.03	0.02	0.97	(0.93, 1.01)	0.01	1.08	1.33E-01	0.411
Nutrient	Threonine	0.05	0.03	1.05	(0.98, 1.12)	0.72	3.18	1.34E-01	0.414
Food	Skim milk	-0.03	0.02	0.97	(0.93, 1.01)	0.59	1.07	1.36E-01	0.414
Food	Candy without chocolate	-0.03	0.02	0.97	(0.94, 1.01)	0.64	1.06	1.37E-01	0.434
Nutrient	Fruit carbohydrate minus juice sugar	-0.03	0.02	0.97	(0.93, 1.01)	0.92	1.34	1.47E-01	0.434
Food	Beef liver	0.03	0.02	1.03	(0.99, 1.07)	0.51	1.02	1.48E-01	0.436
Nutrient	Total folate	-0.03	0.02	0.97	(0.92, 1.01)	0.1	1.48	1.51E-01	0.436
Nutrient	Total copper	-0.04	0.03	0.96	(0.92, 1.01)	0.85	1.71	1.52E-01	0.436
Food	Raw spinach	-0.03	0.02	0.97	(0.93, 1.01)	0.07	1.06	1.52E-01	0.436
Nutrient	Dietary beta carotene	-0.03	0.02	0.97	(0.93, 1.01)	0.02	1.29	1.53E-01	0.436
Nutrient	Caprylic acid	0.03	0.02	1.03	(0.99, 1.08)	0.1	1.44	1.54E-01	0.436
Nutrient	Alanine	0.05	0.03	1.05	(0.98, 1.12)	0.62	2.92	1.55E-01	0.436
Nutrient	Proanthocyanidin, monomers	-0.03	0.02	0.97	(0.93, 1.01)	0.89	1.12	1.56E-01	0.445
Food	Whole milk	0.03	0.02	1.03	(0.99, 1.06)	0.82	1.04	1.62E-01	0.447
Nutrient	Dietary potassium	-0.05	0.03	0.96	(0.90, 1.02)	0.91	2.96	1.64E-01	0.449
Food	French fried potatoes	0.03	0.02	1.03	(0.99, 1.07)	0.96	1.15	1.66E-01	0.449
Food	Celery	-0.03	0.02	0.97	(0.94, 1.01)	0.33	1.08	1.69E-01	0.449
Nutrient	Lysine	0.04	0.03	1.04	(0.98, 1.11)	0.88	2.72	1.69E-01	0.449
Nutrient	Dietary free choline	-0.04	0.03	0.96	(0.90, 1.02)	0.81	2.63	1.71E-01	0.449
Food	Green/red peppers	-0.03	0.02	0.97	(0.94, 1.01)	0.98	1.05	1.71E-01	0.449
Nutrient	Lactose	-0.03	0.02	0.97	(0.93, 1.01)	0.11	1.23	1.72E-01	0.449
Nutrient	Total anthocyanidins	-0.03	0.02	0.97	(0.93, 1.01)	0.51	1.17	1.72E-01	0.451
Nutrient	Fruit fiber	-0.03	0.02	0.97	(0.93, 1.01)	0.54	1.28	1.76E-01	0.451
Nutrient	Total MUFA	0.05	0.04	1.05	(0.98, 1.13)	1	3.49	1.76E-01	0.453
Food	Iceberg or head lettuce	-0.03	0.02	0.97	(0.94, 1.01)	0.33	1.06	1.78E-01	0.465
Nutrient	Pantothenic acid	-0.03	0.02	0.97	(0.93, 1.02)	0.26	1.51	1.86E-01	0.465
Food	Mixed vegetables	0.03	0.02	1.03	(0.99, 1.07)	0	1.08	1.87E-01	0.477
Nutrient	Methionine	0.04	0.03	1.04	(0.98, 1.11)	0.86	2.92	1.94E-01	0.477
Nutrient	Peonidin	-0.03	0.02	0.97	(0.94, 1.01)	0.26	1.11	1.95E-01	0.478
Nutrient	Beta cryptoxanthin	-0.03	0.02	0.97	(0.93, 1.01)	0.74	1.27	1.97E-01	0.478
Nutrient	Tryptophan	0.05	0.04	1.05	(0.98, 1.12)	0.63	3.56	1.98E-01	0.501
Nutrient	Glycosolated B6	-0.03	0.03	0.97	(0.92, 1.02)	0.06	1.73	2.13E-01	0.501
Nutrient	Dietary omega 6 (no gamma 18:3)	-0.04	0.03	0.96	(0.91, 1.02)	0.34	2.5	2.13E-01	0.506
Food	Other fruit juices	0.02	0.02	1.02	(0.99, 1.06)	0.74	1.08	2.18E-01	0.506
Nutrient	Dietary calcium	-0.03	0.03	0.97	(0.92, 1.02)	0.19	1.8	2.19E-01	0.527
Food	Peanut butter	-0.02	0.02	0.98	(0.94, 1.02)	0.12	1.09	2.32E-01	0.532
Nutrient	Myricetin	-0.02	0.02	0.98	(0.94, 1.02)	0.67	1.1	2.36E-01	0.539
Nutrient	Progoitrin	0.02	0.02	1.02	(0.98, 1.06)	0.02	1.05	2.42E-01	0.540
Food	Cabbage	0.02	0.02	1.02	(0.98, 1.06)	0.69	1.08	2.44E-01	0.545
Nutrient	Dairy calcium	-0.03	0.02	0.97	(0.93, 1.02)	0.22	1.37	2.50E-01	0.545
Nutrient	Formononetin	-0.02	0.02	0.98	(0.94, 1.02)	0.73	1.12	2.50E-01	0.546
Nutrient	Dietary sulfur	0.04	0.04	1.04	(0.97, 1.12)	0.98	3.88	2.53E-01	0.546
Nutrient	Thearubigins	-0.02	0.02	0.98	(0.94, 1.02)	0.88	1.03	2.53E-01	0.547
Food	Butter	0.02	0.02	1.02	(0.98, 1.06)	0.24	1.04	2.57E-01	0.547
Nutrient	Lauric acid	0.02	0.02	1.03	(0.98, 1.07)	0.03	1.39	2.57E-01	0.550
Food	Yellow squash	0.02	0.02	1.02	(0.98, 1.06)	0.03	1.04	2.60E-01	0.555
Food	White rice	-0.02	0.02	0.98	(0.94, 1.02)	0.06	1.13	2.65E-01	0.562
Nutrient	Isoleucine	0.04	0.03	1.04	(0.97, 1.11)	0.86	3.17	2.71E-01	0.562
Nutrient	Pelargonidin	-0.02	0.02	0.98	(0.94, 1.02)	0.66	1.09	2.72E-01	0.566

Supplementary Table 3. cont.

Type	Exposure	Effect size	SE(effect size)	Hazard ratio	95% CI	P-value of PH	VIF	P-value	FDR
Nutrient	Petunidin	-0.02	0.02	0.98	(0.94, 1.02)	0.48	1.1	2.76E-01	0.571
Food	Popcorn	-0.02	0.02	0.98	(0.94, 1.02)	0.25	1.07	2.82E-01	0.571
Nutrient	Total riboflavin	-0.02	0.02	0.98	(0.93, 1.02)	0.11	1.41	2.84E-01	0.571
Nutrient	Oleic acid	0.04	0.04	1.04	(0.97, 1.11)	0.96	3.37	2.84E-01	0.579
Nutrient	Thiamine	-0.02	0.02	0.98	(0.93, 1.02)	0.2	1.42	2.90E-01	0.580
Nutrient	Phylloquinone vitamin K1	-0.02	0.02	0.98	(0.94, 1.02)	0.03	1.25	2.93E-01	0.580
Nutrient	Lutein and zeaxanthin	-0.02	0.02	0.98	(0.94, 1.02)	0.05	1.23	2.95E-01	0.580
Nutrient	Dietary phylloquinone vitamin K1	-0.02	0.02	0.98	(0.94, 1.02)	0.02	1.25	2.96E-01	0.584
Nutrient	Total sugar	0.03	0.03	1.03	(0.97, 1.09)	0.6	2.5	3.01E-01	0.588
Nutrient	Leucine	0.04	0.03	1.04	(0.97, 1.11)	0.87	3.39	3.08E-01	0.588
Food	Sweet rolls	0.02	0.02	1.02	(0.98, 1.06)	0.69	1.13	3.10E-01	0.588
Nutrient	Total flavonoids	-0.02	0.02	0.98	(0.94, 1.02)	0.97	1.16	3.10E-01	0.588
Nutrient	Alpha linolenic	-0.03	0.03	0.97	(0.92, 1.03)	0.26	2.02	3.11E-01	0.588
Nutrient	Fructose	0.03	0.03	1.03	(0.98, 1.08)	0.4	1.74	3.11E-01	0.590
Nutrient	Dietary vitamin C	-0.02	0.02	0.98	(0.93, 1.02)	0.29	1.44	3.16E-01	0.590
Nutrient	Napoleiferin	-0.02	0.02	0.98	(0.94, 1.02)	0.02	1.07	3.17E-01	0.590
Nutrient	Glutamic acid	0.04	0.04	1.04	(0.96, 1.12)	0.96	4.23	3.18E-01	0.590
Food	Salsa	-0.02	0.02	0.98	(0.94, 1.02)	0.07	1.03	3.20E-01	0.590
Nutrient	Carbohydrate from refined grain	0.03	0.03	1.03	(0.97, 1.08)	0.81	1.9	3.21E-01	0.591
Nutrient	Dietary vitamin B12	0.02	0.02	1.02	(0.98, 1.07)	0.13	1.33	3.23E-01	0.595
Nutrient	Delphinidin	-0.02	0.02	0.98	(0.94, 1.02)	0.49	1.1	3.32E-01	0.595
Nutrient	Arginine	0.03	0.03	1.03	(0.97, 1.10)	0.35	3.12	3.33E-01	0.595
Nutrient	Kaempferol	-0.02	0.02	0.98	(0.94, 1.02)	0.28	1.08	3.33E-01	0.595
Nutrient	Lycopene	0.02	0.02	1.02	(0.98, 1.06)	0.44	1.28	3.34E-01	0.595
Food	Eggplnt, zucchini, summer squash	-0.02	0.02	0.98	(0.94, 1.02)	0.67	1.05	3.35E-01	0.595
Nutrient	Eriodictyol	-0.02	0.02	0.98	(0.95, 1.02)	0.69	1.09	3.37E-01	0.595
Nutrient	Total dietary omega 3	-0.03	0.03	0.97	(0.92, 1.03)	0.1	2.05	3.38E-01	0.596
Nutrient	Delta tocotrienol	-0.02	0.02	0.98	(0.94, 1.02)	0.45	1.07	3.43E-01	0.596
Nutrient	Choline from glycerophosphocholine	-0.02	0.02	0.98	(0.93, 1.02)	0.12	1.6	3.44E-01	0.596
Food	Chocolate bars	0.02	0.02	1.02	(0.98, 1.06)	0.83	1.09	3.45E-01	0.596
Nutrient	Total niacin	-0.02	0.02	0.98	(0.94, 1.02)	0.26	1.48	3.46E-01	0.596
Food	Oranges	-0.02	0.02	0.98	(0.94, 1.02)	0.73	1.07	3.47E-01	0.598
Food	Beer	-0.02	0.02	0.98	(0.95, 1.02)	0.18	1.03	3.50E-01	0.600
Nutrient	4-Hydroxyglucobrassicin	-0.02	0.02	0.98	(0.94, 1.02)	0.03	1.08	3.53E-01	0.610
Nutrient	Catechin	-0.02	0.02	0.98	(0.94, 1.02)	0.86	1.2	3.63E-01	0.615
Nutrient	Dairy protein	-0.02	0.02	0.98	(0.94, 1.02)	0.49	1.41	3.69E-01	0.623
Nutrient	Glucoerucin	-0.02	0.02	0.98	(0.95, 1.02)	0.03	1.05	3.77E-01	0.623
Nutrient	Starch	-0.03	0.03	0.97	(0.92, 1.03)	0.55	2.5	3.79E-01	0.623
Nutrient	Cysteine	0.03	0.04	1.03	(0.96, 1.11)	0.66	3.76	3.80E-01	0.625
Nutrient	Total retinol	-0.02	0.02	0.98	(0.94, 1.02)	0.81	1.32	3.84E-01	0.634
Nutrient	Total theaflavins and polymers proanthocyanidis	-0.02	0.02	0.98	(0.94, 1.02)	0.82	1.12	3.93E-01	0.636
Nutrient	Sucrose	0.02	0.03	1.02	(0.97, 1.08)	0.9	2.04	3.96E-01	0.641
Nutrient	Tyrosine	0.03	0.04	1.03	(0.96, 1.10)	0.94	3.43	4.02E-01	0.641
Nutrient	Flavonoids (no proanthocyanidins)	-0.02	0.02	0.98	(0.95, 1.02)	0.9	1.09	4.05E-01	0.641
Nutrient	Taurine	0.02	0.02	1.02	(0.97, 1.07)	0.98	1.42	4.06E-01	0.641
Food	Peach/apricot	0.02	0.02	1.02	(0.98, 1.06)	0.63	1.09	4.08E-01	0.667
Nutrient	Total choline (no betaine)	0.03	0.03	1.03	(0.96, 1.09)	0.53	2.79	4.31E-01	0.680
Nutrient	Total omega 3	-0.02	0.03	0.98	(0.93, 1.03)	0.18	1.96	4.45E-01	0.680
Nutrient	Epigallocatechin	-0.01	0.02	0.99	(0.95, 1.02)	0.95	1.04	4.46E-01	0.688
Nutrient	Natural sugar	-0.02	0.03	0.98	(0.93, 1.03)	0.58	1.74	4.55E-01	0.690
Food	1% -2% milk	-0.01	0.02	0.99	(0.95, 1.02)	0.51	1.03	4.58E-01	0.691
Nutrient	Dietary copper	-0.02	0.03	0.98	(0.92, 1.04)	0.32	2.79	4.62E-01	0.699
Nutrient	Valine	0.03	0.04	1.03	(0.96, 1.10)	0.99	3.43	4.70E-01	0.700
Food	English muffins/bagels/rolls	-0.01	0.02	0.99	(0.95, 1.03)	0.76	1.09	4.74E-01	0.712
Nutrient	Sugar from fruit juice	-0.01	0.02	0.99	(0.95, 1.03)	0.76	1.14	4.90E-01	0.712
Nutrient	Choline from phosphocholine	-0.02	0.03	0.98	(0.94, 1.03)	0.69	1.75	4.90E-01	0.712
Nutrient	Asparagine	0.02	0.03	1.02	(0.96, 1.10)	0.67	3.24	4.91E-01	0.715
Nutrient	Sum of sulfur from methionine and cysteine	0.02	0.03	1.02	(0.96, 1.09)	0.7	2.69	4.95E-01	0.721
Nutrient	Biochanin A	-0.01	0.02	0.99	(0.95, 1.03)	0.74	1.13	5.03E-01	0.721
Nutrient	Caffeine	0.01	0.02	1.01	(0.97, 1.06)	0.57	1.1	5.07E-01	0.721

Supplementary Table 3. cont.

Type	Exposure	Effect size	SE(effect size)	Hazard ratio	95% CI	P-value of PH	VIF	P-value	FDR
Nutrient	Fiber from cruciferous veg	-0.01	0.02	0.99	(0.95, 1.03)	0.14	1.1	5.08E-01	0.721
Food	Chowder/cream soup	0.01	0.02	1.01	(0.97, 1.05)	0.78	1.09	5.09E-01	0.721
Nutrient	Phenylalanine	0.02	0.04	1.02	(0.95, 1.10)	0.93	3.89	5.11E-01	0.723
Nutrient	Cystine	-0.02	0.04	0.98	(0.91, 1.05)	0.33	3.76	5.19E-01	0.723
Nutrient	Total flavan-3-ols	-0.01	0.02	0.99	(0.95, 1.03)	0.83	1.08	5.20E-01	0.723
Nutrient	Epicatechin 3-gallate	-0.01	0.02	0.99	(0.95, 1.03)	0.98	1.03	5.20E-01	0.733
Nutrient	Glucobrassicin	-0.01	0.02	0.99	(0.95, 1.03)	0.04	1.08	5.31E-01	0.735
Food	Tea	-0.01	0.02	0.99	(0.95, 1.03)	0.71	1.03	5.35E-01	0.735
Food	Yams or sweet potatoes	-0.01	0.02	0.99	(0.95, 1.03)	0.04	1.09	5.39E-01	0.735
Nutrient	Dairy vitamin D	-0.01	0.02	0.99	(0.95, 1.03)	0.06	1.19	5.40E-01	0.744
Food	Tomato sauce	0.01	0.02	1.01	(0.97, 1.06)	0.65	1.17	5.50E-01	0.750
Nutrient	Glucoerysolin	0.01	0.02	1.01	(0.97, 1.05)	0.84	1.07	5.58E-01	0.762
Nutrient	Aspartic acid	0.02	0.03	1.02	(0.95, 1.09)	0.79	3.33	5.72E-01	0.767
Nutrient	Gluten	-0.01	0.03	0.99	(0.94, 1.04)	0.95	1.86	5.81E-01	0.767
Food	Decaffeinated coffee	-0.01	0.02	0.99	(0.95, 1.03)	0.18	1	5.82E-01	0.770
Nutrient	Gluconasturtiin	-0.01	0.02	0.99	(0.95, 1.03)	0	1.06	5.89E-01	0.770
Nutrient	Total glucosinolates	-0.01	0.02	0.99	(0.95, 1.03)	0.03	1.09	5.90E-01	0.772
Nutrient	Bergapten	-0.01	0.02	0.99	(0.95, 1.03)	0.15	1.09	5.96E-01	0.772
Food	Blueberries	-0.01	0.02	0.99	(0.95, 1.03)	0.89	1.06	5.97E-01	0.772
Nutrient	Serine	0.02	0.04	1.02	(0.95, 1.10)	0.77	3.97	5.99E-01	0.772
Food	Onions as a garnish	-0.01	0.02	0.99	(0.95, 1.03)	0.55	1.05	6.03E-01	0.772
Food	Potatoes, baked/boiled/mashed	0.01	0.02	1.01	(0.97, 1.05)	0.26	1.22	6.03E-01	0.772
Food	Crackers	-0.01	0.02	0.99	(0.95, 1.03)	0.47	1.09	6.08E-01	0.772
Nutrient	Glucose	0.01	0.03	1.01	(0.96, 1.07)	0.4	1.89	6.11E-01	0.772
Food	Ice cream	-0.01	0.02	0.99	(0.95, 1.03)	0.13	1.09	6.13E-01	0.772
Food	Cooked onions	-0.01	0.02	0.99	(0.95, 1.03)	0.42	1.05	6.14E-01	0.772
Food	Grapefruit and juice	-0.01	0.02	0.99	(0.95, 1.03)	0.05	1.04	6.16E-01	0.772
Food	Pizza	-0.01	0.02	0.99	(0.95, 1.03)	0.34	1.18	6.17E-01	0.778
Food	Cooked spinach	0.01	0.02	1.01	(0.97, 1.05)	0.6	1.05	6.27E-01	0.778
Nutrient	Gallocatechin	-0.01	0.02	0.99	(0.95, 1.03)	0.98	1.02	6.29E-01	0.778
Nutrient	Sulfur from cysteine	-0.01	0.03	0.99	(0.93, 1.04)	0.42	2.35	6.30E-01	0.778
Food	Margarine	0.01	0.02	1.01	(0.97, 1.05)	0.73	1.08	6.33E-01	0.784
Nutrient	4-Methoxyglucobrassicin	-0.01	0.02	0.99	(0.95, 1.03)	0.03	1.09	6.42E-01	0.788
Nutrient	Total potassium	-0.01	0.03	0.99	(0.93, 1.05)	0.85	2.81	6.49E-01	0.792
Nutrient	Carbohydrate from potato	0.01	0.02	1.01	(0.97, 1.05)	0.62	1.31	6.55E-01	0.792
Food	Pancakes or waffles	0.01	0.02	1.01	(0.97, 1.05)	0.99	1.1	6.57E-01	0.809
Nutrient	Cyanidin	-0.01	0.02	0.99	(0.95, 1.03)	0.41	1.21	6.80E-01	0.809
Nutrient	Theaflavin	-0.01	0.02	0.99	(0.95, 1.03)	0.98	1.02	6.80E-01	0.817
Food	Cream	0.01	0.02	1.01	(0.97, 1.05)	0.4	1.04	6.92E-01	0.824
Nutrient	Proline	0.01	0.04	1.01	(0.94, 1.09)	0.67	3.91	7.01E-01	0.835
Food	banana	-0.01	0.02	0.99	(0.95, 1.03)	0.76	1.13	7.17E-01	0.835
Nutrient	Linolenic acid	-0.01	0.03	0.99	(0.93, 1.05)	0.72	2.51	7.20E-01	0.835
Nutrient	Glucoiberin	0.01	0.02	1.01	(0.97, 1.05)	0.05	1.09	7.22E-01	0.835
Food	Cake	0.01	0.02	1.01	(0.97, 1.05)	0.71	1.18	7.24E-01	0.848
Nutrient	Carbohydrates	0.01	0.04	1.01	(0.93, 1.10)	0.77	5.16	7.45E-01	0.848
Nutrient	Natural vitamin B6	-0.01	0.03	0.99	(0.93, 1.05)	0.52	2.65	7.47E-01	0.848
Food	Beans or lentils	0.01	0.02	1.01	(0.97, 1.05)	0.71	1.1	7.47E-01	0.848
Food	Other fish	0.01	0.02	1.01	(0.97, 1.05)	0.38	1.04	7.49E-01	0.857
Nutrient	Glutamine	0.01	0.04	1.01	(0.93, 1.10)	0.81	4.62	7.61E-01	0.862
Nutrient	Vegetable fiber	-0.01	0.02	0.99	(0.95, 1.04)	0.01	1.36	7.69E-01	0.867
Nutrient	Delta tocopherol	-0.01	0.03	0.99	(0.94, 1.04)	0.83	1.88	7.80E-01	0.867
Food	Other cooked cereal	0.01	0.02	1.01	(0.97, 1.04)	0.36	1.03	7.81E-01	0.888
Nutrient	Dietary zinc	0.01	0.03	1.01	(0.95, 1.07)	0.15	2.71	8.08E-01	0.893
Food	Cauliflower	0	0.02	1	(0.96, 1.03)	0.24	1.04	8.16E-01	0.908
Food	Brown rice	0	0.02	1	(0.96, 1.05)	0.35	1.05	8.38E-01	0.908
Nutrient	Glucoibervirin	0	0.02	1	(0.97, 1.04)	0.16	1.06	8.40E-01	0.908
Food	Shrimp, lobster, scallops	0	0.02	1	(0.97, 1.04)	0.85	1.05	8.41E-01	0.915
Food	Egg	0	0.02	1	(0.96, 1.04)	0.35	1.12	8.53E-01	0.923
Food	Cottage ricotta cheese	0	0.02	1	(0.96, 1.04)	0.04	1.05	8.66E-01	0.923
Food	Chicken liver	0	0.02	1	(0.96, 1.03)	0.97	1.01	8.68E-01	0.925

Supplementary Table 3. cont.

Type	Exposure	Effect size	SE(effect size)	Hazard ratio	95% CI	P-value of PH	VIF	P-value	FDR
Food	Cooked carrot	0	0.02	1	(0.97, 1.04)	0.49	1.09	8.72E-01	0.940
Nutrient	Epicatechin	0	0.02	1	(0.96, 1.04)	0.89	1.19	8.93E-01	0.951
Nutrient	Glucoraphenin	0	0.02	1	(0.96, 1.03)	0.14	1.03	9.10E-01	0.951
Food	Cantaloupe	0	0.02	1	(0.97, 1.04)	0.11	1.04	9.12E-01	0.971
Food	Strawberries	0	0.02	1	(0.96, 1.04)	0.57	1.06	9.38E-01	0.973
Food	Non-dairy coffee whitener	0	0.02	1	(0.97, 1.04)	0.07	1.01	9.43E-01	0.973
Nutrient	Sum of betaine and choline	0	0.03	1	(0.93, 1.07)	0.82	3.19	9.46E-01	0.976
Nutrient	Dietary retinol	0	0.02	1	(0.96, 1.04)	0.42	1.21	9.55E-01	0.976
Nutrient	22:5 fatty acid	0	0.02	1	(0.96, 1.04)	0.74	1.12	9.56E-01	0.978
Nutrient	Carbohdyrate from legumes	0	0.02	1	(0.96, 1.05)	0.04	1.28	9.62E-01	0.979
Food	Potato chips or corn chips	0	0.02	1	(0.96, 1.04)	0.27	1.13	9.67E-01	0.979
Food	Kale	0	0.02	1	(0.96, 1.04)	0.1	1.05	9.69E-01	0.985
Nutrient	Gamma tocotrienol	0	0.02	1	(0.96, 1.05)	0.61	1.29	9.80E-01	0.996
Food	Chicken/turkey, without skin	0	0.02	1	(0.96, 1.04)	0.96	1.04	9.95E-01	0.996

Supplementary Table 4: **EWAS Results for Time-Dose Effects.** All the exposures analyzed in our study, statistically significant or non-significant, are listed here. P-values are associated with two-sided Wald tests.

Type	Exposure	Effect size	SE(effect size)	Hazard ratio	95% CI	P-value of PH	VIF	P-value	FDR
Nutrient	Trans 16:1	0.19	0.03	1.21	(1.15, 1.28)	0.44	1.80	3.17E-13	0.000
Nutrient	Alcohol	-0.14	0.02	0.87	(0.84, 0.91)	0.20	1.11	6.65E-12	0.000
Nutrient	Discretionary solid fat	0.17	0.03	1.19	(1.12, 1.26)	0.64	2.14	1.41E-09	0.005
Nutrient	Discretionary liquid fat	-0.14	0.02	0.87	(0.83, 0.91)	0.75	1.45	6.74E-09	0.006
Nutrient	Trans 18:2	0.15	0.03	1.17	(1.11, 1.23)	0.78	1.57	8.40E-09	0.006
Food	White wine	-0.13	0.02	0.88	(0.84, 0.92)	0.71	1.06	1.27E-08	0.006
Nutrient	Animal fat	0.15	0.03	1.16	(1.10, 1.23)	0.77	2.06	3.30E-08	0.007
Nutrient	Animal MUFA	0.14	0.03	1.15	(1.10, 1.22)	0.86	1.95	6.44E-08	0.008
Nutrient	Palmitoleic acid	0.15	0.03	1.16	(1.10, 1.23)	0.80	2.21	9.10E-08	0.008
Nutrient	Trans 18:1	0.14	0.03	1.16	(1.10, 1.22)	0.86	1.83	1.04E-07	0.008
Nutrient	Sodium	0.16	0.03	1.17	(1.11, 1.25)	0.33	2.29	1.08E-07	0.008
Nutrient	Supplemental or fortified folic acid	-0.12	0.02	0.89	(0.85, 0.93)	0.65	1.63	8.04E-07	0.020
Nutrient	Hydroxyproline	0.12	0.02	1.13	(1.07, 1.18)	0.83	1.59	9.81E-07	0.020
Nutrient	Total trans fat	0.13	0.03	1.14	(1.08, 1.20)	0.45	1.86	1.83E-06	0.022
Nutrient	Carbohydrate from wholegrain	-0.10	0.02	0.90	(0.87, 0.94)	0.32	1.29	1.88E-06	0.022
Nutrient	Carbohydrate from milled wholegrain	-0.10	0.02	0.90	(0.87, 0.94)	0.19	1.28	2.03E-06	0.022
Food	White bread	0.09	0.02	1.09	(1.05, 1.13)	0.03	1.10	2.39E-06	0.022
Nutrient	Added bran from wheat, rice, ...	-0.10	0.02	0.91	(0.87, 0.95)	0.00	1.15	4.48E-06	0.028
Nutrient	Cholesterol	0.11	0.03	1.12	(1.07, 1.18)	0.08	1.82	6.79E-06	0.030
Food	Liquor	-0.09	0.02	0.92	(0.88, 0.95)	0.34	1.05	1.00E-05	0.034
Nutrient	Stearic acid	0.13	0.03	1.14	(1.07, 1.21)	0.33	2.54	1.35E-05	0.036
Food	Apple juice or cider	0.08	0.02	1.08	(1.04, 1.12)	1.00	1.05	1.46E-05	0.036
Nutrient	Natural germ	-0.09	0.02	0.91	(0.87, 0.95)	0.44	1.23	1.49E-05	0.036
Food	Doughnuts	0.08	0.02	1.08	(1.04, 1.12)	0.11	1.09	2.03E-05	0.039
Nutrient	Total saturated fat	0.13	0.03	1.14	(1.07, 1.21)	0.32	2.73	2.95E-05	0.042
Food	Yogurt	-0.09	0.02	0.91	(0.88, 0.95)	0.04	1.06	3.07E-05	0.042
Nutrient	Heme iron	0.10	0.02	1.10	(1.05, 1.16)	0.89	1.58	3.82E-05	0.044
Nutrient	Natural bran	-0.09	0.02	0.92	(0.88, 0.96)	0.46	1.26	3.97E-05	0.044
Nutrient	Palmitic acid	0.13	0.03	1.14	(1.07, 1.22)	0.58	3.04	4.66E-05	0.047
Nutrient	Dietary choline from phosphatidylcholine	0.10	0.03	1.11	(1.05, 1.17)	0.28	2.05	1.14E-04	0.069
Nutrient	Supplemental selenium	-0.08	0.02	0.92	(0.88, 0.96)	0.89	1.34	1.39E-04	0.073
Nutrient	Alpha tocotrienol	-0.09	0.02	0.91	(0.87, 0.96)	0.24	1.46	1.52E-04	0.074
Nutrient	Choline from sphingomyelin	0.10	0.03	1.10	(1.05, 1.16)	0.84	1.82	1.60E-04	0.074
Nutrient	Beta tocopherol	-0.09	0.03	0.91	(0.87, 0.96)	0.19	1.74	2.39E-04	0.086
Nutrient	Dietary folate	-0.09	0.03	0.91	(0.86, 0.96)	0.30	1.80	2.53E-04	0.086
Nutrient	Cereal fiber	-0.08	0.02	0.92	(0.88, 0.96)	0.00	1.46	2.85E-04	0.087
Food	Hamburger	0.07	0.02	1.07	(1.03, 1.11)	0.72	1.14	2.86E-04	0.087
Nutrient	Total manganese	-0.09	0.02	0.92	(0.87, 0.96)	0.66	1.60	3.10E-04	0.087
Food	Salad/oil and vinegar dressing	-0.07	0.02	0.93	(0.90, 0.97)	0.07	1.06	3.20E-04	0.087
Nutrient	Beta tocotrienol	-0.08	0.02	0.93	(0.89, 0.97)	0.19	1.29	3.36E-04	0.087
Nutrient	Threonine	0.11	0.03	1.12	(1.05, 1.20)	0.62	3.01	3.82E-04	0.091
Food	Hotdog	0.06	0.02	1.06	(1.03, 1.10)	0.78	1.06	4.27E-04	0.094
Nutrient	Total vitamin E	-0.08	0.02	0.92	(0.88, 0.96)	0.39	1.80	4.92E-04	0.098
Nutrient	Tryptophan	0.12	0.03	1.13	(1.05, 1.20)	0.35	3.35	5.74E-04	0.100
Food	All processed meats	0.07	0.02	1.07	(1.03, 1.11)	0.53	1.12	6.03E-04	0.100
Food	Raisins or grapes	-0.07	0.02	0.93	(0.90, 0.97)	0.75	1.07	6.15E-04	0.100
Nutrient	Dihydrovitamin K	0.08	0.02	1.09	(1.04, 1.14)	0.60	1.47	6.16E-04	0.100
Nutrient	Supplemental iodine	-0.08	0.02	0.93	(0.89, 0.97)	0.14	1.35	6.81E-04	0.103
Nutrient	Animal protein	0.09	0.03	1.10	(1.04, 1.15)	0.51	2.01	7.32E-04	0.105
Nutrient	Myristic acid	0.08	0.02	1.09	(1.03, 1.14)	0.46	1.75	8.08E-04	0.108
Nutrient	Isorhamnetin	-0.07	0.02	0.93	(0.89, 0.97)	0.27	1.19	8.54E-04	0.108
Nutrient	Phytate	-0.09	0.03	0.92	(0.87, 0.96)	0.00	1.88	8.74E-04	0.108
Food	Pie	0.06	0.02	1.06	(1.03, 1.10)	0.10	1.12	1.05E-03	0.116
Nutrient	Menaquinone vitamin K2 (4 isoprenoid residues)	0.07	0.02	1.08	(1.03, 1.13)	0.05	1.44	1.16E-03	0.119
Nutrient	Dietary choline	0.11	0.03	1.11	(1.04, 1.19)	0.34	3.06	1.19E-03	0.119
Nutrient	Plant MUFA	-0.08	0.03	0.92	(0.88, 0.97)	0.88	1.81	1.23E-03	0.119
Nutrient	Dietary manganese	-0.08	0.03	0.92	(0.87, 0.97)	0.01	1.91	1.61E-03	0.133
Food	Beverages with sugar	0.06	0.02	1.06	(1.02, 1.10)	0.51	1.11	1.69E-03	0.134
Food	Peanuts	-0.07	0.02	0.94	(0.90, 0.98)	0.56	1.06	1.95E-03	0.141
Nutrient	Sulfur from methionine	0.09	0.03	1.10	(1.03, 1.16)	0.97	2.46	1.98E-03	0.141

Supplementary Table 4. cont.

Type	Exposure	Effect size	SE(effect size)	Hazard ratio	95% CI	P-value of PH	VIF	P-value	FDR
Food	Romaine or leaf lettuce	-0.06	0.02	0.94	(0.90, 0.98)	0.38	1.07	2.21E-03	0.145
Nutrient	Total pyridoxine	-0.07	0.02	0.93	(0.89, 0.97)	0.20	1.48	2.31E-03	0.145
Nutrient	Isoleucine	0.10	0.03	1.10	(1.04, 1.18)	0.79	3.02	2.35E-03	0.145
Nutrient	Food supplemented alpha tocopherol	-0.06	0.02	0.94	(0.90, 0.98)	0.77	1.08	2.43E-03	0.145
Nutrient	Histidine	0.10	0.03	1.10	(1.04, 1.18)	0.75	3.09	2.45E-03	0.145
Nutrient	Total magnesium	-0.09	0.03	0.92	(0.87, 0.97)	0.84	2.21	2.57E-03	0.147
Food	Raw carrots	-0.06	0.02	0.94	(0.90, 0.98)	0.03	1.08	3.02E-03	0.155
Nutrient	Sinigrin	0.06	0.02	1.06	(1.02, 1.10)	0.05	1.07	3.05E-03	0.155
Food	Whole milk	0.05	0.02	1.06	(1.02, 1.09)	0.01	1.05	3.28E-03	0.155
Nutrient	Methionine	0.09	0.03	1.10	(1.03, 1.16)	0.97	2.77	3.31E-03	0.155
Nutrient	Glycine	0.09	0.03	1.10	(1.03, 1.17)	0.70	2.83	3.37E-03	0.155
Nutrient	Lysine	0.09	0.03	1.09	(1.03, 1.16)	0.92	2.58	3.40E-03	0.155
Nutrient	Alanine	0.09	0.03	1.10	(1.03, 1.16)	0.80	2.82	3.63E-03	0.158
Food	Olive oil	-0.06	0.02	0.94	(0.90, 0.98)	0.58	1.06	4.04E-03	0.166
Food	Red wine	-0.06	0.02	0.94	(0.90, 0.98)	0.97	1.04	4.89E-03	0.181
Nutrient	Dietary retinol	0.06	0.02	1.06	(1.02, 1.10)	0.94	1.19	5.60E-03	0.193
Food	Beef or lamb as a main dish	0.06	0.02	1.06	(1.02, 1.10)	0.61	1.21	6.00E-03	0.197
Nutrient	Protein	0.10	0.03	1.10	(1.03, 1.18)	0.88	3.49	6.09E-03	0.197
Food	Other nuts	-0.06	0.02	0.94	(0.90, 0.98)	0.54	1.10	7.62E-03	0.214
Nutrient	Choline from phosphatidylcholine	0.07	0.03	1.07	(1.02, 1.12)	0.22	1.87	7.80E-03	0.214
Nutrient	Cysteine	0.10	0.04	1.10	(1.03, 1.18)	0.48	3.78	7.98E-03	0.214
Food	String beans	0.05	0.02	1.05	(1.01, 1.09)	0.27	1.07	8.22E-03	0.214
Nutrient	Malvidin	-0.05	0.02	0.95	(0.91, 0.99)	0.41	1.10	8.38E-03	0.214
Nutrient	Total calcium	-0.06	0.02	0.94	(0.90, 0.99)	0.16	1.37	8.42E-03	0.214
Nutrient	Pantothenic acid	-0.06	0.02	0.94	(0.89, 0.98)	0.19	1.65	8.53E-03	0.214
Food	Beef/pork/lamb as a sandwich	0.05	0.02	1.05	(1.01, 1.10)	0.76	1.18	8.84E-03	0.214
Food	Beef liver	0.05	0.02	1.05	(1.01, 1.08)	0.44	1.01	8.90E-03	0.214
Nutrient	Carbohydrate from intact wholegrain	-0.05	0.02	0.95	(0.91, 0.99)	0.58	1.11	8.94E-03	0.214
Food	Cold breakfast cereal	-0.05	0.02	0.95	(0.91, 0.99)	0.03	1.06	9.02E-03	0.214
Food	Dark m,eat fish	-0.05	0.02	0.95	(0.91, 0.99)	0.38	1.05	9.30E-03	0.215
Nutrient	Total beta carotene	-0.05	0.02	0.95	(0.91, 0.99)	0.12	1.26	9.60E-03	0.216
Nutrient	Dietary tocopherols	-0.08	0.03	0.93	(0.88, 0.98)	0.67	2.35	1.01E-02	0.220
Nutrient	Total fat	0.09	0.04	1.09	(1.02, 1.17)	0.33	3.51	1.04E-02	0.221
Nutrient	Glucocheirolin	0.05	0.02	1.05	(1.01, 1.09)	0.02	1.02	1.09E-02	0.224
Nutrient	Tyrosine	0.08	0.03	1.09	(1.02, 1.16)	0.98	3.17	1.21E-02	0.233
Nutrient	Leucine	0.08	0.03	1.09	(1.02, 1.16)	0.74	3.19	1.21E-02	0.233
Nutrient	Arginine	0.08	0.03	1.08	(1.02, 1.16)	0.63	3.06	1.33E-02	0.240
Food	Muffins or biscuits	0.05	0.02	1.05	(1.01, 1.09)	0.05	1.09	1.35E-02	0.240
Nutrient	Eicosenoic acid	-0.06	0.02	0.94	(0.90, 0.99)	0.26	1.32	1.36E-02	0.240
Nutrient	Asparagine	0.08	0.03	1.09	(1.02, 1.16)	0.56	3.23	1.41E-02	0.240
Nutrient	Sum of sulfur from methionine and cysteine	0.07	0.03	1.07	(1.01, 1.14)	0.84	2.49	1.41E-02	0.240
Nutrient	Synthetic vitamin B6	-0.05	0.02	0.95	(0.92, 0.99)	0.14	1.05	1.42E-02	0.240
Nutrient	Dairy fat	0.05	0.02	1.06	(1.01, 1.10)	0.61	1.45	1.45E-02	0.240
Nutrient	Dietary sulfur	0.09	0.04	1.09	(1.02, 1.17)	0.77	3.51	1.57E-02	0.245
Nutrient	Dietary vitamin B12	0.05	0.02	1.05	(1.01, 1.10)	0.15	1.31	1.58E-02	0.245
Nutrient	Aspartic acid	0.08	0.03	1.08	(1.02, 1.16)	0.97	3.24	1.58E-02	0.245
Nutrient	Glutamic acid	0.09	0.04	1.09	(1.02, 1.18)	0.80	4.05	1.62E-02	0.246
Nutrient	Gluconapin	0.05	0.02	1.05	(1.01, 1.09)	0.03	1.05	1.66E-02	0.246
Nutrient	Fiber from legumes	-0.05	0.02	0.95	(0.91, 0.99)	0.50	1.23	1.67E-02	0.246
Food	Margarine	0.05	0.02	1.05	(1.01, 1.09)	0.86	1.06	1.72E-02	0.246
Nutrient	Vegetable fat	-0.07	0.03	0.94	(0.89, 0.99)	0.51	2.08	1.73E-02	0.246
Nutrient	Valine	0.08	0.03	1.08	(1.01, 1.16)	0.99	3.24	1.76E-02	0.247
Food	Brussels sprouts	0.04	0.02	1.04	(1.01, 1.08)	0.04	1.02	1.81E-02	0.248
Nutrient	Total zinc	-0.05	0.02	0.95	(0.91, 0.99)	0.61	1.46	1.89E-02	0.250
Food	Broccoli	-0.05	0.02	0.95	(0.92, 0.99)	0.02	1.07	1.89E-02	0.250
Nutrient	Hesperetin	-0.05	0.02	0.96	(0.92, 0.99)	0.58	1.10	2.03E-02	0.258
Nutrient	Taurine	0.05	0.02	1.05	(1.01, 1.10)	0.69	1.42	2.09E-02	0.259
Nutrient	Glucoraphanin	-0.04	0.02	0.96	(0.92, 0.99)	0.02	1.07	2.11E-02	0.259
Nutrient	Glucobarbarin	-0.05	0.02	0.95	(0.92, 0.99)	0.03	1.06	2.20E-02	0.263
Nutrient	Total vitamin C	-0.05	0.02	0.95	(0.91, 0.99)	0.65	1.30	2.25E-02	0.265

Supplementary Table 4. cont.

Type	Exposure	Effect size	SE(effect size)	Hazard ratio	95% CI	P-value of PH	VIF	P-value	FDR
Nutrient	Glucoerysolin	0.03	0.02	1.03	(0.99, 1.07)	0.84	1.04	1.25E-01	0.495
Nutrient	Luteolin	-0.03	0.02	0.97	(0.93, 1.01)	0.68	1.17	1.31E-01	0.507
Food	Peas, or lima beans	0.03	0.02	1.03	(0.99, 1.07)	0.78	1.08	1.41E-01	0.529
Nutrient	Carbohydrate from potato	0.03	0.02	1.03	(0.99, 1.08)	0.36	1.26	1.43E-01	0.530
Nutrient	Glucoalyssin	-0.03	0.02	0.97	(0.93, 1.01)	0.01	1.07	1.45E-01	0.531
Nutrient	Lauric acid	0.03	0.02	1.03	(0.99, 1.07)	0.19	1.32	1.51E-01	0.543
Food	Pasta	-0.03	0.02	0.97	(0.93, 1.01)	0.12	1.14	1.53E-01	0.543
Nutrient	Caffeine	0.03	0.02	1.03	(0.99, 1.07)	0.63	1.13	1.54E-01	0.543
Nutrient	Dietary magnesium	-0.05	0.03	0.95	(0.90, 1.02)	0.17	2.96	1.56E-01	0.543
Nutrient	Crude fiber	-0.04	0.03	0.96	(0.91, 1.01)	0.03	1.96	1.58E-01	0.543
Nutrient	Beta cryptoxanthin	-0.03	0.02	0.97	(0.93, 1.01)	0.81	1.20	1.58E-01	0.543
Nutrient	Dietary riboflavin	-0.04	0.03	0.96	(0.91, 1.02)	0.56	2.12	1.59E-01	0.543
Food	Ketchup or red chili sauce	-0.03	0.02	0.97	(0.93, 1.01)	0.54	1.09	1.60E-01	0.544
Nutrient	Gamma tocotrienol	-0.03	0.02	0.97	(0.93, 1.01)	0.52	1.26	1.68E-01	0.556
Nutrient	Delta tocopherol	0.03	0.02	1.03	(0.99, 1.08)	0.79	1.66	1.68E-01	0.556
Food	Apple and pear	-0.03	0.02	0.97	(0.94, 1.01)	0.26	1.08	1.71E-01	0.557
Food	Oranges	-0.03	0.02	0.97	(0.94, 1.01)	0.48	1.05	1.71E-01	0.557
Food	Potatoes, baked/boiled/mashed	0.03	0.02	1.03	(0.99, 1.07)	0.10	1.17	1.75E-01	0.559
Nutrient	Daidzein	-0.03	0.02	0.97	(0.93, 1.01)	0.65	1.08	1.77E-01	0.559
Nutrient	Maltose	0.03	0.02	1.03	(0.99, 1.08)	0.70	1.65	1.78E-01	0.559
Nutrient	Total MUFA	0.04	0.03	1.04	(0.98, 1.11)	0.54	2.88	1.78E-01	0.559
Nutrient	Lycopene	0.03	0.02	1.03	(0.99, 1.07)	0.20	1.26	1.82E-01	0.564
Nutrient	Dietary copper	0.04	0.03	1.04	(0.98, 1.10)	0.15	2.57	1.84E-01	0.564
Food	Orange juice	-0.03	0.02	0.97	(0.94, 1.01)	0.98	1.06	1.88E-01	0.566
Nutrient	Delphinidin	-0.03	0.02	0.97	(0.94, 1.01)	0.55	1.09	1.88E-01	0.566
Nutrient	Sulfur from cysteine	0.04	0.03	1.04	(0.98, 1.09)	0.82	2.20	1.89E-01	0.566
Nutrient	Proline	0.05	0.03	1.05	(0.98, 1.12)	0.60	3.54	1.90E-01	0.566
Nutrient	Petunidin	-0.03	0.02	0.97	(0.94, 1.01)	0.57	1.09	1.94E-01	0.570
Nutrient	Vegetable protein	0.04	0.03	1.04	(0.98, 1.09)	0.92	2.26	2.07E-01	0.592
Nutrient	Dietary iron	-0.03	0.03	0.97	(0.92, 1.02)	0.00	2.08	2.08E-01	0.592
Nutrient	Total iron	-0.03	0.02	0.97	(0.93, 1.02)	0.03	1.45	2.14E-01	0.595
Food	Tomato juice or V-8	0.02	0.02	1.02	(0.99, 1.06)	0.18	1.03	2.15E-01	0.595
Nutrient	Dietary thiamine	-0.04	0.03	0.96	(0.90, 1.02)	0.00	2.80	2.15E-01	0.595
Nutrient	Glucoiberin	0.02	0.02	1.02	(0.99, 1.07)	0.05	1.09	2.16E-01	0.595
Nutrient	Sum of betaine and choline	0.04	0.03	1.04	(0.98, 1.11)	0.77	3.10	2.19E-01	0.597
Nutrient	Naringenin	-0.02	0.02	0.98	(0.94, 1.02)	0.64	1.11	2.25E-01	0.606
Nutrient	Acrylamide	-0.03	0.02	0.97	(0.93, 1.02)	0.42	1.42	2.27E-01	0.607
Food	Decaffeinated coffee	-0.02	0.02	0.98	(0.94, 1.01)	0.23	1.00	2.33E-01	0.615
Nutrient	Added sugar	0.03	0.02	1.03	(0.98, 1.08)	0.55	1.67	2.54E-01	0.648
Food	Cream	0.02	0.02	1.02	(0.98, 1.06)	0.44	1.04	2.60E-01	0.652
Food	Yellow squash	0.02	0.02	1.02	(0.98, 1.06)	0.27	1.04	2.60E-01	0.652
Food	Cooked onions	0.02	0.02	1.02	(0.98, 1.06)	0.90	1.04	2.65E-01	0.657
Food	Corn	0.02	0.02	1.02	(0.98, 1.06)	0.40	1.08	2.69E-01	0.658
Nutrient	Myricetin	-0.02	0.02	0.98	(0.94, 1.02)	0.44	1.12	2.69E-01	0.658
Nutrient	6'7'-Dihydroxybergamottin	0.02	0.02	1.02	(0.98, 1.06)	0.00	1.07	2.74E-01	0.661
Nutrient	Carbohydrate from refined grain	0.03	0.03	1.03	(0.98, 1.08)	0.46	1.74	2.76E-01	0.661
Food	Pancakes or waffles	0.02	0.02	1.02	(0.98, 1.06)	0.62	1.07	2.77E-01	0.661
Food	Beer	-0.02	0.02	0.98	(0.94, 1.02)	0.48	1.03	2.84E-01	0.668
Nutrient	Oleic acid	0.03	0.03	1.03	(0.97, 1.10)	0.63	2.78	2.85E-01	0.668
Nutrient	Bergamottin	0.02	0.02	1.02	(0.98, 1.06)	0.00	1.07	2.87E-01	0.668
Food	Ice cream	0.02	0.02	1.02	(0.98, 1.06)	0.28	1.07	3.00E-01	0.683
Nutrient	Total dietary omega 3 (no alpha 18:3)	-0.02	0.02	0.98	(0.94, 1.02)	0.65	1.13	3.01E-01	0.683
Nutrient	Dietary pyridoxine	-0.03	0.03	0.97	(0.92, 1.03)	0.12	2.15	3.03E-01	0.684
Nutrient	Dietary long chain fatty acid	-0.02	0.02	0.98	(0.94, 1.02)	0.71	1.14	3.05E-01	0.684
Nutrient	Dietary beta carotene	-0.02	0.02	0.98	(0.94, 1.02)	0.15	1.26	3.10E-01	0.688
Nutrient	Glucoibervirin	0.02	0.02	1.02	(0.98, 1.06)	0.08	1.06	3.16E-01	0.694
Nutrient	Total free choline	-0.03	0.03	0.97	(0.92, 1.03)	0.32	2.37	3.22E-01	0.698
Nutrient	Starch	-0.03	0.03	0.97	(0.92, 1.03)	0.12	2.21	3.22E-01	0.698
Nutrient	Carbohdyrate from legumes	0.02	0.02	1.02	(0.98, 1.06)	0.05	1.24	3.26E-01	0.700
Nutrient	Proanthocyanidin, dimers	-0.02	0.02	0.98	(0.94, 1.02)	0.85	1.25	3.28E-01	0.700

Supplementary Table 4. cont.

Type	Exposure	Effect size	SE(effect size)	Hazard ratio	95% CI	P-value of PH	VIF	P-value	FDR
Nutrient	Vegetable fiber	0.02	0.02	1.02	(0.98, 1.07)	0.03	1.34	3.32E-01	0.702
Food	Wholegrain bread	-0.02	0.02	0.98	(0.95, 1.02)	0.18	1.07	3.38E-01	0.708
Nutrient	Bergapten	0.02	0.02	1.02	(0.98, 1.06)	0.44	1.09	3.51E-01	0.717
Nutrient	Alpha carotene	-0.02	0.02	0.98	(0.94, 1.02)	0.08	1.16	3.53E-01	0.717
Nutrient	Glycitein	-0.02	0.02	0.98	(0.94, 1.02)	0.09	1.06	3.54E-01	0.717
Nutrient	Dietary zinc	0.03	0.03	1.03	(0.97, 1.09)	0.34	2.48	3.54E-01	0.717
Nutrient	Delta tocotrienol	-0.02	0.02	0.98	(0.94, 1.02)	0.76	1.14	3.55E-01	0.717
Food	Jams, jellies	-0.02	0.02	0.98	(0.95, 1.02)	0.70	1.10	3.58E-01	0.718
Food	Raw spinach	-0.02	0.02	0.98	(0.94, 1.02)	0.87	1.05	3.61E-01	0.718
Food	Candy without chocolate	-0.02	0.02	0.98	(0.95, 1.02)	0.70	1.04	3.62E-01	0.718
Food	Tomatoes	-0.02	0.02	0.98	(0.95, 1.02)	0.38	1.07	3.65E-01	0.719
Nutrient	Total furocoumarins	0.02	0.02	1.02	(0.98, 1.06)	0.00	1.07	3.70E-01	0.723
Food	Potato chips or corn chips	0.02	0.02	1.02	(0.98, 1.06)	0.27	1.11	3.74E-01	0.726
Food	Mixed vegetables	0.02	0.02	1.02	(0.98, 1.06)	0.03	1.07	3.76E-01	0.726
Nutrient	Caprylic acid	0.02	0.02	1.02	(0.98, 1.06)	0.18	1.31	3.81E-01	0.729
Nutrient	Gluten	-0.02	0.02	0.98	(0.93, 1.03)	0.36	1.66	3.83E-01	0.729
Food	Cake	0.02	0.02	1.02	(0.98, 1.06)	0.26	1.13	3.85E-01	0.729
Food	banana	-0.02	0.02	0.98	(0.95, 1.02)	0.55	1.09	3.89E-01	0.731
Nutrient	Glutamine	0.03	0.04	1.03	(0.96, 1.11)	0.54	4.19	3.91E-01	0.732
Food	Yams or sweet potatoes	-0.02	0.02	0.98	(0.95, 1.02)	0.14	1.07	4.00E-01	0.740
Food	Tofu or soybeans	-0.02	0.02	0.98	(0.94, 1.03)	0.01	1.03	4.27E-01	0.768
Food	Blueberries	-0.02	0.02	0.98	(0.94, 1.02)	0.59	1.05	4.27E-01	0.768
Nutrient	Natural vitamin B6	0.02	0.03	1.02	(0.96, 1.09)	0.22	2.71	4.32E-01	0.771
Food	Grapefruit and juice	0.02	0.02	1.02	(0.98, 1.06)	0.01	1.05	4.39E-01	0.775
Food	Salsa	-0.02	0.02	0.98	(0.94, 1.03)	0.73	1.04	4.40E-01	0.775
Food	Popcorn	-0.02	0.02	0.98	(0.94, 1.03)	0.41	1.09	4.50E-01	0.785
Nutrient	Dietary vitamin C	-0.02	0.02	0.98	(0.94, 1.03)	0.12	1.39	4.55E-01	0.787
Food	Kale	-0.02	0.02	0.98	(0.95, 1.03)	0.51	1.04	4.58E-01	0.787
Food	Other fruit juices	0.01	0.02	1.01	(0.98, 1.05)	0.29	1.05	4.62E-01	0.787
Nutrient	Proanthocyanidin, trimers	-0.02	0.02	0.98	(0.94, 1.03)	0.64	1.41	4.62E-01	0.787
Nutrient	Formononetin	-0.01	0.02	0.99	(0.95, 1.03)	0.73	1.09	4.65E-01	0.787
Nutrient	Fructose	0.02	0.02	1.02	(0.97, 1.07)	0.32	1.67	4.67E-01	0.787
Food	Chicken/turkey, without skin	0.01	0.02	1.01	(0.98, 1.05)	0.67	1.05	4.76E-01	0.795
Food	Beans or lentils	0.01	0.02	1.01	(0.98, 1.05)	0.67	1.08	4.82E-01	0.799
Nutrient	Choline from phosphocholine	0.02	0.02	1.02	(0.97, 1.07)	0.65	1.74	4.85E-01	0.799
Nutrient	22:5 fatty acid	0.01	0.02	1.01	(0.97, 1.06)	0.84	1.09	4.88E-01	0.800
Food	Skim milk	0.01	0.02	1.01	(0.98, 1.05)	0.43	1.05	4.93E-01	0.803
Nutrient	EPA fatty acid	-0.01	0.02	0.99	(0.95, 1.03)	0.43	1.06	4.97E-01	0.803
Nutrient	Total flavonoids	-0.01	0.02	0.99	(0.95, 1.03)	0.55	1.18	4.98E-01	0.803
Nutrient	Choline derivative betaine	-0.02	0.02	0.98	(0.94, 1.03)	0.01	1.68	5.07E-01	0.807
Food	Eggplnt, zucchini, summer squash	-0.01	0.02	0.99	(0.95, 1.03)	0.95	1.05	5.07E-01	0.807
Nutrient	Napoleiferin	-0.01	0.02	0.99	(0.95, 1.03)	0.01	1.07	5.15E-01	0.810
Nutrient	Total long chain fatty acid	-0.01	0.02	0.99	(0.95, 1.03)	0.64	1.09	5.17E-01	0.810
Food	Egg	0.01	0.02	1.01	(0.97, 1.05)	0.07	1.08	5.21E-01	0.810
Nutrient	Kaempferol	-0.01	0.02	0.99	(0.95, 1.03)	0.71	1.10	5.21E-01	0.810
Nutrient	Bergaptol	0.01	0.02	1.01	(0.97, 1.05)	0.01	1.06	5.24E-01	0.810
Nutrient	Epicatechin	0.01	0.02	1.01	(0.97, 1.05)	0.56	1.19	5.25E-01	0.810
Nutrient	Lactose	-0.01	0.02	0.99	(0.95, 1.03)	0.60	1.20	5.32E-01	0.811
Nutrient	Lutein and zeaxanthin	-0.01	0.02	0.99	(0.95, 1.03)	0.43	1.23	5.33E-01	0.811
Food	Peanut butter	-0.01	0.02	0.99	(0.95, 1.03)	0.42	1.07	5.35E-01	0.811
Food	Chocolate bars	0.01	0.02	1.01	(0.97, 1.05)	0.78	1.07	5.39E-01	0.811
Food	Onions as a garnish	-0.01	0.02	0.99	(0.95, 1.03)	0.35	1.05	5.39E-01	0.811
Nutrient	Omega 3 (20:5+22:6, no alpha 18:3)	-0.01	0.02	0.99	(0.95, 1.03)	0.63	1.09	5.48E-01	0.815
Nutrient	Glucoerucin	-0.01	0.02	0.99	(0.95, 1.03)	0.04	1.04	5.49E-01	0.815
Nutrient	Gamma tocopherol	-0.02	0.03	0.98	(0.94, 1.04)	0.92	1.96	5.51E-01	0.815
Nutrient	Dietary niacin	-0.02	0.03	0.98	(0.93, 1.04)	0.13	2.50	5.63E-01	0.823
Nutrient	Proanthocyanidin, monomers	-0.01	0.02	0.99	(0.95, 1.03)	0.46	1.14	5.65E-01	0.823
Nutrient	Biochanin A	-0.01	0.02	0.99	(0.95, 1.03)	0.85	1.10	5.67E-01	0.823
Food	Celery	0.01	0.02	1.01	(0.97, 1.05)	0.98	1.06	5.72E-01	0.824
Nutrient	Oxalate	-0.01	0.02	0.99	(0.94, 1.03)	0.34	1.38	5.73E-01	0.824

Supplementary Table 4. cont.

Type	Exposure	Effect size	SE(effect size)	Hazard ratio	95% CI	P-value of PH	VIF	P-value	FDR
Nutrient	Total sugar	0.02	0.03	1.02	(0.96, 1.08)	0.94	2.36	5.78E-01	0.824
Food	Non-dairy coffee whitener	0.01	0.02	1.01	(0.97, 1.05)	0.08	1.01	5.79E-01	0.824
Nutrient	Dietary betaine	-0.01	0.02	0.99	(0.94, 1.03)	0.01	1.68	5.86E-01	0.828
Nutrient	Flavonoids (no proanthocyanidins)	-0.01	0.02	0.99	(0.95, 1.03)	0.38	1.11	5.88E-01	0.828
Nutrient	Natural sugar	-0.01	0.02	0.99	(0.94, 1.04)	0.96	1.68	5.96E-01	0.833
Food	Cooked carrot	0.01	0.02	1.01	(0.97, 1.05)	0.25	1.06	6.00E-01	0.835
Food	Cooked spinach	0.01	0.02	1.01	(0.97, 1.05)	0.66	1.04	6.05E-01	0.837
Food	Other cooked cereal	-0.01	0.02	0.99	(0.95, 1.03)	0.37	1.02	6.11E-01	0.840
Nutrient	Dietary phylloquinone vitamin K1	0.01	0.02	1.01	(0.97, 1.05)	0.08	1.28	6.34E-01	0.858
Nutrient	Alpha linolenic	-0.01	0.03	0.99	(0.94, 1.04)	0.16	1.81	6.35E-01	0.858
Nutrient	DHA fatty acid	-0.01	0.02	0.99	(0.95, 1.03)	0.99	1.10	6.40E-01	0.861
Food	Strawberries	0.01	0.02	1.01	(0.97, 1.05)	0.74	1.05	6.52E-01	0.870
Nutrient	Fiber from cruciferous veg	-0.01	0.02	0.99	(0.95, 1.03)	0.12	1.12	6.57E-01	0.872
Nutrient	Glycosolated B6	-0.01	0.02	0.99	(0.94, 1.04)	0.00	1.68	6.60E-01	0.872
Food	Other cheese	0.01	0.02	1.01	(0.97, 1.05)	0.20	1.09	6.68E-01	0.877
Nutrient	Dairy vitamin D	0.01	0.02	1.01	(0.97, 1.05)	0.21	1.15	6.77E-01	0.883
Food	Green/red peppers	0.01	0.02	1.01	(0.97, 1.05)	0.82	1.05	6.80E-01	0.883
Nutrient	4-Hydroxyglucobrassicin	-0.01	0.02	0.99	(0.95, 1.03)	0.01	1.08	6.98E-01	0.897
Nutrient	Dietary vitamin D	-0.01	0.02	0.99	(0.95, 1.03)	0.24	1.29	7.03E-01	0.899
Nutrient	Linolenic acid	0.01	0.03	1.01	(0.96, 1.06)	0.23	1.96	7.30E-01	0.922
Food	Shrimp, lobster, scallops	0.01	0.02	1.01	(0.97, 1.05)	0.51	1.05	7.41E-01	0.926
Nutrient	Dietary potassium	-0.01	0.03	0.99	(0.93, 1.05)	0.87	2.97	7.42E-01	0.926
Food	Tomato sauce	0.01	0.02	1.01	(0.97, 1.05)	0.93	1.15	7.45E-01	0.927
Food	Canned tuna	-0.01	0.02	0.99	(0.95, 1.03)	0.47	1.08	7.50E-01	0.928
Nutrient	Eriodictyol	-0.01	0.02	0.99	(0.96, 1.03)	0.96	1.08	7.56E-01	0.929
Nutrient	Sugar from fruit juice	-0.01	0.02	0.99	(0.96, 1.03)	0.91	1.11	7.58E-01	0.929
Nutrient	Total flavan-3-ols	-0.01	0.02	0.99	(0.96, 1.03)	0.77	1.10	7.60E-01	0.929
Food	White rice	-0.01	0.02	0.99	(0.95, 1.04)	0.04	1.13	7.63E-01	0.929
Nutrient	Total phospherous	-0.01	0.03	0.99	(0.93, 1.06)	0.73	3.22	7.71E-01	0.933
Food	Cottage ricotta cheese	0.01	0.02	1.01	(0.97, 1.04)	0.30	1.04	7.92E-01	0.947
Food	Cauliflower	0.01	0.02	1.01	(0.97, 1.04)	0.09	1.05	7.93E-01	0.947
Nutrient	Phylloquinone vitamin K1	0.01	0.02	1.01	(0.96, 1.05)	0.12	1.28	8.06E-01	0.951
Nutrient	Gluconasturtiin	0.00	0.02	1.00	(0.96, 1.03)	0.01	1.05	8.13E-01	0.951
Food	Pizza	0.00	0.02	1.01	(0.96, 1.05)	0.33	1.17	8.15E-01	0.951
Nutrient	Dietary alpha tocopherol	-0.01	0.03	0.99	(0.94, 1.05)	0.31	2.00	8.22E-01	0.951
Food	Crackers	0.00	0.02	1.00	(0.97, 1.04)	0.47	1.08	8.23E-01	0.951
Food	Tea	0.00	0.02	1.00	(0.96, 1.03)	0.33	1.02	8.24E-01	0.951
Nutrient	Thearubigins	0.00	0.02	1.00	(0.96, 1.03)	0.79	1.04	8.31E-01	0.951
Nutrient	Epigallocatechin	0.00	0.02	1.00	(0.96, 1.03)	0.83	1.05	8.32E-01	0.951
Nutrient	Total omega 3	0.01	0.03	1.01	(0.96, 1.06)	0.20	1.71	8.36E-01	0.951
Food	Brown rice	0.00	0.02	1.00	(0.96, 1.05)	0.75	1.04	8.36E-01	0.951
Nutrient	Sucrose	0.01	0.03	1.01	(0.96, 1.06)	0.72	1.90	8.37E-01	0.951
Nutrient	Glucose	0.01	0.03	1.01	(0.96, 1.06)	0.36	1.80	8.38E-01	0.951
Nutrient	Dairy calcium	0.00	0.02	1.00	(0.95, 1.04)	0.39	1.31	8.39E-01	0.951
Nutrient	4-Methoxyglucobrassicin	0.00	0.02	1.00	(0.97, 1.04)	0.02	1.09	8.51E-01	0.958
Food	Low caff beverages	0.00	0.02	1.00	(0.96, 1.04)	0.00	1.10	8.63E-01	0.966
Nutrient	Carbohydrates	-0.01	0.04	0.99	(0.92, 1.08)	0.25	4.62	8.78E-01	0.976
Nutrient	Dairy protein	0.00	0.02	1.00	(0.96, 1.04)	0.52	1.34	8.93E-01	0.986
Nutrient	Total retinol	0.00	0.02	1.00	(0.96, 1.04)	0.90	1.34	9.09E-01	0.988
Nutrient	Dietary calcium	0.00	0.02	1.00	(0.95, 1.05)	0.63	1.72	9.11E-01	0.988
Nutrient	Glucoraphenin	0.00	0.02	1.00	(0.97, 1.04)	0.05	1.02	9.14E-01	0.988
Nutrient	Theaflavin	0.00	0.02	1.00	(0.96, 1.04)	0.75	1.03	9.14E-01	0.988
Nutrient	Cystine	0.00	0.03	1.00	(0.94, 1.06)	0.32	2.95	9.23E-01	0.988
Nutrient	Dietary phosphorous	0.00	0.03	1.00	(0.94, 1.07)	0.79	3.29	9.29E-01	0.988
Food	Cantaloupe	0.00	0.02	1.00	(0.96, 1.04)	0.19	1.03	9.34E-01	0.988
Nutrient	Total theaflavins and polymers proanthocyanidis	0.00	0.02	1.00	(0.96, 1.04)	0.91	1.14	9.38E-01	0.988
Nutrient	Total potassium	0.00	0.03	1.00	(0.94, 1.07)	0.99	2.83	9.38E-01	0.988
Food	Mayo	0.00	0.02	1.00	(0.96, 1.04)	0.97	1.11	9.39E-01	0.988
Nutrient	Total folate	0.00	0.02	1.00	(0.95, 1.05)	0.04	1.50	9.40E-01	0.988
Nutrient	Dietary omega 6 (no gamma 18:3)	0.00	0.03	1.00	(0.95, 1.06)	0.24	2.25	9.40E-01	0.988

Supplementary Table 4. cont.

Type	Exposure	Effect size	SE(effect size)	Hazard ratio	95% CI	P-value of PH	VIF	P-value	FDR
Nutrient	Pelargonidin	0.00	0.02	1.00	(0.96, 1.04)	0.69	1.07	9.43E-01	0.988
Nutrient	Dietary free choline	0.00	0.03	1.00	(0.94, 1.07)	0.25	2.73	9.50E-01	0.988
Nutrient	Total glucosinolates	0.00	0.02	1.00	(0.96, 1.04)	0.01	1.10	9.50E-01	0.988
Nutrient	Total dietary omega 3	0.00	0.03	1.00	(0.95, 1.05)	0.07	1.86	9.56E-01	0.988
Nutrient	Epicatechin 3-gallate	0.00	0.02	1.00	(0.96, 1.04)	0.81	1.03	9.58E-01	0.988
Nutrient	Glucobrassicin	0.00	0.02	1.00	(0.96, 1.04)	0.02	1.09	9.64E-01	0.988
Food	Cabbage	0.00	0.02	1.00	(0.96, 1.04)	0.95	1.06	9.66E-01	0.988
Food	English muffins/bagels/rolls	0.00	0.02	1.00	(0.96, 1.04)	0.53	1.09	9.67E-01	0.988
Nutrient	Dietary vitamin A	0.00	0.02	1.00	(0.96, 1.04)	0.03	1.30	9.71E-01	0.988
Nutrient	Total polyunsaturated fat	0.00	0.03	1.00	(0.95, 1.06)	0.51	2.28	9.72E-01	0.988
Nutrient	Nitrate	0.00	0.02	1.00	(0.96, 1.04)	0.06	1.22	9.74E-01	0.988
Nutrient	Gallocatechin	0.00	0.02	1.00	(0.96, 1.04)	0.65	1.03	9.82E-01	0.991
Nutrient	Choline from glycerophosphocholine	0.00	0.02	1.00	(0.95, 1.05)	0.46	1.60	9.90E-01	0.993
Nutrient	Linoleic acid	0.00	0.03	1.00	(0.95, 1.06)	0.67	2.21	9.91E-01	0.993

Supplementary Table 5: **Validation in NHS II.** From 54 statistically significant exposures found in NHS, 38 were validated in NHS II. P-values are associated with two-sided Wald tests.

Type	Exposure	Effect size	SE(effect size)	Hazard ratio	95% CI	P-value of PH	VIF	p-value
Nutrient	Cereal fiber	-0.28	0.06	0.75	(0.68, 0.84)	0.7	1.62	2.70E-07
Nutrient	Total manganese	-0.28	0.06	0.76	(0.68, 0.85)	0.32	1.68	5.82E-07
Nutrient	Alcohol	-0.21	0.04	0.81	(0.74, 0.88)	0.3	1.12	1.06E-06
Nutrient	Beta tocotrienol	-0.25	0.05	0.78	(0.70, 0.86)	0.56	1.39	1.92E-06
Nutrient	Alpha tocotrienol	-0.25	0.05	0.78	(0.70, 0.87)	0.84	1.52	3.64E-06
Nutrient	Dietary manganese	-0.28	0.06	0.76	(0.68, 0.85)	0.74	1.96	3.72E-06
Nutrient	Added Bran from wheat, rice, ...	-0.21	0.05	0.81	(0.74, 0.89)	0.79	1.13	5.86E-06
Food	Cold breakfast cereal	-0.2	0.05	0.82	(0.74, 0.89)	0.16	1.11	1.18E-05
Nutrient	Dietary folate	-0.25	0.06	0.78	(0.69, 0.88)	0.08	2.03	3.50E-05
Nutrient	Natural bran	-0.2	0.05	0.82	(0.75, 0.90)	0.73	1.25	3.62E-05
Nutrient	Carb from milled wholegrain	-0.2	0.05	0.82	(0.75, 0.90)	0.69	1.26	3.90E-05
Nutrient	Stearic acid	0.28	0.07	1.32	(1.15, 1.51)	0.45	2.64	6.26E-05
Nutrient	Carb from wholegrain	-0.19	0.05	0.83	(0.75, 0.91)	1	1.28	6.47E-05
Food	Salad/oil and vinegar dressing	-0.16	0.05	0.85	(0.78, 0.93)	0.15	1.17	3.41E-04
Food	Raw carrots	-0.17	0.05	0.84	(0.77, 0.93)	0.93	1.14	4.02E-04
Food	Red wine	-0.18	0.05	0.84	(0.76, 0.93)	0.72	1.07	7.00E-04
Nutrient	Phytate	-0.21	0.06	0.81	(0.71, 0.92)	0.34	2.18	9.66E-04
Nutrient	Beta tocopherol	-0.21	0.06	0.81	(0.71, 0.92)	0.9	2.08	1.01E-03
Nutrient	Apigenin	-0.15	0.04	0.86	(0.79, 0.94)	0.86	1.2	1.07E-03
Nutrient	Supplemental or fortified folic acid	-0.17	0.05	0.85	(0.77, 0.94)	0.03	1.41	1.38E-03
Nutrient	Discretionary solid fat	0.21	0.07	1.23	(1.08, 1.41)	0.72	2.46	1.49E-03
Nutrient	Natural germ	-0.14	0.05	0.87	(0.79, 0.95)	0.18	1.27	2.42E-03
Nutrient	Total saturated fat	0.22	0.07	1.25	(1.08, 1.44)	0.65	3.04	2.88E-03
Nutrient	Trans 16:1	0.17	0.06	1.19	(1.06, 1.33)	0.47	2.06	3.27E-03
Nutrient	Palmitic acid	0.23	0.08	1.25	(1.08, 1.46)	0.94	3.31	3.47E-03
Food	Beverages with sugar	0.12	0.04	1.12	(1.04, 1.22)	0.29	1.11	4.38E-03
Food	White wine	-0.14	0.05	0.87	(0.79, 0.96)	0.83	1.07	4.40E-03
Nutrient	Synthetic vitamin B6	-0.13	0.05	0.88	(0.80, 0.96)	0.16	1.08	4.92E-03
Nutrient	Trans 18:1	0.15	0.06	1.16	(1.04, 1.29)	0.17	1.71	7.17E-03
Nutrient	Supplemental selenium	-0.12	0.05	0.89	(0.81, 0.97)	0.09	1.28	7.41E-03
Nutrient	Palmitoleic acid	0.16	0.06	1.18	(1.04, 1.33)	0.77	2.19	9.24E-03
Nutrient	Animal fat	0.16	0.06	1.18	(1.04, 1.33)	0.97	2.23	1.03E-02
Nutrient	Animal MUFA	0.15	0.06	1.16	(1.03, 1.31)	0.67	2.03	1.18E-02
Food	Hotdog	0.12	0.05	1.12	(1.03, 1.23)	0.75	1.1	1.18E-02
Food	Raisins or grapes	-0.12	0.05	0.89	(0.80, 0.98)	0.28	1.08	1.90E-02
Nutrient	Hydroxyproline	0.12	0.05	1.12	(1.01, 1.25)	0.15	1.55	3.22E-02
Food	Yogurt	-0.09	0.05	0.91	(0.83, 1.00)	0.14	1.1	3.95E-02
Nutrient	Isorhamnetin	-0.08	0.04	0.92	(0.84, 1.00)	0.95	1.14	5.90E-02
Nutrient	Heme iron	0.10	0.05	1.10	(0.99, 1.22)	0.35	1.53	6.91E-02
Nutrient	Dietary tocopherols	-0.12	0.07	0.89	(0.77, 1.02)	0.50	2.81	8.60E-02
Nutrient	Discretionary liquid fat	-0.08	0.05	0.92	(0.83, 1.02)	0.91	1.63	1.17E-01
Nutrient	Myristic acid	0.07	0.06	1.07	(0.96, 1.20)	0.67	1.98	2.23E-01
Food	Liquor	-0.05	0.04	0.95	(0.88, 1.03)	0.83	1.02	2.27E-01
Nutrient	Sodium	-0.09	0.08	0.91	(0.78, 1.07)	1.00	3.97	2.48E-01
Food	Peanuts	-0.03	0.04	0.97	(0.89, 1.05)	0.75	1.08	4.33E-01
Nutrient	Cholesterol	0.05	0.06	1.05	(0.93, 1.18)	0.71	2.02	4.34E-01
Nutrient	Plant MUFA	-0.04	0.06	0.96	(0.86, 1.08)	0.65	2.03	4.96E-01
Food	All processed meats	0.02	0.04	1.02	(0.94, 1.11)	0.50	1.12	6.27E-01
Food	Doughnuts	0.02	0.04	1.02	(0.94, 1.10)	0.03	1.06	6.52E-01
Food	Apple juice or cider	0.02	0.04	1.02	(0.93, 1.11)	0.43	1.06	6.87E-01
Nutrient	Trans 18:2	-0.02	0.05	0.98	(0.88, 1.09)	0.53	1.74	7.64E-01
Food	Hamburger	0.01	0.04	1.01	(0.93, 1.10)	0.93	1.20	7.74E-01
Food	White bread	-0.01	0.04	0.99	(0.91, 1.08)	0.01	1.11	7.80E-01

Supplementary Table 6: **Hazard Ratios of CHD by quintiles of intakes of each exposure, with first quintile as baseline.** To help interpret our findings, we calculated the HR of each quintile of exposure intake compared with the first quintile, as a reference group. For example, for isorhamnetin, the HR for Q2 (the second quntile of consumption) compared to Q1 (the first quintile of consumption) is 0.94. The HR for Q3 and Q4 compared to Q1 are 0.88 and 0.82, respectively. The HR for Q5 (which is the maximum level of isorhamnetin consumption) is 0.73, compared to Q1. This decreasing trend illustrates the protective association of isorhamnetin with CHD.

Type	Exposure	Hazard ratio	Q1 (20%)	Q2 (40%)	Q3 (60%)	Q4 (80%)	Q5 (100%)
Nutrient	Alcohol	0.88	1	0.90	0.82	0.74	0.63
Nutrient	Added bran from wheat, rice, oat, corn	0.87	1	0.89	0.81	0.75	0.57
Nutrient	Trans 16:1	1.19	1	1.09	1.19	1.32	3.31
Nutrient	Discretionary liquid fat	0.86	1	0.92	0.85	0.78	0.38
Nutrient	Animal MUFA	1.17	1	1.09	1.18	1.30	2.84
Nutrient	Discretionary solid fat	1.18	1	1.10	1.20	1.32	2.75
Food	White wine	0.89	1	0.99	0.93	0.85	0.58
Nutrient	Palmitoleic acid	1.17	1	1.10	1.19	1.31	2.45
Nutrient	Animal fat	1.17	1	1.09	1.18	1.29	2.73
Food	Salad/oil and vinegar dressing	0.90	1	0.94	0.87	0.82	0.67
Food	Yogurt	0.90	1	0.97	0.92	0.83	0.59
Nutrient	Phytate	0.88	1	0.93	0.87	0.81	0.33
Nutrient	Stearic acid	1.16	1	1.09	1.17	1.28	2.31
Nutrient	Carbohydrate from milled wholegrain	0.91	1	0.93	0.88	0.83	0.55
Nutrient	Sodium	1.14	1	1.08	1.15	1.24	2.12
Food	Raw carrots	0.91	1	0.97	0.91	0.83	0.68
Nutrient	Total saturated fat	1.16	1	1.09	1.17	1.28	2.44
Nutrient	Hydroxyproline	1.12	1	1.06	1.12	1.20	1.91
Nutrient	Isorhamnetin	0.91	1	0.94	0.88	0.82	0.73
Food	Liquor	0.92	1	1.00	0.99	0.92	0.71
Nutrient	Carbohydrate from wholegrain	0.91	1	0.94	0.90	0.85	0.51
Nutrient	Cereal fiber	0.91	1	0.94	0.90	0.85	0.37
Food	Red wine	0.91	1	1.00	0.98	0.93	0.56
Nutrient	Trans 18:2	1.12	1	1.06	1.12	1.20	1.83
Nutrient	Dietary tocopherols	0.88	1	0.93	0.87	0.81	0.34
Nutrient	Palmitic acid	1.15	1	1.08	1.16	1.26	2.22
Nutrient	Dietary folate	0.90	1	0.94	0.89	0.84	0.38
Food	Doughnuts	1.08	1	1.02	1.06	1.09	1.90
Nutrient	Beta tocotrienol	0.92	1	0.95	0.90	0.85	0.71
Nutrient	Plant MUFA	0.90	1	0.94	0.89	0.84	0.48
Food	Hotdog	1.07	1	1.05	1.09	1.13	1.97
Food	White bread	1.08	1	1.04	1.12	1.17	1.26
Nutrient	Natural germ	0.92	1	0.94	0.91	0.86	0.65
Nutrient	Apigenin	0.92	1	0.96	0.92	0.87	0.74
Nutrient	Beta tocopherol	0.91	1	0.95	0.90	0.85	0.49
Nutrient	Natural bran	0.92	1	0.95	0.91	0.87	0.60
Nutrient	Supplemental selenium	0.92	1	1.00	0.95	0.85	0.81
Food	Apple juice or cider	1.07	1	1.00	1.03	1.06	1.60
Nutrient	Dietary manganese	0.91	1	0.95	0.90	0.85	0.57
Food	Peanuts	0.93	1	0.98	0.95	0.91	0.60
Nutrient	Alpha tocotrienol	0.92	1	0.95	0.91	0.86	0.67
Nutrient	Myristic acid	1.10	1	1.06	1.10	1.16	1.66
Nutrient	Cholesterol	1.10	1	1.05	1.10	1.16	2.19
Nutrient	Supplemental or fortified folic acid	0.92	1	0.94	0.89	0.85	0.59
Food	All processed meats	1.07	1	1.04	1.06	1.11	1.51
Nutrient	Trans 18:1	1.09	1	1.06	1.11	1.17	1.58
Nutrient	Total manganese	0.92	1	0.95	0.91	0.88	0.69
Food	Hamburger	1.07	1	1.03	1.06	1.09	1.57
Food	Beverages with sugar	1.07	1	1.02	1.06	1.13	1.29
Nutrient	Synthetic vitamin B6	0.94	1	0.97	0.92	0.88	0.73
Food	Cold breakfast cereal	0.94	1	0.95	0.91	0.88	0.74
Food	Raisins or grapes	0.93	1	0.96	0.95	0.89	0.67
Nutrient	Heme iron	1.08	1	1.04	1.08	1.14	1.85

Supplementary Table 7: **EWAS Results for Polyphenols.** Polyphenols are a large family of phytochemicals with great chemical diversity, known to be bioactive compounds of foods, species, medicinal plants and nutraceuticals, with various positive health effects [28]. In our analysis, we covered a wide range of polyphenols. Among 44 pholyphenol-related exposures, 17 were found to be significantly associated with lower CHD risk meeting a 0.05 threshold P-value. However, only isorhamnetin and apigenin remained significant after correcting for multiple testing (HR < 0.92; P-value $< 2 \times 10^{-4}$), in line with [29, 30]. P-values are associated with two-sided Wald tests.

Exposure	Effect size	SE(effect size)	Hazard ratio	95% CI	P-value of PH	VIF	P-value	FDR
Isorhamnetin	-0.09	0.02	0.91	(0.87, 0.95)	0.32	1.27	1.59E-05	6.11E-03
Apigenin	-0.08	0.02	0.92	(0.89, 0.96)	0.81	1.15	1.79E-04	2.02E-02
Total flavones	-0.06	0.02	0.94	(0.90, 0.98)	0.63	1.23	3.81E-03	9.63E-02
Total isoflavones	-0.06	0.02	0.95	(0.91, 0.99)	0.58	1.14	8.57E-03	1.38E-01
Quercetin	-0.05	0.02	0.95	(0.91, 0.99)	0.68	1.27	1.22E-02	1.69E-01
Hesperetin	-0.05	0.02	0.95	(0.92, 0.99)	0.62	1.13	1.31E-02	1.75E-01
Proanthocyanidin, 7-10mers	-0.05	0.02	0.95	(0.91, 0.99)	0.97	1.28	2.00E-02	2.15E-01
Total flavanones	-0.05	0.02	0.95	(0.92, 0.99)	0.97	1.14	2.15E-02	2.18E-01
Total flavonols	-0.05	0.02	0.95	(0.91, 0.99)	0.68	1.22	2.51E-02	2.20E-01
Genistein	-0.05	0.02	0.95	(0.91, 0.99)	0.4	1.1	2.59E-02	2.23E-01
Proanthocyanidin, 4-6mers	-0.05	0.02	0.95	(0.91, 0.99)	0.84	1.34	2.84E-02	2.31E-01
Naringenin	-0.04	0.02	0.96	(0.92, 1.00)	0.5	1.12	3.32E-02	2.49E-01
Malvidin	-0.04	0.02	0.96	(0.92, 1.00)	0.18	1.12	3.48E-02	2.54E-01
Proanthocyanidin, polymers	-0.05	0.02	0.96	(0.92, 1.00)	0.62	1.27	3.48E-02	2.54E-01
Glycitein	-0.05	0.02	0.96	(0.91, 1.00)	0.08	1.05	4.19E-02	2.73E-01
Isoflavones (no biochanin A or formononetin)	-0.04	0.02	0.96	(0.92, 1.00)	0.54	1.11	4.65E-02	2.79E-01
Luteolin	-0.04	0.02	0.96	(0.92, 1.00)	0.83	1.2	4.85E-02	2.81E-01
Daidzein	-0.04	0.02	0.96	(0.92, 1.00)	0.41	1.07	5.91E-02	3.03E-01
Proanthocyanidin, dimers	-0.04	0.02	0.96	(0.92, 1.00)	0.89	1.24	7.43E-02	3.29E-01
Proanthocyanidin, trimers	-0.04	0.02	0.96	(0.92, 1.01)	0.72	1.44	1.17E-01	3.99E-01
Proanthocyanidin, monomers	-0.03	0.02	0.97	(0.93, 1.01)	0.89	1.12	1.56E-01	4.37E-01
Total anthocyanidins	-0.03	0.02	0.97	(0.93, 1.01)	0.51	1.17	1.72E-01	4.51E-01
Peonidin	-0.03	0.02	0.97	(0.94, 1.01)	0.26	1.11	1.95E-01	4.78E-01
Myricetin	-0.02	0.02	0.98	(0.94, 1.02)	0.67	1.1	2.36E-01	5.32E-01
Formononetin	-0.02	0.02	0.98	(0.94, 1.02)	0.73	1.12	2.50E-01	5.45E-01
Thearubigins	-0.02	0.02	0.98	(0.94, 1.02)	0.88	1.03	2.53E-01	5.46E-01
Pelargonidin	-0.02	0.02	0.98	(0.94, 1.02)	0.66	1.09	2.72E-01	5.61E-01
Petunidin	-0.02	0.02	0.98	(0.94, 1.02)	0.48	1.1	2.76E-01	5.65E-01
Lutein and zeaxanthin	-0.02	0.02	0.98	(0.94, 1.02)	0.05	1.23	2.95E-01	5.79E-01
Total flavonoids	-0.02	0.02	0.98	(0.94, 1.02)	0.97	1.16	3.10E-01	5.87E-01
Delphinidin	-0.02	0.02	0.98	(0.94, 1.02)	0.49	1.1	3.32E-01	5.95E-01
Kaempferol	-0.02	0.02	0.98	(0.94, 1.02)	0.28	1.08	3.33E-01	5.95E-01
Eriodictyol	-0.02	0.02	0.98	(0.95, 1.02)	0.69	1.09	3.37E-01	5.95E-01
Catechin	-0.02	0.02	0.98	(0.94, 1.02)	0.86	1.2	3.63E-01	6.11E-01
Total theaflavins and polymers proanthocyanidis	-0.02	0.02	0.98	(0.94, 1.02)	0.82	1.12	3.93E-01	6.35E-01
Flavonoids (no proanthocyanidins)	-0.02	0.02	0.98	(0.95, 1.02)	0.9	1.09	4.05E-01	6.42E-01
Epigallocatechin	-0.01	0.02	0.99	(0.95, 1.02)	0.95	1.04	4.46E-01	6.81E-01
Biochanin A	-0.01	0.02	0.99	(0.95, 1.03)	0.74	1.13	5.03E-01	7.22E-01
Epicatechin 3-gallate	-0.01	0.02	0.99	(0.95, 1.03)	0.98	1.03	5.20E-01	7.24E-01
Total flavan-3-ols	-0.01	0.02	0.99	(0.95, 1.03)	0.83	1.08	5.20E-01	7.24E-01
Gallocatechin	-0.01	0.02	0.99	(0.95, 1.03)	0.98	1.02	6.29E-01	7.78E-01
Cyanidin	-0.01	0.02	0.99	(0.95, 1.03)	0.41	1.21	6.80E-01	8.10E-01
Theaflavin	-0.01	0.02	0.99	(0.95, 1.03)	0.98	1.02	6.80E-01	8.10E-01
Epicatechin	0	0.02	1	(0.96, 1.04)	0.89	1.19	8.93E-01	9.40E-01

2 Supplementary Figures

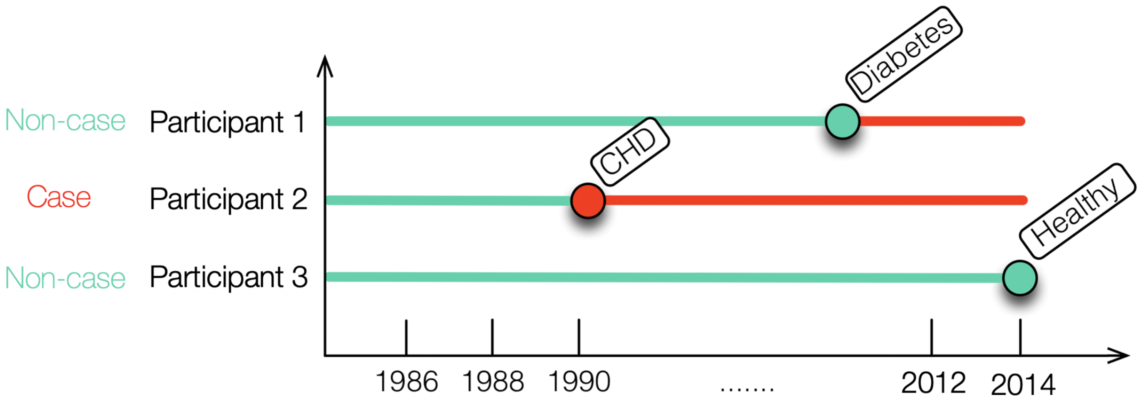

Supplementary Figure 1: **Participant Selection.** All participants were healthy in 1986. Participant 1 was healthy up to 2008 and then developed Diabetes. Her dietary data used in the analysis from 1986 to 2008 (non-case). Participant 2 developed CHD in 1990. Her dietary data was used in the analysis from 1986 to 1990 (case). Participant 3 did not develop any disease up to 2014 (non-case).

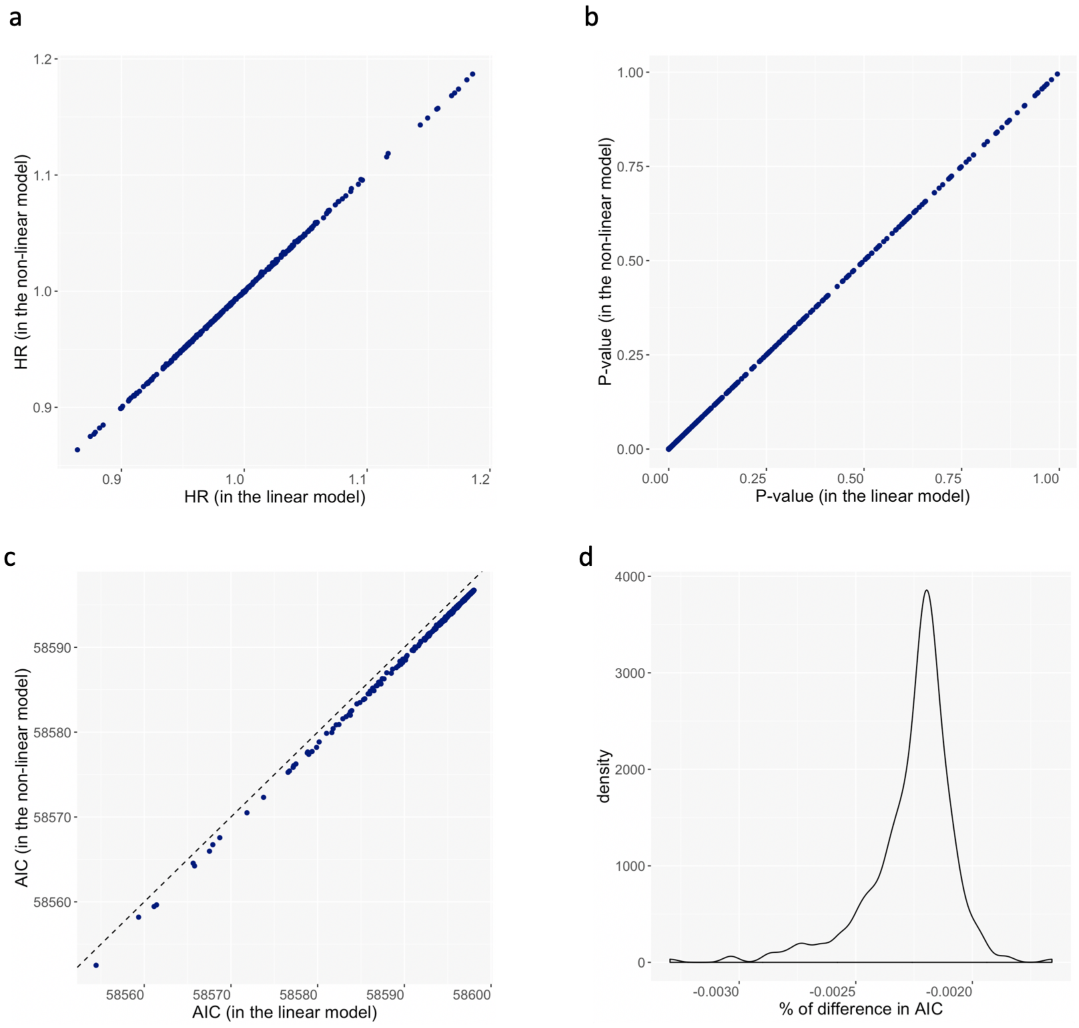

Supplementary Figure 2: Comparing the linear model with the non-linear model where a spline of degree four for age is included. In Supplementary Figure 2–a and 2–b, we compared the hazard ratios and P-values from the linear model with the non-linear model where we included a spline of degree four for age. In both cases, we see that they define a straight line of unit slope, implying that two models are almost identical in terms of the estimated hazard ratios and P-values. In Supplementary Figure 2–c, we plotted the AIC obtained from each version of the model. The AIC from non-linear models is only slightly smaller than the AIC of linear models. In Supplementary Figure 2–d, we report the distribution of the percentage of the difference between AIC in the two modeling frameworks. We found that that the average of the percentage of the difference in AIC is -0.00225, with a very small variance. Therefore, the change in AIC is not sufficiently large to choose the non-linear model over the linear model, and add to the complexity of the analysis, which results in a significantly higher run-time for each analysis and each iteration of the permutation process. Source data are provided in Source Data - Supplementary Figure 2.xlsx.

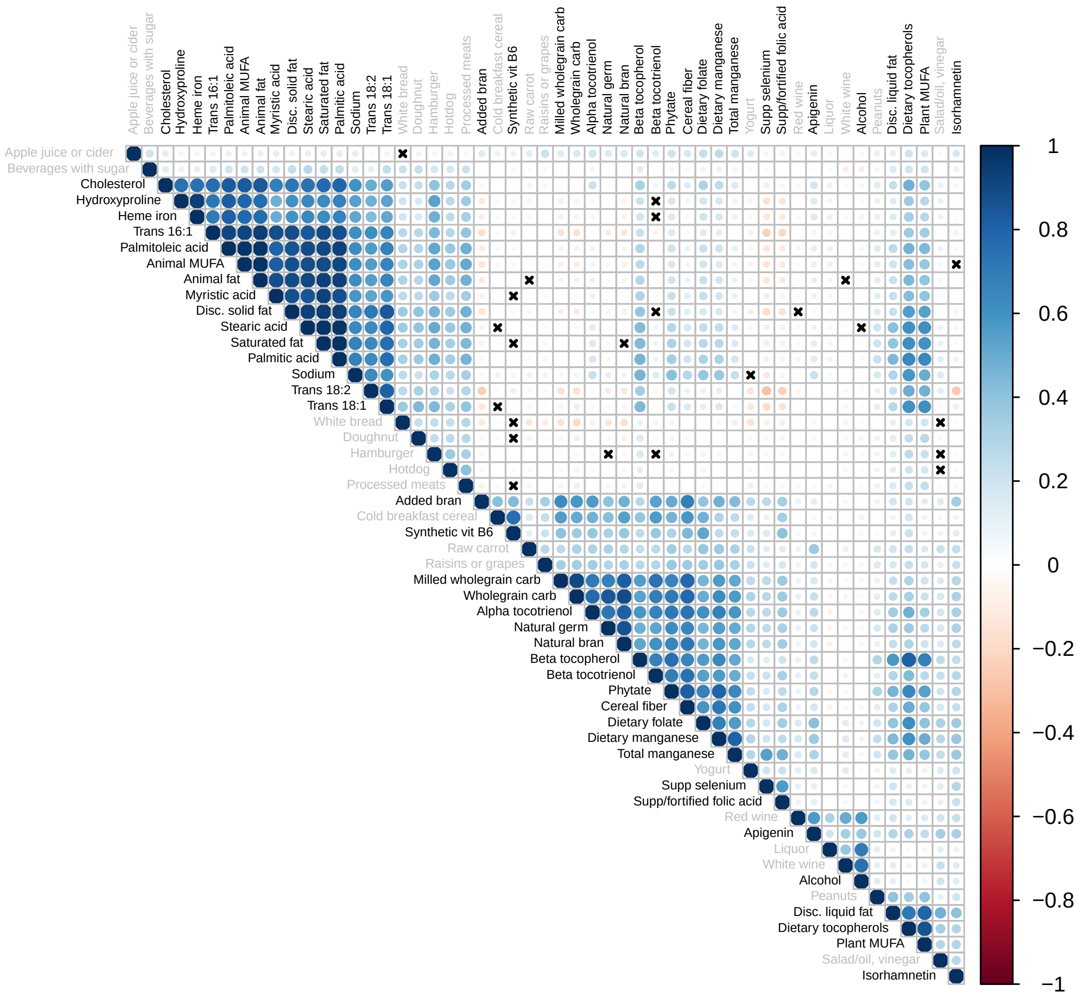

Supplementary Figure 3: **Correlation Matrix.** We looked at the Spearman correlation between 53 significant associations found in NHS. We calculated 1,378 pairwise correlations. Similar to [27], we used a permutation-based approach to estimate the two-sided P-value of significance for each pair of correlations. Given a pair of X and Y, we shuffled values of X and computed the correlation with Y, repeating this procedure for all pairs. The P-value for a correlation was the fraction of correlations from the permuted dataset with greater absolute value (equation 1). Next, we control for multiple testing using the Benjamini-Hochberg step-down approach.

$$P - value_{x,y} = \sum_{i=1}^{1378} I(|\rho_{x,y}| < |\widetilde{\rho}_i|)/1378 \tag{1}$$

We show the resulting correlation in a correlation matrix, where non-significant correlations are indicated with a cross. Nutrients are color-coded with black labels and food items are color-coded with gray. Source data are provided in Source Data - Supplementary Figure 3.xlsx.

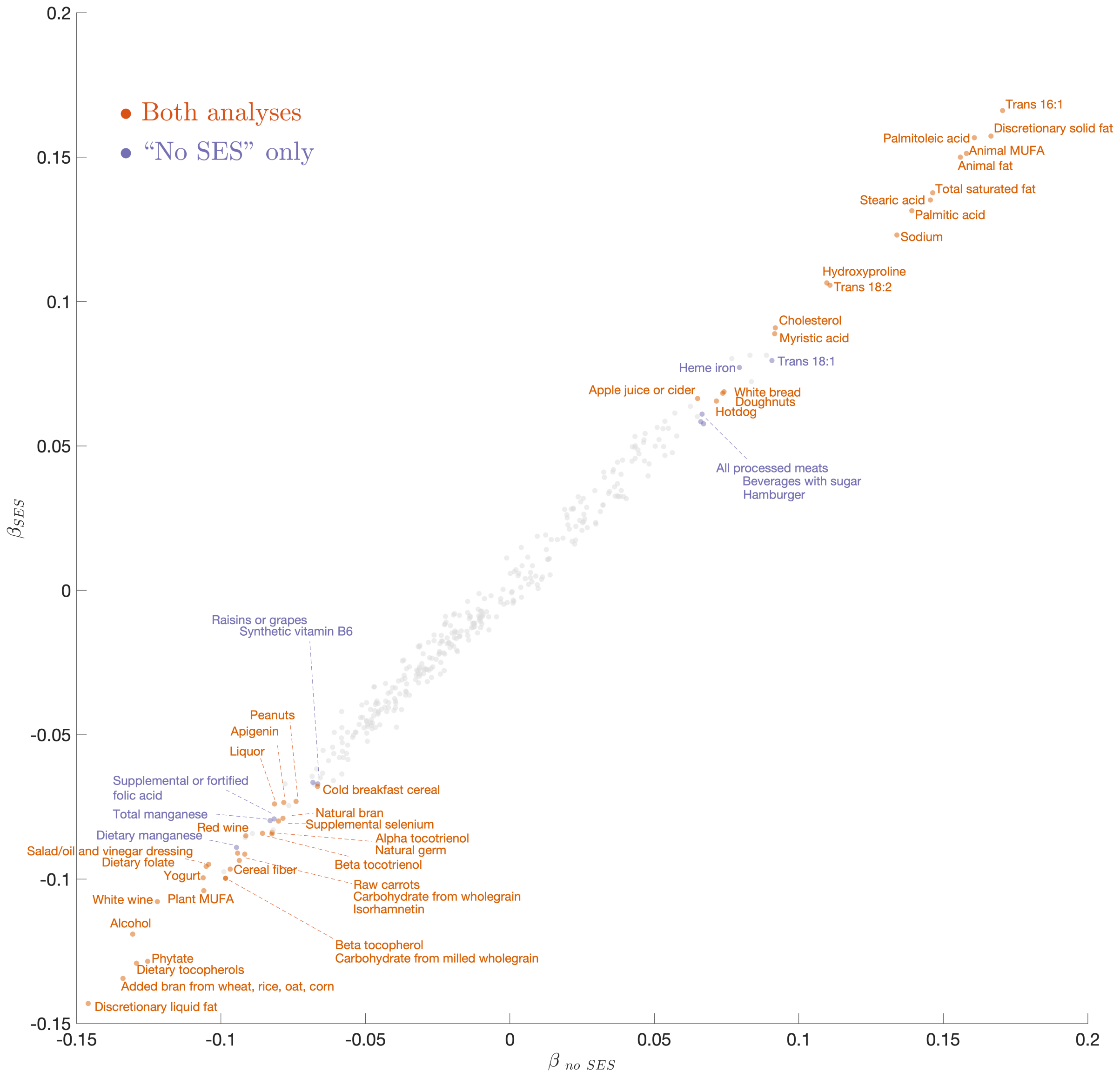

Supplementary Figure 4: **Relation between the original coefficients ($\beta_{no\ SES}$) and those estimated by including census tract family median income (β_{SES}).** The study population in NHS comprises all registered nurses with a relatively high homogeneity in educational attainment and socioeconomic status. Nevertheless, we investigated the role of SES by including census tract family median income in our analysis [31, 32]. Given the zip-code of each individual in time we were able to associate the median income of families living in that area [33]. The results are consistent with our previous analysis, confirming 43 out of 53 significant exposures originally found without SES, with only the marginal associations being affected by the additional correction. We show the relationship between the original coefficients ($\beta_{no\ SES}$) and those estimated considering SES (β_{SES}). The observed linearity proves that the two analyses are consistent over the whole panel of nutrients and foods. Indeed, the exposures are ranked in a similar fashion according to the estimated p-value, with a Spearman correlation coefficient equal to 0.9733. Their agreement further improves when the exposures with the strongest signals are taken into account (orange). The 10 exposures whose significance is affected by SES (purple) were among the weakest of the previous analysis ($\beta_{no\ SES} \to 0$). Despite the marginal effect on the significance of dietary exposures, SES strongly influences CHD outcomes, with an estimated hazard ratio across the 347 exposures in NHS1 fluctuating in the narrow range, 0.9035 ± 0.0017 (see Supplementary Figure 5). Source data are provided in Source Data - Supplementary Figure 4.xlsx.

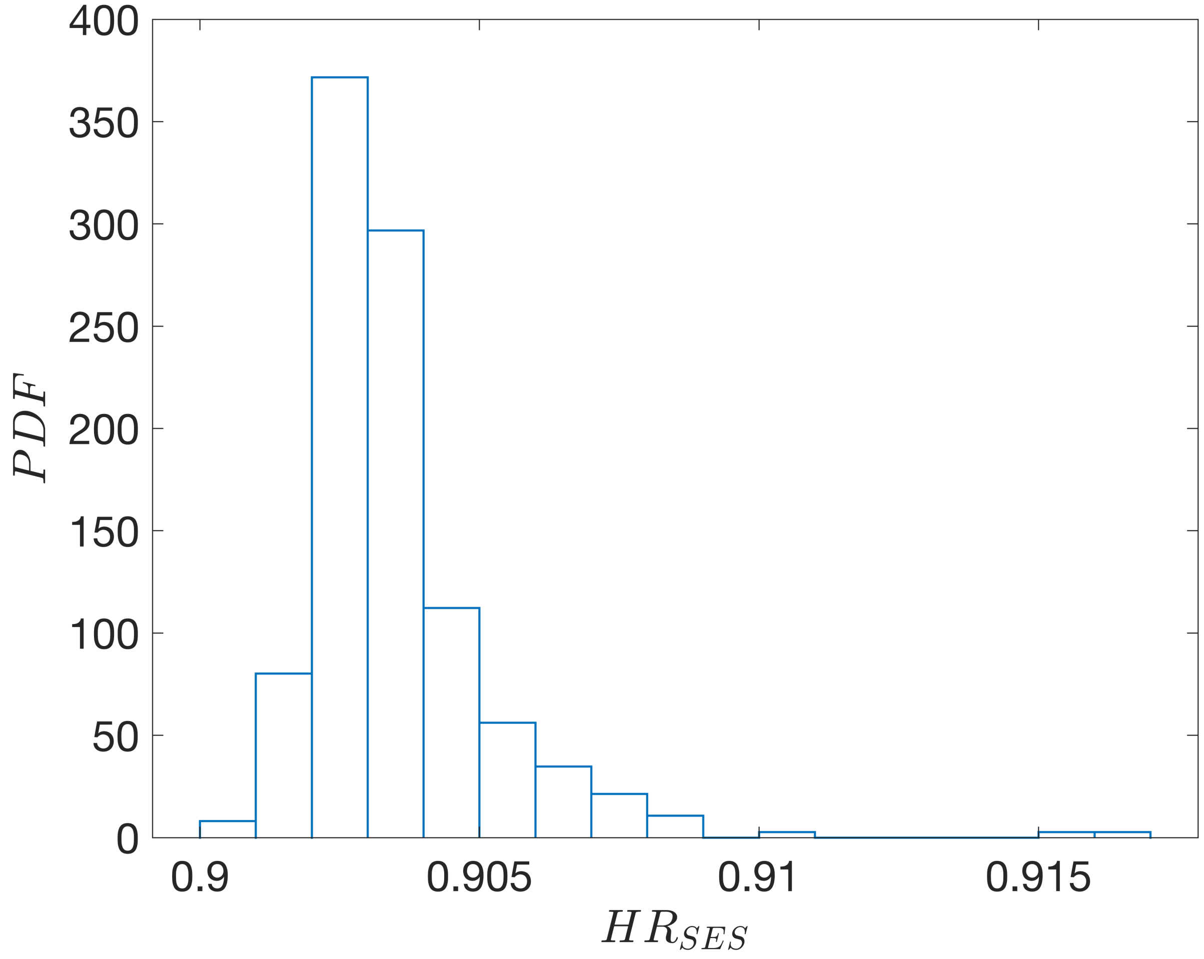

Supplementary Figure 5: **Estimation of the hazard ratio (HR_{SES}) associated with the census tract family median income.** For each exposure in NHS I we collected HR_{SES}. SES is negatively associated with CHD risk, minimally varying across the 347 exposures. Source data are provided in Source Data - Supplementary Figure 5.xlsx.

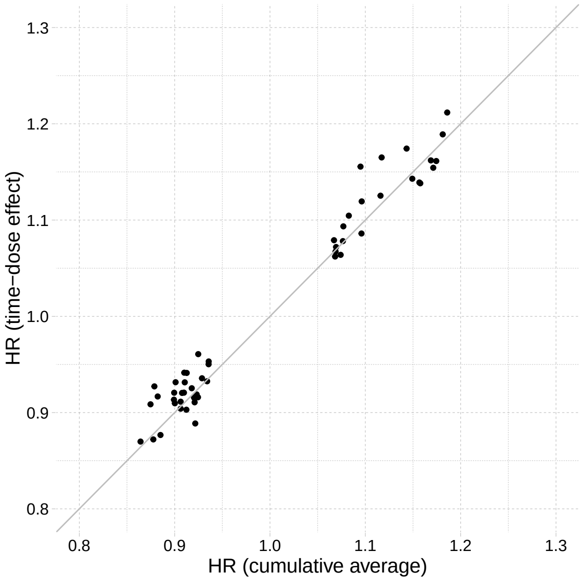

Supplementary Figure 6: **The estimated Hazard Ratio using cumulative averages versus time-dose effects.** We implemented another approach to explore the associations between dietary factors and CHD risk. Instead of using the cumulative average of the food intakes from baseline to the start of each 2-year follow-up interval (which represents the long-term usual intake and reduces the random within-person variation), we investigated the time-dose effect of the dietary exposures, the so-called "latest-diet". We show the results of our analysis on the time-dose effect in Supplementary Table 4. After using the permutation procedure to account for multiple testing, we found 29 significant associations, including 6 food items and 23 nutrients. There are 28 common exposures among the original analysis and the time-dose effect analysis. In the latter, the PH assumption is violated for three exposures (the p-value for the PH assumption test is less than 0.05). The hazard ratios (HRs) obtained using the cumulative averages is consistent with HRs calculated with time-dose effect (Spearman Correlation 0.65), and the directionality for exposures that have been already found statistically significant in our original analysis is conserved. Overall, the time-dose effect analysis yielded weaker associations between dietary exposures and CHD risk. Source data are provided in Source Data - Supplementary Figure 6.xlsx.

3 Supplementary Methods

3.1 Participant Selection

From 121,527 participants in NHS, we excluded 47,949 from the analysis who dropped out from the study before 1986, whose reported average energy intake was less than 600 or more than 3,500 kcal/day, who left more than 70 questions in the FFQ unanswered, or whose demographic data at baseline were missing. Moreover, 3,652 participants with a history of cardiovascular disease (CVD), 2,255 with diabetes mellitus, and 4,860 with cancer in the baseline year were excluded from the analysis. During the follow-up period, 14,832 individuals developed cancer, 8,111 developed diabetes mellitus, and 8,573 developed other cardiovascular diseases such as stroke (overall 25,965 participants) for whom we stopped updating the dietary records. The average number of follow-up years for non-case and case individuals were 26 and 16 years, respectively.

In NHS II, from 116,429 participants, 21,181 were excluded if their reported average energy intake was less than 600 or more than 3,500 kcal/day, they left more than 70 questions in the FFQ unanswered, or their demographic data at baseline were missing. In addition, 903 participants with a history of cardiovascular disease (CVD), 922 with diabetes mellitus, and 2,562 with cancer in the baseline year were excluded. During the follow-up period, 15,734 individuals developed cancer, 6,518 developed diabetes mellitus, and 3,318 developed other cardiovascular diseases such as stroke (overall 23,250 participants) for whom we stopped updating the dietary records. The average number of follow-up years for non-case and case individuals were 22 and 14 years, respectively.

Our ascertainment of CHD follows the guidelines offered by WHO and has been applied in other cohorts as well, such as such as the Women's Health Initiative (WHI) Study [1], the Atherosclerosis Risk in Communities (ARIC) Study [2], and PREDIMED [3]. While our definition is one of the most used in the literature, other less stringent criteria were used in other cohorts, such as EPIC [4], Jackson Heart and Framingham Offspring Cohort Studies [5].

3.2 Cumulative Average of Dietary Exposures

The use of cumulative average measurements takes advantage of all prior data and helps reduce random within-person variation, and thus offers a statistically more powerful test of an association of cumulative exposure [6]. The use of the cumulative average model to examine the association between disease incidence and repeated measurements of exposures in cohort studies can be dated back to the 1960s [7], and has been used by many researchers [8–13].

It has also been shown that analyses using cumulative averages to reflect the dietary records yield stronger associations than those using either only baseline diet or most recent diet [14]. In [14], a study analyzing the association between dietary fat and CHD, Hu, et al., argued that observing stronger associations might be due to the fact that the use of the cumulative averages reduces measurement error from intraindividual variations over time. It is also possible that the cumulative averages, which reflect long-term diet, are more relevant etiologically than either the most remote (baseline) or the most recent diets.

3.3 Multiple Testing

Since in EWAS we are conducting multiple tests, we need to control the number of false positives to extract the truly significant associations. In the GWAS setting, Bonferroni correction is utilized which controls for Type I errors, guaranteeing the family-wide error rate. The Bonferroni corrected p-value is computed by dividing the original significance threshold α by the number of tests conducted on the dependent variable. The corrected p-value will be used to reject a null hypothesis. This method assumes all tests are independent of each other which is not always the case. Having correlated tests, the Bonferroni threshold would be too conservative and results in high rate of false negatives, in turn, losing the power of detection [15]. To eliminate the assumption of independence among tests, one can use the number of *effective* tests in Bonferroni adjustment, usually determined by principal component analysis [16–18]. However, by applying both approaches to our results we find the same 31 tests to be statistically significant with a threshold of 0.05.

An alternative approach is to control the false discovery rate (FDR) which is the proportion of significant results that are actually false positives. FDR-controlling methods, such as Benjamini-Hochberg, are less conservative compared to FWER procedures. Therefore, they have more power of detection, at the cost of higher Type I error [19, 20]. The Benjamini-Hochberg procedure also assumes that the individual tests are independent of each other. We also utilized this procedure, finding 81 tests to be statistically significant, controlling for 0.05 FDR. Other more complicated techniques, such as [21], have been developed for controlling FDR that may be more appropriate when the independence assumption is violated.

Estimating the FDR through permutations of the dependent variable is preferred in the scenario in which the variables are correlated [22–24]. In the EWAS context that the independence assumption hardly meets, we compute an empirical estimate of the FDR derived through permutations of the phenotype multiple times, effectively creating a null distribution of test statistics. Since the estimate of the FDR utilizes the data itself, it inherently considers the

correlated structure of the data [23–25], an important quality given the dense correlation of environmental factors. The FDR is the ratio of the proportion of results that were called significant at a given level α in the null distribution and the proportion of results called significant from our real tests.

Here, we discuss the procedure we used to create a distribution of P-values. Sampling from an unbiased urn follows a central hyper-geometric distribution. In our study, since the confounding effect of adjusting variables exists, certain subjects have greater odds of developing CHD than other subjects. Sampling from such population resembles sampling from a biased urn which follows a non-central hyper-geometric distribution. In our permutation scheme, we maintain the confounding role of adjusting variables even tough the association between exposures and the disease is broken [26]. We use Cox regression to estimate the probability of the developing CHD for each subject at each time. Further, we permute the disease status according to the estimated probabilities. Assume N_0 is the number of non-case subjects and N_1 is the number of case subjects. D_j is a binary variable denoting the disease status of subject j. $\hat{\theta}_j$ indicates the estimated CHD odd for subject j. For permutation k,

$$r_k = (r_{k1}, r_{k2}, ..., r_{kN})^T \tag{2}$$

r_k follows a multivariate hyper-geometric distribution. We can sample r_k by using Fisher's non-central hyper-geometric distribution with non-centrality parameter $\hat{\theta} = (\hat{\theta}_1, \hat{\theta}_2, ..., \hat{\theta}_N)$. While such sampling is computationally expensive, we simplify the sampling process. We generate a uniform random number and compare it with a spectrum of cumulative CHD odds and assign CHD to each subject accordingly. We continue generating random numbers until N_1 subjects are picked. Next, we re-run the analysis and collect null P-values 1000 times.

Assuming that the number of tests is i and the number of permutations is j, the raw FDR is the ratio of raw number of results that exceed that p-value threshold in the permuted data and the number of results that exceed that p-value among real p-values.

$pval_{real}$ (sorted)	$pval_{permuted}$ 1	2	3	....	j	FDR_{raw}	FDR
P_1	$p_{1,1}$	$p_{1,2}$	$p_{1,3}$	....	$p_{1,j}$	r_1	f_1
P_2						r_2	f_2
⋮	⋮	⋱				⋮	⋮
P_i	$p_{i,1}$			....	$p_{i,j}$	r_i	f_i

FDR Calculation

The raw FDR is calculated using equation (3).

$$r_{i'} = \frac{[I(p_{i',1} < P_i') + ... + I(p_{i',j} < P_i')]/j}{I(P_1 < P_i') + ... + I(P_i < P_i')} \tag{3}$$

As FDR should be a monotonically increasing function of p-value, we ensure that FDR for a p-value is the minimum of the FDR's for all p-values equal or greater than that p-value:

$$f_i' = min\{r_{i'}, r_{i'+1}, ..., r_i\} \tag{4}$$

The null hypothesis for test i' will be rejected if f_i' is smaller than an FDR threshold.

Supplementary References

[1] Mossavar-Rahmani, Y. *et al.* Artificially sweetened beverages and stroke, coronary heart disease, and all-cause mortality in the Women's Health Initiative. *Stroke* **50**, 555–562 (2019).

[2] Bidulescu, A., Chambless, L. E., Siega-Riz, A. M., Zeisel, S. H. & Heiss, G. Usual choline and betaine dietary intake and incident coronary heart disease: the Atherosclerosis Risk in Communities (ARIC) study. *BMC Cardiovascular Disorders* **7**, 20 (2007).

[3] Garcia-Arellano, A. *et al.* Dietary inflammatory index and incidence of cardiovascular disease in the PREDIMED study. *Nutrients* **7**, 4124–4138 (2015).

[4] Guallar-Castillón, P. *et al.* Consumption of fried foods and risk of coronary heart disease: Spanish cohort of the European Prospective Investigation into Cancer and Nutrition study. *BMJ* **344**, e363 (2012).

[5] Joshi, P. H. *et al.* Remnant lipoprotein cholesterol and incident coronary heart disease: the Jackson heart and Framingham offspring cohort studies. *Journal of the American Heart Association* **5**, e002765 (2016).

[6] Willett, W. *Nutritional Epidemiology* (Oxford University Press, 2012).

[7] Kahn, H. A. & Dawber, T. R. The development of coronary heart disease in relation to sequential biennial measures of cholesterol in the Framingham study. *Journal of Clinical Epidemiology* **19**, 611–620 (1966).

[8] Larsson, S. C., Giovannucci, E. & Wolk, A. Coffee consumption and stomach cancer risk in a cohort of Swedish women. *International Journal of Cancer* **119**, 2186–2189 (2006).

[9] Kim, E. H. *et al.* Dietary fat and risk of postmenopausal breast cancer in a 20-year follow-up. *American Journal of Epidemiology* **164**, 990–997 (2006).

[10] Haring, B. *et al.* Dietary protein intake and coronary heart disease in a large community based cohort: results from the Atherosclerosis Risk in Communities (ARIC) Study. *PLoS One* **9** (2014).

[11] Halton, T. L. *et al.* Low-carbohydrate-diet score and the risk of coronary heart disease in women. *New England Journal of Medicine* **355**, 1991–2002 (2006).

[12] Streppel, M. T., Ocké, M. C., Boshuizen, H. C., Kok, F. J. & Kromhout, D. Dietary fiber intake in relation to coronary heart disease and all-cause mortality over 40 y: the Zutphen Study. *The American Journal of Clinical Nutrition* **88**, 1119–1125 (2008).

[13] Eshak, E. S. *et al.* Soft drink intake in relation to incident ischemic heart disease, stroke, and stroke subtypes in Japanese men and women: the Japan Public Health Centre–based study cohort I. *The American Journal of Clinical Nutrition* **96**, 1390–1397 (2012).

[14] Hu, F. B. *et al.* Dietary fat and coronary heart disease: a comparison of approaches for adjusting for total energy intake and modeling repeated dietary measurements. *American Journal of Epidemiology* **149**, 531–540 (1999).

[15] Hochberg, Y. A sharper bonferroni procedure for multiple tests of significance. *Biometrika* **75**, 800–802 (1988).

[16] Gao, X., Starmer, J. & Martin, E. R. A multiple testing correction method for genetic association studies using correlated single nucleotide polymorphisms. *Genetic Epidemiology* **32**, 361–369 (2008).

[17] Cheverud, J. M. A simple correction for multiple comparisons in interval mapping genome scans. *Heredity* **87**, 52–58 (2001).

[18] Ge, T., Chen, C. Y., Neale, B. M., Sabuncu, M. R. & Smoller, J. W. Phenome-wide heritability analysis of the UK Biobank. *PLoS Genetics* **13**, 1–21 (2017).

[19] Benjamini, Y. & Hochberg, Y. Controlling the false discovery rate: a practical and powerful approach to multiple testing. *Journal of the Royal Statistical Society. Series B (Methodological)* 289–300 (1995).

[20] Benjamini, Y. & Hochberg, Y. On the adaptive control of the false discovery rate in multiple testing with independent statistics. *Journal of Educational and Behavioral Statistics* **25**, 60–83 (2000).

[21] Reiner, A., Yekutieli, D. & Benjamini, Y. Identifying differentially expressed genes using false discovery rate controlling procedures. *Bioinformatics* **19**, 368–375 (2003).

[22] Manly, B. F. *Randomization, bootstrap and Monte Carlo methods in biology*, vol. 70 (CRC press, 2006).

[23] Efron, B. *Large-scale inference: empirical Bayes methods for estimation, testing, and prediction*, vol. 1 (Cambridge University Press, 2012).

[24] Westfall, P. H. & Young, S. S. *Resampling-based multiple testing: Examples and methods for p-value adjustment*, vol. 279 (John Wiley & Sons, 1993).

[25] Louis, G. M. B., Smarr, M. M. & Patel, C. J. The exposome research paradigm: an opportunity to understand the environmental basis for human health and disease. *Current Environmental Health Reports* **4**, 89–98 (2017).

[26] Epstein, M. P. *et al.* A permutation procedure to correct for confounders in case-control studies, including tests of rare variation. *The American Journal of Human Genetics* **91**, 215–223 (2012).

[27] Patel, C. J. & Manrai, A. K. Development of exposome correlation globes to map out environment-wide associations. In *Pacific Symposium on Biocomputing Co-Chairs*, 231–242 (World Scientific, 2014).

[28] Tresserra-Rimbau, A., Lamuela-Raventos, R. M. & Moreno, J. J. Polyphenols, food and pharma. current knowledge and directions for future research. *Biochemical Pharmacology* **156**, 186–195 (2018).

[29] Gao, L. *et al.* Isorhamnetin protects against cardiac hypertrophy through blocking PI3K–AKT pathway. *Molecular & Cellular Biochemistry* **429**, 167–177 (2017).

[30] Zhang, K., Song, W., Li, D. & Jin, X. Apigenin in the regulation of cholesterol metabolism and protection of blood vessels. *Experimental & Therapeutic Medicine* **13**, 1719–1724 (2017).

[31] Hu, Y. *et al.* Mediterranean diet and incidence of rheumatoid arthritis in women. *Arthritis Care and Research* **67**, 597–606 (2015).

[32] Pun, V. C. *et al.* Prospective study of ambient particulate matter exposure and risk of pulmonary embolism in the nurses' health study cohort. *Environmental Health Perspectives* **123**, 1265–1270 (2015).

[33] U.S.-Census-Bureau. 2000 Census of Population and Housing File 3. Tech. Rep. 2002.
