# Supplementary material for: A systematic comprehensive longitudinal evaluation of dietary factors associated with acute myocardial infarction and fatal coronary heart disease
Source: Nat Commun. 2020 Nov 27;11:6074. doi: 10.1038/s41467-020-19888-2 (PMC7699643; doi:10.1038/s41467-020-19888-2)
Supplement: Supplementary file 3 — Reporting Summary [file 41467_2020_19888_MOESM3_ESM.pdf]

nature research

Corresponding author(s): Albert-Laszlo Barabasi

Last updated by author(s): Oct 21, 2020

Reporting Summary

Nature Research wishes to improve the reproducibility of the work that we publish. This form provides structure for consistency and transparency in reporting. For further information on Nature Research policies, see our Editorial Policies and the Editorial Policy Checklist.

Statistics

For all statistical analyses, confirm that the following items are present in the figure legend, table legend, main text, or Methods section.

n/a	Confirmed	
☐	☒	The exact sample size (n) for each experimental group/condition, given as a discrete number and unit of measurement
☐	☒	A statement on whether measurements were taken from distinct samples or whether the same sample was measured repeatedly
☐	☒	The statistical test(s) used AND whether they are one- or two-sided<br>*Only common tests should be described solely by name; describe more complex techniques in the Methods section.*
☐	☒	A description of all covariates tested
☐	☒	A description of any assumptions or corrections, such as tests of normality and adjustment for multiple comparisons
☐	☒	A full description of the statistical parameters including central tendency (e.g. means) or other basic estimates (e.g. regression coefficient) AND variation (e.g. standard deviation) or associated estimates of uncertainty (e.g. confidence intervals)
☐	☒	For null hypothesis testing, the test statistic (e.g. F, t, r) with confidence intervals, effect sizes, degrees of freedom and P value noted<br>*Give P values as exact values whenever suitable.*
☒	☐	For Bayesian analysis, information on the choice of priors and Markov chain Monte Carlo settings
☒	☐	For hierarchical and complex designs, identification of the appropriate level for tests and full reporting of outcomes
☐	☒	Estimates of effect sizes (e.g. Cohen's d, Pearson's r), indicating how they were calculated

Our web collection on statistics for biologists contains articles on many of the points above.

Software and code

Policy information about availability of computer code

Data collection	Demographic and dietary data were collected using dietary questionnaires, designed internally by Nurses' Health Study (NHS). Disease-related data were collected using medical questionnaires. The ascertainment of a disease was done by physicians, reviewing the medical records. Death-related data were identified from state vital statistics records and the National Death Index, or were reported by the families and the postal system. Detailed information are provided here: www.hsph.harvard.edu/nutritionsource/nurses-health-study.
Data analysis	Statistical analyses were performed using R version 3.4.0., Python version 2.7.16 and MATLAB 2019a. The programming materials are available on GitHub platform (https://github.com/soodimilanlouei/EWAS-NHS).

For manuscripts utilizing custom algorithms or software that are central to the research but not yet described in published literature, software must be made available to editors and reviewers. We strongly encourage code deposition in a community repository (e.g. GitHub). See the Nature Research guidelines for submitting code & software for further information.

Data

Policy information about availability of data

All manuscripts must include a data availability statement. This statement should provide the following information, where applicable:

- Accession codes, unique identifiers, or web links for publicly available datasets
- A list of figures that have associated raw data
- A description of any restrictions on data availability

The authors declare that all data supporting the findings of this study are available upon request to Nurses' Health Study and when the request for data access is approved. Access is restricted due to participant confidentiality and privacy concerns. Further information including the procedures to obtain and access data from the Nurses' Health Studies is described at https://www.nurseshealthstudy.org/researchers (contact email: nhsaccess@channing.harvard.edu). The Food Frequency Questionnaires used in NHS are available in this link (https://www.nurseshealthstudy.org/participants/questionnaires). Harvard University Food Composition

Database can be accessed in this link (https://regepi.bwh.harvard.edu/health/nutrition.html). The source data underlying Figures 1, 3-a, 3-b, and 4-b, and Supplementary Figures 2-6 are provided as a Source Data file.

Field-specific reporting

Please select the one below that is the best fit for your research. If you are not sure, read the appropriate sections before making your selection.

☒ Life sciences ☐ Behavioural & social sciences ☐ Ecological, evolutionary & environmental sciences

For a reference copy of the document with all sections, see nature.com/documents/nr-reporting-summary-flat.pdf

Life sciences study design

All studies must disclose on these points even when the disclosure is negative.

Sample size	Sample size was determined to include all plausible participants in the cohort.
Data exclusions	In the baseline year, participants with a history of cardiovascular disease (CVD), diabetes mellitus, and cancer were excluded. We also excluded participants whose demographic data were missing, whose reported average energy intake was less than 600 or more than 3,500 kcal/day, or left more than 70 questions in the FFQ unanswered. These exclusion criteria were chosen by virtue of minimizing reverse causation bias and reducing the impact of measurement errors and missing data. The exclusion criteria are well-established in the nutritional epidemiology and appropriate references are mentioned in the main manuscript.
Replication	The majority of findings were replicated in NHS II and the remaining findings were discussed in the context of available literature. Rather than validation through experimentation, we followed an approach similar to (Patel et al., PLoS one, 2010).
Randomization	Our analysis was performed on Nurses' Health Study data which is among the largest prospective investigations into the risk factors for major chronic diseases. There are several small randomized studies within NHS focusing on a handful dietary factors. Since we aimed to investigate the effect of a comprehensive list of dietary factors on Coronary Heart Disease, those studies were not suitable for our purpose.
Blinding	Similar to the previous point, our analysis is done on a huge body of dataset that has been collected prospectively in Nurses' Health Study, and within this cohort, a blinded sub-study to examine the effect of a comprehensive list of dietary factors on Coronary Heart Disease was not available.

Reporting for specific materials, systems and methods

We require information from authors about some types of materials, experimental systems and methods used in many studies. Here, indicate whether each material, system or method listed is relevant to your study. If you are not sure if a list item applies to your research, read the appropriate section before selecting a response.

Materials & experimental systems

n/a	Involved in the study	
☒	☐	Antibodies
☒	☐	Eukaryotic cell lines
☒	☐	Palaeontology and archaeology
☒	☐	Animals and other organisms
☐	☒	Human research participants
☒	☐	Clinical data
☒	☐	Dual use research of concern

Methods

n/a	Involved in the study	
☒	☐	ChIP-seq
☒	☐	Flow cytometry
☒	☐	MRI-based neuroimaging

Human research participants

Policy information about studies involving human research participants

Population characteristics	The research participants were registered women nurses, from across the U.S., with average age 53 at baseline year. At baseline, all participants were healthy and free of chronic diseases.
Recruitment	Nurses' Health Study gathered registered nurses ages 30-55 years from across the U.S. to respond to a series of questionnaires. Nurses were specifically chosen because of their ability to complete the health-related, often very technical, questionnaires thoroughly and accurately. Dietary data were collected using an internally designed Food Frequency Questionnaires (FFQs), with documented reproducibility and validity. However, FFQs require good memory, literacy, and numerical skills, hence, susceptible to recall bias.
Ethics oversight	The study protocol was approved by the institutional review board (IRB) of the Brigham and Women's Hospital, and the IRB allowed participants' completion of questionnaires to be considered as implied consent. Written informed consent was obtained from participants to release medical records documenting the incidence of Coronary Heart Disease.

Note that full information on the approval of the study protocol must also be provided in the manuscript.
