# Supplementary material for: A systematic comprehensive longitudinal evaluation of dietary factors associated with acute myocardial infarction and fatal coronary heart disease
Source: Nat Commun. 2020 Nov 27;11:6074. doi: 10.1038/s41467-020-19888-2 (PMC7699643; doi:10.1038/s41467-020-19888-2)
Supplement: Supplementary file 4 — Supplementary Software [file 41467_2020_19888_MOESM4_ESM.zip › EWAS-NHS-master/KG-SI/Rest/Cassidy (2016).pdf]

See corresponding editorial on page 549.

Habitual intake of anthocyanins and flavanones and risk of cardiovascular disease in men[1,2]

*Aedín Cassidy,[3] Monica Bertoia,[4,6] Stephanie Chiuve,[4–6] Alan Flint,[4] John Forman,[6] and Eric B Rimm[4–6]**

[3]Department of Nutrition and Preventive Medicine, Norwich Medical School, University of East Anglia, Norwich, United Kingdom; Departments of [4]Nutrition and [5]Epidemiology, Harvard T.H. Chan School of Public Health, Boston, MA; and [6]Channing Division of Network Medicine, Department of Medicine, Brigham and Women's Hospital, Boston, MA

ABSTRACT

Background: Although increased fruit intake reduces cardiovascular disease (CVD) risk, which fruits are most beneficial and what key constituents are responsible are unclear. Habitual intakes of flavonoids, specifically anthocyanins and flavanones, in which >90% of habitual intake is derived from fruit, are associated with decreased CVD risk in women, but associations in men are largely unknown.

Objective: We examined the relation between habitual anthocyanin and flavanone intake and coronary artery disease and stroke in the Health Professionals Follow-Up Study.

Design: We followed 43,880 healthy men who had no prior diagnosed CVD or cancer. Flavonoid intake was calculated with the use of validated food-frequency questionnaires.

Results: During 24 y of follow-up, 4046 myocardial infarction (MI) and 1572 stroke cases were confirmed by medical records. Although higher anthocyanin intake was not associated with total or fatal MI risk, after multivariate adjustment an inverse association with nonfatal MI was observed (HR: 0.87; 95% CI: 0.75, 1.00; $P = 0.04$; P-trend = 0.098); this association was stronger in normotensive participants (HR: 0.81; 95% CI: 0.69, 0.96; P-interaction = 0.03). Anthocyanin intake was not associated with stroke risk. Although flavanone intake was not associated with MI or total stroke risk, higher intake was associated with a lower risk of ischemic stroke (HR: 0.78; 95% CI: 0.62, 0.97; $P = 0.03$, P-trend = 0.059), with the greatest magnitude in participants aged ≥65 y (P-interaction = 0.04).

Conclusions: Higher intakes of fruit-based flavonoids were associated with a lower risk of nonfatal MI and ischemic stroke in men. Mechanistic studies and clinical trials are needed to unravel the differential benefits of anthocyanin- and flavanone-rich foods on cardiovascular health. *Am J Clin Nutr* 2016;104:587–94.

Keywords: flavonoids, anthocyanins, flavanones, heart disease, stroke, men

INTRODUCTION

Recent data suggest that dietary fruit intake is the third most important modifiable risk factor for reducing global rates of noncommunicable diseases after high blood pressure and smoking (1). However, individual fruits might not be equally associated with health, and which fruits are most beneficial and what key constituents are responsible for the health benefits remain to be established. Recent prospective data provide evidence that suggests beneficial effects of higher intakes of specific fruits, including blueberries and grapes, for reducing the risk of type 2 diabetes (2), and growing evidence highlights the beneficial effect of specific flavonoids from plant bioactive compounds present in fruits that improve endothelial function, blood pressure, and insulin sensitivity (3–7). Thus, flavonoids might be key constituents of fruits that decrease the risk of cardiovascular disease (CVD),[7] a major contributor to morbidity and mortality (8–10).

Although fruit contributes to the intake of several different flavonoid subclasses, in the habitual diet >90% of the intake of 2 main subclasses of flavonoids, anthocyanins and flavanones, is derived from fruit (3, 6). Supportive data for their effects on vascular function come from mechanistic studies and show that their downstream metabolites can alter signaling pathways involved in vascular inflammation, inhibit atherosclerosis development, improve endothelial function principally via improved blood flow, enhance production or reduce NAD(P)H oxidase-dependent elimination of endothelial nitric oxide, and inhibit platelet function (11–14). Relative to other flavonoid subclasses, there have been limited trials to our knowledge that have examined the impact of anthocyanins and flavanones on CVD risk factors. In several short-term interventions, anthocyanin- or flavanone-rich foods resulted in reductions in both systolic and diastolic blood pressure and favorable changes in arterial stiffness, although whether the benefits are greater in nonmedicated participants has to our knowledge not yet been examined (13,

[1] Supported by NIH research grants CA55075, HL35464, and UM1 CA167552 and the Biotechnology and Biological Sciences Research Council. This is an open access article distributed under the CC-BY license (http://creativecommons.org/licenses/by/3.0/).

[2] Supplemental Table 1 is available from the "Online Supporting Material" link in the online posting of the article and from the same link in the online table of contents at http://ajcn.nutrition.org.

*To whom correspondence should be addressed. E-mail: erimm@hsph.harvard.edu.

[7] Abbreviations used: CAD, coronary artery disease; CVD, cardiovascular disease; FFQ, food-frequency questionnaire; MI, myocardial infarction.

Received February 22, 2016. Accepted for publication June 23, 2016.

First published online August 3, 2016; doi: 10.3945/ajcn.116.133132.

15–20). Anthocyanins have been shown to exert beneficial effects on total and LDL cholesterol concentrations, with some evidence that the effect is mediated by improvements in cholesterol efflux capacity (21, 22). Recent trial evidence supports an anti-inflammatory effect of anthocyanins in patients with hypercholesterolemia (23), and recent cross-sectional data provide supporting evidence that suggests that an anti-inflammatory effect may be a key component underlying the reduction in risk associated with higher habitual anthocyanin intake (7).

With a few exceptions, most of the prospective studies that have examined associations between total and flavonoid subclasses on CVD risk have focused on women (6, 24–29). Several studies were unable to determine flavanones and anthocyanin intake accurately because they were poorly captured in earlier food compositional databases (29–31). Our focus on anthocyanins and flavanones stems from our previous studies in women, in which we observed an inverse association between the intake of flavanones and risk of ischemic stroke and among younger women higher habitual anthocyanin intake and a lower risk of nonfatal myocardial infarction (MI) (6, 26). Based on these data, we hypothesized a priori that a higher intake of anthocyanins would be associated with a reduced risk of MI in men and a higher habitual intake of flavanones would be associated with a reduced risk of ischemic stroke.

METHODS

Study population

In 1986, 51,529 men aged 32–81 y were enrolled in the Health Professionals Follow-Up Study. Each participant returned a questionnaire by mail on lifestyle and medical history and received follow-up questionnaires biennially to record newly diagnosed illnesses and to update lifestyle factors. Beginning in 1986, every 4 y participants completed semiquantitative food-frequency questionnaires (FFQs) (32, 33). Participants who reported a history of MI, stroke, angina, other CVDs or coronary bypass surgery, or cancer (except nonmelanoma skin cancer) at baseline were excluded. Participants who were missing dietary data at baseline or had implausible values for total caloric intake (<800 or >4200 kcal/d) were also excluded, resulting in the inclusion of 43,880 men in these analyses. The institutional review board at the Harvard T.H. Chan School of Public Health reviewed and approved this study, and participants provided implied consent by virtue of returning their questionnaires.

Outcome assessment

The outcomes were incident MI [which included nonfatal MI and fatal coronary artery disease (CAD)] and stroke (ischemic and hemorrhagic) that occurred after the return of the 1986 questionnaire and before January 2010. We requested permission to review the medical records of all participants who reported a physician diagnosis of a CAD or stroke during follow-up. Physicians blinded to risk factor status reviewed all relevant records to confirm all cases.

Nonfatal MI was confirmed if data in the medical records met WHO criteria based on symptoms plus either diagnostic electrocardiographic changes or elevated cardiac enzyme concentrations (34). Fatal CAD was defined as a fatal MI if confirmed by hospital records, autopsy, or if CAD was listed on the death certificate as the cause of death and evidence of previous CAD was available.

Stroke, cerebrovascular pathology caused by infection, trauma, or malignancy was excluded, and "silent" strokes discovered only by radiologic imaging were also excluded. Strokes were confirmed with the use of the National Survey of Stroke criteria (35), which requires a neurological deficit of rapid or sudden onset lasting ≥24 h or until death. Fatal strokes were identified by next of kin, postal authorities, or the National Death Index and confirmed by medical records, autopsy reports, and death certificates with stroke listed as the underlying cause. We categorized types of stroke as ischemic (embolic or thrombotic), hemorrhagic (subarachnoid or intraparenchymal), and unknown. Strokes that required hospitalization and for which confirmatory information was obtained but medical records were unavailable were designated as probable (25% of total strokes). Because the exclusion of probable strokes did not alter the results, we included both confirmed and probable strokes in this analysis.

Dietary assessment

Dietary intake data were collected from participants in 1986 and subsequently every 4 y. A database for assessment of intake of the different flavonoid subclasses was constructed as previously described (5). Briefly, intakes of individual compounds (energy-adjusted aglycone equivalents) were calculated as the sum of the consumption frequency of each food multiplied by the content of the specific flavonoid (aglycone equivalents) for the specified portion size. We derived intakes of flavanones (eriodictyol, hesperetin, naringenin) and anthocyanins (cyanidin, delphinidin, malvidin, pelargonidin, petunidin, peonidin). The validity and reproducibility of the FFQs have been reported previously. Correlations between major dietary sources of flavonoids, including anthocyanins and flavanones (fruits, vegetables, tea, wine) measured by diet records and FFQs, were 0.70, 0.50, 0.77, and 0.83, respectively (36, 37); similar correlations were observed between several urinary flavonoid biomarkers and fruit and vegetable intake (0.43–0.66) (38).

Statistical methods

Participants contributed person-time from the date of return of the 1986 questionnaire to the date of MI or stroke diagnosis, death, or the end of follow-up (January 2010). We used left-truncated Cox proportional hazards regression models with time-varying covariates, with a counting process data structure and age in years as the time scale, stratifying additionally on calendar year (39) to estimate the HR for MI risk and stroke with the use of the lowest flavonoid intake quintile as the referent group. We controlled for BMI (in kg/m^2) (<25, 25–29.9, or ≥30), physical activity (metabolic equivalents/wk in quintiles), alcohol consumption (0, 0.1–4.9, 5–14.9, 15–29.9, or ≥30 g/d), smoking (never, past, or current: 1–14 or ≥15 cigarettes/d), marital status, history of hypertension, history of hypercholesterolemia, quintiles of energy intake, cereal fiber, fat intake (SFAs, PUFAs, or *trans* fatty acids) and folate, and family history of MI. We defined subgroup analyses a priori to examine effect modification by age (participants aged <65 or ≥65 y), the presence of type 2 diabetes mellitus, and hypertension. All analyses were conducted with SAS software version 9.2 (SAS Institute). All *P* values were 2-sided.

RESULTS

During 24 y of follow-up among the 43,880 men, 4046 cases of MI (2222 nonfatal) and 1572 cases of stroke (901 ischemic stroke

TABLE 1
Baseline characteristics of men from the Health Professionals Follow-Up Study by quintile of anthocyanin intake (1986)[1]

	Anthocyanin intake, mg/d					
	Quintile 1 (n = 8779)	Quintile 2 (n = 8764)	Quintile 3 (n = 8786)	Quintile 4 (n = 8776)	Quintile 5 (n = 8775)	P-trend
Age, y	53.3 ± 9.7	53.0 ± 9.5	52.9 ± 9.5	53.2 ± 9.5	53.6 ± 9.5	—
BMI, kg/m^2	25.6 ± 3.4	25.6 ± 3.4	25.5 ± 3.4	25.4 ± 3.3	25.3 ± 3.2	<0.0001
Smoking, %						
Never	45	50	52	53	54	<0.0001
Former	41	40	40	40	41	0.38
Current	14	9	8	7	6	<0.0001
Physical activity, METs/wk	17.7 ± 27.7	19.7 ± 26.6	21.4 ± 30.6	22.4 ± 27.5	24.8 ± 34.1	<0.0001
Family history of MI, %	12	12	11	12	13	0.52
History of hypertension, %	22	20	19	20	19	0.0002
History of diabetes, %	3	2	3	2	3	0.03
History of hypercholesterolemia, %	10	10	11	11	12	<0.0001
Energy intake, kcal/d	2050 ± 656	2020 ± 595	2051 ± 697	2100 ± 568	1748 ± 506	<0.0001
Alcohol, g/d	13.3 ± 18.7	10.6 ± 14.6	11.1 ± 14.7	11.5 ± 14.4	10.5 ± 14.2	<0.0001
SFAs, g/d	26.7 ± 6.6	25.6 ± 5.9	24.6 ± 5.8	23.9 ± 5.7	22.5 ± 6.0	<0.0001
MUFAs, g/d	28.9 ± 6.2	28.3 ± 5.8	27.6 ± 5.8	26.8 ± 5.7	25.4 ± 6.0	<0.0001
PUFAs, g/d	13.2 ± 3.8	13.4 ± 3.4	13.3 ± 3.4	13.2 ± 3.4	12.9 ± 3.4	<0.0001
trans Fatty acids, g/d	3.1 ± 1.2	3.0 ± 1.1	2.8 ± 1.1	2.7 ± 1.1	2.5 ± 1.1	<0.0001
Cereal fiber, g/d	5.3 ± 4.0	5.7 ± 3.6	5.9 ± 4.3	6.0 ± 3.7	6.3 ± 4.0	<0.0001
AHEI score	42.2 ± 10.7	45.8 ± 10.5	48.2 ± 10.5	49.9 ± 10.6	51.7 ± 10.6	<0.0001
Flavanones, mg/d	42.2 ± 44.5	48.3 ± 42.7	51.6 ± 44.3	54.7 ± 43.3	62.2 ± 48.7	<0.0001

[1]Values are age-adjusted (except for age) means ± SDs, unless otherwise specified. Intakes of SFAs, MUFAs, PUFAs, *trans* fatty acids, and cereal fiber are energy-adjusted values. AHEI, Alternative Healthy Eating Index; MET, metabolic equivalent task; MI, myocardial infarction.

cases) were reported. Baseline characteristics of the participants according to quintiles of anthocyanin intake are shown in **Table 1**. The median age of cases at baseline was 53 y, with an age range of 39–77 y. Men with higher anthocyanin intake smoked less, exercised more, and had a lower intake of saturated fat, energy, and alcohol and higher cereal fiber intakes. Anthocyanin intake ranged from 0 to 613 mg/d (IQR: 3.9–15.7 mg/d) and flavanone intake from 0 to 728 mg/d (IQR: 18.8–70.9 mg/d). Participant characteristic trends were similar across quintiles of flavanones (**Supplemental Table 1**).

Although anthocyanin intake was inversely associated with MI risk (total, fatal, and nonfatal) in age-adjusted models, after

TABLE 2
Relation between flavanone and anthocyanin intake and MI in participants from the Health Professionals Follow-Up Study[1]

	Quintile 1	Quintile 2	Quintile 3	Quintile 4	Quintile 5	P-trend
Flavanones, mg/d	7.5	23.6	43.5	64.5	103.9	
Total MI cases, n/person-years	844/174,303	761/176,057	792/173,908	819/168,361	830/164,287	
Age-adjusted	1.0	0.89 (0.80, 0.98)	0.86 (0.78, 0.94)	0.86 (0.78, 0.95)	0.86 (0.78, 0.94)	0.006
Multivariable	1.0	0.96 (0.87, 1.06)	0.96 (0.87, 1.06)	0.99 (0.89, 1.09)	0.98 (0.88, 1.08)	0.87
Nonfatal MI cases, n	490	405	439	444	444	
Age-adjusted	1.0	0.82 (0.72, 0.94)	0.85 (0.75, 0.97)	0.87 (0.76, 0.99)	0.86 (0.76, 0.98)	0.15
Multivariable	1.0	0.87 (0.76, 0.99)	0.93 (0.82, 1.06)	0.98 (0.85, 1.12)	1.01 (0.88, 1.16)	0.34
Fatal MI cases, n/person-years	350/174,303	354/176,057	352/173,908	375/168,361	385/164,287	
Age-adjusted	1.0	0.98 (0.85, 1.14)	0.87 (0.75, 1.01)	0.86 (0.75, 1.00)	0.86 (0.74, 0.99)	0.02
Multivariable	1.0	1.10 (0.95, 1.28)	1.01 (0.87, 1.18)	1.01 (0.87, 1.18)	0.95 (0.81, 1.11)	0.22
Anthocyanins, mg/d	1.9	4.5	7.8	13.7	26.3	
Total MI cases, n/person-years	863/167,657	833/171,701	793/173,427	814/173,317	743/170,814	
Age-adjusted	1.0	0.96 (0.87, 1.05)	0.89 (0.81, 0.98)	0.90 (0.82, 0.99)	0.82 (0.74, 0.90)	<0.0001
Multivariable	1.0	1.04 (0.94, 1.14)	1.00 (0.90, 1.10)	1.04 (0.94, 1.14)	0.97 (0.87, 1.07)	0.44
Nonfatal MI cases, n	482	448	443	469	380	
Age-adjusted	1.0	0.92 (0.81, 1.04)	0.90 (0.79, 1.02)	0.94 (0.83, 1.07)	0.76 (0.66, 0.87)	0.0002
Multivariable	1.0	0.96 (0.84, 1.09)	0.97 (0.85, 1.10)	1.04 (0.91, 1.18)	0.87 (0.75, 1.00)	0.098
Fatal MI cases, n/person-years	379/167,657	384/171,701	349/173,427	343/173,317	361/170,814	
Age-adjusted	1.0	1.01 (0.88, 1.17)	0.89 (0.77, 1.03)	0.85 (0.73, 0.98)	0.88 (0.77, 1.02)	0.04
Multivariable	1.0	1.14 (0.99, 1.32)	1.02 (0.88, 1.19)	1.03 (0.88, 1.20)	1.10 (0.94, 1.28)	0.56

[1]Values are HRs (95% CIs) unless otherwise specified; median intakes are expressed as mg/d. The multivariable model was adjusted for age, physical activity, smoking, BMI, alcohol, energy, cereal fiber, folate, SFAs, PUFAs, *trans* fatty acids, marital status, hypertension, hypercholesterolemia, and family history of MI. MI, myocardial infarction.

additionally adjusting for lifestyle, diet, and health status the reduction in risk was only statistically significant for nonfatal MI risk (**Table 2**). After multivariate adjustment, a higher habitual intake of anthocyanins was associated with a 14% lower risk of nonfatal MI (HR: 0.87; 95% CI: 0.75, 1.00; *P*-trend = 0.098) (Table 2). We also examined the association between extreme deciles of anthocyanin intake and nonfatal MI risk [median intake: 0.97 mg/d in the bottom decile and 35.9 mg/d in the top decile; RR: 0.81 (95% CI: 0.67, 0.99)] in fully adjusted models. In stratified analyses, we found that the inverse association was stronger in participants who were not hypertensive (HR: 0.81; 95% CI: 0.69, 0.96) than participants diagnosed with hypertension (HR: 1.05; 95% CI: 0.79, 1.39; *P*-interaction = 0.03). In participants with diabetes (HR: 0.97; 95% CI: 0.65, 1.45), the inverse association between anthocyanin intake and nonfatal MI was not significantly different from men who did not have diabetes (HR: 0.85; 95% CI: 0.73, 0.99; *P*-interaction = 0.67). When we restricted our analyses to men ≤65 y in other stratified analyses, the results did not change (data not shown).

Although anthocyanin intake was not associated with a lower risk of stroke, higher flavanone intake was associated with a lower risk of ischemic stroke (HR: 0.78; 95% CI: 0.62, 0.97; *P*-trend = 0.059). This inverse association was present only in men aged >65 y (HR: 0.71; 95% CI: 0.54, 0.92) compared with men ≤65 y (HR: 1.0; 95% CI: 0.66, 1.51; *P*-interaction = 0.04). In other stratified analyses, we found that the inverse association was similar in those with and without hypertension (data not shown). Flavanone intake was not associated with nonfatal MI risk after multivariate adjustment, and we observed no associations between anthocyanin intake and fatal MI risk or hemorrhagic stroke (Tables 2 and 3). For flavanones, we observed no associations with fatal MI risk, although we observed a positive association between intake and the risk of hemorrhagic stroke (*n* = 225 cases). When we restricted follow-up to 1998 (12 y), the findings did not change (data not shown).

DISCUSSION

In this prospective cohort study of well-characterized men with 24 y of follow-up, we observed that a higher intake of anthocyanins was associated with a 14% lower risk of nonfatal MI and a higher intake of flavanones was associated with a 22% lower risk of ischemic stroke; these inverse associations were independent of established dietary and nondietary CVD risk factors. These compounds are commonly consumed through fruit intake because they are present in either red or blue fruits (anthocyanins) or citrus fruits (flavanones) and therefore readily incorporated into the habitual diet (**Table 4**) (40, 41). A simple dietary change therefore has the potential to have a considerable population-level impact on CVD prevention efforts in men.

To date, the few prospective cohort studies that have examined associations between habitual anthocyanin intake and CVD risk have predominantly focused on women. Evidence has suggested that increased intake is associated with a lower risk of nonfatal MI, with the greatest magnitude of association observed in younger women (6, 24). In the 2 studies that have previously examined the relation between anthocyanin intake and CVD mortality, beneficial effects were only observed in women, and an earlier study based on early flavonoid composition databases found no association (25, 30). For stroke, in agreement with our previous findings, there is currently no evidence to our knowledge for a beneficial effect of higher anthocyanin intake in either men or women (24–26, 30). However, a higher habitual flavanone intake was associated with a 19% lower risk of ischemic stroke in 69,622 women over 14 y of follow-up (26), which is similar in magnitude to the risk reduction we observed in men (22%). Our study therefore provides novel data that support a beneficial association between a higher habitual intake of flavanones and lower risk of ischemic stroke and higher anthocyanin intake

TABLE 3
Relation between flavanone and anthocyanin intake and risk of stroke in participants from the Health Professionals Follow-Up Study[1]

	Quintile 1	Quintile 2	Quintile 3	Quintile 4	Quintile 5	*P*-trend
Flavanones, mg/d	7.5	23.6	43.5	64.5	103.9	
Total stroke cases, *n*/person-years	316/179,004	296/179,926	313/177,867	316/172,733	331/168,344	
Age-adjusted	1.0	0.93 (0.79, 1.09)	0.89 (0.76, 1.05)	0.87 (0.75, 1.02)	0.89 (0.76, 1.04)	0.14
Multivariable	1.0	0.96 (0.82, 1.13)	0.94 (0.80, 1.10)	0.92 (0.78, 1.08)	0.95 (0.80, 1.12)	0.51
Ischemic stroke cases, *n*/person-years	200/179,004	173/179,926	168/177,867	188/172,733	172/168,344	
Age-adjusted	1.0	0.86 (0.70, 1.05)	0.76 (0.62, 0.94)	0.83 (0.68, 1.02)	0.74 (0.60, 0.91)	0.01
Multivariable	1.0	0.88 (0.71, 1.08)	0.79 (0.64, 0.98)	0.87 (0.70, 1.07)	0.78 (0.62, 0.97)	0.059
Hemorrhagic stroke cases, *n*/person-years	31/179,004	46/179,926	49/177,867	47/172,733	54/168,344	
Age-adjusted	1.0	1.48 (0.94, 2.34)	1.44 (0.92, 2.27)	1.34 (0.85, 2.12)	1.50 (0.96, 2.34)	0.22
Multivariable	1.0	1.58 (1.00, 2.51)	1.53 (0.96, 2.43)	1.41 (0.88, 2.27)	1.75 (1.09, 2.82)	0.09
Anthocyanins, mg/d	1.9	4.5	7.8	13.7	26.3	
Total stroke cases, *n*/person-years	346/172,074	314/176,190	281/177,780	303/177,669	328/174,162	
Age-adjusted	1.0	0.90 (0.77, 1.05)	0.79 (0.67, 0.93)	0.84 (0.72, 0.98)	0.89 (0.77, 1.04)	0.42
Multivariable	1.0	0.96 (0.82, 1.12)	0.86 (0.73, 1.00)	0.90 (0.77, 1.06)	1.00 (0.85, 1.17)	0.71
Ischemic stroke cases, *n*/person-years	200/172,074	178/176,190	176/177,780	168/177,669	179/174,162	
Age-adjusted	1.0	0.88 (0.72, 1.08)	0.86 (0.70, 1.05)	0.81 (0.66, 0.99)	0.85 (0.69, 1.04)	0.17
Multivariable	1.0	0.94 (0.76, 1.15)	0.93 (0.75, 1.14)	0.86 (0.70, 1.06)	0.93 (0.75, 1.15)	0.51
Hemorrhagic stroke cases, *n*/person-years	48/172,074	58/176,190	32/177,780	42/177669	47/174162	
Age-adjusted	1.0	1.21 (0.83, 1.78)	0.64 (0.41, 1.00)	0.84 (0.55, 1.26)	0.92 (0.62, 1.38)	0.50
Multivariable	1.0	1.26 (0.86, 1.86)	0.70 (0.44, 1.10)	0.88 (0.58, 1.35)	1.06 (0.69, 1.61)	0.93

[1]Values are HRs (95% CIs) unless otherwise specified; median intakes are expressed as mg/d. The multivariable model was adjusted for age, physical activity, smoking, BMI, alcohol, energy, cereal fiber, folate, SFAs, PUFAs, *trans* fatty acids, marital status, hypertension, hypercholesterolemia, and family history of myocardial infarction.

TABLE 4
Common sources of anthocyanins and flavanones[1]

	mg/100 g[2]	Serving size	mg/serving
Anthocyanins			
Blueberries	163.3	1/2 cup	120.8
Blackberries	100.6	1/2 cup	70.4
Black plums	56.0	1 medium	37.0
Red grapes	48.0	1/2 cup	36.5
Raspberries	48.6	1/2 cup	30.2
Red wine	19.3	5 oz	28.3
Cherries	32.0	1/2 cup	22.4
Strawberries	27.0	1/2 cup	20.5
Flavanones			
Orange (fresh/raw)	42.6	1 medium	55.8
Orange juice (fresh/raw)	14.3	6 oz	27.2
Orange juice (chilled)	19.0	6 oz	35.3
Orange juice (blood)	14.4	6 oz	26.8
Grapefruit (fresh/raw)	54.5	0.5 medium	69.8
Grapefruit (fresh/raw—pink)	33.0	0.5 medium	42.2
Grapefruit juice (white)	21.2	6 oz	39.2
Grapefruit juice (pink)	17.8	6 oz	32.9
Lemon (fresh/raw)	49.8	1 medium (58 g)	28.9
Lemon juice	20.7	38 g	7.9
Lime (fresh/raw)	47.4	1 medium (55 g)	26.1
Lime juice	11.5	38 g	4.4
Tangerine (fresh/raw)	18.0	1 medium (84 g)	15.1

[1]1 cup = 225 g; 1 oz = 28 g.
[2]Based on reference 41.

and lower risk of nonfatal MI in men. The reasons for these differential associations with CVD risk are unclear, but of all of the flavonoid subclasses, flavanones are one of the best absorbed and most effective at crossing the blood-brain barrier (42, 43), inhibiting platelet function, and decreasing plaque progression (3, 14). In addition, one flavanone, naringenin, has been shown to localize in the brain after oral ingestion (44). This may provide support for the inverse association between flavanones and risk of ischemic stroke, whereas anthocyanins may be inversely associated with nonfatal MI risk through mechanisms related to improvements in cholesterol efflux capacity or a reduced susceptibility of the myocardium to ischemia or reperfusion injury (12, 22). Of interest, in previous food-based analyses we found that a higher fruit and vegetable intake was associated with a 19% reduction in nonfatal MI risk but only marginally associated with fatal CHD, which is similar to our lack of association for anthocyanin intake and fatal MI risk (45). Together, these population-based data support the available mechanistic findings that flavonoids primarily exert their cardioprotective effects by reducing blood pressure, endothelial function, and insulin sensitivity (3–7).

It is also interesting to note that the magnitude of the inverse association between flavanone intake and risk of ischemic stroke was most pronounced in the participants aged ≥65 y, whereas the association between anthocyanin intake and risk of nonfatal MI did not differ in younger or older men. However, we did see that the inverse association was stronger in participants who were not hypertensive than in those taking hypertensive medication. Therefore, in this cohort of older men aged 40–75 y at baseline who were followed for >24 y, medication use may have exceeded the capacity for dietary constituents such as fruit-based flavonoids to reduce the risk of CVD in older individuals. These data reinforce the importance of dietary intervention strategies for CVD prevention.

Although the overall range in anthocyanin and flavanone intake was similar across the cohort (0–613 mg/d for anthocyanin intake and 0–728 mg/d for flavanones), the variability in intake across the middle 3 quintiles was only 9 mg for anthocyanins and 41 mg for flavanones. With such small differences in the distribution of anthocyanin intake, measurement error or misclassification is likely to be greatest. When we compared extreme deciles of intake, those in the top decile of anthocyanin intake (median intake: 35.9 mg/d) had a 19% lower risk of nonfatal MI, suggesting potentially greater benefits at higher intakes. However, for flavanones, with a wide range in intake across quintiles (median intake: 7.5 mg/d in quintile 1 and 103.9 mg/d in quintile 5), we observed a threshold effect and did not see any further reduction in risk when we compared extreme deciles of intake. To our knowledge, very few dose-response trials on anthocyanins and flavanones and biomarkers of CVD risk have been conducted, but given our knowledge of other subclasses, including the isoflavones and flavan-3-ols, there is likely a threshold of intake required for a biological effect, and very low concentrations of intake are unlikely to be bioactive (46, 47).

Intriguingly, to our knowledge only a few randomized controlled trials have examined the impact of anthocyanins and flavanones on cardiometabolic health relative to other flavonoids such as the flavan-3-ols present in tea and cocoa and the isoflavones present in soy (48). In several short-term interventions (<2 mo), anthocyanin-rich food intake (predominantly consumed as blueberries) resulted in a reduction in both systolic and diastolic blood pressure (15–17) and favorable changes in arterial stiffness (17, 18). A recent 3-mo dose-response study in which participants consumed a strawberry beverage equivalent to 250 and 500 g fresh strawberries/d (containing 78 and 155 mg anthocyanins/d) demonstrated beneficial effects on total and LDL cholesterol concentrations in the high intake group (21), potentially mediated by improvements in cholesterol efflux capacity (22). In a 6-mo study, a considerable decrease in inflammatory biomarkers after the intake of purified anthocyanins (320 mg/d) in patients with hypercholesterolemia (23) was observed, whereas a single dose of a strawberry beverage (containing 39 mg anthocyanins) attenuated the 6-h postprandial inflammatory response to a high-carbohydrate and moderate-fat meal in overweight dyslipidemic participants (49). These data are supported by a growing body of evidence from animal models and in vitro experiments that have shown a cardio- and neuroprotective role. Anthocyanins and their metabolites traverse the blood-brain barrier (50) and in animal models inhibit atherosclerosis development, alter cell signaling pathways involved in vascular inflammation, attenuate cyclophospamide-induced cardiac injury, and reduce infarct size after coronary occlusion and perfusion (11, 12, 51).

For flavanones, animal and in vitro data also support cardio- and neuroprotective effects (3, 13, 42, 43, 52, 53). Flavanones and their metabolites improve endothelial function principally via improved blood flow, enhanced production or reduced NAD(P)H oxidase-dependent elimination of endothelial nitric oxide, and inhibited platelet function (14). These mechanisms may underline the observed beneficial effect on ischemic stroke but no observed effect in relation to hemorrhagic stroke, which is more likely related to mechanisms that interfere with platelet function

and clotting. Our results for hemorrhagic stroke may also relate to the small number of cases (n = 227). In vitro, the flavanone aglycones naringenin and hesperetin exert a diverse array of potentially anti-inflammatory effects by interacting with mitogen-activated protein kinase, P13 kinase/Akt, and protein kinase C signaling pathways (52, 54). In mice fed a high-fat diet supplemented with a nutritionally relevant dose of naringin (the glycoside of naringenin), a 41% decrease in plaque progression was observed (3), suggesting that antiatherogenic effects of flavanones are achievable at dietary-relevant doses. To our knowledge, only a handful of placebo-controlled trials with flavanones have been conducted to date. The first long-term trial, a 6-mo flavanone intervention trial with grapefruit juice (containing 210 mg naringenin glycosides/d) in healthy postmenopausal women improved arterial stiffness (19). In another 2-mo intervention with flavanone-rich orange juice, global cognitive function was improved (55), which is consistent with effects previously observed in animal models (44). In 2 short-term trials (3–4 wk), improvements in endothelial function, endothelium-dependent microvascular reactivity, and a reduction in diastolic blood pressure were observed (13, 20), and plasma hesperetin metabolites (phase II) were associated with the beneficial effects on endothelial function (20).

In the habitual diet, flavanones are almost exclusively associated with citrus fruits, with higher concentrations present in whole fruits than in products such as juices (Table 4) (40, 41) because the solid parts of the fruit, particularly the albedo (white spongy section) and the membranes separating the segments, contain higher concentrations than the juice vesicles (pulp) (56). Although on average, blueberries contain the highest concentrations of anthocyanins, a range of other fruits also have the potential to contribute to anthocyanin intake, including other berries, grapes, and grape-derived products including wine (Table 4) (40, 41). Together, these data show that these fruit-based flavonoids can be readily incorporated into the diet.

The strengths of this study include its prospective design, large sample size with long-term follow-up, detailed data on important risk factors and confounders, and comprehensive assessment of habitual anthocyanin and flavanone intake. The limitations of our study also warrant discussion. Although we adjusted for possible confounders that are strongly associated with MI and stroke risk (including BMI, smoking, and family history), there is still the possibility of residual or unmeasured confounding from additional unmeasured factors or measurement error, which may be greatest when comparing those consuming the highest and lowest concentrations of anthocyanins. However, given our detailed adjustment for a comprehensive set of confounders, it is unlikely that these unmeasured factors would account fully for the observed results.

The flavonoid content of foods varies depending on growing conditions and manufacturing processes, but despite this variation, these data allow us to rank intakes and compare high and low intakes in large population groups. There are currently no specific biomarkers for anthocyanins to our knowledge because they are extensively degraded after intake, including the production of an extensive range of microbially derived metabolites, supporting a strong interplay between anthocyanins and the microbiome (57–59). For flavanones, recent methodologic developments provide potential biomarkers for an objective assessment of this subclass in future studies (60). The future direction is to identify and develop validated and robust biomarkers that will best reflect intake and differential metabolism of anthocyanins and flavanones. This will allow us to more accurately determine optimal intakes of these compounds to reduce CVD risk. It is possible that our findings might have been caused by other constituents found in the foods that contribute most to this subclass; however, the addition of other potentially beneficial constituents of fruits, including fiber and folate, to our multivariate model did not substantially attenuate the observed relations, suggesting that anthocyanins and flavanones may be another important cardioprotective constituent. However, in a population-based study such as ours it is impossible to disentangle the relative influence of all constituents of fruits.

Our findings suggest that bioactive compounds present in both citrus and red- or blue-colored fruits commonly consumed in the habitual diet may be associated with a lower risk of CVD in men. Further prospective studies are needed to confirm these associations, including studies with biomarkers of CVD risk to elucidate mechanisms. Randomized trials focusing on commonly consumed anthocyanin- and flavanone-rich foods are also needed to examine dose-response effects, as are trials of longer duration to assess clinically relevant endpoints.

The authors' responsibilities were as follows—AC and EBR: conducted the statistical analysis, interpreted the data, and drafted the manuscript; MB: critically reviewed the manuscript; and all authors: read and approved the final manuscript. AC and EBR received funding from the US Blueberry Highbush Council for a separate project unrelated to this publication. None of the other authors reported a conflict of interest related to the study.

REFERENCES

1. Ezzati M, Riboli E. Behavioral and dietary risk factors for noncommunicable diseases. N Engl J Med 2013;369:954–64.
2. Muraki I, Imamura F, Manson JE, Hu FB, Willett WC, van Dam RM, Sun Q. Fruit consumption and risk of type 2 diabetes: results from three prospective longitudinal cohort studies. BMJ 2013;347:f5001.
3. Chanet A, Milenkovic D, Manach C, Mazur A, Morand C. Citrus flavanones: what is their role in cardiovascular protection? J Agric Food Chem 2012;60:8809–22.
4. Wedick NM, Pan A, Cassidy A, Rimm EB, Sampson L, Rosner B, Willett W, Hu FB, Sun Q, van Dam RM. Dietary flavonoid intakes and risk of type 2 diabetes in US men and women. Am J Clin Nutr 2012;95:925–33.
5. Cassidy A, O'Reilly EJ, Kay C, Sampson L, Franz M, Forman JP, Curhan G, Rimm EB. Habitual intake of flavonoid subclasses and incident hypertension in adults. Am J Clin Nutr 2011;93:338–47.
6. Cassidy A, Mukamal KJ, Liu L, Franz M, Eliassen AH, Rimm EB. High anthocyanin intake is associated with a reduced risk of myocardial infarction in young and middle-aged women. Circulation 2013;127:188–96.
7. Cassidy A, Rogers G, Peterson JJ, Dwyer JT, Lin H, Jacques PF. Higher dietary anthocyanin and flavonol intakes are associated with anti-inflammatory effects in a population of US adults. Am J Clin Nutr 2015;102:172–81.
8. WHO. Global health risks: mortality and burden of disease attributable to selected major risks. Geneva (Switzerland): WHO; 2009.
9. WHO. World health statistics 2009. Geneva (Switzerland): WHO; 2009.
10. Lackland DT, Roccella EJ, Deutsch AF, Fornage M, George MG, Howard G, Kissela BM, Kittner SJ, Lichtman JH, Lisabeth LD, et al. Factors influencing the decline in stroke mortality: a statement from the American Heart Association/American Stroke Association. Stroke 2014;45:315–53.
11. Lamy S, Beaulieu E, Labbe D, Bedard V, Moghrabi A, Barrette S, Kissela BM, Kittner SJ, Lichtman JH, Lisabeth LD, et al. Delphinidin, a dietary anthocyanidin, inhibits platelet-derived growth factor ligand/receptor (PDGF/PDGFR) signaling. Carcinogenesis 2008;29:1033–41.

12. Toufektsian MC, de Lorgeril M, Nagy N, Salen P, Donati MB, Giordano L, Mock HP, Peterek S, Matros A, Petroni K, et al. Chronic dietary intake of plant-derived anthocyanins protects the rat heart against ischemia-reperfusion injury. J Nutr 2008;138:747–52.
13. Rizza S, Muniyappa R, Iantorno M, Kim JA, Chen H, Pullikotil P, Senese N, Tesauro M, Lauro D, Cardillo C, et al. Citrus polyphenol hesperidin stimulates production of nitric oxide in endothelial cells while improving endothelial function and reducing inflammatory markers in patients with metabolic syndrome. J Clin Endocrinol Metab 2011;96:E782–92.
14. Jin YR, Han XH, Zhang YH, Lee JJ, Lim Y, Chung JH, Yun YP. Antiplatelet activity of hesperetin, a bioflavonoid, is mainly mediated by inhibition of PLC-gamma2 phosphorylation and cyclooxygenase-1 activity. Atherosclerosis 2007;194:144–52.
15. Erlund I, Koli R, Alfthan G, Marniemi J, Puukka P, Mustonen P, Mattila P, Jula A. Favorable effects of berry consumption on platelet function, blood pressure, and HDL cholesterol. Am J Clin Nutr 2008;87:323–31.
16. Basu A, Du M, Leyva MJ, Sanchez K, Betts NM, Wu M, Aston CE, Lyons TJ. Blueberries decrease cardiovascular risk factors in obese men and women with metabolic syndrome. J Nutr 2010;140:1582–7.
17. Johnson SA, Figueroa A, Navaei N, Wong A, Kalfon R, Ormsbee LT, Feresin RG, Elam ML, Hooshmand S, Payton ME, et al. Daily blueberry consumption improves blood pressure and arterial stiffness in postmenopausal women with pre- and stage 1-hypertension: a randomized, double-blind, placebo-controlled clinical trial. J Acad Nutr Diet 2015;115:369–77.
18. Dohadwala MM, Holbrook M, Hamburg NM, Shenouda SM, Chung WB, Titas M, Kluge MA, Wang N, Palmisano J, Milbury PE, et al. Effects of cranberry juice consumption on vascular function in patients with coronary artery disease. Am J Clin Nutr 2011;93:934–40.
19. Habauzit V, Verny MA, Milenkovic D, Barber-Chamoux N, Mazur A, Dubray C, Morand C. Flavanones protect from arterial stiffness in postmenopausal women consuming grapefruit juice for 6 mo: a randomized, controlled, crossover trial. Am J Clin Nutr 2015;102:66–74.
20. Morand C, Dubray C, Milenkovic D, Lioger D, Martin JF, Scalbert A, Mazur A. Hesperidin contributes to the vascular protective effects of orange juice: a randomized crossover study in healthy volunteers. Am J Clin Nutr 2011;93:73–80.
21. Basu A, Betts NM, Nguyen A, Newman ED, Fu D, Lyons TJ. Freeze-dried strawberries lower serum cholesterol and lipid peroxidation in adults with abdominal adiposity and elevated serum lipids. J Nutr 2014;144:830–7.
22. Zhu Y, Huang X, Zhang Y, Wang Y, Liu Y, Sun R, Xia M. Anthocyanin supplementation improves HDL-associated paraoxonase 1 activity and enhances cholesterol efflux capacity in subjects with hypercholesterolemia. J Clin Endocrinol Metab 2014;99:561–9.
23. Zhu Y, Ling W, Guo H, Song F, Ye Q, Zou T, Li D, Zhang Y, Li G, Xiao Y, et al. Anti-inflammatory effect of purified dietary anthocyanin in adults with hypercholesterolemia: a randomized controlled trial. Nutr Metab Cardiovasc Dis 2013;23:843–9.
24. Mink PJ, Scrafford CG, Barraj LM, Harnack L, Hong CP, Nettleton JA, Jacobs DR Jr. Flavonoid intake and cardiovascular disease mortality: a prospective study in postmenopausal women. Am J Clin Nutr 2007;85:895–909.
25. McCullough ML, Peterson JJ, Patel R, Jacques PF, Shah R, Dwyer JT. Flavonoid intake and cardiovascular disease mortality in a prospective cohort of US adults. Am J Clin Nutr 2012;95:454–64.
26. Cassidy A, Rimm EB, O'Reilly EJ, Logroscino G, Kay C, Chiuve SE, Rexrode KM. Dietary flavonoids and risk of stroke in women. Stroke 2012;43:946–51.
27. Jacques PF, Cassidy A, Rogers G, Peterson JJ, Dwyer JT. Dietary flavonoid intakes and CVD incidence in the Framingham Offspring Cohort. Br J Nutr 2015;114:1496–503.
28. Peterson JJ, Dwyer JT, Jacques PF, McCullough ML. Associations between flavonoids and cardiovascular disease incidence or mortality in European and US populations. Nutr Rev 2012;70:491–508.
29. Wang X, Ouyang YY, Liu J, Zhao G. Flavonoid intake and risk of CVD: a systematic review and meta-analysis of prospective cohort studies. Br J Nutr 2014;111:1–11.
30. Mursu J, Voutilainen S, Nurmi T, Tuomainen TP, Kurl S, Salonen JT. Flavonoid intake and the risk of ischaemic stroke and CVD mortality in middle-aged Finnish men: the Kuopio Ischaemic Heart Disease Risk Factor Study. Br J Nutr 2008;100:890–5.
31. Knekt P, Kumpulainen J, Jarvinen R, Rissanen H, Heliovaara M, Reunanen A, Hakulinen T, Aromaa A. Flavonoid intake and risk of chronic diseases. Am J Clin Nutr 2002;76:560–8.
32. Rimm EB, Giovannucci EL, Stampfer MJ, Colditz GA, Litin LB, Willett WC. Reproducibility and validity of an expanded self-administered semiquantitative food frequency questionnaire among male health professionals. Am J Epidemiol 1992;135:1114–26.
33. Grobbee DE, Rimm EB, Giovannucci E, Colditz G, Stampfer M, Willett W. Coffee, caffeine, and cardiovascular disease in men. N Engl J Med 1990;323:1026–32.
34. WHO. Cardiovascular survey methods. Geneva (Switzerland): WHO; 1982.
35. Walker AE, Robins M, Weinfeld FD. The National Survey of Stroke. Clinical findings. Stroke 1981;12(Suppl 1):I13–44.
36. Salvini S, Hunter DJ, Sampson L, Stampfer MJ, Colditz GA, Rosner B, Willett WC. Food-based validation of a dietary questionnaire: the effects of week-to-week variation in food consumption. Int J Epidemiol 1989;18:858–67.
37. Feskanich D, Rimm EB, Giovannucci EL, Colditz GA, Stampfer MJ, Litin LB, Willett WC. Reproducibility and validity of food intake measurements from a semiquantitative food frequency questionnaire. J Am Diet Assoc 1993;93:790–6.
38. Krogholm KS, Bysted A, Brantsaeter AL, Jakobsen J, Rasmussen SE, Kristoffersen L, Toft U. Evaluation of flavonoids and enterolactone in overnight urine as intake biomarkers of fruits, vegetables and beverages in the Inter99 cohort study using the method of triads. Br J Nutr 2012;108:1904–12.
39. Therneau TM, Grambsch PM, editors. Modeling survival data: extending the Cox Model. New York: Springer; 2000.
40. Bhagwat S, Haytowitz DB, Holden JM, editors. USDA database for the flavonoid content of selected foods: release 3.1. Washington (DC): USDA; 2014.
41. USDA. National nutrient database for standard reference: release 28. Washington (DC): USDA; 2015.
42. Rendeiro C, Rhodes JS, Spencer JP. The mechanisms of action of flavonoids in the brain: direct versus indirect effects. Neurochem Int 2015;89:126–39.
43. Williamson G, Manach C. Bioavailability and bioefficacy of polyphenols in humans. II. Review of 93 intervention studies. Am J Clin Nutr 2005;81(1 Suppl):243S–55S.
44. Youdim KA, Dobbie MS, Kuhnle G, Proteggente AR, Abbott NJ, Rice-Evans C. Interaction between flavonoids and the blood-brain barrier: in vitro studies. J Neurochem 2003;85:180–92.
45. Bhupathiraju SN, Wedick NM, Pan A, Manson JE, Rexrode KM, Willett WC, Rimm EB, Hu FB. Quantity and variety in fruit and vegetable intake and risk of coronary heart disease. Am J Clin Nutr 2013;98:1514–23.
46. Liu XX, Li SH, Chen JZ, Sun K, Wang XJ, Wang XG, Hui RT. Effect of soy isoflavones on blood pressure: a meta-analysis of randomized controlled trials. Nutr Metab Cardiovasc Dis 2012;22:463–70.
47. Hooper L, Kay C, Abdelhamid A, Kroon PA, Cohn JS, Rimm EB, Cassidy A. Effects of chocolate, cocoa, and flavan-3-ols on cardiovascular health: a systematic review and meta-analysis of randomized trials. Am J Clin Nutr 2012;95:740–51.
48. Hooper L, Kroon PA, Rimm EB, Cohn JS, Harvey I, Le Cornu KA, Ryder JJ, Hall WL, Cassidy A. Flavonoids, flavonoid-rich foods, and cardiovascular risk: a meta-analysis of randomized controlled trials. Am J Clin Nutr 2008;88:38–50.
49. Edirisinghe I, Banaszewski K, Cappozzo J, Sandhya K, Ellis CL, Tadapaneni R, Kappagoda CT, Burton-Freeman BM. Strawberry anthocyanin and its association with postprandial inflammation and insulin. Br J Nutr 2011;106:913–22.
50. Milbury PE, Kalt W. Xenobiotic metabolism and berry flavonoid transport across the blood-brain barrier. J Agric Food Chem 2010;58:3950–6.
51. Liu Y, Tan D, Shi L, Liu X, Zhang Y, Tong C, Song D, Hou M. Blueberry anthocyanins-enriched extracts attenuate cyclophosphamide-induced cardiac injury. PLoS One 2015;10:e0127813.
52. Vafeiadou K, Vauzour D, Lee HY, Rodriguez-Mateos A, Williams RJ, Spencer JP. The citrus flavanone naringenin inhibits inflammatory signalling in glial cells and protects against neuroinflammatory injury. Arch Biochem Biophys 2009;484:100–9.
53. Menze ET, Tadros MG, Abdel-Tawab AM, Khalifa AE. Potential neuroprotective effects of hesperidin on 3-nitropropionic acid-induced neurotoxicity in rats. Neurotoxicology 2012;33:1265–75.
54. Choi EJ, Ahn WS. Neuroprotective effects of chronic hesperetin administration in mice. Arch Pharm Res 2008;31:1457–62.

55. Kean RJ, Lamport DJ, Dodd GF, Freeman JE, Williams CM, Ellis JA, Butler LT, Spencer JP. Chronic consumption of flavanone-rich orange juice is associated with cognitive benefits: an 8-wk, randomized, double-blind, placebo-controlled trial in healthy older adults. Am J Clin Nutr 2015;101:506–14.
56. Thomas-Barberan FA, Clifford MN. Flavanones, chalcones and dihydrochalcones—nature, occurrence and dietary burden. J Sci Food Agric 2000;80:1073–80.
57. Czank C, Cassidy A, Zhang Q, Morrison DJ, Preston T, Kroon PA, Botting NP, Kay CD. Human metabolism and elimination of the anthocyanin, cyanidin-3-glucoside: a (13)C-tracer study. Am J Clin Nutr 2013;97:995–1003.
58. de Ferrars RM, Czank C, Zhang Q, Botting NP, Kroon PA, Cassidy A, Kay CD. The pharmacokinetics of anthocyanins and their metabolites in humans. Br J Pharmacol 2014;171:3268–82.
59. Schär MY, Curtis PJ, Hazim S, Ostertag LM, Kay CD, Potter JF, Cassidy A. Orange juice-derived flavanone and phenolic metabolites do not acutely affect cardiovascular risk biomarkers: a randomized, placebo-controlled, crossover trial in men at moderate risk of cardiovascular disease. Am J Clin Nutr 2015;101:931–8.
60. Achaintre D, Bulete A, Cren-Olive C, Li L, Rinaldi S, Scalbert A. Differential isotope labeling of 38 dietary polyphenols and their quantification in urine by liquid chromatography electrospray ionization tandem mass spectrometry. Anal Chem 2016;88:2637–44.
