# Supplementary material for: A systematic comprehensive longitudinal evaluation of dietary factors associated with acute myocardial infarction and fatal coronary heart disease
Source: Nat Commun. 2020 Nov 27;11:6074. doi: 10.1038/s41467-020-19888-2 (PMC7699643; doi:10.1038/s41467-020-19888-2)
Supplement: Supplementary file 4 — Supplementary Software [file 41467_2020_19888_MOESM4_ESM.zip › EWAS-NHS-master/KG-SI/Rest/Hu (1999).pdf]

Dietary intake of α-linolenic acid and risk of fatal ischemic heart disease among women[1–3]

Frank B Hu, Meir J Stampfer, JoAnn E Manson, Eric B Rimm, Alicja Wolk, Graham A Colditz, Charles H Hennekens, and Walter C Willett

See corresponding editorial on page 827.

ABSTRACT
Background: Experimental studies in laboratory animals and humans suggest that α-linolenic acid (18:3n−3) may reduce the risk of arrhythmia.
Objective: The objective was to examine the association between dietary intake of α-linolenic acid and risk of fatal ischemic heart disease (IHD).
Design: This was a prospective cohort study. The intake of α-linolenic acid was derived from a 116-item food-frequency questionnaire completed in 1984 by 76 283 women without previously diagnosed cancer or cardiovascular disease.
Results: During 10 y of follow-up, we documented 232 cases of fatal IHD and 597 cases of nonfatal myocardial infarction. After adjustment for age, standard coronary risk factors, and dietary intake of linoleic acid and other nutrients, a higher intake of α-linolenic acid was associated with a lower relative risk (RR) of fatal IHD; the RRs from the lowest to highest quintiles were 1.0, 0.99, 0.90, 0.67, and 0.55 (95% CI: 0.32, 0.94; P for trend = 0.01). For nonfatal myocardial infarction there was only a modest, nonsignificant trend toward a reduced risk when extreme quintiles were compared (RR: 0.85; 95% CI: 0.61, 1.19; P for trend = 0.50). A higher intake of oil and vinegar salad dressing, an important source of α-linolenic acid, was associated with reduced risk of fatal IHD when women who consumed this food ≥5–6 times/wk were compared with those who rarely consumed this food (RR: 0.46; 95% CI: 0.27, 0.76; P for trend = 0.001).
Conclusions: This study supports the hypothesis that a higher intake of α-linolenic acid is protective against fatal IHD. Higher consumption of foods such as oil-based salad dressing that provide polyunsaturated fats, including α-linolenic acid, may reduce the risk of fatal IHD. Am J Clin Nutr 1999;69:890–7.

KEY WORDS Ischemic heart disease, diet, α-linolenic acid, risk, Nurses' Health Study, trans fatty acids, women

INTRODUCTION

Experimental studies have suggested an antiarrhythmic effect of α-linolenic acid (18:3n−3). In animal experiments in which arrhythmias were induced by coronary occlusion and reperfusion, significant reductions in the incidence of ventricular fibrillation and cardiac mortality were observed in rats fed with α-linolenic acid–rich diets (1, 2). The antiarrhythmic effect of α-linolenic acid may be attributable to an increased electrical threshold for induction of ventricular fibrillation (1). In a secondary prevention trial of myocardial infarction (MI), patients consuming the experimental diet with the higher α-linolenic acid content experienced a significant reduction in cardiovascular death (3).

We examined the dietary intake of α-linolenic acid in relation to the risk of fatal ischemic heart disease (IHD) among participants in the Nurses' Health Study. Specifically, we conducted 2 parallel analyses: 1 to assess risk of fatal IHD among women without prior diagnosed IHD at baseline and 1 limited to women with a prior MI. Because α-linolenic acid is prone to oxidative modification, the availability of antioxidants such as vitamin E may be important for its biological effects. Thus, we hypothesized a priori that the inverse association between α-linolenic acid intake and fatal IHD would be stronger among vitamin E supplement users. In addition, because trans fatty acids can inhibit the desaturation of α-linolenic acid and may thus alter its biological function (4), we examined whether intake of trans fatty acids might modify the relation between α-linolenic acid and risk of fatal IHD.

SUBJECTS AND METHODS

Study population

The Nurses' Health Study cohort was established in 1976 when 121 700 female, registered nurses aged 30–55 y and residing in 11 large US states completed a mailed questionnaire about

[1] From the Departments of Nutrition and Epidemiology, Harvard School of Public Health; Channing Laboratory, and the Division of Preventive Medicine, Department of Medicine, Brigham and Women's Hospital and Harvard Medical School, Boston.

[2] Supported by grants HL34594, CA40356, and DK 46200 and nutrition training grant T32DK07703 from the National Institutes of Health.

[3] Address reprint requests to FB Hu, Department of Nutrition, Harvard School of Public Health, 665 Huntington Avenue, Boston, MA 02115. E-mail: Frank.Hu@channing.harvard.edu.

Received August 13, 1998.
Accepted for publication December 21, 1998.

 © 1999 American Society for Clinical Nutrition

Downloaded from https://academic.oup.com/ajcn/article-abstract/69/5/890/4714835 by Northeastern University Libraries user on 23 May 2019

their medical history and lifestyle. Every 2 y, follow-up questionnaires were sent to the participants to update information on potential risk factors and to identify newly diagnosed cases of coronary and other diseases.

In 1980, a 61-item food-frequency questionnaire was included to assess the intake of specific fats and other nutrients. In 1984, the food-frequency questionnaire was revised to include questions about 116 individual food items, many of which had been combined into food groupings in the original questionnaire. Similar questionnaires were used to update dietary information in 1986 and 1990. Because the revised questionnaires contained more food items that were critical for the assessment of α-linolenic acid intake, we used data from the 1984 questionnaire as baseline.

After ≥4 mailings, 81 757 women returned the 1984 diet questionnaire. In the primary analyses, we excluded those who left ≥11 items blank, those with implausibly high or low scores for total food and energy intakes [ie, <2761 kJ (660 kcal) or >14 644 kJ/d (3500 kcal/d)], and those with previously diagnosed cancer, angina, MI, stroke, or other cardiovascular diseases at baseline to avoid biased reporting of diet induced by the presence of these diseases. The final 1984 baseline population for the analysis of fatal IHD among women without prior IHD included 76 283 women. For the analysis of fatal IHD among women with a prior MI, we included 1117 women who completed the 1984 questionnaire and were diagnosed with a nonfatal MI at baseline or during follow-up. The study was approved by the Human Research Committee at Brigham and Women's Hospital.

Dietary assessment

The reproducibility and validity of the food-frequency questionnaires were described in detail elsewhere (5). The correlation between the calculated dietary intake of a fatty acid from the food-frequency questionnaire and the proportion of the fatty acid in adipose tissue was 0.34 ($P < 0.001$) for linolenic acid, 0.37 ($P < 0.001$) for linoleic acid, and 0.40 ($P < 0.001$) for trans fatty acids (6). The correlation coefficients for linolenic acid intake were 0.57 between the 1984 and 1986 questionnaires and 0.48 between the 1986 and 1990 questionnaires.

To calculate intakes of linolenic acid and other nutrients, a commonly used unit or portion size for each food (eg, one egg or one slice of bread) was specified and the participants were asked how often on average during the previous year they had consumed that amount. Nine responses were possible, ranging from "never" to "≥6 times/d." The intake of nutrients was computed by multiplying the frequency of consumption of each unit of food by the nutrient content of the specified portions. Composition values for linolenic acids and other nutrients were obtained from the Harvard University Food Composition Database (compiled on 22 November 1993) derived from US Department of Agriculture sources (7) and supplemented with manufacturer information. Because the US Department of Agriculture database has values only for total linolenic acid, we accounted for γ-linolenic acid in animal fats when computing intakes of α-linolenic acid. α-Linolenic acid constitutes most of the total linolenic acid in the diet, but only accounts for ≈60% of total linolenic acid in beef fat (F Sacks, personal communication, 1998). We therefore subtracted 40% of the total linolenic acid content in beef. [Linolenic acid is present in only small amounts in beef and other animal fats (8), so analyses using total linolenic acid or the adjusted value yielded near identical results.] In addition, we repeated the primary analyses using α-linolenic acid from plant sources only.

Ascertainment of cases

The primary endpoint for this study was fatal IHD that occurred after the return of the 1984 questionnaire but before 1 June 1994; nonfatal MI was a secondary endpoint. Deaths were identified from the National Death Index, next of kin, or the postal system. Using all sources combined, we estimated that follow-up for the deaths was >98% complete (9). Fatal IHD was defined as fatal MI if it was confirmed by hospital records or autopsy, or if IHD was listed as the primary cause of death on the death certificate and evidence of previous IHD was available. The statement of the cause of death on the death certificates was never relied on by itself as providing sufficient confirmation of death due to IHD.

We requested permission to review medical records from women who reported having a nonfatal MI on a follow-up questionnaire. The records were reviewed by study physicians, who had no knowledge of the self-reported risk-factor status. Nonfatal MI was confirmed if it met the criteria of the World Health Organization of symptoms plus either diagnostic electrocardiographic changes or elevated cardiac enzyme concentrations (10). MIs that required hospital admission and for which confirmatory information was obtained by interview or letter, but for which no medical records were available, were designated as probable (17% of total MI cases). We included all confirmed and probable cases in the analyses because results were the same after probable cases were excluded.

Data analysis

For the analysis of fatal IHD among women without prior IHD at baseline, person-years (the number of persons studied times the number of years of follow-up) for each participant were calculated from the date of return of the 1984 questionnaire to the date of the fatal IHD event, death, or 1 June 1994. Women with fatal IHD or cancer noted on previous questionnaires were excluded from subsequent follow-up; thus, the cohort at risk included only those who remained free from cancer and were alive at the beginning of each follow-up interval. For the analysis of fatal IHD among women with a prior MI, person-years were calculated from the date of return of the 1984 questionnaire to the date of the fatal IHD event.

Women were grouped in quintiles of intake of α-linolenic acid adjusted for total energy intake by using regression analysis (11) based on the 1984 questionnaire. Incidence rates were calculated by dividing the number of events by person-years of follow-up in each quintile. The relative risk (RR) was computed as the rate in a specific category of α-linolenic acid intake divided by that in the lowest quintile, with adjustment for 5-y age categories and smoking status [never, past, and current (1–14, 15–24, and ≥25 cigarettes/d). A multivariate pooled logistic model (12) was used to estimate the RRs and 95% CIs of fatal IHD associated with various intakes of α-linolenic acid and foods that were primary sources of α-linolenic acid in this cohort, with simultaneous adjustment for other risk factors. Tests of linear trend across increasing categories of α-linolenic acid or food intakes were conducted by treating the categories as a continuous variable and assigning the median intake for the category as its value. Nondietary covariates, including age, cigarette smoking, body mass index (BMI), menopausal hormone

Downloaded from https://academic.oup.com/ajcn/article-abstract/69/5/890/4714835 by Northeastern University Libraries user on 23 May 2019

TABLE 1
Major contributors to the overall intake of α-linolenic acid based on the 1984 food-frequency questionnaire in the Nurses' Health Study

Foods	Value
	%
Mayonnaise or other creamy salad dressing	16.7
Oil and vinegar salad dressing (eg, Italian)	12.2
Margarine	6.8
Beef, pork, or lamb as main dish	6.5
Milk	4.1
Cheese	3.9
Iceberg lettuce	2.0
Dark bread	1.6
Ice cream	1.6
Pie	1.4
Tomato sauce	1.4
Broccoli	1.3

use, alcohol use, multivitamin use, and vitamin E supplement use, were updated biennially. Aspirin use was assessed in 1984 and 1988. Vigorous exercise was assessed in 1980. All dietary variables were assessed in 1984, 1986, and 1990. Our primary analyses used the baseline diet. We also conducted alternative analyses using the updated dietary information (13). In the updated analyses, we related incidence of fatal IHD to the cumulative average α-linolenic acid intake from all available questionnaires up to the start of each 2-y follow-up interval. In the multivariate analyses of the association between α-linolenic acid intake and risk of fatal IHD among prevalent MI patients, we adjusted for the same covariates described above.

RESULTS

During 698750 person-years of follow-up, we documented 232 cases of fatal IHD and 597 nonfatal MIs. Among 1117 women with prevalent MI, 74 had fatal IHD during the follow-up. The mean (±SD) daily intake of α-linolenic acid in 1984 was 1.10 ± 0.45 g, or 0.57 ± 0.16% of total energy intake. Median daily energy-adjusted α-linolenic acid intake ranged from 0.71 g in the lowest quintile to 1.36 g in the highest quintile. Approximately 70% of α-linolenic acid was from vegetable or plant sources (eg, salad dressings and vegetable oils) (Table 1). On the basis of the 116 food items in the food-frequency questionnaire, the largest contributors (in absolute percentage) to the overall intake of α-linolenic acid were mayonnaise or other creamy salad dressing (16.7%); oil and vinegar (eg, Italian) salad dressing (12.2%); margarine (6.8%); beef, pork, and lamb as a main dish (6.5%); dairy products; and green-leafy vegetables such as iceberg lettuce.

Mean age and BMI, prevalence of current smoking, history of hypertension, parental MI before 60 y of age, current postmenopausal hormone use, regular exercise, multivitamin use, vitamin E supplement use, and aspirin use did not vary appreciably according to intake of α-linolenic acid (Table 2). α-Linolenic acid intake was correlated with intakes of linoleic acid (r = 0.68), oleic acid (r = 0.56), and trans fatty acids (r = 0.39), but not with n−3 fatty acids from fish (r = 0.07).

TABLE 2
Relation of potential ischemic heart disease risk factors to energy-adjusted α-linolenic acids intake in 1984[1]

	Quintiles of α-linolenic acid intake				
	1 (lowest, 0.71 g/d)	2 (0.86 g/d)	3 (0.98 g/d)	4 (1.12 g/d)	5 (highest, 1.36 g/d)
Current smokers (%)	24.4	23.7	23.5	23.5	25.6
History of hypertension (%)	22.1	21.6	20.8	22.0	21.5
History of diabetes (%)	3.0	2.8	3.0	3.0	3.4
History of hypercholesterolemia (%)	9.1	8.2	7.9	8.1	8.2
Parental MI before age 65 y (%)	14.5	14.5	14.5	14.9	14.9
Current postmenopausal hormone use (%)	19.5	18.4	19.6	19.4	19.6
Vigorous exercise ≥1/wk (%)	43.0	42.5	42.7	44.5	44.2
Multivitamin use (%)	39.7	37.4	37.0	35.7	35.2
Vitamin E supplement use (%)	18.5	16.3	16.4	16.9	17.4
Aspirin use ≥1/wk (%)	44.4	45.8	45.4	45.1	42.7
Age (y)	50.1 ± 7.2[2]	50.0 ± 7.2	50.2 ± 7.2	50.4 ± 7.2	50.8 ± 7.1
Alcohol (g/d)	8.3 ± 14.1	6.9 ± 11.2	6.8 ± 10.5	6.8 ± 10.0	6.5 ± 9.6
BMI (kg/m^2)	24.8 ± 4.7	25.0 ± 4.7	25.0 ± 4.7	25.1 ± 4.8	25.2 ± 4.8
Saturated fat (g/d)	20.0 ± 4.3	21.8 ± 4.1	22.5 ± 4.3	22.9 ± 4.5	23.4 ± 5.0
Linoleic acid (g/d)	7.9 ± 2.4	9.0 ± 2.1	9.9 ± 2.0	10.8 ± 2.1	13.0 ± 2.9
Oleic acid (g/d)	17.9 ± 4.1	19.7 ± 3.6	20.6 ± 3.5	21.2 ± 3.7	22.2 ± 4.0
Eicosapentaenoic and docosahexaenoic acids (g/d)	0.17 ± 0.16	0.17 ± 0.15	0.17 ± 0.14	0.18 ± 0.15	0.20 ± 0.17
trans Fatty acids (g/d)	2.7 ± 0.9	3.2 ± 0.9	3.4 ± 1.0	3.6 ± 1.0	3.9 ± 1.1
Dietary vitamin C (mg/d)	143 ± 75	137 ± 62	134 ± 58	133 ± 57	134 ± 61
Dietary vitamin E (mg/d)	5.8 ± 4.4	6.0 ± 3.0	6.3 ± 2.7	6.7 ± 2.7	7.6 ± 2.8
Folate (μg/d)	394 ± 246	381 ± 225	377 ± 220	377 ± 220	390 ± 241
Fiber (g/d)	16.3 ± 5.4	16.4 ± 4.8	16.3 ± 4.5	16.4 ± 4.6	16.6 ± 5.1
Vegetables (servings/d)[3]	3.0 ± 1.7	3.3 ± 1.8	3.6 ± 1.9	3.9 ± 2.0	4.2 ± 2.3

[1] MI, myocardial infarction.
[2] $\bar{x}$ ± SD.
[3] A composite score of 28 vegetable foods.

Downloaded from https://academic.oup.com/ajcn/article-abstract/69/5/890/4714835 by Northeastern University Libraries user on 23 May 2019

TABLE 3
Relative risks (RR) and 95% CIs of fatal ischemic heart disease and nonfatal myocardial infarction (MI) by quintiles of median energy-adjusted intakes of α-linolenic acid

	Quintiles of α-linolenic acid intake					
	1 (lowest, 0.71 g/d)	2 (0.86 g/d)	3 (0.98 g/d)	4 (1.12 g/d)	5 (highest, 1.36 g/d)	P for trend
Fatal CHD						
No. of cases	49	52	51	41	39	
Person-years	138 468	139 658	140 606	139 711	140 306	
Crude mortality rate (per 100 000 person-years)	35	37	36	29	28	
RR adjusted for age and smoking	1.0	1.07 (0.73, 1.58)	1.02 (0.69, 1.51)	0.78 (0.52, 1.19)	0.71 (0.47, 1.08)	0.03
Multivariate RR[1]	1.0	0.99 (0.66, 1.48)	0.90 (0.59, 1.39)	0.67 (0.42, 1.09)	0.55 (0.32, 0.94)	0.01
Nonfatal MI						
No. of cases	121	114	120	131	111	
Person-years	138 468	139 658	140 606	139 711	140 306	
Crude incidence rate (per 100 000 person-years)	87	82	85	94	79	
RR adjusted for age and smoking	1.0	0.95 (0.73, 1.22)	0.98 (0.76, 1.26)	1.05 (0.82, 1.35)	0.84 (0.64, 1.08)	0.29
Multivariate RR[1]	1.0	0.92 (0.71, 1.21)	0.94 (0.71, 1.25)	1.02 (0.76, 1.19)	0.85 (0.61, 1.19)	0.50

[1] 95% CIs in parentheses. Models include the following variables: age (5-y category), time period (7 periods), BMI (5 categories), cigarette smoking [never, past, and current smoking (1–14, 15–24, and ≥25 cigarettes/d)], history of hypertension, history of diabetes, history of hypercholesterolemia, menopausal status (premenopausal, postmenopausal without hormone replacement, postmenopausal with past hormone replacement, and postmenopausal with current hormone replacement), parental history of MI before 65 y of age, multiple vitamin use, vitamin E supplement use, alcohol consumption (4 categories), aspirin use (nonuser, 1–6/wk, ≥7/wk, and dose unknown), vigorous exercise ≥1 time/wk, and dietary intakes (in quintiles) of saturated fat, linoleic acid, vitamins C and E, and total energy.

α-Linolenic acid intake was positively associated with intakes of dietary vitamin E (r = 0.23) and vegetables (r = 0.22) and only slightly, but significantly correlated with intake of vitamin C (r = −0.04) and alcohol (r = −0.05).

Analyses of women with no prior IHD at baseline

After adjustment for age and smoking, energy-adjusted intake of α-linolenic acid was inversely associated with risk of fatal IHD; the RR for the highest compared with the lowest quintile was 0.71 (95% CI: 0.47, 1.08; P for trend = 0.03) (Table 2). This inverse association became stronger after adjustment for other coronary risk factors (RRs from the lowest to highest quintiles: 1.0, 0.99, 0.90, 0.67, and 0.55; 95% CI: 0.32, 0.94; P for trend = 0.01). Additional adjustment for vegetable intake did not materially change the result when extreme quintiles were compared (RR: 0.58; 95% CI: 0.33, 1.01; P for trend = 0.02).

α-Linolenic acid intake was only weakly associated with reduced risk of nonfatal MI (Table 3). The multivariate RR for extreme quintiles of intake was 0.85 (95% CI: 0.61, 1.19; P for trend = 0.50). The association for total IHD (combining nonfatal MI and fatal IHD) was intermediate between that for fatal IHD and nonfatal MI when extreme quartiles were compared (multivariate RR: 0.75; 95% CI: 0.56, 1.00; P for trend = 0.05). In the multivariate model for fatal IHD, further adjustment for intakes of fish n−3 fatty acids, oleic acid, trans fatty acids, cholesterol, folate, or fiber did not materially alter the association. This inverse association was similar for aspirin nonusers (RR for extreme quintiles of intake: 0.51; 95% CI: 0.24,1.09; P for trend = 0.08) and users (RR for extreme quintiles of intake: 0.60; 95% CI: 0.28,1.31; P for trend = 0.06). When intake of α-linolenic acid was expressed as a percentage of total energy intake rather than adjusted for total energy intake by regression analysis, there was a similar inverse association [RRs for fatal IHD from the lowest to the highest quintiles: 1.0 (reference group), 0.89 (95% CI: 0.59, 1.34), 0.90 (0.59, 1.38), 0.66 (0.41, 1.07), and 0.52 (0.30, 0.90); P for trend = 0.01]. The analyses limited to α-linolenic acid from plant sources also yielded similar results [RRs for fatal IHD by quintiles of α-linolenic acid intake: 1.0 (reference group), 1.17 (95% CI: 0.80, 1.74), 0.83 (0.52, 1.30), 0.55 (0.33, 0.94), and 0.64 (0.36, 1.13); P for trend = 0.03].

We conducted several alternative analyses of the relation of α-linolenic acid intake to fatal IHD. The multivariate RRs for fatal IHD when updated dietary information was used were 1.00, 0.96, 1.02, 0.71, and 0.57 (95% CI: 0.33, 0.98) from the lowest to highest quintiles of α-linolenic acid intake (P for trend = 0.02). When we excluded women who had diabetes or hypercholesterolemia at baseline, the RRs for fatal IHD by quintiles of α-linolenic acid intake were 1.0, 0.93, 0.80, 0.58, and 0.55 (95% CI: 0.29, 1.07; P for trend = 0.04). Exclusion of IHD cases that occurred in the first 4 y of follow-up to avoid potential change in diet due to preclinical conditions did not change the result.

We also examined the association between the ratio of α-linolenic acid to linoleic acid and fatal IHD risk because α-linolenic acid metabolism could be inhibited by high concentrations of linoleic acid (14). The median values for quintiles of the ratio were 0.07, 0.09, 0.10, 0.11, and 0.14. The multivariate RRs for quintiles of the ratio were 1.0, 1.02, 0.84, 0.87, and 0.84 (95% CI: 0.53, 1.33; P for trend = 0.40).

Stratified analyses

When the relation between α-linolenic acid and fatal IHD was examined by use of vitamin E supplements (Figure 1), we observed inverse associations among both users and nonusers (P for interaction = 0.44), but a slightly stronger association was seen among supplement users. When examined within categories of trans fatty acid intake, inverse associations between α-linolenic acid and fatal IHD were observed among women with higher and lower intakes of trans fatty acids (P for interaction = 0.54), but the relation was somewhat stronger among

Downloaded from https://academic.oup.com/ajcn/article-abstract/69/5/890/4714835 by Northeastern University Libraries user on 23 May 2019

those with lower intakes of trans fatty acids (Figure 2). The association between α-linolenic acid and fatal IHD risk did not vary appreciably with intakes of linoleic acid or n−3 fatty acids from fish. In addition, the results were similar by smoking status (current smokers versus nonsmokers) and alcohol drinking status (current drinkers versus nondrinkers).

Analyses of women with a prior MI

We examined whether a higher α-linolenic acid intake could reduce the risk of fatal IHD among women with a prior MI. After adjustment for age, the RRs for fatal IHD across quintiles of α-linolenic acid intake were 1.0 (reference group), 0.57 (95% CI: 0.28,1.15), 0.77 (0.41,1.45), 0.48 (0.23, 0.99), and 0.51 (0.25, 1.06); the P for trend was 0.06. After adjustment for nondietary and dietary confounding variables, the RRs were 1.0 (reference group), 0.61 (95% CI: 0.28, 1.30), 0.90 (0.41, 1.97), 0.61 (0.24, 1.52), and 0.35 (0.12, 1.01); the P for trend was 0.07.

Food analyses

To identify the foods that contributed most to differences in α-linolenic acid intake among study participants, we used stepwise regression with energy-adjusted α-linolenic acid as the dependent variable and all foods as predictor variables. The major determinants of α-linolenic acid were oil and vinegar salad dressing and mayonnaise or other creamy salad dressings (total r^2 = 0.50). We then examined the relation of these salad dressings to risk of fatal IHD (Table 4). For both foods, we had to combine adjacent categories of responses in the original questions to provide a sufficient number of women in each group. Greater intake of oil and vinegar salad dressing was associated with a reduced risk of fatal IHD (multivariate RR for the comparison of women who consumed the dressing ≥5–6 times/wk and those who consumed it <1 time/mo: 0.46 (95% CI: 0.27, 0.76; P for trend = 0.001). A higher intake of mayonnaise or other creamy salad dressing was associated with a nonsignificant lower risk of fatal IHD [multivariate RR for the comparison of women who consumed the dressing ≥5–6 times/wk and those who consumed it <1 time/mo: 0.84 (95% CI: 0.50, 1.44; P for trend = 0.44)]. Additional adjustment for vegetable intake did not materially alter the results.

DISCUSSION

In this prospective study we found a significant inverse association between dietary intake of α-linolenic acid and risk of fatal IHD. The risk was further lowered among women who also took vitamin E supplements or who had a lower intake of trans fatty acids. In addition, among women with prevalent MI, we observed a trend toward lower risk of fatal IHD for those with a higher intake of α-linolenic acid.

Inaccurate assessment of α-linolenic acid using a food-frequency questionnaire is a potential concern. However, the calculated intakes of linolenic acid, linoleic acid, and trans fatty acids were reasonably correlated with amounts of these fatty acids in adipose tissue (6), suggesting the validity of our instrument in measuring diet. In addition, we assessed the dietary intake of α-linolenic acid and other nutrients 3 times during the study period, so that analyses using updated dietary information accommodate changes in dietary habits within individuals and in food composition. Confidence in the validity of our findings increased because similar results were obtained when both baseline data and updated dietary information were used. Women with a higher intake of α-linolenic acid did not appreciably differ from those with a lower intake for most of the known IHD risk factors, including age, BMI, smoking, parental history of MI, postmenopausal hormone use, and history of hypertension, diabetes, and hypercholesterolemia. This finding suggests that residual confounding by these variables was unlikely to explain our findings. Women with a higher intake of α-linolenic acid were more likely to have a higher consumption of other dietary fats, especially linoleic acid. However, adjustment for other fats actually somewhat strengthened the association. Because salad dressings are major sources of

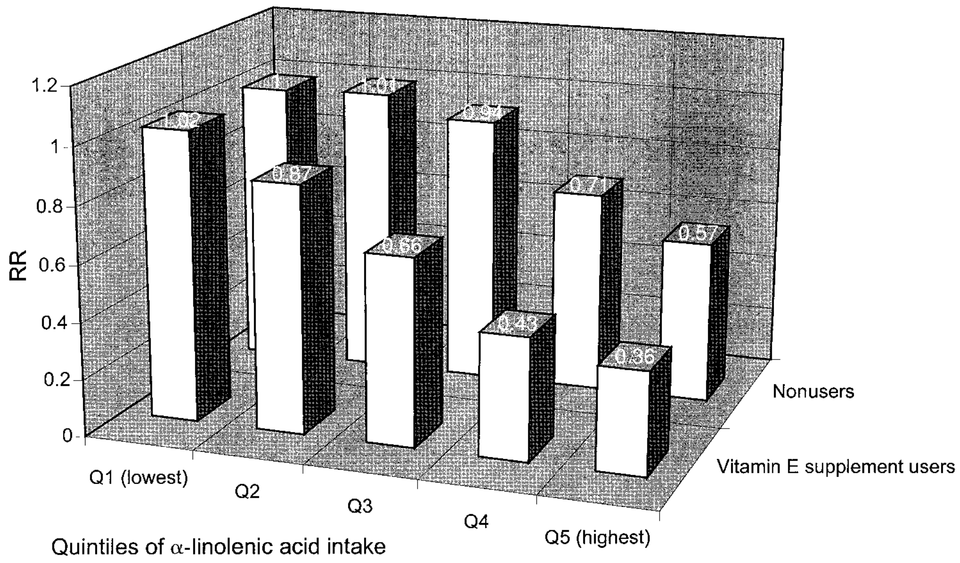

FIGURE 1. Multivariate relative risk (RR) of fatal ischemic heart disease by quintiles of α-linolenic acid intake and vitamin E supplement use in women from the Nurses' Health Study cohort. The model included the same variables as in Table 3. The reference group included women who were in the lowest quintile of α-linolenic acid intake and nonusers of vitamin E supplements.

Downloaded from https://academic.oup.com/ajcn/article-abstract/69/5/890/4714835 by Northeastern University Libraries user on 23 May 2019

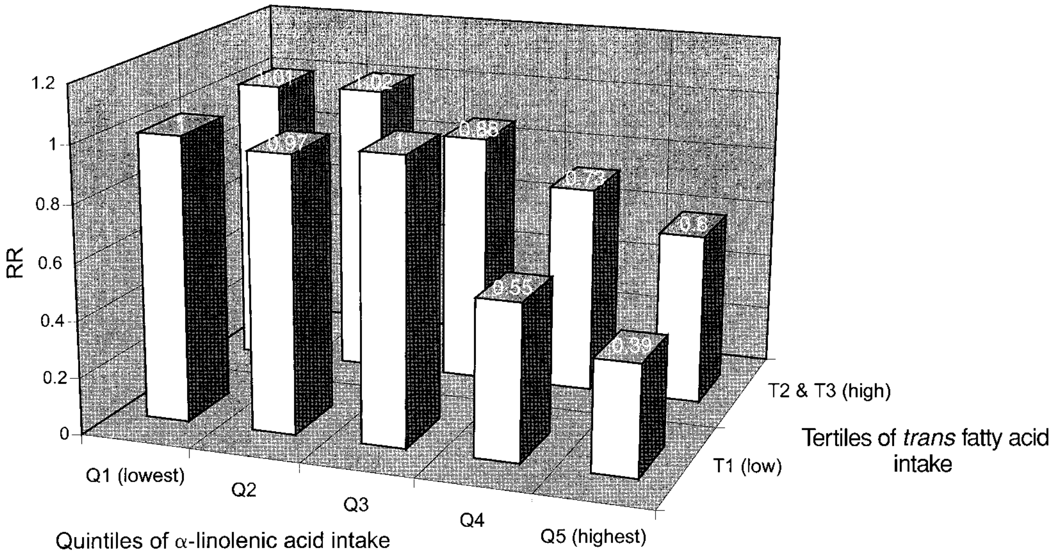

FIGURE 2. Multivariate relative risk (RR) of fatal ischemic heart disease by quintiles of α-linolenic acid and tertiles (T) of trans fatty acid intakes in women from the Nurses' Health Study cohort. The model included the same variables as in Table 3. The reference group included women who were in the lowest quintile of α-linolenic acid intake and the lowest tertile of trans fatty acid intake.

α-linolenic acid, α-linolenic acid may be a proxy for vegetable intake, but adjustment for vegetable intake did not alter the results.

In support of an inverse association between α-linolenic acid intake and risk of fatal IHD, we found that specific foods that are primary dietary sources of α-linolenic acid were associated with reduced risk. In particular, we found a significantly lower risk of fatal IHD among women who consumed oil and vinegar salad dressing more frequently (≥5–6 times/wk) compared with those who rarely consumed it. We also observed a nonsignificant inverse association between intake of mayonnaise salad dressing and fatal IHD. Both oil and vinegar and mayonnaise salad dressings are commonly made from unhydrogenated soybean oil, which contains ≈7% α-linolenic acid. However, the observed inverse associations cannot be attributed to α-linolenic acid alone because these salad dressings also contain linoleic acid and other potential beneficial nutrients. Nevertheless, these data raise concern about widespread use of fat-free salad dressings, which has eliminated an important source α-linolenic acid and linoleic acid in the US diet.

α-Linolenic acid may contribute to a reduced risk of fatal IHD through its antiarrhythmic effect. In cell culture studies, α-linolenic acid was shown to slow the beating rate of isolated neonatal rat cardiac myocytes (15). Also, significant reductions in cardiac arrhythmia were observed in rats fed red meat supplemented with fish oil or canola oil (8% α-linolenic acid) when compared with rats fed only red meat or red meat supplemented with sheep fat (2). In another experiment, mortality due to ventricular fibrillation was significantly lower in rats fed a diet containing canola oil (0%) than in rats fed olive oil (30%) (1). In both exper-

TABLE 4
Relative risks (RR) and 95% CIs of fatal ischemic heart disease by intake of specific foods that are main sources of α-linolenic acid

	Categories of intake				
	<1 time/mo	1–4 times/mo	2–4 times/wk	≥5–6 times/wk	P for trend
Oil and vinegar salad dressing (15 mL)					
No. of cases	84	92	38	18	
Person-years	182719	265105	147431	103494	
Crude mortality rate (per 100000 person-years)	46	35	26	17	
RR adjusted for age and smoking	1.0	0.83 (0.62, 1.11)	0.60 (0.41, 0.87)	0.37 (0.22, 0.62)	<0.001
Multivariate RR[1]	1.0	0.92 (0.68, 1.24)	0.68 (0.46, 1.01)	0.46 (0.27, 0.76)	0.001
Mayonnaise or other creamy salad dressing (15 mL)					
No. of cases	28	108	66	30	
Person-years	72935	320963	210238	94614	
Crude mortality rate (per 100000 person-years)	38	34	31	32	
RR adjusted for age and smoking	1.0	0.96 (0.63, 1.46)	0.88 (0.56, 1.36)	0.84 (0.50, 1.42)	0.37
Multivariate RR[1]	1.0	0.97 (0.64, 1.48)	0.91 (0.58, 1.43)	0.84 (0.50, 1.44)	0.44

[1]95% CIs in parentheses. Models include the following variables: age (5-y category), time period (7 periods), BMI (5 categories), cigarette smoking [never, past, and current smoking (1–14, 15–24, and ≥25 cigarettes/d)], history of hypertension, history of diabetes, history of hypercholesterolemia, menopausal status (premenopausal, postmenopausal without hormone replacement, postmenopausal with past hormone replacement, and postmenopausal with current hormone replacement), parental history of myocardial infarction before 65 y of age, multiple vitamin use, vitamin E supplement use, alcohol consumption (4 categories), aspirin use (nonuser, 1–6/wk, ≥7/wk, and dose unknown), vigorous exercise ≥1 time/wk, and dietary intakes (in quintiles) of saturated fat, linoleic acid, vitamins C and E, and total energy.

Downloaded from https://academic.oup.com/ajcn/article-abstract/69/5/890/4714835 by Northeastern University Libraries user on 23 May 2019

iments, arrhythmias were induced by coronary occlusion and reperfusion. In a secondary prevention trial, cardiovascular deaths were significantly lower among patients randomly assigned to a Mediterranean diet enriched with α-linolenic acid than in those in the control group, although other dietary changes occurred simultaneously in the experiment (3). In both animals and humans, α-linolenic acid can be metabolized to long-chain polyunsaturated n−3 fatty acids, including eicosapentaenoic (20:5n−3) and docosahexaenoic (22:6n−3) acids even though the efficiency of this conversion is still a matter of debate (16). Previous studies have suggested that higher intakes of long-chain n−3 fatty acids from fish may reduce the risk of fatal arrhythmias and sudden death in humans (17, 18). Our observations that α-linolenic acid were primarily associated with reduced risk of fatal IHD and that higher α-linolenic acid intake reduced the risk of death among prevalent MI patients are consistent with the antiarrhythmic effect of α-linolenic acid. Our results are also consistent with those of prospective studies in men in which the apparent protective effects of α-linolenic acid were primarily seen for fatal IHD (19–21).

Besides its antiarrhythmic effect, α-linolenic acid may reduce coronary risk through its antithrombotic effect (22). α-Linolenic acid and its metabolite, eicosapentaenoic acid, can decrease generation of thromboxane A_2, a proaggregatory vasoconstrictor, through their inhibitory action on the conversion from linoleic acid to arachidonic acid and the enzyme cyclooxygenase (15, 23–25). However, the similarity of the inverse association between α-linolenic acid intake and risk of fatal IHD among aspirin users and nonusers suggests that the effect of α-linolenic acid is probably due to its antiarrhythmic rather than its antithrombotic properties; otherwise, the effect of α-linolenic acid would be masked by aspirin use.

It is thought that dietary α-linolenic acid may be essential at 0.5% of energy intake (26). On the basis of food disappearance data, Hunter (27) estimated the average dietary intake of α-linolenic acid in the United States in 1985 to be ≈1.2 g/d, or 0.5% of the energy intake, which is similar to the mean daily intakes estimated in the present study (1.1 g/d) and in the Health Professional Follow-up Study (1.1 g/d) (20). These estimates suggest that perhaps half the US population may not be meeting the requirement for α-linolenic acid (16). In addition, the optimal balance between dietary α-linolenic acid and linoleic acid is not clear (1, 14). In this study, we found that the ratio was less strongly related to risk of fatal IHD than was α-linolenic acid alone because both fatty acids were inversely related to the risk.

We observed a somewhat stronger effect of α-linolenic acid among vitamin E supplement users, suggesting that the availability of antioxidants such as vitamin E may be important for the biologic effect of α-linolenic acid. In addition, because of its susceptibility to oxidation, α-linolenic acid–rich oils are frequently hydrogenated during processing, converting unsaturated fatty acids to trans fatty acids. Substantial evidence suggests that trans fatty acids have adverse effects on coronary disease (13, 20, 21, 28). In metabolic studies, trans fatty acids raise LDL and lower HDL in humans (29). Moreover, trans isomers may interfere with biological functions of essential fatty acids by competing with linoleic and α-linolenic acid for $\Delta^{6/5}$-desaturase (30). In this study, we found that the reduction in fatal IHD risk associated with α-linolenic acid was somewhat attenuated among women with a higher intake of trans fatty acids.

In conclusion, this study provides support for the hypothesis that a higher intake of α-linolenic acid will reduce the risk of fatal IHD. Our findings suggest that a reduction in consumption of foods such as oil-based salad dressings that contain polyunsaturated fats, including α-linolenic acid, may increase the risk of fatal IHD.

We are indebted to the participants in the Nurses' Health Study for their continuing outstanding level of cooperation; to Al Wing, Mark Shneyder, Stefanie Parker, Gary Chase, Karen Corsano, Lisa Dunn, Barbara Egan, Lori Ward, and Jill Arnold for their unfailing help; and to Frank E Speizer, Principal Investigator of the Nurses' Health Study, for his support.

REFERENCES

1. McLennan PL, Dallimore JA. Dietary canola oil modifies myocardial fatty acids and inhibits cardiac arrhythmias in rats. J Nutr 1995;125:1003–9.
2. Siebert BD, McLennan PL, Woodhouse JA, Charnock JS. Cardiac arrhythmia in rats in response to dietary n−3 fatty acids from red meat, fish oil and canola oil. Nutr Res 1993;13:1407–18.
3. de Lorgeril M, Renaud S, Mamelle N, et al. Mediterranean α-linolenic acid-rich diet in secondary prevention of coronary heart disease. Lancet 1994;343:1454–9.
4. Hill EG, Johnson SB, Lawson LD, Mahfouz MM, Holman RT. Perturbation of the metabolism of essential fatty acids by dietary hydrogenated vegetable oil. Proc Natl Acad Sci U S A 1982;79:953–7.
5. Willett WC, Sampson L, Browne ML, et al. The use of a self-administered questionnaire to assess diet four years in the past. Am J Epidemiol 1988;127:188–99.
6. Garland M, Sacks FM, Colditz GA, et al. The relation between dietary intake and adipose tissue composition of selected fatty acids in US women. Am J Clin Nutr 1998;67:25–30.
7. US Department of Agriculture. Composition of foods: raw, processed, and prepared, 1963–1988. Agriculture handbook no. 8. Washington, DC: US Government Printing Office, 1989.
8. Nettleton JA. Omega-3 fatty acids: comparison of plant and seafood sources in human nutrition. J Am Diet Assoc 1991;91:331–7.
9. Stampfer MJ, Willett WC, Speizer FE, et al. Test of the National Death Index. Am J Epidemiol 1984;119:837–9.
10. Rose GA, Blackburn H. Cardiovascular survey methods. World Health Organ Monogr Ser 1982;58.
11. Willett WC. Nutritional epidemiology. New York: Oxford University Press, 1990.
12. D'Agostino RB, Lee M-L, Belanger AJ, Cupples LA, Anderson K, Kannel WB. Relation of pooled logistic regression to time dependent Cox regression analysis: The Framingham Heart Study. Stat Med 1990;9:1501–15.
13. Hu FB, Stampfer MJ, Manson JE, et al. Dietary fat intake and risk of coronary heart disease in women. N Engl J Med 1997;337:1491–9.
14. Chan JK, McDonald BE, Gerrard JM, Bruce VM, Weaver BJ, Holub BJ. Effect of dietary α-linolenic acid and its ratio to linoleic acid on platelet and plasma fatty acids and thrombogenesis. Lipids 1993;28:811–7.
15. Kang JX, Leaf A. Antiarrhythmic effects of polyunsaturated fatty acids: recent studies. Circulation 1996;94:1774–80.
16. Cunnane SC. Metabolism and function of α-linolenic acid in humans. In: Cunnane SC, Thompson LU, eds. Flaxseed in human nutrition. Champaign, IL: AOAC Press, 1995:99–127.
17. Siscovick DS, Raghunathan TE, King I, et al. Dietary intake and cell membrane levels of long-chain n−3 polyunsaturated fatty acids and the risk of primary cardiac arrest. JAMA 1995;274:1364–7.
18. Albert CM, Hennekens CH, O'Donnell CJ, et al. Fish consumption and risk of sudden cardiac death. JAMA 1998;279:23–8.
19. Dolecek TA. Epidemiologic evidence of relationships between dietary polyunsaturated fatty acids and mortality in the Multiple Risk Factor Intervention Trial. Proc Soc Exp Biol Med 1992;200:177–82.
20. Ascherio A, Rimm EB, Giovannucci EL, Spiegelman D, Stampfer MJ, Willett WC. Dietary fat and risk of coronary heart disease in men: cohort follow up study in the United States. BMJ 1996;313:84–90.

Downloaded from https://academic.oup.com/ajcn/article-abstract/69/5/890/4714835 by Northeastern University Libraries user on 23 May 2019

21. Pietinen P, Ascherio A, Korhonen P, et al. Intake of fatty acids and risk of coronary heart disease in a cohort of Finnish men: The Alpha-Tocopherol, Beta-Carotene Cancer Prevention Study. Am J Epidemiol 1997;145:876–87.
22. Owren PA. Coronary thrombosis. Its mechanism and possible prevention by linolenic acid. Ann Intern Med 1965;63:167–84.
23. Kinsella JE. Effects of polyunsaturated fatty acids on factors related to cardiovascular disease. Am J Cardiol 1987;60:23G–32G.
24. Nair SSD, Leitch JW, Falconer J, Garg ML. Prevention of cardiac arrhythmia by dietary (n−3) polyunsaturated fatty acids and their mechanism of action. J Nutr 1997;127:383–93.
25. Renaud S, Nordoy A. "Small is beautiful": α-linolenic acid and eicosapentaenoic acid in man. Lancet 1983;1:1169 (letter).
26. Kinsella JE. Food lipids and fatty acids: importance in food quality, nutrition, and health. Food Technol 1988;42:124–45.
27. Hunter JE. n−3 Fatty acids from vegetable oils. Am J Clin Nutr 1990;51:809–14.
28. Willett WC, Stampfer MJ, Manson JE, et al. trans-Fatty acid intake in relation to risk of coronary heart disease among women. Lancet 1993;341:581–5.
29. Mensink RPM, Katan MB. Effect of dietary trans fatty acids on high-density and low-density lipoprotein cholesterol levels in healthy subjects. N Engl J Med 1990;323:439–45.
30. Kinsella JE, Bruckner G, Mai J, Shimp J. Metabolism of trans fatty acids with emphasis on the effects of trans, trans-octadecadienoate on lipid composition, essential fatty acid, and prostaglandins: an overview. Am J Clin Nutr 1981;34:2307–18.

Downloaded from https://academic.oup.com/ajcn/article-abstract/69/5/890/4714835 by Northeastern University Libraries user on 23 May 2019
