# Supplementary material for: A systematic comprehensive longitudinal evaluation of dietary factors associated with acute myocardial infarction and fatal coronary heart disease
Source: Nat Commun. 2020 Nov 27;11:6074. doi: 10.1038/s41467-020-19888-2 (PMC7699643; doi:10.1038/s41467-020-19888-2)
Supplement: Supplementary file 4 — Supplementary Software [file 41467_2020_19888_MOESM4_ESM.zip › EWAS-NHS-master/KG-SI/Rest/Jimenez (2012).pdf]

NIH Public Access
Author Manuscript
Stroke. Author manuscript; available in PMC 2013 April 01.

Published in final edited form as:
Stroke. 2012 April ; 43(4): 939–945. doi:10.1161/STROKEAHA.111.639435.

Alcohol Consumption and Risk of Stroke in Women

Monik Jimenez, ScD[1], **Stephanie E. Chiuve, ScD**[1,2], **Robert J. Glynn, PhD, ScD**[1], **Meir J. Stampfer, MD, DrPH**[2,3,4], **Carlos A. Camargo Jr., MD, DrPH**[3,4], **Walter C. Willett, MD, DrPH**[2,3,4], **JoAnn E. Manson, MD, DrPH**[1,3,4], and **Kathryn M. Rexrode, MD, MPH**[1]

[1]Division of Preventive Medicine, Brigham and Women's Hospital

[2]Department of Nutrition, Harvard School of Public Health

[3]Channing Laboratory, Department of Medicine, Brigham and Women's Hospital

[4]Department of Epidemiology, Harvard School of Public Health

[5]Department of Biostatistics, Harvard School of Public Health

Abstract

Background and Purpose—Light-to-moderate alcohol consumption has been consistently associated with lower risk of heart disease, but data for stroke are less certain. A lower risk of stroke with light-to-moderate alcohol intake has been suggested but the dose response among women remains uncertain and the data in this subgroup have been sparse.

Methods—A total of 83,578 female participants of the Nurses' Health Study who were free of diagnosed cardiovascular disease and cancer at baseline, were followed from 1980–2006. Data on self-reported alcohol consumption were assessed at baseline and updated approximately every 4 years, while stroke and potential confounder data were updated at baseline and biennially. Strokes were classified according to the National Survey of Stroke criteria.

Results—We observed 2,171 incident strokes over 1,695,324 person-years. In multivariable adjusted analyses, compared to abstainers, the relative risks of stroke were RR=0.83 (95% CI 0.75–0.92) for <5 g/day, RR=0.79 (95% CI: 0.70–0.90) for 5–14.9 g/day, RR=0.87 (0.72–1.05) for 15–29.9 g/day and RR=1.06 (95% CI=0.86–1.30) for 30–45 g/day. Results were similar for ischemic and hemorrhagic stroke.

Conclusions—Light-to-moderate alcohol consumption was associated with a lower risk of total stroke. In this population of women with modest alcohol consumption, an elevated risk of total stroke related to alcohol was not observed.

Keywords

Risk factors for Stroke; alcohol; ischemic stroke; subarachnoid hemorrhage

Introduction

The association between alcohol consumption and risk of stroke remains debated with regard to associations by dose, sex and stroke type. Some studies have suggested a lower risk of stroke among those with light-to-moderate alcohol intake, and a possibly greater risk at higher levels, but the inflection point is uncertain and the shape of the dose response may

Corresponding Author: Kathryn M. Rexrode, MD, MPH, 900 Commonwealth Ave, 3rd Floor, Boston, MA 02215, Telephone: 617-278-0834, Fax: 617-232-3541, krexrode@partners.org.

Disclosures: None

NIH-PA Author Manuscript
NIH-PA Author Manuscript
NIH-PA Author Manuscript

NIH-PA Author Manuscript NIH-PA Author Manuscript NIH-PA Author Manuscript

vary by gender. A meta-analysis by Reynolds et al.[1] observed a greater risk reduction for stroke associated with low-to-moderate alcohol consumption among women than men; however, prospective cohort data were limited, especially at higher levels of intake. Further, the association may differ by stroke type and the shape of the dose response may vary by gender within types. For example, a linear association between alcohol consumption and hemorrhagic stroke has been suggested but may characterize the association only among men; more data are needed.[2] Furthermore, consumption patterns vary substantially between genders; only 42% of women reported drinking ≥12 drinks in the past year compared to 60% of men.[3]

Most studies have utilized only baseline assessments of alcohol consumption, which fail to account for changes in alcohol consumption over time. Moreover, studies to date have not evaluated potential effect modification by key risk factors such as age or hormone therapy use in women. Finally, few studies have adequately adjusted for confounding by lifestyle and/or socioeconomic factors and have been limited by few events.

In our prior work from this cohort Chiuve et al.[4] observed a significantly lower risk of stroke for alcohol consumption <15 gm/day compared to abstainers; however, this was not the main focus of the analysis. Therefore, we have analyzed the association between alcohol intake and risk of total, ischemic and hemorrhagic stroke in more detail with special consideration of factors specific to alcohol consumption, such as a detailed analysis of former drinkers and lifetime abstainers using updated information on alcohol intake over time. Importantly, we evaluated whether these associations varied by age, hormone use, aspirin use, hypertension, smoking status and history of atrial fibrillation.

Materials and Methods

Study population

The Nurses' Health Study (NHS) enrolled 121,700 female registered nurses living in 11 US states, 30–55 years who completed a mailed questionnaire in 1976. Follow-up questionnaires are mailed biennially, with a semi-quantitative food frequency questionnaire (FFQ) mailed approximately every 4 years since 1980. Detailed descriptions of the NHS have been previously published.[5]

Women were excluded from the analysis if they were abstainers at baseline in 1980 and reported "greatly decreasing" their alcohol intake in the previous 10 yrs (potentially "sick quitters" n=3,120), if alcohol data at baseline was missing, consumed >45 g/day (approximately 1% of population; 31 events), if they reported a history of stroke (n=268), cancer (except non-melanoma skin cancer; n=3,394), cardiovascular disease (CVD: myocardial infarction (MI), coronary artery bypass graft surgery (CABG) and angioplasty (PTCA; n=597), or if they had missing date of birth. The final sample consisted of 83,578 women.

Alcohol and Diet Assessment

In 1980, 1984, 1986 and every 4 years thereafter participants were asked to complete a FFQ on their intake of specific foods and beverages, each with specified portion size, during the previous 12 months. Separate questions asked about the consumption of wine (4oz [120ml] until 2004, 5oz [148ml] thereafter), beer (12oz [360ml]), and spirits (1.5oz (44ml)).[6] Beer was assumed to contain 13g (regular: 13g, light: 11g), wine 11g (4oz: 11g, 5oz: 13.6g) and spirits 14g of alcohol/serving. Total alcohol was calculated as the sum of all three beverage types and categorized (0, >0–4.9, 5–14.9, 15–29.9, 30–45 g/day), to maintain consistency with the literature.[7] Former drinkers were defined using questions on patterns of consumption collected in 1988, 1996, 2000 and 2004 (data supplement). Alcohol

NIH-PA Author Manuscript NIH-PA Author Manuscript NIH-PA Author Manuscript

consumption obtained on two FFQs were reproduced against four one-week diet records taken 3-months apart with correlation coefficients ranging from 0.86–0.90.[8] Furthermore, reported alcohol was significantly correlated with serum high density lipoprotein levels (HDL) (r=0.40, p-value<0.001).[8]

Cerebrovascular Disease Assessment

These analyses included all nonfatal and fatal strokes diagnosed after the return of the 1980 questionnaire but before June 2006. Women (or next-of-kin for decedents) reporting stroke on follow-up questionnaires were asked for permission to review medical records, which were reviewed by a physician blinded to exposure status. We excluded cerebrovascular pathology due to infection, trauma, malignancy and "silent" strokes discovered only by radiologic imaging. Stroke was classified according the National Survey of Stroke[9] criteria requiring evidence of a neurologic deficit with sudden or rapid onset that persisted for >24 hours or until death. Strokes were classified as ischemic stroke (thrombotic or embolic occlusion of a cerebral artery), hemorrhagic stroke (subarachnoid and intraparenchymal hemorrhage) or stroke of probable/unknown subtype when a stroke was documented but the subtype could not be ascertained due to unobtainable medical records. We classified stroke as probable if the supporting information was provided, but medical records were not available or only a death certificate was available. Seventy-six percent of total strokes were confirmed by medical records and/or death certificate documentation (medical records: n=1556; death certificate: n=106), while 27% were probable of unspecified subtype due to unavailable medical record documentation (n=596). The primary endpoints for this study were total (ischemic, hemorrhagic and strokes of probable/unknown subtype), ischemic and hemorrhagic stroke. Deaths were detected through information provided by the next of kin, postal authorities or by systematic searches of the National Death Index. Classification of fatal stroke was confirmed by review of hospital records or autopsy. Analyses were repeated excluding probable events and results were similar; therefore the analyses presented include both confirmed and probable events.

Statistical Analysis

Descriptive analyses for baseline characteristics were conducted for the full cohort and separately by categories of alcohol intake. Age and multivariable adjusted time-varying Cox models were used to estimate hazard ratios (HR) and corresponding 95% confidence intervals (CI) for the association between alcohol consumption and risk of stroke using age (months) as the underlying time scale stratified by calendar year (questionnaire cycle). Participants contributed follow-up from the date of return of the 1980 questionnaire until the earliest of the following: death, stroke or June 2006.

Data on all covariates were collected on each biennial questionnaire, with the exception of height (collected in 1976), atrial fibrillation (collected beginning in 2000), education (collected in 1992), marital status (1992, 1996, and 2000) and dietary variables (collected every 4 years). All models adjusted for smoking. Multivariable models additionally adjusted for physical activity, body mass index (BMI kg/m^2), family history of heart disease, history of heart disease, diabetes and hypertension, bilateral oophorectomy, postmenopausal status, hormone therapy, high cholesterol, multivitamin intake, aspirin, composite 6 nutrient diet score,[10] highest level of education, husband's highest level of education, and marital status (data supplement). To address missing information, data were carried forward <1 questionnaire cycle, and the missing indicator method was used to model missing values in categorical variables.

In primary analyses, we modeled alcohol consumption utilizing the most recent alcohol intake, based on acute effects on blood pressure, platelets and thrombotic factors. In

NIH-PA Author Manuscript

NIH-PA Author Manuscript

NIH-PA Author Manuscript

secondary analyses, we modeled the cumulative average of alcohol consumption over follow-up which assumes long-term exposure to alcohol as the biologic mechanism.[11] *A priori* we proposed to evaluate effect modification by selected risk factors, age, hypertension status, aspirin use, smoking status, hormone therapy, and atrial fibrillation. History of atrial fibrillation was ascertained beginning in 2000, hence this analysis utilized a subgroup (n=51,846). Effect modification was assessed by a likelihood ratio test, comparing a model including interaction terms to a model with main effects only.

We examined a non-linear relation between alcohol consumption and incident total stroke non-parametrically with restricted cubic splines based on 3 knots of alcohol consumption,[12] located at the 5th, 50th and 95th percentiles.[13] Tests for non-linearity used the likelihood ratio test, comparing the model with only the linear term to the model with the linear and the cubic spline terms. In sensitivity analyses, we stopped updating alcohol exposure upon self-report of hypertension, diabetes, MI, CABG, PTCA or cancer to assess potential bias due to change in alcohol consumption as a consequence of diagnosis. We conducted a separate analysis categorizing non-drinkers separately as former drinkers or abstainers. Additionally, we evaluated an alternate upper category of alcohol consumption (>45 g/day, N=84,611; total stroke=2,202). We also evaluated whether the association differed according to alcohol type. Residual confounding by smoking status was examined by subgroup analysis among never smokers. All p-values are two-sided. Analyses were conducted with SAS for UNIX statistical software (version 9.1.3; SAS Institute, Cary, NC). This study was approved by the Institutional Review Board of Brigham and Women's Hospital and all procedures followed were in accordance with institutional guidelines. Participants provided informed consent to participate.

Results

The baseline characteristics of the study population by categories of alcohol consumption are shown in Table 1. The mean age at baseline was 46 years. Approximately 30% of women reported no alcohol consumption, 35% reported very low levels of intake (approx. ≤½ a glass/day) and 4% reported 30–45 g/day (approximately 2–3 glasses/day). Overall, heavier alcohol consumption was associated with a higher prevalence of current smoking, history of hypertension, increased physical activity and lower BMI compared to abstainers.

Over 26 years of follow-up we documented 2,171 total stroke events, 1,206 of which were ischemic, 363 hemorrhagic, and 602 of probable/unknown subtype. In multivariable analyses, low (>0–4.9 g/day) and moderate (5–14.9 g/day) consumption were associated with lower risk of total stroke compared to abstainers (Table 2), whereas women who consumed 30–45 g/day did not have a greater risk of total stroke. Figure 1, shows the non-linear J-shaped association between alcohol consumption and risk of incident total stroke (p<0.001 for deviation from linearity). Greater risk of stroke was observed for alcohol intake >36 g/day, however, the confidence limits were exceptionally wide due to few events in the higher range of intake. In sensitivity analyses, former drinkers did not exhibit an elevated risk compared to lifetime abstainers (data not shown).

In multivariable analyses, the associations between alcohol consumption and ischemic stroke were similar, but not statistically significant. Intake of 30–45 g/day was suggestive of a non-significant increased risk of ischemic stroke. There was no association between alcohol consumption (30–45 g/day) and risk of hemorrhagic stroke when compared to abstainers.

Stratified analyses by key risk factors were performed (Table 3). There was no evidence to suggest that the association varied significantly by age, hypertension, aspirin use, hormone

NIH-PA Author Manuscript NIH-PA Author Manuscript NIH-PA Author Manuscript

therapy, or smoking. However, there was a suggestion that the association varied by history of atrial fibrillation, with a significantly lower risk of total stroke among moderate drinkers (>0–29.9 g/d) without a history of atrial fibrillation (p-value for interaction=0.03).

In sensitivity analyses, we also modeled alcohol intake using a cumulative method and obtained similar results as our main analysis using recent intake. We additionally examined an alternate upper category of >45 g/day, which utilized more observations and results were similar to those of our main analysis despite limited power (>45 g/day: HR=1.03; 95% CI: 0.71–1.48). We considered a multivariable model without adjustment for hypertension, since it may be part of the causal pathway, but results were materially unchanged (data not shown). Analyses by alcohol type provided similar results (results not shown). To assess a potential bias due to changes in alcohol consumption as a consequence of chronic disease diagnosis, we stopped updating alcohol exposure upon self-reports of chronic disease and observed similar results (data not shown). Lastly, there was no evidence of residual confounding by smoking, shown by similar results among never smokers (Table 3).

Discussion

In this population of nearly 85,000 women who were free of reported CVD at baseline, we observed an inverse association between low-to-moderate alcohol consumption and risk of total stroke. We observed a lower risk at low-to-moderate intakes and a suggestion towards greater risk of ischemic stroke at intakes of 30–45 g/d. Low-to-moderate alcohol consumption was not associated with a greater risk of hemorrhagic stroke in this population. We had limited power to evaluate heavy drinking; only 1% of women reported consuming >45 g/day.

Our results are generally consistent with previous studies.[1,4] Reynolds et al.[1] reported 20–30% lower risk of total stroke for men and women at low levels of intake and a nearly fourfold greater risk at the highest level (≥60 g/day) compared to abstainers (p<0.001 nonlinear trend) which was based on 35 studies; however, only 16 were among women. In the Framingham Heart Study,[14] one of the few studies with multiple assessments and lengthy follow-up, there was no clear association between alcohol consumption and risk of ischemic stroke among women. Overall, the data indicate a modestly lower risk of ischemic stroke for low-to-moderate alcohol consumption with a potentially greater risk at levels beyond the range observed in our study.

Data for hemorrhagic stroke have been inconsistent; some suggesting a linearly increasing association[1] and others suggesting increased risk only at higher levels of intake.[2] Patra et al.[2] reported a J-shaped association between alcohol consumption and hemorrhagic stroke among women, with an inverse association for ≤36 g/d, in contrast to a linear association among men; however the data among women were based on a mere 8 studies. Our results are similar, but underpowered at high levels of consumption.

We conducted several *a priori* stratified analyses and observed significant effect modification by atrial fibrillation. Moderate alcohol consumption was only associated with a lower risk of total stroke compared to abstention among women without a history of atrial fibrillation. Alcohol may increase risk of atrial fibrillation leading to greater risk of atrial clot formation and embolic stroke;[15] however, these analyses were likely underpowered. Since alcohol has been shown to increase serum estrogen levels among women on hormone therapy[16] we hypothesized that alcohol might amplify the risk of stroke observed with hormone therapy; however, the interaction was not statistically significant. Chance findings associated with evaluation of multiple subgroups may also explain apparent heterogeneity.

NIH-PA Author Manuscript NIH-PA Author Manuscript NIH-PA Author Manuscript

Alcohol may influence risk of stroke though several mechanisms dependent on level of consumption. Alcohol consumption may be anti-thrombotic and –atherogenic leading to increased HDL, decreased platelet aggregation, clot formation and increased fibrinolysis.[17] This may simultaneously lower risk of ischemic while increasing risk of hemorrhagic stroke. Higher levels of alcohol consumption may increase risk of ischemic and hemorrhagic stroke through both acute and chronic effects on blood pressure. Alcohol may influence risk of stroke through acute (e.g. atrial fibrillation and blood pressure) and chronic processes (e.g. atherosclerosis), shown by the similar results obtained in sensitivity analyses examining recent and cumulative intakes. Notably, the inverse association between alcohol and CHD is substantially stronger than for stroke, exhibiting a linear dose-response curve. Unidentified risk factors and/or differences in the strength of the associations for alcohol with key risk factors for stroke and CHD (e.g. hypertension) may explain these differences.[18]

Alcohol consumption was measured using self-reported FFQs with high reproducibility in this population and validity against plasma HDL.[8] We cannot rule out bias due to underreporting alcohol potentially leading to spurious associations at lower levels of consumption. Adjustment for various demographic, lifestyle, and dietary factors had a marginal influence on the association, minimizing potential unmeasured confounding by lifestyle factors.

We had limited power to assess heavy alcohol consumption and drinking patterns (e.g. binge drinking) due to variable collection and the range of available data. Although the gender and demographic composition of the NHS, potentially limit generalizability to men or ethnically diverse populations; similar results have been reported across diverse populations, with the exception of particular Asian populations (e.g. Japanese).[19]

Important strengths include the large sample size, longitudinal design with 26 years of follow-up and updated information on alcohol and confounders. Furthermore, we conducted detailed analyses to address changes in alcohol consumption over time, either due to behavior, chronic disease or dependency.

Summary

In this population of women, modest alcohol consumption was not associated with an elevated stroke risk, within the range of alcohol consumption observed in this cohort. Low-to-moderate alcohol consumption was associated with a modestly lower risk of total stroke. Hence, our data are consistent with the current American Heart Association guidelines for women, suggesting a modest inverse association between alcohol consumption of ≤1 drink/day with risk of total, ischemic and hemorrhagic stroke.

Supplementary Material

Refer to Web version on PubMed Central for supplementary material.

Acknowledgments

Funding: R01HL088521-02S1 and HL34594 from the National Heart, Lung and Blood Institute of the National Institutes of Health.

References

1. Reynolds K, Lewis B, Nolen JD, Kinney GL, Sathya B, He J. Alcohol consumption and risk of stroke: A meta-analysis. JAMA. 2003; 289:579–588. [PubMed: 12578491]

NIH-PA Author Manuscript

2. Patra J, Taylor B, Irving H, Roerecke M, Baliunas D, Mohapatra S, Rehm J. Alcohol consumption and the risk of morbidity and mortality for different stroke types--a systematic review and meta-analysis. BMC Public Health. 2010; 10:258. [PubMed: 20482788]

3. Pleis J, Lucas J, Ward B. Summary health statistics for us adults: National health interview survey, 2008. 2009:10.

4. Chiuve SE, Rexrode KM, Spiegelman D, Logroscino G, Manson JE, Rimm EB. Primary prevention of stroke by healthy lifestyle. Circulation. 2008; 118:947–954. [PubMed: 18697819]

5. Stampfer MJ, Colditz GA, Willett WC, Speizer FE, Hennekens CH. A prospective study of moderate alcohol consumption and the risk of coronary disease and stroke in women. N Engl J Med. 1988; 319:267–273. [PubMed: 3393181]

6. U.S. Department of Agriculture and U.S. Department of Health and Human Services. Dietary guidelines for Americans, 2010. 7. Washington, DC: U.S. Government Printing Office; Dec. 2010

7. Fuchs CS, Stampfer MJ, Colditz GA, Giovannucci EL, Manson JE, Kawachi I, Hunter DJ, Hankinson SE, Hennekens CH, Rosner B. Alcohol consumption and mortality among women. N Engl J Med. 1995; 332:1245–1250. [PubMed: 7708067]

8. Giovannucci E, Colditz G, Stampfer MJ, Rimm EB, Litin L, Sampson L, Willett WC. The assessment of alcohol consumption by a simple self-administered questionnaire. Am J Epidemiol. 1991; 133:810–817. [PubMed: 2021148]

9. Walker AE, Robins M, Weinfeld FD. The National Survey of Stroke. Clinical findings. Stroke. 1981; 12:I13–44. [PubMed: 7222164]

10. Stampfer MJ, Hu FB, Manson JE, Rimm EB, Willett WC. Primary prevention of coronary heart disease in women through diet and lifestyle. N Engl J Med. 2000; 343:16–22. [PubMed: 10882764]

11. Hu FB, Stampfer MJ, Rimm E, Ascherio A, Rosner BA, Spiegelman D, Willett WC. Dietary fat and coronary heart disease: A comparison of approaches for adjusting for total energy intake and modeling repeated dietary measurements. Am J Epidemiol. 1999; 149:531–540. [PubMed: 10084242]

12. Govindarajulu US, Malloy EJ, Ganguli B, Spiegelman D, Eisen EA. The comparison of alternative smoothing methods for fitting non-linear exposure-response relationships with cox models in a simulation study. Int J Biostat. 2009; 5:Article 2. [PubMed: 20231865]

13. Heinzl H, Kaider A. Gaining more flexibility in cox proportional hazards regression models with cubic spline functions. Comput Methods Programs Biomed. 1997; 54:201–208. [PubMed: 9421665]

14. Djousse L, Ellison RC, Beiser A, Scaramucci A, D'Agostino RB, Wolf PA. Alcohol consumption and risk of ischemic stroke: The Framingham Study. Stroke. 2002; 33:907–912. [PubMed: 11935035]

15. Schoonderwoerd BA, Smit MD, Pen L, Van Gelder IC. New risk factors for atrial fibrillation: Causes of 'not-so-lone atrial fibrillation'. Europace. 2008; 10:668–673. [PubMed: 18480076]

16. Ginsburg ES, Mello NK, Mendelson JH, Barbieri RL, Teoh SK, Rothman M, Gao X, Sholar JW. Effects of alcohol ingestion on estrogens in postmenopausal women. JAMA. 1996; 276:1747–1751. [PubMed: 8940324]

17. Rimm EB, Williams P, Fosher K, Criqui M, Stampfer MJ. Moderate alcohol intake and lower risk of coronary heart disease: Meta-analysis of effects on lipids and haemostatic factors. BMJ. 1999; 319:1523–1528. [PubMed: 10591709]

18. Hvidtfeldt UA, Tolstrup JS, Jakobsen MU, Heitmann BL, Gronbaek M, O'Reilly E, Balter K, Goldbourt U, Hallmans G, Knekt P, Liu S, Pereira M, Pietinen P, Spiegelman D, Stevens J, Virtamo J, Willett WC, Rimm EB, Ascherio A. Alcohol intake and risk of coronary heart disease in younger, middle-aged, and older adults. Circulation. 2010; 121:1589–1597. [PubMed: 20351238]

19. Kiyohara Y, Kato I, Iwamoto H, Nakayama K, Fujishima M. The impact of alcohol and hypertension on stroke incidence in a general Japanese population. The Hisayama Study. Stroke. 1995; 26:368–372. [PubMed: 7886708]

NIH-PA Author Manuscript
NIH-PA Author Manuscript
NIH-PA Author Manuscript

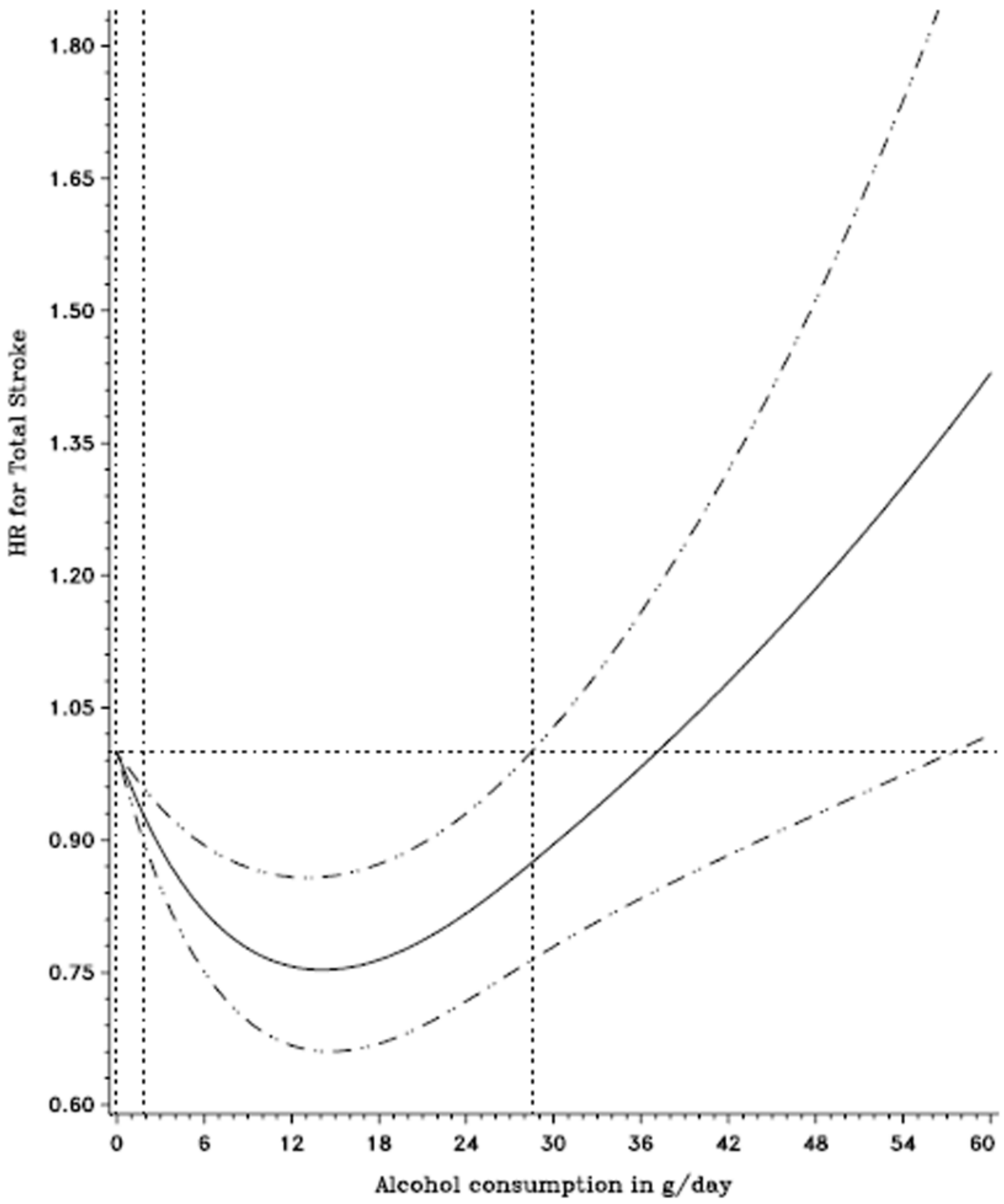

Figure 1.
Multivariable association between alcohol consumption and total stroke. Association estimated by Cox regression based on restricted cubic splines, dashed lines represent 95% confidence limits for adjusted estimates. Dashed vertical lines represent knot placement at 5th, 50th, and 95th percentiles.

NIH-PA Author Manuscript
NIH-PA Author Manuscript
NIH-PA Author Manuscript

Table 1

Baseline characteristics of population by categories of alcohol consumption in 1980

	Alcohol Intake categories g/day (median)				
	None (0)	>0–4.9 (1.8)	5.0–14.9 (10.2)	15.0–29.9 (19.5)	30–45 (35.8)
N, %	25,134 (30%)	29,420 (35%)	19,544 (23%)	6,093 (7%)	3,387 (4%)
Age	46.1 ± 7.2	46.0 ± 7.3	46.1 ± 7.1	46.3 ± 6.9	46.4 ± 6.9
White, %	97%	98%	98%	99%	99%
Alcohol Consumed (g/d)	0	1.9 ± 1.2	9.7 ± 3.0	20.9 ± 4.7	36.1 ± 3.4
Body Mass Index (kg/m^2)	25.3 ± 5.1	24.5 ± 4.4	23.5 ± 3.7	23.2 ± 3.5	23.4 ± 3.6
Smoking					
Never, %	59	45	34	27	19
Past, %	19	28	34	38	28
Current, %	22	27	33	35	53
Hormone Therapy					
Premenopausal, %	61	62	62	62	61
Never Users, %	26	25	24	23	23
Past Users, %	11	11	10	11	11
Current Users, %	8	8	8	9	9
Multivitamin Use, %	32	34	35	37	35
Physical Activity, % (h/wk)					
≥6	18	22	26	28	23
3.5–6	13	13	13	13	11
1.0–3.5	32	31	28	26	30
<1.0	37	34	33	33	36
Diabetes, %	1	0.5	0.3	0.3	0.2
Hypertension, %	17	15	15	16	19
High Cholesterol, %	2	2	2	2	2
Family History of MI, %	20	21	21	20	21

Values are means ± SD or percentages and all values, except age, are standardized to the age distribution of the study population.

g/d=grams/day; kg/m^2=kilograms/meters^2; h/wk=hours/week; MI=myocardial infarction

NIH-PA Author Manuscript NIH-PA Author Manuscript NIH-PA Author Manuscript

Table 2

Multivariable* association between alcohol and incidence of total, ischemic and hemorrhagic stroke

		Alcohol Intake categories g/day					
**N=83,578**		**None**	**>0–4.9**	**5.0–14.9**	**15.0–29.9**	**30–45**	**p-value, deviation from linearity**
Total Stroke							
Events	2,171	1045	552	341	131	102	
Smoking adjusted	HR (95% CI)	1.00	0.74 (0.67–0.82)	0.66 (0.58–0.75)	0.71 (0.59–0.86)	0.94 (0.76–1.16)	<0.001
Multivariable Model	HR (95% CI)	1.00	0.83 (0.75–0.92)	0.79 (0.70–0.90)	0.87 (0.72–1.05)	1.06 (0.86–1.30)	<0.001
Ischemic Stroke							
Events	1,206	566	318	196	65	61	
Smoking adjusted	HR (95% CI)	1.00	0.78 (0.68–0.90)	0.71 (0.60–0.84)	0.67 (0.52–0.87)	1.06 (0.81–1.39)	<0.001
Multivariable Model	HR (95% CI)	1.00	0.88 (0.76–1.02)	0.86 (0.72–1.02)	0.82 (0.63–1.07)	1.17 (0.89–1.54)	0.002
Hemorrhagic Stroke							
Events	363	156	97	65	27	18	
Smoking adjusted	HR (95% CI)	1.00	0.76 (0.59–0.98)	0.71 (0.53–0.96)	0.84 (0.55–1.27)	0.91 (0.55–1.49)	0.89
Multivariable Model	HR (95% CI)	1.00	0.82 (0.63–1.06)	0.76 (0.56–1.03)	0.88 (0.58–1.35)	0.97 (0.58–1.60)	0.66

* All models adjusted for age (months)

Multivariable Model covariates: Smoking, physical activity, BMI kg/m^2, history of heart disease, family history of heart disease, history of diabetes, bilateral oophorectomy, postmenopausal status, use of hormone therapy, high cholesterol, multivitamin intake, aspirin intake, 6 nutrient diet score, highest level of education achieved, husband's level of education, marital status

NIH-PA Author Manuscript NIH-PA Author Manuscript NIH-PA Author Manuscript

Table 3

Multivariable association between alcohol and total stroke stratified by key risk factors, HR (95% CI)

		Alcohol Intake categories g/day					
**Total Stroke**		**None**	**>0–4.9**	**5.0–14.9**	**15.0–29.9**	**30–45**	**p-value for interaction**
**Age**							
<60	Events	822	399	239	94	75	0.75
	HR (95% CI)	1.00	0.77 (0.62–0.95)	0.82 (0.64–1.05)	0.91 (0.63–1.30)	0.92 (0.61–1.40)	
≥60	Events	223	153	102	37	27	
	HR (95% CI)	1.00	0.85 (0.76–0.97)	0.78 (0.67–0.91)	0.86 (0.69–1.07)	1.12 (0.88–1.43)	
**Hypertension**							
Yes	Events	613	278	163	55	58	0.57
	HR (95% CI)	1.00	0.82 (0.71–0.95)	0.76 (0.64–0.92)	0.71 (0.53–0.94)	1.00 (0.76–1.33)	
No	Events	432	274	178	76	44	
	HR (95% CI)	1.00	0.85 (0.72–0.99)	0.81 (0.68–0.98)	1.04 (0.81–1.34)	1.11 (0.80–1.53)	
**Aspirin Use**							
Non-user	Events	484	243	145	59	47	>.99
	HR (95% CI)	1.00	0.81 (0.69–0.95)	0.80 (0.66–0.97)	0.91 (0.69–1.20)	1.13 (0.82–1.54)	
1–5/wk	Events	133	89	55	20	15	
	HR (95% CI)	1.00	0.82 (0.62–1.09)	0.80 (0.57–1.12)	0.87 (0.53–1.42)	1.06 (0.60–1.87)	
≥6/wk	Events	428	220	141	52	40	
	HR (95% CI)	1.00	0.84 (0.71–1.00)	0.79 (0.65–0.97)	0.78 (0.58–1.05)	1.01 (0.72–1.41)	
**Hormone Therapy**							
Never	Events	360	161	115	36	25	0.19
	HR (95% CI)	1.00	0.75 (0.62–0.91)	0.89 (0.72–1.12)	0.84 (0.59–1.19)	0.76 (0.50–1.16)	
Past	Events	337	154	98	47	30	
	HR (95% CI)	1.00	0.81 (0.66–0.98)	0.77 (0.60–0.97)	0.97 (0.71–1.34)	1.01 (0.68–1.49)	
Current	Events	300	192	104	39	42	
	HR (95% CI)	1.00	0.90 (0.74–1.09)	0.74 (0.59–0.94)	0.82 (0.58–1.17)	1.57 (1.12–2.22)	
**Smoking**							
Never	Events	493	180	88	28	16	0.56
	HR (95% CI)	1.00	0.74 (0.62–0.89)	0.81 (0.64–1.02)	0.91 (0.62–1.35)	1.32 (0.79–2.19)	
Former	Events	382	241	153	66	40	

			Alcohol Intake categories g/day				
**Total Stroke**		**None**	**>0–4.9**	**5.0–14.9**	**15.0–29.9**	**30–45**	**p-value for interaction**
	HR (95% CI)	1.00	0.83 (0.70–0.98)	0.74 (0.60–0.90)	0.84 (0.64–1.10)	1.04 (0.74–1.45)	
Current	Events	170	131	100	37	46	
	HR (95% CI)	1.00	0.99 (0.78–1.26)	0.93 (0.71–1.20)	1.00 (0.69–1.46)	1.19 (0.84–1.68)	
**Atrial Fibrillation** *							
Yes	Events	63	19	16	14	7	0.03
	HR (95% CI)	1.00	0.73 (0.41–1.27)	0.85 (0.46–1.56)	1.65 (0.84–3.25)	1.30 (0.53–3.19)	
No	Events	365	136	81	35	21	
	HR (95% CI)	1.00	0.75 (0.62–0.92)	0.64 (0.50–0.82)	0.55 (0.39–0.79)	0.71 (0.45–1.11)	

See Table 2 for multivariable model covariates.

*n=51,846

NIH-PA Author Manuscript NIH-PA Author Manuscript NIH-PA Author Manuscript
