# Supplementary material for: A systematic comprehensive longitudinal evaluation of dietary factors associated with acute myocardial infarction and fatal coronary heart disease
Source: Nat Commun. 2020 Nov 27;11:6074. doi: 10.1038/s41467-020-19888-2 (PMC7699643; doi:10.1038/s41467-020-19888-2)
Supplement: Supplementary file 4 — Supplementary Software [file 41467_2020_19888_MOESM4_ESM.zip › EWAS-NHS-master/KG-SI/Rest/Le Ma (2018).pdf]

Clinical Epidemiology

Dovepress
open access to scientific and medical research

Open Access Full Text Article

ORIGINAL RESEARCH

Intake of glucosinolates and risk of coronary heart disease in three large prospective cohorts of US men and women

This article was published in the following Dove Press journal:
Clinical Epidemiology

Le Ma[1,2]
Gang Liu[1]
Geng Zong[1]
Laura Sampson[1]
Frank B Hu[1,3,4]
Walter C Willett[1,3,4]
Eric B Rimm[1,3,4]
JoAnn E Manson[3,5,6]
Kathryn M Rexrode[6]
Qi Sun[1,4]

[1]Department of Nutrition, Harvard T.H. Chan School of Public Health, Boston, MA, USA; [2]Department of Maternal, School of Public Health, Xi'an Jiaotong University Health Science Center, Xi'an, China; [3]Department of Epidemiology, Harvard T.H. Chan School of Public Health, [4]Channing Division of Network Medicine, Department of Medicine, [5]Department of Medicine, [6]Division of Preventive Medicine, Department of Medicine, Brigham and Women's Hospital, Boston, MA, USA

Importance: Glucosinolates, a group of phytochemicals abundant in cruciferous vegetables, may have cardioprotective properties. However, no prospective study has evaluated the association of intake of glucosinolates with the risk of coronary heart disease (CHD).
Objective: The objective of the study was to evaluate the association between the intake of glucosinolates and incident CHD in US men and women.
Design: Prospective longitudinal cohort study.
Setting: Health professionals in the USA.
Participants: We followed 74,241 women in the Nurses' Health Study (NHS; 1984–2012), 94,163 women in the NHSII (1991–2013), and 42,170 men in the Health Professionals Follow-Up Study (1986–2012), who were free of cardiovascular disease and cancer at baseline.
Exposure: Glucosinolate intake was assessed using validated semi-quantitative food frequency questionnaires at baseline and updated every 2–4 years during follow-up.
Main outcome measures: Incident cases of CHD were confirmed by medical record review.
Results: During 4,824,001 person-years of follow-up, 8,010 cases of CHD were identified in the three cohorts. After adjustment for major lifestyle and dietary risk factors of CHD, weak but significantly positive associations were observed for glucosinolates with CHD risk when comparing the top with bottom quintiles (hazard ratio [HR]:1.09; 95% CI: 1.01, 1.17; P_{trend}<0.001). Higher intakes of three major subtypes of glucosinolates were consistently associated with a higher CHD risk, although the association for indolylglucosinolate did not achieve statistical significance. Regarding cruciferous vegetable intake, participants who consumed one or more servings per week of Brussels sprouts (HR: 1.16; 95% CI: 1.06, 1.26; *P*<0.001) and cabbage (HR: 1.09; 95% CI: 1.02, 1.17; *P*=0.009) had a significantly higher CHD risk than those who consumed these cruciferous vegetables less than once per month.
Conclusion and relevance: In these three prospective cohort studies, dietary glucosinolate intake was associated with a slightly higher risk of CHD in US adults. These results warrant replications in further studies including biomarker-based studies. Further studies are needed to confirm these findings and elucidate mechanistic pathways that may underlie these associations.
Keywords: coronary heart disease, glucosinolate, cruciferous vegetable, diet

Introduction

The American Heart Association guidelines underscore the importance of increasing consumption of vegetables for the prevention of heart disease and other chronic conditions, and a variety of vegetables in a healthy diet has been recently emphasized by the US Department of Agriculture Dietary Guidelines for Americans as well.[1,2] Growing evidence indicates that specific types of vegetables may have distinct effects

Correspondence: Qi Sun
Department of Nutrition, Harvard T.H. Chan School of Public Health, 665 Huntington Ave, Boston, MA 02115, USA
Tel +1 617 432 7490
Fax +1 617 432 3435
Email qisun@hsph.harvard.edu

© 2018 Ma et al. This work is published and licensed by Dove Medical Press Limited. The full terms of this license are available at https://www.dovepress.com/terms.php and incorporate the Creative Commons Attribution – Non Commercial (unported, v3.0) License (http://creativecommons.org/licenses/by-nc/3.0/). By accessing the work you hereby accept the Terms. Non-commercial uses of the work are permitted without any further permission from Dove Medical Press Limited, provided the work is properly attributed. For permission for commercial use of this work, please see paragraphs 4.2 and 5 of our Terms (https://www.dovepress.com/terms.php).

on the risk for coronary heart disease (CHD), which may be due to the heterogeneous composition of nutrients and other constituents in vegetables.[3,4]

Glucosinolates are a class of secondary plant metabolites that are particularly rich in cruciferous vegetables.[5,6] Dietary glucosinolates can be hydrolyzed to biologically active compounds, such as isothiocyanates (ITCs), which are able to modulate cellular redox status and protect against carcinogenesis in animal experiements.[6,7] Emerging evidence from experimental studies has shown that glucosinolate metabolites can reduce oxidative stress, inflammation, endothelial dysfunction, and cardiomyocyte death,[8–10] indicating that these compounds may also have beneficial effects on the cardiovascular system. Despite the evidence from basic science research, human data regarding glucosinolates intake and CHD risk are limited. A couple of epidemiologic studies investigated cruciferous vegetable intake in relation to risk of CHD, and mixed results were observed.[11,12] Furthermore, existing evidence from relatively small clinical trials regarding the effects of glucosinolates or glucosinolate-rich foods on the development of coronary intermediate endpoints remains limited and inconclusive.[13,14]

In the current investigation, we aimed to evaluate the hypothesis that higher glucosinolate intake is associated with lower risk of CHD. To test this hypothesis, we prospectively examined dietary glucosinolate intake, as well as major dietary glucosinolate sources, in relation to the risk of CHD.

Subjects and methods

Study population

Participants in this analysis were US men and women from three prospective cohort studies: Nurses' Health Study (NHS; n=121,700 female registered nurses enrolled in 1976), NHSII (n=116,686 younger female registered nurses enrolled in 1989), and Health Professionals Follow-Up Study (HPFS; n=51,529 male health professionals enrolled in 1986). Detailed descriptions of the cohorts are provided elsewhere.[15] For this analysis, we excluded participants who had diagnoses of cardiovascular disease (CVD) or cancer at baseline, left 70 or more items blank on the food frequency questionnaire (FFQ), reported implausible energy intake (<3,347 or >17,573 kJ/day for men and <2,510 or >14,644 kJ/day for women), did not complete the baseline FFQ or questions of cruciferous vegetables intake, or who only returned the baseline questionnaire. After exclusions, a total of 210,574 participants (74,241 in NHS, 94,163 in NHSII, and 42,170 in HPFS) were included in the current analysis. The study protocol was approved by the institutional review boards of Brigham and Women's Hospital and Harvard T.H. Chan School of Public Health. The completion of the self-administered questionnaire was considered to imply written informed consent.

Assessment of glucosinolate intake

In each cohort, the intake of glucosinolates was assessed using validated FFQs every 2–4 years. The FFQs inquired about the consumption of selected foods (with a prespecified serving size) during the past year with nine categories of intake frequency. The Harvard University Food Composition Database was primarily used to calculate the nutrient values, complemented by published data.[16] Intake of individual glucosinolates was calculated by multiplying the glucosinolate levels in a prespecified portion size with the consumption frequency for each contributing food item and then summing the intake levels across all contributing food items. Intake of total and subgroups of glucosinolates was derived by summing up individual glucosinolates in each category. Glucosinolate intakes were energy-adjusted using the residual method. Reasonable validity and reproducibility of the assessments of food sources of glucosinolates, including broccoli, cabbage, and Brussels sprouts, have been demonstrated in validation studies.[17–19]

Assessment of covariates

In all three cohorts, information on age, body weight, medical history, smoking status, physical activity, parental history of myocardial infarction (MI) before age 65 years, medical history, menopausal status and use of hormone therapy (women only), and medication use was collected and updated in biennial validated questionnaires. Alcohol intake was assessed and updated by validated FFQs. Detailed descriptions on the validity and reproducibility of these assessments have been published elsewhere.[20–22] We calculated an Alternative Healthy Eating Index (AHEI) score to quantify the overall diet quality of the participants.[23] The AHEI score summarizes the intake of 11 foods or nutrients that are most predictive of chronic diseases: vegetables, fruits, whole grains, nuts and legumes, long-chain n-3 fats, polyunsaturated fats, sugar-sweetened beverages and fruit juice, red and processed meat, trans fat, sodium, and alcohol. Individual food/nutrient items were scored from 0 (worst) to 10 (best) based on prespecified criteria,[24] with a higher score received for higher intake of healthy foods/nutrients (i.e., vegetables, fruits, whole grains, nuts and legumes, long-chain n-3 fats, polyunsaturated fats), lower intake of less healthy components (i.e., sugar-sweetened beverages and fruit juice, red and processed meat,

trans fat, and sodium), or moderate intake of alcohol. A total AHEI score has the possible range from 0 (lowest quality) to 110 (highest quality). For the current analysis, we excluded cruciferous vegetables when calculating the AHEI score.

Ascertainment of endpoint

The primary endpoints for this study were incident CHD (defined as nonfatal MI and fatal CHD). Participants who reported a new diagnosis of MI on a biennial follow-up questionnaire were asked for permission to review their medical records. Medical records were reviewed by the study physicians blinded to the exposure status of the patients. Nonfatal CHD cases were confirmed according to the World Health Organization criteria, which require typical symptoms plus either diagnostic electrocardiographic changes or elevated cardiac enzyme concentrations.[25] Fatal CHD was identified by reports from next of kin, postal authorities, or by searching the National Death Index. Fatal CHD was confirmed through reviewing death certificates, hospital records, or autopsy reports if CHD was listed as the cause of death and if evidence of previous CHD was available from medical records. When CHD was listed as the underlying cause on the death certificate but no prior knowledge of CHD was indicated and medical records concerning the death were unavailable, we designated such cases as probable fatal CHD cases.[26] Because the exclusion of probable CHD cases did not alter the results, we included both confirmed and probable cases in our study to maximize statistical power.

Statistical analysis

We calculated person-years of follow-up from the return date of the baseline questionnaire to the date of CHD diagnosis, death, or the end of follow-up (NHS: 30 June, 2012; NHSII: 30 June, 2013; and HPFS: 31 January, 2012), whichever came first. To better represent long-term habitual intake and to reduce random within-person variation, we used the cumulative average of food intakes from all FFQs from baseline through the end of follow-up.[27] We stopped updating diet after participants reported a diagnosis of angina, coronary artery bypass graft, diabetes, or cancer, because of possible changes of usual diet after occurrence of these conditions. The hazard ratios (HRs) and 95% CIs of incident CHD were estimated for glucosinolate intake by using time-dependent Cox proportional hazards regression after pooling data from three cohorts. The analysis was stratified jointly by age (years) and calendar year, and adjusted for ethnicity (Caucasian, African American, Asian, and other ethnicity), body mass index (BMI, <23.0, 23.0–24.9, 25.0–29.9, 30.0–34.9, ≥35 kg/m^2, or missing), smoking status (never, former, current [1–14, 15–24, or ≥25 cigarettes/day], or missing), alcohol intake (0, 0.1–4.9, 5.0–14.9, and ≥15.0 g/day for women; 0, 0.1–4.9, 5.0–29.9, and ≥30.0 g/day for men; or missing), physical activity (<3.0, 3.0–8.9, 9.0–17.9, 18.0–26.9, ≥27.0 metabolic equivalents of task-hours/week, or missing), menopausal status and postmenopausal hormone use (premenopause, postmenopause [never, former, or current hormone use], or missing), oral contraceptive use (yes, no, or missing, NHSII only), family history of heart disease (yes/no), multivitamin use (yes/no), hypertension (yes/no), hypercholesterolemia (yes/no), total energy intake (kcal/day), and modified AHEI score (quintiles). A test for linear trend was conducted by assigning the median value to each category and modeling this value as a continuous variable. We used restricted cubic spline regressions with four knots to examine the dose–response relationships between glucosinolate intake and the risk of CHD. We evaluated the potential effect modification by race, age, BMI, the modified AHEI score, physical activity, smoking status, and alcohol consumption using the likelihood ratio test by comparing models with main effects and interaction terms with models containing the main effects only. We also examined the associations of major glucosinolate subgroups and individual glucosinolates, separately, on the risk of CHD. To test the robustness of our findings, we conducted four sensitivity analyses: 1) adjusting for individual dietary variables instead of the modified AHEI score; 2) using only baseline dietary variables; 3) continuing updating dietary information after participant reported a diagnosis of cancer or diabetes; and 4) placing a 4- or 8-year lag between the assessments of glucosinolate intake and CHD ascertainment. Statistical analyses were performed using SAS statistical software, version 9.3 (SAS Institute Inc.). All *P*-values presented were two-sided, with statistical significance defined as $P<0.05$.

Results

During 4,824,001 person-years of follow-up, we documented 8,010 incident cases of nonfatal MI or fatal CHD. Stratified incidence density of CHD according to total glucosinolate intake by various characteristics of participants is shown in Table S1. Table 1 presents the age-standardized baseline characteristics of the study population by glucosinolate intake. In all three cohorts, participants with higher glucosinolate intake were older and more physically active and had a higher modified AHEI score. They consumed less red meat and more fruits and vegetables. Higher glucosinolate intake was associated with lower trans fat intake and a higher polyunsaturated fat-to-saturated fat ratio.

Table 1 Baseline characteristics of the participants according to total glucosinolate intake in the NHS, NHSII, and HPFS[a]

Characteristics	NHS			NHSII			HPFS		
	Q1	Q3	Q5	Q1	Q3	Q5	Q1	Q3	Q5
Participants, n	14,826	14,855	14,858	18,828	18,820	18,828	8,429	8,433	8,430
Glucosinolate intake, mg/day	3.28[b]	10.5	29.1	2.06	7.68	25.9	2.15	9.87	29.4
Age[c], years	49.3	50.3	51.2	35.2	36.2	37.0	52.7	53.0	54.2
Caucasians, %	98	98	97	96	96	95	96	95	94
Current smoker, %	26	24	23	13	12	13	11	10	8
Alcohol intake, g/day	6.69	7.23	6.41	2.99	3.07	3.06	11.0	12.6	10.5
Physical activity, MET/week	11.7	14.0	17.1	17.8	20.5	25.2	19.3	20.7	23.8
BMI, kg/m^2	24.7	25.0	25.4	24.7	24.6	24.8	24.9	24.9	25.0
Family history of myocardial infarction, %	38	39	40	33	32	33	32	31	32
Multivitamin use, %	64	63	59	44	42	44	40	41	45
Ever menopausal hormone use, %	21	22	22	3	3	3	–	–	–
Current use of oral contraceptive, %	–	–	–	11	11	10	–	–	–
Total energy intake, kcal/day	1,776	1,782	1,686	1,883	1,707	1,692	2,031	2,082	1,905
Modified AHEI score	42.7	46.4	53.0	40.7	45.9	51.5	45.1	48.5	54.6
Trans fat intake, % energy	2.04	1.94	1.70	1.81	1.67	1.44	1.40	1.31	1.08
Polyunsaturated fat-to-saturated fat ratio	0.52	0.55	0.58	0.49	0.52	0.57	0.53	0.56	0.63
Total fruits intake, servings/day	1.83	2.12	2.47	0.97	1.12	1.41	2.02	2.33	2.70
Total vegetables intake, servings/day	2.17	2.88	4.39	2.17	2.89	4.60	2.18	2.90	4.30
Cruciferous vegetables intake, servings/day	0.12	0.36	1.01	0.09	0.32	0.94	0.14	0.39	1.02
Red meat intake, servings/day	1.24	1.19	0.99	0.92	0.77	0.64	1.29	1.24	0.93

Notes: [a]Values were standardized to the age distribution of the study population. [b]Data are mean unless otherwise indicated. [c]Values were not age adjusted.
Abbreviations: AHEI, Alternative Healthy Eating Index; BMI, body mass index; HPFS, the Health Professionals Follow-Up Study; MET, metabolic equivalents of task; NHS, Nurses' Health Study.

In the three cohorts, higher intake of total glucosinolates was consistently associated with a higher risk of CHD after adjustment for demographic, lifestyle, and dietary risk factors (Table 2). In pooled multivariable analyses, an increased intake of total glucosinolate was significantly associated with a slightly higher CHD risk. The multivariable-adjusted HR (95% CI) of CHD comparing participants in the highest vs lowest quintiles was 1.09 (95% CI: 1.01, 1.17; P_{trend}<0.001).

Spline regression analyses showed that the association between total glucosinolate intake and risk of CHD was likely to be linear ($P_{linearity}$<0.001 and $P_{curvature}$=0.70; Figure S1). For each SD increment of glucosinolate intake, the risk of CHD increased by 3% (95% CI: 1%, 5%; P=0.01).

We did not detect statistically significant interactions of total glucosinolate intake with ethnicity, age, BMI, the modified AHEI score, physical activity, smoking status, or alcohol consumption in relation to CHD risk (all $P_{interaction}$ >0.10; Table S2). The association of glucosinolate intake with CHD appeared to be stronger among white participants (HR: 1.09; 95% CI: 1.01, 1.17; P_{trend}=0.001) than among non-white participants (HR: 0.95; 95% CI: 0.62, 1.46; P_{trend}=0.34), comparing extreme quintiles.

In the sensitivity analyses, adjustment for other major dietary factors instead of the modified AHEI score slightly attenuated the HR (95% CI) per SD increment of glucosinolate intake for CHD to 1.02 (0.99, 1.04) (P=0.15). When we continued updating dietary variables throughout follow-up even after a diagnosis of cancer or diabetes, the associations did not change materially (HR per SD change: 1.03; 95% CI: 1.00, 1.05; P=0.02). Use of baseline glucosinolate intake instead of the cumulative average yielded similar results (HR per SD change: 1.03; 95% CI: 1.00, 1.05; P=0.01). Placing a 4-year (HR per SD change: 1.03; 95% CI: 1.01, 1.05; P=0.01) or an 8-year lag (HR per SD change: 1.03; 95% CI: 1.00, 1.05; P=0.03) also did not change the associations of CHD (Table S3).

Trends toward increased CHD risk were observed for all three glucosinolate subgroups. Multivariable HRs (95% CIs) for CHD comparing the highest vs lowest quintiles of glucosinolates were 1.10 (1.02, 1.18), 1.04 (0.97, 1.12), and 1.16 (1.08, 1.24) for aliphatic glucosinolate, indolylglucosinolate, and aromatic glucosinolate, respectively (Table 3). Each SD increment of these glucosinolate subgroup intakes was associated with a 3%, 2%, and 3% greater risk of CHD, respectively. In the analyses of individual glucosinolates, a positive trend was also observed for glucobrassicin, sinigrin, and glucoiberin, although only the associations for sinigrin and glucoiberin achieved statistical significance (Table S4).

Higher cruciferous vegetable consumption was non-significantly associated with an increased risk of CHD.

Table 2 Hazard ratio (95% CI) of coronary heart disease according to quintiles of total glucosinolate intake

Cohort and Model	Quintiles of total glucosinolate intake					P_{trend}
	1 (low)	2	3	4	5 (high)	
NHS						
Median intake, mg/day	4.1	7.2	10.4	14.4	22.2	
Number of cases/person-year	623/373,483	591/374,230	648/374,463	631/374,038	672/373,560	
Rate per 100,000 person-years	167	158	173	169	180	
Model 1[a]	1	0.91 (0.82, 1.01)	0.95 (0.86, 1.06)	0.97 (0.87, 1.08)	0.98 (0.89, 1.09)	0.66
Model 2[b]	1	0.97 (0.87, 1.08)	1.03 (0.92, 1.14)	1.03 (0.93, 1.15)	1.02 (0.92, 1.13)	0.44
Model 3[c]	1	1.00 (0.89, 1.11)	1.08 (0.97, 1.21)	1.12 (1.00, 1.24)	1.15 (1.03, 1.29)	0.003
NHSII						
Median intake, mg/day	2.7	5.1	8.0	12.6	21.1	
Number of cases/person-year	136/405,224	137/405,753	109/406,088	124/406,057	157/405,520	
Rate per 100,000 person-years	34	34	27	31	39	
Model 1[a]	1	0.94 (0.74, 1.19)	0.73 (0.57, 0.94)	0.80 (0.62, 1.02)	0.93 (0.74, 1.18)	0.79
Model 2[b]	1	1.06 (0.83, 1.34)	0.88 (0.68, 1.13)	0.98 (0.76, 1.25)	1.04 (0.82, 1.32)	0.76
Model 3[c]	1	1.08 (0.85, 1.37)	0.91 (0.70, 1.18)	1.04 (0.81, 1.34)	1.16 (0.91, 1.49)	0.20
HPFS						
Median intake, mg/day	3.2	6.7	10.5	15.1	24.4	
Number of cases/person-year	919/184,615	758/185,287	764/185,379	834/185,248	907/185,056	
Rate per 100,000 person-years	498	409	412	450	490	
Model 1[a]	1	0.85 (0.78, 0.94)	0.84 (0.76, 0.93)	0.91 (0.83, 1.00)	0.91 (0.83, 1.00)	0.52
Model 2[b]	1	0.88 (0.80, 0.97)	0.89 (0.81, 0.98)	0.97 (0.88, 1.07)	0.96 (0.88, 1.06)	0.70
Model 3[c]	1	0.90 (0.81, 0.99)	0.91 (0.83, 1.01)	1.01 (0.91, 1.11)	1.03 (0.93, 1.13)	0.09
Pooled[d]						
Model 1[a]	1	0.88 (0.82, 0.95)	0.88 (0.82, 0.94)	0.92 (0.86, 0.99)	0.94 (0.88, 1.01)	0.76
Model 2[b]	1	0.93 (0.87, 1.00)	0.95 (0.88, 1.01)	1.00 (0.93, 1.07)	0.99 (0.93, 1.06)	0.42
Model 3[c]	1	0.95 (0.89, 1.02)	0.98 (0.91, 1.05)	1.05 (0.98, 1.13)	1.09 (1.01, 1.17)	<0.001

Notes: [a]Estimates are calculated in Cox proportional hazards models. Model 1, adjusted for age (years). [b]Model 2, further adjusted for ethnicity (Caucasian, African American, Asian, and other ethnicity), family history of myocardial infarction (yes/no), hypertension (yes/no), hypercholesterolemia (yes/no), smoking status (never, former, current [1–14, 15–24, or ≥25 cigarettes/day], or missing), alcohol intake (0, 0.1–4.9, 5.0–14.9, and ≥15.0 g/day for women, 0, 0.1–4.9, 5.0–29.9, and ≥30.0 g/day for men, or missing), physical activity (<3.0, 3.0–8.9, 9.0–17.9, 18.0–26.9, ≥27.0 MET-hours/week, or missing), menopausal status and postmenopausal hormone use (premenopause, postmenopause [never, former, or current hormone use], or missing, for women), oral contraceptive use (yes, no, or missing, for NHSII), multivitamin use (yes/no), BMI (<23, 23–24.9, 25–29.9, 30–34.9, ≥35 kg/m^2, or missing), and total energy intake (kcal/day) based on model 1. [c]Model 3, further adjusted for modified Alternative Healthy Eating Index score (in quintiles), based on model 2. [d]Results from each cohort were pooled using fixed-effects model.
Abbreviations: BMI, body mass index; HPFS, Health Professionals Follow-up Study; MET, metabolic equivalents of task; NHS, Nurses' Health Study.

Compared with less than one serving cruciferous vegetable/week, the multivariable-adjusted HR was 1.04 (95% CI: 0.95, 1.14) for more than one serving/day of total cruciferous vegetables (Table 4). For individual cruciferous vegetables, significant associations were observed for Brussels sprouts (HR: 1.16; 95% CI: 1.08, 1.26; $P<0.001$) and cabbage (HR: 1.09; 95% CI: 1.02, 1.17; $P=0.009$; Table S5). Each two servings/week increment of Brussels sprouts and cabbage intake was associated with a 13% (95% CI: 5%, 21%) and 2% (95% CI: 0%, 3%) higher risk of CHD, respectively.

Discussion

In three cohorts of US men and women, we found weak to modest positive associations between intake of total and individual glucosinolates and incident CHD. This association was independent of established dietary and non-dietary CVD risk factors, and largely persisted among participants with various risk profiles. Increased consumption of food sources of glucosinolates, particularly Brussels sprouts and cabbage, was also associated with a higher risk of CHD.

The glucosinolates–myrosinase system is known as "mustard oil bomb" and used by Brassicales as a defense system against the aggressions of pathogens. Upon rupture of cellular membranes, active myrosinase comes in contact with glucosinolates, hydrolyzes the glucosinolates, and subsequently produces highly reactive metabolites that serve as a defense for the plants.[28] Mastication of fresh or lightly cooked Brassica vegetables with active myrosinase and metabolism by human gut microbiota when the myrosinase is inactivated are the two primary sources of exposure to ITCs and other metabolites.[29] Abundant evidence from experimental studies has illustrated that ITCs and other metabolites of glucosinolates may exhibit anticarcinogenic, anti-inflammatory, and antioxidant effects.[30,31] These bioactive compounds have been shown to induce Phase II and antioxidant gene expression through activation of nuclear

Table 3 HR (95% CI) of coronary heart disease according to quintiles of glucosinolate subgroups[a]

Variables and Cohort	Quintiles of intake 1 (low)	2	3	4	5 (high)	P_{trend}
Aliphatic glucosinolate						
NHS	1	0.99 (0.89, 1.10)	1.09 (0.97, 1.21)	1.13 (1.01, 1.26)	1.21 (1.09, 1.35)	<0.001
NHSII	1	1.04 (0.82, 1.32)	0.93 (0.72, 1.20)	0.98 (0.76, 1.27)	1.18 (0.93, 1.51)	0.15
HPFS	1	0.90 (0.81, 0.99)	0.89 (0.80, 0.98)	0.99 (0.90, 1.09)	1.00 (0.91, 1.10)	0.24
Pooled results[b]	1	0.95 (0.88, 1.01)	0.97 (0.90, 1.04)	1.05 (0.98, 1.12)	1.10 (1.02, 1.18)	<0.001
Indolylglucosinolate						
NHS	1	0.95 (0.85, 1.05)	1.04 (0.94, 1.16)	1.06 (0.95, 1.18)	1.05 (0.94, 1.18)	0.12
NHSII	1	1.11 (0.87, 1.40)	0.92 (0.72, 1.19)	1.00 (0.78, 1.29)	1.13 (0.88, 1.45)	0.41
HPFS	1	0.89 (0.81, 0.99)	0.97 (0.88, 1.07)	0.99 (0.89, 1.09)	1.01 (0.92, 1.12)	0.25
Pooled results[b]	1	0.93 (0.87, 1.00)	1.00 (0.93, 1.07)	1.02 (0.95, 1.09)	1.04 (0.97, 1.12)	0.04
Aromatic glucosinolate						
NHS	1	1.01 (0.91, 1.13)	1.07 (0.96, 1.19)	1.02 (0.92, 1.14)	1.26 (1.14, 1.40)	<0.001
NHSII	1	0.98 (0.78, 1.24)	0.94 (0.74, 1.20)	0.89 (0.68, 1.15)	1.03 (0.81, 1.32)	0.82
HPFS	1	0.97 (0.88, 1.07)	1.04 (0.94, 1.15)	1.11 (1.00, 1.22)	1.09 (0.99, 1.21)	0.02
Pooled results[b]	1	0.99 (0.92, 1.06)	1.04 (0.97, 1.12)	1.05 (0.98, 1.13)	1.16 (1.08, 1.24)	<0.001

Notes: [a]Estimates are calculated in Cox proportional hazards models, adjusted for age (years), ethnicity (Caucasian, African American, Asian, and other ethnicity), family history of myocardial infarction (yes/no), hypertension (yes/no), hypercholesterolemia (yes/no), smoking status (never, former, current [1–14, 15–24, or ≥25 cigarettes/day], or missing), alcohol intake (0, 0.1–4.9, 5.0–14.9, and ≥15.0 g/day for women, 0, 0.1–4.9, 5.0–29.9, and ≥30.0 g/day for men, or missing), physical activity (<3.0, 3.0–8.9, 9.0–17.9, 18.0–26.9, ≥27.0 MET-hours/week, or missing), menopausal status and postmenopausal hormone use (premenopause, postmenopause [never, former, or current hormone use], or missing, for women), oral contraceptive use (yes, no, or missing, for NHSII), multivitamin use (yes/no), BMI (<23, 23–24.9, 25–29.9, 30–34.9, ≥35 kg/m², or missing), and total energy intake (kcal/day), and the modified Alternate Healthy Eating Index score (quintiles). [b]Results from each cohort were pooled using fixed-effects model.
Abbreviations: BMI, body mass index; HPFS, Health Professionals Follow-Up Study; HR, hazard ratio; MET, metabolic equivalents of task; NHS, Nurses' Health Study.

Table 4 HR (95% CI) of coronary heart disease according to consumption of total cruciferous vegetables[a]

Cohort	Consumption level <2 serving/week	3–4 servings/week	5–6 servings/week	≥1 serving/day	Every two servings/week	P_{trend}
NHS						
Number of cases/person-year	1,019/588,840	1,249/744,024	688/413,660	209/123,250		
Rate per 100,000 person-years	173	168	166	170		
Multivariable adjusted HR[b]	1	0.99 (0.91, 1.07)	1.05 (0.95, 1.16)	1.02 (0.87, 1.19)	1.02 (0.99, 1.05)	0.43
NHSII						
Number of cases/person-year	307/927,209	204/637,036	94/338,994	58/125,404		
Rate per 100,000 person-years	33	32	28	46		
Multivariable adjusted HR[b]	1	1.01 (0.84, 1.21)	0.83 (0.65, 1.06)	1.29 (0.95, 1.75)	1.02 (0.96, 1.09)	0.53
HPFS						
Number of cases/person-year	1,417/314,283	1,463/335,363	914/195,157	388/80,782		
Rate per 100,000 person-years	451	436	468	480		
Multivariable adjusted HR[b]	1	1.01 (0.93, 1.09)	1.07 (0.98, 1.17)	1.02 (0.91, 1.16)	1.00 (0.98, 1.03)	0.27
Pooled results[c]	1	1.00 (0.95, 1.05)	1.04 (0.98, 1.11)	1.04 (0.95, 1.14)	1.01 (0.99, 1.03)	0.16

Notes: [a]Total cruciferous vegetables included broccoli, cabbage, cauliflower, Brussels sprouts, kale, mustard, and chard greens. [b]Estimates are calculated in Cox proportional hazards models. Adjusted for age (years), ethnicity (Caucasian, African American, Asian, and other ethnicity), family history of myocardial infarction(yes/no), smoking status (never, former, current [1–14, 15–24, or ≥25 cigarettes/day], or missing), alcohol intake (0, 0.1–4.9, 5.0–14.9, and >15.0 g/day in women, 0, 0.1–4.9, 5.0–29.9, and >30.0 g/day in men, or missing), hypertension (yes/no), hypercholesterolemia (yes/no), (never, former, current [1–14, 15–24, or ≥25 cigarettes/day], or missing), alcohol intake (0, 0.1–4.9, 5.0–14.9, and ≥15.0 g/day for women, 0, 0.1–4.9, 5.0–29.9, and ≥30.0 g/day for men, or missing), physical activity (<3.0, 3.0–8.9, 9.0–17.9, 18.0–26.9, ≥27.0 MET-hours/week, or missing), menopausal status and postmenopausal hormone use (premenopause, postmenopause [never, former, or current hormone use], or missing, for women), oral contraceptive use (yes, no, or missing, for NHSII), multivitamin use (yes/no), BMI (<23, 23–24.9, 25–29.9, 30–34.9, ≥35 kg/m², or missing), and total energy intake (kcal/day), and the modified Alternate Healthy Eating Index score (quintiles). [c]Results from each cohort were pooled using fixed-effects model.
Abbreviations: BMI, body mass index; HPFS, Health Professionals Follow-Up Study; HR, hazard ratio; MET, metabolic equivalents of task; NHS, Nurses' Health Study.

factor erythroid-2-related factor 2-regulated transcription.[32] ITCs could also modulate cytokine production and inhibit the lipopolysaccharide-stimulated inflammatory response in human monocytes.[33] These lines of evidence constitute the knowledge base for us to hypothesize that glucosinolate intake is associated with a lower CHD risk. However, the findings of the current investigation are contradictory to our initial hypothesis.

Indeed, emerging evidence suggests that the health effects of ITCs and other glucosinolates can be complex. Administration of glucosinolates and their degradation products induces the activities of certain Phase I enzymes with an influence on the metabolism of xenobiotics and on the generation of reactive oxygen species (ROS) in vitro.[34] Cumulatively, elevated ROS production may accelerate decline in cardiomyocyte function and progression to CHD.[35,36] Such pro-oxidant activity of ITCs is one of the mechanisms underlying ITCs' potentially anticarcinogenic role, because the variation of the intracellular redox status triggers apoptosis and other defensive mechanisms.[37] ITCs may also undergo oxidative desulfuration to produce the corresponding isocyanate by cytochrome P450 enzymes.[38] Moreover, glucosinolate hydrolysis products could rapidly accumulate in the cytoplasm of the cells, bind to glutathione and other cellular thiols, and react with the SH groups, which leads to intracellular glutathione depletion and subsequent ROS generation.[39,40] In addition to depleting glutathione and other thiols, the breakdown products of glucosinolates can also enhance the cellular concentration of ROS and oxidative stress by inducing rapid loss of transmembrane potential, mitochondrial damage, and loss of cytochrome *c*.[41,42] Glutathione depletion was also found to significantly accelerate ITC-triggered apoptosis through a mitochondrial redox-sensitive mechanism.[42,43] As other products of glucosinolate hydrolysis, nitriles also have a potential to induce cytotoxicity and genotoxicity.[44]

The potentially complicated biological effects of glucosinolates and their metabolites are also suggested by mixed evidence from human trials.[13,14,45,46] In a 12-week intervention study among participants with elevated risk of developing CVD, supplementation with 400 g high-glucosinolate broccoli per week led to significant reduction of plasma low-density lipoprotein-C level.[13] In a randomized double-blind clinical trial among diabetes patients, Mirmiran et al observed beneficial effects of 10 g/day broccoli sprouts powder on serum interleukin-6 and C-reactive protein levels, but not on tumor necrosis factor α.[45] In contrast, among individuals with moderate risk for the development of CVD, supplementation with broccoli did not exert significant changes in CVD risk markers.[46] In patients with established hypertension, a 4-week treatment with dried broccoli sprouts did not exert any significant effect on serum cholesterol levels and endothelial function measured by flow-mediated dilation.[14]

To our knowledge, the current study is the first prospective investigation that assessed the relationship between dietary glucosinolates and CHD risk. Of note, previous studies that focused on cruciferous vegetable intake in relation to CHD risk overall demonstrated no associations between the consumption of these vegetables and CHD risk.[11,12,47] In the prospective Danish Diet, Cancer and Health cohort study, increasing consumption of total vegetables and cruciferous vegetables was not significantly associated with the risk of acute coronary syndrome after multivariable adjustment.[11] Similarly, Genkinger et al also found no inverse association between dietary intake of cruciferous vegetables and CVD mortality in a community-based prospective cohort study.[47]

The strengths of this study include the prospective design, the large sample size, long follow-up durations, detailed and repeated dietary and lifestyle assessments, and high rates of follow-up. There are several potential limitations that also need to be considered. First, some measurement errors and misclassification in the assessment of food consumption are inevitable, although the FFQs used in these cohorts have been validated against multiple diet records and demonstrated reasonable reproducibility and validity. Because of the prospective study design, misclassification of glucosinolate intake was unlikely to be correlated with study outcome ascertainment and, therefore, more likely to attenuate associations toward the null. Second, although we controlled for a large number of potential dietary and lifestyle factors in multivariate models, it is possible that residual and unmeasured confounding may still remain. Third, several factors, such as cooking methods, storage time, and temperature, can determine the activities of myrosinase and subsequently influence the bioavailability of glucosinolates and the production of breakdown products. Although the urinary excretion of ITCs was correlated significantly with cruciferous vegetable or glucosinolate consumption, potentially large between-individual variability in the production of ITCs upon the intake of the same food sources may render our observations less extrapolatable to ITCs.[48–50] Future studies should examine circulating levels of ITCs in relation to chronic disease risk to provide evidence complementary to research on glucosinolate intake. Fourth, a plant-based diet may contain a variety of secondary plant metabolites, including glucosinolates, polyphenols, and other phytonutrients. It is very likely that these phytochemicals may have additive or synergistic effects on modulating human health beyond the effects of a specific group of phytochemicals, although the current analysis was unable to explore this possibility which would require a larger study population for detecting interactions between dietary components. Finally, participants in our study are mostly health care professionals of European ancestry. Our ethnicity-stratified analysis implied that the positive association between glucosinolate intake and CHD risk was primarily observed in white participants,

whereas the association was entirely absent in minorities. It is likely that the effects of glucosinolate intake may be modulated by variabilities in genes operating in the complex biological pathways of glucosinolates and their products. For example, the association between low cruciferous vegetable intake and breast cancer risk appeared to be somewhat more pronounced among Chinese women with the Val/Val genotype in *GSTP1* gene,[51] which encodes glutathione *S*-transferases, enzymes involved in the biological effects of ITCs.[52] Despite this plausibility, we cannot exclude the role of chance in this finding of interaction by ethnicity. Nonetheless, caution must be taken when extrapolating the current findings to other ethnic groups.

In conclusion, our data do not support the hypothesis that a higher glucosinolate intake decreases the risk of CHD. In contrast, our findings suggest that a higher glucosinolate intake may be associated with a small increment of CHD risk among US men and women. Given the observational nature of the current analysis and the complex metabolism and biology of glucosinolates, future studies are warranted to replicate these findings and elucidate the mechanistic pathways linking glucosinolates, ITCs, and cardiovascular health.

Key points

Question: What is the association of dietary glucosinolate intake with incident coronary heart disease (CHD) in US adults?

Findings: In three cohorts of US men and women, the intake of total and subtypes of glucosinolates, as well as cruciferous vegetables, was associated with a slightly increased risk of developing CHD. These associations were independent of established and potential confounders of CHD and persistent in various sensitivity analyses.

Meaning: Our findings highlight the potentially complicated biological effects of glucosinolate intake on human health.

Acknowledgments

This work was supported by the National Institutes of Health (CA186107, HL034594, CA176726, CA167552, and HL35464). The study sponsor had no role in the design and conduct of the study; the collection, management, analysis, or interpretation of the data; the preparation, review, or approval of the paper; or the decision to submit the paper for publication.

Author contributions

QS and LM participated in project conception and development of research methods; QS, FBH, WCW, EBR, KMR, EBR, and JEM obtained funding and provided oversight; LM, GL, GZ, and QS analyzed data and performed the analysis; LM drafted the paper. All authors contributed toward data analysis, drafting and revising the paper and agree to be accountable for all aspects of the work.

Disclosure

The authors report no conflicts of interest in this work.

References

1. Van Horn L, Carson JA, Appel LJ, et al. Recommended dietary pattern to achieve adherence to the American Heart Association/American College of Cardiology (AHA/ACC) Guidelines: a Scientific Statement From the American Heart Association. *Circulation.* 2016;134(22):e505–e529.
2. US Department of Health and Human Services and US Department of Agriculture. *2015–2020 Dietary Guidelines for Americans*. 8th ed. December 2015. Available from: http://health.gov/dietaryguidelines/2015/guidelines/. Accessed October 27, 2017.
3. Bhupathiraju SN, Wedick NM, Pan A, et al. Quantity and variety in fruit and vegetable intake and risk of coronary heart disease. *Am J Clin Nutr.* 2013;98(6):1514–1523.
4. Oude Griep LM, Verschuren WM, Kromhout D, Ocké MC, Geleijnse JM. Colours of fruit and vegetables and 10-year incidence of CHD. *Br J Nutr.* 2011;106(10):1562–1569.
5. Thomson CA, Ho E, Strom MB. Chemopreventive properties of 3,3′-diindolylmethane in breast cancer: evidence from experimental and human studies. *Nutr Rev.* 2016;74(7):432–434.
6. Herr I, Büchler MW. Dietary constituents of broccoli and other cruciferous vegetables: implications for prevention and therapy of cancer. *Cancer Treat Rev.* 2010;36(5):377–383.
7. Fofaria NM, Ranjan A, Kim SH, Srivastava SK. Mechanisms of the anticancer effects of isothiocyanates. *Enzymes.* 2015;37:111–137.
8. Jang M, Cho IH. Sulforaphane ameliorates 3-nitropropionic acid-induced striatal toxicity by activating the keap1-Nrf2-ARE pathway and inhibiting the MAPKs and NF-κB pathways. *Mol Neurobiol.* 2016;53(4):2619–2635.
9. Kwon JS, Joung H, Kim YS, et al. Sulforaphane inhibits restenosis by suppressing inflammation and the proliferation of vascular smooth muscle cells. *Atherosclerosis.* 2012;225(1):41–49.
10. Carrasco-Pozo C, Tan KN, Gotteland M, Borges K. Sulforaphane protects against high cholesterol-induced mitochondrial bioenergetics impairments, inflammation, and oxidative stress and preserves pancreatic β-cells function. *Oxid Med Cell Longev.* 2017;2017:3839756.
11. Hansen L, Dragsted LO, Olsen A, et al. Fruit and vegetable intake and risk of acute coronary syndrome. *Br J Nutr.* 2010;104(2):248–255.
12. Cornelis MC, El-Sohemy A, Campos H. GSTT1 genotype modifies the association between cruciferous vegetable intake and the risk of myocardial infarction. *Am J Clin Nutr.* 2007;86(3):752–758.
13. Armah CN, Derdemezis C, Traka MH, et al. Diet rich in high glucoraphanin broccoli reduces plasma LDL cholesterol: evidence from randomised controlled trials. *Mol Nutr Food Res.* 2015;59(5):918–926.
14. Christiansen B, Bellostas Muguerza N, Petersen AM, et al. Ingestion of broccoli sprouts does not improve endothelial function in humans with hypertension. *PLoS One.* 2010;5(8):e12461.
15. Mozaffarian D, Hao T, Rimm EB, Willett WC, Hu FB. Changes in diet and lifestyle and long-term weight gain in women and men. *N Engl J Med.* 2011;364(25):2392–2404.
16. Steinbrecher A, Linseisen J. Dietary intake of individual glucosinolates in participants of the EPIC-Heidelberg cohort study. *Ann Nutr Metab.* 2009;54(2):87–96.
17. Salvini S, Hunter DJ, Sampson L, et al. Food-based validation of a dietary questionnaire: the effects of week-to-week variation in food consumption. *Int J Epidemiol.* 1989;18(4):858–867.

18. Feskanich D, Rimm EB, Giovannucci EL, et al. Reproducibility and validity of food intake measurements from a semiquantitative food frequency questionnaire. *J Am Diet Assoc.* 1993;93(7):790–796.
19. Hu FB, Rimm E, Smith-Warner SA, et al. Reproducibility and validity of dietary patterns assessed with a food-frequency questionnaire. *Am J Clin Nutr.* 1999;69(2):243–249.
20. Rimm EB, Stampfer MJ, Colditz GA, Chute CG, Litin LB, Willett WC. Validity of self-reported waist and hip circumferences in men and women. *Epidemiology.* 1990;1(6):466–473.
21. Giovannucci E, Colditz G, Stampfer MJ, et al. The assessment of alcohol consumption by a simple self-administered questionnaire. *Am J Epidemiol.* 1991;133(8):810–817.
22. Wolf AM, Hunter DJ, Colditz GA, et al. Reproducibility and validity of a self-administered physical activity questionnaire. *Int J Epidemiol.* 1994;23(5):991–999.
23. Wang T, Heianza Y, Sun D, et al. Improving adherence to healthy dietary patterns, genetic risk, and long term weight gain: gene-diet interaction analysis in two prospective cohort studies. *BMJ*. 2018;360:j5644.
24. Chiuve SE, Fung TT, Rimm EB, et al. Alternative dietary indices both strongly predict risk of chronic disease. *J Nutr*. 2012;142(6):1009–1018.
25. Rose GA. *Cardiovascular Survey Methods*. Geneva Albany, NY: World Health Organization; WHO Publications Centre distributor; 1982.
26. Wu H, Ding EL, Toledo ET, et al. A novel fatty acid lipophilic index and risk of CHD in US men: the health professionals follow-up study. *Br J Nutr.* 2013;110(3):466–474.
27. Hu FB, Stampfer MJ, Rimm E, et al. Dietary fat and coronary heart disease: a comparison of approaches for adjusting for total energy intake and modeling repeated dietary measurements. *Am J Epidemiol.* 1999;149(6):531–540.
28. Angelino D, Dosz EB, Sun J, et al. Myrosinase-dependent and -independent formation and control of isothiocyanate products of glucosinolate hydrolysis. *Front Plant Sci.* 2015;6:831.
29. Hanschen FS, Bauer A, Mewis I, et al. Thermally induced degradation of aliphatic glucosinolates: identification of intermediary breakdown products and proposed degradation pathways. *J Agric Food Chem.* 2012;60(39):9890–9899.
30. Agerbirk N, Olsen CE. Glucosinolate hydrolysis products in the crucifer Barbarea vulgaris include a thiazolidine-2-one from a specific phenolic isomer as well as oxazolidine-2-thiones. *Phytochemistry.* 2015;115:143–151.
31. Márton MR, Krumbein A, Platz S, et al. Determination of bioactive, free isothiocyanates from a glucosinolate-containing phytotherapeutic agent: a pilot study with in vitro models and human intervention. *Fitoterapia.* 2013;85:25–34.
32. Krajka-Kuźniak V, Paluszczak J, Szaefer H, Baer-Dubowska W. The activation of the Nrf2/ARE pathway in HepG2 hepatoma cells by phytochemicals and subsequent modulation of phase II and antioxidant enzyme expression. *J Physiol Biochem.* 2015;71(2):227–238.
33. Reddy SA, Shelar SB, Dang TM, et al. Sulforaphane and its methylcarbonyl analogs inhibit the LPS-stimulated inflammatory response in human monocytes through modulating cytokine production, suppressing chemotactic migration and phagocytosis in a NF-κB- and MAPK-dependent manner. *Int Immunopharmacol.* 2015;24(2):440–450.
34. Kadir NH, David R, Rossiter JT, Gooderham NJ. The selective cytotoxicity of the alkenyl glucosinolate hydrolysis products and their presence in Brassica vegetables. *Toxicology.* 2015;334:59–71.
35. Watt J, Ewart MA, Greig FH, Oldroyd KG, Wadsworth RM, Kennedy S. The effect of reactive oxygen species on whole blood aggregation and the endothelial cell-platelet interaction in patients with coronary heart disease. *Thromb Res.* 2012;130(2):210–215.
36. Chen X, Niroomand F, Liu Z, et al. Expression of nitric oxide related enzymes in coronary heart disease. *Basic Res Cardiol.* 2006;101(4):346–353.
37. Wang L, Tian Z, Yang Q, et al. Sulforaphane inhibits thyroid cancer cell growth and invasiveness through the reactive oxygen species-dependent pathway. *Oncotarget.* 2015;6(28):25917–25931.
38. Lee MS. Enzyme induction and comparative oxidative desulfuration of isothiocyanates to isocyanates. *Chem Res Toxicol.* 1996;9(7):1072–1078.
39. Valgimigli L, Iori R. Antioxidant and pro-oxidant capacities of ITCs. *Environ Mol Mutagen.* 2009;50(3):222–237.
40. Øverby A, Stokland RA, Åsberg SE, Sporsheim B, Bones AM. Allyl isothiocyanate depletes glutathione and upregulates expression of glutathione S-transferases in Arabidopsis thaliana. *Front Plant Sci.* 2015;6:277.
41. Sehrawat A, Croix CS, Baty CJ, et al. Inhibition of mitochondrial fusion is an early and critical event in breast cancer cell apoptosis by dietary chemopreventative benzyl isothiocyanate. *Mitochondrion.* 2016;30:67–77.
42. Nakamura Y, Kawakami M, Yoshihiro A, et al. Involvement of the mitochondrial death pathway in chemopreventive benzyl isothiocyanate-induced apoptosis. *J Biol Chem.* 2002;277(10):8492–899.
43. Zhang T, Shao Y, Chu TY, et al. MiR-135a and MRP1 play pivotal roles in the selective lethality of phenethyl isothiocyanate to malignant glioma cells. *Am J Cancer Res.* 2016;6(5):957–972.
44. Kupke F, Herz C, Hanschen FS, et al. Cytotoxic and genotoxic potential of food-borne nitriles in a liver in vitro model. *Sci Rep.* 2016;6:37631.
45. Mirmiran P, Bahadoran Z, Hosseinpanah F, Keyzad A, Azizi F. Effects of broccoli sprout with high sulforaphane concentration on inflammatory markers in type 2 diabetic patients: a randomized double-blind placebo-controlled clinical trial. *J Func Foods.* 2012;4(4):837–841.
46. Armah CN, Traka MH, Dainty JR, et al. A diet rich in high-glucoraphanin broccoli interacts with genotype to reduce discordance in plasma metabolite profiles by modulating mitochondrial function. *Am J Clin Nutr.* 2013;98(3):712–722.
47. Genkinger JM, Platz EA, Hoffman SC, Comstock GW, Helzlsouer KJ. Fruit, vegetable, and antioxidant intake and all-cause, cancer, and cardiovascular disease mortality in a community-dwelling population in Washington County, Maryland. *Am J Epidemiol.* 2004;160(12):1223–1233.
48. Kristensen M, Krogholm KS, Frederiksen H, Bügel SH, Rasmussen SE. Urinary excretion of total isothiocyanates from cruciferous vegetables shows high dose-response relationship and may be a useful biomarker for isothiocyanate exposure. *Eur J Nutr.* 2007;46(7):377–382.
49. Seow A, Shi CY, Chung FL, et al. Urinary total isothiocyanate (ITC) in a population-based sample of middle-aged and older Chinese in Singapore: relationship with dietary total ITC and glutathione S-transferase M1/T1/P1 genotypes. *Cancer Epidemiol Biomarkers Prev.* 1998;7(9):775–781.
50. Oliviero T, Verkerk R, Vermeulen M, Dekker M. In vivo formation and bioavailability of isothiocyanates from glucosinolates in broccoli as affected by processing conditions. *Mol Nutr Food Res.* 2014;58(7):1447–1456.
51. Lee SA, Fowke JH, Lu W, et al. Cruciferous vegetables, the GSTP1 Ile105Val genetic polymorphism, and breast cancer risk. *Am J Clin Nutr*. 2008;87(3):753–760.
52. Shapiro TA, Fahey JW, Dinkova-Kostova AT, et al. Safety, tolerance, and metabolism of broccoli sprout glucosinolates and isothiocyanates: a clinical phase I study. *Nutr Cancer*. 2006;55(1):53–62.

Supplementary materials

Table S1 Stratified incidence density of coronary heart disease according to total glucosinolate intake by various characteristics of participants

Variables	Quintiles of total glucosinolate intake				
	1 (low)	2	3	4	5 (high)
Race					
Caucasians					
Number of cases/person-year	1,699/945,169	1,516/945,577	1,529/944,642	1,622/940,917	1,730/930,405
Rate per 100,000 person-years	180	160	162	172	186
Other races					
Number of cases/person-year	47/30,953	31/32,364	47/33,939	60/37,056	76/46,461
Rate per 100,000 person-years	152	96	138	162	164
Age (years)					
<55					
Number of cases/person-year	229/505,930	218/496,956	191/486,075	195/475,685	217/452,437
Rate per 100,000 person-years	45	44	39	41	48
55–65					
Number of cases/person-year	416/238,154	415/248,691	420/251,803	437/256,379	428/263,890
Rate per 100,000 person-years	175	167	167	170	162
65–75					
Number of cases/person-year	546/149,876	472/153,545	506/159,670	532/163,449	594/172,029
Rate per 100,000 person-years	364	307	317	325	345
≥75					
Number of cases/person-year	555/82,065	442/78,614	459/80,902	518/82,313	567/88,347
Rate per 100,000 person-years	676	562	567	629	642
BMI (kg/m^2)					
<30					
Number of cases/person-year	1,394/787,946	1,206/794,488	1,240/794,357	1,327/784,483	1,369/760,442
Rate per 100,000 person-years	177	152	156	169	180
≥30					
Number of cases/person-year	347/183,344	339/178,732	325/179,533	347/188,850	429/211,045
Rate per 100,000 person-years	189	190	181	187	203
Modified AHEI score					
<Median level					
Number of cases/person-year	916/516,984	610/381,223	549/308,146	416/235,340	297/137,629
Rate per 100,000 person-years	177	160	178	177	216
≥Median level					
Number of cases/person-year	750/443,390	893/580,826	980/654,476	1,209/726,799	1,458/823,343
Rate per 100,000 person-years	169	154	150	166	177
Physical activity (METs-hour/week)					
<Median level					
Number of cases/person-year	1,039/533,922	904/491,541	879/465,121	914/439,758	957/419,322
Rate per 100,000 person-years	195	184	189	208	228
≥Median level					
Number of cases/person-year	667/425,086	616/471,283	665/500,235	742/523,339	820/540,135
Rate per 100,000 person-years	157	131	133	142	152
Smoking status					
Never					
Number of cases/person-year	1,447/873,732	1,305/886,590	1,336/886,751	1,461/888,765	1,566/887,533
Rate per 100,000 person-years	166	147	151	164	176
Ever					
Number of cases/person-year	299/102,390	242/91,351	240/91,829	221/89,208	240/89,332
Rate per 100,000 person-years	292	265	261	248	269
Alcohol consumption					
Never					
Number of cases/person-year	773/407,410	646/357,427	606/343,936	634/331,461	745/354,692
Rate per 100,000 person-years	190	181	176	191	210
Ever					
Number of cases/person-year	973/568,712	901/620,514	970/634,644	1,048/646,513	1,061/622,173
Rate per 100,000 person-years	171	145	158	162	171

Abbreviations: AHEI, Alternative Healthy Eating Index; BMI, body mass index; MET, metabolic equivalents of task.

Table S2 Stratified hazard ratio (95% CI) of coronary heart disease according to total glucosinolate intake by various characteristics of participants[a]

Variables	Quintiles of total glucosinolate intake					P_{trend}	$P_{interaction}$[b]
	1 (low)	2	3	4	5 (high)		
Race							>0.99
Caucasians	1	0.97 (0.90, 1.04)	0.99 (0.92, 1.06)	1.05 (0.98, 1.13)	1.09 (1.01, 1.17)	0.001	
Other races	1	0.63 (0.39, 1.02)	0.85 (0.54, 1.34)	0.98 (0.64, 1.51)	0.95 (0.62, 1.46)	0.34	
Age (years)							0.77
<65	1	1.03 (0.91, 1.17)	1.07 (0.94, 1.21)	1.13 (0.99, 1.28)	1.12 (0.98, 1.28)	0.06	
≥65	1	0.91 (0.83, 0.99)	0.95 (0.87, 1.04)	1.03 (0.94, 1.12)	1.06 (0.97, 1.16)	0.01	
BMI (kg/m^2)							0.58
<30	1	1.00 (0.99, 1.01)	0.94 (0.87, 1.02)	0.98 (0.91, 1.06)	1.06 (0.98, 1.15)	0.003	
≥30	1	1.03 (0.88, 1.20)	0.99 (0.85, 1.15)	1.00 (0.86, 1.16)	1.11 (0.95, 1.29)	0.20	
Modified AHEI score							0.57
<Median level	1	0.94 (0.85, 1.04)	1.00 (0.90, 1.11)	1.00 (0.89, 1.12)	1.11 (0.97, 1.27)	0.12	
≥Median level	1	1.02 (0.93, 1.13)	1.02 (0.93, 1.12)	1.12 (1.02, 1.23)	1.14 (1.05, 1.25)	<0.001	
Physical activity							0.33
<Median level	1	1.02 (0.93, 1.11)	1.03 (0.94, 1.12)	1.11 (1.01, 1.21)	1.13 (1.03, 1.24)	0.005	
≥Median level	1	0.89 (0.80, 0.99)	0.93 (0.84, 1.04)	1.00 (0.90, 1.11)	1.05 (0.94, 1.17)	0.04	
Smoking status							0.50
Never	1	0.96 (0.89, 1.03)	0.99 (0.91, 1.06)	1.07 (0.99, 1.15)	1.09 (1.01, 1.18)	0.001	
Ever	1	0.95 (0.80, 1.13)	0.97 (0.82, 1.16)	0.96 (0.80, 1.15)	1.07 (0.89, 1.29)	0.36	
Alcohol consumption							0.91
Never	1	1.01 (0.91, 1.13)	0.99 (0.89, 1.11)	1.05 (0.94, 1.17)	1.10 (0.99, 1.23)	0.06	
Ever	1	0.92 (0.84, 1.01)	0.98 (0.89, 1.07)	1.06 (0.97, 1.16)	1.08 (0.98, 1.18)	0.004	

Notes: [a]Estimates are calculated in Cox proportional hazards models, adjusted for age (years), ethnicity (Caucasian, African American, Asian, and other ethnicity), family history of myocardial infarction (yes/no), hypertension (yes/no), hypercholesterolemia (yes/no), smoking status (never, former, current [1–14, 15–24, or ≥25 cigarettes/day], or missing), alcohol intake (0, 0.1–4.9, 5.0–14.9, and ≥15.0 g/day for women, 0, 0.1–4.9, 5.0–29.9, and ≥30.0 g/day for men, or missing), physical activity (<3.0, 3.0–8.9, 9.0–17.9, 18.0–26.9, ≥27.0 MET-hours/week, or missing), menopausal status and postmenopausal hormone use (premenopause, postmenopause [never, former, or current hormone use], or missing, for women), oral contraceptive use (yes, no, or missing, for Nurses' Health Study II), multivitamin use (yes/no), BMI (<23, 23–24.9, 25–29.9, 30–34.9, ≥35 kg/m^2, or missing), and total energy intake (kcal/day), and the modified AHEI score (quintiles). [b]$P_{interaction}$ was calculated using the likelihood ratio test.
Abbreviations: AHEI, Alternative Healthy Eating Index; BMI, body mass index; MET, metabolic equivalents of task.

Table S3 Sensitivity analyses for the association between total glucosinolate intake and coronary heart disease in three cohorts[a]

Variables	Quintiles of intake					P_{trend}	Every SD increment
	1 (low)	2	3	4	5 (high)		
Using baseline glucosinolate intake as an exposure	1	0.93 (0.86, 0.99)	0.96 (0.89, 1.03)	1.03 (0.96, 1.11)	1.04 (0.97, 1.12)	0.01	1.03 (1.01, 1.05)
Adjustment for major dietary factors instead of modified AHEI score	1	0.95 (0.89, 1.02)	0.98 (0.91, 1.05)	1.04 (0.97, 1.12)	1.06 (0.98, 1.14)	0.02	1.02 (0.99, 1.04)
Continuing updating diet after diagnosis of cardiovascular disease or cancer	1	0.96 (0.90, 1.03)	1.01 (0.94, 1.08)	1.06 (0.99, 1.14)	1.10 (1.02, 1.18)	<0.001	1.03 (1.00, 1.05)
Using a 4-year lag period	1	0.94 (0.87, 1.01)	0.99 (0.92, 1.06)	1.06 (0.98, 1.14)	1.06 (0.99, 1.15)	0.008	1.03 (1.01, 1.05)
Using an 8-year lag period	1	0.94 (0.87, 1.02)	0.98 (0.91, 1.06)	1.04 (0.96, 1.13)	1.05 (0.97, 1.14)	0.03	1.03 (1.00, 1.05)

Notes: [a]Estimates are calculated in Cox proportional hazards models, adjusted for age (years), ethnicity (Caucasian, African American, Asian, and other ethnicity), family history of myocardial infarction (yes/no), hypertension (yes/no), hypercholesterolemia (yes/no), smoking status (never, former, current [1–14, 15–24, or ≥25 cigarettes/day], or missing), alcohol intake (0, 0.1–4.9, 5.0–14.9, and ≥15.0 g/day for women, 0, 0.1–4.9, 5.0–29.9, and ≥30.0 g/day for men, or missing), physical activity (<3.0, 3.0–8.9, 9.0–17.9, 18.0–26.9, ≥27.0 MET-hours/week, or missing), menopausal status and postmenopausal hormone use (premenopause, postmenopause [never, former, or current hormone use], or missing, for women), oral contraceptive use (yes, no, or missing, for Nurses' Health Study II), multivitamin use (yes/no), BMI (<23, 23–24.9, 25–29.9, 30–34.9, ≥35 kg/m^2, or missing), and total energy intake (kcal/day), and the modified AHEI score (quintiles).
Abbreviations: AHEI, Alternative Healthy Eating Index; BMI, body mass index; MET, metabolic equivalents of task.

Table S4 HR (95% CI) of coronary heart disease according to quintiles of main individual glucosinolates[a]

	Quintiles of intake					P_{trend}
	**1 (low)**	**2**	**3**	**4**	**5 (high)**	
Glucobrassicin						
NHS	1	0.96 (0.86, 1.07)	1.06 (0.96, 1.18)	1.09 (0.98, 1.21)	1.09 (0.98, 1.22)	0.03
NHSII	1	1.03 (0.81, 1.32)	0.88 (0.68, 1.13)	1.05 (0.82, 1.34)	1.06 (0.83, 1.35)	0.57
HPFS	1	0.92 (0.83, 1.01)	0.94 (0.85, 1.04)	1.02 (0.93, 1.13)	1.01 (0.91, 1.11)	0.28
Pooled results[b]	1	0.95 (0.88, 1.01)	0.99 (0.92, 1.06)	1.05 (0.98, 1.13)	1.05 (0.97, 1.12)	0.02
Sinigrin						
NHS	1	1.04 (0.93, 1.17)	1.09 (0.97, 1.22)	1.15 (1.03, 1.28)	1.35 (1.21, 1.51)	<0.001
NHSII	1	1.21 (0.94, 1.56)	1.20 (0.93, 1.55)	1.16 (0.89, 1.51)	1.28 (1.00, 1.65)	0.13
HPFS	1	0.96 (0.87, 1.06)	0.93 (0.84, 1.03)	0.98 (0.89, 1.08)	1.05 (0.96, 1.16)	0.09
Pooled results[b]	1	1.01 (0.94, 1.09)	1.01 (0.94, 1.09)	1.06 (0.99, 1.14)	1.19 (1.11, 1.27)	<0.001
Glucoraphanin						
NHS	1	1.00 (0.90, 1.10)	1.05 (0.94, 1.16)	0.89 (0.79, 0.99)	0.93 (0.83, 1.04)	0.04
NHSII	1	0.95 (0.75, 1.20)	0.95 (0.75, 1.21)	0.81 (0.62, 1.05)	1.00 (0.78, 1.27)	0.99
HPFS	1	0.92 (0.84, 1.01)	0.97 (0.88, 1.07)	0.96 (0.87, 1.06)	1.02 (0.92, 1.12)	0.33
Pooled results[b]	1	0.96 (0.89, 1.02)	1.00 (0.93, 1.07)	0.92 (0.86, 0.99)	0.98 (0.91, 1.05)	0.64
Glucoiberin						
NHS	1	0.98 (0.87, 1.09)	1.08 (0.97, 1.20)	1.05 (0.94, 1.18)	1.19 (1.06, 1.32)	<0.001
NHSII	1	1.12 (0.88, 1.42)	0.91 (0.70, 1.18)	0.97 (0.75, 1.26)	1.20 (0.94, 1.53)	0.16
HPFS	1	0.94 (0.86, 1.04)	0.97 (0.88, 1.07)	1.04 (0.94, 1.14)	0.99 (0.90, 1.10)	0.55
Pooled results[b]	1	0.97 (0.91, 1.04)	1.01 (0.94, 1.08)	1.04 (0.97, 1.11)	1.09 (1.01, 1.17)	0.004
Neoglucobrassicin						
NHS	1	1.00 (0.91, 1.11)	1.05 (0.95, 1.17)	0.90 (0.81, 1.01)	0.95 (0.85, 1.06)	0.09
NHSII	1	1.09 (0.87, 1.38)	0.97 (0.76, 1.24)	0.83 (0.63, 1.08)	1.05 (0.82, 1.34)	0.97
HPFS	1	0.94 (0.85, 1.03)	0.96 (0.87, 1.05)	0.97 (0.88, 1.07)	1.02 (0.92, 1.12)	0.34
Pooled results[b]	1	0.98 (0.91, 1.05)	1.00 (0.93, 1.07)	0.93 (0.87, 1.00)	0.99 (0.92, 1.06)	0.76

Notes: [a]Estimates are calculated in Cox proportional hazards models, adjusted for age (years), ethnicity (Caucasian, African American, Asian, and other ethnicity), family history of myocardial infarction (yes/no), hypertension (yes/no), hypercholesterolemia (yes/no), smoking status (never, former, current [1–14, 15–24, or ≥25 cigarettes/day], or missing), alcohol intake (0, 0.1–4.9, 5.0–14.9, and ≥15.0 g/day for women, 0, 0.1–4.9, 5.0–29.9, and ≥30.0 g/day for men, or missing), physical activity (<3.0, 3.0–8.9, 9.0–17.9, 18.0–26.9, ≥27.0 MET-hours/week, or missing), menopausal status and postmenopausal hormone use (premenopause, postmenopause [never, former, or current hormone use], or missing, for women), oral contraceptive use (yes, no, or missing, for NHSII), multivitamin use (yes/no), BMI (<23, 23–24.9, 25–29.9, 30–34.9, ≥35 kg/m^2, or missing), and total energy intake (kcal/day), and the modified Alternate Healthy Eating Index score (quintiles). [b]Results from each cohort were pooled using fixed-effects model.
Abbreviations: BMI, body mass index; HPFS, Health Professionals Follow-Up Study; HR, hazard ratio; MET, metabolic equivalents of task; NHS, Nurses' Health Study.

Table S5 Hazard ratio (95% CI) of coronary heart disease according to consumption levels of individual cruciferous vegetables[a]

**Variables and Cohort**	**Consumption levels**				**Every two servings/week**	P_{trend}
	**1 (low)**	**2**	**3**	**4 (high)**		
Broccoli						
Consumption level	<1 serving/week	1–2 servings/week	2–3 servings/week	≥4 servings/week		
NHS	1	0.99 (0.92, 1.07)	0.93 (0.85, 1.02)	1.13 (0.90, 1.43)	0.97 (0.90, 1.04)	0.53
NHSII	1	1.01 (0.84, 1.21)	0.93 (0.75, 1.15)	1.29 (0.84, 1.97)	1.03 (0.91, 1.16)	0.84
HPFS	1	1.01 (0.93, 1.08)	1.05 (0.96, 1.14)	0.92 (0.74, 1.15)	1.01 (0.96, 1.07)	0.70
Pooled results[b]	1	1.00 (0.95, 1.05)	0.99 (0.93, 1.05)	1.05 (0.90, 1.22)	1.02 (0.97, 1.07)	0.95
Cabbage						
Consumption level	<1 serving/month	1–2 servings/month	2–4 servings/month	≥1 serving/week		
NHS	1	1.07 (0.96, 1.19)	1.00 (0.90, 1.12)	1.20 (1.08, 1.33)	1.09 (1.00, 1.19)	<0.001
NHSII	1	1.01 (0.84, 1.22)	0.86 (0.63, 1.19)	1.16 (0.93, 1.45)	1.14 (0.95, 1.37)	0.18
HPFS	1	1.06 (0.94, 1.20)	1.03 (0.89, 1.18)	1.01 (0.92, 1.11)	1.01 (0.99, 1.02)	0.71
Pooled results[b]	1	1.06 (0.98, 1.14)	1.00 (0.92, 1.09)	1.09 (1.02, 1.17)	1.02 (1.00, 1.03)	0.02
Cauliflower						
Consumption level	<1 serving/month	1–2 servings/month	2–4 servings/month	≥1 serving/week		
NHS	1	1.10 (1.00, 1.21)	0.93 (0.84, 1.03)	1.07 (0.97, 1.18)	1.02 (0.94, 1.10)	0.63
NHSII	1	1.02 (0.84, 1.24)	1.10 (0.82, 1.47)	0.97 (0.79, 1.20)	1.03 (0.87, 1.22)	0.71
HPFS	1	0.99 (0.91, 1.08)	1.00 (0.91, 1.11)	1.07 (0.98, 1.16)	1.03 (0.96, 1.10)	0.09
Pooled results[b]	1	1.04 (0.98, 1.10)	0.98 (0.91, 1.05)	1.06 (0.99, 1.13)	1.02 (0.97, 1.08)	0.16

(*Continued*)

Table S5 (*Continued*)

Variables and Cohort	Consumption levels				Every two servings/week	P_{trend}
	1 (low)	2	3	4 (high)		
Brussels sprouts						
Consumption level	<1 serving/month	1–2 servings/month	2–4 servings/month	≥1 serving/week		
NHS	1	1.24 (1.14, 1.35)	1.10 (0.98, 1.25)	1.38 (1.22, 1.57)	1.34 (1.19, 1.50)	<0.001
NHSII	1	1.15 (0.94, 1.40)	1.04 (0.66, 1.64)	0.79 (0.55, 1.14)	0.82 (0.57, 1.19)	0.28
HPFS	1	1.04 (0.97, 1.13)	1.10 (0.99, 1.23)	1.07 (0.96, 1.19)	1.01 (0.91, 1.11)	0.13
Pooled results[b]	1	1.13 (1.07, 1.19)	1.10 (1.02, 1.19)	1.16 (1.08, 1.26)	1.12 (1.04, 1.20)	<0.001
Kale, mustard, or chard greens						
Consumption level	<1 serving/month	1–2 servings/month	2–4 servings/month	≥1 serving/week		
NHS	1	0.94 (0.80, 1.11)	1.13 (0.91, 1.41)	1.12 (0.89, 1.42)	0.92 (0.75, 1.14)	0.23
NHSII	1	0.97 (0.68, 1.40)	1.83 (1.02, 3.27)	1.48 (0.92, 2.37)	1.10 (0.91, 1.34)	0.04
HPFS	1	1.02 (0.91, 1.15)	1.05 (0.87, 1.26)	0.97 (0.82, 1.14)	0.92 (0.80, 1.06)	0.89
Pooled results[b]	1	0.99 (0.91, 1.09)	1.11 (0.97, 1.28)	1.05 (0.92, 1.19)	0.97 (0.88, 1.07)	0.28

Notes: [a]Estimates are calculated in Cox proportional hazards models, adjusted for age (years), ethnicity (Caucasian, African American, Asian, and other ethnicity), family history of myocardial infarction (yes/no), hypertension (yes/no), hypercholesterolemia (yes/no), smoking status (never, former, current [1–14, 15–24, or ≥25 cigarettes/day], or missing), alcohol intake (0, 0.1–4.9, 5.0–14.9, and ≥15.0 g/day for women, 0, 0.1–4.9, 5.0–29.9, and ≥30.0 g/day for men, or missing), physical activity (<3.0, 3.0–8.9, 9.0–17.9, 18.0–26.9, ≥27.0 MET-hours/week, or missing), menopausal status and postmenopausal hormone use (premenopause, postmenopause [never, former, or current hormone use], or missing, for women), oral contraceptive use (yes, no, or missing, for NHSII), multivitamin use (yes/no), BMI (<23, 23–24.9, 25–29.9, 30–34.9, ≥35 kg/m^2, or missing), and total energy intake (kcal/day), and the modified Alternative Healthy Eating Index score (quintiles). [b]Results from each cohort were pooled using fixed-effects model.
Abbreviations: BMI, body mass index; HPFS, Health Professionals Follow-Up Study; MET, metabolic equivalents of task; NHS, Nurses' Health Study.

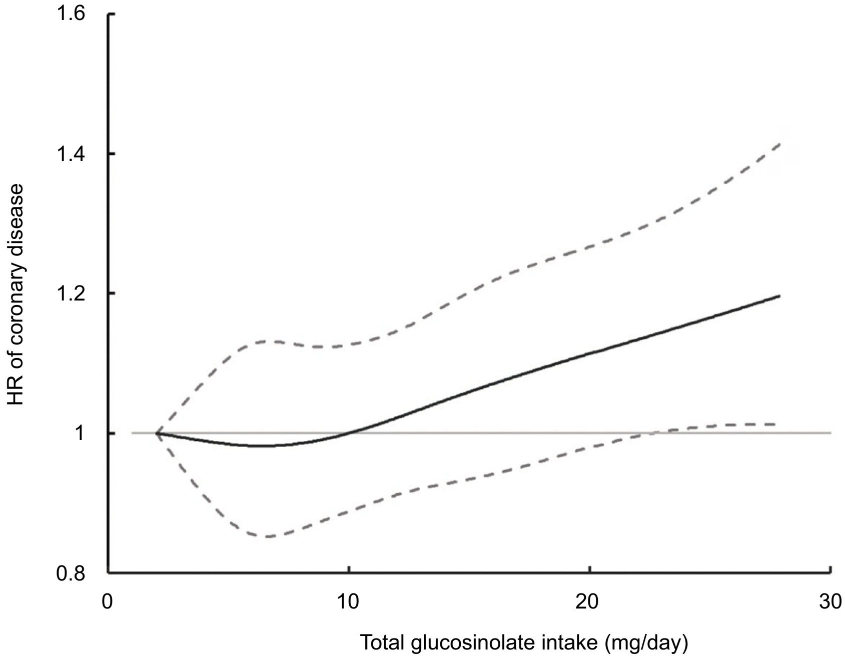

Figure S1 Restricted cubic spline analysis of the association between total glucosinolate intake (mg/day) and coronary heart disease.
Notes: Estimates are calculated in Cox proportional hazards models, adjusted for age (years), ethnicity (Caucasian, African American, Asian, and other ethnicity), family history of myocardial infarction (yes/no), hypertension (yes/no), hypercholesterolemia (yes/no), smoking status (never, former, current [1–14, 15–24, or ≥25 cigarettes/day], or missing), alcohol intake (0, 0.1–4.9, 5.0–14.9, and ≥15.0 g/day for women, 0, 0.1–4.9, 5.0–29.9, and ≥30.0 g/day for men, or missing), physical activity (<3.0, 3.0–8.9, 9.0–17.9, 18.0–26.9, ≥27.0 MET-hours/week, or missing), menopausal status and postmenopausal hormone use (premenopause, postmenopause [never, former, or current hormone use], or missing, for women), oral contraceptive use (yes, no, or missing, for Nurses' Health Study II), multivitamin use (yes/no), BMI (<23, 23–24.9, 25–29.9, 30–34.9, ≥35 kg/m^2, or missing) and total energy intake (kcal/day), and the modified Alternative Healthy Eating Index score (quintiles). Solid line is point estimate, and dashed lines are 95% CIs.
Abbreviations: BMI, body mass index; MET, metabolic equivalents of task.

Clinical Epidemiology Dovepress

Publish your work in this journal

Clinical Epidemiology is an international, peer-reviewed, open access, online journal focusing on disease and drug epidemiology, identification of risk factors and screening procedures to develop optimal preventative initiatives and programs. Specific topics include: diagnosis, prognosis, treatment, screening, prevention, risk factor modification, systematic reviews, risk and safety of medical interventions, epidemiology and biostatistical methods, and evaluation of guidelines, translational medicine, health policies and economic evaluations. The manuscript management system is completely online and includes a very quick and fair peer-review system, which is all easy to use.

Submit your manuscript here: https://www.dovepress.com/clinical-epidemiology-journal
