# Supplementary material for: A systematic comprehensive longitudinal evaluation of dietary factors associated with acute myocardial infarction and fatal coronary heart disease
Source: Nat Commun. 2020 Nov 27;11:6074. doi: 10.1038/s41467-020-19888-2 (PMC7699643; doi:10.1038/s41467-020-19888-2)
Supplement: Supplementary file 4 — Supplementary Software [file 41467_2020_19888_MOESM4_ESM.zip › EWAS-NHS-master/KG-SI/Rest/Li (2015).pdf]

Author Manuscript Author Manuscript Author Manuscript Author Manuscript

HHS Public Access
Author manuscript
J Am Coll Cardiol. Author manuscript; available in PMC 2016 October 06.

Published in final edited form as:
J Am Coll Cardiol. 2015 October 6; 66(14): 1538–1548. doi:10.1016/j.jacc.2015.07.055.

Saturated Fat as Compared With Unsaturated Fats and Sources of Carbohydrates in Relation to Risk of Coronary Heart Disease: A Prospective Cohort Study

Yanping Li, PHD[#,*], **Adela Hruby, PHD, MPH**[#,*], **Adam M. Bernstein, MD, SCD**[†], **Sylvia H. Ley, PHD**[*], **Dong D. Wang, MD**[*], **Stephanie E. Chiuve, SCD**[*,‡], **Laura Sampson, RD**[*], **Kathryn M. Rexrode, MD, MPH**[‡], **Eric B. Rimm, SCD**[*,§,‖], **Walter C. Willett, MD, DRPH**[*,§,‖], and **Frank B. Hu, MD, PHD**[*,§,‖]

[*]Department of Nutrition, Harvard T.H. Chan School of Public Health, Boston, Massachusetts

[†]Wellness Institute, Cleveland Clinic, Lyndhurst, Ohio

[‡]Division of Preventive Medicine, Department of Medicine, Brigham and Women's Hospital, and Harvard Medical School, Boston, Massachusetts

[§]Department of Epidemiology, Harvard T.H. Chan School of Public Health, Boston, Massachusetts

[‖]Channing Division of Network Medicine, Department of Medicine, Brigham and Women's Hospital, Harvard Medical School, Boston, Massachusetts

[#] These authors contributed equally to this work.

Abstract

Background—The associations between dietary saturated fat and risk of coronary heart disease (CHD) remain controversial, but few studies have compared saturated with unsaturated fats and sources of carbohydrates in relation to CHD risk.

Objective—This study sought to investigate associations of saturated fats as compared with unsaturated fats and different sources of carbohydrates in relation to CHD risk.

Methods—We followed 84,628 women (Nurses' Health Study, 1980 to 2010), and 42,908 men (Health Professionals Follow-up Study, 1986 to 2010) who were free of diabetes, cardiovascular disease, and cancer at baseline. Diet was assessed by semiquantitative food frequency questionnaire every 4 years.

Results—During 24 to 30 years of follow-up, we documented 7,667 incident cases of CHD. Higher intakes of polyunsaturated fatty acids (PUFAs) and carbohydrates from whole grains were

Reprint requests and correspondence: Frank B. Hu, MD, PhD, Departments of Nutrition and Epidemiology, Harvard T.H. Chan School of Public Health, 665 Huntington Avenue, Boston, Massachusetts 02115, Telephon: 617 432 0113 fax: 617 432 2435, nhbfh@channing.harvard.edu.

Publisher's Disclaimer: This is a PDF file of an unedited manuscript that has been accepted for publication. As a service to our customers we are providing this early version of the manuscript. The manuscript will undergo copyediting, typesetting, and review of the resulting proof before it is published in its final citable form. Please note that during the production process errors may be discovered which could affect the content, and all legal disclaimers that apply to the journal pertain.

Disclosures: The authors have reported that they have no relationships relevant to the contents of this paper to disclose.

Author Manuscript Author Manuscript Author Manuscript Author Manuscript

significantly associated with lower risk of CHD (hazard ratios [HR] (95% confidence intervals [CI]) comparing the highest to the lowest quintile for PUFA: 0.80 [0.73 to 0.88], p trend <0.0001; and for carbohydrates from whole grains: 0.90 [0.83 to 0.98], p trend = 0.003). In contrast, carbohydrates from refined starches/added sugars were positively associated with risk of CHD (1.10 [1.00 to 1.21], p trend = 0.04). Replacing 5% of energy intake from saturated fats with equivalent energy intake from either PUFAs, monounsaturated fats (MUFAs), or carbohydrates from whole grains was associated with 25%, 15%, and 9% lower risk of CHD, respectively (PUFAs: 0.75 [0.67 to 0.84]; p < 0.0001; MUFAs: 0.85 [0.74 to 0.97]; p = 0.02; carbohydrates from whole grains (0.91 [0.85 to 0.98]; p = 0.01). Replacing saturated fat with carbohydrates from refined starches/added sugars was not significantly associated with CHD risk (p > 0.10).

Conclusions—Our findings indicate that unsaturated fats, especially PUFAs, and/or high-quality carbohydrates should replace dietary saturated fats to reduce CHD risk.

Keywords

diet; follow-up studies; glycemic index

A recent systematic review and meta-analysis reported no significant association between consumption of saturated fatty acids (SFAs) and risk of coronary heart disease (CHD) (1), but the study failed to specify the replacement macronutrient for saturated fat. Another meta-analysis observed that substituting SFAs with overall carbohydrates was not associated with risk of CHD (2). Consistent with this analysis, a low-carbohydrate diet score (a higher score being indicative of higher protein and fat intake and lower intake of carbohydrates) was not associated with increased CHD risk in women (3). None of these studies, however, discriminated between different sources of dietary carbohydrates.

Carbohydrates, traditionally classified as simple versus complex, depending on the number of chained sugar moieties, are also frequently classified according to their effect on blood sugar levels, as quantified by the glycemic index (GI). Carbohydrate classification by GI has been shown to be more strongly associated with cardiometabolic disease than total carbohydrates. For example, while no association was observed between overall carbohydrate intake and risk of CHD (4,5), diets with low GI were associated with lower risk of CHD (4,5) and type 2 diabetes (6) when compared with high-GI diets, Therefore, it is not surprising that null associations between SFAs and coronary risk were observed in studies that did not distinguish between the quality of carbohydrates that were being substituted for saturated fats.

Only a few studies have considered the quality of the carbohydrates substituting for SFAs in CHD (7,8). One study (7) observed that substituting SFAs with carbohydrates was associated with a nonsignificantly lower risk of myocardial infarction (MI) among participants who consumed a low-GI diet, but with significantly increased risk among participants who consumed a high-GI diet. However, this association was not replicated in another study (8). To address uncertainties about the associations between dietary fats, carbohydrate quality, and CHD, we aimed to investigate the predicted effects of isocaloric substitutions of carbohydrates for fats, with an *a priori* hypothesis that the effects of different types of fats on risk of CHD depend on the carbohydrate quality of the

replacement. Two well-established cohorts of U.S. women and men: the Nurses' Health Study (NHS) (9) and the Health Professionals Follow-up Study (HPFS) (10) were the basis for this study. This analysis may have important public health implications for guiding people toward healthy dietary choices as they work to reduce their saturated fat intake.

Participants and Methods

Study population

In 1976, 121,701 female nurses in the United States aged 30 to 55 years enrolled in the NHS (9). In 1980, 98,047 of these women completed an extensive food frequency questionnaire (FFQ). In 1986, 51,529 U.S. men, aged 40 to 75 years, were enrolled in the HPFS and returned questionnaires about diet and medical history (10). Participants from both cohorts have provided information on diet and lifestyle factors, medical history, and newly diagnosed diseases through self-administered mailed questionnaires at baseline and every 2 to 4 years thereafter.

For the present analysis, we excluded women and men (n = 7,615) with implausible FFQ data (<800 or >4,200 kcal/day for men, <600 or >3,500 kcal/day for women, or >70 food items missing). We also excluded participants with previously diagnosed cancer (n = 5,676), cardiovascular disease (n = 5,609), or diabetes (n = 3,137) at baseline, or loss of follow-up after baseline (n = 3). The final analysis sample size was 84,628 women and 42,908 men.

The study protocol was approved by the institutional review boards of Brigham and Women's Hospital and the Harvard T.H. Chan School of Public Health. Return of the self-administered questionnaires was considered informed consent.

Ascertainment of CHD

We included nonfatal MI and CHD death as our primary endpoint of total CHD, which was identified primarily through a review of medical records, as previously described (11). Participants (or next of kin, for deceased participants) reporting a primary endpoint were asked for permission to have their medical records reviewed by physicians who were blinded to the participant's risk factor status. MI was confirmed if the criteria of the World Health Organization were met (12). MIs that required hospital admission and for which confirmatory information were obtained by phone interview or letter, but for which no medical records were available, were classified as probable. We included all confirmed and probable cases because results were similar in both previous (9) and present analyses when probable cases were excluded.

Deaths were identified by reports from next of kin, the U.S. postal system, or using certificates obtained from state vital statistics departments and the National Death Index. Follow-up for deaths was >98% complete (13). Cases of fatal CHD specifically were confirmed by either hospital records, autopsy, if CHD was listed as the cause of death on the death certificate, or if it was listed as an underlying cause of death and if evidence of previous CHD was available (9,13).

Author Manuscript Author Manuscript Author Manuscript Author Manuscript

Author Manuscript Author Manuscript Author Manuscript Author Manuscript

Dietary assessment

NHS participants completed a FFQ first in 1980, and again in 1984, 1986, 1990, 1994, 1998, 2002, and 2006. In HPFS, we assessed dietary information using the FFQ, administered every 4 years from 1986 to 2006. Participants were asked how often, on average, they had consumed specific foods during the past year. Serving sizes were specified for each food in the FFQ. The questionnaire had 9 possible responses, ranging from never or <1 time per month to ≥6 times per day. Participants were asked to specify the types of fat or oil used for frying and baking, at the table, and the type, if any, of margarine usually used.

Daily intake of fat and fatty acids was calculated by multiplying the frequency of consumption of each food item by its nutrient content and summing the nutrient contributions of all foods on the basis of U.S. Department of Agriculture food composition data (14), taking into account types of margarine and fats used in cooking and baking. The FFQ provides a reasonably good estimate of total and specific types of fat when compared with multiple dietary records; correlation coefficients between intakes from the 1986 questionnaire and 1986 dietary records were 0.57 for total fat, 0.68 for SFAs, 0.48 for PUFAs, and 0.58 for monounsaturated fats (MUFAs) (15). Correlations between *trans*-fat intake assessed by the FFQ and the composition of *trans* fat in adipose tissue was 0.51 (16).

In our analysis of carbohydrates, we focused on the major food sources of carbohydrates, including potatoes, added sugars from beverages and foods, and grains. These were then classified into 2 carbohydrate source categories: 1) carbohydrates from whole grains; and 2) carbohydrates from refined starches/sugars, mainly including foods with relatively high GI values, such as potatoes, refined grains, and added sugar from beverages and foods (17). The food list is detailed in **Online Table 1**. We did not include carbohydrates from fruits, vegetables, or legumes in our carbohydrate categories because these foods have benefits separate from their carbohydrate quality, and have been previously been shown to be inversely associated with CHD in our cohorts (18).

Because dietary intake may affect CHD over an extended period of time, to best represent long-term intake, we calculated cumulative average intakes of nutrients using repeated FFQ data for our primary analysis (19). We applied time-varying Cox analysis, in which the average of dietary intake at or before the beginning of a 4-year interval was used as a predictor for incident CHD in that 4-year interval. For each follow-up period, baseline CHD cases were excluded. For example, for the NHS, 1980 (baseline) intake was used to assess the risk of CHD in the 1980 to 1984 follow-up period. The average of 1980 and 1984 intakes was used to assess the risk of CHD in the 1984 to 1986 follow-up period, and the average of 1980, 1984, and 1986 intakes was used to assess the risk of CHD in the 1986 to 1990 follow-up period, and so on. Thus, we maintained a strictly prospective analysis in assessing the association between a cumulative dietary exposure and CHD.

Statistical analysis

To describe and visualize changes in dietary habits over time, we assessed 2- to 4-year changes in energy intake from different fatty acids and carbohydrate sources according to deciles of 2- to 4-year changes in SFAs as a percentage of energy intake, using generalized

Author Manuscript Author Manuscript Author Manuscript Author Manuscript

linear models with repeated-measures analysis with adjustment for age. Only 1984 to 1986 changes in NHS were 2-year changes; all other changes were 4-year changes. For the change analyses, individuals without consecutively completed FFQs were excluded. Changes in energy intake from different fatty acids and carbohydrate sources were evaluated as continuous variables and were censored at the 0.5^{th} and 99.5^{th} percentiles to minimize the influence of outliers.

For prospective risk analyses of dietary factors on CHD, individuals contributed person-time from the return of the baseline questionnaire (1980 for NHS; 1986 for HPFS) until the date of diagnosis of CHD, death, loss to follow-up, or the end of the follow-up period (June 30, 2010 for NHS; January 30, 2010 for HPFS), whichever came first.

Participants were divided into quintile categories of intake according to their nutrient intakes. We used multivariable Cox proportional hazards models to estimate hazard ratios (HRs) and 95% confidence intervals (CIs) comparing people in a given quintile category of nutrient intake with those in the lowest quintile. To quantify a linear trend, we conducted a Wald test for linear trend by assigning the median intake within each quintile and modeling this variable continuously.

In the multivariate-adjusted model, we adjusted for known risk factors for CHD, including: body mass index (BMI: <20.9, 21 to 24.9, 25 to 29.9, 30 to 31.9, and ≥32 kg/m^2); family history of diabetes (yes or no) and MI (yes or no); menopausal status (pre- or post-menopausal) and hormone therapy use (never, past, or current) in women; regular use of aspirin (yes or no) and multiple vitamins (yes or no); smoking status (never, former, current: 1 to 14, 15 to 24, or ≥25 cigarettes/day); physical activity (quintiles); presence of hypertension (yes or no) and hypercholesterolemia (yes or no) at baseline; and alcohol intake (0, 0.1 to 4.9, 5.0 to 9.9, 10.0 to 14.9, 15.0 to 29.9 or ≥30 g/day for men; for women the highest 2 categories were merged into ≥15 g/day); energy intake (kcal/day); and percentages of energy from protein and dietary cholesterol (all in quintiles). We also simultaneously included the percentages of energy from all types of fats to estimate the main association of each fatty acid. We separately included both categories of carbohydrate sources to estimate the main associations of each of the 2 carbohydrate sources.

When estimating the effect of substituting 1 type of fat or 1 source of carbohydrate for another, we included energy contributions from the 2 nutrient types as continuous variables in the same multivariate-adjusted model. The differences in their coefficients and covariance were used to estimate the HR and 95% CI of the substitution.

Incidence of diabetes, angina, hypertension, coronary artery surgery or angioplasty, and hypercholesterolemia during follow-up periods were not included in multivariable models, because these may be considered intermediate outcomes on the causal pathway between diet and CHD. However, intermediate diagnoses were included in sensitivity analyses. In additional sensitivity analyses, we stopped updating diet at the beginning of the interval in which the participant developed the potential intermediate outcome(s).

Because of differences between the 2 cohorts in sex, follow-up time, and the questionnaires, all analyses were performed separately in each cohort to achieve better control of

Author Manuscript
Author Manuscript
Author Manuscript
Author Manuscript

confounding. To obtain overall estimates for both sexes and to increase statistical power, the HRs from the age- and multivariable-adjusted models from the 2 cohorts were combined with the use of a fixed-effect inverse variance-weighted meta-analysis because no significant heterogeneity between the cohorts was observed. Data were analyzed using a commercially available software program (SAS, version 9.2; SAS Institute, Inc., Cary, North Carolina), and statistical significance was set at a 2-tailed p value <0.05.

Results

Participant characteristics

We documented 7,667 cases of CHD (4,931 nonfatal MIs and 2,736 CHD deaths) over 30 years of follow-up in the NHS, and 24 years of follow-up in the HPFS. At baseline (**Table 1**), men and women with high intake of SFAs as a percentage of energy were slightly younger, had a higher BMI, a lower prevalence of physical activity and multivitamin use, and consumed more cholesterol. Participants with higher energy intake from SFAs also tended to have higher energy intake from MUFAs and *trans* fats, and lower energy intake from carbohydrates (**Table 1**). Carbohydrates from whole grains contributed 0.7% to 5.2% of energy, whereas carbohydrates from refined starches/added sugars contributed 17.7% to 23.9% of energy. The percentage of energy from carbohydrates from whole grains was strongly negatively associated with intake of saturated fat, but the energy contribution from carbohydrates from refined starches/add sugars was minimally associated with energy from saturated fat (**Table 1**).

Changes in energy intake from different fats and carbohydrate sources

Mean 2- to 4-year changes in energy intake from SFAs were positively associated with changes in energy intake from MUFAs and negatively associated with changes in energy intake from carbohydrates from refined starches/sugars. Associations were much weaker between changes in energy intake from SFAs and changes in energy intake from PUFAs or carbohydrates from whole grains (**Online Figure 1**). In other words, participants generally replaced calories derived from SFAs with calories from low-quality carbohydrates, rather than with calories from PUFAs or high-quality carbohydrates.

Main associations of different types of fats and sources of carbohydrates on risk of CHD

Intakes of SFAs, MUFAs, PUFAs, and *trans* fats were each significantly associated with risk of CHD in age-adjusted analyses (**Table 2**). When we incorporated all types of fat into the same model, so that the HRs represented replacing energy from total carbohydrates with the same percentage of energy from each type of fat, higher PUFA intake was associated with lower risk of CHD (for highest vs. lowest quintiles, HR [95% CI]: 0.80 [0.73 to 0.88], p trend <0.0001), and *trans* fat intake was significantly associated with increased risk of CHD (1.20 [1.09 to 1.32], p trend = 0.002). Neither SFA nor MUFA were associated with CHD in multivariable models when they were modeled as replacement for total carbohydrate.

In analyses of different sources of carbohydrates, risk of CHD was significantly lower with higher consumption of energy from carbohydrates from whole grains (for highest vs. lowest quintiles, HR [95% CI]: 0.90 [0.83 to 0.98], p trend = 0.003), and significantly higher with

increasing consumption of carbohydrates from refined starches/added sugars (1.10 [1.00 to 1.21], p trend = 0.04) (**Table 3**).

Substitution analyses

The associations of various isocaloric dietary substitutions on the risk of CHD are presented in the **Central Illustration**. Isocaloric dietary substitutions of SFAs by *trans* fats or carbohydrates from refined starches/sugars were not associated with risk of CHD ($p > 0.1$ for both). Replacing 5% of energy from SFAs with 5% of energy from PUFAs was associated with 25% lower risk of CHD (HR [95% CI]: 0.75 [0.67 to 0.84], $p < 0.0001$). Similarly, replacing 5% of energy from SFAs with 5% of energy from MUFAs or from carbohydrates from whole grains was associated with significantly lower risk of CHD (MUFAs: 0.85 [0.74 to 0.97], p = 0.02; carbohydrates from whole grains: 0.91 [0.85 to 0.98], p = 0.01). The results were consistent in NHS and HPFS (**Online Table 2**). Replacement of 5% of energy from saturated fats with 5% of energy from unsaturated fats (MUFA + PUFA) was associated with 17% lower risk of CHD (0.83 [0.75 to 0.91], p = 0.0001).

Isocaloric (5%) dietary substitutions of carbohydrates from refined starches/added sugars with PUFAs or with carbohydrates from whole grains were also significantly associated with lower risk of CHD (PUFAs: 0.78 [0.71 to 0.85], $p < 0.0001$; carbohydrates from whole grains: 0.89 [0.84 to 0.94], $p < 0.0001$) (**Central Illustration**).

Sensitivity analyses

In sensitivity analyses with further adjustment for potential intermediate outcomes (hypercholesterolemia, diabetes, angina, hypertension, coronary artery surgery, or angioplasty), isocaloric (5%) dietary substitutions of SFAs with PUFAs, MUFAs, or carbohydrates from whole grains resulted in 28%, 20%, and 11% lower risk of CHD, respectively (all $p < 0.01$). In sensitivity analyses that stopped updating dietary intake after diagnosis of potential intermediate outcomes, the isocaloric (5%) dietary substitutions of SFAs with PUFAs, MUFAs, or carbohydrates from whole grains resulted in 29%, 16%, and 23% lower risk of CHD, respectively (all $p < 0.01$).

Discussion

In 2 large, independent prospective cohorts of U.S. men and women, we found that replacing intake of energy from SFAs with energy from MUFAs, PUFAs, or carbohydrates from whole grains was associated with lower risk of CHD. In addition, isocaloric substitution of carbohydrates from refined starches/added sugars with carbohydrates from whole grains or with energy from PUFAs was also associated with lower risk of CHD. Our findings provide epidemiological evidence for the current dietary guidelines, which recommend both “replacing saturated fatty acids with monounsaturated and polyunsaturated fatty acids” and “replacing refined grains with whole grains” (20).

A large body of evidence indicates that higher intake of most dietary SFAs increases blood levels of low-density lipoprotein (LDL) cholesterol and the LDL to high-density lipoprotein (HDL) ratio (21), both of which are associated with higher risk of CHD (22). A reduction in

Author Manuscript Author Manuscript Author Manuscript Author Manuscript

Author Manuscript Author Manuscript Author Manuscript Author Manuscript

saturated fat intake has therefore been at the heart of most dietary recommendations, with the objective of reducing the risk of cardiovascular diseases. Recently, this hypothesis was challenged by studies that failed to find an association between saturated fats and CHD (1,2). However, in these studies, the replacement nutrient was not specified, although by default most of the other calories in almost all diets would have been carbohydrates, primarily from refined grains and added sugars. Notably, in our own cohorts, we also observed the less healthful replacement to be the norm.

In clinical trials, replacement of saturated fats with refined carbohydrates has been associated with lower high-density lipoprotein (HDL) cholesterol and higher triglycerides (21). The fact that predominantly low-quality carbohydrates, such as refined grains and added sugars, have served as the primary isocaloric replacements of saturated fats in prior analyses, likely explains the previous null associations between SFA and CHD. The importance of the source of carbohydrate was also suggested by a previous analysis from Denmark that was stratified according to the overall dietary GI (7). In that study, replacing SFAs with carbohydrates was associated with higher risk of MI when the dietary GI was high; but when the dietary GI was low, replacing SFAs with carbohydrates was associated with nonsignificantly lower risk of MI. Our study provides further evidence that the macronutrient substituted for saturated fat is critically important: replacing SFAs with high-quality carbohydrates such as whole grains may decrease risk of CHD, whereas replacing SFAs with low-quality carbohydrates, such as white bread, white rice, or potatoes, is not beneficial for CHD prevention.

There is strong scientific evidence for CHD reduction when SFAs are replaced with PUFAs. A pooled analysis of 11 prospective cohort studies indicated that replacing 5% of energy from SFAs with energy from PUFAs was associated with 13% lower risk of CHD (2). This estimate was confirmed by a meta-analysis of 8 clinical trials that used PUFA consumption as a replacement for SFA consumption, finding that each 1% of energy from PUFAs replacing SFAs reduced the occurrence of CHD events by 2% (23). Substitution of SFAs by PUFAs also reduces the total-to-HDL cholesterol ratio, as compared with replacing SFAs with carbohydrates (23).

Our findings suggest that PUFAs, such as those from vegetable oils, nuts, and seeds, should have an expanded role as replacement for SFAs. However, our data and data from national surveys suggest that, when decreasing SFA intake, most people appear to increase the intake of low-quality carbohydrates, such as refined starches and/or added sugars, rather than increase intake of unsaturated fats. The 2010 American Dietary Guidelines recommend "consuming at least half of all grains as whole grains" (20). Although whole-grain consumption has increased in the United States in recent decades (24), overall intake is still quite low (around 1 ounce-equivalent of whole grains per day); fewer than 5% people in the United States consume the minimum recommended amount of whole grains, which, for most, is about 3 ounce-equivalents per day (20). These consumption patterns suggest there is substantial room for improvement in the American diet when it comes to intakes of PUFA and whole grains as meaningful replacements for SFAs.

Author Manuscript Author Manuscript Author Manuscript Author Manuscript

Because monounsaturated fats tend to share the same food sources as saturated fats (e.g., meats, dairy, and partially hydrogenated fats), the estimated association between monounsaturated fats and CHD risk in most epidemiologic studies in the United States is likely to be confounded by these food sources. In our study, replacing saturated fats with monounsaturated fats was associated with a lower CHD risk, suggesting that there are healthful benefits to replacing food sources of saturated fats with plant sources of monounsaturated fats, such as vegetables oils (e.g., olive oil and canola oil), nuts, and seeds. The apparent benefit that we observed for monounsaturated fats is consistent with their effects on LDL and HDL, which are similar to those for polyunsaturated fats (21,23). In the PREDIMED trial, the intervention groups consuming a Mediterranean diet supplemented with extra-virgin olive oil or mixed nuts experienced 30% lower risk of cardiovascular disease compared with the control group (25).

Several limitations warrant consideration. Given its observational nature, our study cannot prove causality. Similar to other observational studies, it is difficult to rule out residual confounding, despite careful control for potential confounders in the analyses. In addition, measurement errors are inevitable in estimates of food and nutrient intakes. However, our adjustment for energy intake and use of prospectively collected, cumulatively averaged intake reduced the magnitude of measurement errors (26). In addition, we could not make finer distinctions between subtypes of CHD (e.g., ST-segment elevation MI), which would require ECG data that was not collected in the cohorts. Such subtypes may be relevant to the present hypothesis and future research in this area is warranted. Although our cohorts benefitted from their homogeneity in terms of internal validity, the associations we observed in this population may not be generalizable to more diverse populations. Strengths of the present study include its large sample size, high rates of long-term follow-up, and detailed, repeated assessments of diet and lifestyle. The consistent findings across 2 cohorts demonstrate the robustness of the results. All participants were health professionals, minimizing potential confounding by educational attainment or differential access to health care. In addition, the FFQs used in these studies were validated against multiple weighed diet records and biochemical markers.

In sum, our data suggest that replacing SFAs with PUFAs, MUFAs, or carbohydrates from whole grains is associated with lower CHD risk. We further observed that replacing carbohydrates from refined starches/sugars with PUFAs or carbohydrates from whole grains, was associated with lower risk of CHD. Our observations, together with evidence from previous studies, indicate that evidence-based population-level and individual-level recommendations to reduce SFA consumption should specify replacing SFAs with unsaturated fats, especially PUFAs and/or high-quality carbohydrates.

Supplementary Material

Refer to Web version on PubMed Central for supplementary material.

Acknowledgements

The cohorts were supported by grants of UM1 CA186107, R01 HL034594, R01 HL35464, R01 HL60712, and UM1 CA167552 from the National Institutes of Health

Author Manuscript Author Manuscript Author Manuscript Author Manuscript

ABBREVIATIONS

**BMI**	body mass index
**CHD**	coronary heart disease
**FFQ**	food frequency questionnaire
**MI**	myocardial infarction
**MUFA**	monounsaturated fatty acids
**PUFA**	polyunsaturated fatty acids
**SFA**	saturated fatty acids
**%E**	percent of energy intake

References

1. Chowdhury R, Warnakula S, Kunutsor S, et al. Association of dietary, circulating, and supplement fatty acids with coronary risk: a systematic review and meta-analysis. Ann Intern Med. 2014; 160:398–406. [PubMed: 24723079]
2. Jakobsen MU, O'Reilly EJ, Heitmann BL, et al. Major types of dietary fat and risk of coronary heart disease: a pooled analysis of 11 cohort studies. Am J Clin Nutr. 2009; 89:1425–32. [PubMed: 19211817]
3. Halton TL, Willett WC, Liu S, et al. Low-carbohydrate-diet score and the risk of coronary heart disease in women. N Engl J Med. 2006; 355:1991–2002. [PubMed: 17093250]
4. Barclay AW, Petocz P, McMillan-Price J, et al. Glycemic index, glycemic load, and chronic disease risk--a meta-analysis of observational studies. Am J Clin Nutr. 2008; 87:627–37. [PubMed: 18326601]
5. Liu S, Willett WC, Stampfer MJ, et al. A prospective study of dietary glycemic load, carbohydrate intake, and risk of coronary heart disease in US women. Am J Clin Nutr. 2000; 71:1455–61. [PubMed: 10837285]
6. Bhupathiraju SN, Tobias DK, Malik VS, et al. Glycemic index, glycemic load, and risk of type 2 diabetes: results from 3 large US cohorts and an updated meta-analysis. Am J Clin Nutr. 2014; 100:218–32. [PubMed: 24787496]
7. Jakobsen MU, Dethlefsen C, Joensen AM, et al. Intake of carbohydrates compared with intake of saturated fatty acids and risk of myocardial infarction: importance of the glycemic index. Am J Clin Nutr. 2010; 91:1764–8. [PubMed: 20375186]
8. Similä ME, Kontto JP, Männistö S, et al. Glycaemic index, carbohydrate substitution for fat and risk of CHD in men. Br J Nutr. 2013; 110:1704–11. [PubMed: 23534456]
9. Willett WC, Green A, Stampfer MJ, et al. Relative and absolute excess risks of coronary heart disease among women who smoke cigarettes. N Engl J Med. 1987; 317:1303–9. [PubMed: 3683458]
10. Hu FB, Willett WC. Diet and coronary heart disease: findings from the Nurses' Health Study and Health Professionals' Follow-up Study. J Nutr Health Aging. 2001; 5:132–8. [PubMed: 11458281]
11. Chiuve SE, Sampson L, Willett WC. The association between a nutritional quality index and risk of chronic disease. Am J Prev Med. 2011; 40:505–13. [PubMed: 21496749]
12. Rose, GA.; Blackburn, H.; Gillum, RF., et al. Cardiovascular Survey Methods. Second. World Health Organization; Geneva, Switzerland: 1982. WHO monograph series, No. 56
13. Stampfer MJ, Willett WC, Speizer FE, et al. Test of the National Death Index. Am J Epidemiol. 1984; 119:837–9. [PubMed: 6720679]
14. U.S. Department of Agriculture. Composition of foods-raw, processed, and prepared, 1963–1992. Department of Agriculture, Government Printing Office; Washington, DC: 1993.

Author Manuscript Author Manuscript Author Manuscript Author Manuscript

15. Willett, W. Nutritional Epidemiology. Oxford University Press; New York, NY: 1998.
16. London SJ, Sacks FM, Caesar J, et al. Fatty acid composition of subcutaneous adipose tissue and diet in postmenopausal US women. Am J Clin Nutr. 1991; 54:340–5. [PubMed: 1858698]
17. Jenkins DJ, Wolever TM, Taylor RH, et al. Glycemic index of foods: a physiological basis for carbohydrate exchange. Am J Clin Nutr. 1981; 34:362–6. [PubMed: 6259925]
18. Bhupathiraju SN, Wedick NM, Pan A, et al. Quantity and variety in fruit and vegetable intake and risk of coronary heart disease. Am J Clin Nutr. 2013; 98:1514–23. [PubMed: 24088718]
19. Hu FB, Stampfer MJ, Rimm E, et al. Dietary fat and coronary heart disease: a comparison of approaches for adjusting for total energy intake and modeling repeated dietary measurements. Am J Epidemiol. 1999; 149:531–40. [PubMed: 10084242]
20. U.S. Department of Agriculture, U.S. Department of Health and Human Services. Dietary Guidelines for Americans 2010. 7th. U.S. Government Printing Office; Washington, DC: Dec. 2010
21. Mensink RP, Zock PL, Kester AD, et al. Effects of dietary fatty acids and carbohydrates on the ratio of serum total to HDL cholesterol and on serum lipids and apolipoproteins: a meta-analysis of 60 controlled trials. Am J Clin Nutr. 2003; 77:1146–55. [PubMed: 12716665]
22. Prospective Studies Collaboration. Lewington S, Whitlock G, et al. Blood cholesterol and vascular mortality by age, sex, and blood pressure: a meta-analysis of individual data from 61 prospective studies with 55,000 vascular deaths. Lancet. 2007; 370:1829–39. [PubMed: 18061058]
23. Mozaffarian D, Micha R, Wallace S. Effects on coronary heart disease of increasing polyunsaturated fat in place of saturated fat: a systematic review and meta-analysis of randomized controlled trials. PLoS Med. 2010; 7:e1000252. [PubMed: 20351774]
24. Wang DD, Leung CW, Li Y, et al. Trends in dietary quality among adults in the United States, 1999 through 2010. JAMA Intern Med. 2014; 174:1587–95. [PubMed: 25179639]
25. Estruch R, Ros E, Salas-Salvadó J, et al. PREDIMED Study Investigators. Primary prevention of cardiovascular disease with a Mediterranean diet. N Engl J Med. 2013; 368:1279–90. [PubMed: 23432189]
26. Willett W, Stampfer MJ. Total energy intake: implications for epidemiologic analyses. Am J Epi. 1986; 124:17–27.

Author Manuscript

Author Manuscript

Author Manuscript

Author Manuscript

PERSPECTIVES

Competency in Patient Care

Guiding individuals toward healthy dietary choices as they work to reduce their saturated fat intake should specify their replacement with polyunsaturated or monounsaturated fats or high-quality carbohydrates.

Translational Outlook

Further individual-level and population-level research is needed to optimize dietary scoring schemes and optimum substitution ratios to replace saturated fats with unsaturated fats, carbohydrates and other food components.

Author Manuscript
Author Manuscript
Author Manuscript
Author Manuscript

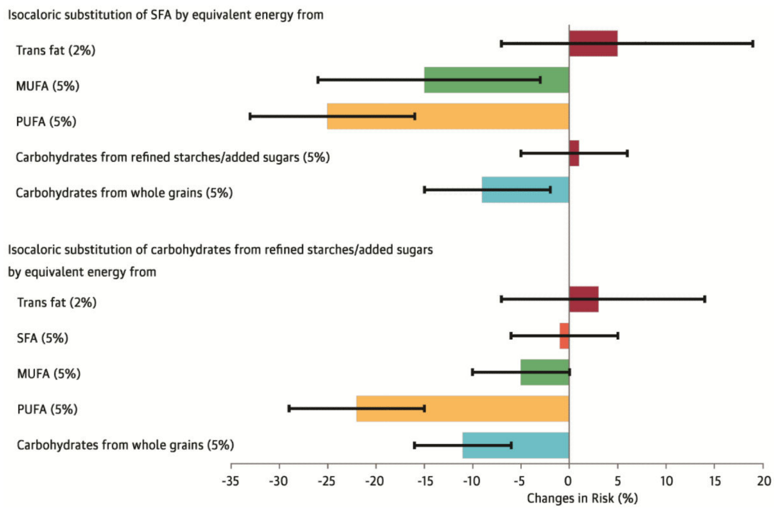

FIGURE. Central Illustration. Fat, Carbohydrates, and Heart Disease: Estimated Percent Changes in the Risk of Coronary Heart Disease Associated with Isocaloric Substitutions of 1 Dietary Component for Another

Changes in risk are derived from hazard ratios and represented as solid bars; I bars represent 95% confidence intervals. The multivariate model was adjusted for: total energy intake; the energy contribution from protein; cholesterol intake; alcohol intake; smoking status; body mass index; physical activity; use of vitamins and aspirin; family history of myocardial infarction and diabetes; and presence of baseline hypercholesterolemia and hypertension.

Author Manuscript Author Manuscript Author Manuscript Author Manuscript

Table 1

Baseline Characteristics of Men and Women Across Quintile Categories of Saturated Fatty Acid Intake, as a Percentage of Total Energy Intake

	NHS, 1980					HPFS, 1986				
**SFA intake (%E)**	**Q1 (9.6)**	**Q2 (11.3)**	**Q3 (12.6)**	**Q4 (14.1)**	**Q5 (16.9)**	**Q1 (7.4)**	**Q2 (9.2)**	**Q3 (10.4)**	**Q4 (11.6)**	**Q5 (13.6)**
Age, yrs	47.7	46.6	46.3	46.0	45.8	54.2	53.6	53.0	52.6	52.5
BMI, kg/m^2	23.8	24.1	24.2	24.3	24.4	24.6	25.2	25.4	25.7	26.0
Exercise, h/week	4.3	4.0	4.1	4.0	3.8	3.9	3.2	2.8	2.6	2.2
Current smoking, %	27	28	26	27	30	6	7	9	10	14
Premenopausal, %	55	56	56	56	57	-		-		-
Multivitamin use, %	38	36	36	34	32	69	65	63	61	58
Regular aspirin use, %	43	46	47	48	47	26	27	27	27	26
Alcohol, g/day	9.4	8.2	7.6	6.9	5.4	14.6	13.6	12.3	10.7	8.7
Cholesterol, mg/dl	235	269	286	310	363	216	263	290	318	367
Hypercholesterolemia, %	7	6	5	5	4	17	12	10	9	7
Hypertension, %	17	17	15	15	15	21	20	20	19	18
Family history of diabetes, %	27	28	27	27	28	20	20	19	21	21
Family history of MI, %	19	20	19	19	19	36	33	31	32	30
Total energy, kcal/day	1393	1453	1492	1552	1634	1886	1928	1997	2032	2069
Nutrient intake, %E										
Protein	18.4	18.5	18.7	18.8	19.4	18.2	18.4	18.4	18.5	18.8
Total fatty acids	25.2	30.5	33.4	36.6	43.9	23.4	28.6	31.3	33.8	38.2
MUFA	9.8	12.1	13.4	15.0	18.3	8.7	10.9	12.0	13.0	14.6
PUFA	4.5	5.0	5.2	5.4	5.4	5.4	5.9	6.0	6.1	6.0
Trans fat	1.6	2.0	2.1	2.2	2.4	0.8	1.1	1.3	1.4	1.5
Total carbohydrates	51.3	46.8	44.2	41.2	34.2	55.1	49.8	47.5	45.3	41.3
Carbohydrates from whole grains	1.20	1.14	1.05	0.99	0.72	5.2	3.6	3.0	2.5	2.0
Carbohydrates from refined starches/added sugars	23.9	22.8	21.8	20.8	17.7	23.3	23.6	23.8	23.8	22.6

BMI = body mass index; HPFS = Health Professionals Follow-up Study; MI = myocardial infarction; MUFA = monounsaturated fatty acids; NHS = Nurses' Health Study; PUFA = polyunsaturated fatty acids; SFA = saturated fatty acids; %E = percent of energy intake.

Author Manuscript Author Manuscript Author Manuscript Author Manuscript

Table 2

Hazard Ratios of Coronary Heart Disease by Intake Of Fatty Acids as Percentages of Total Energy Intake

		Quintile Categories of Fat Intake as a percentage of Total Energy Intake					p Trend
		1	2	3	4	5	
**Total fatty acids**							
NHS	Median (%E)	27.6	31.7	34.5	37.6	42.7	
	Cases	808	735	676	698	575	
	Age-adjusted	1 *(ref.)*	1.02 (0.92-1.13)	1.05 (0.94-1.16)	1.21 (1.10-1.35)	1.31 (1.17-1.46)	<0.0001
	Multivariate*	1 *(ref.)*	0.94 (0.85-1.04)	0.89 (0.80-0.99)	0.94 (0.84-1.05)	0.87 (0.76-0.99)	0.06
HPFS	Median (%E)	24.1	28.5	31.4	34.2	38.3	
	Cases	833	800	819	878	845	
	Age-adjusted	1 *(ref.)*	1.00 (0.91-1.10)	1.06 (0.96-1.17)	1.19 (1.08-1.31)	1.20 (1.09-1.32)	<0.0001
	Multivariate*	1 *(ref.)*	0.94 (0.85-1.04)	0.94 (0.84-1.04)	0.99 (0.89-1.10)	0.89 (0.79-1.00)	0.16
Pooled	Multivariate*	1 *(ref.)*	0.94 (0.87-1.01)	0.91 (0.85-0.99)	0.97 (0.89-1.04)	0.88 (0.81-0.96)	0.02
**SFA**							
NHS	Median (%E)	9.6	11.3	12.6	14.1	16.9	
	Cases	791	754	711	649	587	
	Age-adjusted	1 *(ref.)*	1.10 (1.00-1.22)	1.18 (1.07-1.31)	1.28 (1.15-1.42)	1.54 (1.38-1.73)	<0.0001
	Multivariate*	1 *(ref.)*	0.96 (0.86-1.08)	0.95 (0.83-1.09)	0.94 (0.81-1.09)	1.01 (0.84-1.22)	0.73
HPFS	Median (%E)	7.4	9.2	10.4	11.6	13.6	
	Cases	808	815	835	870	847	
	Age-adjusted	1 *(ref.)*	1.07 (0.97-1.18)	1.16 (1.05-1.28)	1.26 (1.15-1.39)	1.32 (1.19-1.45)	<0.0001
	Multivariate*	1 *(ref.)*	0.95 (0.86-1.08)	0.96 (0.85-1.10)	0.95 (0.82-1.10)	0.87 (0.74-1.02)	0.16
Pooled	Multivariate*	1 *(ref.)*	0.96 (0.89-1.04)	0.96 (0.87-1.05)	0.95 (0.85-1.05)	0.93 (0.82-1.05)	0.46
***Trans* fat**							
NHS	Median (%E)	1.1	1.5	1.7	2.0	2.6	
	Cases	726	771	748	683	564	
	Age-adjusted	1 *(ref.)*	1.17 (1.06-1.30)	1.26 (1.14-1.40)	1.31 (1.17-1.45)	1.38 (1.23-1.56)	<0.0001
	Multivariate*	1 *(ref.)*	1.08 (0.97-1.21)	1.13 (1.00-1.28)	1.15 (1.00-1.31)	1.20 (1.02-1.40)	0.04
HPFS	Median (%E)	0.7	1.0	1.3	1.5	1.9	
	Cases	705	823	877	874	896	
	Age-adjusted	1 *(ref.)*	1.20 (1.08-1.32)	1.26 (1.14-1.39)	1.31 (1.18-1.44)	1.39 (1.25-1.53)	<0.0001
	Multivariate*	1 *(ref.)*	1.18 (1.06-1.32)	1.21 (1.07-1.36)	1.22 (1.07-1.38)	1.21 (1.06-1.38)	0.03
Pooled	Multivariate*	1 *(ref.)*	1.13 (1.05-1.22)	1.17 (1.08-1.27)	1.18 (1.08-1.30)	1.20 (1.09-1.32)	0.002
**PUFA**							
NHS	Median (%E)	3.9	4.9	5.6	6.3	7.4	
	Cases	763	749	650	726	604	
	Age-adjusted	1 *(ref.)*	0.91 (0.82-1.01)	0.80 (0.72-0.89)	0.92 (0.83-1.03)	0.83 (0.75-0.93)	0.003
	Multivariate*	1 *(ref.)*	0.91 (0.82-1.01)	0.78 (0.70-0.88)	0.88 (0.78-0.99)	0.76 (0.67-0.87)	0.0003
HPFS	Median (%E)	4.4	5.2	5.8	6.4	7.5	

Author Manuscript Author Manuscript Author Manuscript Author Manuscript

		Quintile Categories of Fat Intake as a percentage of Total Energy Intake					p Trend
		1	2	3	4	5	
	Cases	886	893	799	831	766	
	Age-adjusted	1 *(ref.)*	1.02 (0.93-1.12)	0.93 (0.84-1.02)	0.98 (0.89-1.08)	0.92 (0.84-1.02)	0.07
	Multivariate*	1 *(ref.)*	1.00 (0.91-1.10)	0.90 (0.81-1.00)	0.92 (0.83-1.03)	0.83 (0.74-0.94)	0.001
Pooled	Multivariate*	1 *(ref.)*	0.96 (0.89-1.03)	0.85 (0.78-0.92)	0.90 (0.84-0.98)	0.80 (0.73-0.88)	<0.0001
**MUFA**							
NHS	Median (%E)	10.4	12.1	13.4	14.8	17.3	
	Cases	788	747	744	669	544	
	Age-adjusted	1 *(ref.)*	1.06 (0.96-1.18)	1.17 (1.06-1.29)	1.18 (1.06-1.31)	1.27 (1.13-1.42)	<0.0001
	Multivariate*	1 *(ref.)*	1.01 (0.90-1.14)	1.05 (0.91-1.21)	0.97 (0.83-1.15)	0.90 (0.74-1.09)	0.22
HPFS	Median (%E)	9.0	10.9	12.1	13.3	15.1	
	Cases	810	811	832	833	889	
	Age-adjusted	1 *(ref.)*	1.02 (0.93-1.13)	1.09 (0.99-1.20)	1.12 (1.02-1.23)	1.24 (1.13-1.36)	<0.0001
	Multivariate*	1 *(ref.)*	0.97 (0.86-1.09)	1.01 (0.88-1.15)	1.00 (0.86-1.17)	1.08 (0.91-1.29)	0.33
Pooled	Multivariate*	1 *(ref.)*	0.99 (0.91-1.08)	1.03 (0.93-1.13)	0.99 (0.89-1.11)	1.00 (0.87-1.13)	0.86

Ref. = reference group. Abbreviations as in Table 1.

* The multivariate model was adjusted for total energy intake, the energy contribution from protein, cholesterol intake, alcohol intake, smoking status, body mass index, physical activity, use of vitamins and aspirin, family history of MI and diabetes, and presence of baseline hypercholesterolemia and hypertension. The model was also adjusted for dietary intake of fruits and vegetables. The specific fat models were adjusted for the percentage of energy from all fat types simultaneously. Multivariate models thus represent the effect of substituting a percentage of energy from total fat or the specific fat type for an equivalent percentage of energy from total carbohydrates.

Author Manuscript Author Manuscript Author Manuscript Author Manuscript

Table 3

Hazard ratios of Coronary Heart Disease by Intake of Carbohydrates of Varying Quality as Percentages of Total Energy Intake

		Quintile Categories of Carbohydrate Intake as a Percentage of Total Energy Intake					p Trend
		1	2	3	4	5	
**Total carbohydrates**							
NHS	Median (%E)	34.7	41.7	45.8	49.4	54.5	
	Cases	560	647	705	740	840	
	Age-adjusted	1 *(ref.)*	0.94 (0.84-1.05)	0.90 (0.81-1.01)	0.85 (0.76-0.96)	0.84 (0.75-0.94)	0.0009
	Multivariate*	1 *(ref.)*	1.02 (0.90-1.14)	1.03 (0.91-1.17)	1.03 (0.90-1.17)	1.09 (0.94-1.25)	0.26
HPFS	Median (%E)	38.7	44.6	48.5	52.3	58.1	
	Cases	818	780	848	857	877	
	Age-adjusted	1 *(ref.)*	0.93 (0.84-1.02)	0.95 (0.86-1.05)	0.91 (0.83-1.00)	0.88 (0.80-0.97)	0.009
	Multivariate*	1 *(ref.)*	0.98 (0.88-1.08)	1.02 (0.92-1.14)	0.99 (0.89-1.11)	1.00 (0.88-1.14)	0.92
Pooled	Multivariate*	1 *(ref.)*	0.99 (0.92-1.07)	1.02 (0.94-1.11)	1.01 (0.92-1.10)	1.04 (0.94-1.14)	0.41
**Carbohydrates from refined starches/added sugars**							
NHS	Median (%E)	14.8	19.4	22.5	25.5	30.3	
	Cases	594	683	712	762	741	
	Age-adjusted	1 *(ref.)*	1.00 (0.89-1.11)	1.03 (0.92-1.15)	1.14 (1.02-1.28)	1.24 (1.11-1.39)	<0.0001
	Multivariate *	1 *(ref.)*	1.01 (0.90-1.14)	1.02 (0.90-1.15)	1.10 (0.96-1.25)	1.10 (0.95-1.28)	0.15
HPFS	Median (%E)	16.7	20.9	23.8	26.8	31.6	
	Cases	849	836	833	824	833	
	Age-adjusted	1 *(ref.)*	1.00 (0.91-1.10)	1.04 (0.95-1.15)	1.07 (0.97-1.18)	1.21 (1.10-1.33)	<0.0001
	Multivariate *	1 *(ref.)*	1.02 (0.93-1.13)	1.04 (0.94-1.16)	1.04 (0.93-1.16)	1.10 (0.97-1.25)	0.15
Pooled	Multivariate *	1 *(ref.)*	1.02 (0.95-1.10)	1.03 (0.95-1.12)	1.06 (0.98-1.16)	1.10 (1.00-1.21)	0.04
**Carbohydrates from whole grains**							
NHS	Median (%E)	0.4	1.0	1.8	2.8	4.6	
	(%E) Cases	598	731	680	715	768	
	Age-adjusted	1 *(ref.)*	0.95 (0.85-1.06)	0.74 (0.66-0.83)	0.67 (0.60-0.76)	0.62 (0.55-0.69)	<0.0001
	Multivariate *	1 *(ref.)*	1.05 (0.94-1.18)	0.90 (0.80-1.01)	0.90 (0.79-1.02)	0.92 (0.81-1.05)	0.08
HPFS	Median (%E)	0.8	2.0	3.1	4.4	7.0	
	Cases	895	868	820	840	752	
	Age-adjusted	1 *(ref.)*	0.94 (0.85-1.03)	0.82 (0.74-0.90)	0.80 (0.73-0.88)	0.69 (0.63-0.76)	<0.0001
	Multivariate *	1 *(ref.)*	1.03 (0.93-1.13)	0.96 (0.87-1.07)	0.99 (0.90-1.10)	0.88 (0.79-0.99)	0.01

		Quintile Categories of Carbohydrate Intake as a Percentage of Total Energy Intake					p Trend
		**1**	**2**	**3**	**4**	**5**	
Pooled	Multivariate *	1 *(ref.)*	1.04 (0.96-1.11)	0.94 (0.87-1.01)	0.95 (0.88-1.03)	0.90 (0.83-0.98)	0.003

Abbreviations as in **Tables 1 and 2**.

* The multivariate model was adjusted for total energy intake, the energy contribution from protein, cholesterol intake, alcohol intake, smoking status, body mass index, physical activity, use of vitamins and aspirin, family history of MI and diabetes, and presence of baseline hypercholesterolemia and hypertension. Except for total carbohydrate models, models were also further adjusted for the percentage of energy from carbohydrates from whole grains, from refined starches/sugars, and from other foods simultaneously. Multivariate models thus represent the effect of substituting a percentage of energy from total carbohydrates or the specific carbohydrate type for an equivalent percentage of energy from total fat.

Author Manuscript Author Manuscript Author Manuscript Author Manuscript
