# Supplementary material for: A systematic comprehensive longitudinal evaluation of dietary factors associated with acute myocardial infarction and fatal coronary heart disease
Source: Nat Commun. 2020 Nov 27;11:6074. doi: 10.1038/s41467-020-19888-2 (PMC7699643; doi:10.1038/s41467-020-19888-2)
Supplement: Supplementary file 4 — Supplementary Software [file 41467_2020_19888_MOESM4_ESM.zip › EWAS-NHS-master/KG-SI/Rest/Liu (2000).pdf]

A prospective study of dietary glycemic load, carbohydrate intake, and risk of coronary heart disease in US women[1–3]

Simin Liu, Walter C Willett, Meir J Stampfer, Frank B Hu, Mary Franz, Laura Sampson, Charles H Hennekens, and JoAnn E Manson

ABSTRACT
Background: Little is known about the effects of the amount and type of carbohydrates on risk of coronary heart disease (CHD).
Objective: The objective of this study was to prospectively evaluate the relations of the amount and type of carbohydrates with risk of CHD.
Design: A cohort of 75 521 women aged 38–63 y with no previous diagnosis of diabetes mellitus, myocardial infarction, angina, stroke, or other cardiovascular diseases in 1984 was followed for 10 y. Each participant's dietary glycemic load was calculated as a function of glycemic index, carbohydrate content, and frequency of intake of individual foods reported on a validated food-frequency questionnaire at baseline. All dietary variables were updated in 1986 and 1990.
Results: During 10 y of follow-up (729 472 person-years), 761 cases of CHD (208 fatal and 553 nonfatal) were documented. Dietary glycemic load was directly associated with risk of CHD after adjustment for age, smoking status, total energy intake, and other coronary disease risk factors. The relative risks from the lowest to highest quintiles of glycemic load were 1.00, 1.01, 1.25, 1.51, and 1.98 (95% CI: 1.41, 2.77 for the highest quintile; *P* for trend < 0.0001). Carbohydrate classified by glycemic index, as opposed to its traditional classification as either simple or complex, was a better predictor of CHD risk. The association between dietary glycemic load and CHD risk was most evident among women with body weights above average [ie, body mass index (in kg/m^2) ≥ 23].
Conclusion: These epidemiologic data suggest that a high dietary glycemic load from refined carbohydrates increases the risk of CHD, independent of known coronary disease risk factors. *Am J Clin Nutr* 2000;71:1455–61.

KEY WORDS Diet, carbohydrate, fiber, glycemic load, glycemic index, coronary heart disease, Nurses' Health Study, women

INTRODUCTION

High intake of carbohydrates can raise plasma fasting triacylglycerol, primarily by enhancing hepatic synthesis of VLDL (1), and can also reduce HDL (2), thus creating an adverse lipid profile (3, 4). In addition, the negative effect of a low-fat, high-carbohydrate diet may be intensified by the underlying degree of insulin resistance (5). Because of adverse effects on lipid and glucose metabolism (6), it is uncertain whether a low-fat, high-carbohydrate diet is effective in the prevention of coronary heart disease (CHD) (7).

Carbohydrates with different physical forms, chemical structures, particle sizes, and fiber contents induce distinct plasma glucose and insulin responses (8). This physiologic response to carbohydrate can be quantified by glycemic indexes (8–10), which compare the plasma glucose response to specific foods with the response induced by the same amount of carbohydrate from a standard carbohydrate source, usually white bread or pure glucose. In metabolic studies and dietary trials, substituting foods with low glycemic indexes for those with high indexes reduced serum insulin and glucose responses (11–13), urinary C-peptide excretion (a marker of insulin production), and glycated hemoglobin concentrations in diabetic and nondiabetic subjects (9, 13–16). In addition, greater intake of starches with high glycemic indexes leads to insulin resistance in animals (17, 18) and is associated with insulin resistance (12, 13, 19), a lower concentration of HDL (13, 20), and hypertriglyceridemia (13, 14) in humans. Furthermore, a high dietary glycemic load (ie, the product of the glycemic index of a specific food and its carbohydrate content—a variable representing the quality and quantity of carbohydrate and the interaction between the 2) has been associated with an increased risk of type 2 diabetes (21, 22). Although the available evidence strongly indicates that insulin resistance, hyperglycemia, and associated disorders of lipid metabolism are important determinants of CHD (23–25), the association between dietary glycemic load and risk of CHD has not been examined in humans.

[1] From the Departments of Epidemiology and Nutrition, the Harvard School of Public Health; the Channing Laboratory; and the Division of Preventive Medicine, the Department of Medicine, Brigham and Women's Hospital and Harvard Medical School, Boston.

[2] Supported by CA40356, the main Nurses' Health Study Grant, and Nutrition Training Grant T32DK07703 from the US National Institutes of Health.

[3] Address reprint requests to S Liu, Division of Preventive Medicine, Department of Medicine, Harvard Medical School, 900 Commonwealth Avenue East, Boston, MA 02215. E-mail. simin.liu@channing.harvard.edu.

Received August 18, 1999.
Accepted for publication November 11, 1999.

Downloaded from https://academic.oup.com/ajcn/article-abstract/71/6/1455/4729405 by Northeastern University Libraries user on 23 May 2019

In this 10-y follow-up study of female nurses, we examined prospectively *1*) whether dietary glycemic load is related to risk of CHD, *2*) whether the glycemic index can predict risk of CHD better than can the traditional classification of carbohydrates into simple and complex forms, and *3*) whether the relation of glycemic load to risk of CHD is modified by adiposity.

SUBJECTS AND METHODS

The Nurses' Health Study was initiated in 1976 when 121 700 female registered nurses aged 30–55 y answered a mailed questionnaire about their medical histories and lifestyles. Since then, the cohort has been followed up every 2 y to ascertain exposure and incident diseases. In 1984, we collected dietary information with a 126-item semiquantitative food-frequency questionnaire (SFFQ) that included detailed assessment of carbohydrate-containing foods. After >4 mailings, 81 757 women returned the SFFQ and satisfied a priori criteria of daily energy intakes between 2514 kJ (600 kcal) and 14 665 kJ (3500 kcal). We further excluded women with previously diagnosed diabetes (n = 2248) and cardiovascular disease [including angina, myocardial infarction (MI), stroke, and other cardiovascular diseases; n = 3122]. The final baseline population was 75 521 women aged 38–63 y in 1984. The study was conducted according to the ethical guidelines of Brigham and Women's Hospital, Boston.

Measurements of dietary intake were repeated in 1986 and 1990 by using almost identical SFFQs. For each food, a commonly used unit or portion size (eg, one slice of bread) was specified and the subject was asked how often during the previous year, on average, she had consumed that amount. Nine responses were possible, ranging from "never" to "≥6 times per day." Nutrient scores were computed by multiplying the frequency of consumption of each unit of food from the SFFQ by the nutrient content of the specified portion according to food-composition tables from the US Department of Agriculture (26) and other sources. A full description of the SFFQ and the procedures used for calculating nutrient intake, as well as data on reproducibility and validity in this cohort, were reported previously (27). The correlation coefficient for energy-adjusted carbohydrate intake between the SFFQs and diet records was 0.73. The performance of the SFFQ for assessing the individual foods high in carbohydrate has also been documented (28). For example, correlation coefficients were 0.71 for white bread, 0.77 for dark bread, 0.66 for potatoes, and 0.94 for yogurt (28).

The method used to assess glycemic indexes of individual foods and mixed meals, as well as the measurement of dietary glycemic load in the Nurses' Health Study cohort, were reported elsewhere (10, 15, 21). Briefly, we calculated glycemic load by multiplying the carbohydrate content of each food by its glycemic index, then multiplied this value by the frequency of consumption and summed the values from all foods. Dietary glycemic load thus represents the quality and quantity of carbohydrates and the interaction between the 2, given that the product of glycemic index and carbohydrate intake indicates that a higher glycemic index has a greater effect at higher carbohydrate intakes. Each unit of dietary glycemic load represents the equivalent of 1 g carbohydrate from white bread or pure glucose. We also created a variable we termed overall dietary glycemic index by dividing the average daily glycemic load by the average daily carbohydrate intake. Expression of the glycemic load per unit of carbohydrate allowed the carbohydrate content to be matched gram by gram and thus reflects the overall quality of carbohydrate intake.

The primary endpoint for this analysis was incident CHD, which includes fatal CHD and nonfatal MI occurring during the 10-y period between the return of the 1984 questionnaire and 1 June 1994 (29). A diagnosis of nonfatal MI was confirmed by medical records by using criteria proposed by the World Health Organization—symptoms plus either typical electrocardiographic changes or elevation of cardiac enzymes (29). Fatal CHD was ascertained by using the National Death Index and was confirmed by medical records, autopsy reports, or death certificates. Fatal CHD was confirmed if CHD was listed as the cause of death, if it was the underlying and most plausible cause, or if evidence of previous CHD was available. On the basis of all sources combined, mortality follow-up was >98% complete (30).

Statistical analysis

For each study participant, person-years of follow-up (ie, the number of persons studied times the number of years of follow-up) were counted from the date of return of the 1984 questionnaire to the date of CHD diagnosis; the date of death; or 1 June 1994, whichever came first. Women were grouped in quintiles of glycemic load, overall dietary glycemic index, and carbohydrate intake (simple versus complex). Incidence rates were calculated as the number of CHD events divided by the person-time of follow-up in each quintile. Incidence rate ratios were calculated by dividing the incidence rate of CHD in a particular category of exposure by the corresponding rate in the reference category. Tests for trends were conducted by assigning the median value to each quintile and modeling this value as a continuous variable. The log likelihood ratio test was used to assess the significance of interaction terms.

In a multivariate analysis, the estimated relative risks (RRs) were simultaneously adjusted for potential confounding variables by using a pooled logistic regression that was asymptotically equivalent to the Cox proportional-hazards regression (31). To best represent the participants' long-term dietary patterns during follow-up, we used a cumulative average method based on all available measurements of diet up to the beginning of each 2-y interval (32, 33). Other covariates, including age, body mass index (BMI; in kg/m^2), smoking status, alcohol intake, physical activity, postmenopausal hormone use, multivitamin use, use of vitamin E supplements, parental history of MI before age 60 y, history of hypertension, and history of hypercholesterolemia, were assessed and updated every 2 y. To control for total energy intake, all nutrients, glycemic load, and overall dietary glycemic index were adjusted for total energy intake by using the residual method (34). In addition, when examining the effect of substituting carbohydrate for fat, we used multivariate nutrient-density models that simultaneously included energy intake, the percentages of energy derived from protein and carbohydrate, and other confounding variables. All reported P values are two-sided. Furthermore, because adiposity (BMI) is an important determinant of insulin resistance (35), we hypothesized a priori that adiposity could modify the relation between glycemic load and CHD and we evaluated this hypothesis in stratified analyses.

RESULTS

At baseline in 1984, the mean dietary glycemic load varied nearly 2-fold between the highest and lowest quintiles of the study population (**Table 1**). Women with high dietary glycemic

Downloaded from https://academic.oup.com/ajcn/article-abstract/71/6/1455/4729405 by Northeastern University Libraries user on 23 May 2019

TABLE 1

Age-standardized baseline characteristics according to quintiles of energy-adjusted glycemic load among 75 521 US female nurses aged 38–63 y, 1984[1]

	Quintile of glycemic load score				
Variable	1	2	3	4	5
Quintile mean score	117	145	161	177	206
Parental myocardial infarction before age 60 y (%)	15	15	15	15	14
Vigorous activity at least once/wk (%)	43	44	44	43	43
Current smoker (%)	33	26	22	20	20
Current postmenopausal hormone use (%)	12	12	11	11	11
Exercise (h/wk)	3.2 ± 2.1[2]	3.2 ± 2.1	3.2 ± 2.1	3.2 ± 2.1	3.2 ± 2.1
BMI (kg/m^2)	25 ± 5	25 ± 5	25 ± 5	25 ± 4	24 ± 4
Daily dietary intake[3]					
Total energy (kJ)	7113 ± 2244	7453 ± 2211	7515 ± 2206	7386 ± 2198	7005 ± 2189
Glycemic index[4]	72 ± 6	75 ± 4	77 ± 4	78 ± 4	80 ± 4
Carbohydrate (g)	144 ± 20	171 ± 11	186 ± 11	200 ± 11	226 ± 20
Polyunsaturated fat (g)	13 ± 4	12 ± 3	12 ± 3	11 ± 3	10 ± 3
Monounsaturated fat (g)	25 ± 5	24 ± 4	23 ± 3	21 ± 3	19 ± 3
Saturated fat (g)	25 ± 5	24 ± 4	22 ± 4	21 ± 3	18 ± 4
Trans fatty acids (g)	4 ± 1	4 ± 1	4 ± 1	3 ± 1	3 ± 1
Cholesterol (mg)	337 ± 123	306 ± 88	287 ± 77	266 ± 71	228 ± 74
Protein (g)	79 ± 15	75 ± 11	72 ± 11	68 ± 10	62 ± 10
Dietary fiber (g)	14 ± 4	16 ± 4	17 ± 4	17 ± 5	18 ± 6
Cereal fiber (g)	3 ± 2	4 ± 2	4 ± 2	5 ± 2	5 ± 2
Alcohol (g)	16 ± 18	8 ± 10	6 ± 8	4 ± 6	3 ± 5
Dietary vitamin E (mg)	7 ± 4	7 ± 3	7 ± 3	6 ± 3	6 ± 4
Folate (μg)	367 ± 240	373 ± 220	378 ± 215	393 ± 226	407 ± 250
Foods (servings/d)					
Cooked potatoes (mashed or baked)	0.24	0.31	0.33	0.35	0.37
Dark bread	0.47	0.59	0.63	0.67	0.66
White bread	0.49	0.60	0.68	0.69	0.67
Orange juice	0.30	0.41	0.46	0.52	0.58
White rice	0.09	0.10	0.11	0.11	0.12
Pizza	0.06	0.07	0.07	0.07	0.06
Cold breakfast cereal	0.14	0.22	0.28	0.32	0.38
Bananas	0.14	0.20	0.23	0.26	0.31
Pasta	0.13	0.15	0.15	0.16	0.15

[1] Glycemic load was defined as an indicator of blood glucose induced by an individual's total carbohydrate intake. Each unit of glycemic load represents the equivalent of 1 g carbohydrate from white bread.

[2] $\bar{x}$ ± SD.

[3] All dietary variables, including glycemic load and glycemic index, were adjusted for total energy intake.

[4] Glycemic index was defined as glycemic load divided by the total amount of carbohydrate.

loads consumed more carbohydrates, dietary fiber, cereal fiber, vitamin E, and folate but had lower intakes of fats, cholesterol, proteins, and alcohol and smoked less than did women with low glycemic loads (Table 1). Mean age and BMI, history of parental MI before age 60 y, physical activity, and current use of postmenopausal hormones did not vary appreciably across quintiles of dietary glycemic load. Dietary glycemic load did not appear to be determined by any particular food; the 2 most important contributors to dietary glycemic load in this population were mashed or baked potatoes (8%) and cold breakfast cereals (4%); other carbohydrate-containing foods contributed smaller amounts.

During 729 472 person-years of follow-up, we documented 761 cases of CHD (208 fatal and 553 nonfatal MIs). After adjustment for age and smoking status (model X), the estimated RR for women in the highest quintile compared with those in the lowest quintile of energy-adjusted dietary glycemic load was 1.57 (95% CI, 1.27, 1.95; *P* for trend < 0.0001) (**Table 2**). This association remained essentially unchanged after further adjustment for other known coronary disease risk factors. In an analysis that included age, BMI, smoking status, alcohol intake, parental family history of MI before age 60 y, self-reported history of hypertension, history of high cholesterol, menopausal status, aspirin use, use of multiple vitamins, use of vitamin E supplements, physical activity, protein intake, dietary fiber, dietary vitamin E and folate, and total energy intake (model 2, Table 2), the RR for the highest compared with the lowest quintile of dietary glycemic load was 1.56 (95% CI: 1.17, 2.07; *P* for trend < 0.0001).

We further adjusted for saturated, monounsaturated, polyunsaturated, and *trans* fats (model 4, Table 2). In this model, in which all fats, protein, and total energy intake were held constant, glycemic load represented the effect of substituting high-glycemic-index carbohydrates for low-glycemic-index carbohydrates on CHD risk. Compared with carbohydrates with a low glycemic index, carbohydrates with a high glycemic index were strongly associated with increased risk of CHD. The RR for the highest compared with the lowest quintile of glycemic load was 1.98 (95% CI, 1.41, 2.77; *P* for trend < 0.0001) in this model. Similar findings for glycemic load were observed when all types of fats were replaced with carbohydrate in the multivariate model, with a multivariate adjusted RR of 1.89 (95% CI: 1.45,

Downloaded from https://academic.oup.com/ajcn/article-abstract/71/6/1455/4729405 by Northeastern University Libraries user on 23 May 2019

TABLE 2

Adjusted relative risks (with 95% CIs) of coronary heart disease (CHD) according to quintiles of energy-adjusted glycemic load among 75 521 US female nurses aged 38–63 y, 1984–1994

	Quintile of energy-adjusted dietary glycemic load score				
	1 (lowest)	2	3	4	5 (highest)
Cases of CHD	139	128	148	160	186
Person-years	147 341	141 515	146 413	149 977	144 226
Relative risk (95% CI)					
Model 1: adjusted for age and smoking[1]	1.00	0.92 (0.73, 1.17)	1.08 (0.85, 1.36)	1.27 (1.01, 1.59)	1.57 (1.27, 1.95)
Model 2: multivariate (without fats)[1,2]	1.00	0.94 (0.73, 1.20)	1.11 (0.86, 1.43)	1.28 (0.98, 1.66)	1.56 (1.17, 2.07)
Model 3: multivariate with additional adjustment for saturated and *trans* fats[1]	1.00	0.97 (0.76, 1.25)	1.19 (0.92, 1.55)	1.42 (1.07, 1.88)	1.85 (1.34, 2.54)
Model 4: multivariate with additional adjustment for all fats[1]	1.00	1.01 (0.78, 1.29)	1.25 (0.96, 1.64)	1.51 (1.13, 2.03)	1.98 (1.41, 2.77)

[1] $P < 0.0001$ for trend.

[2] Includes the following: age (5-y categories); BMI (6 categories); cigarette smoking (never, past, and current smoking of 1–14, 15–24, and ≥25 cigarettes/d; alcohol intake (4 categories); parental family history of myocardial infarction before the age of 60 y; self-reported history of hypertension or history of high cholesterol; menopausal status (premenopausal, postmenopausal without hormone replacement, postmenopausal with past hormone replacement, and postmenopausal with current hormone replacement); aspirin use (4 categories); use of multiple vitamin or vitamin E supplement; physical activity (h/wk in 5 categories); protein intake (in quintiles); dietary fiber, vitamin E, and folate intakes (in quintiles); and total energy intake (in quintiles).

2.46; *P* for trend < 0.0001) when extreme quintiles of glycemic load were compared.

To better understand the relation of dietary glycemic load to risk of CHD in terms of its constituents, we examined intake of total and specific types of carbohydrates. In model 1 (**Table 3**), based only on food-composition data (without incorporation of glycemic index data of individual foods), total carbohydrate intake, representing the replacement of fat with carbohydrate, appeared to be positively related to CHD risk, but this association was weak and not significant. When total carbohydrate was entered into the multivariate nutrient-density model as a continuous variable, the RR was 1.02 (95% CI: 0.96, 1.08; *P* = 0.50) for an increase of 5% in energy from total carbohydrate, as compared with the equivalent energy from total fat.

Carbohydrates have traditionally been classified as simple (monosaccharides and disaccharides) or complex (polysaccharides, mainly starch) (36). We examined the relation of these mutually exclusive types of carbohydrate to risk of CHD. Neither simple sugar nor starch was significantly related to CHD risk when they were included simultaneously in the same multivariate model (model 2, Table 3). In contrast, the quality of carbohydrate as classified by its glycemic index was significantly associated with the risk of CHD in a multivariate model, in which the same covariates were adjusted for [multivariate adjusted RR: 1.31; 95% CI: 1.02, 1.68; *P* for trend = 0.008 when extreme quintiles were compared (model 3, Table 3)]. Addition of total carbohydrate intake to this model did not change the positive association between overall dietary glycemic index and CHD risk (RR: 1.28; 95% CI: 1.00, 1.64; *P* for trend = 0.02).

The increased risk of CHD associated with high glycemic load was most evident among women with BMIs > 23 (**Figure 1**). Little relation between glycemic load and CHD risk was found among women with BMIs < 23 (RR: 1.11; 95% CI: 0.74, 1.66 for high compared with low glycemic load; *P* < 0.01 for test of interaction between BMI and glycemic load).

DISCUSSION

In this 10-y follow-up study of 75 521 female nurses, we found a significant positive association between dietary glycemic load and risk of CHD that was independent of known coronary disease risk factors, including other measured dietary variables. In addition, glycemic index was a stronger predictor of CHD risk than was the usual classification of simple versus complex carbohydrates. The adverse effect of a high dietary glycemic load was most evident among women with average or above-average body weights.

The prospective design of this study eliminated many potential sources of bias, especially recall bias. One concern was whether women lost to follow-up had disease and exposure experiences that were different from those of the women who remained in the cohort. However, because loss to follow-up accounted for only ≈1.8% of the person-years, any potential bias would have been minimal. Although residual confounding by unknown risk factors is theoretically possible, higher glycemic load was not associated with a generally less favorable risk profile. A clear association between dietary glycemic load and risk was evident after adjustment for age and smoking status. This association became stronger with additional adjustment of a multitude of confounding factors, arguing against the possibility of residual confounding.

Misclassification of dietary exposure is always an important concern. Random within-person variation could attenuate any association of interest, but the SFFQ was designed to minimize this kind of error by assessing average long-term dietary intake. Although the total glycemic effect of diets may not be fully captured by the SFFQ, any measurement errors would be expected to be unrelated to the CHD endpoints because of the prospective design of the Nurses' Health Study. Thus, our estimate of the underlying association between glycemic load and risk of CHD may be somewhat conservative. Furthermore, because updated dietary information during the follow-up was used in our analyses, we accounted for changes in dietary habits over time.

The results of 2 previous cohort studies of men suggested a weak inverse association between intake of carbohydrate and CHD (37, 38). In the Honolulu Heart Program, McGee et al (37) documented 456 cases of CHD among 8000 men of Japanese ancestry who were followed up for 10 y. Measuring diets at baseline with a single 24-h recall, these authors showed that men who later experienced coronary events had lower intakes of energy

Downloaded from https://academic.oup.com/ajcn/article-abstract/71/6/1455/4729405 by Northeastern University Libraries user on 23 May 2019

TABLE 3
Adjusted relative risks (with 95% CIs) of coronary heart disease according to quintiles of energy-adjusted total carbohydrate, type of carbohydrate, and glycemic index among 75 521 US female nurses aged 38–63 y, 1984–1994[1]

	Quintiles of energy-adjusted score				
Variable	1 (lowest)	2	3	4	5 (highest)
Model 1: total carbohydrate	1.00	1.02 (0.80, 1.29)	1.09 (0.84, 1.42)	1.03 (0.76, 1.39)	1.23 (0.86, 1.75)
Model 2: carbohydrate classified by its chemical structure (all types of carbohydrate included simultaneously)[2]					
Starch	1.00	0.96 (0.76, 1.20)	0.91 (0.70, 1.15)	0.90 (0.69, 1.18)	0.94 (0.69, 1.28)
Sucrose	1.00	1.03 (0.81, 1.31)	1.16 (0.91, 1.48)	1.02 (0.78, 1.33)	1.22 (0.94, 1.60)
Fructose	1.00	0.91 (0.72, 1.15)	0.96 (0.75, 1.22)	1.11 (0.87, 1.42)	1.07 (0.82, 1.40)
Lactose	1.00	1.06 (0.85, 1.32)	0.94 (0.70, 1.19)	1.12 (0.88, 1.41)	0.94 (0.74, 1.21)
Model 3: carbohydrate classified by its ability to raise plasma glucose response					
Glycemic index[3]	1.00	1.01 (0.79, 1.29)	1.16 (0.91, 1.47)	1.18 (0.92, 1.49)	1.31 (1.02, 1.68)

[1] All the models were simultaneously adjusted for age (5-y categories); BMI (6 categories); cigarette smoking (never, past, and current smoking of 1–14, 15–24, and ≥25 cigarettes/d); alcohol intake (4 categories); parental family history of myocardial infarction before the age of 60 y; self-reported history of hypertension or history of high cholesterol; menopausal status (premenopausal, postmenopausal without hormone replacement, postmenopausal with past hormone replacement, and postmenopausal with current hormone replacement); aspirin use (4 categories); use of multiple vitamin or vitamin E supplement; physical activity (h/wk in 5 categories); protein intake (in quintiles); intake of saturated, polyunsaturated, and *trans* fats; dietary fiber intake; dietary vitamin E and folate intakes (all in quintiles); and total energy intake (in quintiles).

[2] Types of carbohydrate were mutually exclusive; fructose does not include contribution from sucrose.

[3] *P* for trend = 0.008.

and carbohydrates than did the rest of the cohort. However, no significant associations were found in multivariate analysis when both total energy and carbohydrate intakes were included in the same model. In The Puerto Rico Heart Health Program, Garcia-Palmieri et al (38) measured baseline diets with a 24-h recall among 8218 men and found a significant inverse association between carbohydrate intake and CHD. However, these findings might have resulted from the lack of adjustment for total energy intake. Such an adjustment is important because variation in total energy intake is largely determined by physical activity, a known protective factor against CHD. Neither study measured dietary glycemic load or conducted stratified analysis to examine the effects of carbohydrate intake on MI risk among subgroups of men defined by body weight. In metabolic trials, low-fat, high-carbohydrate diets neither increased insulin sensitivity nor maintained weight loss in diabetic subjects (39, 40) or nondiabetic subjects (41, 42). These diets did, however, worsen dyslipidemia (41, 42), especially among subjects who were prone to insulin resistance (5).

Insulin resistance, hyperglycemia, and related metabolic disorders have long been recognized as important risk factors for CHD (43, 44). In clinical observations and prospective cohorts, diabetic patients have a greater risk of morbidity and mortality from CHD than do nondiabetic patients (25, 45), especially women (25, 46). In the Framingham

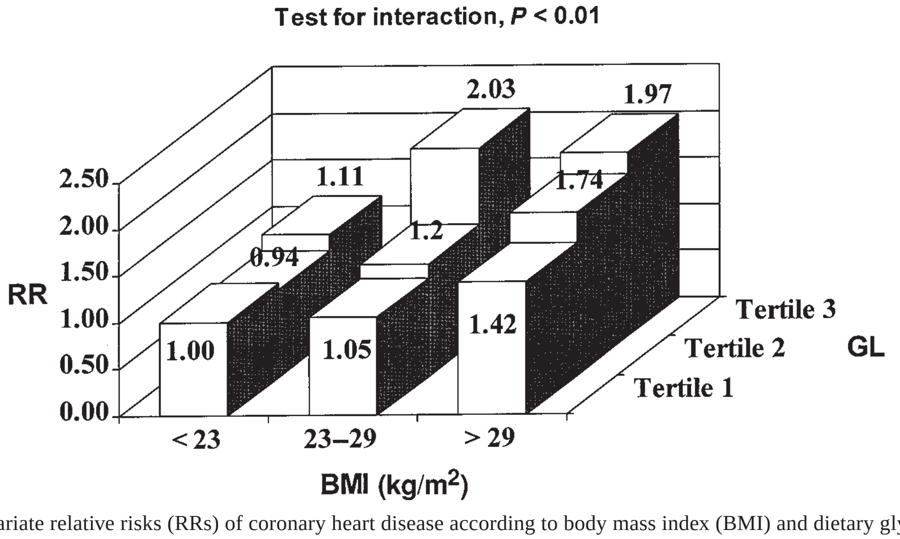

FIGURE 1. Multivariate relative risks (RRs) of coronary heart disease according to body mass index (BMI) and dietary glycemic load (GL). The GL is stratified by tertiles and the RRs are adjusted for the same covariates as those in Table 3. The 95% CIs for the 9 RRs are as follows: 1.00 (referent), 1.05 (0.76–1.45), 1.42 (0.96–2.08), 0.94 (0.63–1.40), 1.20 (0.86–1.68), 1.74 (1.18–2.55), 1.11 (0.74–1.66), 2.02 (1.45–2.83), and 1.97 (1.31–2.96).

Downloaded from https://academic.oup.com/ajcn/article-abstract/71/6/1455/4729405 by Northeastern University Libraries user on 23 May 2019

cohort, a strong positive association was observed between prevalence of CHD and increased glycated hemoglobin concentration, which suggests the important role of hyperglycemia in the development of CHD (47). In the Whitehall study (48), impaired glucose tolerance was associated with increased risk of MI. More recent studies also suggested that the relation between glycated hemoglobin concentration and CHD risk is progressive and continuous in diabetic (49) and nondiabetic (24) subjects. Although the underlying biological mechanisms for our findings remain to be elucidated, a high dietary glycemic load apparently induces hyperglycemia and hyperinsulinemia (9, 13, 50), which can lead, in turn, to hypertension (51), dyslipidemia (52), and possibly impaired fibrinolysis and thrombosis (53, 54), all of which can increase the risk of CHD.

The adverse metabolic responses to diets with a high glycemic load (9, 13, 16, 50) are exacerbated by underlying insulin resistance because increasing amounts of insulin are needed to compensate for the increased glycemic load (13). Our results are consistent with other published reports indicating that high glycemic load does not appear to affect risk of CHD among women with low BMIs (<23) (Figure 1). This finding may partly explain why some populations, such as those in rural China, have low rates of CHD despite high carbohydrate intakes. Traditionally, these populations consume carbohydrates in less refined forms, have high amounts of physical activity, and have a low prevalence of obesity. These factors can improve insulin sensitivity and may lead to greater tolerance of a relatively high glycemic load (25).

Although almost all existing dietary guidelines advise the substitution of complex carbohydrates for simple sugars or fat (55, 56), data that directly relate types of carbohydrate to CHD risk are limited. Our findings cast doubt on the usefulness of the simple versus complex classification of carbohydrates. A better measure would be the glycemic index of carbohydrates, which, in the present study, was more closely related with CHD risk—carbohydrates with a high glycemic index were strongly associated with increased risk of CHD compared with carbohydrates with a low glycemic index after intakes of total energy, protein, fats, and carbohydrates were held constant (model 4, Table 2). Metabolic data indicate that starchy foods, such as white rice and potatoes, are digested and absorbed quickly. Thus, even though these foods are considered to be desirable complex carbohydrates, they each have a high glycemic index and can induce high blood glucose responses. In contrast, the major source of simple sugars in this cohort was fruit and vegetables. Although they are considered to be less-than-desirable simple carbohydrates, these disaccharides (70% fructose and 33% sucrose) have a low glycemic index and yield only a small to moderate blood glucose response (57).

In summary, our findings suggest that a high intake of rapidly digested and absorbed carbohydrate increases the risk of CHD independent of conventional coronary disease risk factors. These data add to the concern that the current low-fat, high-carbohydrate diet recommended in the United States may not be optimal for the prevention of CHD and could actually increase the risk in individuals with high degrees of insulin resistance and glucose intolerance.

We thank the participants in the Nurses' Health Study for their continuing exceptional cooperation; Al Wing, Gary Chase, Karen Corsano, Lisa Dunn, Barbara Egan, Lori Ward, and Jill Arnold for their unfailing help; Eric Rimm, Alberto Ascherio, Graham Colditz, Frank Sacks, and Ed Giovannucci for their insightful comments; and Frank Speizer, principal investigator of the Nurses' Health Study, for his support.

REFERENCES

1. Nestel PJ, Carroll KF, Havenstein N. Plasma triglyceride response to carbohydrates, fats and caloric intake. Metabolism 1970;19:1–18.
2. Mancini M, Mattock M, Rabaya E, Chait A, Lewis B. Studies of the mechanisms of carbohydrate-induced lipaemia in normal man. Atherosclerosis 1973;17:445–54.
3. Stampfer MJ, Krauss RM, Ma J, et al. A prospective study of triglyceride level, low-density lipoprotein particle diameter, and risk of myocardial infarction. JAMA 1996;276:882–8.
4. Hokanson J, Austin M. Plasma triglyceride level is a risk factor for cardiovascular disease independent of high-density lipoprotein cholesterol level: a meta-analysis of population-based prospective studies. J Cardiovasc Risk 1996;3:213–9.
5. Jeppesen J, Schaaf P, Jones C, Zhou MY, Chen YD, Reaven GM. Effects of low-fat, high-carbohydrate diets on risk factors for ischemic heart disease in postmenopausal women. Am J Clin Nutr 1997;65:1027–33. (Published erratum appears in Am J Clin Nutr 1997;66:437.)
6. Willett WC, Lenart EB. Dietary factors. In: Manson JE, Ridker PM, Gaziano JM, Hennekens CH, eds. Prevention of myocardial infarction. New York: Oxford University Press, 1996:351–83.
7. Katan MB, Grundy SM, Willett WC. Should a low-fat, high-carbohydrate diet be recommended for everyone? Beyond low-fat diets. N Engl J Med 1997;337:563–7.
8. Jenkins DJ, Wolever TM, Taylor RH, et al. Glycemic index of foods: a physiological basis for carbohydrate exchange. Am J Clin Nutr 1981;34:362–6.
9. Wolever T. The glycemic index. In: Bourne G, ed. Aspects of some vitamins, minerals and enzymes in health and disease. Basel, Switzerland: Karger, 1990:120–85.
10. Foster-Powell K, Miller JB. International tables of glycemic index. Am J Clin Nutr 1995;62(suppl):871S–93S.
11. Wolever TMS, Jenkins DJA. The use of the glycemic index in predicting the blood glucose response to mixed meals. Am J Clin Nutr 1986;43:167–72.
12. Slabber M, Barnard HC, Kuyl JM, Dannhauser A, Schall R. Effects of a low-insulin-response, energy-restricted diet on weight loss and plasma insulin concentrations in hyperinsulinemic obese females. Am J Clin Nutr 1994;60:48–53.
13. Brand Miller JC. Importance of glycemic index in diabetes. Am J Clin Nutr 1994;59(suppl):747S–52S.
14. Jenkins D, Wolever T, Kalmusky J. Low-glycemic index diet in hyperlipidemia: use of traditional starchy foods. Am J Clin Nutr 1987;46:66–71.
15. Wolever TMS, Jenkins DJ, Jenkins AL, Josse RG. The glycemic index: methodology and clinical implications. Am J Clin Nutr 1991;54:846–54.
16. Wolever T, Bolognesi C. Prediction of glucose and insulin responses of normal subjects after consuming mixed meals varying in energy, protein, fat, carbohydrate and glycemic index. Nutrition 1992;126:2807–12.
17. Byrnes SE, Miller JCB, Denyer GS. Amylopectin starch promotes the development of insulin resistance in rats. J Nutr 1995;125:1430–7.
18. Wiseman C, Higgins J, Denyer G, Brand Miller J. Amylopectin starch induces nonreversible insulin resistance in rats. J Nutr 1996;126:410–5.
19. Frost G, Leeds A, Trew G, Margara R, Dornhorst A. Insulin sensitivity in women at risk of coronary heart disease and the effect of a low glycemic diet. Metabolism 1998;47:1245–51.
20. Frost G, Leeds A, Dore D, Madeiros S, Brading S, Dornhorst A. Glycemic index as a determinant of serum HDL-cholesterol concentration. Lancet 1999;353:1045–8.
21. Salmeron J, Manson JE, Stampfer MJ, Colditz GA, Wing AL, Willett WC. Dietary fiber, glycemic load, and risk of non-insulin-dependent diabetes mellitus in women. JAMA 1997;277:472–7.
22. Salmeron J, Ascherio A, Rimm EB, et al. Dietary fiber, glycemic load, and risk of NIDDM in men. Diabetes Care 1997;20:545–50.

Downloaded from https://academic.oup.com/ajcn/article-abstract/71/6/1455/4729405 by Northeastern University Libraries user on 23 May 2019

23. Reaven GM. Role of insulin resistance in human disease. Diabetes 1988;37:1595–607.
24. Vitelli LL, Shahar E, Heiss G, et al. Glycosylated hemoglobin level and carotid intimal-medial thickening in non-diabetic individuals. Diabetes Care 1997;20:1454–8.
25. Liu S. Insulin resistance, hyperglycemia and risk of major chronic diseases—a dietary perspective. Proc Nutr Soc Aust 1998;22:140–50.
26. US Department of Agriculture. Composition of foods—raw, processed, and prepared, 1963–1988. Agricultural handbook no. 8 series. Washington, DC: Department of Agriculture, US Government Printing Office, 1989.
27. Willett WC. Nutritional epidemology. 2nd ed. New York: Oxford University Press, 1998.
28. Salvini S, Hunter DJ, Sampson L, et al. Food-based validation of a dietary questionnaire: the effects of week-to-week variation in food consumption. Int J Epidemiol 1989;18:858–67.
29. Rose GA, Blackburn H. Cardiovascular survey methods. World Health Organ Monogr Ser 1982; 58.
30. Stampfer MJ, Willett WC, Speizer FE, et al. Test of the National Death Index. Am J Epidemiol 1984;119:837–9.
31. Cupples LA, D'Agostino RB, Anderson K, Kannel WB. Comparison of baseline and repeated measure covariate techniques in the Framingham Heart Study. Stat Med 1988;7:205–222.
32. Hu FB, Stampfer MJ, Manson JE, et al. Dietary fat intake and the risk of coronary heart disease in women. N Engl J Med 1997; 337:1491–9.
33. Liu S, Stampfer MJ, Hu FB, et al. Whole-grain consumption and risk of coronary heart disease: results from the Nurses' Health Study. Am J Clin Nutr 1999;70:412–9.
34. Willett WC, Stampfer MJ. Total energy intake: implications for epidemiologic analyses. Am J Epidemiol 1986;124:17–27.
35. Facchini FS, Hollenbeck CB, Jeppesen J, Chen Y-DI, Reaven GM. Insulin resistance and cigarette smoking. Lancet 1992;339: 1128–30.
36. Murray RK, Granner DK, Mayes PA, Rodwell VW. Harper's biochemistry. 23rd ed. East Norwalk, Connecticut: Appleton & Lange, 1993.
37. McGee DL, Reed DM, Yano K, Kagan A, Tillotson J. Ten-year incidence of coronary heart disease in the Honolulu Heart Program: relationship to nutrient intake. Am J Epidemiol 1984;119:667–76.
38. Garcia-Palmieri MR, Sorlie P, Tillotson J, Costas R Jr, Cordero E, Rodriguez M. Relationship of dietary intake to subsequent coronary heart disease incidence: The Puerto Rico Heart Health Program. Am J Clin Nutr 1980;33:1818–27.
39. Garg A, Grundy SM, Koffler M. Effect of high carbohydrate intake on hyperglycemia, islet cell function, and plasma lipoproteins in NIDDM. Diabetes Care 1992;15:1572–80.
40. Garg A, Bantle JP, Henry RR, et al. Effects of varying carbohydrate content of diet in patients with non-insulin-dependent diabetes mellitus. JAMA 1994;271:1421–8.
41. Jeppesen J, Chen YD, Zhou MY, Wang T, Reaven GM. Effect of variations in oral fat and carbohydrate load on postprandial lipemia. Am J Clin Nutr 1995;62:1201–5.
42. Knopp RH, Walden CE, Retzlaff BM, et al. Long-term cholesterol-lowering effects of 4 fat-restricted diets in hypercholesterolemic and combined hyperlipidemic men: The Dietary Alternatives Study. JAMA 1997;278:1509–15.
43. Haffner SM, Fong D, Hazuda HP, Pugh JA, Patterson JK. Hyperinsulinemia, upper body adiposity, and cardiovascular risk factors in non-diabetics. Metabolism 1988;37:338–45.
44. Stout MP. Insulin and atheroma. 20-yr perspective. Diabetes Care 1990;13:631–54.
45. Stern MP. Diabetes and cardiovascular disease: the 'common soil' hypothesis. Diabetes 1995;44:369–74.
46. Barrett-Connor EL, Cohn BA, Wingard DL, Edelstein SL. Why is diabetes mellitus a stronger risk factor for fatal ischemic heart disease in women than in men? JAMA 1991;265:627–31.
47. Singer DE, Nathan DM, Anderson KM, Wilson PW, Evans JC. Association of HbA1c with prevalent cardiovascular disease in the original cohort of the Framingham Heart Study. Diabetes 1992;41: 202–8.
48. Fuller JH, Shipley MJ, Rose G, Jarrett RJ, Keen H. Mortality from coronary heart disease and stroke in relation to degree of glycemia: the Whitehall study. Br Med J (Clin Res Ed) 1983;287: 867–70.
49. Moss SE, Klein R, Klein BEK, Meuer SM. The association of glycemia and cause-specific mortality in a diabetic population. Arch Intern Med 1994;154:2473–9.
50. Ludwig DS, Majzoub JA, Al-Zahrani A, Dallal GE, Blanco I, Roberts SB. High glycemic index foods, overeating, and obesity. Pediatrics 1999;103:E26.
51. Ferrannini E, Buzzigoli G, Bonadonna Rea. Insulin resistance in essential hypertension. N Engl J Med 1987;317:350–7.
52. Laws A, Reaven GM. Evidence for an independent relationship between insulin resistance and fasting plasma HDL-cholesterol, triglyceride and insulin concentrations. J Intern Med 1992;231:25–30.
53. Juhan-Vague I, Alessi MC, Joly P, et al. Plasma plasminogen activator inhibitor-1 in angina pectoris: influence of plasma insulin and acute-phase response. Arteriosclerosis 1989;9:362–7.
54. Gerstein HC, Yusuf S. Dysglycaemia and risk of cardiovascular disease. Lancet 1996;347:949–50.
55. American Heart Association. Dietary guidelines for healthy American adults. Circulation 1996;94:1795–800.
56. US Department of Agriculture, US Department of Health and Human Services. Nutrition and your health: dietary guidelines for Americans. Washington, DC: US Government Printing Office, 1995.
57. Miller JB, Pang E, Broomhead L. The glycemic index of foods containing sugars: comparison of foods with naturally-occurring v. added sugars. Br J Nutr 1995;73:613–23.

Downloaded from https://academic.oup.com/ajcn/article-abstract/71/6/1455/4729405 by Northeastern University Libraries user on 23 May 2019
