# Supplementary material for: A systematic comprehensive longitudinal evaluation of dietary factors associated with acute myocardial infarction and fatal coronary heart disease
Source: Nat Commun. 2020 Nov 27;11:6074. doi: 10.1038/s41467-020-19888-2 (PMC7699643; doi:10.1038/s41467-020-19888-2)
Supplement: Supplementary file 4 — Supplementary Software [file 41467_2020_19888_MOESM4_ESM.zip › EWAS-NHS-master/KG-SI/Rest/Ma (2018).pdf]

International Journal of Epidemiology, 2018, 311–320
doi: 10.1093/ije/dyx234
Advance Access Publication Date: 13 November 2017
Original article

Miscellaneous

Dietary glutamine, glutamate and mortality: two large prospective studies in US men and women

Wenjie Ma,[1] Yoriko Heianza,[2] Tao Huang,[2] Tiange Wang,[2] Dianjianyi Sun,[2] Yan Zheng,[3] Frank B Hu,[1,3,4] Kathryn M Rexrode,[5] JoAnn E Manson[1,4,5] and Lu Qi[1,2,3,4]*

[1]Department of Epidemiology, Harvard T.H. Chan School of Public Health, Boston, MA, USA, [2]Department of Epidemiology, School of Public Health and Tropical Medicine, Tulane University, New Orleans, LA, USA, [3]Department of Nutrition, Harvard T.H. Chan School of Public Health, Boston, MA, USA, [4]Channing Division of Network Medicine and [5]Division of Preventive Medicine, Department of Medicine, Brigham and Women's Hospital and Harvard Medical School, Boston, MA, USA

*Corresponding author. Department of Epidemiology, School of Public Health and Tropical Medicine, Tulane University, New Orleans, LA 70112, USA. E-mail: lqi1@tulane.edu

Editorial decision 12 October 2017; Accepted 17 October 2017

Abstract

Background: Emerging studies have related circulating glutamine metabolites to various chronic diseases such as cardiovascular disease and cancer; diet is the major source of nutrients involved in glutamine metabolism. However, it remains unknown whether dietary intakes of glutamine, glutamate,and their ratio are related to total and cause-specific mortality.
Methods: We followed 74 082 women from the Nurses' Health Study (1984–2012) and 42 303 men from the Health Professionals Follow-up Study (1986–2012), who were free of cardiovascular disease and cancer at baseline. Diet was updated every 2 to 4 years by using validated food frequency questionnaires. The content of glutamine and glutamate in foods was calculated based on protein fractions generated from gene sequencing methods and adjusted for total energy intake.
Results: We documented 30 424 deaths during 2 878 344 person-years of follow-up. After adjustment for potential confounders including lifestyle and dietary factors, higher intakes of glutamine and glutamine-to-glutamate ratio were associated with significantly lower risk of total and cause-specific mortality. Compared with people in the lowest quintile of dietary glutamine-to-glutamate ratio, the pooled hazard ratio (HR) in the highest quintile was 0.87 [95% confidence interval (CI): 0.84, 0.91; P for trend < 0.001) for total mortality, 0.81 (95% CI: 0.75, 0.88; P for trend < 0.001) for cardiovascular mortality, and 0.93 (95% CI: 0.87, 0.99; P for trend $= 0.01$) for cancer mortality.
Conclusions: We found dietary glutamine and glutamine-to-glutamate ratio were inversely related to risk of mortality, particularly cardiovascular mortality, independent of other dietary and lifestyle factors, in US men and women.

Key words: Glutamine, diet, mortality, epidemiology

© The Author 2017; all rights reserved. Published by Oxford University Press on behalf of the International Epidemiological Association

Key Messages

- In two large, prospective cohorts of US men and women, long-term dietary intakes of glutamine and glutamine-to-glutamate ratio were consistently associated with a decrease in the risk of total mortality and cause-specific mortality.
- The associations appeared to be independent of traditional risk factors and other dietary factors.
- Our research highlights the important roles of glutamine in primary prevention of premature mortality.

Background

Glutamine and its intermediate product, glutamate, are the most abundant amino acids in the human body.[1] Metabolism of glutamine and glutamate plays key roles in maintenance and promotion of a variety of physiological functions, such as synthesis of muscle proteins,[2] activation of immune system[3] and protection of gastrointestinal mucosa.[4]

Emerging evidence has linked glutamine and glutamate to a variety of chronic disorders. Several clinical trials revealed the cardioprotective effects of glutamine supplementation in patients with coronary heart disease[5–7] or diabetes.[8] Our previous analysis indicated that genetic variation in glutamine metabolism was associated with coronary heart disease in individuals with diabetes, further suggesting a mechanistic link.[9] In addition to the potential role in cardiovascular health, glutamine metabolism is closely related to malignancy of tumour cells, and has been implicated in detection, monitoring and treatment of cancer.[10] Recent studies using metabolomics profiling have provided valuable tools to evaluate glutamine and its metabolites, and identified circulating levels of glutamine, glutamate and glutamine-to-glutamate ratio associated with multiple metabolic abnormalities such as obesity, hypertension, dyslipidaemia,[11–14] and risk of developing type 2 diabetes,[15] cardiovascular disease[16] and cancer.[17]

Diet is the major source of glutamine and glutamate, and may modulate circulating levels of their metabolites.[18] To the best of our knowledge, no previous studies have elucidated the associations of long-term intakes of glutamine and glutamate with risk of mortality. Therefore in the present study, we prospectively examined dietary intakes of glutamine, glutamate and their ratio in relation to total and cause-specific mortality in two large cohorts of US women and men, the Nurses' Health Study (NHS) and the Health Professionals Follow-up Study (HPFS).

Methods

Study population

The NHS is a prospective study of a cohort of 121 701 female registered nurses aged 30–55 years from 11 US states, who were enrolled in 1976. The HPFS is a prospective study of a cohort of 51 529 male health professionals aged 40–75 years from all 50 states, enrolled in 1986. Participants were followed with the use of biennial validated questionnaires concerning medical history, lifestyle and health practices. The follow-up rate has been greater than 90% for both cohorts. The study protocol was approved by the Institutional Review Boards at Harvard T.H. Chan School of Public Health and Brigham and Women's Hospital. Return of the questionnaires was considered to imply written informed consent.

For the current investigation, we excluded: participants with cancer or cardiovascular disease (coronary heart disease, stroke, angina and coronary artery bypass grafting) at baseline (1984 for the NHS and 1986 for the HPFS); those who had incomplete information for dietary data; and those who reported implausible total energy intake (<500 or >3500 kcal/day for the NHS and <800 or >4200 kcal/day for the HPFS). After exclusions, a total of 74 082 women and 42 303 men remained in the analysis.

Assessment of diet

Usual dietary habits were assessed by using validated semi-quantitative food frequency questionnaires (FFQs) every 2 to 4 years. The FFQs enquired about mean consumption of foods, beverages and supplements (with a prespecified portion size) during the previous year using nine categories of intake frequency, ranging from less than one per month to six or more per day. The reproducibility and validity of protein intake estimated by the FFQ against dietary records were reported previously, with approximate correlation coefficients between protein intake from FFQ and dietary records of 0.4.[19,20] All nutrient data were adjusted for total energy intake using the residual method.[21]

Assessment of dietary glutamine and glutamate

Dietary glutamine and glutamate values were derived using gene sequencing methods (Swiss Institute of Bioinformatics), which have been validated against US Department of Agriculture and modified biochemical (Khun) methods.[22]

Briefly, we calculated the proteinogenic amino acid content of food proteins consumed in the nutrient database based on: (i) the identification of protein fractions of food proteins reported in the literature; (ii) the identification of the amino acid composition of protein fractions using the Expert Protein Analysis System (ExPASy) sequence database server; (iii) the weighted sum of amino acids content of protein fractions in food proteins; and (iv) the incorporation of amino acid content of food proteins in recipes used to create the nutrient database. The amino acid content of food protein fractions was calculated using the entire sequence of the protein fraction examined. We excluded the terminal residues from the amino acid calculations as terminal amino acids have a short lifetime, even though terminal amino acids have little impact on the final composition of proteins, given their small contribution of the total sequence. The formula to calculate the content of amino acids in food protein derived from sequencing data was described in earlier studies.[23] As an example, the glutamine content of β-casein in milk was calculated as:

[% β-casein × # glutamine in sequence × molecular weight of glutamine (g/mole)]/molecular weight of β-casein (g/mole) = (23.4% × 20 × 146.15)/(23583.2) = 2.9%.

Ascertainment of mortality

Deaths were reported by the next of kin or the postal authorities or identified by searching the National Death Index. More than 97% of deaths can be identified in these cohorts.[24] For all deaths, we sought death certificates and requested permission from the next of kin to review medical records when appropriate. The underlying cause of death was assigned according to the *International Classification of Diseases, 8th Revision (ICD-8)*. In this analysis we also specifically considered deaths due to cardiovascular disease (*ICD-8* codes 390.0–458.9 or 795.0–795.9) or cancer (*ICD-8* codes 140.0–207.9).

Assessment of covariates

Data on demographics, medical history and lifestyle habits have been collected by self-administered questionnaires since baseline and updated biennially. Hypertension and hypercholesterolaemia were self-reported, with the validity of these reports confirmed on random sampling of medical records.[25] A supplementary questionnaire was used to confirm self-reported cases of diabetes according to established criteria,[26] and 98% of these cases were validated in comparison with medical records. Information on smoking status, menopausal status and postmenopausal hormone use (women only), family history of myocardial infarction and cancer, multivitamin and aspirin use, height and body weight was obtained. Self-reported weight was validated against technician-measured weight, with a Spearman correlation of 0.97 and mean difference in self-reported and measured weight of −1.50 kg for the NHS participants and −1.06 kg for the HPFS participants.[27] Body mass index (BMI) was calculated as weight in kilograms divided by height squared in metres. Physical activity was assessed every 2–4 years using validated questionnaires.[28]

Statistical analysis

Individuals contributed person-time from the return date of the first FFQ until the date of death or 30 June 2012 for the NHS, or 31 January 2012 for the HPFS, whichever came first. To best represent long-term intake and to minimise within-person variation, we created and used the cumulative average of glutamine, glutamate and glutamine-to-glutamate ratio intakes from all available dietary questionnaires for an individual up to the start of each follow-up interval.[29] We replaced missing values in each FFQ with cumulative average based on previous assessments.

Participants were divided into quintiles according to their energy-adjusted intakes of glutamine, glutamate and glutamine-to-glutamate ratio. Multivariable-adjusted Cox proportional hazards models stratified on age (years) and calendar time (2-year intervals) were used to estimate hazard ratios (HRs) and 95% confidence intervals (CIs) for the associations of nutrient intakes with total and cause-specific mortality. Linear trend was assessed by assigning the median value for the sample to each quintile and modelling this as a continuous variable. Proportional hazards assumption was tested by evaluating the significance of the interaction term between quintiles of nutrient intake and age, and no violation of the proportional hazards assumption was observed (P for interaction > 0.05).

In multivariate analysis, we adjusted for ethnicity and family history of heart disease and cancer, as well as time-varying covariates including age, menopausal status and postmenopausal hormone therapy use (women only), BMI, smoking status, alcohol intake, physical activity, multivitamin use, aspirin use, baseline history of hypertension, hypercholesterolaemia and diabetes, and dietary intakes of total energy, total fat, polyunsaturated fat and trans fat. In the sensitivity analyses, we further controlled for major dietary sources of glutamine and glutamate including intakes of red meat and whole grains; alternatively, we controlled for intakes of total protein, animal protein, plant protein or other types of amino acids that might be related to cardiometabolic risk including branched-chain amino acids and aromatic amino acids.

We also assessed potential effect modification by baseline characteristics including age (≥60 years or <60 years), BMI (≥25 kg/m^2 or <25 kg/m^2), smoking status (ever smoker versus never smoker), alcohol intake (drinkers versus non-drinkers) and levels of physical activity (median). We constructed cross-product terms between quintiles of nutrient intakes and these factors, with statistical significance of multiplicative interaction determined by using the Wald test.

To summarize the associations across the two cohorts, we conducted an inverse variance-weighted, fixed-effect meta-analysis, because no significant heterogeneity was found. Statistical analyses were conducted using SAS software version 9.3 (SAS Institute Inc). All *P*-values are two-sided and a *P*-value of <0.05 was considered as statistically significant.

Results

Table 1 presents baseline characteristics of participants by quintiles of glutamine-to-glutamate ratio. In both cohorts, participants with a higher intake of glutamine-to-glutamate ratio had lower BMI and alcohol intake, and were less likely to have a history of hypertension and diabetes. A higher glutamine-to-glutamate ratio was also related to a lower intake of protein, red meat, vegetables but a higher intake of whole grains and dietary fibre. In women, those with a higher glutamine-to-glutamate ratio were also less likely to be physically active and use multivitamins; whereas in men, those with a higher glutamine-to-glutamate ratio were more likely to be physically active and use multivitamins, but less likely to be current smokers and aspirin users. In our cohorts, the major food contributors to glutamine intake were grains (~32%), dairy products (~16%) and fish/poultry (~13%), and the major contributors to glutamate intake were fish/poultry (~28%), red meats (~24%) and dairy products (~21%) (Supplementary Table 1, available as Supplementary data at *IJE* online).

In the NHS, during 28 years of follow-up (1 913 956 person-years), we documented 17 433 deaths of which

Table 1. Baseline characteristics of participants in NHS and HPFS

	Quintiles of glutamine-to-glutamate ratio					
	NHS			HPFS		
	1	3	5	1	3	5
Glutamine-to-glutamate ratio	0.8 (0.1)	1.0 (0.0)	1.3 (0.2)	0.8 (0.1)	1.0 (0.0)	1.3 (0.2)
Glutamine, g/day	6.2 (1.1)	6.4 (1.0)	6.7 (1.0)	7.5 (1.2)	8.0 (1.1)	8.7 (1.5)
Glutamate, g/day	8.0 (1.6)	6.5 (1.0)	5.3 (0.9)	9.6 (1.7)	8.0 (1.2)	6.6 (1.2)
Age, years[a]	50.7 (7.0)	50.0 (7.2)	50.0 (7.4)	54.1 (9.4)	52.8 (9.5)	52.5 (9.6)
BMI, kg/m^2	25.3 (4.8)	24.9 (4.7)	24.6 (4.6)	26.0 (3.5)	25.3 (3.1)	24.8 (3.1)
Alcohol, g/day	7.8 (12.2)	6.6 (10.6)	5.8 (10.6)	11.7 (15.5)	10.8 (14.2)	10.6 (15.7)
Physical activity, h/week	3.2 (2.2)	3.1 (2.1)	2.9 (2.1)	2.7 (4.3)	2.8 (4.0)	3.0 (4.5)
Past smoker	33.1	31.7	29.1	42.4	39.3	38.5
Current smoker	25.8	22.6	23.5	11.9	8.6	7.2
Multivitamin use, %	38.9	36.7	34.8	61.7	63.6	63.8
Aspirin use, %	70.6	72.2	70.2	27.3	26.8	25.4
Family history of myocardial infarction, %	26.1	24.8	24.3	32.1	31.7	31.6
Family history of cancer, %	13.7	14.5	14.9	33.7	35.0	34.3
History of hypertension, %	23.2	21.4	18.9	22.6	19.1	17.6
History of hypercholesterolaemia, %	8.4	7.5	7.7	11.0	10.7	10.7
History of diabetes, %	3.4	2.7	2.4	3.2	2.2	1.6
White, %	96.8	98.5	98.7	94.2	96.2	96.6
Total energy intake, kcal/day	1729.0 (536.0)	1764.9 (527.9)	1718.5 (533.0)	1930.3 (568.8)	1981.6 (563.4)	1936.6 (563.8)
Protein intake, g/day	79.9 (13.3)	69.0 (9.5)	60.4 (9.2)	102.7 (16.8)	89.7 (12.5)	79.2 (12.5)
Red meat intake, serving/d	1.3 (0.8)	1.1 (0.6)	0.9 (0.5)	1.3 (0.9)	1.1 (0.7)	0.8 (0.6)
Fruit intake, serving/day	1.5 (1.2)	1.4 (1.0)	1.2 (1.0)	1.6 (1.3)	1.6 (1.2)	1.6 (1.3)
Vegetable intake, serving/day	3.2 (1.8)	2.7 (1.5)	2.4 (1.3)	3.2 (1.9)	2.9 (1.6)	2.7 (1.7)
Whole grain intake, g/day	10.5 (10.0)	14.7 (12.6)	19.0 (17.6)	15.5 (14.7)	22.7 (17.6)	31.8 (27.2)
Dietary fiber intake, g/day	15.9 (5.0)	16.4 (4.7)	16.9 (5.1)	19.6 (6.7)	21.1 (6.7)	23.1 (8.1)
Alternate Healthy Eating Index score	44.2 (10.5)	41.8 (10.0)	41.1 (10.0)	47.2 (10.8)	46.5 (10.7)	46.4 (11.1)

Values are means(SD) or percentages and are standardized to the age distribution of the study population.
[a]Value is not age-adjusted.

Table 2. Total mortality and cause-specific mortality according to quintiles of dietary glutamine intake in the meta-analysis of results from the NHS and HPFS

	Quintiles of dietary glutamine intake					
	1	2	3	4	5	*P* for trend
Total mortality						
Model 1[a]	1 (reference)	0.85 (0.82, 0.88)	0.78 (0.76, 0.81)	0.78 (0.75, 0.81)	0.77 (0.74, 0.79)	<0.001
Model 2[b]	1 (reference)	0.96 (0.92, 0.99)	0.92 (0.89, 0.95)	0.91 (0.88, 0.94)	0.85 (0.82, 0.89)	<0.001
Cardiovascular mortality						
Model 1[a]	1 (reference)	0.89 (0.83, 0.95)	0.80 (0.74, 0.86)	0.83 (0.77, 0.89)	0.87 (0.81, 0.93)	<0.001
Model 2[b]	1 (reference)	0.96 (0.90, 1.03)	0.88 (0.82, 0.95)	0.89 (0.83, 0.96)	0.87 (0.80, 0.94)	<0.001
Cancer mortality						
Model 1[a]	1 (reference)	0.83 (0.78, 0.88)	0.80 (0.75, 0.85)	0.78 (0.73, 0.82)	0.76 (0.71, 0.80)	<0.001
Model 2[b]	1 (reference)	0.94 (0.89, 1.00)	0.94 (0.89, 1.00)	0.93 (0.87, 0.98)	0.89 (0.83, 0.95)	<0.001

[a]Adjusted for age (years).

[b]Further adjusted for ethnicity (Caucasian, yes/no), menopausal status [pre- or postmenopausal (never, past or current menopausal hormone use), women only], body mass index (kg/m^2: <21, 21–24.9, 25–29.9, 30–31.9 or ≥32), smoking status (never smoker, past smoker, current smoker: 1–14, 15–24, or ≥25 cigarettes/day), physical activity (h/week: 0, 0.01–1.0, 1.0–3.5, 3.6–6.0 or ≥6), family history of myocardial infarction or cancer (yes/no), hypertension (yes/no), hypercholesterolaemia (yes/no), diabetes (yes/no), multivitamin use (yes/no), aspirin (yes/no), alcohol intake (g/day: 0, 0.1–4.9, 5.0–14.9, 15.0–19.9, 20.0–29.9 or ≥30), and dietary intakes (all in quintiles) of total energy, total fat, polyunsaturated fat and trans fat.

3621 were cardiovascular deaths and 6508 were cancer deaths. In the HPFS, during 26 years of follow-up (964 388 person-years), we documented 12 991 deaths of which 3971 were cardiovascular deaths and 4154 were cancer deaths. The associations of dietary glutamine intake with total and cause-specific mortality in the meta-analysis of results from the NHS and HPFS are shown in Table 2 (results from separate cohort are shown in Supplementary Table 2, available as Supplementary data at *IJE* online). In age-adjusted analyses, a higher intake of glutamine was significantly associated with lower risk of total and cause-specific mortality. After adjustment for a list of potential confounders including demographics, lifestyle, and dietary factors, the associations with cardiovascular mortality did not appreciably change, whereas the associations with total and cancer mortality were attenuated but remained significant; the largest changes in these associations were seen with adjustment for physical activity, smoking status and multivitamin use. The pooled HRs comparing extreme quintiles of glutamine intake were 0.85 (95% CI: 0.82, 0.89; *P* for trend < 0.001) for total mortality, 0.87 (95% CI: 0.80, 0.94; *P* for trend < 0.001) for cardiovascular mortality, and 0.89 (95% CI: 0.83, 0.95; *P* for trend < 0.001) for cancer mortality.

Table 3 shows the pooled associations of dietary intake of glutamate with risk of mortality (results from separate cohort are presented in Supplementary Table 3, available as Supplementary data at *IJE* online). In the combined cohorts, a higher glutamate intake was associated with increased risk of total and cardiovascular mortality in age-adjusted models. The associations were attenuated with multivariate adjustment, becoming statistically insignificant for total mortality (pooled extreme quintile HR: 1.03; 95% CI: 0.99, 1.07; *P* for trend = 0.10) and marginal significant for cardiovascular mortality (pooled extreme quintile HR: 1.09; 95% CI: 1.00, 1.17; *P* for trend = 0.03). Glutamate intake was not significantly associated with cancer mortality.

A higher intake of glutamine-to-glutamate ratio was also significantly associated with lower risk of total and cause-specific mortality (Table 4; Supplementary Table 4, available as Supplementary data at *IJE* online). Although further adjustment for potential confounders attenuated these associations, the statistical significance remained. The multivariable-adjusted HRs comparing extreme quintiles of glutamine-to-glutamate ratio were 0.87 (95% CI: 0.84, 0.91; *P* for trend < 0.001) for total mortality, 0.81 (95% CI: 0.75, 0.88; *P* for trend < 0.001) for cardiovascular mortality and 0.93 (95% CI: 0.87, 0.99; *P* for trend = 0.01) for cancer mortality.

In sensitivity analyses, further adjustment for dietary intakes of red meat and whole grains attenuated the associations of glutamine intake with total and cause-specific mortality, although the inverse relationship remained in the meta-analysis of results from the two cohorts (Supplementary Table 5, available as Supplementary data at *IJE* online). With regard to glutamine-to-glutamate ratio, the associations with total mortality (extreme quintile HR: 0.91; 95% CI: 0.88, 0.95; *P* for trend < 0.001) and cardiovascular mortality (extreme quintile HR: 0.85; 95% CI: 0.78, 0.92; *P* for trend < 0.001) were attenuated but remained significant, whereas the association with cancer mortality (extreme quintile HR: 0.96; 95% CI: 0.89, 1.03; *P* for trend = 0.14) became not significant. When

Table 3. Total mortality and cause-specific mortality according to quintiles of dietary glutamate intake in the meta-analysis of results from the NHS and HPFS

	Quintiles of dietary glutamate intake					
	1	2	3	4	5	*P* for trend
Total mortality						
Model 1[a]	1 (reference)	0.93 (0.90, 0.96)	0.93 (0.90, 0.96)	0.96 (0.93, 1.00)	1.09 (1.05, 1.13)	<0.001
Model 2[b]	1 (reference)	1.01 (0.97, 1.04)	1.00 (0.97, 1.04)	1.02 (0.99, 1.06)	1.03 (0.99, 1.07)	0.10
Cardiovascular mortality						
Model 1[a]	1 (reference)	0.98 (0.92, 1.05)	0.97 (0.90, 1.04)	1.06 (0.99, 1.14)	1.28 (1.19, 1.37)	<0.001
Model 2[b]	1 (reference)	1.03 (0.96, 1.10)	1.00 (0.93, 1.07)	1.05 (0.98, 1.13)	1.09 (1.00, 1.17)	0.03
Cancer mortality						
Model 1[a]	1 (reference)	0.93 (0.88, 0.99)	0.93 (0.88, 0.99)	0.95 (0.89, 1.01)	0.99 (0.93, 1.06)	0.82
Model 2[b]	1 (reference)	1.00 (0.95, 1.06)	1.01 (0.95, 1.07)	1.02 (0.96, 1.08)	0.99 (0.93, 1.06)	0.90

[a]Adjusted for age (years).

[b]Further adjusted for ethnicity (Caucasian, yes/no), menopausal status [pre- or postmenopausal (never, past or current menopausal hormone use), women only], body mass index (kg/m^2: <21, 21–24.9, 25–29.9, 30–31.9 or ≥32), smoking status (never smoker, past smoker, current smoker: 1–14, 15–24, or ≥25 cigarettes/day), physical activity (h/week: 0, 0.01–1.0, 1.0–3.5, 3.6–6.0 or ≥6), family history of myocardial infarction or cancer (yes/no), hypertension (yes/no), hypercholesterolaemia (yes/no), diabetes (yes/no), multivitamin use (yes/no), aspirin (yes/no), alcohol intake (g/day: 0, 0.1–4.9, 5.0–14.9, 15.0–19.9, 20.0–29.9 or ≥30), and dietary intakes (all in quintiles) of total energy, total fat, polyunsaturated fat and trans fat.

Table 4. Total mortality and cause-specific mortality according to quintiles of glutamine-to-glutamate ratio in the meta-analysis of results from the NHS and HPFS

	Quintiles of glutamine-to-glutamate ratio					
	1	2	3	4	5	*P* for trend
Total mortality						
Model 1[a]	1 (reference)	0.81 (0.78, 0.84)	0.75 (0.72, 0.78)	0.72 (0.69, 0.75)	0.70 (0.68, 0.73)	<0.001
Model 2[b]	1 (reference)	0.96 (0.92, 1.00)	0.94 (0.91, 0.98)	0.91 (0.88, 0.95)	0.87 (0.84, 0.91)	<0.001
Cardiovascular mortality						
Model 1[a]	1 (reference)	0.79 (0.73, 0.84)	0.71 (0.66, 0.76)	0.66 (0.61, 0.71)	0.66 (0.61, 0.71)	<0.001
Model 2[b]	1 (reference)	0.91 (0.85, 0.98)	0.88 (0.82, 0.95)	0.83 (0.77, 0.90)	0.81 (0.75, 0.88)	<0.001
Cancer mortality						
Model 1[a]	1 (reference)	0.89 (0.83, 0.94)	0.85 (0.80, 0.90)	0.83 (0.78, 0.88)	0.78 (0.73, 0.83)	<0.001
Model 2[b]	1 (reference)	1.01 (0.95, 1.08)	1.01 (0.95, 1.08)	1.00 (0.94, 1.07)	0.93 (0.87, 0.99)	0.01

[a]Adjusted for age (years).

[b]Further adjusted for ethnicity (Caucasian, yes/no), menopausal status [pre- or postmenopausal (never, past or current menopausal hormone use), women only], body mass index (kg/m^2: <21, 21–24.9, 25–29.9, 30–31.9 or ≥32), smoking status (never smoker, past smoker, current smoker: 1–14, 15–24, or ≥25 cigarettes/day), physical activity (h/week: 0, 0.01–1.0, 1.0–3.5, 3.6–6.0 or ≥6), family history of myocardial infarction or cancer (yes/no), hypertension (yes/no), hypercholesterolaemia (yes/no), diabetes (yes/no), multivitamin use (yes/no), aspirin (yes/no), alcohol intake (g/day: 0, 0.1–4.9, 5.0–14.9, 15.0–19.9, 20.0–29.9, or ≥30), and dietary intakes (all in quintiles) of total energy, total fat, polyunsaturated fat and trans fat.

intakes of total protein, branched-chain amino acids, or aromatic amino acids were further controlled for in the model, the associations of glutamine and glutamine-to-glutamate ratio with total and cardiovascular mortality became even stronger; however, a slight attenuation in the associations was observed with adjustment for plant protein, but not animal protein (data not shown).

In stratified analyses of glutamine-to-glutamate ratio and cardiovascular mortality by baseline characteristics including age, BMI, smoking status, alcohol intake and physical activity (Table 5), a significant interaction was observed of glutamine-to-glutamate ratio with BMI in the NHS (*P* for interaction = 0.03). The inverse, linear association between glutamine-to-glutamate ratio and cardiovascular mortality was more evident among women who were overweight or obese (extreme quintile HR: 0.80; 95% CI: 0.68, 0.93). In the HPFS, no significant interactions between glutamine-to-glutamate ratio and the above baseline characteristics were observed, and the associations largely persisted among participants with various risk profiles. For total mortality (data not shown), we observed a significant interaction between glutamine-to-glutamate ratio and physical activity in the NHS (*P* for interaction = 0.008). A stronger inverse association was seen among more

Table 5. Cardiovascular mortality according to quintiles of glutamine-to-glutamate ratio by various characteristics of participants

	Quintiles of glutamine-to-glutamate ratio					
Characteristics	1	2	3	4	5	*P* for interaction
NHS						
Age						0.18
Age < 60 years	1 (reference)	1.00 (0.71, 1.41)	0.68 (0.45, 1.03)	0.94 (0.64, 1.39)	0.63 (0.40, 1.00)	
Age ≥ 60 years	1 (reference)	0.90 (0.80, 1.01)	0.94 (0.83, 1.05)	0.86 (0.77, 0.97)	0.86 (0.77, 0.97)	
Body mass index						0.03
BMI < 25 kg/m^2	1 (reference)	0.91 (0.77, 1.08)	0.95 (0.80, 1.12)	0.92 (0.78, 1.09)	0.91 (0.77, 1.08)	
BMI ≥ 2 5 kg/m^2	1 (reference)	0.91 (0.79, 1.05)	0.90 (0.77, 1.04)	0.82 (0.70, 0.96)	0.80 (0.68, 0.93)	
Smoking status						0.07
Never	1 (reference)	0.80 (0.66, 0.97)	0.78 (0.64, 0.94)	0.81 (0.67, 0.98)	0.82 (0.68, 0.99)	
Ever	1 (reference)	0.95 (0.83, 1.09)	0.99 (0.86, 1.14)	0.88 (0.76, 1.01)	0.83 (0.72, 0.96)	
Physical activity						0.13
Below median	1 (reference)	0.91 (0.80, 1.04)	0.91 (0.80, 1.04)	0.89 (0.78, 1.02)	0.88 (0.78, 1.01)	
Above median	1 (reference)	0.92 (0.74, 1.14)	0.95 (0.76, 1.18)	0.81 (0.64, 1.02)	0.77 (0.60, 0.97)	
Alcohol intake						0.77
Non-drinker	1 (reference)	0.88 (0.76, 1.02)	0.91 (0.78, 1.05)	0.89 (0.77, 1.04)	0.84 (0.72, 0.97)	
Drinker	1 (reference)	0.94 (0.79, 1.11)	0.93 (0.78, 1.10)	0.81 (0.68, 0.97)	0.87 (0.73, 1.04)	
HPFS						
Age						0.11
Age < 60 years	1 (reference)	0.81 (0.52, 1.26)	0.64 (0.39, 1.05)	0.64 (0.39, 1.08)	0.64 (0.38, 1.10)	
Age ≥ 60 years	1 (reference)	0.93 (0.84, 1.03)	0.87 (0.78, 0.96)	0.82 (0.74, 0.92)	0.80 (0.71, 0.89)	
Body mass index						0.86
BMI < 25 kg/m^2	1 (reference)	0.97 (0.84, 1.13)	0.94 (0.81, 1.09)	0.86 (0.74, 1.00)	0.81 (0.69, 0.95)	
BMI ≥ 25 kg/m^2	1 (reference)	0.90 (0.79, 1.03)	0.79 (0.68, 0.91)	0.78 (0.68, 0.91)	0.79 (0.68, 0.93)	
Smoking status						0.48
Never	1 (reference)	0.83 (0.62, 1.10)	0.90 (0.68, 1.19)	0.83 (0.62, 1.11)	0.71 (0.52, 0.96)	
Ever	1 (reference)	0.91 (0.77, 1.09)	0.87 (0.73, 1.04)	0.79 (0.66, 0.95)	0.77 (0.63, 0.95)	
Physical activity						0.08
Below median	1 (reference)	0.97 (0.86, 1.08)	0.90 (0.80, 1.02)	0.84 (0.74, 0.95)	0.87 (0.76, 0.99)	
Above median	1 (reference)	0.82 (0.68, 1.00)	0.75 (0.62, 0.92)	0.74 (0.61, 0.91)	0.61 (0.49, 0.77)	
Alcohol intake						0.76
Non-drinker	1 (reference)	0.98 (0.82, 1.17)	0.88 (0.74, 1.06)	0.87 (0.72, 1.04)	0.79 (0.65, 0.96)	
Drinker	1 (reference)	0.91 (0.80, 1.02)	0.86 (0.76, 0.97)	0.80 (0.71, 0.91)	0.80 (0.70, 0.92)	

Models were adjusted for ethnicity (Caucasian, yes/no), menopausal status [pre- or postmenopausal (never, past or current menopausal hormone use), women only], body mass index (kg/m^2: < 21, 21–24.9, 25–29.9, 30–31.9 or ≥32), smoking status (never smoker, past smoker, current smoker: 1–14, 15–24, or ≥ 25 cigarettes/day), physical activity (h/week: 0, 0.01–1.0, 1.0–3.5, 3.6–6.0 or ≥6), family history of myocardial infarction or cancer (yes/no), hypertension (yes/no), hypercholesterolaemia (yes/no), diabetes (yes/no), multivitamin use (yes/no), aspirin (yes/no), alcohol intake (g/day: 0, 0.1–4.9, 5.0–14.9, 15.0–19.9, 20.0–29.9, or ≥ 30), and dietary intakes (all in quintiles) of total energy, total fat, polyunsaturated fat and trans fat.

physically active women (extreme quintile HR: 0.82; 95% CI: 0.74, 0.91) than the rest of participants (extreme quintile HR: 0.93; 95% CI: 0.87, 0.99). None of the characteristics significantly interacted with glutamine-to-glutamate ratio in relation to total mortality in the HPFS.

Discussion

In two independent, large prospective cohorts of US men and women, we found consistent associations of long-term dietary intake of glutamine and glutamine-to-glutamate ratio with a decrease in the risk of total mortality and cause-specific mortality. The associations appeared to be independent of traditional risk factors and other dietary factors.

In support of our findings, metabolomics profiling has identified circulating glutamine, glutamate and glutamine-to-glutamate ratio to be associated with multiple metabolic risk factors in cross-sectional studies.[11–14] Specifically, glutamine and glutamine-to-glutamate ratio were inversely associated with BMI, insulin resistance, blood pressure and triglycerides and positively associated with high-density lipoprotein (HDL) cholesterol, whereas glutamate was associated with adverse metabolic profiles. In a recent

prospective study, plasma level of glutamate was associated with increased risk of incident cardiovascular disease, whereas glutamine-to-glutamate ratio was associated with decreased risk.[16] Decreased glutamine levels were also seen in patients with pancreatic[30] or colorectal cancer.[31] There is however a paucity of data evaluating habitual dietary intakes of glutamine and glutamate or major sources of the two nutrients in relation to total and cause-specific mortality, owing to the historical lack of accurate measurement and absence from current nutrient databases.[22] Our findings expand on an emerging appreciation of a role for altered glutamine metabolism in long-term health outcomes, and highlight the need to consider dietary intakes of glutamine and glutamine-to-glutamate ratio in future investigations.

The biological pathways through which dietary consumption of glutamine may affect long-term health are not entirely clear. As the most abundant amino acid in plasma, glutamine is involved in a variety of physiological functions, such as synthesis of proteins and stimulation of immune responses.[1–3] *In vitro* and animal studies have documented the pleiotropic effects of glutamine, such as protecting the heart from ischaemic/reperfusion injury,[32–36] increasing glucose tolerance and decreasing blood pressure[11] as well as attenuating inflammatory responses.[37–39] Small clinical trials further confirmed that glutamine supplementation could enhance myocardial repair and improve cardiovascular risk factors in patients with coronary heart disease[5–7] or type 2 diabetes.[8] However, chronic glutamine supplementation has also been associated with adverse events such as increase in ureagenesis, alterations in amino acid transport and distribution, decrease in endogenous synthesis of glutamine and enhancement in glutamate and ammonia production;[40] in a recent randomized controlled trial, glutamine supplementation was associated with an increase in mortality among critically ill patients with multiorgan failure.[41] Careful consideration should be given to these issues before recommending glutamine supplementation or a high glutamine diet to people with multiple organ failure, in particular those with renal insufficiency and failure.

In our cohorts, a considerable amount of glutamine comes from plant-based protein sources such as refined grains, whole grains and cold cereals, whereas glutamate intake was mostly contributed by animal protein sources including red meats, poultry, fish and dairy products. Different protein sources have been linked to long-term risk of mortality. For example, intake of red and processed meat has been associated with increased risk of total, cardiovascular and cancer mortality.[42] In contrast, higher whole grain consumption has been inversely related to total and cardiovascular mortality.[43] Consistent with these data, current findings from prospective cohorts suggest that a substitution of plant protein for animal protein may be associated with lower risk of mortality, particularly cardiovascular mortality.[44,45] In our analysis, additional adjustment for intake of red meat and whole grains or plant protein slightly attenuated the associations, suggesting glutamine and glutamate may partly account for the divergent health effects of varied protein sources.

The current study has several strengths. For the first time, we associated dietary intakes of glutamine and glutamine-to-glutamate ratio with risk of mortality in prospective cohorts; and the fairly consistent findings across the two cohorts demonstrated robustness of the results. The use of well-established cohorts with high follow-up rates and well-validated assessments of cases minimized selection and ascertainment biases. The prospective nature of the study design and long-term follow-up reduced the potential for reverse causation. A large sample size provided the statistical power to detect relevant associations. The comprehensive assessments of demographics, lifestyle habits and diet minimized the residual confounding.

Several potential limitations should be considered. First, although we adjusted for multiple important risk factors for mortality, it was difficult to rule out residual confounding in observational studies because of unmeasured or imprecisely measured confounders. Second, measurement errors were inevitable in the estimates of food and nutrient intakes, but the use of repeated measurements and cumulative average intakes reduced the magnitude of measurement error to some extent. In addition, random errors in exposure assessments generally attenuate true associations towards the null. Finally, our cohort included mostly Caucasian health professionals, and the results may not be generalizable to other ethnic groups. However, the relative homogeneity of the study populations in socioeconomic status and educational attainment enhanced the internal validity.

In conclusion, our data from two independent cohorts consistently showed that dietary intakes of glutamine and glutamine-to-glutamate ratio were significantly related to reduced risk of total mortality, including cardiovascular and cancer mortality. Future investigations are warranted to verify our findings in other populations and elucidate the roles of glutamine in primary and secondary prevention of chronic diseases and premature mortality.

Supplementary Data

Supplementary data are available at *IJE* online.

Funding

The cohorts were supported by grants UM1 CA186107, P01 CA87969, R01 HL034594, R01 HL088521, UM1 CA167552 and

R01 HL35464 from the National Institutes of Health. The current study was supported by grants from the National Heart, Lung, and Blood Institute (HL071981, HL034594, HL126024), the National Institute of Diabetes and Digestive and Kidney Diseases (DK091718, DK100383, DK078616), the Boston Obesity Nutrition Research Center (DK46200) and United States – Israel Binational Science Foundation (Grant 2011036). L.Q. was a recipient of the American Heart Association Scientist Development Award (0730094 N).

Acknowledgements

We would like to thank the participants and staff of the Nurses' Health Study and the Health Professionals Follow-up Study for their valuable contributions, as well as those in the state cancer registries for their help. The authors assume full responsibility for analyses and interpretation of these data.

Author Contributions

L.Q. has full access to all of the data in the study and takes responsibility for the integrity of the data and the accuracy of the data analysis. Study concept and design: W.M., L.Q.. Statistical analysis: W.M. Drafting of the manuscript: W.M., L.Q. Statistical advice, interpretation of data, critical revision of the manuscript for important intellectual content and approval of the final manuscript for submission: all authors.

Conflict of interest: None.

References

1. Newsholme P, Procopio J, Lima MM, Pithon-Curi TC, Curi R. Glutamine and glutamate - their central role in cell metabolism and function. *Cell Biochem Funct* 2003;**21**:1–9.
2. MacLennan PA, Brown RA, Rennie MJ. A positive relationship between protein synthetic rate and intracellular glutamine concentration in perfused rat skeletal muscle. *FEBS Lett* 1987;**215**: 187–91.
3. Newsholme P. Why is L-glutamine metabolism important to cells of the immune system in health, postinjury, surgery or infection? *J Nutr* 2001;**131**:2515–22S; discussion 2523–24S.
4. Ziegler TR, Bazargan N, Leader LM, Martindale RG. Glutamine and the gastrointestinal tract. *Curr Opin Clin Nutr Metab Care* 2000;**3**:355–62.
5. Khogali SE, Pringle SD, Weryk BV, Rennie MJ. Is glutamine beneficial in ischemic heart disease? *Nutrition* 2002;**18**:123–26.
6. Lomivorotov VV, Efremov SM, Shmirev VA, Ponomarev DN, Lomivorotov VN, Karaskov AM. Glutamine is cardioprotective in patients with ischemic heart disease following cardiopulmonary bypass. *Heart Surg Forum* 2011;**14**:E384–88.
7. Sufit A, Weitzel LB, Hamiel C *et al*. Pharmacologically dosed oral glutamine reduces myocardial injury in patients undergoing cardiac surgery: a randomized pilot feasibility trial. *J Parenter Enteral Nutr* 2012;**36**:556–61.
8. Mansour A, Mohajeri-Tehrani MR, Qorbani M, Heshmat R, Larijani B, Hosseini S. Effect of glutamine supplementation on cardiovascular risk factors in patients with type 2 diabetes. *Nutrition* 2015;**31**:119–26.
9. Qi L, Qi Q, Prudente S *et al*. Association between a genetic variant related to glutamic acid metabolism and coronary heart disease in individuals with type 2 diabetes. *JAMA* 2013;**310**: 821–28.
10. Hensley CT, Wasti AT, DeBerardinis RJ. Glutamine and cancer: cell biology, physiology, and clinical opportunities. *J Clin Invest* 2013;**123**:3678–84.
11. Cheng S, Rhee EP, Larson MG *et al*. Metabolite profiling identifies pathways associated with metabolic risk in humans. *Circulation* 2012;**125**:2222–31.
12. Wurtz P, Makinen VP, Soininen P *et al*. Metabolic signatures of insulin resistance in 7, 098 young adults. *Diabetes* 2012;**61**: 1372–80.
13. Newgard CB, An J, Bain JR *et al*. A branched-chain amino acid-related metabolic signature that differentiates obese and lean humans and contributes to insulin resistance. *Cell Metab* 2009; **9**:311–26.
14. Tai ES, Tan ML, Stevens RD *et al*. Insulin resistance is associated with a metabolic profile of altered protein metabolism in Chinese and Asian-Indian men. *Diabetologia* 2010;**53**: 757–67.
15. Guasch-Ferre M, Hruby A, Toledo E *et al*. Metabolomics in prediabetes and diabetes: a systematic review and meta-analysis. *Diabetes Care* 2016;**39**:833–46.
16. Zheng Y, Hu FB, Ruiz-Canela M *et al*. Metabolites of glutamate metabolism are associated with incident cardiovascular events in the PREDIMED PREvencion con DIeta MEDiterranea (PREDIMED) Trial. *J Am Heart Assoc* 2016;**5**. doi: 10.1161/JAHA.116.003755.
17. Armitage EG, Barbas C. Metabolomics in cancer biomarker discovery: current trends and future perspectives. *J Pharm Biomed Anal* 2014;**87**:1–11.
18. Matthews DE, Campbell RG. The effect of dietary protein intake on glutamine and glutamate nitrogen metabolism in humans. *Am J Clin Nutr* 1992;**55**:963–70.
19. Willett WC, Sampson L, Stampfer MJ *et al*. Reproducibility and validity of a semiquantitative food frequency questionnaire. *Am J Epidemiol* 1985;**122**:51–65.
20. Rimm EB, Giovannucci EL, Stampfer MJ, Colditz GA, Litin LB, Willett WC. Reproducibility and validity of an expanded self-administered semiquantitative food frequency questionnaire among male health professionals. *Am J Epidemiol* 1992;**135**: 1114–26; discussion 27–36.
21. Willett W, Stampfer MJ. Total energy intake:implications for epidemiologic analyses. *Am J Epidemiol* 1986;**124**:17–27.
22. Lenders CM, Liu S, Wilmore DW *et al*. Evaluation of a novel food composition database that includes glutamine and other amino acids derived from gene sequencing data. *Eur J Clin Nutr* 2009;**63**:1433–39.
23. Lacey JM, Wilmore DW. Is glutamine a conditionally essential amino acid? *Nutr Rev* 1990;**48**:297–309.
24. Rich-Edwards JW, Corsano KA, Stampfer MJ. Test of the National Death Index and Equifax Nationwide Death Search. *Am J Epidemiol* 1994;**140**:1016–19.
25. Colditz GA, Martin P, Stampfer MJ *et al*. Validation of questionnaire information on risk factors and disease outcomes in a prospective cohort study of women. *Am J Epidemiol* 1986;**123**: 894–900.

26. Classification and diagnosis of diabetes mellitus and other categories of glucose intolerance. National Diabetes Data Group. *Diabetes* 1979;**28**:1039–57.
27. Rimm EB, Stampfer MJ, Colditz GA, Chute CG, Litin LB, Willett WC. Validity of self-reported waist and hip circumferences in men and women. *Epidemiology* 1990;**1**:466–73.
28. Wolf AM, Hunter DJ, Colditz GA *et al*. Reproducibility and validity of a self-administered physical activity questionnaire. *Int J Epidemiol* 1994;**23**:991–99.
29. Hu FB, Stampfer MJ, Rimm E *et al*. Dietary fat and coronary heart disease: a comparison of approaches for adjusting for total energy intake and modeling repeated dietary measurements. *Am J Epidemiol* 1999;**149**:531–40.
30. Urayama S, Zou W, Brooks K, Tolstikov V. Comprehensive mass spectrometry based metabolic profiling of blood plasma reveals potent discriminatory classifiers of pancreatic cancer. *Rapid Commun Mass Spectrom* 2010;**24**:613–20.
31. Tan B, Qiu Y, Zou X *et al*. Metabonomics identifies serum metabolite markers of colorectal cancer. *J Proteome Res* 2013;**12**: 3000–09.
32. Almashhadany A, Alghamdi OA, Van der Touw T, Jones GL, King N. L-Glycyl-L-glutamine provides the isolated and perfused young and middle-aged rat heart protection against ischaemia-reperfusion injury. *Amino Acids* 2015;**47**:1559–65.
33. Khogali SE, Harper AA, Lyall JA, Rennie MJ. Effects of L-glutamine on post-ischaemic cardiac function: protection and rescue. *J Mol Cell Cardiol* 1998;**30**:819–27.
34. Lauzier B, Vaillant F, Merlen C *et al*. Metabolic effects of glutamine on the heart: anaplerosis versus the hexosamine biosynthetic pathway. *J Mol Cell Cardiol* 2013;**55**:92–100.
35. Liu J, Marchase RB, Chatham JC. Glutamine-induced protection of isolated rat heart from ischemia/reperfusion injury is mediated via the hexosamine biosynthesis pathway and increased protein O-GlcNAc levels. *J Mol Cell Cardiol* 2007;**42**:177–85.
36. Wischmeyer PE, Vanden Hoek TL, Li C *et al*. Glutamine preserves cardiomyocyte viability and enhances recovery of contractile function after ischemia-reperfusion injury. *J Parenter Enteral Nutr* 2003;**27**:116–22.
37. Johnson AT, Kaufmann YC, Luo S, Todorova V, Klimberg VS. Effect of glutamine on glutathione, IGF-I, and TGF-beta 1. *J Surg Res* 2003;**111**:222–28.
38. Cruzat VF, Rogero MM, Tirapegui J. Effects of supplementation with free glutamine and the dipeptide alanyl-glutamine on parameters of muscle damage and inflammation in rats submitted to prolonged exercise. *Cell Biochem Funct* 2010;**28**:24–30.
39. Raizel R, Leite JS, Hypolito TM *et al*. Determination of the anti-inflammatory and cytoprotective effects of l-glutamine and l-alanine, or dipeptide, supplementation in rats submitted to resistance exercise. *Br J Nutr* 2016;**116**:470–79.
40. Holecek M. Side effects of long-term glutamine supplementation. *J Parenter Enteral Nutr* 2013;**37**:607–16.
41. Heyland D, Muscedere J, Wischmeyer PE *et al*. A randomized trial of glutamine and antioxidants in critically ill patients. *N Engl J Med* 2013;**368**:1489–97.
42. Pan A, Sun Q, Bernstein AM *et al*. Red meat consumption and mortality: results from 2 prospective cohort studies. *Arch Intern Med* 2012;**172**:555–63.
43. Zong G, Gao A, Hu FB, Sun Q. Whole grain intake and mortality from all causes, cardiovascular disease, and cancer: a meta-analysis of prospective cohort studies. *Circulation* 2016;**133**: 2370–80.
44. Kelemen LE, Kushi LH, Jacobs DR Jr, Cerhan JR. Associations of dietary protein with disease and mortality in a prospective study of postmenopausal women. *Am J Epidemiol* 2005;**161**: 239–49.
45. Song M, Fung TT, Hu FB *et al*. Association of animal and plant protein intake with all-cause and cause-specific mortality. *JAMA Intern Med* 2016;**176**:1453–63.
