# Supplementary material for: A systematic comprehensive longitudinal evaluation of dietary factors associated with acute myocardial infarction and fatal coronary heart disease
Source: Nat Commun. 2020 Nov 27;11:6074. doi: 10.1038/s41467-020-19888-2 (PMC7699643; doi:10.1038/s41467-020-19888-2)
Supplement: Supplementary file 4 — Supplementary Software [file 41467_2020_19888_MOESM4_ESM.zip › EWAS-NHS-master/KG-SI/Rest/Paik (2014).pdf]

NIH Public Access
Author Manuscript
Osteoporos Int. Author manuscript; available in PMC 2015 August 01.

Published in final edited form as:
Osteoporos Int. 2014 August ; 25(8): 2047–2056. doi:10.1007/s00198-014-2732-3.

Calcium Supplement Intake and Risk of Cardiovascular Disease in Women

Julie M. Paik, MD, MPH, MSc[1,2], **Gary C. Curhan, MD, ScD**[1,2,4], **Qi Sun, MD, ScD**[1,5], **Kathryn M. Rexrode, MD, MPH**[1,3], **JoAnn E. Manson, MD, DrPH**[1,3,4], **Eric B. Rimm, ScD**[1,4,5], and **Eric N. Taylor, MD, MSc**[1,6]

Julie M. Paik: jmpaik@partners.org; Gary C. Curhan: gcurhan@partners.org; Qi Sun: qisun@hsph.harvard.edu; Kathryn M. Rexrode: krexrode@partners.org; JoAnn E. Manson: jmanson@rics.bwh.harvard.edu; Eric B. Rimm: erimm@hsph.harvard.edu; Eric N. Taylor: entaylor@partners.org

[1]Channing Division of Network Medicine, Department of Medicine, Brigham and Women's Hospital and Harvard Medical School, Boston, MA

[2]Renal Division, Department of Medicine, Brigham and Women's Hospital and Harvard Medical School, Boston, MA

[3]Division of Preventive Medicine, Department of Medicine, Brigham and Women's Hospital and Harvard Medical School, Boston, MA

[4]Department of Epidemiology, Harvard School of Public Health, Boston, MA

[5]Department of Nutrition, Harvard School of Public Health, Boston, MA

[6]Division of Nephrology and Transplantation, Maine Medical Center, Portland, ME

Abstract

Background—Some recent reports suggest that calcium supplements may increase cardiovascular disease (CVD) risk.

Purpose—The objective was to examine the independent associations between calcium supplement use and risk of CVD.

[1]To whom correspondence should be addressed: Julie M. Paik, M.D., Channing Laboratory, 181 Longwood Avenue, Room 432, Boston, MA 02115. Telephone 617-525-2029; Fax 617-525-2008; jmpaik@partners.org.

Authors' Contributions: JP contributed to the study conception and design, analysis and interpretation of data, drafting of the article and critical revisions of the article, and approved the final version of the manuscript. GC contributed to the conception and design, acquisition of data, analysis and interpretation of data, critical revisions of the article, and approved the final version of the manuscript. QS contributed to the analysis, critical revisions of the article, and approved the final version of the manuscript. KR contributed to the acquisition of data, interpretation of data, critical revisions of the article, and approved the final version of the manuscript. JM contributed to the acquisition of data, interpretation of data, critical revisions of the article, and approved the final version of the manuscript. ER contributed to the acquisition of data, interpretation of data, critical revisions of the article, and approved the final version of the manuscript. ET contributed to the conception and design, analysis and interpretation of data, critical revisions of the article, funding, and approved the final version of the manuscript.

Disclosure of Potential Conflicts of Interest: Julie M. Paik, Gary C. Curhan, Qi Sun, Kathryn M. Rexrode, JoAnn E. Manson, Eric B. Rimm, and Eric N. Taylor declare that they have no conflicts of interest. The authors declare no financial relationships with any organizations that might have an interest in the submitted work in the previous 3 years; no other relationships or activities that could appear to have influenced the submitted work.

Previous Presentation of Information Reported in Manuscript: An oral presentation of this work was presented at the annual meeting of the American Society of Bone and Mineral Research in Minneapolis, Minnesota on October 14, 2012.

Listing of Online Supplemental Material: Supplemental Table 1

NIH-PA Author Manuscript
NIH-PA Author Manuscript
NIH-PA Author Manuscript

NIH-PA Author Manuscript

NIH-PA Author Manuscript

NIH-PA Author Manuscript

Methods—We conducted a prospective cohort study of supplemental calcium use and incident CVD in 74,245 women in the Nurses' Health Study (1984–2008) free of CVD and cancer at baseline. Calcium supplement intake was assessed every four years. Outcomes were incident coronary heart disease (CHD) (nonfatal or fatal MI) and stroke (ischemic or hemorrhagic), confirmed by medical record review.

Results—During 24 years of follow-up, 4,565 cardiovascular events occurred (2,709 CHD and 1,856 strokes). At baseline, women who took calcium supplements had higher levels of physical activity, smoked less, and had lower trans fat intake compared with those who did not take calcium supplements. After multivariable adjustment for age, body mass index, dietary calcium, vitamin D intake, and other CVD risk factors, the relative risk of CVD for women taking >1,000mg/day of calcium supplements compared with none was 0.82 (95% CI 0.74 to 0.92; p for trend <0.001). For women taking >1,000mg/day of calcium supplements compared with none, the multivariable-adjusted relative risk for CHD was 0.71 (0.61 to 0.83; p for trend<0.001) and for stroke was 1.03 (0.87 to 1.21; p for trend=0.61). The relative risks were similar in analyses limited to non-smokers, women without hypertension, and women who had regular physical exams.

Conclusions—Our findings do not support the hypothesis that calcium supplement intake increases CVD risk in women.

Keywords

Calcium Supplements; Cardiovascular Disease; Coronary Heart Disease; Stroke; Prospective Study

Introduction

Calcium supplements are widely taken in the United States [1] and there is growing interest in their association with cardiovascular disease (CVD). Calcium supplements may improve lipid profiles [2] and lower blood pressure [3]. On the other hand, they could increase CVD risk by elevating serum calcium [4], which could promote vascular calcification [5] and CVD events [6].

Previous studies on the association between calcium supplements and CVD have reported conflicting results [7–13]. In a post-hoc analysis of a randomized controlled trial (RCT) of calcium supplementation (1,000 mg/day) in healthy postmenopausal women (n=1,471) designed to study the effects of calcium on bone density and fracture risk, women taking calcium supplements had a two-fold increased risk of myocardial infarction (MI) [14]. However, there were only 31 adjudicated MIs in this study, and when unreported MI events were added from a national database of hospital admissions, the results were no longer statistically significant. In a subsequent meta-analysis of CVD in 11,921 participants in 11 RCTs, most of which included supplement doses≥1,000mg/day [15–24] and utilized measures of bone health as primary outcomes, calcium supplements were associated with 27% higher risk of MI [25] (mean follow-up of 4 years, total of 296 MIs). An updated meta-analysis [26] which included participants in the Women's Health Initiative (WHI) who were not taking calcium supplements at baseline also reported that co-administration of calcium supplements and vitamin D was associated with a 24% higher risk of MI. In contrast,

NIH-PA Author Manuscript

NIH-PA Author Manuscript

NIH-PA Author Manuscript

another meta-analysis [7] which included RCTs of calcium supplements alone [14, 18–19, 27] or in combination with vitamin D supplements [28–30] suggested no association between calcium supplements and CVD risk.

Because of the inherent limitations of post-hoc analyses of RCTs not specifically designed to test the effect of calcium supplementation on CVD (small numbers of CVD events, inclusion of non-adjudicated CVD cases, non-adherence to study protocols, lack of CVD covariate information, etc.), observational prospective cohort studies examining associations between calcium supplement use and CVD risk are needed. However, observational studies to date have not included follow-up beyond 11 years [31–33], a large number of events [12, 32] or repeated, detailed assessments of calcium supplement intake and dose [12–13, 31–32, 34].

To examine independent associations between calcium supplement use and the risk of CVD in women, we conducted a prospective analysis with 24 years of follow-up (1984–2008) among 74,245 women in the Nurses' Health Study (NHS) cohort without history of CVD or cancer at baseline.

Methods

Study Population

The Nurses' Health Study is an ongoing, prospective cohort study which began in 1976, enrolling 121,700 female registered nurses between the ages of 30 and 55 years of age. The cohort is followed with biennial mailed questionnaires that ask about lifestyle practices and newly diagnosed diseases. The follow-up has been >90% of the eligible person-time. This analysis includes 74,245 women participating in NHS (1984–2008) who completed the 1984 dietary questionnaire and were free of CVD and cancer at baseline. The study protocol was reviewed and approved by the Brigham and Women's Hospital Institutional Review Board.

Assessment of Supplemental Calcium Intake and Diet

To assess participants' calcium supplement intake and diet, we used semiquantitative food-frequency questionnaires (FFQ) which assessed participants' average intake over the previous year. The participants were asked to complete FFQs in 1984, 1986, 1990, 1994, 1998, 2002, and 2006. The intakes of supplemental calcium, vitamins A, D, and E in multivitamins or isolated form were determined by the brand, type, and frequency of reported use. We categorized study participants into 5 categories of calcium supplement intake: none, 1–100mg/day, 101–500mg/day, 501–1,000mg/day, and >1,000mg/day.

The FFQs also inquired about the intake of over 130 individual food items and 22 beverages in the previous year. Intake of calcium and other dietary factors were computed from the reported frequency of consumption of each specified unit of food and from U.S. Department of Agriculture data on the content of the relevant nutrient in specified portions. Nutrient values were adjusted for total energy intake to determine the nutrient composition of the diet independent of the total amount of food eaten.

NIH-PA Author Manuscript

NIH-PA Author Manuscript

NIH-PA Author Manuscript

The FFQ has been extensively validated [35]. In 173 NHS participants, nutrient intake reported on two FFQs was compared with four seven-day records kept by the nurses who weighed and measured everything they ate or drank [36]. The values for the nutrient data on the FFQs and the food diaries were correlated and the degree of reproducibility was not modified by obesity or other personal characteristics.

To examine dietary patterns, we also used responses to each FFQ to construct a Dietary Approaches to Stop Hypertension (DASH) score, which has been associated with lower CVD risk in NHS [37]. The DASH score focuses on 8 components that are emphasized or minimized in the DASH diet: high intake of fruits, vegetables, nuts and legumes, low-fat dairy products, and whole grains and low intake of sodium, sweetened beverages, and red and processed meats. We classified participants into quintiles according to their intake ranking (with reverse scoring for sodium, red and processed meats, and sweetened beverages) and summed up the component scores to obtain an overall DASH score ranging from 8 to 40. We updated the DASH score throughout the follow-up period.

Assessment of Nondietary Factors

Age, body mass index (<21.0, 21.0–22.9, 23.0–24.9, 25.0–26.9, 27.0–29.9, 30.0–32.9, 33.0–34.9, or ≥35.0 kg/m^2), race, smoking status (never, past, and current smoker of 1–14 cigarettes/day, 15–24 cigarettes/day, or ≥25 cigarettes/day), physical activity (in quintiles of metabolic equivalent task scores), history of diabetes, hypertension, high cholesterol, or osteoporosis, family history of heart disease, postmenopausal hormone use (never, past, current), bisphosphonate use, thiazide use, and aspirin use (<1, 1–2, 3–6, 7–14, or ≥ 15 tablets/week) were ascertained from the biennial questionnaires. Self-reported weight was highly reliable (r=0.97) among a subset of participants who underwent direct measurement of their weight [38]. Physical activity reported on the questionnaires has been previously validated in comparison with physical activity diaries (r=0.79) in a comparable cohort [39]. Self-reported hypertension and diabetes were previously validated in this cohort. Race or ethnicity was self-reported and categorized in this analysis as white, African-American, Hispanic and Asian.

Ascertainment of Cases

CVD outcomes were defined as CHD (nonfatal or fatal MI) and stroke (ischemic or hemorrhagic). To confirm newly diagnosed cases of CHD or stroke reported on the biennial questionnaires, we requested permission to access the participants' medical records and cases were confirmed by NHS study physicians. To confirm nonfatal MI, we used the World Health Organization criteria, which require typical symptoms plus either diagnostic electrocardiographic findings or elevated cardiac enzyme concentrations. Nonfatal strokes were confirmed by medical records according to the National Survey of Stroke [40] criteria, which requires a constellation of neurologic deficits of sudden or rapid onset lasting ≥ 24 hours or until death. Based on findings from computed tomography, magnetic resonance imaging, or autopsy, strokes were classified as ischemic (embolic or thrombotic), hemorrhagic (subarachnoid or intracerebral), or undetermined. Cerebrovascular diseases caused by infection, trauma, or malignancy, or silent strokes were excluded. Undetermined strokes were excluded from this analysis. CHD and stroke events for which confirmatory

NIH-PA Author Manuscript
NIH-PA Author Manuscript
NIH-PA Author Manuscript

information was obtained by interview or letter but for which medical records were unavailable were designated as "probable".

Deaths were identified by reports from next of kin, postal authorities, or by searching the National Death Index. CHD and stroke deaths were classified by examining autopsy reports, hospital records, or death certificates. Fatal CHD was confirmed by medical records or autopsy reports or if CHD was listed as the cause of death on the death certificate and there was prior evidence of CHD in the medical records or if the participant previously reported CHD. We designated as probable those cases in which CHD was the underlying cause on the death certificates but no prior knowledge of CHD was indicated and medical records concerning the death were unavailable. Similarly, we followed the same procedures to classify confirmed or probable fatal stroke cases.

Since the exclusion of probable CVD events (30% of CHD and 3% of stroke cases) did not alter the results, we included both confirmed and probable CVD cases in the main analysis.

Statistical Analyses

The study design was prospective; information on calcium supplement use was collected before the diagnosis of CVD. For each participant, person-months of follow-up were counted from the date on which the 1984 questionnaire was returned 1) to the date on which CVD was diagnosed or death occurred or 2) to June 30, 2008, whichever occurred first. Information on calcium supplement use and other covariates recorded in response to the 1984 questionnaire was updated on subsequent questionnaires. We allocated person-time of follow-up according to exposure status at the start of each follow-up period. Thus, it was possible for study participants to change from one calcium supplement category to another over time. If information on calcium supplement use or diet was missing at the start of a time period, the participant was excluded from that time period. Period-specific categories of calcium supplement intake were used in the analysis. The Mantel extension test was used to evaluate linear trends across categories of calcium supplement intake. We used Cox proportional-hazards regression to simultaneously adjust for CVD risk factors.

The final multivariable model included calcium supplement intake and the following variables: age, body mass index, race or ethnicity, alcohol intake, smoking status, physical activity, postmenopausal hormone use, aspirin use, multivitamin use, history of hypertension, diabetes, or high cholesterol, family history of heart disease, physical exam within the past two years, recent mammogram, and intakes (quintiles) of dietary calcium, total vitamin D, vitamin E, magnesium, trans fat, fiber, glycemic load, polyunsaturated fat-to-saturated fat ratio, and total energy.

We calculated 95% confidence intervals for all relative risks. All P values are two-tailed. Tests for trend were performed by assigning the median value to each category and modeling as a continuous variable. Data were analyzed with the Statistical Analysis Systems software package (version 9.2; SAS Institute Inc., Cary, North Carolina).

NIH-PA Author Manuscript

NIH-PA Author Manuscript

NIH-PA Author Manuscript

Results

Calcium Supplement Intake and CVD Risk

During 1,680,894 person-years of follow-up over a 24 year period, we confirmed 4,565 CVD cases (2,709 CHD events and 1,856 strokes). The characteristics of the cohort according to baseline calcium supplement category are shown in Table 1. At baseline, 30.5% of the study participants were taking calcium supplements. For our analyses, however, the updated calcium supplement values were used for each time period. Calcium supplement use increased over the study period so that by 2004, 80% of the study participants were taking calcium supplements.

The mean age, total vitamin D intake, postmenopausal hormone use, self-reported history of high cholesterol, and physical activity level increased with higher supplemental calcium intake. The percentage of current smokers decreased with greater supplemental calcium intake. The percentage of women with self-reported diabetes or hypertension and the mean daily intake of dietary calcium were similar across the calcium supplement categories. The Spearman correlation coefficients between calcium supplements and total and supplemental vitamin D intakes were 0.33 and 0.43, respectively.

After adjusting for age, the relative risk of CVD was 0.69 (95% CI 0.62, 0.76; p for trend<0.001) for women taking >1,000mg/day of calcium supplements compared with none (Table 2). After further adjusting for body mass index, vitamin D intake and other CVD risk factors, the relative risk of CVD was 0.82 (95% CI 0.74, 0.92; p for trend<0.001) for women taking >1,000mg/day of calcium supplements compared with none.

Calcium Supplement Intake and CHD Risk

During the 24-year study period, there were 2,151 nonfatal and 558 fatal CHD events. After adjusting for age, the relative risk of CHD was 0.56 (95% CI 0.49, 0.64; p for trend<0.001) for women taking >1,000mg/day of calcium supplements compared to none (Table 2). The multivariable-adjusted relative risk of CHD was 0.71 (95% CI 0.61, 0.83; p for trend<0.001).

The age-adjusted relative risk of nonfatal CHD for women taking >1,000mg/day of calcium supplements compared with none was 0.59 (95% CI 0.51, 0.69; p for trend <0.001) and was attenuated after multivariable adjustment (0.77; 95% CI 0.65, 0.91; p for trend<0.001) (Table 3). The age-adjusted relative risk of fatal CHD for women taking >1,000mg/day of calcium supplements compared with none was 0.45 (95% CI 0.33, 0.63; p for trend <0.001) and was attenuated and no longer significant after multivariable adjustment (0.71; 95% CI 0.50, 1.00; p for trend=0.06) (Table 3).

Calcium Supplement Intake and Stroke Risk

During the 24-year study period, there were 1,449 ischemic and 407 hemorrhagic strokes. After adjusting for age, the relative risk of stroke was 0.89 (95% CI 0.77, 1.04; p for trend=0.007) for women taking >1,000mg/day of calcium supplements compared with none,

NIH-PA Author Manuscript

NIH-PA Author Manuscript

NIH-PA Author Manuscript

but was no longer significant after multivariable adjustment (1.03; 95% CI 0.87, 1.21; p for trend=0.61) (Table 2).

We studied the association between calcium supplement use and subtype of stroke (Table 4). The age-adjusted relative risk of ischemic stroke was 0.85 (95% CI 0.72, 1.01; p for trend=0.003) and the multivariable-adjusted relative risk was 0.96 (95% CI 0.79, 1.16; p for trend=0.22) for women taking >1,000mg/day of calcium supplements compared with none. The age-adjusted relative risk of hemorrhagic stroke was 1.07 (95% CI 0.78, 1.46; p for trend=0.83) and the multivariable-adjusted relative risk was 1.32 (95% CI 0.94, 1.87; p for trend=0.24) for women taking >1,000mg/day of calcium supplements compared with none.

Additional Analyses

We also examined calcium supplement doses >1,500mg/day, cumulative average calcium supplement intake, duration of calcium supplement use, lifetime intake of calcium supplements, total calcium (dietary and supplemental) intake and risk of CVD. Compared with non-users, the multivariable-adjusted relative risk among calcium supplement users taking >1,500mg/day was 0.78 (95% CI 0.63, 0.96; p for trend<0.001) for CVD, 0.73 (95% CI 0.55, 0.97; p for trend<0.001) for CHD, and 0.84 (95% CI 0.61, 1.14; p for trend=0.46) for stroke. Since there were relatively few cases (105 CHD and 105 strokes) in participants with cumulative average calcium supplement intake >1,000mg/day, we examined cumulative average calcium supplement intake >500mg/day. Compared with non-users, the multivariable-adjusted relative risk was 0.89 (95% CI 0.81, 0.98; p for trend=0.02) for CVD, 0.79 (95% CI 0.70, 0.89; p for trend<0.001) for CHD, and 1.08 (95% CI 0.93, 1.25; p for trend=0.31) for stroke for cumulative average calcium supplement intake >500mg/day. We also examined the association between duration of calcium supplement use (none, 1–3.9 years, 4–7.9 years, 8–11.9 years, and ≥12 years of use) and CVD and did not find an association in multivariable analysis (Online Supplemental Table 1). We also did not find an association between lifetime calcium supplement intake and higher CVD risk. We also examined the association between total calcium (dietary and supplemental) intake and CVD risk. For women with total calcium intake >2,000mg/day compared to women with a total calcium intake 1–500mg/day, the multivariable adjusted relative risk for CVD was 0.92 (95% CI 0.79, 1.07).

The multivariable-adjusted relative risks for the association between calcium supplement use and CVD were similar in analyses restricted to women who were non-smokers, women without hypertension, women without high cholesterol, and women who had regular mammograms and/or regular physical exams. We also found the association was unchanged after additional adjustment for thiazide use, menopausal status, and the DASH dietary score.

We also performed multivariable-adjusted analyses stratified by median vitamin D intake, median dietary calcium intake, multivitamin (MVI) use (yes/no), and postmenopausal hormone use (never and past vs. current users). Associations between calcium supplement use and CVD risk were similar in participants above and below the median intakes of vitamin D and dietary calcium, and were similar in users and non-users of both MVIs and postmenopausal hormones.

NIH-PA Author Manuscript

NIH-PA Author Manuscript

NIH-PA Author Manuscript

We also examined women who newly started calcium supplements (i.e., women who were not taking calcium supplements in 1984 and subsequently took calcium supplements), and did not observe an association between calcium supplement use and increased risk of CVD. In additional multivariable-adjusted analyses including all self-reported non-fatal CVD events (rather than investigator confirmed and probable events), the association between calcium supplement intake >1,000mg/day compared with none was 0.85 (95% CI 0.77, 0.93; p for trend<0.001) for non-fatal CVD and 0.82 (95% CI 0.72, 0.92; p for trend<0.001) for non-fatal CHD. Finally, no significant interaction was found between age and calcium supplement intake (p values for interaction 0.14 for CVD, 0.32 for CHD, and 0.39 for stroke).

Discussion

In our long term prospective study of women, calcium supplement intake was not associated with increased risk of CVD. Rather, we observed an inverse association between calcium supplement use and CHD risk. Our study has several distinct strengths that distinguish it from other reports, including the large number of events that were confirmed by medical record review, large sample size, long-term follow-up, detailed and repeated assessment of calcium supplement use that allowed for examination of dose-response relationship, and assessment and inclusion of CVD risk factors as covariates.

Observational prospective studies to date on calcium supplements and CHD risk in women [12–13, 31–34] have reported disparate results. The Iowa Women's Health Study [34] of 34,486 postmenopausal women with 8 years of follow-up did not find a risk of fatal CHD associated with taking ≥500mg/day of calcium supplements versus none (RR 0.88; 95% CI 0.64, 1.23). In the National Institutes of Health-AARP Diet and Health Study [13] with 12 years of follow-up, no association was found between supplemental calcium intake (assessed once at baseline) and CVD death (RR 1.06; 95% CI 0.96, 1.18), heart disease death (RR 1.05; 0.93, 1.18) or stroke death (RR 1.08; 0.87, 1.33) in women (although some positive associations were observed in men).

In contrast, the Kuopio Osteoporosis Risk Factor and Prevention Study [31] of 10,555 Finish women aged 52–62 years old reported an increased risk of CHD (RR 1.24; 95% CI 1.02, 1.52) associated with calcium supplement use. However, this study assessed calcium supplement intake as a binary variable (yes/no) only once at baseline and had < 7 years of follow-up. The European Prospective Investigation into Cancer and Nutrition study (EPIC-Heidelberg) [32] of 23,980 men and women, aged 35–64 years old, found that calcium supplement users compared to non-users had an increased MI risk (RR 1.86; 95% CI 1.17, 2.96). However, the prevalence of calcium supplement use in this study was only 3.6%, which is substantially lower than that observed in a German elderly population [41] and in the U.S. [1], raising the possibility of important differences between study populations. In the Swedish mammographic cohort [12], use of calcium supplements was not associated with all-cause mortality, but participants with dietary calcium > 1,400 mg/d who also used calcium supplements had higher CVD mortality (RR=2.57; 95% CI 1.19, 5.55). However, this finding was based on only 7 deaths. In addition, the prevalence of calcium supplement use in the Swedish mammographic cohort was only 6% [42–43]. Finally, there was

NIH-PA Author Manuscript
NIH-PA Author Manuscript
NIH-PA Author Manuscript

significant loss to follow-up in this study (only 63.5% of baseline participants were included in the calcium supplement analysis).

To date, no RCT has tested the effect of calcium supplementation on CVD as its primary endpoint. However, Bolland et al. conducted secondary analyses of calcium supplement RCTs with non-CVD endpoints and reported positive associations between calcium supplements and CHD [14, 25–26]. No association was found with other cardiovascular outcomes. Although these well conducted analyses have numerous distinct strengths, some investigators [7] suggest interpreting with caution because: 1) the studies are post-hoc analyses of RCTs not specifically designed to test the effect of calcium supplementation on CVD, 2) the number of CVD events was relatively small, 3) most of the CVD events were not adjudicated, 4) most of the individual trials in the meta-analyses did not report a significant difference in CVD events between the calcium and placebo groups, and 5) the meta-analyses utilized unpublished (in addition to published) data. Additional potential limitations include non-adherence by participants to study protocols and lack of covariate information for many participants [44].

The relationship between calcium supplements and CVD risk in WHI has generated controversy. A recent analysis of participants in the WHI RCT and the WHI prospective observational study (OS) by Prentice et al. did not find an association with CVD among women taking calcium supplements alone (OS) or calcium in combination with vitamin D (RCT, OS, and combined RCT and OS analysis) [33]. In contrast, the results of the updated meta-analysis by Bolland et al. [26] included WHI and reported a positive association between calcium supplements and MI risk. Of note, the study by Bolland et al. only included a subset of WHI participants who were not taking calcium supplements at baseline.

Because CVD risk associated with calcium supplements in some previous reports was attenuated when utilizing confirmed (rather than self-reported) cases of CVD, some investigators have suggested that gastrointestinal side effects from calcium supplement use may have led to misreports of MIs [45]. For example, in a study combining data from two RCTs of calcium supplementation [14, 19], self-reported MI rates were higher in the calcium group compared to the placebo group (3.6% vs. 2.1%; p=0.02), but after adjudication, the MI rates decreased in the calcium group from 3.6% to 2.4% and the difference between the calcium and placebo group was no longer statistically significant [45]. In our study, we performed analyses examining the association between calcium supplements and self-reported non-fatal CVD and CHD cases and did not observe substantial changes in our risk estimates.

A major limitation to our observational study is the possibility that residual confounding and/or confounding by unknown or unmeasured factors related to better cardiovascular health may have contributed to the inverse association between calcium supplement use and risk of CHD. To address this, we performed a wide variety of subgroup-analyses (such as restriction to women without hypertension, women without high cholesterol, and non-smokers). We also adjusted our analyses for behaviors that may be associated with overall health status, such as regular physical exams, screenings (i.e., mammograms), and the use of multivitamins. Thus, it is also possible that supplemental calcium intake lowers CVD risk.

NIH-PA Author Manuscript

NIH-PA Author Manuscript

NIH-PA Author Manuscript

Calcium supplements may improve lipid profiles [2] and lower blood pressure [3], and previous observational studies have reported inverse associations between calcium intake and CHD (e.g. the Iowa Women's Health Study [34] and the WHI follow-up study by Prentice et al. [33]).

There are other limitations to our study. Many calcium supplements also contain vitamin D. It is possible that study participants taking supplemental calcium under-reported their supplemental vitamin D intake. In addition, socioeconomic variables were not included in our models. Finally, since our study population was female and almost entirely white, our findings are not necessarily generalizable to men or other races.

In this large-scale, long-term prospective cohort study in women, calcium supplement use was inversely associated with risk of CHD. Our findings do not support an increased risk of CVD with calcium supplement use in women. Additional prospective cohort studies examining potential CVD risk associated with calcium supplement use are needed, and future randomized trials of calcium supplementation, if conducted, should be designed to optimize assessment of CVD events.

Supplementary Material

Refer to Web version on PubMed Central for supplementary material.

Acknowledgments

We are indebted to the participants in the Nurses' Health Study for their continuing cooperation.

Funding: This research was supported by the National Institutes of Health grants HL092947, HL34594, HL088521, DK091417, and CA087969.

REFERENCES

1. Gahche J, Bailey R, Burt V, Hughes J, Yetley E, Dwyer J, Picciano M, McDowell M, Sempos C. Dietary supplement use among U.S. adults has increased since NHANES III (1988–1994). NCHS Data Brief. 2011; 61:1–8. [PubMed: 21592424]
2. Reid I, Mason B, Horne A, Ames R, Clearwater J, Bava U, Orr-Walker B, Wu F, Evans M, Gamble G. Effects of calcium supplementation on serum lipid concentrations in normal older women: a randomized controlled trial. Am J Med. 2002; 112:343–347. [PubMed: 11904107]
3. Griffith L, Guyatt G, Cook R, Bucher H, Cook D. The influence of dietary and nondietary calcium supplementation on blood pressure: an updated metaanalysis of randomized controlled trials. Am J Hypertens. 1999; 12(1 Pt 1):84–92. [PubMed: 10075392]
4. Heller HJ, Greer LG, Haynes SD, Poindexter JR, Pak CY. Pharmacokinetic and pharmacodynamic comparison of two calcium supplements in postmenopausal women. J Clin Pharmacol. 2000; 40:1237–1244. [PubMed: 11075309]
5. Rubin M, Rundek T, McMahon D, Lee H, Sacco R, Silverberg S. Carotid artery plaque thickness is associated with increased serum calcium levels: the Northern Manhattan study. Atherosclerosis. 2007; 194:426–432. [PubMed: 17030035]
6. Foley RN, Collins AJ, Ishani A, Kalra PA. Calcium-phosphate levels and cardiovascular disease in community-dwelling adults: the Atherosclerosis Risk in Communities (ARIC) Study. Am Heart J. 2008; 156:556–563. [PubMed: 18760141]
7. Wang L, Manson JE, Sesso HD. Calcium intake and risk of cardiovascular disease: a review of prospective studies and randomized clinical trials. Am J Cardiovasc Drugs. 2012; 12:105–116. [PubMed: 22283597]

NIH-PA Author Manuscript NIH-PA Author Manuscript NIH-PA Author Manuscript

8. Reid IR, Bolland MJ, Avenell A, Grey A. Cardiovascular effects of calcium supplementation. Osteoporosis International. 2011; 22:1649–1658. [PubMed: 21409434]
9. Wang L, Manson J, Song Y, Sesso H. Systematic review: Vitamin D and calcium supplementation in prevention of cardiovascular events. Ann Intern Med. 2010; 152:315–323. [PubMed: 20194238]
10. Abrahamsen B, Sahota O. Do calcium plus vitamin D supplements increase cardiovascular risk? BMJ. 2011; 342:d2080. [PubMed: 21505220]
11. Reid IR, Bolland MJ, Grey A. Does calcium supplementation increase cardiovascular risk? Clinical Endocrinology. 2010; 73:689–695. [PubMed: 20184602]
12. Michaelsson K, Melhus H, Warensjo Lemming E, Wolk A, Byberg L. Long term calcium intake and rates of all cause and cardiovascular mortality: community based prospective longitudinal cohort study. BMJ. 2013; 346:f228. [PubMed: 23403980]
13. Xiao Q, Murphy RA, Houston DK, Harris TB, Chow WH, Park Y. Dietary and Supplemental Calcium Intake and Cardiovascular Disease Mortality: The National Institutes of Health-AARP Diet and Health Study. JAMA Intern Med. 2013:1–8.
14. Bolland M, Barber P, Doughty R, Mason B, Horne A, Ames R, Gamble G, Grey A, Reid I. Vascular events in healthy older women receiving calcium supplementation: randomised controlled trial. BMJ. 2008; 336:262–266. [PubMed: 18198394]
15. Reid IR, Ames RW, Evans MC, Gamble GD, Sharpe SJ. Long-term effects of calcium supplementation on bone loss and fractures in postmenopausal women: a randomized controlled trial. Am J Med. 1995; 98:331–335. [PubMed: 7709944]
16. Reid IR, Mason B, Horne A, Ames R, Reid HE, Bava U, Bolland MJ, Gamble GD. Randomized controlled trial of calcium in healthy older women. Am J Med. 2006; 119:777–785. [PubMed: 16945613]
17. Reid IR, Ames RW, Evans MC, Gamble GD, Sharpe SJ. Effect of calcium supplementation on bone loss in postmenopausal women. N Engl J Med. 1993; 328:460–464. [PubMed: 8421475]
18. Baron JA, Beach M, Mandel JS, et al. Calcium supplements for the prevention of colorectal adenomas. Calcium Polyp Prevention Study Group. N Engl J Med. 1999; 340:101–107. [PubMed: 9887161]
19. Prince RL, Devine A, Dhaliwal SS, Dick IM. Effects of calcium supplementation on clinical fracture and bone structure: results of a 5-year, double-blind, placebo-controlled trial in elderly women. Arch Intern Med. 2006; 166:869–875. [PubMed: 16636212]
20. Riggs BL, O'Fallon WM, Muhs J, O'Connor MK, Kumar R, Melton LJ 3rd. Long-term effects of calcium supplementation on serum parathyroid hormone level, bone turnover, and bone loss in elderly women. J Bone Miner Res. 1998; 13:168–174. [PubMed: 9495509]
21. Bonnick S, Broy S, Kaiser F, Teutsch C, Rosenberg E, DeLucca P, Melton M. Treatment with alendronate plus calcium, alendronate alone, or calcium alone for postmenopausal low bone mineral density. Curr Med Res Opin. 2007; 23:1341–1349. [PubMed: 17594775]
22. Grant AM, Avenell A, Campbell MK, et al. Oral vitamin D3 and calcium for secondary prevention of low-trauma fractures in elderly people (Randomised Evaluation of Calcium Or vitamin D, RECORD): a randomised placebo-controlled trial. Lancet. 2005; 365:1621–1628. [PubMed: 15885294]
23. Bonithon-Kopp C, Kronborg O, Giacosa A, Rath U, Faivre J. Calcium and fibre supplementation in prevention of colorectal adenoma recurrence: a randomised intervention trial. European Cancer Prevention Organisation Study Group. Lancet. 2000; 356:1300–1306. [PubMed: 11073017]
24. Lappe JM, Travers-Gustafson D, Davies KM, Recker RR, Heaney RP. Vitamin D and calcium supplementation reduces cancer risk: results of a randomized trial. Am J Clin Nutr. 2007; 85:1586–1591. [PubMed: 17556697]
25. Bolland M, Avenell A, Baron J, Grey A, MacLennan G, Gamble G, Reid I. Effect of calcium supplements on risk of myocardial infarction and cardiovascular events: meta-analysis. BMJ. 2010; 341:c3691. [PubMed: 20671013]
26. Bolland MJ, Grey A, Avenell A, Gamble GD, Reid IR. Calcium supplements with or without vitamin D and risk of cardiovascular events: reanalysis of the Women's Health Initiative limited access dataset and meta-analysis. BMJ. 2011; 342:d2040. [PubMed: 21505219]

NIH-PA Author Manuscript

NIH-PA Author Manuscript

NIH-PA Author Manuscript

27. Lewis JR, Calver J, Zhu K, Flicker L, Prince RL. Calcium supplementation and the risks of atherosclerotic vascular disease in older women: results of a 5-year RCT and a 4.5-year follow-up. J Bone Miner Res. 2011; 26:35–41. [PubMed: 20614474]
28. Brazier M, Grados F, Kamel S, Mathieu M, Morel A, Maamer M, Sebert JL, Fardellone P. Clinical and laboratory safety of one year's use of a combination calcium + vitamin D tablet in ambulatory elderly women with vitamin D insufficiency: results of a multicenter, randomized, double-blind, placebo-controlled study. Clin Ther. 2005; 27:1885–1893. [PubMed: 16507374]
29. Hsia J, Heiss G, Ren H, et al. Calcium/vitamin D supplementation and cardiovascular events. Circulation. 2007; 115:846–854. [PubMed: 17309935]
30. Lappe JM, Heaney RP. Calcium supplementation: Results may not be generalisable. BMJ. 2008; 336(author reply 404):403. [PubMed: 18292136]
31. Pentti K, Tuppurainen M, Honkanen R, Sandini L, Kröger H, Alhava E, Saarikoski S. Use of calcium supplements and the risk of coronary heart disease in 52–62-year-old women: The Kuopio Osteoporosis Risk Factor and Prevention Study. Maturitas. 2009; 63:73–78. [PubMed: 19394167]
32. Li K, Kaaks R, Linseisen J, Rohrmann S. Associations of dietary calcium intake and calcium supplementation with myocardial infarction and stroke risk and overall cardiovascular mortality in the Heidelberg cohort of the European Prospective Investigation into Cancer and Nutrition study (EPIC-Heidelberg). Heart. 2012; 98:920–925. [PubMed: 22626900]
33. Prentice RL, Pettinger MB, Jackson RD, et al. Health risks and benefits from calcium and vitamin D supplementation: Women's Health Initiative clinical trial and cohort study. Osteoporosis International. 2013; 24:567–580. [PubMed: 23208074]
34. Bostick R, Kushi L, Wu Y, Meyer K, Sellers T, Folsom A. Relation of calcium, vitamin D, and dairy food intake to ischemic heart disease mortality among postmenopausal women. Am J Epidemiol. 1999; 149:151–161. [PubMed: 9921960]
35. Willett W, Sampson L, Stampfer M, Rosner B, Bain C, Witschi J, Hennekens C, Speizer F. Reproducibility and validity of a semiquantitative food frequency questionnaire. Am J Epidemiol. 1985; 122:51–65. [PubMed: 4014201]
36. Salvini S, Hunter D, Sampson L, Stampfer M, Colditz G, Rosner B, Willett W. Food-based validation of a dietary questionnaire: the effects of week-to-week variation in food consumption. Int J Epidemiol. 1989; 18:858–867. [PubMed: 2621022]
37. Fung TT, Chiuve SE, McCullough ML, Rexrode KM, Logroscino G, Hu FB. Adherence to a DASH-style diet and risk of coronary heart disease and stroke in women. Arch Intern Med. 2008; 168:713–720. [PubMed: 18413553]
38. Rimm E, Stampfer M, Colditz GA, Chute C, Litin L, Willett W. Validity of self-reported waist and hip circumference in men and women. Epidemiology. 1990; 1:466–473. [PubMed: 2090285]
39. Wolf A, Hunter D, Colditz G, Manson J, Stampfer M, Corsano K, Rosner B, Kriska A, Willett W. Reproducibility and validity of a self-administered physical activity questionnaire. Int J Epidemiol. 1994; 23:991–999. [PubMed: 7860180]
40. Walker AE, Robins M, Weinfeld FD. The National Survey of Stroke. Clinical findings. Stroke. 1981; 12:I13–I44. [PubMed: 7222164]
41. Schwarzpaul S, Strassburg A, Luhrmann PM, Neuhauser-Berthold M. Intake of vitamin and mineral supplements in an elderly german population. Ann Nutr Metab. 2006; 50:155–162. [PubMed: 16407640]
42. Michaelsson K, Holmberg L, Mallmin H, Sorensen S, Wolk A, Bergstrom R, Ljunghall S. Diet and hip fracture risk: a case-control study. Study Group of the Multiple Risk Survey on Swedish Women for Eating Assessment. Int J Epidemiol. 1995; 24:771–782. [PubMed: 8550275]
43. Michaelsson K, Melhus H, Bellocco R, Wolk A. Dietary calcium and vitamin D intake in relation to osteoporotic fracture risk. Bone. 2003; 32:694–703. [PubMed: 12810177]
44. Nordin BE, Lewis JR, Daly RM, Horowitz J, Metcalfe A, Lange K, Prince RL. The calcium scare--what would Austin Bradford Hill have thought? Osteoporosis International. 2011; 22:3073–3077. [PubMed: 21633827]
45. Lewis JR, Zhu K, Prince RL. Adverse events from calcium supplementation: relationship to errors in myocardial infarction self-reporting in randomized controlled trials of calcium supplementation. J Bone Miner Res. 2012; 27:719–722. [PubMed: 22139587]

NIH-PA Author Manuscript
NIH-PA Author Manuscript
NIH-PA Author Manuscript

Table 1

Age-Standardized Baseline Characteristics of Women According to Categories of Supplemental Calcium Intake in 1984

	Supplemental Calcium Intake				
	**None (n=51,623)**	**1–100 mg (n=1,617)**	**101–500 mg (n=14,699)**	**501–1000 mg (n=4,516)**	**>1000 mg (n=1,790)**
Age, years[a]	49.6 ±7.2	48.9 ±7.2	51.8 ± 6.8	52.0 ± 6.6	52.4 ± 6.5
BMI, kg/m^2	25.2 ± 4.8	24.7 ± 4.5	24.5 ± 4.4	24.4 ± 4.3	24.4 ± 4.5
Physical Activity, METS/week	13.2 ± 20.2	14.6 ± 19.7	16.2 ± 23.6	16.8 ± 20.8	18.1 ± 24.3
Dietary Calcium Intake, mg/day	708 ± 251	726 ± 239	720 ± 247	733 ± 265	730 ± 269
Supplemental Calcium Intake, mg/day	0	52 ± 34	350 ± 152	893 ± 170	1408 ± 209
Total Vitamin D Intake, IU/day	256 ± 190	417 ± 264	428 ± 290	474 ± 327	560 ± 378
Dietary Vitamin D Intake, IU/day	187 ± 97	190 ± 92	189 ± 98	190 ± 102	187 ± 103
Vitamin E intake, mg/day	26 ± 60	54 ± 89	76 ± 105	98 ± 119	138 ± 152
Magnesium, mg/day	275 ± 67	318 ± 70	313 ± 81	331 ± 96	348 ± 110
Retinol, IU/day	3,351 ± 3,444	6,929 ± 7,066	6,378 ± 6,118	7,802 ± 7,956	10,134 ± 10,576
Total energy intake, kcal/day	1,754 ± 534	1,788 ± 528	1,727 ± 524	1,695 ± 514	1,672 ± 527
Trans fat intake, % of total energy	2.0 ± 0.6	1.9 ± 0.6	1.8 ± 0.6	1.7 ± 0.6	1.7 ± 0.6
Polyunsaturated-to-saturated fat ratio	0.5 ± 0.2	0.5 ± 0.2	0.6 ± 0.2	0.6 ± 0.2	0.6 ± 0.2
Glycemic Load	99.0 ± 19.5	99.2 ± 19.2	99.1 ± 19.9	99.5 ± 20.4	99.3 ± 22.2
Cereal fiber intake, g/day	4.0 ± 2.2	4.2 ± 2.3	4.4 ± 2.4	4.6 ± 2.6	4.5 ± 2.8
Alcohol, gm/day	6.8 ± 11.3	6.7 ± 11.1	7.2 ± 11.3	6.9 ± 10.8	6.9 ± 11.3
White Race, %	97.7	98.1	97.8	97.9	98.5
Current Smoker, %	26.0	25.9	19.8	19.3	19.0
Postmenopausal, %	58.3	57.0	63.4	64.3	67.2
Postmenopausal Hormone Use, %	20.0	23.9	32.0	35.4	35.7
Hypertension, %	21.2	19.5	20.7	21.3	22.7
High Cholesterol, %	7.5	7.8	8.6	9.3	10.9
Diabetes, %	3.0	2.8	2.6	2.7	2.9
Family History of Heart Disease, %	19.4	18.7	18.8	18.7	19.9
Aspirin Use (at least once/month), %	70.3	73.2	74.6	72.7	70.2
Multivitamin Supplement User, %	24.0	100.0	63.6	68.6	77.1

NIH-PA Author Manuscript NIH-PA Author Manuscript NIH-PA Author Manuscript

[a]Value is not age adjusted.

Values are means ± SD or percentages and are standardized to the age distribution of the study population.

NIH-PA Author Manuscript
NIH-PA Author Manuscript
NIH-PA Author Manuscript

Table 2

Age-Adjusted and Multivariate Relative Risks for Cardiovascular Disease According to Calcium Supplement Intake Category [a]

	None	1–100 mg/day	101–500 mg/day	501–1000 mg/day	>1000 mg/day	P for Trend
**Total CVD**						
Cases of CVD (n)	1,863	211	1,151	806	514	
Person-years (n)	684,044	64,174	429,584	310,161	192,931	
Age-adjusted Relative Risk (95% CI)	1.00	0.92 (0.80, 1.06)	0.80 (0.74, 0.86)	0.71 (0.65, 0.78)	0.69 (0.62, 0.76)	<0.0001
Multivariate Relative Risk (95% CI) [b]	1.00	0.97 (0.84, 1.12)	0.88 (0.81, 0.95)	0.83 (0.76, 0.92)	0.82 (0.74, 0.92)	0.0008
**Total CHD**						
Cases of CHD (n)	1,173	128	684	463	261	
Person-years (n)	682,662	63,985	428,626	309,461	192,417	
Age-adjusted Relative Risk (95% CI)	1.00	0.90 (0.75, 1.09)	0.77 (0.70, 0.84)	0.66 (0.59, 0.74)	0.56 (0.49, 0.64)	<0.0001
Multivariate Relative Risk (95% CI) [b]	1.00	0.97 (0.80, 1.16)	0.86 (0.78, 0.95)	0.81 (0.72, 0.91)	0.71 (0.61, 0.83)	<0.0001
**Total Stroke**						
Cases of Total Stroke (n)	710	83	467	343	253	
Person-years (n)	684,304	64,159	429,622	310,154	192,873	
Age-adjusted Relative Risk (95% CI)	1.00	0.94 (0.75, 1.19)	0.86 (0.77, 0.97)	0.80 (0.70, 0.91)	0.89 (0.77, 1.04)	0.007
Multivariate Relative Risk (95% CI) [b]	1.00	0.98 (0.78, 1.24)	0.93 (0.82, 1.05)	0.90 (0.78, 1.05)	1.03 (0.87, 1.21)	0.61

[a] Calcium supplement use was updated throughout the analysis period (1984–2008). Relative risks are for the risk of CVD, CHD, or stroke compared with the group that took no calcium supplements.

[b] The multivariate model includes: diet calcium intake, total vitamin D intake, vitamin E intake, magnesium intake, multivitamin use, BMI, family history of heart disease, smoking status, alcohol intake, postmenopausal hormone use, physical activity, race, aspirin use, history of hypertension, diabetes, or high cholesterol, glycemic load, trans fat intake, polyunsaturated fat-to-saturated fat ratio, fiber intake, total energy intake, recent physical exam, and recent mammogram.

NIH-PA Author Manuscript NIH-PA Author Manuscript NIH-PA Author Manuscript

Table 3

Age-Adjusted and Multivariate Relative Risks for Coronary Heart Disease Subcategories According to Calcium Supplement Intake Category[a]

	None	1–100 mg/day	101–500 mg/day	501–1000 mg/day	>1000 mg/day	P for Trend
**Nonfatal CHD**						
Cases of Nonfatal CHD (n)	909	100	551	375	216	
Person-years (n)	682,665	63,986	428,626	309,462	192,417	
Age-adjusted Relative Risk (95% CI)	1.00	0.92 (0.74, 1.13)	0.80 (0.72, 0.89)	0.68 (0.60, 0.77)	0.59 (0.51, 0.69)	<0.0001
Multivariate Relative Risk (95% CI) [b]	1.00	0.97 (0.79, 1.20)	0.91 (0.82, 1.02)	0.85 (0.74, 0.97)	0.77 (0.65, 0.91)	0.0004
**Fatal CHD**						
Cases of Fatal CHD (n)	264	28	133	88	45	
Person-years (n)	683,493	64,063	429,123	309,783	192,594	
Age-adjusted Relative Risk (95% CI)	1.00	0.86 (0.58, 1.28)	0.65 (0.53, 0.81)	0.59 (0.46, 0.76)	0.45 (0.33, 0.63)	<0.0001
Multivariate Relative Risk (95% CI) [b]	1.00	0.96 (0.65, 1.43)	0.83 (0.66, 1.03)	0.88 (0.67, 1.14)	0.71 (0.50, 1.00)	0.06

[a]. Calcium supplement use was updated throughout the analysis period (1984–2008). Relative risks are for the risk of nonfatal or fatal CHD compared with the group that took no calcium supplements.

[b]The multivariate model includes: diet calcium intake, total vitamin D intake, vitamin E intake, magnesium intake, multivitamin use, BMI, family history of heart disease, smoking status, alcohol intake, postmenopausal hormone use, physical activity, race, aspirin use, history of hypertension, diabetes, or high cholesterol, glycemic load, trans fat intake, polyunsaturated fat-to-saturated fat ratio, fiber intake, total energy intake, recent physical exam, recent mammogram.

NIH-PA Author Manuscript
NIH-PA Author Manuscript
NIH-PA Author Manuscript

Table 4

Age-Adjusted and Multivariate Relative Risks for Stroke Subcategories According to Calcium Supplement Intake Category [a]

	None	1–100 mg/day	101–500 mg/day	501–1000 mg/day	>1000 mg/day	P for Trend
**Ischemic Stroke**						
Cases of Ischemic Stroke (n)	549	62	383	262	193	
Person-years (n)	684,127	64,138	429,543	310,076	192,806	
Age-adjusted Relative Risk (95% CI)	1.00	0.89 (0.68, 1.16)	0.90 (0.79, 1.03)	0.77 (0.66, 0.90)	0.85 (0.72, 1.01)	0.003
Multivariate Relative Risk (95% CI) [b]	1.00	0.91 (0.70, 1.19)	0.96 (0.83, 1.10)	0.86 (0.73, 1.01)	0.96 (0.79, 1.16)	0.22
**Hemorrhagic Stroke**						
Cases of Hemorrhagic Stroke (n)	161	21	84	81	60	
Person-years (n)	683,673	64,084	429,203	309,861	192,661	
Age-adjusted Relative Risk (95% CI)	1.00	1.16 (0.73, 1.84)	0.72 (0.55, 0.94)	0.91 (0.69, 1.21)	1.07 (0.78, 1.46)	0.83
Multivariate Relative Risk (95% CI) [b]	1.00	1.27 (0.80, 2.02)	0.82 (0.62, 1.08)	1.09 (0.81, 1.48)	1.32 (0.94, 1.87)	0.24

[a]. Calcium supplement use was updated throughout the analysis period (1984–2008). Relative risks are for the risk of ischemic stroke or hemorrhagic stroke compared with the group that took no calcium supplements.

[b]The multivariate model includes: diet calcium intake, total vitamin D intake, vitamin E intake, magnesium intake, multivitamin use, BMI, family history of heart disease, smoking status, alcohol intake, postmenopausal hormone use, physical activity, race, aspirin use, history of hypertension, diabetes, or high cholesterol, glycemic load, trans fat intake, polyunsaturated fat-to-saturated fat ratio, fiber intake, total energy intake, recent physical exam, recent mammogram.
