# Supplementary material for: A systematic comprehensive longitudinal evaluation of dietary factors associated with acute myocardial infarction and fatal coronary heart disease
Source: Nat Commun. 2020 Nov 27;11:6074. doi: 10.1038/s41467-020-19888-2 (PMC7699643; doi:10.1038/s41467-020-19888-2)
Supplement: Supplementary file 4 — Supplementary Software [file 41467_2020_19888_MOESM4_ESM.zip › EWAS-NHS-master/KG-SI/Rest/Rimm (1993).pdf]

VITAMIN E CONSUMPTION AND THE RISK OF CORONARY HEART DISEASE IN MEN

Eric B. Rimm, Sc.D., Meir J. Stampfer, M.D., Alberto Ascherio, M.D., Edward Giovannucci, M.D., Graham A. Colditz, M.B., B.S., and Walter C. Willett, M.D.

Abstract *Background.* The oxidative modification of low-density lipoproteins increases their incorporation into the arterial intima, an essential step in atherogenesis. Although dietary antioxidants, such as vitamin C, carotene, and vitamin E, have been hypothesized to prevent coronary heart disease, prospective epidemiologic data are sparse.

Methods. In 1986, 39,910 U.S. male health professionals 40 to 75 years of age who were free of diagnosed coronary heart disease, diabetes, and hypercholesterolemia completed detailed dietary questionnaires that assessed their usual intake of vitamin C, carotene, and vitamin E in addition to other nutrients. During four years of follow-up, we documented 667 cases of coronary disease.

Results. After controlling for age and several coronary risk factors, we observed a lower risk of coronary disease among men with higher intakes of vitamin E (P for trend = 0.003). For men consuming more than 60 IU per day of vitamin E, the multivariate relative risk was 0.64 (95 percent confidence interval, 0.49 to 0.83) as compared with those consuming less than 7.5 IU per day. As compared with men who did not take vitamin E supplements, men who took at least 100 IU per day for at least two years had a multivariate relative risk of coronary disease of 0.63 (95 percent confidence interval, 0.47 to 0.84). Carotene intake was not associated with a lower risk of coronary disease among those who had never smoked, but it was inversely associated with the risk among current smokers (relative risk, 0.30; 95 percent confidence interval, 0.11 to 0.82) and former smokers (relative risk, 0.60; 95 percent confidence interval, 0.38 to 0.94). In contrast, a high intake of vitamin C was not associated with a lower risk of coronary disease.

Conclusions. These data do not prove a causal relation, but they provide evidence of an association between a high intake of vitamin E and a lower risk of coronary heart disease in men. Public policy recommendations with regard to the use of vitamin E supplements should await the results of additional studies. (N Engl J Med 1993;328:1450-6.)

ANTIOXIDANTS such as vitamin C, carotenoids, and vitamin E[1,2] are hypothesized to help prevent atherosclerosis by blocking the oxidative modification of low-density lipoprotein (LDL), which may be selectively incorporated by monocytes in the arterial wall.[3,4] Oxidized LDL may also contribute to atherogenicity by reducing macrophage motility in the intima,[5] increasing monocyte accumulation,[6] and increasing cytotoxicity.[7]

Accumulating laboratory data support a link between dietary antioxidants and a reduced risk of atherosclerosis, but epidemiologic evidence is limited.[8-10] We therefore examined these relations in men enrolled in the Health Professionals Follow-up Study.

Methods

The Health Professionals Follow-up Study is a prospective investigation of 51,529 male health professionals who were 40 to 75 years of age in 1986. The study population included 29,683 dentists, 10,098 veterinarians, 4185 pharmacists, 3745 optometrists, 2218 osteopathic physicians, and 1600 podiatrists. The study began in 1986, when the participants completed a detailed questionnaire on diet and medical history. We mailed follow-up questionnaires in 1988 and 1990 to update information on exposure and to ascertain events related to newly diagnosed coronary disease.[11,12]

We used a priori criteria to exclude from the study 1530 men whose reported daily energy intake was outside the range of 800 to 4200 kcal or who left blank 70 or more questions about food on the dietary questionnaire. Because men with cardiovascular disease or related conditions may alter their dietary patterns after diagnosis, we excluded a further 10,089 men who reported myocardial infarction, angina, stroke, coronary-artery bypass grafting or angioplasty, diabetes, or hypercholesterolemia on the base-line 1986 questionnaire. The 39,910 remaining men were eligible for follow-up.

After repeated mailings, we received questionnaires from or confirmed the deaths of over 96 percent of eligible participants in 1988 and 1990.[13] The remaining nonresponding participants were assumed to be alive if they were not listed in the National Death Index.

Dietary Assessment

The 1986 dietary questionnaire inquired about the average frequency of intake of 131 foods over the previous year. Ten additional items specifically addressed the current use of vitamin supplements, including the type, dose, and duration. Although we have no information on the specific forms of vitamin E supplements (natural vs. synthetic), we did ask the participants to identify specific brands of multivitamins, cooking oils, and cold cereals. Nutrient intakes were computed by multiplying the frequency with which each food item or supplement was consumed by its nutrient content, derived primarily from information from the Department of Agriculture,[14] for the portion size or dose listed.[15-17] We calculated nutrient scores for both dietary and supplementary intake, except in the case of carotene, for which only the total intake was calculated, because only 2.2 percent of the men reported taking carotene supplements. Carotene intake was calculated on the basis of the values for vitamin A in vegetables and fruits and the one third of vitamin A from dairy products that is in the form of carotene.[14] We assigned a value of 10,000 IU for men who reported taking carotene supplements.

We assessed the validity of the food-frequency questionnaire in a random sample of 127 men living in the Boston area. We compared nutrient intake as specified on the questionnaire with two one-week diet records spaced approximately six months apart.[16-18] Pearson correlation coefficients between the diet records and the dietary questionnaire were adjusted for total energy intake[19] and for within-person variability in reported daily intake.[20] The adjusted correlation coefficients were 0.64 for carotene, 0.92 for total vitamin C, and 0.92 for total vitamin E. For men not taking supplements, the coefficients were lower: 0.77 for vitamin C and 0.42 for vitamin E.[16]

Case Ascertainment

Fatal coronary disease, nonfatal myocardial infarction, coronary-artery bypass grafting, and percutaneous transluminal coronary angioplasty occurring between 1986 and January 31, 1990, were considered as end points. Participants who reported an incident myocardial infarction on the 1988 or the 1990 questionnaire were

From the Departments of Epidemiology (E.B.R., M.J.S., A.A., G.A.C., W.C.W.) and Nutrition (A.A., W.C.W.), Harvard School of Public Health; and the Channing Laboratory, Department of Medicine, Harvard Medical School and Brigham and Women's Hospital (M.J.S., E.G., G.A.C., W.C.W.) — all in Boston. Address reprint requests to Dr. Rimm at the Department of Nutrition, Harvard School of Public Health, 665 Huntington Ave., Boston, MA 02115.

Supported by research grants (HL 35464 and CA 55075) from the National Institutes of Health.

The New England Journal of Medicine
Downloaded from nejm.org at NORTHEASTERN UNIVERSITY on November 18, 2017. For personal use only. No other uses without permission.
Copyright © 1993 Massachusetts Medical Society. All rights reserved.

sent a letter asking them to confirm the report and requesting permission to review the medical records. Myocardial infarctions were confirmed with use of the criteria of the World Health Organization[21]: compatible symptoms plus either typical electrocardiographic changes or elevation of cardiac enzymes. We classified 3 percent of the fatal and 18 percent of the nonfatal myocardial infarctions as "probable" because the medical records were unobtainable although the diagnosis was corroborated by supplementary information. Reports of bypass grafting or angioplasty were confirmed by medical records for 98 of 102 participants (96 percent). Therefore, participants' own reports of these end points were considered sufficient for confirmation.

Deaths were reported by next of kin, coworkers, postal authorities, or the National Death Index. Fatal infarctions were confirmed from medical records or autopsy reports. Fatal coronary disease was also considered confirmed if it was listed as the underlying cause on the death certificate, and a diagnosis of incident coronary disease (after January 1, 1986) was confirmed on the basis of records or interviews. The listing of a cause of death on the death certificate was not accepted in itself as confirming fatal coronary disease. Sudden death was defined as death within one hour of the onset of symptoms in men who had no previous serious illness or plausible cause of death other than coronary disease. Because in men sudden death is generally attributable to coronary disease, we included such deaths (for 33 men) as indicative of fatal coronary disease. When subjects had multiple end points, only the first was included in the analysis. The physicians reviewing the medical records were unaware of the reports of dietary intake.

Table 1. Relative Risk of Coronary Heart Disease, According to Quintile Group for Carotene, Vitamin C, and Vitamin E Intake among 39,910 Male Health Professionals.

Variable*	Quintile Group 1	2	3	4	5	P Value for Trend
**Vitamin E — median intake** (IU/day)	**6.4**	**8.5**	**11.2**	**25.2**	**419**	
Coronary disease — no. of cases	155	140	130	127	115	
Relative risk						
Age-adjusted	1.0	0.88	0.77	0.74	0.59	0.001
95% CI	—	0.70–1.10	0.61–0.98	0.59–0.93	0.47–0.75	
Multivariate	1.0	0.90	0.82	0.77	0.64	0.003
95% CI	—	0.71–1.14	0.64–1.07	0.60–0.98	0.49–0.83	
Multivariate with antioxidants	1.0	0.89	0.81	0.71	0.60	0.01
95% CI	—	0.70–1.14	0.62–1.05	0.54–0.92	0.44–0.81	
**Carotene — median intake** (IU/day)	**3969**	**6019**	**8114**	**11,653**	**19,034**	
Coronary disease — no. of cases	124	135	146	141	121	
Relative risk						
Age-adjusted	1.0	0.92	0.96	0.89	0.71	0.02
95% CI	—	0.73–1.18	0.75–1.22	0.70–1.14	0.55–0.92	
Multivariate	1.0	0.93	0.93	0.87	0.71	0.02
95% CI	—	0.72–1.20	0.72–1.20	0.67–1.15	0.53–0.96	
Multivariate with antioxidants	1.0	0.92	0.91	0.86	0.71	0.03
95% CI	—	0.71–1.18	0.70–1.18	0.65–1.13	0.53–0.86	
**Vitamin C — median intake** (mg/day)	**92**	**149**	**218**	**392**	**1,162**	
Coronary disease — no. of cases	118	133	159	137	120	
Relative risk						
Age-adjusted	1.0	0.99	1.11	0.94	0.83	0.15
95% CI	—	0.77–1.27	0.87–1.40	0.74–1.21	0.64–1.08	
Multivariate	1.0	1.02	1.15	1.02	0.89	0.10
95% CI	—	0.79–1.32	0.89–1.48	0.78–1.32	0.68–1.16	
Multivariate with antioxidants	1.0	1.08	1.32	1.26	1.25	0.98
95% CI	—	0.84–1.41	1.01–1.71	0.95–1.67	0.91–1.71	

*Multivariate denotes multivariate logistic-regression analysis with control for age (in five-year categories), smoking status (never smoked, formerly smoked, or currently smoking less than 15, 15 to 24, or 25 or more cigarettes per day), body-mass index (the weight in kilograms divided by the square of the height in meters) (in quintile groups), total calories (in quintile groups), dietary fiber (in quintile groups), alcohol consumption (0, 0.1 to 15, 15.1 to 30, or 30.1 or more g per day), reported hypertension, regular aspirin use, physical activity (more than 90 minutes per week), parental history of myocardial infarction before the age of 60, and profession. Multivariate with antioxidants indicates that quintile groups for vitamin E, carotene, and vitamin C were added to the multivariate model. The lowest quintile group served as the reference category in each analysis. CI denotes confidence interval.

Statistical Analysis

Each participant's follow-up time began with the date of return of the 1986 questionnaire and continued until the diagnosis of an end point, death, or January 31, 1990, whichever came first. Relative risks were calculated by dividing the incidence rate of coronary disease among the men in each category of antioxidant intake by the rate for the men in the lowest category. Adjusted relative risks for age (in five-year categories) were derived by the Mantel–Haenszel method.[22] The Mantel extension test[23] was used to test for linear trends. To adjust for other risk factors, we used multiple logistic regression to generate odds ratios as an estimate of relative risk. In multivariate logistic models, we tested for significant monotonic trends by assigning each participant the median value for the category and modeling this value as a continuous variable. All P values are two-sided.

Results

During 139,883 person-years of follow-up, we documented 667 coronary end points: 360 bypass grafts or angioplasties, 201 nonfatal myocardial infarctions, and 106 fatal coronary events.

The age-adjusted and multivariate relative risks of coronary disease according to quintile group for the intake of vitamin E, carotene, and vitamin C (including supplements) are shown in Table 1. As compared with the men in the lowest quintile group for vitamin E intake, the men in the highest quintile group had an age-adjusted relative risk of coronary disease of 0.59 (95 percent confidence interval, 0.47 to 0.75; P for trend = 0.001). The relative risks were similar after multivariate adjustment. For a high intake of carotene, the age-adjusted relative risk of coronary disease was 0.71 (95 percent confidence interval, 0.55 to 0.92; P for trend = 0.02). The relative risks for carotene were also not materially altered by multivariate adjustment. A high intake of vitamin C was initially suggestive of a slight inverse association (age-adjusted relative risk, 0.83; 95 percent confidence interval, 0.64 to 1.08) (Table 1). After further adjustment for risk factors and the use of other antioxidants, however, the relative risk was 1.25 (95 percent confidence interval, 0.91 to 1.71; P for trend = 0.98).

All the participants in the two highest quintile groups for vitamin E intake used multivitamins or specific vitamin E supplements. We suspected that these men might differ substantially from those who did not take supplements, and we therefore exam-

The New England Journal of Medicine
Downloaded from nejm.org at NORTHEASTERN UNIVERSITY on November 18, 2017. For personal use only. No other uses without permission.
Copyright © 1993 Massachusetts Medical Society. All rights reserved.

ined the distribution of risk factors according to quintile group for total vitamin E consumption (Table 2). The prevalence of hypertension and the percentage of calories derived from fat were similar. Differences in the intake of dietary fiber, cholesterol, and total fat were generally proportional to absolute differences in caloric intake and were not due to differences in dietary composition. However, because patterns of smoking, fiber intake, aspirin use, and physical activity were different, we controlled for these variables in all the multivariate analyses.

When the 5804 men with base-line hypercholesterolemia or diabetes were included, the results were not appreciably different from those in Table 1. Also, there were no material differences between the specific diagnostic categories. For fatal coronary disease or nonfatal myocardial infarction, the multivariate relative risk between the highest and lowest quintile groups for vitamin E intake was 0.63 (95 percent confidence interval, 0.45 to 0.89), and for bypass grafting or angioplasty the relative risk was 0.68 (95 percent confidence interval, 0.48 to 0.97). The corresponding results for carotene and vitamin C were also similar to the results in Table 1. Excluding probable cases of myocardial infarction did not alter these results appreciably.

To assess further the role of vitamin E supplements, we divided total intake into dietary and supplemental sources. Quintiles of dietary vitamin E intake were calculated on the basis of dietary intake without supplements, whereas the dose categories for supplemental vitamin E were those specified in the base-line questionnaire (Table 3). The two lowest categories of supplement use represent men who reported using multiple vitamins or using vitamin E supplements infrequently. Among men taking any supplemental vitamin E, we found only a modest inverse association between the dose and the risk of coronary disease (P for trend = 0.22). The maximal reduction in risk was seen among men consuming 100 to 249 IU per day, with no further decrease at higher doses. We found a suggestion of an inverse association between dietary vitamin E and the risk of coronary disease among men who did not take vitamin supplements, with a relative risk of 0.79 (95 percent confidence interval, 0.54 to 1.15) for the highest as compared with the lowest quintile group (P for trend = 0.11) (Table 3). The variation in dietary vitamin E intake was much lower than the variation in intake from supplements; the median of the highest quintile group for dietary intake (12.9 IU per day) fell within the lowest category of supplemental intake. We also examined the association between the duration of vitamin use and the risk of coronary disease (Table 4). Using the categories for the duration of vitamin use that appeared on the base-line questionnaire (0 to 1, 2 to 4, 5 to 9, and 10 or more years), we found a suggestion of an inverse trend between the duration of vitamin E use and the risk of coronary disease. Men reporting use of vitamin E supplements for 10 or more years had a relative risk of 0.65 (95 percent confidence interval, 0.46 to 0.92) as compared with nonusers.

The multivariate relative risk of coronary disease among men taking specific vitamin E supplements (i.e., not multivitamins) was 0.75 (95 percent confidence interval, 0.61 to 0.93) as compared with nonusers. Among men who took vitamin E supplements in doses of at least 100 IU per day for two or more years, the relative risk was 0.63 (95 percent confidence interval, 0.47 to 0.84) as compared with nonusers after we controlled for multivitamin use.

Table 3. Relative Risk of Coronary Heart Disease, According to Quintile Group for Dietary Vitamin E Intake and Category of Supplemental Vitamin E.*

Variable	Quintile Group for Dietary Intake† 1	2	3	4	5	P Value for Trend
**Dietary vitamin E (IU/day)**	**1.6–6.9**	**7.0–8.1**	**8.2–9.3**	**9.4–11.0**	**11.1**	
Coronary disease — no. of cases	79	89	90	79	56	
No. of person-years	17,617	16,560	15,605	14,569	12,591	0.11
Relative risk‡	1.0	1.10	1.17	0.97	0.79	
95% CI	—	0.80–1.51	0.84–1.62	0.69–1.37	0.54–1.15	
**Supplemental vitamin E (IU/day)**	**0**	**<25**	**25–99**	**100–249**	**≥250**	
Coronary disease — no. of cases	406	120	40	17	84	
No. of person-years	79,699	26,197	9,607	5,677	18,700	0.22§
Relative risk‡	1.0	0.85	0.78	0.54	0.70	
95% CI	—	0.69–1.05	0.59–1.08	0.33–0.88	0.55–0.89	

*Relative risks for dietary vitamin E intake were calculated among nonusers of vitamin E supplements. There was a total of 393 cases of coronary heart disease after the exclusion of 17,916 men who reported current use of either multivitamins or vitamin E supplements in 1986. CI denotes confidence interval.

†Quintiles of dietary vitamin E intake were derived from the entire cohort.

‡Relative risks were derived by multivariate logistic-regression analysis as described in the notes to Table 1.

§Calculated after the exclusion of nonusers of supplements.

Table 2. Base-Line Characteristics, According to Quintile Group for Total Vitamin E Intake, among 39,910 Men Free of Heart Disease, Diabetes, and Hypercholesterolemia in 1986.*

Variable	Quintile Group for Vitamin E Intake 1	2	3	4	5
**Risk factors**					
Age (yr)	53.6	53.6	53.7	53.6	53.7
Body-mass index	25.7	25.5	25.5	25.3	25.1
Current smoker (%)	13.8	9.8	8.2	10.0	9.2
Hypertension (%)	18.9	17.5	17.3	19.1	17.2
Aspirin use (%)†	22	23	24	30	31
Exercise (%)‡	16	21	23	24	27
**Mean dietary intake**					
Total fat (g/day)	61.3	72.4	82.8	74.6	68.4
Calories (kcal/day)	1713	1976	2216	2031	1944
Calories from fat (%)	32.1	32.8	33.5	32.8	31.5
Dietary fiber (g/day)	15.8	21.3	26.4	23.4	23.7
Cholesterol (mg/day)	302	336	357	330	322
Alcohol (g/day)	13.3	11.5	10.9	11.8	11.8

*Standardized for age to the total cohort of 39,910 men free of disease at base line.

†Indicates regular use more than twice per week.

‡Moderate-to-heavy exercise at least 90 minutes per week.

The New England Journal of Medicine
Downloaded from nejm.org at NORTHEASTERN UNIVERSITY on November 18, 2017. For personal use only. No other uses without permission.
Copyright © 1993 Massachusetts Medical Society. All rights reserved.

Table 4. Relative Risk of Coronary Heart Disease, According to the Duration of Current Supplement Use among Participants in the Study.*

Variable†	Years of Supplement Use					P Value for Trend‡
	no use	0–1	2–4	5–9	≥10	
**Vitamin E**						
Coronary disease — no. of cases	556	11	19	32	35	
No. of person-years	113,819	2296	5977	7,572	8,162	
Relative risk	1.0	0.95	0.59	0.78	0.65	0.10
95% CI	—	0.52–1.75	0.37–0.93	0.54–1.12	0.46–0.92	
**Multivitamins**						
Coronary disease — no. of cases	418	18	28	50	102	
No. of person-years	81,872	2995	8894	11,807	22,092	
Relative risk	1.0	1.38	0.69	0.88	0.75	0.23
95% CI	—	0.85–2.24	0.47–1.02	0.66–1.19	0.60–0.94	
**Vitamin C**						
Coronary disease — no. of cases	527	10	20	38	59	
No. of person-years	106,886	1729	6499	9,635	12,221	
Relative risk	1.0	1.19	0.67	0.83	0.80	0.94
95% CI	—	0.63–2.26	0.42–1.05	0.59–1.16	0.60–1.05	

*Relative risks were derived by multivariate logistic-regression analysis as described in the notes to Table 1.

†Numbers of cases do not total 667 because of missing information about the duration of supplement use. CI denotes confidence interval.

‡Tests for trend were calculated after the exclusion of nonusers of vitamins.

The relative risk of coronary disease in men taking 100 or more IU per day of vitamin E for two or more years, as compared with nonusers of supplements, ranged from 0.57 to 0.67 in multivariate analyses after we controlled separately for quintile groups for the intake (from dietary sources and supplements combined) of retinol; vitamins B_1, B_2, B_6, B_{12}, C, and D; and calcium, folate, niacin, iron, magnesium, and zinc. Whereas the relative risks associated with vitamin E remained significant in each model, those comparing the highest with the lowest quintile groups for the other nutrients were not statistically significant.

The reduction in the risk of coronary disease associated with the highest quintile group for total vitamin E intake was somewhat less among current smokers (relative risk, 0.67; 95 percent confidence interval, 0.34 to 1.31) than among those who had never smoked (relative risk, 0.52; 95 percent confidence interval, 0.34 to 0.78). The inverse association was not appreciably modified by age, family history of myocardial infarction, or dietary intake of alcohol, carotene, vitamin C, polyunsaturated fat, total fat, magnesium, or iron.

In our analysis of overall mortality (in 578 men), the relative risk was 0.78 (95 percent confidence interval, 0.60 to 1.01; P for trend = 0.06) when we compared the highest and lowest quintile groups for vitamin E intake. Mortality during the first four years of follow-up of a healthy population is skewed toward death from sudden causes, such as trauma, and diseases with a delayed time to death are underrepresented.

The association between dietary carotene and the risk of coronary disease differed significantly according to base-line smoking status (Table 5). Among men who had never smoked, no association was observed. We did, however, find a significant inverse association between carotene intake and coronary disease among current and former smokers. Among current smokers, the relative risk was 0.30 (95 percent confidence interval, 0.11 to 0.82) when the highest and lowest quintile groups for intake were compared; among former smokers, the risk was 0.60 (95 percent confidence interval, 0.38 to 0.94). The reduction in risk among current smokers was significant even in the third quintile group for dietary carotene intake (whose intake was equivalent to half a carrot per day).

Discussion

These data are compatible with the hypothesis that an increased intake of antioxidants, primarily as vitamin E, but also as dietary carotene in former and current smokers, is associated with a reduced risk of coronary disease. Although we cannot exclude the possibility that an unknown variable associated with high antioxidant intake is responsible for the reduction in coronary disease, the lack of a significant association of coronary disease with other micronutrients (e.g., vitamin C) consumed as either diet or supplements suggests a causal interpretation.

The high rate of follow-up reduced potential bias due to loss to follow-up, and because dietary information was collected prospectively, we eliminated recall bias. To reduce the possibility of bias associated with recent dietary changes related to disease, we excluded men with coronary disease, diabetes, or high cholesterol levels at base line. The finding that the apparent benefit of vitamin E supplements was stronger with longer-term use is further evidence of a specific effect rather than simply self-selection.

Men with a higher intake of vitamin E have somewhat healthier risk profiles (Table 2). After we controlled for this health-conscious behavior (Table 1), however, a strong protective association with vitamin E intake persisted. Any remaining residual confounding would need to be large to explain the relative risk we observed. Serum lipids were not measured in the total cohort and could potentially be confounding variables. In a subsample of participants,[16] however, vitamin E intake was not correlated with either total or high-density lipoprotein cholesterol ($r = -0.02$ for both). In studies of vitamin E supplementation, vitamin E did not alter blood lipid levels.[24,25] Hence, confounding according to lipid level is an unlikely explanation of our results. If uncontrolled confounding explains the inverse association for vitamin E, we would expect a similar effect for vitamin C, which was

The New England Journal of Medicine
Downloaded from nejm.org at NORTHEASTERN UNIVERSITY on November 18, 2017. For personal use only. No other uses without permission.
Copyright © 1993 Massachusetts Medical Society. All rights reserved.

Table 5. Multivariate Relative Risk of Coronary Heart Disease, According to Quintile Group for Dietary Carotene Intake and Smoking Status.*

Variable	Quintile Group 1	2	3	4	5	P Value for Trend
Carotene intake (IU)	<5030	5030–7019	7020–9592	9593–14,387	≥14,388	
**Men who never smoked** (n = 18,551)						
Coronary disease — no. of cases	33	49	56	48	58	
Relative risk	1.0	1.28	1.37	1.07	1.09	0.64
95% CI	—	0.81–2.01	0.86–2.16	0.66–1.74	0.66–1.79	
**Former smokers** (n = 15,946)						
Coronary disease — no. of cases	54	53	71	66	49	
Relative risk	1.0	0.76	0.89	0.82	0.60	0.04
95% CI	—	0.52–1.13	0.61–1.30	0.55–1.22	0.38–0.94	
**Current smokers** (n = 3923)						
Coronary disease — no. of cases	33	25	14	18	6	
Relative risk	1.0	0.72	0.46	0.65	0.30	0.02
95% CI	—	0.41–1.25	0.23–0.91	0.33–1.27	0.11–0.82	

*Relative risks were derived by multivariate logistic-regression analysis as described in the notes to Table 1. Thirty-four cases for which information on smoking was missing were excluded. CI denotes confidence interval.

similarly associated with a healthier lifestyle (data not shown). After adjustment for vitamin E, however, vitamin C was not associated with a reduction in coronary disease (Table 1), a result that supported a specific effect of vitamin E.

Our findings for vitamin E are consistent with geographic correlations between serum vitamin E levels and coronary mortality rates[8] and reduced serum levels of alpha-tocopherol (the principal component of vitamin E) in patients with angina.[26] Most notably, the associations we found for vitamin E supplements were remarkably similar to those found among women in the Nurses' Health Study.[27] Vitamin E appeared to reduce atherosclerosis in a small experiment in monkeys[28] and to decrease the rate of restenosis among patients after angioplasty.[29] Little difference in vitamin E levels was seen, however, between persons with and persons without myocardial infarction when measurements were made in previously collected and stored serum samples.[30-32] These studies may have failed to find differences in serum alpha-tocopherol levels for several reasons: because few participants used supplements, because a single measurement cannot distinguish between short- and long-term supplement use, and because the degradation of alpha-tocopherol in stored samples[4] reduces the overall variation. Alternatively, intracellular antioxidant levels may be more important than serum levels in inhibiting the cell-mediated oxidation of LDL.[33]

In addition to the strong association with vitamin E supplements, we found a moderate reduction in the risk of coronary disease with increasing intake of nonsupplemental dietary vitamin E. The primary sources of dietary vitamin E are vegetable oils and to a lesser extent seeds, cereal grains, and nuts. Although we incorporated information on specific brands of margarines and cooking oils into the dietary intake scores, the vitamin E content of foods is dependent on cooking, processing, and storage.[34] Limited variability in dietary vitamin E intake and error in measurement due to the instability of vitamin E may have obscured a stronger inverse association. In a subsample of 121 men in our study, we found only a weak partial correlation (r = 0.11) between dietary vitamin E and serum alpha-tocopherol levels, which was greatly enhanced (r = 0.51) after the inclusion of vitamin E from supplements.[1] The lack of a strong association between dietary vitamin E alone and coronary disease is consistent with experimental evidence in which the resistance of LDL to oxidation[35-38] is increased only by supplemental vitamin E intake at levels 10 to 100 times the standard recommended dietary allowance.[39] Supplementation at this level has been shown to be nontoxic over a moderate follow-up period.[40]

With regard to carotene, in a cohort of 1299 elderly Massachusetts residents followed for 4.75 years, Gaziano et al. found a relative risk of 0.55 (95 percent confidence interval, 0.34 to 0.87) for death from cardiovascular causes when the highest and the lowest quartile groups for intake were compared.[10] In a preliminary report from the ongoing Physicians' Health Study trial, men with angina who were randomly assigned to receive beta carotene had fewer subsequent cardiovascular events than those assigned to placebo.[41]

We and others have reported lower levels of plasma carotene (but not alpha-tocopherol)[1,2,26,35,42] among smokers. The increased oxidative stress brought on by smoking may increase the susceptibility of lipids to oxidation[43-46] and increase the demand for plasma antioxidants to quench oxygen free radicals.

Vitamin C preserves the endogenous antioxidants[47] and quenches oxidants in hydrophilic environments.[48] Much of the oxidation of LDL particles, however, occurs in the subendothelial space, a hydrophobic environment that favors a protective effect of fat-soluble vitamins (vitamin E or carotene) over water-soluble vitamins (vitamin C).[49]

Cross-cultural data suggest that men in countries with higher rates of mortality from coronary causes have, on average, plasma vitamin C levels that border on being deficient.[8] Because the median of the lowest quintile group for dietary vitamin C intake was 78 mg per day in our study (recommended daily allowance, 60 mg),[39] we could not test this hypothesis.

In the follow-up of the cohort in the first National

The New England Journal of Medicine
Downloaded from nejm.org at NORTHEASTERN UNIVERSITY on November 18, 2017. For personal use only. No other uses without permission.
Copyright © 1993 Massachusetts Medical Society. All rights reserved.

Health and Nutrition Examination Survey,[9] men reporting an intake of 50 mg or more of vitamin C per day (including supplements) had a standardized mortality ratio of 0.58 for all cardiovascular disease. However, vitamin C from diet alone was not appreciably related to cardiovascular disease. Hence, the effect appears to be explained by the use of vitamin supplements, and perhaps not specifically those of vitamin C, because the authors did not account for vitamin E use.

We found an inverse association between vitamin E intake and coronary disease that was weak at best when only dietary sources were taken into account. At the higher levels of intake reached with supplementation, the association became significant. We cannot rule out the possibility that confounding may partly account for our results; a cause-and-effect relation cannot be established from these observational data. However, these findings, together with similar findings in women,[27] support the hypothesis that supplemental vitamin E may reduce the risk of coronary disease. Public policy recommendations about the use of vitamin E supplements should await the results of additional studies.

We are indebted to the participants of the Health Professionals Follow-up Study for their continued cooperation and participation; to Al Wing, Karen Corsano, Mira Koyfman, and Steve Stuart for computer assistance; and to Mary Johnson, Betsy Frost-Hawes, Kerry Pillsworth, Mitzi Wolff, Jan Vomacka, and Cindy Dyer for their assistance in the compilation of the data and the preparation of the manuscript.

References

1. Ascherio A, Stampfer MJ, Colditz GA, Rimm EB, Litin L, Willett WC. Correlations of vitamin A and E intakes with the plasma concentrations of carotenoids and tocopherols among American men and women. J Nutr 1992;122:1792-801.
2. Stryker WS, Kaplan LA, Stein EA, Stampfer MJ, Sober A, Willett WC. The relation of diet, cigarette smoking, and alcohol consumption to plasma beta-carotene and alpha-tocopherol levels. Am J Epidemiol 1988;127:283-96.
3. Steinberg D, Witztum JL. Lipoproteins and atherogenesis: current concepts. JAMA 1990;264:3047-52.
4. Diplock AT. Antioxidant nutrients and disease prevention: an overview. Am J Clin Nutr 1991;53:Suppl:189S-193S.
5. Quinn MT, Parthasarathy S, Steinberg D. Endothelial cell-derived chemotactic activity for mouse peritoneal macrophages and the effects of modified forms of low density lipoprotein. Proc Natl Acad Sci U S A 1985;82:5949-53.
6. Quinn MT, Parthasarathy S, Fong LG, Steinberg D. Oxidatively modified low density lipoproteins: a potential role in recruitment and retention of monocyte/macrophages during atherogenesis. Proc Natl Acad Sci U S A 1987;84:2995-8.
7. Hessler JR, Robertson AL Jr, Chisolm GM III. LDL-induced cytotoxicity and its inhibition by HDL in human vascular smooth muscle and endothelial cells in culture. Atherosclerosis 1979;32:213-29.
8. Gey KF, Brubacher GB, Stähelin HB. Plasma levels of antioxidant vitamins in relation to ischemic heart disease and cancer. Am J Clin Nutr 1987;45: Suppl:1368-77.
9. Enstrom JE, Kanim LE, Klein MA. Vitamin C intake and mortality among a sample of the United States population. Epidemiology 1992;3:194-202.
10. Gaziano JM, Manson JE, Branch LG, et al. A prospective study of β-carotene in fruits and vegetables and decreased cardiovascular mortality in the elderly. Am J Epidemiol 1992;136:985. abstract.
11. Grobbee DE, Rimm EB, Giovannucci E, Colditz G, Stampfer MJ, Willett WC. Coffee, caffeine, and cardiovascular disease in men. N Engl J Med 1990;323:1026-32.
12. Rimm EB, Giovannucci EL, Willett WC, et al. Prospective study of alcohol consumption and risk of coronary disease in men. Lancet 1991;338:464-8.
13. Rimm EB, Stampfer MJ, Colditz GA, Giovannucci E, Willett WC. Effectiveness of various mailing strategies among nonrespondents in a prospective cohort study. Am J Epidemiol 1990;131:1068-71.
14. Composition of foods. Handbook 8 series. Washington, D.C.: Department of Agriculture, 1976-1989.
15. Willett WC, Sampson L, Stampfer MJ, et al. Reproducibility and validity of a semiquantitative food frequency questionnaire. Am J Epidemiol 1985; 122:51-65.
16. Rimm EB, Giovannucci EL, Stampfer MJ, Colditz GA, Litin LB, Willett WC. Reproducibility and validity of an expanded self-administered semi-quantitative food frequency questionnaire among male health professionals. Am J Epidemiol 1992;135:1114-26.
17. Giovannucci E, Colditz GA, Stampfer MJ, et al. The assessment of alcohol consumption by a simple self-administered questionnaire. Am J Epidemiol 1991;133:810-7.
18. Hunter DJ, Rimm EB, Sacks FM, et al. Comparison of measures of fatty acid intake by subcutaneous fat aspirate, food frequency questionnaire, and diet records in a free-living population of US men. Am J Epidemiol 1992;135:418-27.
19. Willett WC, Stampfer MJ. Total energy intake: implications for epidemiologic analyses. Am J Epidemiol 1986;124:17-27.
20. Beaton GH, Milner J, Corey P, et al. Sources of variance in 24-hour dietary recall data: implications for nutrition study design and interpretation. Am J Clin Nutr 1979;32:2546-59.
21. Rose GA, Blackburn H. Cardiovascular survey methods. 2nd ed. World Health Organization monograph series no. 58. Geneva: World Health Organization, 1982.
22. Rothman KJ. Modern epidemiology. Boston: Little, Brown, 1986.
23. Mantel N. Chi-square tests with one degree of freedom; extensions of the Mantel-Haenszel procedure. J Am Stat Assoc 1963;58:690-700.
24. Stampfer MJ, Willett WC, Castelli WP, Taylor JO, Fine J, Hennekens CH. The effect of vitamin E on lipids. Am J Clin Pathol 1983;79:714-6.
25. Kalbfleisch JH, Barboriak JJ, Else BA, Hughes CV, Tristani FE. α-Tocopherol supplements and high-density-lipoprotein-cholesterol levels. Br J Nutr 1986;55:71-7.
26. Riemersma RA, Wood DA, Macintyre CCA, Elton RA, Gey KF, Oliver MF. Risk of angina pectoris and plasma concentrations of vitamins A, C, and E and carotene. Lancet 1991;337:1-5.
27. Stampfer MJ, Hennekens CH, Manson JE, Colditz GA, Rosner B, Willett WC. Vitamin E consumption and the risk of coronary disease in women. N Engl J Med 1993;328:1444-9.
28. Verlangieri AJ, Bush MJ. Effects of d-α-tocopherol supplementation on experimentally induced primate atherosclerosis. J Am Coll Nutr 1992;11: 131-8.
29. DeMaio SJ, King SB III, Lembo NJ, et al. Vitamin E supplementation, plasma lipids and incidence of restenosis after percutaneous transluminal coronary angioplasty (PTCA). J Am Coll Nutr 1992;11:68-73.
30. Street DA, Comstock GW, Salkeld RM, Schüep W, Klag M. A population-based case-control study of the association of serum antioxidants and myocardial infarction. Am J Epidemiol 1991;134:719-20. abstract.
31. Kok FJ, de Bruijn AM, Vermeeren R, et al. Serum selenium, vitamin antioxidants, and cardiovascular mortality: a 9-year follow-up study in the Netherlands. Am J Clin Nutr 1987;45:462-8.
32. Salonen JT, Salonen R, Penttila I, et al. Serum fatty acids, apolipoproteins, selenium and vitamin antioxidants and the risk of death from coronary artery disease. Am J Cardiol 1985;56:226-31.
33. Parthasarathy S. Evidence for an additional intracellular site of action of probucol in the prevention of oxidative modification of low density lipoprotein: use of a new water-soluble probucol derivative. J Clin Invest 1992;89:1618-21.
34. Vitamin E: a scientific status summary by the Institute of Food Technologists' expert panel on food safety & nutrition and the Committee on Public Information. Nutr Rev 1977;35:57-62.
35. Princen HMG, van Poppel G, Vogelezang C, Buytenhek R, Kok FJ. Supplementation with vitamin E but not β-carotene in vivo protects low density lipoprotein from lipid peroxidation in vitro: effect of cigarette smoking. Arterioscler Thromb 1992;12:554-62.
36. Jialal I, Grundy SM. Effect of dietary supplementation with alpha-tocopherol on the oxidative modification of low density lipoprotein. J Lipid Res 1992;33:899-906.
37. Dieber-Rotheneder M, Puhl M, Waeg G, Striegl G, Esterbauer H. Effect of oral supplementation with D-alpha-tocopherol on the vitamin E content of human low density lipoproteins and resistance to oxidation. J Lipid Res 1991;32:1325-32.
38. Esterbauer H, Dieber-Rotheneder M, Striegl G, Waeg G. Role of vitamin E in preventing the oxidation of low-density lipoprotein. Am J Clin Nutr 1991;53:Suppl:314S-321S.
39. National Research Council. Recommended dietary allowances. Washington, D.C.: National Academy Press, 1989.
40. Bendich A, Machlin LJ. Safety of oral intake of vitamin E. Am J Clin Nutr 1988;48:612-9.

The New England Journal of Medicine
Downloaded from nejm.org at NORTHEASTERN UNIVERSITY on November 18, 2017. For personal use only. No other uses without permission.
Copyright © 1993 Massachusetts Medical Society. All rights reserved.

41. Gaziano JM, Manson JE, Ridker PM, Buring JE, Hennekens CH. Beta carotene therapy for chronic stable angina. Circulation 1990;82:Suppl III:III-201. abstract.
42. Nierenberg DW, Stukel TA, Baron JA, Dain BJ, Greenberg ER. Determinants of plasma levels of beta-carotene and retinol: Skin Cancer Prevention Study Group. Am J Epidemiol 1989;130:511-21.
43. Harats D, Ben-Naim M, Dabach Y, Hollander G, Stein O, Stein Y. Cigarette smoking renders LDL susceptible to peroxidative modification and enhanced metabolism by macrophages. Atherosclerosis 1989;79:245-52.
44. Church DF, Pryor WA. Free radical chemistry of cigarette smoke and its toxicological implications. Environ Health Perspect 1985;64:111-26.
45. Duthie GG, Arthur JR, James WPT. Effects of smoking and vitamin E on blood antioxidant status. Am J Clin Nutr 1991;53:Suppl:1061S-1063S.
46. Hoshino E, Shariff R, Van Gossum A, et al. Vitamin E suppresses increased lipid peroxidation in cigarette smokers. JPEN J Parenter Enteral Nutr 1990;14:300-5.
47. Jialal I, Grundy SM. Preservation of the endogenous antioxidants in low density lipoprotein by ascorbate but not probucol during oxidative modification. J Clin Invest 1991;87:597-601.
48. Stocker R, Frei B. Endogenous antioxidant defenses in human blood plasma. In: Sies H, ed. Oxidative stress: oxidants and antioxidants. Orlando, Fla.: Academic Press, 1991:213-43.
49. Niki E, Yamamoto Y, Komuro E, Sato K. Membrane damage due to lipid oxidation. Am J Clin Nutr 1991;53:Suppl:201S-205S.

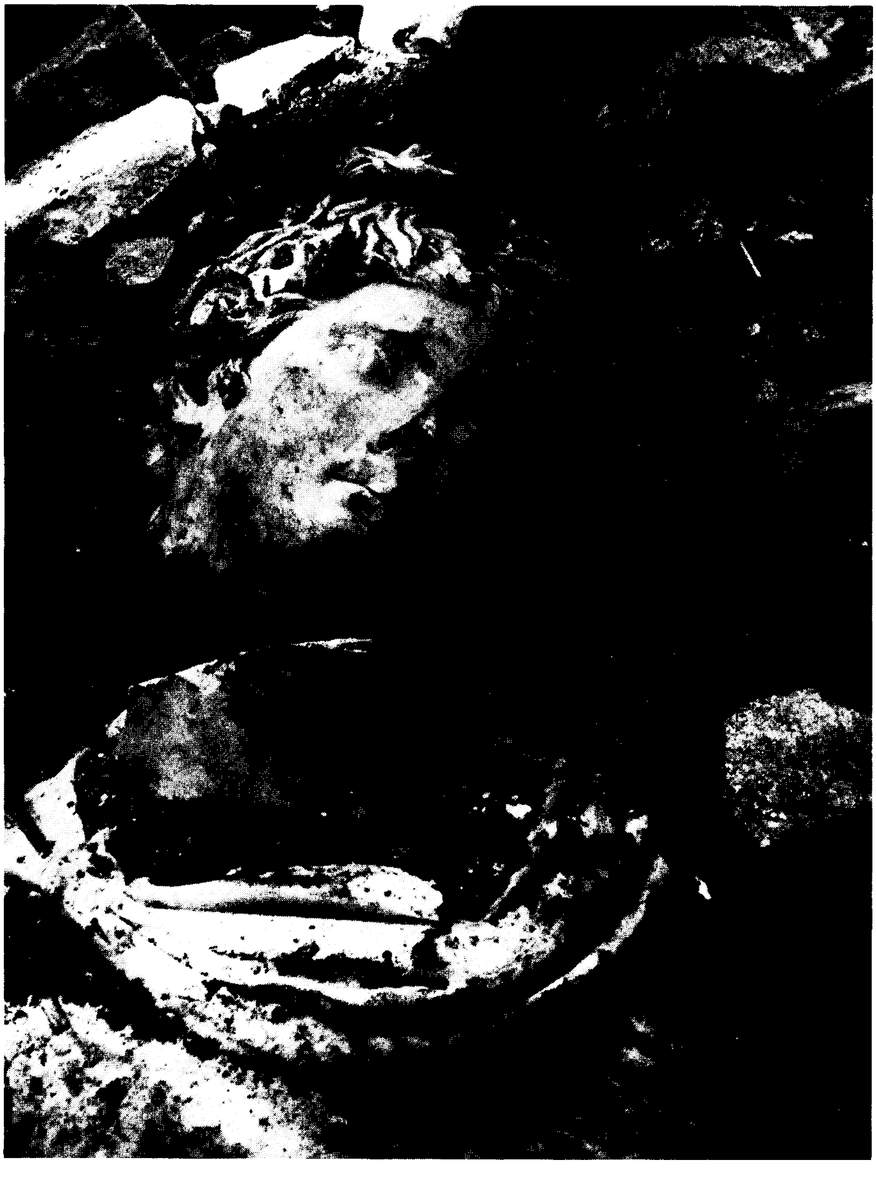

Fallen Goddess MICHAEL DIMAIO, M.D.

The New England Journal of Medicine
Downloaded from nejm.org at NORTHEASTERN UNIVERSITY on November 18, 2017. For personal use only. No other uses without permission.
Copyright © 1993 Massachusetts Medical Society. All rights reserved.
