# Supplementary material for: A systematic comprehensive longitudinal evaluation of dietary factors associated with acute myocardial infarction and fatal coronary heart disease
Source: Nat Commun. 2020 Nov 27;11:6074. doi: 10.1038/s41467-020-19888-2 (PMC7699643; doi:10.1038/s41467-020-19888-2)
Supplement: Supplementary file 4 — Supplementary Software [file 41467_2020_19888_MOESM4_ESM.zip › EWAS-NHS-master/KG-SI/Rest/Siri-Tarino (2010).pdf]

See corresponding editorial on page 497.

Meta-analysis of prospective cohort studies evaluating the association of saturated fat with cardiovascular disease[1–5]

Patty W Siri-Tarino, Qi Sun, Frank B Hu, and Ronald M Krauss

ABSTRACT

Background: A reduction in dietary saturated fat has generally been thought to improve cardiovascular health.

Objective: The objective of this meta-analysis was to summarize the evidence related to the association of dietary saturated fat with risk of coronary heart disease (CHD), stroke, and cardiovascular disease (CVD; CHD inclusive of stroke) in prospective epidemiologic studies.

Design: Twenty-one studies identified by searching MEDLINE and EMBASE databases and secondary referencing qualified for inclusion in this study. A random-effects model was used to derive composite relative risk estimates for CHD, stroke, and CVD.

Results: During 5–23 y of follow-up of 347,747 subjects, 11,006 developed CHD or stroke. Intake of saturated fat was not associated with an increased risk of CHD, stroke, or CVD. The pooled relative risk estimates that compared extreme quantiles of saturated fat intake were 1.07 (95% CI: 0.96, 1.19; $P = 0.22$) for CHD, 0.81 (95% CI: 0.62, 1.05; $P = 0.11$) for stroke, and 1.00 (95% CI: 0.89, 1.11; $P = 0.95$) for CVD. Consideration of age, sex, and study quality did not change the results.

Conclusions: A meta-analysis of prospective epidemiologic studies showed that there is no significant evidence for concluding that dietary saturated fat is associated with an increased risk of CHD or CVD. More data are needed to elucidate whether CVD risks are likely to be influenced by the specific nutrients used to replace saturated fat. *Am J Clin Nutr* 2010;91:535–46.

INTRODUCTION

Early animal studies showed that high dietary saturated fat and cholesterol intakes led to increased plasma cholesterol concentrations as well as atherosclerotic lesions (1). These findings were supported by associations in humans in which dietary saturated fat correlated with coronary heart disease (CHD) risk (2, 3). More recent epidemiologic studies have shown positive (4–10), inverse (11, 12), or no (4, 13–18) associations of dietary saturated fat with CVD morbidity and/or mortality.

A limited number of randomized clinical interventions have been conducted that have evaluated the effects of saturated fat on risk of CVD. Whereas some studies have shown beneficial effects of reduced dietary saturated fat (19–21), others have shown no effects of such diets on CVD risk (22, 23). The studies that showed beneficial effects of diets reduced in saturated fat replaced saturated fat with polyunsaturated fat, with the implication that the CVD benefit observed could have been due to an increase in polyunsaturated fat or in the ratio of polyunsaturated fat to saturated fat (P:S), a hypothesis supported by a recent pooling analysis conducted by Jakobsen et al (24).

The goal of this study was to conduct a meta-analysis of well-designed prospective epidemiologic studies to estimate the risk of CHD and stroke and a composite risk score for both CHD and stroke, or total cardiovascular disease (CVD), that was associated with increased dietary intakes of saturated fat. Large prospective cohort studies can provide statistical power to adjust for covariates, thereby enabling the evaluation of the effects of a specific nutrient on disease risk. However, such studies have caveats, including a reliance on nutritional assessment methods whose validity and reliability may vary (25), the assumption that diets remain similar over the long term (26) and variable adjustment for covariates by different investigators. Nonetheless, a summary evaluation of the epidemiologic evidence to date provides important information as to the basis for relating dietary saturated fat to CVD risk.

SUBJECTS AND METHODS

Study selection

Two investigators (QS and PS-T) independently conducted a systematic literature search of the MEDLINE (http://www.ncbi.nlm.nih.gov/pubmed/) and EMBASE (http://www.embase.com) databases through 17 September 2009 by using the following search terms: ("saturated fat" or "dietary fat") and ("coronary" or "cardiovascular" or "stroke") and ("cohort" or "follow up").

[1] From the Children's Hospital Oakland Research Institute, Oakland, CA (PWS-T and RMK), and the Departments of Nutrition (QS and FBH) and Epidemiology (FBH), Harvard School of Public Health, Boston, MA.

[2] PWS-T and QS contributed equally to this work.

[3] The contents of this article are solely the responsibility of the authors and do not necessarily represent the official view of the National Center for Research Resources (http://www.ncrr.nih.gov) or the National Institutes of Health.

[4] Supported by the National Dairy Council (PWS-T and RMK) and made possible by grant UL1 RR024131-01 from the National Center for Research Resources, a component of the National Institutes of Health (NIH), and NIH Roadmap for Medical Research (PWS-T and RMK). QS was supported by a Postdoctoral Fellowship from Unilever Corporate Research. FBH was supported by NIH grant HL60712.

[5] Address correspondence to RM Krauss, Children's Hospital Oakland Research Institute, 5700 Martin Luther King Junior Way, Oakland, CA 94609. E-mail: rkrauss@chori.org.

Received March 6, 2009. Accepted for publication November 25, 2009.
First published online January 13, 2010; doi: 10.3945/ajcn.2009.27725.

Studies were eligible if *1*) data related to dietary consumption of saturated fat were available; *2*) the endpoints were nonfatal or fatal CVD events, but not CVD risk factors; *3*) the association of saturated fat with CVD was specifically evaluated; *4*) the study design was a prospective cohort study; and *5*) study participants were generally healthy adults at study baseline. The initial search yielded 661 unique citations, of which 19 studies met the inclusion criteria and were selected as appropriate for inclusion in this meta-analysis (**Figure 1**) (4–6, 8–11, 14–16, 18, 27–34). All 4 investigators participated in the selection process (PS-T, QS, FBH, and RMK). Additional reference searches were performed by conducting a hand review of references from retrieved articles, and 3 more studies were identified (13, 17, 35). Of the 22 identified studies, 10 studies provided data appropriate to the constraints of this meta-analysis, specifically, relative risk (RR) estimates for CVD as a function of saturated fat intake (10, 14–16, 28, 30–34). Where such data were not provided ($n = 12$), data requests were made to investigators. Investigators from 6 of the studies (4, 5, 8, 18, 29, 35) responded with the requested data. A data set of the Honolulu Heart Study (9), obtained from the National Heart, Lung, and Blood Institute (36), was used to derive the RR estimates for this study. Investigators from 5 studies either did not respond or could no longer access data sets (6, 11, 13, 17, 27). However, 4 of 5 of these studies provided data for saturated fat intake as a continuous variable (6, 11, 13, 17). For these studies, we derived RR estimates (*see* Statistical analysis) for categorical saturated fat intake and CHD and/or stroke. The study by Boden-Albala et al (27) was excluded because the RR estimate published did not correspond to the values given for the upper and lower bounds of the CI, ie, using these values to derive the SE resulted in different estimates, and the authors did not respond to our attempts to obtain the correct data.

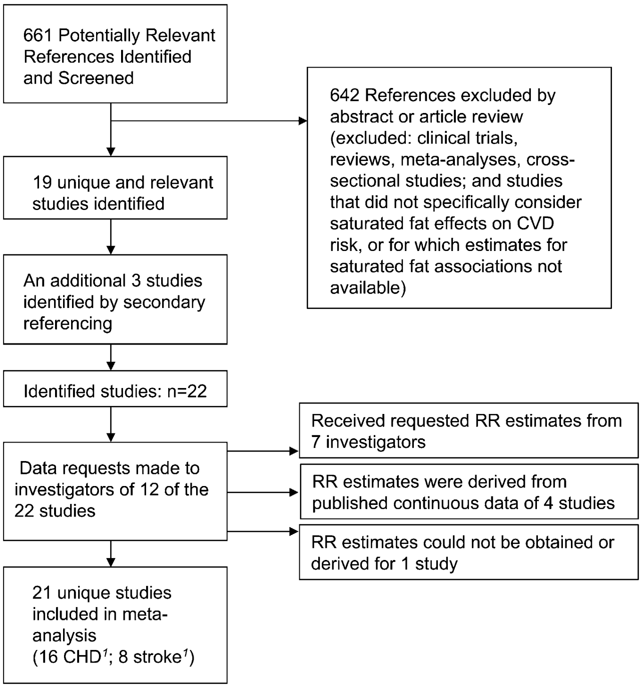

FIGURE 1. Study selection process. CHD, coronary heart disease; CVD, cardiovascular disease; RR, relative risk. [1]Three studies provided outcome data for both CHD and stroke.

Altogether, this meta-analysis included data from 21 unique studies, with 16 studies providing risk estimates for CHD and 8 studies providing data for stroke as an endpoint. Data were derived from 347,747 participants, of whom 11,006 developed CVD.

Data extraction

Two authors (PS-T and QS) independently extracted and tabulated data from each study using a standard extraction form. Discrepancies were resolved via review of the original articles and group discussion. From each study, we extracted information on first author, publication year, disease outcome, country of origin, method of outcome ascertainment, sample size, age, sex, average study follow-up time, number of cases, dietary assessment method and the validity of the method, number of dietary assessments, covariates adjusted, unit of measurement, RR of CVD comparing extreme quantiles of saturated fat intake or per unit of saturated fat intake, and corresponding 95% CIs, SEs, or exact P values.

Statistical analysis

RRs and 95% CIs were log transformed to derive corresponding SEs for β-coefficients by using Greenland's formula (37). Otherwise, we used exact P values to derive SEs where possible. To minimize the possibility that the association for saturated fat intake may be influenced by extreme values, we contacted the authors of studies in which RRs were presented as per-unit increments of saturated fat intake and requested RRs comparing extreme quantiles. For studies for which we did not receive responses, we used the published trend RRs comparing the 25th and 75th percentiles to estimate RRs comparing high with low dichotomized saturated fat intakes (38).

Meta-analyses were performed by using STATA 10.0 (StataCorp, College Station TX) and Review Manager 5.0 (The Nordic Cochrane Centre, The Cochrane Collaboration, Copenhagen, Denmark; http://www.cc-ims.net/RevMan). $P < 0.05$ was considered statistically significant. Random-effects models taking into account both within-study and between-study variability were used to estimate pooled RRs for associations of dietary saturated fat with CHD risk. Relative to fixed-effects models, random-effects models were more appropriate for the current study because test statistics showed evidence of heterogeneity among these studies. When several RRs were given for subgroup analyses within a single study, random-effects models were used to pool the RRs into one composite estimate.

We used the STATA METAINF module to examine the influence of an individual study on the pooled estimate of RR by excluding each study in turn. We used the STATA METAREG module to examine whether the effect size of these studies depended on certain characteristics of each study, including age, sex, sample size, duration of follow-up, whether disease outcomes were confirmed by medical record review, and a score evaluating overall study quality. This quality score was derived from the following information: dietary assessment method (where 5 points were given for diet records, 4 for validated FFQs, 3 for FFQs that were not formally validated, 2 for diet history, and 1 for 24-h recall), number of dietary assessments, and number of adjusted established risk factors for CVD. Points were totaled to construct a composite quality score for each study.

We further conducted secondary analyses to examine age- and sex-specific effects (ie, age $<$60 y compared with $\geq$60 y and

TABLE 1
Study design characteristics of 21 unique prospective epidemiologic studies of saturated fat intake and risk of coronary heart disease (CHD) or stroke[1]

Study	Disease outcome	Dietary assessment method	FFQ items	Validation of nutrients	Correlation with diet record[2]	Validation in study population	Multiple assessment of diet	Assessment interval	Average duration of follow-up
								y	y
CHD									
Shekelle et al, 1981 (17)	Fatal CHD	Interview (28 d)	195	Not validated	—	—	Baseline	—	20
McGee et al, 1984 (9)[3]	Total CHD	24-h recall (1 d)	—	NA	—	—	Baseline	—	10
Kushi et al, 1985 (13)	Fatal CHD	Diet-history (time frame unknown)	—	Not validated	—	—	Baseline	—	20
Posner et al, 1991 (16)	Total CHD	24-h recall (1 d)	—	NA	—	—	Baseline	—	16
Fehily et al, 1993 (28)	Total CHD	7-d diet records	—	NA	—	—	Baseline	—	5
Goldbourt et al, 1993 (35)[3]	Fatal CHD	FFQ (time frame unknown)	NR	Not validated	—	—	Baseline	—	23
Ascherio et al, 1996 (4)	Total CHD	FFQ (1 y)	131	Validated	0.75	Yes	Twice	4	14
Esrey et al, 1996 (6)	Fatal CHD	24-h recall (1 d)	—	NA	—	—	Baseline	—	12
Mann et al, 1997 (32)	Fatal CHD	FFQ (time interval unknown)	NR	Not validated	—	—	Baseline	—	13
Pietinen et al, 1997 (15)	Total CHD	FFQ (1 y)	276	Validated	0.70	Yes	Baseline	—	6
Boniface and Tefft, 2002 (5)	Fatal CHD	FFQ (time frame unknown)	30	Validated	0.34	Yes	Baseline	—	16
Jakobsen et al, 2004 (8)	Total CHD	7-d diet records	—	NA	—	—	Baseline	—	16
Leosdottir et al, 2007 (14)[3]	Fatal CHD	FFQ (1 y) and 7-d menu-diary	168	Validated	0.50	Yes	Baseline	—	8
Oh et al, 2005 (33)	Total CHD	FFQ (1 y)	116	Validated	0.68	Yes	6 times	2–4	20
Tucker et al, 2005 (18)	Fatal CHD	7-d diet records	—	NA	—	—	4 times	3–9	18
Xu et al, 2006 (10)	Total CHD	24-h recall (1 d)	—	NA	—	—	Baseline	—	7
Stroke									
McGee et al, 1984 (9)[3]	Total stroke	24-h recall (1 d)	—	NA	—	—	Baseline	—	10
Goldbourt et al, 1993 (35)[3]	Fatal stroke	FFQ (time frame unknown)	NR	Not validated	—	—	Baseline	—	23
Gillman et al, 1997 (11)	Ischemic stroke	24-h recall (1 d)	—	NA	—	—	Baseline	—	20
Iso et al, 2001 (31)	Hemorrhagic stroke	FFQ (1 y)	116	Validated	0.68	Yes	4 times	2–4	14
He et al, 2003 (29)	Total stroke	FFQ (1 y)	131	Validated	0.75	Yes	3 times	4	14
Iso et al, 2003 (30)	Hemorrhagic stroke	24-h recall (1 d)	—	NA	—	—	Baseline	—	14
Sauvaget et al, 2004 (34)	Fatal stroke	1-d diet record	—	NA	—	—	Baseline	—	14
Leosdottir et al, 2007 (14)[3]	Ischemic stroke	FFQ (1 y) and 7-d menu-diary	168	Validated	0.50	Yes	Baseline	—	8

[1] FFQ, food-frequency questionnaire; NR, not reported; NA, not applicable.
[2] Only pertains to studies that used an FFQ to measure saturated fat intake.
[3] These studies provided both CHD and stroke outcome data.

TABLE 2
Baseline characteristics of participants of 21 unique prospective epidemiologic studies of saturated fat intake and risk of coronary heart disease (CHD) or stroke[1]

Study	No. of subjects	Age	Sex	Country of residence	Smoker	Disease outcome	Method of diagnosis
		y			%		
CHD							
Shekelle et al, 1981 (17)	1900	40–55	Male	USA	NR	Fatal CHD	DC
McGee et al, 1984 (9)[2]	8006	45–68	Male	USA	NR	Total CHD	MR and DC
Kushi et al,1985 (13)	1001	30–69	Male	USA	NR	Fatal CHD	DC
Posner et al, 1991 (16)	813	45–65	Male	USA	41.6	Total CHD	MR
Fehily et al, 1993 (28)	512	45–59	Male	UK	NR	Total CHD	DC
Goldbourt et al, 1993 (35)[2]	9767[3]	≥40	Male	Israel	NR	Fatal CHD	MR and DC
Ascherio et al, 1996 (4)	38,463	40–75	Male	USA	9.5	Total CHD	MR and DC
Esrey et al, 1996 (6)	4546	30–79	Both	Canada	33.9	Fatal CHD	MR
Mann et al, 1997 (32)	10,802	16–79	Both	UK	19.5	Fatal CHD	MR
Pietinen et al, 1997 (15)	21,930	50–69	Male	Finland	100	Total CHD	MR and DC
Boniface and Tefft, 2002 (5)	2676	40–75	Both	UK	NR	Total CHD	DC
Jakobsen et al, 2004 (8)	3686	30–71	Both	Denmark	NR	Total CHD	MR
Leosdottir et al, 2007 (14)[2]	28,098	45–73	Both	Sweden	29.5	Total CHD	DC
Oh et al, 2005 (33)	78,778	30–55	Female	USA	NR	Total CHD	MR and DC
Tucker et al, 2005 (18)	266[3]	34–80	Male	USA	21.8	Fatal CHD	MR and DC
Xu et al, 2006 (10)	2938	47–79	Both	USA	29.7	Total CHD	MR
Stroke							
McGee et al, 1984 (9)[2]	8006	45–68	Male	USA	NR	Total stroke	MR and DC
Goldbourt et al, 1993 (35)[2]	9767[3]	≥40	Male	Israel	NR	Fatal stroke	MR and DC
Gillman et al, 1997 (11)	832	45–65	Male	USA	NR	Ischemic stroke	MR
Iso et al, 2001 (31)	85,764	34–59	Female	USA	NR	Hemorrhagic stroke	MR and DC
He et al, 2003 (29)	38,463[3]	40–75	Male	USA	9.5	Total stroke	MR and DC
Iso et al, 2003 (30)	4775	40–69	Both	Japan	13.5	Hemorrhagic stroke	MR and DC
Sauvaget et al, 2004 (34)	3731	35–89	Both	Japan	26–35	Ischemic stroke	DC
Leosdottir et al, 2007 (14)[2]	28,098	45–73	Both	Sweden	29.5	Ischemic stroke	DC

[1] NR, not reported; MR, medical records; DC, death certificate.
[2] These studies provided both CHD and stroke outcome data.
[3] These numbers, as provided by the respective investigators, differ from those used in the original publications.

male compared with female) of saturated fat on CVD risk. These secondary analyses were performed with only those studies that provided stratified data according to these variables.

To examine the effects of replacing saturated fat with carbohydrate or polyunsaturated fat, we performed secondary meta-analyses with studies that provided pertinent and extractable data. Because energy from carbohydrate intake was excluded in fully adjusted models (including adjustments for total energy and energy from protein and fats other than saturated fat) in 6 of 21 studies, the regression coefficients of saturated fat could be interpreted as the effects of isocalorically replacing carbohydrate intake with saturated fat (25). Similarly, there were 5 studies (4, 9, 29, 31, 33) in which the regression coefficients of saturated fat could be interpreted as the effects of isocalorically replacing polyunsaturated fat intake with saturated fat. Finally, because consideration for total energy intake has been shown to be relevant in the evaluation of nutrient-disease associations (39), we also performed a subanalysis of studies (n = 15) that provided total energy intake data (4, 6, 8–11, 15, 16, 18, 29–31, 33). Begg funnel plots were used to assess potential publication bias (40).

RESULTS

The study design characteristics of the 21 studies identified by database searches and secondary referencing that were included in this meta-analysis (4–6, 8–11, 13–18, 28–35) are shown in **Table 1**. Altogether, there were 16 studies that considered the association of saturated fat with CHD and 8 studies that evaluated the association of saturated fat with stroke. Dietary assessments included 24-h recalls, food-frequency questionnaires (FFQs), and multiple daily food records. The duration of follow-up ranged from 6 to 23 y, with a mean and median follow-up of 14.3 and 14 y, respectively.

The baseline characteristics of the study participants are provided in **Table 2**. The number of subjects in each study ranged from 266 to 85,764. The age of participants ranged from ≈30 to 89 y. There were 11 studies conducted exclusively in men, 2 studies conducted exclusively in women, and 8 studies that enrolled both men and women. There were 12 studies that were conducted in North America, 6 in Europe, 2 in Japan, and 1 in Israel.

The level of adjustment for covariates varied according to study (**Table 3**). Wherever possible, risk estimates from the most fully adjusted models were used in the estimation of the pooled RR. Studies that reported positive associations between saturated fat and CVD risk included the Lipid Research Clinics Study (6), the Health Professionals Follow-Up Study (4), the Health and Lifestyle Survey (5), the Strong Heart Study (10), and studies by Mann et al (32) and Jakobsen et al (8). Notably, these positive associations were specific to subsets of the study population, ie, younger versus older (6, 8, 10), women versus men (5, 8), or for some, but not all, CHD endpoints (4).

TABLE 3

Relative risk (RR) estimates for the association of saturated fat intake and risk of coronary heart disease (CHD) or stroke in 21 unique prospective epidemiologic studies[1]

Study	Sex	Cases	Median or mean saturated fat intake	Adjusted covariates	Multivariate adjusted RR (95% CI)
Coronary heart disease studies					
Shekelle et al, 1981 (17) (Western Electric Study)	Male	Fatal CHD: 215	16.6% of total energy	Age, SBP, cigarettes per day, serum cholesterol, alcoholic drinks per month, BMI, geographic origin	β = 0.031, P = 0.144<br>For 1-unit increase in saturated fat
McGee et al, 1984 (9) (Honolulu Heart Study)[2]	Male	Total CHD: 1177	12.7% of total energy (age-adjusted)[3]	Age, total energy intake, SBP, BMI, smoking, family history of MI, physical activity, intakes of PUFA, alcohol, protein, carbohydrate, vegetable, and cholesterol	$RR_{men < 60\ y}$ = 0.92 (0.68, 1.23)[2]<br>$RR_{men \geq 60\ y}$ = 0.70 (0.41, 1.20)[2]<br>Pooled RR = 0.86 (0.67, 1.12)<br>P for test of heterogeneity = 0.39<br>For fifth vs first quintile
Kushi et al, 1985 (13) (Ireland Boston Diet Heart Study)	Male	Fatal CHD: 110	16.8% of total energy[3]	Age, SBP, serum cholesterol, cigarette smoking, alcohol intake, cohort	β = 0.061, P = 0.05<br>For 1-unit increase in saturated fat
Posner et al, 1991 (16) (Framingham Study)	Male	Total CHD: 213	45–55 y old: 15.2% of total energy[3]<br>56–65 y old: 14.8% of total energy[3]	Variable of interest, energy intake, physical activity, serum cholesterol, SBP, left ventricular hypertrophy, cigarette smoking, glucose intolerance, Metropolitan relative weight	$RR_{45–55\ y}$ = 0.78 (0.61, 1.00)<br>$RR_{\geq 56\ y}$ = 1.06 (0.86, 1.30)<br>Pooled RR = 0.92 (0.68, 1.24)<br>P for test of heterogeneity = 0.06<br>For recommended versus actual intake (15.2% vs 10%)
Fehily et al, 1993 (28) (Caerphilly Study)	Male	Total CHD: 21	17.3% for CHD-free subjects and 18.1% for CHD cases	None	RR = 1.57 (0.56, 4.42)<br>For third vs first tertile
Goldbourt et al, 1993 (35) (Israeli Ischemic Heart Disease Study)	Male	Fatal CHD: 1070	NR	Age, blood pressure, serum cholesterol, ever-smoking, diabetes prevalence in 1963	$RR_{men < 60\ y}$ = 1.05 (0.87, 1.27)[4]<br>$RR_{men \geq 60\ y}$ = 0.66 (0. 44, 1.00)[4]<br>Pooled RR = 0.86 (0.56, 1.35)<br>P for test of heterogeneity = 0.05<br>For fourth vs first quartile
Ascherio et al, 1996 (4) (Health Professionals Follow-Up Study)	Male	Total CHD: 1702	Fifth quintile: 14.8% of total energy<br>First quintile: 7.2% of total energy	Age, BMI, smoking, physical activity, history of hypertension or high blood cholesterol, history of MI < age 60 y, energy intake, fiber	$RR_{men < 60\ y}$ = 1.24 (0.87, 1.77)[4]<br>$RR_{men \geq 60\ y}$ = 1.01 (0.73, 1.41)[4]<br>Pooled RR = 1.11 (0.87, 1.42)<br>P for test of heterogeneity = 0.42<br>For fifth vs first quintile
Esrey et al, 1996 (6) (Lipid Research Clinics Study)	Both	Fatal CHD: 92	30–59 y old: 16.8% for CHD deaths and 15.1% for non-CHD deaths[3]<br>60–79 y old: 13.8% for CHD deaths and 14.3% for non-CHD deaths[3]	Age, sex, energy intake, serum lipids, SBP, cigarette smoking status, BMI, glucose intolerance	$RR_{<60\ y}$ = 1.11 (1.04, 1.18)[5]<br>$RR_{\geq 60\ y}$ = 0.96 (0.88, 1.05)<br>Pooled RR = 0.97 (0.80, 1.18)<br>P for test of heterogeneity = 0.40<br>For 1-unit increase in saturated fat
Mann et al, 1997 (32)	Both	Fatal CHD: 45	Men: Third tertile, 41.0 g/d; First tertile, 14.6 g/d<br>Women: Third tertile, 38.1 g/d; First tertile, 13.7 g/d	Age, sex, smoking, social class	RR = 2.77 (1.25, 6.13)[5]<br>For third vs first tertile

(Continued)

TABLE 3 (*Continued*)

Study	Sex	Cases	Median or mean saturated fat intake	Adjusted covariates	Multivariate adjusted RR (95% CI)
Pietinen et al, 1997 (15) (Alpha-Tocopherol, Beta-Carotene Study)	Male	Total CHD: 635	Fifth quintile: 67.5 g/d<br>First quintile: 34.7 g/d	Age, treatment group, smoking, BMI, blood pressure, education, intakes of energy, alcohol, fiber, physical activity, intakes of linoleic acid and *trans* and monounsaturated fats	RR = 0.93 (0.60, 1.44)<br>For fifth vs first quintile
Boniface and Tefft, 2002 (5) (Health and Lifestyle Survey)	Both	Fatal CHD: 155	Men: 47.0 g/d[3]<br>Women: 34.4 g/d[3]	Age, alcohol, smoking, exercise, social class	$RR_{men\ <60\ y}$ = 1.51 (0.69, 3.31)[4]<br>$RR_{men\ \geq 60y}$ = 1.01 (0.57, 1.80)[4]<br>$RR_{women\ <60\ y}$ = 1.32 (0.38, 4.57)[4]<br>$RR_{women\ \geq 60y}$ = 2.34 (1.02, 5.40)[4,5]<br>Pooled RR = 1.37 (1.17, 1.65)[5]<br>*P* for test of heterogeneity = 0.44<br>For third tertile vs first tertile
Jakobsen et al, 2004 (8)	Both	Total CHD: 326	Men:<br>19.7% of total energy<br>Women:<br>19.5% of total energy	Fat intake as % total energy intake, total energy intake, cohort identification, % energy protein, % energy other fatty acids, family history of MI, smoking, physical activity, education, alcohol, fiber, cholesterol, SBP, BMI	$RR_{women\ <60\ y}$ = 4.78 (0.95, 24.10)[4]<br>$RR_{women\ \geq 60\ y}$ = 1.03 (0.53, 2.00)[4]<br>$RR_{men\ <60\ y}$ = 1.01 (0.48, 2.14)[4]<br>$RR_{men\ \geq 60\ y}$ = 0.79 (0.48, 1.29)[4]<br>Pooled RR = 1.03 (0.66, 1.60)<br>*P* for test of heterogeneity = 0.61<br>For third tertile vs first tertile
Leosdottir et al, 2007 (14) (Malmo Diet and Cancer Study)	Both	Total CHD: 908	Men:<br>Fourth quartile, 22.3% of total energy;<br>First quartile, 12.3% of total energy<br>Women:<br>Fourth quartile, 21.8% of total energy;<br>First quartile, 12.2% of total energy	Age, smoking habits, alcohol consumption, socioeconomic status, marital status, physical activity, BMI, fiber intake, and blood pressure.	RR_{women} = 0.81 (0.53, 1.24)<br>RR_{men} = 1.02 (0.76, 1.37)<br>Pooled RR = 0.95 (0.74, 1.21)<br>*P* for test of heterogeneity = 0.38<br>For fourth vs first quartile
Oh et al, 2005 (33) (Nurses' Health Study)	Female	Total CHD: 1766	Fifth quintile: 17.6% of total energy;<br>First quintile: 10.1% of total energy	Age, BMI, cigarette smoking, alcohol intake, parental history of MI, history of hypertension, menopausal status, hormone use, aspirin use, multivitamin use, vitamin E supplement use, physical activity, intakes of energy, protein, cholesterol, MUFAs, PUFAs, *trans* fat; α-linolenic acid, marine n−3 fatty acids, cereal fiber, and fruit and vegetables	RR = 0.97 (0.74, 1.27)<br>For fifth vs first quintile
Tucker et al, 2005 (18) (Baltimore Longitudinal Study of Aging)	Male	Fatal CHD: 71	Survivors: 12.3%<br>CHD deaths: 13.8%<br>Other deaths: 14.0 % of total energy[3]	Age, total energy intake, BMI, smoking, alcohol use, physical activity score, supplement use, fruit and vegetable intakes, secular trend	$RR_{men\ <60\ y}$ = 0.57 (0.14, 2.30)[4]<br>$RR_{men\ \geq 60\ y}$ = 2.31 (0.73, 7.27)[4]<br>Pooled RR = 1.22 (0.31, 4.77)<br>*P* for test of heterogeneity = 0.13<br>For third tertile vs first tertile

(Continued)

TABLE 3 (*Continued*)

Study	Sex	Cases	Median or mean saturated fat intake	Adjusted covariates	Multivariate adjusted RR (95% CI)
Xu et al, 2006 (10) (Strong Heart Study)	Both	Total CHD: 138	Fourth quartile: 16.5% of total energy<br>First quartile: 7.5% of total energy	Variable of interest as % of energy, sex, age, study center, diabetes status, BMI, HDL, LDL, TG, smoking, alcohol consumption, hypertension, energy from protein, total energy intake	$RR_{<60\ y}$ = 5.17 (1.60, 16.4)[5]<br>$RR_{\geq 60\ y}$ = 0.80 (0.41, 1.54)<br>Pooled RR = 1.91 (0.31, 11.84)<br>*P* for test of heterogeneity = 0.006<br>For fourth quartile vs first quartile
Stroke studies					
McGee et al, 1984 (9) (Honolulu Heart Study)[2]	Male	Total stroke: 492	12.7% of total energy (age-adjusted)[3]	Age, total energy intake, SBP, BMI, smoking, family history of MI, physical activity, intakes of PUFAs, alcohol, protein, carbohydrate, vegetables, and cholesterol	$RR_{men\ <60\ y}$ = 0.95 (0.60, 1.50)[2]<br>$RR_{men\ \geq 60\ y}$ = 1.23 (0.66, 2.29)[2]<br>Pooled RR = 1.04 (0.72, 1.50)<br>*P* for test of heterogeneity = 0.52<br>For fifth vs first quintile
Goldbourt et al, 1993 (35) (Israeli Ischemic Heart Disease Study)	Male	Fatal stroke: 362	NR	Age, body height, blood pressure, smoking, diabetes	$RR_{men\ <60\ y}$ = 0.75 (0.54, 1.05)[4]<br>$RR_{men\ \geq 60\ y}$ = 1.26 (0.70, 2.29)[4]<br>Pooled RR = 0.92 (0.56, 1.51)<br>*P* for test of heterogeneity = 0.13<br>For fourth quartile vs first quartile
Gillman et al, 1997 (11) (Framingham Study)	Male	Ischemic stroke: 61	15.0% of total energy[3]	Age, total energy, SBP, cigarette smoking, glucose intolerance, BMI, physical activity, left ventricular hypertrophy, and intakes of alcohol and fruit and vegetables	$RR_{ischemic\ stroke}$ = 0.90 (0.83, 0.96)[5]<br>For 1% increase in saturated fat
Iso et al, 2001 (31) (Nurses' Health Study)	Female	Hemorrhagic stroke: 74	Fifth quintile: 36 g/d<br>First quintile: 20 g/d	Age, smoking, time interval, BMI, alcohol intake, menopausal status, postmenopausal hormone use, vigorous exercise, usual aspirin use, multivitamins, vitamin E, n−3 fatty acids, calcium, total energy intake, quintiles of cholesterol, MUFAs, PUFAs (linoleic), vegetable protein, *trans* or unsaturated fat, animal protein, history of hypertension, diabetes, and high cholesterol	RR = 1.05 (0.33, 3.39)<br>For fifth quintile vs first quintile
He et al, 2003 (29) (Health Professionals Follow-Up Study)	Male	Total stroke: 598	Fifth quintile: 31 g/d<br>First quintile: 17 g/d	BMI, physical activity, history of hypertension, smoking status, aspirin use, multivitamin use, alcohol consumption, potassium, fiber, vitamin E, fruit and vegetables, total energy, hypercholesterolemia, other fats (MUFAs, PUFAs, and *trans* fats)	$RR_{men\ <60\ y}$ = 0.72 (0.35, 1.51)[4]<br>$RR_{men\ \geq 60\ y}$ = 0.82 (0.49, 1.36)[4]<br>Pooled RR= 0.79 (0.52, 1.19)<br>*P* for test of heterogeneity = 0.79<br>For fifth quintile vs first quintile

(Continued)

TABLE 3 (*Continued*)

Study	Sex	Cases	Median or mean saturated fat intake	Adjusted covariates	Multivariate adjusted RR (95% CI)
Iso et al, 2003 (30)	Both	Hemorrhagic stroke: 67	Fourth quartile: 17.1 g/d<br>First quartile: 5.2 g/d	Age, sex, total energy intake, BMI, hypertension, diabetes, total cholesterol, smoking status, ethanol intake, menopausal status (for women)	RR = 0.30 (0.13, 0.71)[5]<br>For fourth quartile vs first quartile
Sauvaget et al, 2004 (34) (Adult Health Study)	Both	Ischemic stroke: 60	NR	Age and sex stratified and adjusted for radiation dose, city, BMI, smoking, alcohol, history of hypertension and diabetes	HR = 0.58 (0.28, 1.20)<br>For third tertile vs first tertile
Leosdottir et al, 2007 (14) (Malmo Diet and Cancer Study)	Both	Ischemic stroke:648	Men:<br>Fourth quartile, 22.3% of total energy;<br>First quartile, 12.3% of total energy<br>Women:<br>Fourth quartile, 21.8% of total energy;<br>First quartile, 12.2% of total energy	Age, smoking habits, alcohol consumption, socioeconomic status, marital status, physical activity, BMI, fiber intake, blood pressure	RR_{women} = 1.26 (0.81, 1.96)<br>RR_{men} = 1.19 (0.80, 1.77)<br>Pooled RR = 1.22 (0.91, 1.64)<br>*P* for test of heterogeneity = 0.85<br>For fourth vs first quartile

[1] MI, myocardial infarction; MUFA, monounsaturated fatty acid; NR, not reported; PUFA, polyunsaturated fatty acid; SBP, systolic blood pressure; HR, hazard ratio; TG, triglycerides.
[2] RR estimates were derived from a provided data set (36).
[3] Values represent the mean intake of saturated fatty acid.
[4] Data were provided by study investigators on request.
[5] Statistically significant relation.

Although the Strong Heart Study reported a positive association between saturated fat and CVD in younger than in older individuals (ie, RR = 5.17; 95% CI: 1.6, 16.4), the fully adjusted model that included adjustment for polyunsaturated fatty acids, *trans* fats, and monounsaturated fatty acids was not statistically significant (RR = 2.98; 95% CI: 0.66, 13.6).

In contrast, a number of studies did not show significant associations of dietary saturated fat intake with CHD, including the Western Electric Study (17), the Honolulu Heart Study (9), the Ireland Boston Diet Heart Study (13), the Caerphilly Study (28), the Framingham Heart Study (16), the Israeli Ischemic Study (35), the Alpha-Tocopherol, Beta-Carotene Study (15), the Nurses' Health Study (33), the Malmo Diet and Cancer Study (14), and the Baltimore Longitudinal Study of Aging (18).

With respect to stroke, although inverse associations of saturated fat intake with hemorrhagic stroke were reported in 2 studies (11, 30), no association between saturated fat and stroke was found in 6 other studies (9, 14, 29, 31, 34, 35). The relation of saturated fat with ischemic versus hemorrhagic stroke may differ given their different biological mechanisms, and consideration of these 2 disease states as distinct endpoints may be important.

Individual study estimates as well as the overall estimate for CHD, stroke, and CVD are shown in **Figure 2**. Saturated fat intake was not associated with an elevated risk of CHD, stroke, or CVD as a composite outcome. The RRs (95% CIs) were 1.07 (0.96, 1.19) for risk of CHD, 0.81 (0.62, 1.05) for risk of stroke, and 1.00 (0.89, 1.11) for overall CVD risk. Two of the 8 studies included in the meta-analysis related to stroke examined hemorrhagic stroke exclusively (30, 31). When these 2 studies were excluded from the meta-analysis, the pooled RR (95% CI) was 0.86 (0.67, 1.11).

We documented heterogeneity among studies that examined saturated fat in relation to CHD ($P = 0.04$) or stroke ($P = 0.01$). However, age ($P = 0.16$ for CHD, 0.40 for stroke), sex ($P = 0.52$ for CHD, 0.25 for stroke), sample size ($P = 0.44$ for CHD, 0.71 for stroke), duration of follow-up ($P = 0.53$ for CHD, 0.42 for stroke), medical record review for CVD outcome confirmation ($P = 0.17$ for CHD, 0.30 for stroke), and study quality as assessed by a quality score ($P = 0.62$ for CHD, 0.70 for stroke) could not explain this heterogeneity. Quality scores for each study are provided in Supplementary Table 1 (*see* "Supplemental data" in the online issue).

No individual study had a particularly large influence on the pooled estimate of RR for CVD, although Gillman et al's (11) and Boniface and Tefft's (5) studies had relatively stronger effects on the overall RR estimate than did other studies. The pooled RRs for CVD were 1.03 (95% CI: 0.93, 1.14) after excluding Gillman et al and 0.97 (95% CI: 0.88, 1.08) after excluding Boniface and Tefft. When these 2 studies were excluded simultaneously, the pooled RRs (95% CI) were 1.02 (0.94, 1.11) for CHD, 0.86 (0.65, 1.14) for stroke, and 1.00 (0.92, 1.10) for CVD, respectively.

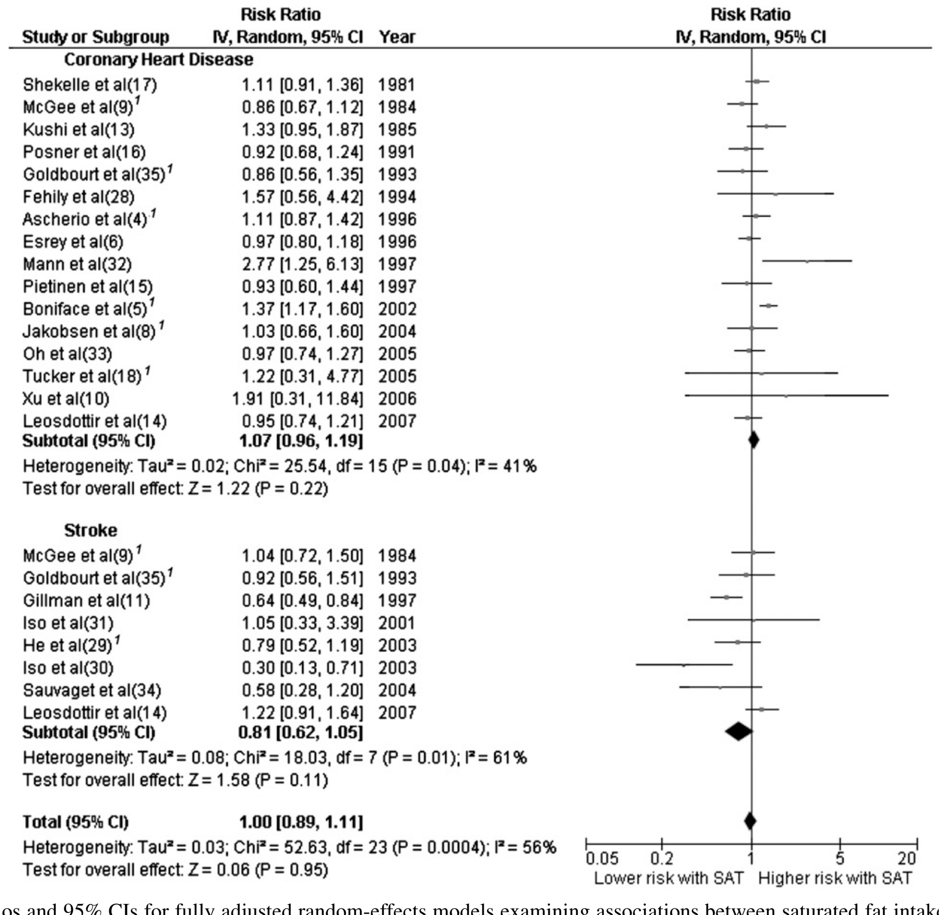

FIGURE 2. Risk ratios and 95% CIs for fully adjusted random-effects models examining associations between saturated fat intake in relation to coronary heart disease and stroke. [1]Updated data were provided by respective investigators (4, 5, 8, 18, 29, 35) or derived from a provided data set (9, 36). SAT, saturated fat intake; IV, inverse variance.

Subgroup analyses evaluating the association of saturated fat with CVD by sex or age (< or ≥ 60 y) showed no significant associations (*see* Supplementary Figures 1 and 2, respectively, under "Supplemental data" in the online issue). In men, the pooled RR (95% CI) of CVD in relation to saturated fat intake was 0.97 (0.87, 1.08), whereas in women this figure was 1.06 (0.86, 1.32). The associations for saturated fat intake were similar between participants who were younger than 60 y at baseline and those who were older: the pooled RRs (95% CIs) were 0.98 (0.84, 1.13) and 0.98 (0.86, 1.10), respectively. Further stratification by both age and sex (ie, men or women younger than 60 y and men or women older than 60 y) also showed no significant associations between saturated fat and CHD risk, although sample size may have been inadequate for these analyses (*see* Supplementary Figures 3 and 4, respectively, under "Supplemental data" in the online issue). The limited number of studies excluded our ability to further stratify the analysis by study outcome.

Of 21 studies, 15 studies adjusted for total energy intake in the fully adjusted model. Secondary analyses conducted within these studies showed results largely similar to the primary analysis (*see* Supplementary Figure 5 under "Supplemental data" in the online issue). Six studies further adjusted for energy from other fats and protein, but left energy from carbohydrate out of the fully adjusted model. In these studies, the pooled RR (95% CI) was 0.98 (0.86, 1.13) for CHD, 0.93 (0.71, 1.21) for stroke, and 0.97 (0.86, 1.10) for overall CVD (*see* Supplementary Figure 6 under "Supplemental data" in the online issue). Similarly, 5 studies adjusted for energy from carbohydrate, protein, and fats but not polyunsaturated fat. The pooled RR (95% CI) for these studies was 1.07 (0.91, 1.25) for CHD, 0.95 (0.81, 1.13) for stroke, and 1.02 (0.92, 1.14) for overall CVD (*see* Supplementary Figure 7 under "Supplemental data" in the online issue).

A funnel plot of the 21 studies that evaluated the association of saturated fat with CVD is provided in **Figure 3**. The larger studies at the top of the plot were somewhat more symmetrically distributed than were the smaller studies at the bottom. This suggests the heterogeneity of the study estimates as well as possible publication bias favoring studies with significant results.

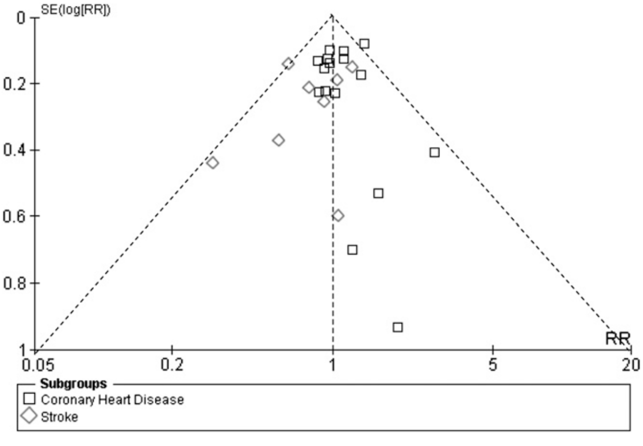

FIGURE 3. Funnel plot of studies of saturated fat intake in relation to cardiovascular disease. Dotted lines are pseudo 95% CIs. The large studies at the top of the plot were somewhat more symmetrically distributed than the small studies at the bottom. This indicates publication bias favoring studies with significant results. RR, risk ratio.

DISCUSSION

This study sought to evaluate the effects of dietary saturated fat on CVD risk by summarizing the data available from informative epidemiologic studies and including, where possible and relevant, supplementary information that had been provided on request from investigators of the component studies. The conglomeration of data from 16 studies with CHD as an endpoint and 8 studies with stroke as the endpoint showed no association of dietary saturated fat on disease prevalence after adjustment for other nutrients wherever possible. Evaluation of the subset of studies (n = 15) that adjusted for total energy, which has been shown to be relevant in evaluating nutrient-disease relations (39), yielded similar findings. This study had several strengths, including the selection of prospective epidemiologic studies that statistically adjusted for relevant covariates and the inclusion of large studies with a significant number of incident cases. Furthermore, the use of the random-effects model in our analyses allowed for the heterogeneity of variance between studies.

A caveat of this study was its reliance on the accuracy of the dietary assessments of the component studies, which may vary depending on the method used (25). Underreporting of calories has often contributed to the error associated with dietary assessments, particularly in overweight individuals. Generally, 4- to 7-d food records are considered to be the most accurate means of dietary assessment, but such methods are generally not feasible in large cohort studies. A single 24-h recall is relatively easy to collect, but the information does not reflect long-term dietary patterns. FFQs have become the method of choice in large epidemiologic studies because they are inexpensive and can assess long-term diets (25); however, this method is also subject to random and systematic errors.

As part of a quality score, the method of nutrient assessment was taken into account, and the risk estimates that each study contributed were adjusted based on this quality score, which also considered whether the dietary assessment method had been validated or repeatedly performed as well as the number of covariates included in the model. The latter criterion assumed that investigators included all relevant covariates in their regression models. Evaluation of the studies on the basis of this quality score did not change the findings of this meta-analysis.

Only a limited number of studies provided data that enabled the evaluation of the effects of isocalorically replacing saturated fat with carbohydrate or polyunsaturated fat, and, as such, the statistical power was diminished for the secondary analyses restricted to these studies. Most recently, however, an analysis conducted in a pooled cohort of studies showed a lower CHD risk when saturated fat was replaced with polyunsaturated fat and increased nonfatal myocardial infarction, but not fatal CHD, risk when saturated fat was replaced with carbohydrate (24).

Inverse associations of polyunsaturated fat and CVD risk have previously been reported (41, 42). Replacement of 5% of total energy from saturated fat with polyunsaturated fat has been estimated to reduce CHD risk by 42% (43). Notably, the amount of dietary polyunsaturated fat in relation to saturated fat (ie, the P:S ratio) has been reported to be more significantly associated with CVD than saturated fat alone, with a reduced CHD risk found with P:S ratios ≥ 0.49 (44). Only 1 of the 21 studies that met criteria for inclusion in this meta-analysis evaluated the relation of the P:S ratio with CHD (14). No effect was seen in this study,

in which the average P:S ratio was ≈0.4, nor was there an association of P:S ratio with CVD in the Israeli Ischemic Heart Study (U Goldbourt, personal communication, 2008). However, these studies were relatively small.

Of note, in intervention trials that have shown protective effects of reducing saturated fat, ie, the Veteran Affairs (19), Oslo Diet Heart (20), and Finnish Mental Hospital (21) studies, the calculated P:S ratios ranged from 1.4 to 2.4—values that are much higher than the threshold of 0.49 above which CHD risk has been reported to be reduced (44). Relatively high P:S ratios (1.25–1.5) were also observed in the Anti-Coronary Club Study, an early trial that showed beneficial effects of a lower fat diet (30–32% of total energy) (45). The presumed beneficial effects of diets with reduced saturated fat on CVD risk may therefore be dependent on a significant increase in polyunsaturated fat in the diet. Existing epidemiologic studies and clinical trials support that substituting polyunsaturated fat for saturated fat is more beneficial for CHD risk than exchanging carbohydrates for saturated fat in the diet, as described further elsewhere (46).

With respect to dietary carbohydrate, the type of carbohydrate (ie, a high or low glycemic index) that replaces saturated fat is likely important in influencing dietary effects on CVD risk (47). However, there was insufficient information in the component studies of this meta-analysis to permit examination of this issue.

Our results suggested publication bias, such that studies with significant associations tended to be received more favorably for publication. If unpublished studies with null associations were included in the current analysis, the pooled RR estimate for CVD could be even closer to null. Furthermore, despite several indications in the published literature that sex and age may modify the association of saturated fat with CHD (5, 6, 8, 10), we did not observe effects of these variables on CHD risk. The lack of an association may have been related to limited statistical power.

Although an inverse association of saturated fat with stroke risk has been previously described (48), saturated fat intake was not significantly associated with risk of stroke in the current meta-analysis. The exclusion of 2 studies conducted in Japan (30, 34), where saturated fat intake is known to be significantly lower than in Western populations, did not substantially change the RR estimate (RR = 0.90; 95% CI: 0.70, 1.15). The exclusion of 2 studies that evaluated hemorrhagic stroke specifically (30, 31) also did not alter the RR estimate; however, these findings were likely limited by statistical power.

In conclusion, our meta-analysis showed that there is insufficient evidence from prospective epidemiologic studies to conclude that dietary saturated fat is associated with an increased risk of CHD, stroke, or CVD. However, the available data were not adequate for determining whether there are CHD or stroke associations with saturated fat in specific age and sex subgroups. Furthermore, there was insufficient statistical power for this meta-analysis to assess the effects on CVD risk of replacing specific amounts of saturated fat with either polyunsaturated fat or carbohydrate. Finally, nutritional epidemiologic studies provide only one category of evidence for evaluating the relation of saturated fat intake to risk for CHD, stroke, and CVD. An overall assessment requires consideration of results of clinical trials as well as information regarding the effects of saturated fat on underlying disease mechanisms, as discussed elsewhere in this issue (46).

We thank David Boniface, Alberto Ascherio, Dariush Mozaffarian, Katherine Tucker, Uri Goldbourt, and Marianne Jakobsen for providing data requested for inclusion in this meta-analysis. The Honolulu Heart Program is conducted and supported by the National Heart, Lung, and Blood Institute (NHLBI) in collaboration with the Honolulu Heart Program Study Investigators. This manuscript was prepared by using a limited access data set obtained from the NHLBI and does not necessarily reflect the opinions or views of the Honolulu Heart Program or the NHLBI.

The authors' responsibilities were as follows—PWS-T, QS, FBH, and RMK: selected the studies for inclusion in the meta-analysis; PWS-T and QS: extracted data from the studies and wrote the manuscript; QS: performed the statistical analyses; and FBH and RMK: provided significant advice and consultation. No conflicts of interest were reported.

REFERENCES

1. Anitschkow N. A history of experimentation on arterial atherosclerosis in animals. Cowdry's arteriosclerosis: a survey of the problem. Springfield, IL: Charles C Thomas, 1967:21–44.
2. Kato H, Tillotson J, Nichaman MZ, Rhoads GG, Hamilton HB. Epidemiologic studies of coronary heart disease and stroke in Japanese men living in Japan, Hawaii and California. Am J Epidemiol 1973;97:372–85.
3. Keys A, Aravanis C, Blackburn HW, et al. Epidemiological studies related to coronary heart disease: characteristics of men aged 40-59 in seven countries. Acta Med Scand Suppl 1966;460:1–392.
4. Ascherio A, Rimm EB, Giovannucci EL, Spiegelman D, Stampfer M, Willett WC. Dietary fat and risk of coronary heart disease in men: cohort follow up study in the United States. BMJ 1996;313:84–90.
5. Boniface DR, Tefft ME. Dietary fats and 16-year coronary heart disease mortality in a cohort of men and women in Great Britain. Eur J Clin Nutr 2002;56:786–92.
6. Esrey KL, Joseph L, Grover SA. Relationship between dietary intake and coronary heart disease mortality: lipid research clinics prevalence follow-up study. J Clin Epidemiol 1996;49:211–6.
7. Garcia-Palmieri MR, Sorlie P, Tillotson J, Costas R Jr, Cordero E, Rodriguez M. Relationship of dietary intake to subsequent coronary heart disease incidence: the Puerto Rico Heart Health Program. Am J Clin Nutr 1980;33:1818–27.
8. Jakobsen MU, Overvad K, Dyerberg J, Schroll M, Heitmann BL. Dietary fat and risk of coronary heart disease: possible effect modification by gender and age. Am J Epidemiol 2004;160:141–9.
9. McGee DL, Reed DM, Yano K, Kagan A, Tillotson J. Ten-year incidence of coronary heart disease in the Honolulu Heart Program: relationship to nutrient intake. Am J Epidemiol 1984;119:667–76.
10. Xu J, Eilat-Adar S, Loria C, et al. Dietary fat intake and risk of coronary heart disease: the Strong Heart Study. Am J Clin Nutr 2006;84:894–902.
11. Gillman MW, Cupples LA, Millen BE, Ellison RC, Wolf PA. Inverse association of dietary fat with development of ischemic stroke in men. JAMA 1997;278:2145–50.
12. Mozaffarian D, Rimm EB, Herrington DM. Dietary fats, carbohydrate, and progression of coronary atherosclerosis in postmenopausal women. Am J Clin Nutr 2004;80:1175–84.
13. Kushi LH, Lew RA, Stare FJ, et al. Diet and 20-year mortality from coronary heart disease. The Ireland-Boston Diet-Heart Study. N Engl J Med 1985;312:811–8.
14. Leosdottir M, Nilsson PM, Nilsson JA, Berglund G. Cardiovascular event risk in relation to dietary fat intake in middle-aged individuals: data from The Malmo Diet and Cancer Study. Eur J Cardiovasc Prev Rehabil 2007;14:701–6.
15. Pietinen P, Ascherio A, Korhonen P, et al. Intake of fatty acids and risk of coronary heart disease in a cohort of Finnish men. The Alpha-Tocopherol, Beta-Carotene Cancer Prevention Study. Am J Epidemiol 1997;145:876–87.
16. Posner BM, Cobb JL, Belanger AJ, Cupples LA, D'Agostino RB, Stokes J III. Dietary lipid predictors of coronary heart disease in men. The Framingham Study. Arch Intern Med 1991;151:1181–7.
17. Shekelle RB, Shryock AM, Paul O, et al. Diet, serum cholesterol, and death from coronary heart disease. The Western Electric study. N Engl J Med 1981;304:65–70.
18. Tucker KL, Hallfrisch J, Qiao N, Muller D, Andres R, Fleg JL. The combination of high fruit and vegetable and low saturated fat intakes is

more protective against mortality in aging men than is either alone: the Baltimore Longitudinal Study of Aging. J Nutr 2005;135:556–61.
19. Dayton S, Pearce M, Hashimoto S, et al. A controlled clinical trial of a diet high in unsaturated fat in preventing complications of atherosclerosis. Circulation 1969;40(suppl 2):1–63.
20. Leren P. The Oslo Diet-Heart Study: eleven-year report. Circulation 1970;42:935–42.
21. Turpeinen O, Karvonen MJ, Pekkarinen M, Miettinen M, Elosuo R, Paavilainen E. Dietary prevention of coronary heart disease: the Finnish Mental Hospital Study. Int J Epidemiol 1979;8:99–118.
22. Frantz ID Jr, Dawson EA, Ashman PL, et al. Test of effect of lipid lowering by diet on cardiovascular risk. The Minnesota Coronary Survey. Arteriosclerosis 1989;9:129–35.
23. Morris JN, Ball KP, Antonis A, et al Controlled trial of soya-bean oil in myocardial infarction. Lancet 1968;XXX:693–9.
24. Jakobsen MU, O'Reilly EJ, Heitmann BL, et al. Major types of dietary fat and risk of coronary heart disease: a pooled analysis of 11 cohort studies. Am J Clin Nutr 2009;89:1425–32.
25. Willett W. Nutritional epidemiology. 2nd ed. New York, NY: Oxford University Press, 1998.
26. Kromhout D, de Lezenne Coulander C, Obermann-de Boer GL, van Kampen-Donker M, Goddijn E, Bloemberg BP. Changes in food and nutrient intake in middle-aged men from 1960 to 1985 (the Zutphen Study). Am J Clin Nutr 1990;51:123–9.
27. Boden-Albala B, Elkind MS, White H, Szumski A, Paik MC, Sacco RL. Dietary total fat intake and ischemic stroke risk: the Northern Manhattan Study. Neuroepidemiology 2009;32:296–301.
28. Fehily AM, Yarnell JW, Sweetnam PM, Elwood PC. Diet and incident ischaemic heart disease: the Caerphilly Study. Br J Nutr 1993;69:303–14.
29. He K, Merchant A, Rimm EB, et al. Dietary fat intake and risk of stroke in male US healthcare professionals: 14 year prospective cohort study. BMJ 2003;327:777–82.
30. Iso H, Sato S, Kitamura A, Naito Y, Shimamoto T, Komachi Y. Fat and protein intakes and risk of intraparenchymal hemorrhage among middle-aged Japanese. Am J Epidemiol 2003;157:32–9.
31. Iso H, Stampfer MJ, Manson JE, et al. Prospective study of fat and protein intake and risk of intraparenchymal hemorrhage in women. Circulation 2001;103:856–63.
32. Mann JI, Appleby PN, Key TJ, Thorogood M. Dietary determinants of ischaemic heart disease in health conscious individuals. Heart 1997;78: 450–5.
33. Oh K, Hu FB, Manson JE, Stampfer MJ, Willett WC. Dietary fat intake and risk of coronary heart disease in women: 20 years of follow-up of the nurses' health study. Am J Epidemiol 2005;161:672–9.
34. Sauvaget C, Nagano J, Hayashi M, Yamada M. Animal protein, animal fat, and cholesterol intakes and risk of cerebral infarction mortality in the adult health study. Stroke 2004;35:1531–7.
35. Goldbourt U, Yaari S, Medalie JH. Factors predictive of long-term coronary heart disease mortality among 10,059 male Israeli civil servants and municipal employees. A 23-year mortality follow-up in the Israeli Ischemic Heart Disease Study. Cardiology 1993;82:100–21.
36. National Heart, Lung, and Blood Institute. Honolulu Heart Program limited data set. Bethesda, MD: National Heart, Lung, and Blood Institute, 2008.
37. Greenland S. Quantitative methods in the review of epidemiologic literature. Epidemiol Rev 1987;9:1–30.
38. Ding EL, Song Y, Malik VS, Liu S. Sex differences of endogenous sex hormones and risk of type 2 diabetes: a systematic review and meta-analysis. JAMA 2006;295:1288–99.
39. Willett WC, Howe GR, Kushi LH. Adjustment for total energy intake in epidemiologic studies. Am J Clin Nutr 1997;65(suppl):1220S–8S; discussion 1229S–31S.
40. Egger M, Altman DG, Smith GD, eds. Systematic reviews in health care: meta-analysis in context. London, United Kingdom: BMJ Books, 2001.
41. Laaksonen DE, Nyyssonen K, Niskanen L, Rissanen TH, Salonen JT. Prediction of cardiovascular mortality in middle-aged men by dietary and serum linoleic and polyunsaturated fatty acids. Arch Intern Med 2005;165:193–9.
42. Soinio M, Laakso M, Lehto S, Hakala P, Ronnemaa T. Dietary fat predicts coronary heart disease events in subjects with type 2 diabetes. Diabetes Care 2003;26:619–24.
43. Hu FB, Stampfer MJ, Manson JE, et al. Dietary fat intake and the risk of coronary heart disease in women. N Engl J Med 1997;337:1491–9.
44. Hu FB, Stampfer MJ, Manson JE, et al. Dietary saturated fats and their food sources in relation to the risk of coronary heart disease in women. Am J Clin Nutr 1999;70:1001–8.
45. Christakis G, Rinzler SH, Archer M, Maslansky E. Summary of the research activities of the anti-coronary club. Public Health Rep 1966;81:64–70.
46. Siri-Tarino PW, Sun Q, Hu FB, Krauss RM. Saturated fat, carbohydrate, and cardiovascular disease. Am J Clin Nutr 2010;91:502–9.
47. Barclay AW, Petocz P, McMillan-Price J, et al. Glycemic index, glycemic load, and chronic disease risk–a meta-analysis of observational studies. Am J Clin Nutr 2008;87:627–37.
48. Corvol JC, Bouzamondo A, Sirol M, Hulot JS, Sanchez P, Lechat P. Differential effects of lipid-lowering therapies on stroke prevention: a meta-analysis of randomized trials. Arch Intern Med 2003;163: 669–76.
