# Supplementary material for: A systematic comprehensive longitudinal evaluation of dietary factors associated with acute myocardial infarction and fatal coronary heart disease
Source: Nat Commun. 2020 Nov 27;11:6074. doi: 10.1038/s41467-020-19888-2 (PMC7699643; doi:10.1038/s41467-020-19888-2)
Supplement: Supplementary file 4 — Supplementary Software [file 41467_2020_19888_MOESM4_ESM.zip › EWAS-NHS-master/KG-SI/Rest/Stampfer (1993).pdf]

VITAMIN E CONSUMPTION AND THE RISK OF CORONARY DISEASE IN WOMEN

MEIR J. STAMPFER, M.D., CHARLES H. HENNEKENS, M.D., JOANN E. MANSON, M.D., GRAHAM A. COLDITZ, M.B., B.S., BERNARD ROSNER, PH.D., AND WALTER C. WILLETT, M.D.

Abstract *Background.* Interest in the antioxidant vitamin E as a possible protective nutrient against coronary disease has intensified with the recognition that oxidized low-density lipoprotein may be involved in atherogenesis.

Methods. In 1980, 87,245 female nurses 34 to 59 years of age who were free of diagnosed cardiovascular disease and cancer completed dietary questionnaires that assessed their consumption of a wide range of nutrients, including vitamin E. During follow-up of up to eight years (679,485 person-years) that was 97 percent complete, we documented 552 cases of major coronary disease (437 nonfatal myocardial infarctions and 115 deaths due to coronary disease).

Results. As compared with women in the lowest fifth of the cohort with respect to vitamin E intake, those in the top fifth had a relative risk of major coronary disease of 0.66 (95 percent confidence interval, 0.50 to 0.87) after adjustment for age and smoking. Further adjustment for a variety of other coronary risk factors and nutrients, including other antioxidants, had little effect on the results. Most of the variability in intake and reduction in risk was attributable to vitamin E consumed as supplements. Women who took vitamin E supplements for short periods had little apparent benefit, but those who took them for more than two years had a relative risk of major coronary disease of 0.59 (95 percent confidence interval, 0.38 to 0.91) after adjustment for age, smoking status, risk factors for coronary disease, and use of other antioxidant nutrients (including multivitamins).

Conclusions. Although these prospective data do not prove a cause-and-effect relation, they suggest that among middle-aged women the use of vitamin E supplements is associated with a reduced risk of coronary heart disease. Randomized trials of vitamin E in the primary and secondary prevention of coronary disease are being conducted; public policy recommendations about the widespread use of vitamin E should await the results of these trials. (N Engl J Med 1993;328:1444-9.)

RAPIDLY growing evidence suggests that oxidation of low-density lipoprotein (LDL) plays an important part in atherosclerosis. As Steinberg et al. have found,[1-3] oxidized LDL is taken up more readily than native LDL by macrophages to create foam cells. Also, oxidized LDL is chemotactic for circulating monocytes,[4] and it inhibits the motility of tissue macrophages.[5] It may also be cytotoxic to endothelial cells[6] and may increase vasoconstriction in arteries.[7] Oxidized LDL has been identified in atherosclerotic lesions,[8-10] and elevated titers of circulating autoantibodies to epitopes of oxidized LDL are found in patients with atherosclerosis.[11] Lipid peroxide concentrations have been found to be higher in patients with atherosclerosis.[12] In addition, the susceptibility of LDL to oxidation was correlated with the severity of atherosclerosis.[13]

Vitamin E is a potent lipid-soluble antioxidant carried in LDL.[14,15] It inhibits the proliferation of smooth-muscle cells in vitro,[16] and when added to plasma, it increases the resistance of LDL to oxidation.[17] LDL from volunteers given alpha-tocopherol supplements showed increased resistance to oxidation.[18] Several small trials of vitamin E in peripheral vascular disease have been reported, but the results have been inconclusive.[19-23] We therefore studied the association between vitamin E intake and the incidence of major coronary disease events in the Nurses' Health Study.

METHODS

The Nurses' Health Study began in 1976, when 121,700 female registered nurses living in 11 states completed mailed questionnaires containing items about lifestyle and medical history.[24,25] Every two years, follow-up questionnaires are sent to update the information and identify newly diagnosed conditions.

Ascertainment of Diet and Use of Vitamin E Supplements

In 1980, a dietary questionnaire was included in the mailing that listed 61 food items, each with a specified portion size. The women reported the average frequency with which they had consumed each item during the previous year. We computed the average daily intake of nutrients by multiplying the frequency of consumption of each item by the nutrient content, and totaling the nutrient intake for all the food items. The reproducibility and validity of this questionnaire have been described elsewhere.[26-36] The correlation between vitamin E intake as assessed by the questionnaire and plasma alpha-tocopherol levels was 0.34 ($P = 0.006$) in one study[26] and 0.42 ($P = 0.03$) in another.[31] Because the correlation between two plasma levels measured eight weeks apart is about 0.6,[36] a higher correlation between a single plasma determination and dietary intake cannot be expected.

We assessed the dietary intake of the participants in 1980, but information on vitamin E intake and use of multivitamin supplements was also collected on each subsequent biennial questionnaire. Participants reported whether they regularly used multivitamin supplements, and if so, the exact type and brand. We also inquired about specific supplements, including vitamins A, C, and E and beta carotene.

Study Population

A total of 98,462 nurses returned the 1980 diet questionnaire. We excluded women who left 10 or more items blank (4 percent), whose reported food scores were implausible (2.7 percent), and who had a history of cancer (except nonmelanoma skin cancer), angina, myocardial infarction, stroke, or other cardiovascular disease. A total of 87,245 women remained. All the exclusions were made before the analysis of the data.

Follow-up questionnaires were mailed to the participants in 1982, 1984, 1986, and 1988. Women who reported diagnoses of cardiovascular diseases were asked for permission to examine their medical records. We included women with cardiovascular conditions that occurred after the return of the 1980 questionnaire but before June 1, 1988. For each woman, only the first event was considered. On the basis of the responses to the questionnaire, follow-up for nonfatal outcomes was 97.1 percent of the total possible person-years.

A myocardial infarction was considered confirmed if it met the World Health Organization criteria[37] of symptoms and either typical electrocardiographic changes or elevated cardiac enzymes. Infarctions of indeterminate age were excluded. We designated as

From the Channing Laboratory and the Division of Preventive Medicine, Department of Medicine (M.J.S., C.H.H., J.E.M., G.A.C., B.R., W.C.W.), Harvard Medical School and Brigham and Women's Hospital; and the Departments of Epidemiology (M.J.S., G.A.C., W.C.W.), Biostatistics (B.R.), and Nutrition (W.C.W.), Harvard School of Public Health — all in Boston. Address reprint requests to Dr. Stampfer at the Channing Laboratory, 180 Longwood Ave., Boston, MA 02115.

Supported by research grants (HL 24074, HL 34594, CA 40935, and CA 40356) from the National Institutes of Health.

The New England Journal of Medicine
Downloaded from nejm.org at NORTHEASTERN UNIVERSITY on November 18, 2017. For personal use only. No other uses without permission.
Copyright © 1993 Massachusetts Medical Society. All rights reserved.

probable infarctions those that were reported by the nurse, required hospitalization, and were corroborated by additional information (in a letter or telephone interview), but for which records were unobtainable.

Most deaths were reported by relatives or postal authorities. We searched the National Death Index for the names of nonrespondents and estimate that over 98 percent of deaths were identified.[38] When death from cardiovascular disease was suspected, we requested permission to review the medical records. A death was designated as due to coronary heart disease if it involved a confirmed fatal myocardial infarction or if the woman was known to have had coronary heart disease and coronary disease was listed as the underlying cause on the death certificate without another, more plausible cause. In no case did we rely solely on the listing of a cause on the death certificate as confirmation of death from coronary disease. Since sudden death in women can often occur without coronary disease, we excluded 26 cases of sudden death — i.e., death within one hour of the onset of symptoms in an apparently healthy woman without other evidence of coronary disease.

Although the primary a priori end point was major coronary disease (defined as nonfatal myocardial infarction or death due to coronary disease), we also assessed other cardiovascular events. We considered as one category coronary-artery surgery (bypass or angioplasty), on the basis of reports by the women.

Strokes were confirmed by medical records and classified, according to the criteria of the National Survey of Stroke, as ischemic strokes (embolic or thrombotic), subarachnoid hemorrhages, intracerebral hemorrhages, or strokes of unknown cause.[39] We included strokes that required hospitalization and were corroborated by information in a letter or interview, and fatal strokes that could be confirmed by medical records, other reliable information, or a death certificate.

Statistical Analysis

The primary analysis was based on incidence rates. For all women, person-months were allocated according to the exposure variables on the most recent questionnaire until death or an end point was reached, or until May 31, 1988. We used information on the intake of vitamin E in 1980 from dietary sources only. Height and parental history of myocardial infarction were both ascertained in 1976; all other exposures apart from diet, including the consumption of multivitamins and vitamin E supplements, were updated on each follow-up questionnaire.

To avoid a spurious finding due to the influence of disease on the use of vitamin supplements, women with a diagnosis of any cardiovascular disease or cancer (except nonmelanoma skin cancer) were excluded from further analysis. Because diet was not updated after 1980, the analyses of dietary vitamin E intake were based on women who did not have those diagnoses in 1980. In the analyses of the use of vitamin E supplements, for which data were updated biennially, women with new diagnoses of cardiovascular disease or cancer were excluded at the beginning of each two-year follow-up period. Thus, at the start of each such period, the base population included no women who reported these diagnoses.

For the analyses of total vitamin E intake, we divided the cohort according to quintiles based on intake, both with and without adjustment for total energy intake.[28] The relative risk was defined as the incidence in women in various categories of vitamin E intake (the number of end points divided by the person-time of follow-up in that category) divided by the corresponding rate for the women in the lowest category of intake. For the analyses of the use of vitamin E supplements, we compared users of such supplements with nonusers. Relative risks with 95 percent confidence intervals were adjusted for age in five-year categories, and tests for trend across fifths of the group were performed.[40] Stratified analyses that adjusted for age and one other variable at the same time were performed to assess possible effect modification and confounding. To adjust simultaneously for multiple risk factors, proportional-hazards models[41] were employed with vitamin E supplements used as a time-dependent variable. All P values were two-tailed.

Results

During 679,485 person-years of follow-up from 1980 to 1988, 552 cases of major coronary disease were documented: 437 nonfatal myocardial infarctions (360 confirmed and 77 probable) and 115 confirmed deaths from coronary disease. Because there were no material differences in any analyses between fatal and nonfatal outcomes or in the results when the probable cases were excluded, these have been combined into a single category. Analyses with or without adjustment for total energy intake yielded very similar results; for simplicity, therefore, only the unadjusted results are shown. We observed a pronounced and statistically significant reduction in the risk of major coronary disease among women with a high intake of vitamin E, as compared with those with a low intake (Table 1). After adjustment for age and smoking status, the relative risk for those in the highest fifth of intake was 0.66 (95 percent confidence interval, 0.50 to 0.87), as compared with those in the lowest fifth. The apparent benefit was attributable mainly to the use of vitamin E from supplements, because high levels of intake from dietary sources were not associated with significant reductions in risk. Even the highest levels of dietary intake of vitamin E were far lower, however, than the intake among supplement users. Analyses adjusting for age and smoking status (Table 1) showed even more clearly that the lower risk of coronary disease was associated primarily with the intake of vitamin E from supplements rather than from the diet. Subsequent analyses therefore focused on vitamin E supplementation.

The amount of vitamin E in multivitamins is typically 30 IU or less, whereas specific vitamin E supple-

Table 1. Age-Adjusted Relative Risks of Major Coronary Heart Disease, According to Quintile Group for Total Vitamin E Intake and Intake of Vitamin E from Dietary Sources.*

Variable	Quintile Group for Vitamin E Intake 1	2	3	4	5	P Value for Trend
**Total intake (including supplements)**						
Median (IU/day)	2.8	4.2	5.9	17	208	—
Range (IU/day)	1.2–3.5	3.6–4.9	5.0–8.0	8.1–21.5	21.6–1000	—
Age-adjusted relative risk	1.0	0.90	1.00	0.68	0.59	—
95% Confidence interval	—	0.70–1.16	0.78–1.27	0.52–0.89	0.45–0.78	<0.001
Relative risk adjusted for age and smoking	1.0	1.00	1.15	0.74	0.66	—
95% Confidence interval	—	0.78–1.28	0.90–1.48	0.57–0.98	0.50–0.87	<0.001
**Dietary intake (without supplements)**						
Median (IU/day)	2.6	3.6	4.4	5.4	7.7	—
Range (IU/day)	0.3–3.1	3.2–3.9	4.0–4.8	4.9–6.2	6.3–100	—
Age-adjusted relative risk	1.0	0.97	0.77	0.98	0.79	—
95% Confidence interval	—	0.75–1.26	0.59–1.01	0.77–1.26	0.61–1.03	0.12
Relative risk adjusted for age and smoking	1.0	1.04	0.87	1.14	0.95	—
95% Confidence interval	—	0.80–1.35	0.66–1.14	0.89–1.47	0.72–1.23	0.99

*Major heart disease includes nonfatal myocardial infarction and death due to coronary disease.

The New England Journal of Medicine
Downloaded from nejm.org at NORTHEASTERN UNIVERSITY on November 18, 2017. For personal use only. No other uses without permission.
Copyright © 1993 Massachusetts Medical Society. All rights reserved.

ments usually contain 100 IU or more. After adjustment for age and smoking status in separate stratified analyses, both vitamin E supplements and multivitamins were associated with a lower risk of major coronary disease. The relative risk for women who took multivitamins was 0.78 (95 percent confidence interval, 0.64 to 0.96), and for those who took vitamin E supplements it was 0.57 (95 percent confidence interval, 0.41 to 0.78), as compared with the risk in those who took neither.

The women who took multivitamins or vitamin E supplements differed somewhat from those who took neither. Table 2 shows the age-standardized percentages and means for various known or suspected coronary risk factors, according to the use of multivitamins or vitamin E. Among women who took supplements there was a higher proportion of postmenopausal hormone users, vigorous exercisers, and nonsmokers. The magnitude of the overall differences was modest, however. For example, the 5.2 percent difference in hormone use between women who took both multivitamins and vitamin E supplements and those who took neither would account for a risk reduction of just 2.5 percent.[24]

Table 3 summarizes the multivariate models that controlled simultaneously for risk factors including age, body-mass index, smoking status, alcohol intake, menopausal status, postmenopausal hormone use, vigorous activity, regular use of aspirin, hypertension, high cholesterol level, diabetes, total energy intake, use of vitamin E supplements, and use of multivitamin supplements. The relative risk associated with the use of specific vitamin E supplements was 0.63 (95 percent confidence interval, 0.45 to 0.88). Those who took multivitamins were also at lower risk, although this association was not statistically significant.

Because users of both vitamin E and multivitamin supplements had a somewhat higher intake of other antioxidant nutrients, we repeated the multivariate analyses and controlled for the intake of carotene and vitamin C (including that consumed as supplements). This adjustment slightly attenuated the apparent benefit of vitamin E, but the association remained statistically significant with a relative risk of 0.69 (95 percent confidence interval, 0.49 to 0.97).

The best evidence for a mechanism by which vitamin E could reduce coronary disease is of a reduction in atherosclerosis. We therefore reasoned that the short-term use of vitamin E supplements would be associated with little reduction in risk. Users of vitamin E for less than two years had no significant reductions in risk, with a relative risk of 0.86 (95 percent confidence interval, 0.52 to 1.43). Use of vitamin E for two or more years, however, was associated with a decrease in risk of 41 percent (62 to 9 percent) even after adjustment for the intake of other antioxidants. We found no significant trend toward lower risk for periods of more than two years, but because of the small numbers of long-term users, we had little statistical power to detect such a trend if one were present. Users of vitamin E for 15 or more years had a relative risk of 0.59 (95 percent confidence interval, 0.14 to 2.39).

When we adjusted for coronary risk factors and excluded women who used vitamin E supplements for less than two years, we found that a dose of less than 100 IU per day was associated with little or no apparent benefit, but the confidence intervals were broad; the relative risk was 0.93 (95 percent confidence interval, 0.23 to 3.75). There was no suggestion of a trend toward a greater decrease in risk with higher daily doses; the relative risks were 0.56 (95 percent confidence interval, 0.21 to 1.51) for doses of 100 to 250 IU per day, 0.56 (95 percent confidence interval, 0.33 to 0.96) for doses of 300 to 500 IU per day, and 0.58 (95 percent confidence interval, 0.24 to 1.42) for doses of 600 or more IU per day. The results in models that omitted both the lowest dose and the shortest duration of vitamin E use are shown in Table 3.

To distinguish further the effect of vitamin E supplements from that of multivitamins, we compared the risk among women who used only vitamin E supplements, women who used only multivitamins, women who used both, and women who used neither (the reference group). After omitting women who used vitamin E supplements in low doses and for short periods and adjusting for the factors shown in Table 3, we found a relative risk of 0.41 (95 percent confidence

Table 2. Exposure to Various Risk Factors for Coronary Heart Disease, According to Type of Vitamin Use in 1984, after Standardization for Age.*

Variable	Type of Vitamins Used			
	None	Multivitamins Alone	Vitamin E Alone	Vitamin E Plus Multivitamins
Percentage of cohort	62.0	22.8	4.9	10.3
Mean age (yr)	49.8	50.0	51.5	51.1
Current smoker (%)	27.7	24.9	23.9	22.7
Former smoker (%)	28.0	30.6	32.5	33.5
High blood pressure (%)†	21.4	21.5	19.1	19.0
Diabetes (%)	2.8	2.9	2.8	2.7
High cholesterol (%)†	7.6	8.3	8.6	9.2
Family history of MI before age 60 (%)‡	18.4	17.7	16.2	17.5
Quetelet index§	25.3	24.6	24.4	24.5
Saturated fat (g/day)	28.0	27.7	26.7	26.8
Polyunsaturated fat (g/day)	9.3	9.2	8.9	9.0
Caloric intake (kcal/day)	1576	1588	1542	1569
Cholesterol (mg/day)	326	334	331	340
Alcohol (g/day)	6.3	7.0	6.9	6.9
Dietary fiber (g/day)	16.1	16.6	17.0	17.6
Current use of postmenopausal hormones (%)	11.8	15.7	15.6	17.0
Vigorous activity ≥1 time/wk (%)	61.1	66.3	67.2	69.6
Aspirin (regular use) (%)	57.0	47.7	49.3	49.8

*Except for the data on mean age, all data shown are standardized to the age distribution of the entire cohort.

†Determined on the basis of the women's reports of physicians' diagnoses of this condition.

‡MI denotes myocardial infarction.

§Calculated as the weight in kilograms divided by the square of the height in meters.

The New England Journal of Medicine
Downloaded from nejm.org at NORTHEASTERN UNIVERSITY on November 18, 2017. For personal use only. No other uses without permission.
Copyright © 1993 Massachusetts Medical Society. All rights reserved.

Table 3. Relative Risks of Major Coronary Heart Disease, According to the Use of Multivitamin and Vitamin E Supplements, with Adjustment for Age and Coronary Risk Factors.*

Variable	Vitamin E Supplements		Multivitamins	
	users	nonusers	users	nonusers
No. of cases of coronary disease	49	503	152	400
No. of person-years	93,921	585,564	231,310	448,175
	relative risk associated with vitamin use (95% confidence interval)			
Crude risk (unadjusted)	0.61 (0.45–0.81)		0.74 (0.61–0.89)	
Adjusted for age and smoking status†	0.57 (0.41–0.78)		0.78 (0.64–0.96)	
Basic model	0.63 (0.45–0.88)		0.87 (0.70–1.07)	
Adjusted further for intake of carotene and vitamin C	0.69 (0.49–0.97)		0.92 (0.74–1.15)	
Excluding vitamin E use for <2 yr	0.54 (0.36–0.82)		0.88 (0.70–1.09)	
Excluding vitamin E use for <2 yr and adjusted further for intake of carotene and vitamin C	0.59 (0.38–0.91)		0.91 (0.72–1.14)	
Excluding vitamin E use for <2 yr and doses <100 IU/day	0.52 (0.34–0.80)		0.88 (0.71–1.10)	
Excluding vitamin E use for <2 yr and doses <100 IU/day, and adjusted further for intake of carotene and vitamin C	0.57 (0.36–0.89)		0.92 (0.73–1.15)	

*Adjusted for age (in five-year categories), time period, Quetelet index (in five categories), smoking (never smoked, formerly smoked, or currently smoking 1 to 14, 15 to 24, 25 to 34, or 35 or more cigarettes per day), alcohol intake (none, or 0.1 to 4.9, 5.0 to 14.9, or 15 or more grams per day), menopausal status (premenopausal, postmenopausal, or status uncertain), postmenopausal hormone use (never, formerly, or currently), exercise (works up a sweat less than once per week or once or more per week), regular use of aspirin, hypertension, high cholesterol level, diabetes, total energy intake (in quintiles), use of vitamin E supplements, and use of multivitamin supplements.

†All the women were free of diagnosed cardiovascular disease and cancer (except nonmelanoma skin cancer) at the start of the observation period in 1980. In this and each subsequent model, women who had these diagnoses at the start of each two-year questionnaire cycle were excluded from further follow-up.

interval, 0.18 to 0.93) for users of vitamin E supplements only, 0.87 (95 percent confidence interval, 0.69 to 1.09) for users of multivitamins only, and 0.50 (95 percent confidence interval, 0.31 to 0.83) for users of both. In separate multivariate analyses, the relative risk for specific vitamin E use among the multivitamin users was 0.58 (95 percent confidence interval, 0.35 to 0.97); among nonusers of multivitamins, it was 0.46 (95 percent confidence interval, 0.22 to 0.98).

We observed no marked differences between subgroups with respect to the association of vitamin E supplements with lower risk. Some investigators have suggested that antioxidants might be more important among cigarette smokers, but we found similar reductions in risk among vitamin E users who smoked (relative risk, 0.55; 95 percent confidence interval, 0.30 to 1.02) and those who did not (relative risk, 0.52; 95 percent confidence interval, 0.29 to 0.90). Adjustment for the intake of saturated, monounsaturated, or polyunsaturated fat and analyses of different categories of fat intake had no material effect. Because the diagnosis of diabetes or high cholesterol level could alter diet, we performed analyses in which women with those conditions were excluded; we also performed analyses in which there was no exclusion for prevalent disease at the base line of each two-year follow-up period. The findings from these alternate analyses were virtually the same as those for the whole cohort.

In further analyses, we explored the effect of vitamin E supplementation on other cardiovascular outcomes and overall mortality (Table 4). For none of the categories, including overall mortality, was the association as pronounced as for major coronary disease, which was a priori the main hypothesis. However, there were suggestive trends for all the outcomes.

Discussion

In this large prospective study, we observed a risk of major coronary disease among women who took vitamin E supplements that was about 40 percent lower than the risk in women who did not take these supplements; this association changed little after adjustment for coronary risk factors and the intake of other dietary antioxidants. The prospective study design eliminated the potential for biased recall of vitamin E use, and because the follow-up rate was high, it is unlikely that differential follow-up could have affected the results materially.

Information on vitamin use and other potential risk factors was reported by the women, but we believe it to be reliable. Reports of various conditions have been validated by review of the medical records and by direct measurement.[27,42] Also, the risk factors reported by the women were strong predictors of subsequent cardiovascular disease,[24,43-45] and all the participants were registered nurses with a demonstrated interest in medical research.

Perhaps healthier women select themselves for vitamin supplementation. Nurses who took vitamin E had a somewhat better risk profile than those who did not, but the differences were not striking. Adjustment for a wide range of risk factors had only a modest effect on the estimates of relative risk (from 0.61 to 0.63) (Table 3), suggesting only minor confounding by the risk factors measured. Some unmeasured factor might be a confounding variable, but it would have to be both highly associated with use of vitamin E supplements and a very strong risk factor to explain these results.

Although we cannot rule out such self-selection as an explanation, other lines of evidence also support a

Table 4. Relative Risks for Cardiovascular Outcomes, According to the Use of Multivitamins and Vitamin E Supplements, after Adjustment for Age and Coronary Risk Factors.*

Outcome	No. of Cases	Vitamin E Supplements	Multivitamins
		relative risk (95% confidence interval)	
Major coronary disease	440†	0.54 (0.36–0.82)	0.88 (0.70–1.09)
Cardiovascular mortality	195	0.58 (0.30–1.12)	0.77 (0.58–1.16)
Ischemic stroke	154	0.71 (0.39–1.31)	0.86 (0.59–1.24)
Coronary-artery surgery‡	366	0.73 (0.48–1.09)	0.76 (0.59–0.97)
Overall mortality	974	0.87 (0.69–1.10)	1.10 (0.94–1.27)

*The risk factors included in the adjustment are listed in the first note to Table 3. All analyses in this table were made after the exclusion of cancer and cardiovascular disease at the start of each two-year period and the exclusion of women who had taken vitamin E supplements for less than two years.

†This number is lower than the total of 552 because of the additional criteria for exclusion used in these analyses.

‡Includes coronary-artery bypass grafting and percutaneous transluminal coronary angioplasty.

The New England Journal of Medicine
Downloaded from nejm.org at NORTHEASTERN UNIVERSITY on November 18, 2017. For personal use only. No other uses without permission.
Copyright © 1993 Massachusetts Medical Society. All rights reserved.

cause-and-effect relation. If self-selection were the entire explanation, one would expect to have observed a similar apparent benefit among short-term users of vitamin E and users of other vitamin supplements, such as multivitamins and vitamin C. In multivariate analyses (Table 3), a significant risk reduction was observed for vitamin E, but not for multivitamin supplements. Also, further analysis indicated a lower risk among the women who took vitamin E, but not among those who took vitamin C, even in the highest quintile, which included only users of specific vitamin C supplements. We had insufficient statistical power to test adequately for a trend toward increased protection with longer use. The lack of a dose–response association is not surprising, because blood levels do not rise in a linear fashion with increasing doses of vitamin E.[36] The modest trends toward reduced risk among users of multivitamins may possibly be explained by an effect of folate and vitamin B_6 to reduce otherwise elevated levels of homocyst(e)ine in a subgroup.[46]

As with postmenopausal estrogens,[24] we did not observe the same magnitude of effect of vitamin E on cardiovascular outcomes other than major coronary disease. Although the findings were not statistically significant, there were trends toward a reduction in the risk of mortality from cardiovascular causes, ischemic stroke, coronary-artery surgery, and overall mortality.

A benefit of vitamin E in reducing the risk of major coronary heart disease is plausible because of the substantial evidence indicating the importance of oxidation of LDL in atherosclerosis.[1-3,47,48] No association was observed between plasma levels of vitamin E and resistance to LDL oxidation among nonusers of supplements, but vitamin E supplementation markedly increased the resistance.[18] This finding is consistent with our results in suggesting that supplementation at levels far higher than those achievable by diet alone may be needed to reduce LDL oxidation. In a randomized trial in monkeys fed an atherogenic diet, animals given vitamin E supplements had less arterial stenosis than those given placebo,[49] but the study was small and the results somewhat inconsistent.

Epidemiologic data on this research question are sparse. Gey et al. observed that in regions of Europe with high rates of coronary disease, the mean plasma levels of vitamin E were lower than in other regions,[50] but this association could also be due to other differences. Although Salonen et al. found no association between plasma levels of vitamin E and coronary disease,[51] there were few users of vitamin E supplements in the population. In a case–control study of angina, Riemersma et al.[52] observed a statistically significant relative risk of 0.37 when the highest and the lowest quintiles for plasma vitamin E intake were compared; no significant associations were seen for plasma levels of vitamin C or carotene. In a small, four-month randomized trial of vitamin E among patients undergoing angioplasty, restenosis was reduced by 25 percent in the treatment group, but the result was not statistically significant.[21]

Rimm et al.[53] have reported findings in men that were very similar to those we observed, using a similar prospective design. As in our study, those findings could not be explained by confounding by other coronary risk factors or dietary variables.

Although we cannot exclude the possibility of residual confounding, the consistency of our findings, the biologic plausibility, and the similar results from another prospective cohort[53] all support the possibility of a causal explanation and suggest that vitamin E supplements may reduce the risk of coronary heart disease. Randomized trials of adequate size in secondary and primary prevention will be needed to test this question.[3] Because vitamin E appears not to be toxic, even in high doses,[54] such trials, notably the Women's Health Study,[55] have been initiated. Public policy recommendations about the widespread use of vitamin E should await the results of these trials.

We are indebted to the participants in the Nurses' Health Study for their continuing outstanding level of cooperation; to Frank E. Speizer, M.D., overall principal investigator for the study; and to Mark Shneyder, Stefanie Bechtel, Gary Chase, Karen Corsano, Kate Saunders, Lisa Dunn, Barbara Egan, Lori Ward, and Marion McPhee for their unfailing help.

References

1. Steinberg D, Parthasarathy S, Carew TE, Khoo JC, Witztum JL. Beyond cholesterol: modifications of low-density lipoprotein that increase its atherogenicity. N Engl J Med 1989;320:915-24.
2. Steinberg D, Witztum JL. Lipoproteins and atherogenesis: current concepts. JAMA 1990;264:3047-52.
3. Steinberg D. Antioxidants and atherosclerosis: a current assessment. Circulation 1991;84:1420-5.
4. Quinn MT, Parthasarathy S, Fong LG, Steinberg D. Oxidatively modified low density lipoproteins: a potential role in recruitment and retention of monocyte/macrophages during atherogenesis. Proc Natl Acad Sci U S A 1987;84:2995-8.
5. Quinn MT, Parthasarathy S, Steinberg D. Endothelial cell-derived chemotactic activity for mouse peritoneal macrophages and the effects of modified forms of low-density lipoprotein. Proc Natl Acad Sci U S A 1985;82:5949-53.
6. Morel DW, DiCorleto PE, Chisolm GM. Endothelial and smooth muscle cells alter low density lipoprotein in vitro by free radical oxidation. Arteriosclerosis 1984;4:357-64.
7. Kugiyama K, Kerns SA, Morrisett JD, Roberts R, Henry PD. Impairment of endothelium-dependent arterial relaxation by lysolecithin in modified low-density lipoproteins. Nature 1990;344:160-2.
8. Palinski W, Rosenfeld ME, Ylä-Herttuala S, et al. Low-density lipoprotein undergoes oxidative modification in vivo. Proc Natl Acad Sci U S A 1989; 86:1372-6.
9. Rosenfeld ME, Palinski W, Ylä-Herttuala S, Butler S, Witztum JL. Distribution of oxidation specific lipid-protein adducts and apolipoprotein B in atherosclerotic lesions of varying severity from WHHL rabbits. Arteriosclerosis 1990;10:336-49.
10. Ylä-Herttuala S, Palinski W, Rosenfeld ME, et al. Evidence for the presence of oxidatively modified low density lipoprotein in atherosclerotic lesions of rabbit and man. J Clin Invest 1989;84:1086-95.
11. Salonen JT, Ylä-Herttuala S, Yamamoto R, et al. Autoantibody against oxidised LDL and progression of carotid atherosclerosis. Lancet 1992;339: 883-7.
12. Stringer MD, Görög PG, Freeman A, Kakkar VV. Lipid peroxides and atherosclerosis. BMJ 1989;298:281-4.
13. Regnström J, Nilsson J, Tornvall P, Landou C, Hamsten A. Susceptibility to low-density lipoprotein oxidation and coronary atherosclerosis in man. Lancet 1992;339:1183-6.
14. Stocker R, Frei B. Endogenous antioxidant defenses in human blood plasma. In: Sies H, ed. Oxidative stress: oxidants and antioxidants. Orlando, Fla.: Academic Press, 1991:213-43.
15. Packer L. Protective role of vitamin E in biological systems. Am J Clin Nutr 1991;53:Suppl:1050S-1055S.
16. Boscoboinik D, Szewczyk A, Hensey C, Azzi A. Inhibition of cell proliferation by α-tocopherol: role of protein kinase C. J Biol Chem 1991;266: 6188-94.
17. Esterbauer H, Dieber-Rotheneder M, Striegl G, Waeg G. Role of vitamin E in preventing the oxidation of low-density lipoprotein. Am J Clin Nutr 1991; 53:Suppl:314S-321S.

The New England Journal of Medicine
Downloaded from nejm.org at NORTHEASTERN UNIVERSITY on November 18, 2017. For personal use only. No other uses without permission.
Copyright © 1993 Massachusetts Medical Society. All rights reserved.

18. Princen HMG, von Poppel G, Vogelezang C, Buytenhek R, Kok FJ. Supplementation with vitamin E but not β-carotene in vivo protects low density lipoprotein from lipid peroxidation in vitro: effect of cigarette smoking. Arterioscler Thromb 1992;12:554-62.
19. Livingstone PD, Jones C. Treatment of intermittent claudication with vitamin E. Lancet 1958;2:602-4.
20. Williams HTG, Fenna D, Macbeth RA. Alpha tocopherol in the treatment of intermittent claudication. Surg Gynecol Obstet 1971;132:662-6.
21. DeMaio SJ, King SB III, Lembo NJ, et al. Vitamin E supplementation, plasma lipids and incidence of restenosis after percutaneous transluminal coronary angioplasty (PTCA). J Am Coll Nutr 1992;11:68-73.
22. Haeger K. Long-time treatment of intermittent claudication with vitamin E. Am J Clin Nutr 1974;27:1179-81.
23. Gillilan RE, Mondell B, Warbasse JR. Quantitative evaluation of vitamin E in the treatment of angina pectoris. Am Heart J 1977;93:444-9.
24. Stampfer MJ, Colditz GA, Willett WC, et al. Postmenopausal estrogen therapy and cardiovascular disease: ten-year follow-up from the Nurses' Health Study. N Engl J Med 1991;325:756-62.
25. Willett WC, Stampfer MJ, Colditz GA, Rosner BA, Hennekens CH, Speizer FE. Dietary fat and the risk of breast cancer. N Engl J Med 1987;316:22-8.
26. Willett WC, Stampfer MJ, Underwood BA, Speizer FE, Rosner BR, Hennekens CH. Validation of a dietary questionnaire with plasma carotenoid and alpha-tocopherol levels. Am J Clin Nutr 1983;38:631-9.
27. Willett WC, Sampson L, Stampfer MJ, et al. Reproducibility and validity of a semiquantitative food frequency questionnaire. Am J Epidemiol 1985;122:51-65.
28. Willett WC, Stampfer MJ. Total energy intake: implications for epidemiologic analyses. Am J Epidemiol 1986;124:17-27.
29. Colditz GA, Willett WC, Stampfer MJ, et al. The influence of age, relative weight, smoking, and alcohol intake on the reproducibility of a dietary questionnaire. Int J Epidemiol 1987;16:392-8.
30. Willett WC, Sampson L, Browne ML, et al. The use of a self-administered questionnaire to assess diet four years in the past. Am J Epidemiol 1988;127:188-99.
31. Stryker WS, Kaplan LA, Stein EA, Stampfer MJ, Sober A, Willett WC. The relation of diet, cigarette smoking, and alcohol consumption to plasma beta-carotene and alpha-tocopherol levels. Am J Epidemiol 1988;127:283-96.
32. Hunter DJ, Sampson L, Stampfer MJ, Colditz GA, Rosner B, Willett WC. Variability in portion sizes of commonly consumed foods among a population of women in the United States. Am J Epidemiol 1988;127:1240-9.
33. Salvini S, Hunter DJ, Sampson L, et al. Food-based validation of a dietary questionnaire: the effects of week-to-week variation in food consumption. Int J Epidemiol 1989;18:858-67.
34. Romieu I, Stampfer MJ, Stryker WS, et al. Food predictors of plasma beta-carotene and alpha-tocopherol: validation of a food frequency questionnaire. Am J Epidemiol 1990;131:864-76.
35. Willett WC. Nutritional epidemiology. New York: Oxford University Press, 1990.
36. Willett WC, Stampfer MJ, Underwood BA, Taylor JO, Hennekens CH. Vitamins A, E, and carotene: effects of supplementation on their plasma levels. Am J Clin Nutr 1983;38:559-66.
37. IHD registers: report of the fifth working group. Copenhagen, Denmark: World Health Organization, 1971.
38. Stampfer MJ, Willett WC, Speizer FE, et al. Test of the National Death Index. Am J Epidemiol 1984;119:837-9.
39. Walker AE, Robins M, Weinfeld FD. The National Survey of Stroke: clinical findings. Stroke 1981;12:Suppl 1:I-13–I-44.
40. Rothman KJ, Boice JD Jr. Epidemiologic analysis with a programmable calculator. Washington, D.C.: Government Printing Office, 1979. (NIH publication no. (PHS) 70-1649.)
41. Cox DR. Regression models and life-tables. J R Stat Soc [B] 1972;34:187-220.
42. Colditz GA, Martin P, Stampfer MJ, et al. Validation of questionnaire information on risk factors and disease outcomes in a prospective cohort study of women. Am J Epidemiol 1986;123:894-900.
43. Colditz GA, Stampfer MJ, Willett WC, Rosner B, Speizer FE, Hennekens CH. A prospective study of parental history of myocardial infarction and coronary heart disease in women. Am J Epidemiol 1986;123:48-58.
44. Stampfer MJ, Colditz GA, Willett WC, Rosner B, Speizer FE, Hennekens CH. Coronary heart disease risk factors in women: the Nurses' Health Study experience. In: Eaker E, Packard B, Wenger NK, Clarkson TB, Tyroler HA, eds. Coronary heart disease in women. New York: Haymarket-Doyma, 1987:112-6.
45. Colditz GA, Nurses' Health Study Research Group. The Nurses' Health Study: findings during 10 years of follow-up of a cohort of U.S. women. Curr Probl Obstet Gynecol Fertil 1990;13:129-74.
46. Stampfer MJ, Malinow MR, Willett WC, et al. A prospective study of plasma homocyst(e)ine and risk of myocardial infarction in US physicians. JAMA 1992;268:877-81.
47. Kagan VE, Serbinova EA, Forte T, Scita G, Packer L. Recycling of vitamin E in human low density lipoproteins. J Lipid Res 1992;33:385-97.
48. Jialal I, Grundy SM. Effect of dietary supplementation with alpha-tocopherol on the oxidative modification of low density lipoprotein. J Lipid Res 1992;33:899-906.
49. Verlangieri AJ, Bush MJ. Effects of d-α-tocopherol supplementation on experimentally induced primate atherosclerosis. J Am Coll Nutr 1992;11:131-8.
50. Gey KF, Brubacher GB, Stähelin HB. Plasma levels of antioxidant vitamins in relation to ischemic heart disease and cancer. Am J Clin Nutr 1987;45:1368-77.
51. Salonen JT, Salonen R, Seppänen K, et al. Relationship of serum selenium and antioxidants to plasma lipoproteins, platelet aggregability and prevalent ischaemic heart disease in Eastern Finnish men. Atherosclerosis 1988;70:155-60.
52. Riemersma RA, Wood DA, Macintyre CCA, Elton RA, Gey KF, Oliver MF. Risk of angina pectoris and plasma concentrations of vitamins A, C, and E and carotene. Lancet 1991;337:1-5.
53. Rimm EB, Stampfer MJ, Ascherio A, Giovannucci E, Colditz GA, Willett WC. Vitamin E consumption and the risk of coronary heart disease in men. N Engl J Med 1993;328:1450-6.
54. Bendich A, Machlin LJ. Safety of oral intake of vitamin E. Am J Clin Nutr 1988;48:612-9.
55. Women's Health Study Research Group. The Women's Health Study: summary of the study design. J Myocardial Ischemia 1992;4:27-9.

The New England Journal of Medicine
Downloaded from nejm.org at NORTHEASTERN UNIVERSITY on November 18, 2017. For personal use only. No other uses without permission.
Copyright © 1993 Massachusetts Medical Society. All rights reserved.
