# Supplementary material for: A systematic comprehensive longitudinal evaluation of dietary factors associated with acute myocardial infarction and fatal coronary heart disease
Source: Nat Commun. 2020 Nov 27;11:6074. doi: 10.1038/s41467-020-19888-2 (PMC7699643; doi:10.1038/s41467-020-19888-2)
Supplement: Supplementary file 4 — Supplementary Software [file 41467_2020_19888_MOESM4_ESM.zip › EWAS-NHS-master/KG-SI/Rest/Wolk (1999).pdf]

Long-term Intake of Dietary Fiber and Decreased Risk of Coronary Heart Disease Among Women

Alicja Wolk, DMSc
JoAnn E. Manson, MD
Meir J. Stampfer, MD
Graham A. Colditz, MD
Frank B. Hu, MD
Frank E. Speizer, MD
Charles H. Hennekens, MD
Walter C. Willett, MD

Context Epidemiological studies of men suggest that dietary fiber intake protects against coronary heart disease (CHD), but data on this association in women are sparse.

Objective To examine the association between long-term intake of total dietary fiber as well as fiber from different sources and risk of CHD in women.

Design and Setting The Nurses' Health Study, a large, prospective cohort study of US women followed up for 10 years from 1984. Dietary data were collected in 1984, 1986, and 1990, using a validated semiquantitative food frequency questionnaire.

Participants A total of 68 782 women aged 37 to 64 years without previously diagnosed angina, myocardial infarction (MI), stroke, cancer, hypercholesterolemia, or diabetes at baseline.

Main Outcome Measure Incidence of acute MI or death due to CHD by amount of fiber intake.

Results Response rate averaged 80% to 90% during the 10-year follow-up. We documented 591 major CHD events (429 nonfatal MIs and 162 CHD deaths). The age-adjusted relative risk (RR) for major CHD events was 0.53 (95% confidence interval [CI], 0.40-0.69) for women in the highest quintile of total dietary fiber intake (median, 22.9 g/d) compared with women in the lowest quintile (median, 11.5 g/d). After controlling for age, cardiovascular risk factors, dietary factors, and multivitamin supplement use, the RR was 0.77 (95% CI, 0.57-1.04). For a 10-g/d increase in total fiber intake (the difference between the lowest and highest quintiles), the multivariate RR of total CHD events was 0.81 (95% CI, 0.66-0.99). Among different sources of dietary fiber (eg, cereal, vegetables, fruit), only cereal fiber was strongly associated with a reduced risk of CHD (multivariate RR, 0.63; 95% CI, 0.49-0.81 for each 5-g/d increase in cereal fiber).

Conclusions Our findings in women support the hypothesis that higher fiber intake, particularly from cereal sources, reduces the risk of CHD.

JAMA. 1999;281:1998-2004 www.jama.com

CORONARY HEART DISEASE (CHD) is the leading cause of death among women.[1-4] Epidemiological studies of men suggest that dietary fiber or foods rich in fiber, especially from cereal products, protect against CHD[5-17] but data on women are sparse.[18-20]

High intake of dietary fiber, especially soluble fiber, decreases low-density lipoprotein cholesterol and has little or no effect on high-density lipoprotein cholesterol.[21,22] However, the effect of soluble fiber on serum cholesterol level is modest and could not account for a substantial reduction in CHD incidence. Diets high in dietary fiber also may have other beneficial physiological effects, such as increased insulin sensitivity, lower triglyceride levels, and favorable effects on plasminogen activator type 1 and factor VII activity.[23-33]

We examined in detail the relation between intake of dietary fiber and risk of CHD in the Nurses' Health Study, a large, prospective cohort of women. Estimates of fiber intake were based on 3 dietary measurements during 10 years of follow-up, thus providing a measure of long-term consumption.

See also Patient Page.

METHODS

The Nurses' Health Study Cohort

The Nurses' Health Study cohort was established in 1976, when 121 700 female registered nurses aged 30 to 55 years residing in 11 large US states com-

Author Affiliations: Department of Medical Epidemiology, Karolinska Institutet, Stockholm, Sweden (Dr Wolk); the Channing Laboratory (Drs Manson, Stampfer, Colditz, Speizer, Hennekens, and Willett) and the Division of Preventive Medicine (Drs Manson and Hennekens), Department of Medicine, Brigham and Women's Hospital and Harvard Medical School, the Departments of Environmental Science (Dr Speizer), Epidemiology (Drs Manson, Stampfer, Colditz, Hennekens, and Willett), and Nutrition (Drs Stampfer, Hu, and Willett), Harvard School of Public Health, and the Department of Ambulatory Care and Prevention (Dr Hennekens), Harvard Medical School, Boston, Mass.
Corresponding Author and Reprints: Alicja Wolk, DMSc, Department of Medical Epidemiology, Karolinska Institutet, Box 281, SE-171 77, Stockholm, Sweden (e-mail: alicja.wolk@mep.ki.se).

 ©1999 American Medical Association. All rights reserved.

Downloaded From: https://jamanetwork.com/ by a Northeastern University Libraries User on 05/23/2019

pleted a mailed questionnaire on their medical history and lifestyle. Every 2 years, follow-up questionnaires have been sent to update information on potential risk factors and identify newly diagnosed cases of CHD and other diseases. In 1980, a 61-item food frequency questionnaire was included to assess dietary intake. In 1984, the food frequency questionnaire was expanded to include 116 items and similar questionnaires were used to update diet information in 1986 and 1990. Because the revised questionnaires contained additional detail critical for assessing sources of fiber intake, we used the 1984 questionnaire completed by 81 757 women as baseline. We excluded women with 11 or more food questionnaire items blank, total energy intake of less than 2761 kJ/d or more than 14 644 kJ/d, or previously diagnosed cancer, angina, myocardial infarction (MI), stroke, or other cardiovascular disease.

Women reporting a diagnosis of high serum cholesterol level or diabetes were also excluded because these disorders are associated with increased risk of CHD and also could have caused women to change their fiber intake. The final 1984 baseline population consisted of 68 782 women. On average, more than 90% responded to each subsequent biennial questionnaire[34] and about 80% completed each repeated dietary questionnaire during follow-up periods.

Dietary Assessment

To estimate intakes of dietary fiber and other nutrients, a commonly used unit or portion size for each food was specified in the food frequency questionnaire, and the participant was asked how often during the previous year, on average, she had consumed that amount. Nine responses were possible, ranging from "never" to "6 or more times per day." In addition to the structured questions on food intake, we asked participants to report their most commonly used specific brand and type of cold breakfast cereal, multivitamin supplement, and cooking oil. Intake of nutrients was computed by multiplying the frequency of consumption of each food item by the fiber content of the specified portions,[35-38] taking into account the specific type of breakfast cereal. The dietary fiber content of foods was obtained from US Department of Agriculture (USDA) sources[35] or information from manufacturers based on the Association of Official Analytical Chemists' method.[36-38] We adjusted fiber intake for total energy using regression analysis.[39] The reproducibility and validity of the food frequency questionnaires have been described in detail elsewhere.[40-42] The Pearson correlation coefficient between total dietary fiber intake assessed by the 116-item food frequency questionnaire and assessed by 4 one-week diet records was 0.60.

Identification of CHD Cases

The primary end point for this study was CHD, defined as symptomatic nonfatal MI or fatal CHD that occurred after the return of the 1984 questionnaire but before June 1, 1994. We requested permission to review medical records from women reporting a nonfatal MI on a follow-up questionnaire. The records were reviewed by study physicians with no knowledge of the self-reported risk factor status. Nonfatal MIs were confirmed if they met the World Health Organization's criteria that include symptoms plus either diagnostic electrocardiographic changes or elevated cardiac enzyme levels.[43] Deaths were identified from the National Death Index, next of kin, or the postal system. Using all sources combined, we estimated that follow-up for the deaths was more than 98% complete.[44] Fatal CHD was confirmed by hospital records or autopsy report or if CHD was listed as the cause of death on the certificate and evidence of previous CHD was available. We also included sudden death within 1 hour of onset of symptoms in women with no other plausible causes other than CHD (12.3% of fatal CHD). We identified 429 women with nonfatal MI and 162 women with fatal CHD during the 10-year follow-up.

Data Analysis

Person-time for each participant was calculated from the date of return of the 1984 questionnaire to the date of first CHD event, death, or June 1, 1994. Women who reported coronary events, stroke, or cancer on previous questionnaires were excluded from subsequent follow-up; thus, each participant could contribute only 1 end point and the cohort at risk included only those who remained free from cardiovascular disease and cancer at the beginning of each follow-up interval.

To reduce within-subject variation and best represent long-term diet, we used the updated cumulative average dietary fiber intake from all available dietary questionnaires up to the start of each 2-year follow-up interval.[45] Because women who develop intermediate end points, such as angina, hypercholesterolemia, diabetes, and hypertension, may change their diets, which could confound diet-disease associations,[46] we continued to follow these women but stopped updating dietary exposures at the beginning of the interval during which these intermediate end points occurred. When dietary data were missing at the start of an interval, either because we had stopped updating because of the occurrence of an intermediate end point or because a dietary questionnaire had not been returned, we carried forward the last available dietary data. Nondietary covariates, including age, cigarette smoking, body mass index (weight in kilograms divided by the square of height in meters), menopausal hormone use, multivitamin supplement use, and vitamin E supplement use, were updated biennially. Aspirin use was assessed in 1984 and 1988, and vigorous exercise performance was assessed in 1980.

Women were grouped into quintiles of energy-adjusted intake of dietary fiber, using the updated cumulative average of intake. The relative risk (RR) was computed as the rate in a specific category of dietary fiber intake divided by that in the lowest quintile, with adjustment for 5-year age categories. A multivariate pooled logistic model[47] was used to estimate the RR (with 95% confidence interval [CI]) of CHD associated with various intakes of dietary fiber and foods that were primary sources

©1999 American Medical Association. All rights reserved.

Downloaded From: https://jamanetwork.com/ by a Northeastern University Libraries User on 05/23/2019

of fiber in this cohort, simultaneously adjusting for other risk factors. Tests of linear trend across increasing categories of total dietary fiber were conducted by treating the categories as a continuous variable and assigning the median intake for the category as its value. All *P* values are 2-sided.

RESULTS

During 10 years of follow-up, including 641 515 person-years of observation, we documented 591 cases of CHD (429 women with nonfatal MI and 162 CHD deaths). The mean (SD) energy-adjusted daily intake of total dietary fiber was 16.2 (4.8) g in 1984, 17.5 (5.3) g in 1986, and 18.0 (5.5) g in 1990. There was an approximately 2-fold difference in the median total dietary fiber intake between the highest and lowest quintiles of the population at baseline (TABLE 1) and according to the updated cumulative average (22.9 g/d vs 11.5 g/d).

Table 1. Age-Standardized Baseline Characteristics Across Quintiles of Dietary Fiber Intake in 1984 Among 68 782 Nurses Aged 37 to 64 Years*

	Quintiles of Energy-Adjusted Fiber Intake					
	Total Dietary Fiber			Cereal Fiber		
Characteristics	1	3	5	1	3	5
Dietary fiber, median, g/d	10.9	15.7	22.6	1.9	3.6	7.8
Age, mean, y	48.4	49.6	52.1	49.8	49.2	51.5
Current smokers, %	38.6	22.3	14.0	35.6	23.5	14.2
Current postmenopausal hormone users, %	9.7	10.4	12.3	10.0	10.2	12.5
Parental myocardial infarction <60 y, %	14.2	14.7	14.5	14.5	14.3	13.9
History of hypertension, %	19.8	18.3	18.2	21.8	17.8	16.3
Multivitamin supplement use, %	32.6	36.2	43.0	34.3	35.4	41.7
Vitamin E supplement use, %	12.6	15.2	23.8	14.9	15.7	21.4
Aspirin use ≥ once per week, %	29.2	27.2	31.4	30.0	27.0	28.8
Vigorous exercise ≥ once per week, %	34.7	43.4	53.3	40.9	42.6	47.9
Body mass index, kg/m^2	24.8	24.9	24.4	24.9	24.9	24.2
Alcohol, g/d	11.8	6.4	4.4	12.0	6.3	4.6
Protein, g/d	68.5	71.3	73.7	72.5	70.3	71.1
Fat, g/d						
Total	65.1	62.7	55.0	63.5	62.4	57.3
Saturated	24.4	22.6	19.1	23.6	22.5	20.2
Monounsaturated	24.1	23.0	19.6	23.3	22.9	20.6
Linoleic acid	9.9	10.3	9.7	9.8	10.3	9.9
Trans	3.7	3.5	2.8	3.2	3.5	3.2
Cholesterol, mg/d	303	289	263	306	288	259
Carbohydrates, g/d	168	183	207	170	185	202
Dietary vitamin C, mg/d	103	133	178	141	133	136
Dietary vitamin B_6, mg/d	1.4	1.7	2.1	1.7	1.7	1.9
Dietary vitamin E, mg/d	5.5	6.4	7.5	6.1	6.5	6.6
Dietary folate, mg/d	207	266	361	255	262	317
Dietary beta carotene, mg/d	2322	3910	6886	4096	4096	4449
Magnesium, mg/d	243	279	353	270	273	336
Dietary fiber, g/d						
Total	10.6	15.8	23.7	14.8	15.6	20.2
Cereal	2.8	3.9	6.0	1.9	3.6	8.0
Vegetable	4.0	6.0	9.2	6.3	6.1	6.3
Fruit	1.6	3.3	6.0	3.3	3.4	3.9
Soluble fiber	3.2	4.5	6.8	4.3	4.9	5.7
Insoluble fiber	7.4	11.3	16.9	9.6	11.5	15.5

*Data are presented as mean intake or percentage unless otherwise noted. For total dietary fiber intake, the Pearson correlation coefficient was 0.60 for intake in 1984 vs 1986, 0.51 for intake in 1984 vs 1986, and 0.54 for intake in 1986 vs 1990.

In 1984, approximately 38% of dietary fiber intake was from vegetables; 25%, from cereals; and 22%, from fruits. Cold breakfast cereals contributed approximately 6.4% of the total dietary fiber intake; dark bread, 3.9%; and white bread, 2.2%.

In age-adjusted analyses (TABLE 2), women in the highest quintile of long-term (cumulative average) total dietary fiber intake had a 43% lower risk of nonfatal MI and a 59% lower risk of fatal coronary disease in comparison with the lowest quintile. To increase the stability of risk estimates, we combined fatal and nonfatal CHD events for multivariate analyses. After adjustment for nondietary cardiovascular risk factors and total energy, alcohol, saturated fat, and carbohydrate intake, this risk was 23% lower for the highest compared with the lowest quintile. Cigarette smoking accounted for most of the difference between the age-adjusted and multivariate analyses.

To facilitate comparisons with other studies, we also analyzed total dietary fiber as a continuous variable. From the multivariate analysis (adjustments specified in Table 2), each 10-g increment in long-term total fiber intake corresponded to an RR of total CHD of 0.81 (95% CI, 0.66-0.99). Further simultaneous control for dietary folate, beta carotene, vitamin B_6, vitamin C, vitamin E, magnesium, and *trans* fat intake did not appreciably alter this RR but the confidence interval widened (RR, 0.79; 95% CI, 0.59-1.07).

The physiological effects of fiber may depend on type of fiber. In multivariate analyses of total CHD, RRs were 0.75 (95% CI, 0.53-1.07) for a 5-g/d increase in soluble fiber and 0.88 (95% CI, 0.77-1.01) for a 5-g/d increase in insoluble fiber. Because the correlation between the 2 types of fiber was 0.78, they could not be distinguished in multivariate analysis.

We evaluated separately the effects of 3 main food sources of dietary fiber, namely, cereal, vegetable, and fruit fiber (TABLE 3). In a multivariate analysis, we found that women in the highest compared with the lowest category

 ©1999 American Medical Association. All rights reserved.

Downloaded From: https://jamanetwork.com/ by a Northeastern University Libraries User on 05/23/2019

of long-term cereal fiber intake had a 34% lower risk of total CHD. Intake of vegetable and fruit fiber was not appreciably associated with risk of total CHD. We further analyzed specific food sources of fiber in continuous form, using a 5-g increment, approximately corresponding to the difference between medians in the highest and lowest quintiles of long-term intake. After simultaneous adjustment for each of the 3 main sources of fiber, only the RR for cereal fiber (multivariate RR, 0.63; 95% CI, 0.49-0.81) was statistically significant.

We investigated whether age, smoking status, body mass index, or intake of saturated or *trans* fat might modify the relation of cereal fiber to risk of total CHD. As shown in TABLE 4, the inverse associations with cereal fiber were seen in all subgroups, but these were strongest in never or past smokers, leaner women, women younger than 60 years, those in the lowest tertile of satu-

Table 2. Relative Risk of CHD by Quintiles of Long-term Dietary Fiber Intake Among Women During 10 Years of Follow-up*

	Quintiles of Energy-Adjusted Long-term Total Dietary Fiber Intake, 1984-1990					
	1	2	3	4	5	*P* Value for Trend
Fiber intake, median for 1984-1990, g/d	11.5	14.3	16.4	18.8	22.9	. . .
Person-years, No.	134 707	133 824	130 654	124 522	117 808	. . .
Nonfatal MI						
No.	110	93	81	73	72	. . .
Age-adjusted RR (95% CI)	1.0 (Referent)	0.80 (0.61-1.06)	0.68 (0.51-0.90)	0.57 (0.42-0.77)	0.57 (0.42-0.77)	<.001
Fatal CHD						
No.	38	34	33	34	23	. . .
Age-adjusted RR (95% CI)	1.0 (Referent)	0.83 (0.52-1.31)	0.74 (0.46-1.18)	0.73 (0.46-1.16)	0.41 (0.23-0.70)	.002
Total CHD						
No.	148	127	114	107	95	. . .
Age-adjusted RR (95% CI)	1.0 (Referent)	0.81 (0.64-1.02)	0.69 (0.54-0.89)	0.61 (0.47-0.79)	0.53 (0.40-0.69)	<.001
Age- and smoking-adjusted RR (95% CI)	1.0 (Referent)	0.98 (0.77-1.25)	0.92 (0.72-1.18)	0.89 (0.69-1.15)	0.80 (0.61-1.05)	.06
Multivariate RR (95% CI)†	1.0 (Referent)	0.98 (0.77-1.24)	0.92 (0.71-1.18)	0.87 (0.66-1.15)	0.77 (0.57-1.04)	.07

*CHD indicates coronary heart disease; MI, myocardial infarction; RR, relative risk; CI, confidence interval; and ellipses, data not applicable.
†Multivariate model controlled for age (5-year age groups), study period, body mass index (quintiles), smoking (never, past, 1 to 14 cigarettes per day, 15 to 24 cigarettes per day, or 25 or more cigarettes per day), menopausal status (premenopausal, postmenopausal never hormone use, current use, or past use), aspirin use (less than once, 1 to 6 times, or 7 or more times per week), multivitamin supplement use (no/yes), vitamin E supplement use (no/yes), regular exercise (no vs once or more per week), hypertension (no/yes), parental history of MI before age 60 years (no/yes), alcohol intake (0, less than 5, 5 to 14, or 15 or more g/d), energy intake, saturated fat intake, and carbohydrate intake (quintiles).

Table 3. Relative Risks (RRs) and 95% Confidence Intervals (CIs) of Coronary Heart Disease by Quintiles of Long-term Intake of Cereal, Vegetable, and Fruit Fiber Among Women

	Quintiles of Energy-Adjusted Long-term Fiber Intake, 1984-1990						
	1	2	3	4	5	Continuous Variable	*P* Value for Trend
**Cereal Fiber**							
Intake, median, g/d	2.2	3.1	3.8	4.9	7.7	Per 5 g†	
Multivariate analysis, RR (95% CI)*	1.00 (Referent)	1.06 (0.84-1.33)	0.71 (0.55-0.92)	0.76 (0.59-0.99)	0.68 (0.51-0.89)	0.71 (0.58-0.88)	.002
Multivariate analysis with folate, vitamin B_6, vitamin E, and magnesium, RR (95% CI)	1.00 (Referent)	1.06 (0.85-1.34)	0.71 (0.55-0.93)	0.76 (0.58-0.99)	0.66 (0.49-0.88)	0.63 (0.49-0.81)‡	<.001
**Vegetable Fiber**							
Intake, median, g/d	3.6	4.9	5.9	7.1	9.5	Per 5 g†	
Multivariate analysis, RR (95% CI)*	1.00 (Referent)	0.95 (0.74-1.22)	0.97 (0.75-1.24)	0.92 (0.71-1.19)	0.86 (0.66-1.11)	0.93 (0.80-1.10)	.38
Multivariate analysis with folate, vitamin B_6, vitamin C, and Beta carotene, RR (95% CI)	1.00 (Referent)	1.05 (0.80-1.37)	1.11 (0.82-1.49)	1.10 (0.79-1.52)	1.13 (0.77-1.64)	1.06 (0.84-1.32)‡	.63
**Fruit Fiber**							
Intake median, g/d	1.3	2.4	3.4	4.6	6.8	Per 5 g†	
Multivariate analysis RR (95% CI)*	1.00 (Referent)	0.90 (0.70-1.16)	0.80 (0.61-1.04)	0.98 (0.75-1.27)	0.91 (0.69-1.21)	0.98 (0.82-1.17)	.79
Multivariate analysis with folate, vitamin B_6, vitamin C, and Beta carotene, RR (95% CI)	1.00 (Referent)	0.92 (0.71-1.20)	0.82 (0.61-1.10)	1.00 (0.75-1.35)	0.94 (0.67-1.30)	0.93 (0.74-1.16)‡	.51

*Multivariate model controlled for age (5-year age groups), study period, body mass index (quintiles), smoking (never, past, 1-14 cigarettes per day, 15-24 cigarettes per day, or 25 or more cigarettes per day), menopausal status (premenopausal, postmenopausal never hormone use, current use, or past use), aspirin use (less than once, 1 to 6 times, or 7 or more times per week), multivitamin supplement use (no/yes), vitamin E supplement use (no/yes), regular exercise (no vs once or more per week), hypertension (no/yes), parental history of myocardial infarction before age 60 years (no/yes), alcohol intake (0, less than 5, 5 to 14, or 15 or more g/d), carbohydrate intake, and energy intake (quintiles).
†A 5-g increment approximately corresponds to the difference between medians in the highest and lowest quintiles of long-term intake.
‡These multivariate models for continuous variables were simultaneously adjusted for the other sources of fiber and for carbohydrate, dietary folate, vitamins B_6, C, and E, beta carotene, and magnesium intake.

©1999 American Medical Association. All rights reserved.

Downloaded From: https://jamanetwork.com/ by a Northeastern University Libraries User on 05/23/2019

Table 4. Relative Risk of Total CHD Associated With Long-term Intake of Cereal Fiber by Age, Smoking Status, Body Mass Index, and Long-term Intake of Saturated Fat and *Trans* Fat*

	Total CHD, No.	RR (95% CI) per 5 g of Cereal Fiber	*P* Value for Trend
Age, y			
<60	289	0.63 (0.44-0.90)	.01
≥60	302	0.76 (0.57-0.99)	.05
Smoking status			
Never/past	319	0.59 (0.43-0.79)	<.001
Current	272	0.87 (0.63-1.20)	.39
Body mass index, kg/m^2			
<25	249	0.58 (0.40-0.82)	.003
≥25	278	0.85 (0.62-1.17)	.31
Saturated fat			
Low tertile	177	0.62 (0.44-0.88)	.007
Medium tertile	194	0.79 (0.54-1.15)	.21
High tertile	220	0.68 (0.43-1.07)	.10
Trans fat			
Low tertile	189	0.69 (0.49-0.97)	.03
Medium tertile	211	0.77 (0.53-1.12)	.18
High tertile	191	0.57 (0.35-0.92)	.02

*CHD indicates coronary heart disease; RR, relative risk; and CI, confidence interval. All models were adjusted as in Table 2.

rated fat intake, and those in the highest tertile of *trans* fat intake.

We also examined foods high in cereal fiber, adjusting for standard CHD risk factors. Consumption of cold breakfast cereal 5 or more times per week in comparison with nonuse was associated with a 19% lower risk of total CHD (multivariate RR, 0.81; 95% CI, 0.62-1.06); the corresponding estimate for oatmeal was 29% (RR, 0.71; 95% CI, 0.38-1.34).

COMMENT

In this large, prospective cohort study of middle-aged women, we found a significant inverse association between intake of dietary fiber and risk of CHD. This association was confined to fiber from cereal sources. Women in the highest quintile of cereal fiber intake had a 34% lower risk of total CHD compared with those in the lowest quintile. This inverse association was not explained by higher dietary intakes of vitamin E, folate, vitamin B_6, magnesium, vegetables, or fruits.

The prospective design of our study avoids the potential for bias attributable to differential recall of intake by CHD cases and controls. Moreover, to reduce potential bias attributable to changes in fiber intake due to the diagnosis of diabetes or hypercholesterolemia, women with these conditions at baseline were excluded. Furthermore, we used up to 3 measurements of fiber intake per person during 10 years of follow-up in our analyses. These repeated measurements take into account possible changes in diet with time and reduce random variation in reporting. Women with a higher intake of fiber were more likely to have a higher intake of vitamin E, vitamin B_6, vitamin C, folate, beta carotene, and magnesium, and a lower intake of saturated and *trans* fats. However, adjustments for those nutrients 1 at a time as well as simultaneously did not change estimates appreciably.

Several previous prospective studies of men have reported inverse associations between fiber intake and CHD. Morris et al[11] followed up 337 men in London, England, for 10 to 20 years and reported a reduction in CHD mortality among those with greater intake of dietary fiber, mainly due to higher intake of cereal fiber. In the Zutphen Study of 871 men,[12] 37 men died of CHD during 10 years of follow-up; a risk reduction in the highest quintile of fiber intake was no longer significant after accounting for intake of energy, vegetable protein, and polysaccharides. In a prospective study of 1001 men of Irish descent in Boston, Mass, an inverse association between fiber and CHD risk was of borderline significance after multivariate adjustment.[13]

In a study by Khaw and Barrett-Connor,[19] 859 men and women completed a 24-hour recall of diet at baseline and were followed up for 12 years; 23 women and 42 men died of CHD. A 33% risk reduction was observed among women and 24% among men for a 6-g/d increase in dietary fiber intake. In a cohort of 31 208 California Seventh Day Adventists followed up for 6 years, consumption of whole-wheat bread was associated with lower risk of nonfatal MI, although fiber intake per se was not analyzed and women were not studied separately.[17] In the Health Professionals Follow-up Study of men, 734 cases of CHD were documented during 6 years of follow-up, and a significant 19% decreased risk of total CHD per 10-g/d increase of total dietary fiber and a 29% decrease per 10-g/d increase of cereal fiber were observed.[15]

Our results for women are the same for total fiber (19% per 10-g/d increase), but seem to be stronger for cereal fiber (37% lower risk per 5-g/d increase). In a large cohort of 21 930 Finnish men who smoked enrolled in the Alpha-Tocopherol, Beta-Carotene Cancer Prevention Study, 1399 CHD events (635 deaths) occurred during 6 years of follow-up, and a significant inverse association with dietary fiber was observed.[16] The association was stronger for coronary death (27% lower risk in the highest quintile of total dietary fiber intake compared with the lowest quintile) and was confined to cereal fiber, as in our study. Our results for cereal fiber and CHD are similar to findings for type 2 diabetes mellitus in the Nurses' Health Study, in which a 28% decrease in risk was observed when comparing the extreme quintiles.[48] Recently published findings from the Iowa Women's Health Study showed a 30% to 36% reduction in risk of ischemic heart disease death when comparing the extreme quintiles of whole-grain food intake.[20]

 ©1999 American Medical Association. All rights reserved.

Downloaded From: https://jamanetwork.com/ by a Northeastern University Libraries User on 05/23/2019

Wynder et al[49] have suggested that an observed protective effect of dietary fiber might be due to a generally healthy lifestyle among high-fiber consumers. Although fiber consumption was associated with healthier behavior, after detailed adjustment for a wide variety of lifestyle factors, an independent association remained. Moreover, the association was specific for cereal fiber, which would be difficult to explain by confounding due to overall lifestyle factors, especially because cereal fiber consumption was less strongly associated with healthier behaviors than was overall fiber intake. Also, the inverse association with cereal fiber was strongest among women who did not smoke, excluding the possibility that residual confounding by unmeasured aspects of smoking—the most important confounding variable—could account for our findings.

Fiber reduces both total and low-density lipoprotein cholesterol[21-23] through increased bile acid excretion and decreased hepatic synthesis of cholesterol.[50,51] However, based on a recent meta-analysis of 20 trials of high dosages of oat bran (rich in soluble fiber), the magnitude of the cholesterol-lowering effect was relatively small; reductions in serum cholesterol level ranged from 0.5% to 2% per gram of intake.[21] If a 1% decrease in serum cholesterol level reduces risk of CHD by 3%,[52] then a 5-g/d increase in cereal fiber (on average, a quarter of which is soluble) should decrease risk of CHD by only 1.9% to 7.5%. Therefore, the 37% reduction in CHD we found for every 5-g/d increase in cereal fiber is larger than would be expected from the beneficial effects on serum cholesterol level and suggests that other biological mechanisms are involved.

Other plausible mechanisms could include delayed absorption of macronutrients[53] leading to increased insulin sensitivity[30] and lower triglyceride levels.[31-33] In the Zutphen prospective study, fiber intake was inversely associated with hyperinsulinemia and insulin resistance, giving further support to this hypothesis.[26] These factors may be especially important in women because diabetes[54-56] and hypertriglyceridemia[57] appear to be stronger risk factors for CHD in women than in men. Whole-grain cereal products are also a main source of phytoestrogens in an average Western diet, in which soy products are not frequently consumed.[58] These mechanisms may be more important in women than in men and could be responsible for the stronger apparent effect of cereal fiber among women in our study than previously reported for men.[15,16] Other mechanisms, such as favorable effects of fiber on plasminogen activator type 1[24] and factor VII coagulation activity,[25,27] have also been suggested.

Although we attempted to control for micronutrients that are abundant in whole-grain foods, such as magnesium and folate, it is possible that other constituents of these foods, in addition to fiber per se, contribute to the lower risk of CHD. For this reason, our findings should be interpreted as supporting the consumption of minimally refined grains rather than purified fiber extracts.

In conclusion, our results provide evidence that an increase in foods high in dietary fiber, especially cereal products, may be protective against CHD in women. This provides further reason to replace refined forms of starch with whole-grain products.

Funding/Support: The work reported in this article was supported by grants CA40356, HL24074, and HL0353 from the National Institutes of Health, Bethesda, Md.
Acknowledgment: We are indebted to the participants in the Nurses' Health Study for their continuing outstanding level of cooperation, and to Al Wing, MBA, Mark Shneyder, Stefanie Parker, Gary Chase, Karen Corsano, MSL, Lisa Dunn, Barbara Egan, Lori Ward, and Jill Arnold for their unfailing help. Dr Wolk was a visiting scientist at Harvard School of Public Health, Boston, Mass, during the analyses presented in this article.

REFERENCES

1. Saldo BJ, Manton KG. Health status and service needs of the oldest old: current patterns and future trends. *Milbank Memorial Fund Q Health Soc.* 1985; 63:286-319.
2. Eaker ED, Chesebro JH, Sacks FM, Wenger NK, Whisnant JP, Winston M. Cardiovascular disease in women. *Circulation.* 1993;88:1999-2009.
3. National Center for Health Statistics. *Vital Statistics of the United States 1989*: *Volume II: Mortality*. Washington, DC: US Government Printing Office; 1993. DHHS publication PHS93-1101.
4. Rich-Edwards JW, Manson JE, Hennekens CH, Buring JE. The primary prevention of coronary heart disease in women. *N Engl J Med.* 1995;332:1758-1766.
5. Expert Panel on Detection, Evaluation and Treatment of High Blood Cholesterol in Adults. Summary of the second report of the National Cholesterol Education Program (NCEP): Expert Panel on Detection, Evaluation and Treatment of High Blood Cholesterol in Adults (Adult Treatment Panel). *JAMA.* 1993;269: 3015-3023.
6. Canadian Consensus Conference on the Prevention of Heart and Vascular Disease by Altering Serum Cholesterol and Lipoprotein Risk Factors (1989). *CMAJ.* 1989;49:629-635.
7. US Department of Health and Human Services, Public Health Service. *The Surgeon General's Report on Nutrition and Health*. Washington, DC: US Government Printing Office; 1988.
8. British Cardiac Society Working Group on Coronary Prevention (1987): conclusions and recommendations. *Br Heart J.* 1987;57:188-189.
9. Riccardi G, Rivellese AA, Mancini M. The use of diet to lower plasma cholesterol levels. *Eur Heart J.* 1987;8(suppl E):79-85.
10. Report of the National Cholesterol Education Program Expert Panel on Detection, Evaluation and Treatment of High Blood Cholesterol in Adults. *Ann Intern Med.* 1988;148:36-39.
11. Morris JN, Marr JW, Clayton DG. Diet and heart: a postscript. *BMJ.* 1977;2:1307-1314.
12. Kromhout D, Bosschieter EB, de Lezenne Coulander C. Dietary fiber and 10-year mortality from coronary heart disease, cancer and all causes: the Zutphen Study. *Lancet.* 1982;2:518-521.
13. Kushi LH, Lew RA, Stare FJ, et al. Diet and 20-year mortality from coronary heart disease: the Ireland-Boston Diet-Heart Study. *N Engl J Med.* 1985;312: 811-818.
14. Fehily AM, Yarnell JWG, Sweetnam PM, Elwood PC. Diet and incident ischemic heart disease: the Caerphilly Study. *Br J Nutr.* 1993;69:303-314.
15. Rimm EB, Ascherio A, Giovannucci E, Spiegelman D, Stampfer M, Willett WC. Vegetable, fruit and cereal fiber intake and risk of coronary heart disease among men. *JAMA.* 1996;275:447-451.
16. Pietinen P, Rimm EB, Korhonen P, et al. Intake of dietary fiber and risk of coronary heart disease in a cohort of Finnish men: the Alpha-Tocopherol, Beta-Carotene Cancer Prevention Study. *Circulation.* 1996; 94:2720-2727.
17. Fraser GE, Sabaté J, Beeson WL, Strahan TM. A possible protective effect of nut consumption on risk of coronary heart disease: the Adventist Health Study. *Arch Intern Med.* 1992;152:1416-1424.
18. Burr ML, Sweetnam PM. Vegetarianism, dietary fiber and mortality. *Am J Clin Nutr.* 1982;36:873-877.
19. Khaw KT, Barrett-Connor E. Dietary fiber and reduced ischemic heart disease mortality rates in men and women: a 12-year prospective study. *Am J Epidemiol.* 1987;126:1093-1102.
20. Jacobs DR Jr, Meyer KA, Kushi LH, Folsom AR. Whole-grain intake may reduce the risk of ischemic heart disease death in postmenopausal women: the Iowa Women's Health Study. *Am J Clin Nutr.* 1998; 68:248-257.
21. Ripsin CM, Keenan JM, Jacobs DR Jr, et al. Oat products and lipid lowering: a meta-analysis. *JAMA.* 1992;267:3317-3325.
22. Hunninghake DB, Miller VT, LaRosa JC, et al. Hypocholesterolemic effects of a dietary fiber supplement. *Am J Clin Nutr.* 1994;59:1050-1054.
23. Anderson JW, Tietyen-Clark J. Dietary fiber: hyperlipidemia, hypertension, and coronary heart disease. *Am J Gastroenterol.* 1986;81:907-919.
24. Sundell IB, Ranby M. Oat husk fiber decreases plasminogen activator inhibitor type 1 activity. *Haemostasis.* 1993;23:45-50.

©1999 American Medical Association. All rights reserved.

Downloaded From: https://jamanetwork.com/ by a Northeastern University Libraries User on 05/23/2019

25. Marckmann P, Sandström B, Jespersen J. Favorable long-term effect of a low-fat/high-fiber diet on human blood coagulation and fibrinolysis. *Arterioscler Thromb*. 1993;13:505-511.
26. Feskens EJ, Loeber JG, Kromhout D. Diet and physical activity as determinants of hyperinsulinemia: the Zutphen Elderly Study. *Am J Epidemiol.* 1994;140:350-360.
27. Smith U. Carbohydrates, fat, and insulin action. *Am J Clin Nutr.* 1994;59(suppl):686S-689S.
28. Mennen LI, Witteman JC, den Breeijen JH, et al. The association of dietary fat and fiber with coagulation factor VII in the elderly: the Rotterdam Study. *Am J Clin Nutr.* 1997;65:732-736.
29. Slavin Y, Jacobs D, Marquart L. Whole-grain consumption and chronic disease: protective mechanisms. *Nutr Cancer.* 1997;27:14-21.
30. Hallfrisch J, Scholfield DJ, Behall KM. Diets containing soluble oat extracts improve glucose and insulin responses of moderately hypercholesterolemic men and women. *Am J Clin Nutr.* 1995;61:379-384.
31. Rivellese A, Riccardi G, Giacco A, et al. Effect of dietary fibre on glucose control and serum lipoproteins in diabetic patients. *Lancet*. 1980;2:447-450.
32. Anderson JW, Chen WL. Plant fiber: carbohydrate and lipid metabolism. *Am J Clin Nutr*. 1979;23: 346-363.
33. Anderson JW, Chen WL, Sieling B. Hypolipidemic effects of high-carbohydrate, high-fiber diets. *Metabolism*. 1980;29:551-558.
34. Colditz GA, Manson JE, Hankinson SE. The Nurses' Health Study: 20-years' contribution to the understanding of health among women. *J Womens Health.* 1997;6:49-62.
35. US Department of Agriculture. *Composition of Foods—Raw, Processed, and Prepared, 1963-1988.* Washington, DC: US Dept of Agriculture; 1989. Agricultural Handbook Series, No 8.
36. Prosky L, Asp N, Furda I, DeVries JW, Schweizer TF, Harland BF. Determination of total dietary fiber in foods, food products and total diets: interlaboratory study. *J Assoc Off Anal Chem*. 1984;67:1044-1052.
37. Prosky L, Asp N, Furda I, DeVries JW, Schweizer TF, Harland BF. Determination of total dietary fiber and food products: collaborative study. *J Assoc Off Anal Chem.* 1985;68:677-679.
38. Prosky L, Asp N, Schweizer T, DeVries J, Furda I. Determination of insoluble, soluble and total dietary fiber in foods and food products: interlaboratory study. *J Assoc Off Anal Chem*. 1988;71:1017-1021.
39. Willett WC, Stampfer MJ. Total energy intake: implications for epidemiologic analyses. *Am J Epidemiol.* 1986;124:17-27.
40. Willett WC, Sampson L, Stampfer MJ, et al. Reproducibility and validity of a semiquantitative food frequency questionnaire. *Am J Epidemiol.* 1985;122: 51-65.
41. Willett WC, Reynolds RD, Cottrell-Hoehner S, Sampson L, Browne ML. Validation of a semiquantitative food-frequency questionnaire: comparison with a one-year diet record. *J Am Diet Assoc.* 1987;87:43-47.
42. Willett WC, Sampson L, Browne ML, et al. The use of a self-administered questionnaire to assess diet four years in the past. *Am J Epidemiol.* 1988;127: 188-199.
43. Rose GA, Blackburn H. *Cardiovascular Survey Methods*. Geneva, Switzerland: World Health Organization; 1982. WHO Monograph Series, No. 58.
44. Stampfer MJ, Willett WC, Speizer FE, et al. Test of the National Death Index. *Am J Epidemiol*. 1984; 119:837-839.
45. Hu FB, Stampfer MJ, Manson JE, et al. Dietary fat intake and the risk of coronary heart disease in women. *N Engl J Med.* 1997;337:1491-1499.
46. Shekelle RB, Stamler J, Paul O, Shryock AM, Liu S, Lepper M. Dietary lipids and serum cholesterol level: change in diet confounds the cross-sectional association. *Am J Epidemiol.* 1982;115:506-514.
47. Cupples LA, D'Agostino RB, Anderson K, Kannel WB. Comparison of baseline and repeated measure covariate techniques in the Framingham Heart Study. *Stat Med.* 1988;7:205-222.
48. Salmeron J, Manson JE, Stampfer MJ, Colditz GA, Wing AL, Willett WC. Dietary fiber, glycemic load, and risk of non-insulin-dependent diabetes mellitus in women. *JAMA*. 1997;277:472-477.
49. Wynder E, Stellman SD, Zang EA. High fiber intake: indicator of a healthy lifestyle. *JAMA*. 1996;275: 486-487.
50. Vahouny GV, Tombes R, Cassidy MM, Kritchevsky D, Gallo LL. Dietary fiber, V: binding salts, phospholipids and cholesterol from mixed micelles by bile acid sequestrants and dietary fibers. *Lipids*. 1980;15: 1012-1018.
51. Jenkins DJ, Wolever TM, Rao AV. Effect on blood lipids of very high intakes of fiber in diets low in saturated fat and cholesterol. *N Engl J Med.* 1993;329: 21-26.
52. Law MR, Wald NJ, Thompson SG. By how much and how quickly does reduction in serum cholesterol concentration lower risk of ischaemic heart disease? *BMJ*. 1994;308:367-372.
53. Cara L, Dubois C, Borel P, et al. Effects of oat bran, rice bran, wheat fiber, and wheat germ on postprandial lipemia in healthy adults. *Am J Clin Nutr.* 1992; 55:81-88.
54. Manson JE, Colditz GA, Stampfer MJ, et al. A prospective study of maturity-onset diabetes mellitus and risk of coronary heart disease and stroke in women. *Arch Intern Med*. 1991;151:1141-1147.
55. Barrett-Connor E, Wingard DL. Sex differential in ischemic heart disease mortality in diabetics: a propective population-based study. *Am J Epidemiol.* 1983; 118:489-496.
56. Pan WH, Cedres LB, Liu K, et al. Relationship of clinical diabetes and asymptomatic hyperglycemia to risk of coronary heart disease mortality in men and women. *Am J Epidemiol*. 1986;123:504-516.
57. Castelli WP. The triglyceride issue: a view from Framingham. *Am Heart J.* 1986;112:432-437.
58. Adlercreutz H, Hockerstedt K, Bannwart C, et al. Effect of dietary components, including lignans and phytoestrogens, on enterohepatic circulation and liver metabolism of estrogens and on sex hormone binding globulin (SHBG). *J Steroid Biochem*. 1987;27: 1135-1144.

Science moves, but slowly slowly,
creeping on from point to point.
—Alfred, Lord Tennyson (1809-1892)

 ©1999 American Medical Association. All rights reserved.

Downloaded From: https://jamanetwork.com/ by a Northeastern University Libraries User on 05/23/2019
