# Supplementary material for: A systematic comprehensive longitudinal evaluation of dietary factors associated with acute myocardial infarction and fatal coronary heart disease
Source: Nat Commun. 2020 Nov 27;11:6074. doi: 10.1038/s41467-020-19888-2 (PMC7699643; doi:10.1038/s41467-020-19888-2)
Supplement: Supplementary file 4 — Supplementary Software [file 41467_2020_19888_MOESM4_ESM.zip › EWAS-NHS-master/KG-SI/Rest/Zheng (2016).pdf]

# Dietary phosphatidylcholine and risk of all-cause and cardiovascular-specific mortality among US women and men[1,2]

*Yan Zheng,[3] Yanping Li,[3] Eric B Rimm,[3–5] Frank B Hu,[3–5] Christine M Albert,[6] Kathryn M Rexrode,[6] JoAnn E Manson,[3–6] and Lu Qi,[3,7]**

Departments of [3]Nutrition and [4]Epidemiology, Harvard T.H. Chan School of Public Health, Boston, MA; [5]Channing Division of Network Medicine and [6]Division of Preventive Medicine, Department of Medicine, Brigham and Women's Hospital and Harvard Medical School, Boston, MA; and [7]Department of Epidemiology, School of Public Health and Tropical Medicine, Tulane University, New Orleans, LA

## ABSTRACT

**Background:** The trimethylamine-containing nutrient phosphatidylcholine is the major dietary source for the gut microbiota metabolite trimethylamine-N-oxide (TMAO), which has been related to cardiovascular diseases (CVDs) and mortality. Previous research suggested that the relation of TMAO with CVD risk might be stronger in diabetic than in nondiabetic populations. However, the evidence for an association of dietary phosphatidylcholine with CVD and mortality is limited.

**Objectives:** We aimed to examine whether dietary consumption of phosphatidylcholine, which is mainly derived from eggs, red meat, and fish, is related to all-cause and CVD mortality in 2 cohorts of US women and men. In particular, we also tested if such an association was modified by diabetes status.

**Design:** We followed 80,978 women from the Nurses' Health Study (1980–2012) and 39,434 men from the Health Professionals Follow-Up Study (1986–2012), who were free of cancer and CVD at baseline, for mortality. Dietary intakes and potential confounders were assessed with regularly administered questionnaires. We used Cox proportional hazards models to estimate HRs and 95% CIs.

**Results:** We documented 17,829 all-cause and 4359 CVD deaths during follow-up. After multivariate adjustment for potential confounders, including demographic factors, disease status, lifestyle, and dietary intakes, higher phosphatidylcholine intakes were associated with an increased risk of all-cause and CVD mortality. HRs (95% CIs) comparing the top and bottom quintiles of phosphatidylcholine intake were 1.11 (1.06, 1.17; $P$-trend across quintiles $< 0.0001$) for all-cause mortality and 1.26 (1.15, 1.39; $P$-trend $< 0.0001$) for CVD mortality in the combined data of both cohorts. The associations of phosphatidylcholine with all-cause and CVD mortality were stronger in diabetic than in nondiabetic participants ($P$-interaction = 0.0002 and 0.001, respectively).

**Conclusion:** These data suggest that higher phosphatidylcholine consumption is associated with increased all-cause and CVD mortality in the US population, especially in patients with diabetes, independent of traditional risk factors. *Am J Clin Nutr* 2016;104:173–80.

**Keywords:** choline, mortality, diabetes, cardiovascular disease, TMAO

## INTRODUCTION

Several previous studies found that elevated blood concentrations of trimethylamine-N-oxide (TMAO)[8] significantly predicted the risk of cardiovascular disease (CVD) and mortality in prospective cohorts (1–3). TMAO is produced mainly through metabolism by the gut microbiota of trimethylamine-containing nutrients, especially phosphatidylcholine (1, 2). In mice, higher dietary phosphatidylcholine led to increases in plasma concentrations of TMAO and eventually promoted atherosclerosis (1). In humans, a dietary phosphatidylcholine challenge induced increased postprandial concentrations of TMAO in both blood and urine samples (3). However, the evidence for an association of dietary phosphatidylcholine with CVD and mortality in humans is limited.

In our recent analyses, dietary phosphatidylcholine intake was consistently associated with an increased risk of type 2 diabetes in 3 US populations (4). Furthermore, patients with type 2 diabetes had a 2- to 3-fold higher risk of CVD, cardiovascular-specific mortality, or premature mortality than did the general population (5, 6). Thus far, the exact pathogenic mechanisms underlying this increased risk are not fully understood. A previous study showed that the risk of incident major adverse cardiovascular events, including mortality attributable to increased blood TMAO concentrations, appeared to be greater in diabetic patients than in

[1] Supported by the NIH (UM1 CA186107, R01 HL034594, UM1 CA167552, and R01 HL35464); the National Heart, Lung, and Blood Institute (HL071981, HL034594, and HL126024); the National Institute of Diabetes and Digestive and Kidney Diseases (DK091718, DK100383, and DK078616); the Boston Obesity Nutrition Research Center (DK46200); and United States–Israel Binational Science Foundation grant 2011036.

[2] Supplemental Table 1 is available from the "Online Supporting Material" link in the online posting of the article and from the same link in the online table of contents at http://ajcn.nutrition.org.

*To whom correspondence should be addressed. E-mail: lqi1@tulane.edu.

[8] Abbreviations used: CVD, cardiovascular disease; FFQ, food-frequency questionnaire; HPFS, Health Professionals Follow-Up Study; HRT, hormone replacement therapy; NHS, Nurses' Health Study; TMAO, trimethylamine-N-oxide.

Received January 28, 2016. Accepted for publication May 11, 2016.
First published online June 8, 2016; doi: 10.3945/ajcn.116.131771.

those without diabetes (HRs comparing the top and bottom quintiles of TMAO concentrations were 2.5 and 1.6 in diabetic and nondiabetic subjects, respectively) (3).

In the current study, we prospectively examined the association between dietary phosphatidylcholine intake and mortality, especially CVD-specific mortality, among US women and men from 2 large cohorts: the Nurses' Health Study (NHS) (7) and the Health Professionals Follow-Up Study (HPFS) (8). In particular, we further assessed whether this association was stronger in diabetic than in nondiabetic populations.

## METHODS

### Study population

The NHS cohort began in 1976 with the recruitment of 121,700 female registered nurses, aged 30–55 y, residing in 11 large US states. The medical history, lifestyle information, and disease diagnosis were updated every 2 y with the use of a validated questionnaire (7). The HPFS cohort is a prospective cohort of 51,529 US male health professionals, aged 40–75 y, who returned a questionnaire on diet and medical history in 1986 (8). Detailed descriptions of these cohorts have been presented elsewhere (9, 10). In both cohorts, questionnaires were administered at baseline, as well as biennially after baseline, to collect and update information on lifestyle practices and the occurrence of chronic diseases. The follow-up rates of the participants in these cohorts are all >90%. In the current dietary analysis, we excluded men and women who had diagnoses of CVD (including stroke, myocardial infarction, angina, and/or coronary revascularization) or cancer at baseline (1980 for NHS and 1986 for HPFS, when dietary information was first collected) or who reported unusual total energy intakes (i.e., <800 or >4200 kcal/d for men and <500 or >3500 kcal/d for women).

We defined diabetic participants by using their self-reported diabetes status and included their person-time beginning at either baseline for participants with prevalent diabetes or when a questionnaire that reported a diagnosis of diabetes was returned for participants with incident diabetes during the follow-up period. Participants who developed CVD or cancer before and/or at the same time of diagnosis of type 2 diabetes between the baseline questionnaire and the end of this study period (1 June 2012 for the NHS and 31 January 2012 for the HPFS) were also excluded. The final study sample included 80,978 women and 39,434 men. The present study was approved by the institutional review board at Brigham and Women's Hospital, and the return of the questionnaires implied informed consent.

### Confirmation of type 2 diabetes

Participants who reported a diagnosis of type 2 diabetes on the main questionnaire were mailed a supplementary questionnaire with regard to symptoms, diagnostic tests, and hypoglycemic therapy. National Diabetes Data Group criteria (11) were used to diagnose type 2 diabetes before the release of the American Diabetes Association criteria in 1997. The American Diabetes Association diagnostic criteria were adopted to diagnose cases of type 2 diabetes during the 1998 and 2012 cycles (12). Our validation study showed that 98% of the self-reported cases of type 2 diabetes were confirmed by review of medical records in women (13) and in men (14). The diagnostic criteria that we used have been reported in detail elsewhere (15).

### Outcome ascertainment

The endpoints in this study included all-cause and CVD mortality. The ascertainment of death was documented in a previous study (16). Briefly, deaths were reported by next of kin or the postal system or identified through the National Death Index. We estimated previously that follow-up for deaths was >98% complete (17). We obtained death certificate copies and medical records and determined causes of death according to the *International Classification of Diseases, Eighth Revision*, and we specifically considered deaths due to CVD (codes 390.0–458.9 or 795.0–795.9). CVD is defined as a composite of coronary artery disease and stroke (nonfatal or fatal). Coronary artery disease is defined as a composite of nonfatal or fatal myocardial infarction or fatal coronary artery disease. Briefly, the incidence of nonfatal myocardial infarction and stroke was ascertained from the biennial follow-up questionnaires and confirmed by reviewing medical records with the WHO criteria for myocardial infarction (18) or the National Survey of Stroke criteria for stroke (19). Fatal cases of coronary artery disease and stroke were identified if coronary artery disease or stroke was listed as the cause of death in multiple sources, including autopsy reports, hospital records, and death certificates.

### Assessment of diet and covariates

The women in the NHS completed a food-frequency questionnaire (FFQ) first in 1980 and again in 1984, 1986, 1990, 1994, 1998, 2002, and 2006. We assessed dietary information by using the FFQ from 1986 to 2006 in the HPFS, which was administered every 4 y in the cohort. Participants were asked how often, on average, they had consumed each type of food during the past year. Serving sizes were specified for each food in the FFQ. The questionnaire had 9 possible responses, ranging from never or <1 time/mo to ≥6 times/d. Nutrient intakes were calculated by multiplying the frequency of intake for each food by its nutrient content and summing these products across all food items.

Information on the major food items containing phosphatidylcholine was obtained from the USDA database (http://www.ars.usda.gov/ba/bhnrc/ndl) (20) and from values published by Zeisel et al. (21). Our previous data showed that dietary phosphatidylcholine is derived mainly from intakes of egg, red meat, and fish (4). We used the regression-residual method to adjust nutrient intakes for total energy intake (22). Intakes of choline and betaine measured by our FFQ predicted plasma total homocysteine concentrations in the NHS (23), which indicated the validity and biological relevance of intakes of choline and its derivatives measured by the FFQ.

Information on potential confounders, including age, race, marital status, BMI, family history of diseases, smoking status, alcohol consumption, physical activity, medication use, menopausal status and postmenopausal hormone therapy use for women, and a history of major disease conditions, including diabetes, hypertension, hypercholesterolemia, cancer, and coronary artery bypass graft, was collected via regular biennial questionnaires throughout follow-up of the NHS and the HPFS.

### Statistical analysis

Person-time was calculated for each participant from baseline to the occurrence of death or the end of the study period (1 June 2012 for the NHS and 31 January 2012 for the HPFS). Cox proportional hazards regression was used to examine the associations between dietary phosphatidylcholine and all-cause and CVD mortality. To represent the long-term intake of dietary factors and to reduce measurement error, we conducted analyses with the use of updated dietary data by taking the average of all available previous dietary questionnaires (24). The consumption of phosphatidylcholine was categorized into quintiles in each cohort, and the median values in each quintile were used to test for linear trends of associations across the quintiles. Nondietary covariates were updated at each biennial follow-up cycle. Multivariable models were first adjusted for the following factors (model 1): age (mo); BMI (kg/m$^2$); white race (yes or no); marital status (married or single); menopausal status and postmenopausal hormone replacement therapy [HRT; premenopausal, postmenopausal + HRT nonuser, postmenopausal + current HRT user, postmenopausal + past HRT user, or missing (women only)]; family history of CVD (yes or no); smoking status and smoking pack-years (never; past smoker: ≤5 pack-years, 6–20 pack-years, or ≥21 pack-years; current smoker: ≤5 pack-years, 6–20 pack-years, or ≥21 pack-years); alcohol consumption (0, 0.1–4.9, 5.0–14.9, or ≥15.0 g/d for women and 0, 0.1–4.9, 5.0–29.9, or ≥30.0 g/d for men); physical activity (in quintiles, and a category of missing values); the presence of diabetes, hypertension, or hypercholesterolemia (yes or no for each); and regular aspirin use (yes or no). In model 2 we further adjusted for cumulative averages of dietary factors including total energy, ratio of dietary polyunsaturated to saturated fat, and intakes of *trans* fat (all in quintiles). Modeling of multiplicative interaction terms for age and quintiles of dietary phosphatidylcholine intake did not suggest that the proportional hazards assumption was violated ($P$-interaction $> 0.05$). We further conducted the above-mentioned analysis in diabetic and nondiabetic participants separately.

In sensitivity analyses, we also further mutually adjusted for the following: *1*) major food sources of phosphatidylcholine, including eggs, red meat, and fish, and *2*) major nutrients involved in the phosphatidylcholine metabolism including folate, vitamin B-12, and choline. To minimize residual confounding, we performed additional stratified analyses by age (<60 or ≥60 y), BMI (<30 or ≥30), current smoker (yes or no), current alcohol consumer (yes or no), physical activity (low or high; the cutoff was the median in each cohort), and the major food sources of phosphatidylcholine (i.e., eggs, red meat, and fish; low or high intakes; the cutoff was the median in each cohort). The interaction between phosphatidylcholine and the above factors on all-cause mortality was assessed by testing the significance of the multiplicative interaction term in model 2. Because no significant heterogeneity between cohorts was found, we combined the 2 cohorts and present the results with further adjustment for sex. In addition, we analyzed the associations of phosphatidylcholine intake with incident CVD and incident coronary artery disease in our study population.

The SAS statistical software was used for all analyses (SAS version 9.3 for UNIX; SAS Institute). Significance was set at a 2-tailed $\alpha$ level of 0.05.

## RESULTS

### Characteristics of diabetic cohorts at baseline

Overall, there were 80,978 women from the NHS and 39,434 men from the HPFS included in the current analysis. The mean intake of dietary phosphatidylcholine was, in general, comparable between men and women. Compared with participants who had lower intakes of dietary phosphatidylcholine, those who had higher intakes were more likely to have a higher BMI, consume less alcohol, be more likely to have type 2 diabetes, and consume more eggs and fish in both cohorts (**Table 1**).

### Dietary phosphatidylcholine intake and mortality in the general population

During up to 32 y of follow-up in the NHS (2,078,089 person-years), we confirmed 11,114 deaths; during up to 26 y of follow-up in the HPFS (744,688 person-years), we confirmed 6715 deaths. The crude all-cause mortality rate appeared to be higher in men than in women in each of the quintiles of dietary phosphatidylcholine intake. In age-adjusted analyses, we observed that dietary phosphatidylcholine intake was associated with a higher risk of all-cause mortality in both the NHS and the HPFS (both $P$-trend $< 0.0001$; **Table 2**). The associations were attenuated but remained significant after multivariate adjustment in model 1. Additional adjustment for other dietary factors (i.e., dietary intakes of energy and *trans* fat and ratio of polyunsaturated to saturated fat) further attenuated the observed HR, but the association in both cohorts remained significant (multivariate model 2). In the combined results, the associations between phosphatidylcholine intakes and all-cause mortality were significant in all of the age- and multivariate-adjusted models tested. In multivariate model 2 of the combined cohorts, HRs (95% CIs) across quintiles of phosphatidylcholine intakes were 1.00 (reference), 1.02 (0.97, 1.07), 1.01 (0.96, 1.07), 1.07 (1.02, 1.13), and 1.11 (1.06, 1.17) ($P$-trend $< 0.0001$); each 100-mg/d higher phosphatidylcholine intake was associated with an 8% (95% CI: 5%, 11%) increment in all-cause mortality.

We then performed sensitivity analyses to further adjust for the 3 major food sources of phosphatidylcholine (red meat, eggs, and fish) in the model and found that participants in the top quintile of phosphatidylcholine intake still had a 7% (95% CI: 2%, 13%) higher risk of all-cause mortality than those in the bottom quintile in the combined analysis, and the linear trend across quintiles was retained ($P$-trend = 0.002). When we further adjusted for the main nutrients of folate, vitamin B-12, and choline, the associations remained similar ($P$-trend = 0.03). We also excluded participants who took lecithin (phosphatidylcholine) supplements, and the results did not materially change (data are not shown). The risk of death attributable to a higher intake of phosphatidylcholine (above the cohort-specific median) was 1.66% in the combined data set.

We analyzed the associations of dietary phosphatidylcholine with all-cause mortality in strata by major risk factors and food sources of phosphatidylcholine (**Figure 1**). The various risk profiles defined by age, physical activity, smoking, alcohol intake status, and consumption of major phosphatidylcholine-containing foods did not significantly modify such associations ($P$-interaction $>$ 0.5). However, the association of dietary phosphatidylcholine with all-cause mortality was stronger among obese participants than in those who were not obese ($P$-interaction $< 0.0001$).

**TABLE 1**

Age-adjusted baseline characteristics of participants in the NHS (1980) and HPFS (1986) according to quintiles of dietary phosphatidylcholine intake[1]

| | Quintile of dietary phosphatidylcholine intake | | | | |
|---|---|---|---|---|---|
| | 1 (low) | 2 | 3 | 4 | 5 (high) |
| NHS (*n* = 80,978) | | | | | |
| Phosphatidylcholine intake, mg/d | 134.1 ± 20.3[2] | 174.6 ± 8.2 | 201.4 ± 7.5 | 230.1 ± 9.7 | 296.7 ± 59.7 |
| Age,[3] y | 46.2 ± 7.3 | 46.1 ± 7.2 | 46.2 ± 7.2 | 46.5 ± 7.1 | 46.9 ± 7.0 |
| White, % | 98.0 | 98.0 | 97.7 | 97.4 | 96.7 |
| Married, % | 90.8 | 92.0 | 92.1 | 91.7 | 90.4 |
| BMI, kg/m$^2$ | 23.7 ± 4.3 | 23.9 ± 4.3 | 24.2 ± 4.4 | 24.4 ± 4.5 | 24.9 ± 4.7 |
| Physical activity, MET-h/wk | 13.3 ± 21.3 | 13.7 ± 19.1 | 14.0 ± 19.0 | 14.5 ± 21.0 | 15.0 ± 21.2 |
| Current use of postmenopausal hormones, % | 7.9 | 7.8 | 8.0 | 8.3 | 9.1 |
| Current smoker, % | 32.4 | 28.5 | 27.3 | 26.9 | 26.3 |
| Type 2 diabetes, % | 0.3 | 0.4 | 0.5 | 0.6 | 1.1 |
| Hypertension, % | 13.1 | 14.3 | 14.3 | 14.9 | 16.7 |
| Hypercholesterolemia, % | 5.2 | 4.8 | 4.8 | 4.7 | 5.0 |
| Family history of myocardial infarction, % | 18.6 | 18.2 | 19.1 | 18.4 | 19.0 |
| Current aspirin user, % | 39.9 | 40.3 | 40.1 | 40.3 | 39.9 |
| Dietary factors | | | | | |
| Energy, kcal/d | 1535.8 ± 520.6 | 1592.6 ± 494.9 | 1592.9 ± 484.8 | 1575.1 ± 478.5 | 1529.9 ± 515.0 |
| Alcohol, g/d | 7.7 ± 13.1 | 6.9 ± 11.1 | 6.2 ± 9.7 | 5.9 ± 9.3 | 5.2 ± 8.6 |
| Ratio of polyunsaturated to saturated fat | 0.4 ± 0.2 | 0.4 ± 0.1 | 0.3 ± 0.1 | 0.3 ± 0.1 | 0.3 ± 0.1 |
| *trans* Fat, g/d | 3.8 ± 1.9 | 4.1 ± 1.9 | 4.1 ± 1.8 | 4.0 ± 1.8 | 3.6 ± 1.9 |
| Red meat,[4] serving/d | 1.0 ± 0.6 | 1.1 ± 0.7 | 1.2 ± 0.7 | 1.2 ± 0.7 | 1.1 ± 0.7 |
| Eggs,[4] serving/d | 0.2 ± 0.2 | 0.2 ± 0.3 | 0.3 ± 0.3 | 0.3 ± 0.3 | 0.4 ± 0.4 |
| Fish,[4] serving/d | 0.1 ± 0.2 | 0.1 ± 0.2 | 0.1 ± 0.2 | 0.1 ± 0.2 | 0.2 ± 0.2 |
| HPFS (*n* = 39,434) | | | | | |
| Phosphatidylcholine intake, mg/d | 134.7 ± 17.6 | 170.4 ± 7.7 | 196.2 ± 7.5 | 226.6 ± 10.7 | 324.8 ± 84.5 |
| Age,[3] y | 52.3 ± 9.7 | 52.5 ± 9.4 | 53.0 ± 9.5 | 53.8 ± 9.5 | 54.9 ± 9.4 |
| White, % | 91.2 | 92.0 | 91.0 | 91.1 | 89.9 |
| Married, % | 88.8 | 90.8 | 91.2 | 91.1 | 89.3 |
| BMI, kg/m$^2$ | 24.9 ± 2.9 | 25.2 ± 3.1 | 25.4 ± 3.1 | 25.8 ± 3.4 | 25.8 ± 3.5 |
| Physical activity, MET-h/wk | 23.2 ± 31.4 | 21.9 ± 28.1 | 21.0 ± 26.6 | 19.9 ± 29.3 | 20.9 ± 32.4 |
| Current smoker, % | 8.2 | 9.1 | 8.8 | 10.1 | 11.5 |
| Type 2 diabetes, % | 1.4 | 1.8 | 2.0 | 3.0 | 3.9 |
| Hypertension, % | 19.0 | 18.4 | 20.2 | 20.3 | 19.2 |
| Hypercholesterolemia, % | 12.0 | 10.9 | 10.0 | 9.1 | 9.4 |
| Family history of myocardial infarction, % | 32.4 | 32.4 | 31.5 | 32.0 | 30.7 |
| Current aspirin user, % | 26.2 | 27.1 | 26.2 | 25.8 | 26.6 |
| Dietary factors | | | | | |
| Energy, kcal/d | 1943.8 ± 616.3 | 2008.3 ± 622.9 | 2036.5 ± 616.1 | 2010.9 ± 610.1 | 1983.7 ± 641.3 |
| Alcohol, g/d | 13.5 ± 18.8 | 12.3 ± 15.9 | 11.3 ± 14.4 | 10.3 ± 13.4 | 9.3 ± 13.1 |
| Ratio of polyunsaturated to saturated fat | 0.6 ± 0.2 | 0.6 ± 0.2 | 0.6 ± 0.2 | 0.5 ± 0.2 | 0.5 ± 0.2 |
| *trans* Fat, g/d | 2.7 ± 1.7 | 2.9 ± 1.6 | 3.0 ± 1.6 | 2.9 ± 1.5 | 2.7 ± 1.5 |
| Red meat,[4] serving/d | 0.7 ± 0.6 | 1.0 ± 0.7 | 1.2 ± 0.8 | 1.4 ± 0.8 | 1.4 ± 1.0 |
| Eggs,[4] serving/d | 0.1 ± 0.1 | 0.2 ± 0.2 | 0.3 ± 0.2 | 0.4 ± 0.2 | 0.7 ± 0.7 |
| Fish,[4] serving/d | 0.3 ± 0.2 | 0.3 ± 0.3 | 0.3 ± 0.3 | 0.4 ± 0.3 | 0.4 ± 0.3 |

[1]Values were standardized to the age distribution of the study population. HPFS, Health Professionals Follow-Up Study; MET-h, metabolic equivalent task hours; NHS, Nurses' Health Study.

[2]Mean ± SD (all such values).

[3]Values were not age adjusted.

[4]Serving sizes: red meat = 4 oz.; eggs = 1 whole egg; fish = 4 oz.

In the NHS and the HPFS, there were 2297 and 2060 deaths confirmed to be due to CVD, respectively. The crude CVD-specific mortality rate was higher in men than in women. Dietary phosphatidylcholine intakes were associated with a higher age- and multivariate-adjusted risk of CVD-specific mortality in both cohorts ($P$-trend < 0.0001) (**Table 3**). Women and men in the top quintile of phosphatidylcholine intake tended to have 19% and 39% higher RRs (95% CIs: 5%, 35%, and 20%, 61%, respectively) of CVD-specific mortality than those in the bottom quintile. In the combined results from model 2, HRs (95% CIs) across quintiles of phosphatidylcholine intakes were 1.00 (reference), 1.09 (0.98, 1.21), 1.03 (0.93, 1.14), 1.07 (0.97, 1.18), and 1.26 (1.15, 1.39) ($P$-trend < 0.0001). Each 100-mg/d higher phosphatidylcholine intake was significantly related to a 13% (95% CI: 7%, 19%) increment in CVD-specific mortality after adjustment for covariates from women and men combined. Further adjustment for the major food sources of phosphatidylcholine or the main nutrients involved in phosphatidylcholine metabolism did not change the results meaningfully ($P$-trend < 0.0001 and = 0.002 in the combined data set).

**TABLE 2**
HRs (95% CIs) of all-cause mortality by quintile of energy-adjusted cumulative dietary choline from phosphatidylcholine in US women and men[1]

| | Quintile of intake of choline from phosphatidylcholine | | | | | |
|---|---|---|---|---|---|---|
| | 1 (low) | 2 | 3 | 4 | 5 (high) | *P*-trend |
| NHS | | | | | | |
| Median intake, mg/d | 129.6 | 153.6 | 171.4 | 191.4 | 235.5 | |
| No. of cases/person-years | 2224/416,722 | 2005/416,535 | 1998/416,267 | 2216/415,412 | 2671/413,153 | |
| Rate per 100,000 person-years | 534 | 481 | 480 | 533 | 646 | |
| Age-adjusted | 1 | 0.96 (0.90, 1.02) | 0.98 (0.93, 1.05) | 1.09 (1.03, 1.15) | 1.30 (1.23, 1.37) | <0.0001 |
| Multivariate model 1[2] | 1 | 1.02 (0.96, 1.09) | 1.03 (0.97, 1.09) | 1.09 (1.02, 1.15) | 1.08 (1.02, 1.14) | 0.003 |
| Multivariate model 2[3] | 1 | 1.01 (0.95, 1.08) | 1.01 (0.95, 1.08) | 1.07 (1.00, 1.13) | 1.06 (1.00, 1.12) | 0.03 |
| HPFS | | | | | | |
| Median intake, mg/d | 139.5 | 166.1 | 187.1 | 211.5 | 261.3 | |
| No. of cases/person-years | 1106/149,346 | 1141/149,301 | 1187/149,292 | 1439/148,779 | 1842/147,970 | |
| Rate per 100,000 person-years | 741 | 764 | 795 | 967 | 1245 | |
| Age-adjusted | 1 | 1.01 (0.93, 1.10) | 1.03 (0.94, 1.12) | 1.17 (1.08, 1.27) | 1.42 (1.31, 1.53) | <0.0001 |
| Multivariate model 1[2] | 1 | 1.04 (0.96, 1.14) | 1.05 (0.96, 1.14) | 1.15 (1.06, 1.25) | 1.25 (1.16, 1.36) | <0.0001 |
| Multivariate model 2[3] | 1 | 1.03 (0.95, 1.13) | 1.04 (0.95, 1.13) | 1.13 (1.04, 1.23) | 1.23 (1.13, 1.33) | <0.0001 |
| Pooled results | | | | | | |
| No. of cases/person-years | 3330/565,431 | 3146/565,067 | 3185/564,761 | 3655/563,261 | 4513/560,138 | |
| Rate per 100,000 person-years | 589 | 557 | 564 | 649 | 806 | |
| Age-adjusted | 1 | 0.99 (0.94, 1.04) | 1.01 (0.96, 1.06) | 1.13 (1.08, 1.18) | 1.37 (1.31, 1.43) | <0.0001 |
| Multivariate model 1[2] | 1 | 1.03 (0.98, 1.08) | 1.03 (0.98, 1.08) | 1.10 (1.04, 1.15) | 1.14 (1.09, 1.20) | <0.0001 |
| Multivariate model 2[3] | 1 | 1.02 (0.97, 1.07) | 1.01 (0.96, 1.07) | 1.07 (1.02, 1.13) | 1.11 (1.06, 1.17) | <0.0001 |

[1]HPFS, Health Professionals Follow-Up Study; HRT, hormone replacement therapy; NHS, Nurses' Health Study.

[2]Model 1 adjusted for the following: age (mo); BMI ($kg/m^2$); white race (yes or no); marital status (married or single); menopausal status and postmenopausal HRT [premenopausal, postmenopausal + HRT nonuser, postmenopausal + current HRT user, postmenopausal + past HRT user, or missing (women only)]; family history of cardiovascular disease (yes or no); smoking status and smoking pack-years (never; past smoker: ≤5 pack-years, 6–20 pack-years, or ≥21 pack-years; current smoker: ≤5 pack-years, 6–20 pack-years, or ≥21 pack-years); alcohol consumption (0, 0.1–4.9, 5.0–14.9, or ≥15.0 g/d for women and 0, 0.1–4.9, 5.0–29.9, or ≥30.0 g/d for men); physical activity (in quintiles, and a category of missing values); the presence of diabetes, hypertension, or hypercholesterolemia (yes or no for each); and regular aspirin use (yes or no).

[3]Model 2 further adjusted for dietary intakes of energy and *trans* fat and the ratio of polyunsaturated to saturated fat (quintiles).

## Dietary phosphatidylcholine intake and mortality among diabetic and nondiabetic populations

We documented 2533 all-cause deaths and 799 CVD-specific deaths from 12,769 diabetic participants, and 15,296 all-cause deaths and 3558 CVD-specific deaths from 110,630 nondiabetic participants. In the combined population of men and women, the diabetic participants in the top quintile of phosphatidylcholine intake had a 24% increased risk of all-cause mortality and a 67%

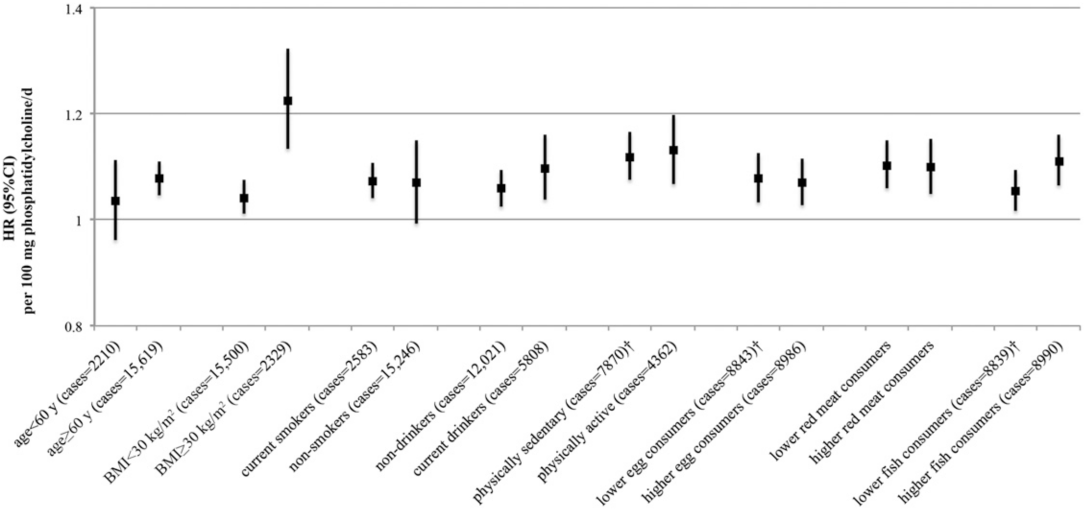


**FIGURE 1** Stratified HRs of all-cause mortality per 100 mg phosphatidylcholine/d. All *P*-interaction values were >0.05, except for BMI (*P*-interaction < 0.0001). Covariates included in the analysis were: age; BMI ($kg/m^2$); white race (yes or no); marital status; menopausal status and postmenopausal HRT (women only); family history of CVD; smoking status and smoking pack-years; alcohol consumption; physical activity; the presence of diabetes, hypertension, or hypercholesterolemia; regular aspirin use (yes or no); dietary intakes of energy and *trans* fat; and the ratio of polyunsaturated to saturated fat. We used Cox proportional hazards models to estimate HRs and 95% CIs with updated dietary measurements from the combined data set of both the NHS and the HPFS. †Cutoffs are the cohort-specific median value. CVD, cardiovascular disease; HPFS, Health Professionals Follow-Up Study; HRT, hormone replacement therapy; NHS, Nurses' Health Study.

**TABLE 3**
HRs (95% CIs) of CVD-specific mortality by quintile of energy-adjusted cumulative dietary choline from phosphatidylcholine in US women and men[1]

| | Quintile of choline intake from phosphatidylcholine | | | | | |
|---|---|---|---|---|---|---|
| | 1 (low) | 2 | 3 | 4 | 5 (high) | *P*-trend |
| NHS | | | | | | |
| Median intake, mg/d | 129.6 | 153.6 | 171.4 | 191.4 | 235.5 | |
| No. of cases/person-years | 434/416,722 | 425/416,535 | 389/416,267 | 436/415,412 | 613/413,153 | |
| Rate per 100,000 person-years | 104 | 102 | 93 | 105 | 148 | |
| Age-adjusted | 1 | 1.04 (0.91, 1.19) | 0.98 (0.85, 1.12) | 1.09 (0.95, 1.25) | 1.50 (1.33, 1.70) | <0.0001 |
| Multivariate model 1[2] | 1 | 1.08 (0.94, 1.23) | 0.99 (0.86, 1.13) | 1.04 (0.91, 1.19) | 1.22 (1.08, 1.39) | 0.002 |
| Multivariate model 2[3] | 1 | 1.07 (0.93, 1.22) | 0.97 (0.85, 1.12) | 1.02 (0.89, 1.17) | 1.19 (1.05, 1.35) | 0.008 |
| HPFS | | | | | | |
| Median intake, mg/d | 139.5 | 166.1 | 187.1 | 211.5 | 261.3 | |
| No. of cases/person-years | 308/150,072 | 342/149,997 | 374/150,062 | 439/149,665 | 597/149,115 | |
| Rate per 100,000 person-years | 205 | 228 | 249 | 293 | 400 | |
| Age-adjusted | 1 | 1.09 (0.93, 1.28) | 1.15 (0.98, 1.34) | 1.28 (1.10, 1.49) | 1.62 (1.41, 1.87) | <0.0001 |
| Multivariate model 1[2] | 1 | 1.12 (0.95, 1.31) | 1.15 (0.98, 1.34) | 1.22 (1.05, 1.42) | 1.41 (1.22, 1.63) | <0.0001 |
| Multivariate model 2[3] | 1 | 1.11 (0.95, 1.31) | 1.14 (0.97, 1.33) | 1.21 (1.04, 1.41) | 1.39 (1.20, 1.61) | <0.0001 |
| Pooled results | | | | | | |
| No. of cases/person-years | 742/565,431 | 767/565,067 | 763/564,761 | 875/563,261 | 1210/560,138 | |
| Rate per 100,000 person-years | 131 | 136 | 135 | 155 | 216 | |
| Age-adjusted | 1 | 1.09 (0.98, 1.20) | 1.08 (0.97, 1.20) | 1.20 (1.09, 1.32) | 1.61 (1.47, 1.76) | <0.0001 |
| Multivariate model 1[2] | 1 | 1.10 (0.99, 1.22) | 1.04 (0.94, 1.16) | 1.09 (0.98, 1.20) | 1.29 (1.18, 1.42) | <0.0001 |
| Multivariate model 2[3] | 1 | 1.09 (0.98, 1.21) | 1.03 (0.93, 1.14) | 1.07 (0.97, 1.18) | 1.26 (1.15, 1.39) | <0.0001 |

[1]CVD, cardiovascular disease; HPFS, Health Professionals Follow-Up Study; HRT, hormone replacement therapy; NHS, Nurses' Health Study.

[2]Model 1 adjusted for the following: age (mo); BMI (kg/m$^2$); white race (yes or no); marital status (married or single); menopausal status and postmenopausal HRT [premenopausal, postmenopausal + HRT nonuser, postmenopausal +current HRT user, postmenopausal + past HRT user, or missing (women only)]; family history of CVD (yes or no); smoking status and smoking pack-years (never; past smoker: ≤5 pack-years, 6–20 pack-years, or ≥21 pack-years; current smoker: ≤5 pack-years, 6–20 pack-years, or ≥21 pack-years); alcohol consumption (0, 0.1–4.9, 5.0–14.9, or ≥15.0 g/d for women and 0, 0.1–4.9, 5.0–29.9, or ≥30.0 g/d for men); physical activity (in quintiles, and a category of missing values); the presence of diabetes, hypertension, or hypercholesterolemia (yes or no for each); and regular aspirin use (yes or no).

[3]Model 2 further adjusted for dietary intakes of energy and *trans* fat and the ratio of polyunsaturated to saturated fat (quintiles).

increased CVD-mortality risk, whereas the nondiabetic participants in the top quintile had a 9% increased all-cause mortality risk and a 19% increased CVD-specific mortality risk than those in the bottom quintile in each population (**Table 4**). Diabetes status modified the association of dietary phosphatidylcholine with risks of all-cause and CVD-specific mortality (*P*-interaction = 0.0002 and 0.001, respectively).

### Dietary phosphatidylcholine and incident diseases

For the associations of dietary phosphatidylcholine intake with incident CVD and incident coronary artery disease, the overall results were not significant (**Supplemental Table 1**).

## DISCUSSION

In the current prospective study in 80,978 women and 39,434 men who were followed for up to 32 y, we observed that higher habitual dietary intakes of phosphatidylcholine were associated with an increased risk of all-cause mortality, especially CVD-specific mortality. Such associations were independent of traditional CVD risk factors.

Phospholipid phosphatidylcholine is a major dietary source of choline and trimethylamine, which are metabolized into circulating TMAO via intestinal microbiota-dependent mechanisms (1, 2, 25). Compelling evidence suggests that circulating TMAO might advance atherosclerosis by disturbing the clearance of cholesterol in the liver (3), and higher blood TMAO concentrations have been related to an increased risk of CVD and mortality (1, 25). Other putative mechanisms may involve upregulation of macrophage scavenger receptors, augmented macrophage cholesterol accumulation, and foam cell formation, which result in increased inflammation and oxidation of LDL cholesterol (1).

Our results are consistent with previous findings that suggest an association between circulating metabolites of dietary phosphatidylcholine and increased risk of CVD (1, 3). Wang et al. (1) found that plasma concentrations of metabolites (including TMAO) from dietary phosphatidylcholine via a gut microbiota–related nutrient metabolism pathway were highly predictive of CVD risk. Furthermore, elevated circulating TMAO concentrations were also associated with an increased risk of major cardiovascular events, including mortality, in another larger cohort study (3). Our study extends these findings by suggesting that the dietary sources of such gut microbiota–related metabolites may also be related to mortality. Our previous results (4) showed that participants in the top quintile of dietary phosphatidylcholine intakes had a 34% (95% CI: 27%, 44%) higher risk of type 2 diabetes than those in the bottom quintile intakes. Taken together with these findings, our data suggest that nutrient pathways via gut microbiota metabolites are likely involved in other clinical outcomes, such as diabetes and mortality.

Although, to our knowledge, no epidemiologic study has assessed the association of dietary phosphatidylcholine intake with cardiovascular and mortality risk thus far, several studies have reported inconsistent associations between dietary intakes of total

**TABLE 4**
HRs (95% CIs) of overall and CVD-specific mortality by quintile of energy-adjusted cumulative dietary choline from phosphatidylcholine by diabetes status[1]

| | Quintile of choline intake from phosphatidylcholine | | | | | |
|---|---|---|---|---|---|---|
| | 1 (low) | 2 | 3 | 4 | 5 (high) | *P*-trend |
| Overall mortality | | | | | | |
| Patients with diabetes | | | | | | |
| Median intake, mg/d | 131.8 | 158.4 | 177.8 | 199.4 | 246.2 | |
| Age-adjusted | 1 | 0.93 (0.79, 1.09) | 0.98 (0.84, 1.15) | 1.15 (0.99, 1.33) | 1.31 (1.14, 1.50) | <0.0001 |
| Multivariate model 1[2] | 1 | 1.06 (0.90, 1.25) | 1.07 (0.91, 1.25) | 1.25 (1.08, 1.45) | 1.27 (1.10, 1.46) | <0.0001 |
| Multivariate model 2[3] | 1 | 1.06 (0.89, 1.25) | 1.06 (0.90, 1.24) | 1.24 (1.06, 1.44) | 1.24 (1.08, 1.44) | 0.0003 |
| Nondiabetics | | | | | | |
| Median intake, mg/d | 130.2 | 152.1 | 168.2 | 188.6 | 234.1 | |
| Age-adjusted | 1 | 0.98 (0.93, 1.04) | 0.99 (0.94, 1.05) | 1.08 (1.03, 1.14) | 1.29 (1.23, 1.36) | <0.0001 |
| Multivariate model 1[2] | 1 | 1.03 (0.98, 1.09) | 1.03 (0.98, 1.09) | 1.07 (1.02, 1.13) | 1.12 (1.07, 1.18) | <0.0001 |
| Multivariate model 2[3] | 1 | 1.02 (0.97, 1.08) | 1.02 (0.96, 1.07) | 1.05 (1.00, 1.11) | 1.09 (1.04, 1.14) | <0.0001 |
| CVD mortality | | | | | | |
| Patients with diabetes | | | | | | |
| Median intake, mg/d | 131.8 | 158.4 | 177.8 | 199.4 | 246.2 | |
| Age-adjusted | 1 | 1.13 (0.83, 1.54) | 1.20 (0.89, 1.62) | 1.37 (1.04, 1.82) | 1.77 (1.36, 2.31) | <0.0001 |
| Multivariate model 1[2] | 1 | 1.28 (0.93, 1.76) | 1.32 (0.97, 1.79) | 1.50 (1.12, 2.00) | 1.73 (1.31, 2.27) | <0.0001 |
| Multivariate model 2[3] | 1 | 1.27 (0.92, 1.75) | 1.30 (0.96, 1.76) | 1.46 (1.10, 1.96) | 1.67 (1.26, 2.20) | 0.0001 |
| Nondiabetics | | | | | | |
| Median intake, mg/d | 130.2 | 152.1 | 168.2 | 188.6 | 234.1 | |
| Age-adjusted | 1 | 1.06 (0.95, 1.18) | 1.03 (0.92, 1.15) | 1.1 (0.99, 1.23) | 1.42 (1.28, 1.57) | <0.0001 |
| Multivariate model 1[2] | 1 | 1.08 (0.96, 1.20) | 1.02 (0.91, 1.14) | 1.04 (0.93, 1.16) | 1.22 (1.10, 1.35) | 0.0002 |
| Multivariate model 2[3] | 1 | 1.07 (0.96, 1.19) | 1.00 (0.90, 1.12) | 1.03 (0.92, 1.14) | 1.19 (1.07, 1.31) | 0.001 |

[1]There were 12,769 diabetic participants and 110,630 nondiabetic participants included in this analysis (a participant could contribute to the analysis among the nondiabetics before he or she developed diabetes and then contribute to the analysis among patients with diabetes). *P*-interaction values for overall mortality and for CVD mortality were 0.0002 and 0.001, respectively. CVD, cardiovascular disease; HPFS, Health Professionals Follow-Up Study; HRT, hormone replacement therapy; NHS, Nurses' Health Study.

[2]Model 1 adjusted for the following: age (mo); BMI (kg/m$^2$); white race (yes or no); marital status (married or single); menopausal status and postmenopausal HRT [premenopausal, postmenopausal + HRT nonuser, postmenopausal +current HRT user, postmenopausal + past HRT user, or missing (women only)]; family history of CVD (yes or no); smoking status and smoking pack-years (never; past smoker: ≤5 pack-years, 6–20 pack-years, or ≥21 pack-years; current smoker: ≤5 pack-years, 6–20 pack-years, or ≥21 pack-years); alcohol consumption (0, 0.1–4.9, 5.0–14.9, or ≥15.0 g/d for women and 0, 0.1–4.9, 5.0–29.9, or ≥30.0 g/d for men); physical activity (in quintiles, a category of missing values); the presence of diabetes, hypertension, or hypercholesterolemia (yes or no for each); and regular aspirin use (yes or no).

[3]Model 2 further adjusted for dietary intakes of energy and *trans* fat and the ratio of polyunsaturated to saturated fat (quintiles).

choline and the risk of coronary artery disease (26–28). Phosphatidylcholine is the major dietary source of choline (20), contributing to ∼54% of choline intake in both of our cohorts. Various choline sources might differentially affect CVD and mortality, although our data show that phosphatidylcholine is likely to be the nutrient of the most importance.

The associations of dietary phosphatidylcholine with all-cause and CVD-specific mortality were stronger among diabetic than in nondiabetic participants, although in both populations such associations were all significant. Our observation is in line with the previous finding that the association of increased blood TMAO concentrations with CVD risk appeared to be stronger in diabetic patients than in those without diabetes (1). The associations of dietary phosphatidylcholine with all-cause and CVD-specific mortality appeared to be consistent in both men and women. This observation is in line with a previous report that the associations of TMAO concentrations with composite risk of incident CVD and mortality were comparable, in general, between sexes (3). The extreme quintile HR of CVD-specific mortality appeared to be slightly stronger than that of all-cause mortality in the combined results, which is in accordance with our hypothesis that the effects of dietary phosphatidylcholine on mortality are mainly through its effects on CVD. Our results on incident CVD and incident coronary artery disease analyses showed attenuated and nonsignificant associations compared with those from the mortality analyses, suggesting that the effects of dietary phosphatidylcholine intake may be stronger on CVD prognosis than on CVD development.

To our knowledge, our study is the first large prospective study to assess the association of dietary phosphatidylcholine intakes with the risk of all-cause and CVD-specific mortality in general US populations and, in particular among diabetic patients, who have a higher risk of CVD and premature mortality than the general population. The strengths of our study include the large sample size, long-term follow-up, the specific diabetic study population, and detailed information on dietary and lifestyle factors. Furthermore, we used cumulative average dietary measurements of phosphatidylcholine intake with the use of repeated FFQ data to reduce measurement errors and to capture long-term dietary exposure.

We acknowledge several limitations of our study. First, similar to other observational studies, we could not show causality, although reverse causality is less likely when mortality is analyzed as the outcome. Second, we did not have a direct measure of circulating TMAO or the correlation of dietary phosphatidylcholine with circulating TMAO in our study. However, previous

studies in humans and in mice consistently indicated close correlations (1, 3). Third, higher intakes of phosphatidylcholine-containing foods, such as eggs and red meat, may be correlated with a cluster of unhealthier dietary and lifestyle habits (e.g., more smoking, less physical activity, and a higher BMI). Although we adjusted for the lifestyle and dietary factors known to be associated with CVD and mortality, the potential of uncontrolled and unmeasured confounders might still remain. In addition, blood homocysteine concentrations might be involved in our observed association between phosphatidylcholine intakes and mortality. However, we were not able to explore this hypothesis in the current analysis, because we had measurements of circulating homocysteine concentrations in only a small sample of our population (29). Furthermore, because the present study was conducted in mainly whites, the associations need to be examined in other race/ethnic groups.

In conclusion, we found that a high intake of phosphatidylcholine, which could lead to a higher production of TMAO, was significantly associated with an increased risk of all-cause and CVD-specific mortality, in particular among diabetic patients.

The authors' responsibilities were as follows—YZ: designed the study, analyzed the data, and wrote the manuscript; YL: analyzed the data and edited and reviewed the manuscript; EBR, FBH, CMA, KMR, and JEM: contributed to the discussion and edited and reviewed the manuscript; and LQ: designed the study, reviewed the data, contributed to the discussion, edited and reviewed the manuscript, and is the guarantor of this work and, as such, had full access to all the data in the study and took responsibility for the integrity of the data and the accuracy of the data analysis. The authors had no conflicts of interest to disclose.

## REFERENCES

1. Wang Z, Klipfell E, Bennett BJ, Koeth R, Levison BS, Dugar B, Feldstein AE, Britt EB, Fu X, Chung YM, et al. Gut flora metabolism of phosphatidylcholine promotes cardiovascular disease. Nature 2011;472:57–63.
2. Bennett BJ, de Aguiar Vallim TQ, Wang Z, Shih DM, Meng Y, Gregory J, Allayee H, Lee R, Graham M, Crooke R, et al. Trimethylamine-N-oxide, a metabolite associated with atherosclerosis, exhibits complex genetic and dietary regulation. Cell Metab 2013;17:49–60.
3. Tang WH, Wang Z, Levison BS, Koeth RA, Britt EB, Fu X, Wu Y, Hazen SL. Intestinal microbial metabolism of phosphatidylcholine and cardiovascular risk. N Engl J Med 2013;368:1575–84.
4. Li Y, Wang DD, Chiuve SE, Manson JE, Willett WC, Hu FB, Qi L. Dietary phosphatidylcholine intake and type 2 diabetes in men and women. Diabetes Care 2015;38:e13–4.
5. Almdal T, Scharling H, Jensen JS, Vestergaard H. The independent effect of type 2 diabetes mellitus on ischemic heart disease, stroke, and death: a population-based study of 13,000 men and women with 20 years of follow-up. Arch Intern Med 2004;164:1422–6.
6. Seshasai SR, Kaptoge S, Thompson A, Di Angelantonio E, Gao P, Sarwar N, Whincup PH, Mukamal KJ, Gillum RF, Holme I, et al. Diabetes mellitus, fasting glucose, and risk of cause-specific death. N Engl J Med 2011;364:829–41.
7. Willett WC, Green A, Stampfer MJ, Speizer FE, Colditz GA, Rosner B, Monson RR, Stason W, Hennekens CH. Relative and absolute excess risks of coronary heart disease among women who smoke cigarettes. N Engl J Med 1987;317:1303–9.
8. Hu FB, Willett WC. Diet and coronary heart disease: findings from the Nurses' Health Study and Health Professionals' Follow-up Study. J Nutr Health Aging 2001;5:132–8.
9. Colditz GA, Manson JE, Hankinson SE. The Nurses' Health Study: 20-year contribution to the understanding of health among women. J Womens Health 1997;6:49–62.
10. Rimm EB, Giovannucci EL, Willett WC, Colditz GA, Ascherio A, Rosner B, Stampfer MJ. Prospective study of alcohol consumption and risk of coronary disease in men. Lancet 1991;338:464–8.
11. National Diabetes Data Group. Classification and diagnosis of diabetes mellitus and other categories of glucose intolerance. Diabetes 1979;28:1039–57.
12. Expert Committee on the Diagnosis and Classification of Diabetes Mellitus. Report of the Expert Committee on the Diagnosis and Classification of Diabetes Mellitus. Diabetes Care 1997;20:1183–97.
13. Manson JE, Rimm EB, Stampfer MJ, Colditz GA, Willett WC, Krolewski AS, Rosner B, Hennekens CH, Speizer FE. Physical activity and incidence of non-insulin-dependent diabetes mellitus in women. Lancet 1991;338:774–8.
14. Hu FB, Leitzmann MF, Stampfer MJ, Colditz GA, Willett WC, Rimm EB. Physical activity and television watching in relation to risk for type 2 diabetes mellitus in men. Arch Intern Med 2001;161:1542–8.
15. Qi L, van Dam RM, Rexrode K, Hu FB. Heme iron from diet as a risk factor for coronary heart disease in women with type 2 diabetes. Diabetes Care 2007;30:101–6.
16. Zhang WL, Lopez-Garcia E, Li TY, Hu FB, van Dam RM. Coffee consumption and risk of cardiovascular events and all-cause mortality among women with type 2 diabetes. Diabetologia 2009;52:810–7.
17. Stampfer MJ, Willett WC, Speizer FE, Dysert DC, Lipnick R, Rosner B, Hennekens CH. Test of the National Death Index. Am J Epidemiol 1984;119:837–9.
18. Rose G, Blackburn H, editors. Cardiovascular survey methods. World Health Organization monograph series no. 56. 2nd ed. Geneva (Switzerland): World Health Organization; 1982.
19. Walker AE, Robins M, Weinfeld FD. The National Survey of Stroke: clinical findings. Stroke 1981;12(Suppl 1):I13–44.
20. Patterson KY, Bhagwat SA, Williams JR, Howe JC, Holden JM. USDA Database for the Choline Content of Common Foods [Internet]. Release Two. Beltsville (MD): USDA; 2008. [cited 2016 Mar 29]. Available from: http://www.ars.usda.gov/SP2UserFiles/Place/80400525/Data/Choline/Choln02.pdf.
21. Zeisel SH, Mar MH, Howe JC, Holden JM. Concentrations of choline-containing compounds and betaine in common foods. J Nutr 2003;133:1302–7.
22. Willett W, Stampfer MJ. Total energy intake: implications for epidemiologic analyses. Am J Epidemiol 1986;124:17–27.
23. Chiuve SE, Giovannucci EL, Hankinson SE, Zeisel SH, Dougherty LW, Willett WC, Rimm EB. The association between betaine and choline intakes and the plasma concentrations of homocysteine in women. Am J Clin Nutr 2007;86:1073–81.
24. Hu FB, Stampfer MJ, Rimm E, Ascherio A, Rosner BA, Spiegelman D, Willett WC. Dietary fat and coronary heart disease: a comparison of approaches for adjusting for total energy intake and modeling repeated dietary measurements. Am J Epidemiol 1999;149:531–40.
25. Koeth RA, Wang Z, Levison BS, Buffa JA, Org E, Sheehy BT, Britt EB, Fu X, Wu Y, Li L, et al. Intestinal microbiota metabolism of L-carnitine, a nutrient in red meat, promotes atherosclerosis. Nat Med 2013;19:576–85.
26. Bidulescu A, Chambless LE, Siega-Riz AM, Zeisel SH, Heiss G. Usual choline and betaine dietary intake and incident coronary heart disease: the Atherosclerosis Risk in Communities (ARIC) Study. BMC Cardiovasc Disord 2007;7:20.
27. Dalmeijer GW, Olthof MR, Verhoef P, Bots ML, van der Schouw YT. Prospective study on dietary intakes of folate, betaine, and choline and cardiovascular disease risk in women. Eur J Clin Nutr 2008;62:386–94.
28. Detopoulou P, Panagiotakos DB, Antonopoulou S, Pitsavos C, Stefanadis C. Dietary choline and betaine intakes in relation to concentrations of inflammatory markers in healthy adults: the ATTICA study. Am J Clin Nutr 2008;87:424–30.
29. Jung S, Je Y, Giovannucci EL, Rosner B, Ogino S, Cho E. Derivation and validation of homocysteine score in u.s. Men and women. J Nutr 2015;145:96–104.
